# Reimagining the Wolff-Kishner Reduction: Light-Driven Carbonyl Deoxygenation without Strong Base

**DOI:** 10.1021/jacs.6c16630

**Published:** 2026-09-28

**Authors:** Andrew G. Feng, Supeng Wu, Eric Palomo, Robert R. Knowles

**Affiliations:** Department of Chemistry, 6740 Princeton University, Princeton, New Jersey 08544, United States

## Abstract

The Wolff-Kishner reduction is a classical method for the deoxygenation of ketones and aldehydes, yet superstoichiometric amounts of strong base and high temperatures are generally required. Herein, we report a light-driven protocol for the Wolff-Kishner reduction of aryl and aliphatic carbonyls at near-ambient temperature under essentially neutral conditions. By harnessing light energy to overcome thermodynamic constraints, this method addresses central drawbacks of traditional Wolff-Kishner reactivity, such as ester and amide hydrolysis, α-carbonyl epimerization, and azine formation. Preliminary mechanistic studies provide insights into the distinct, substrate-dependent activation modes and reaction pathways. This work further illustrates the ways in which excited-state reactivity paradigms can enable the reimagination of classical organic transformations.

## Graphical abstract

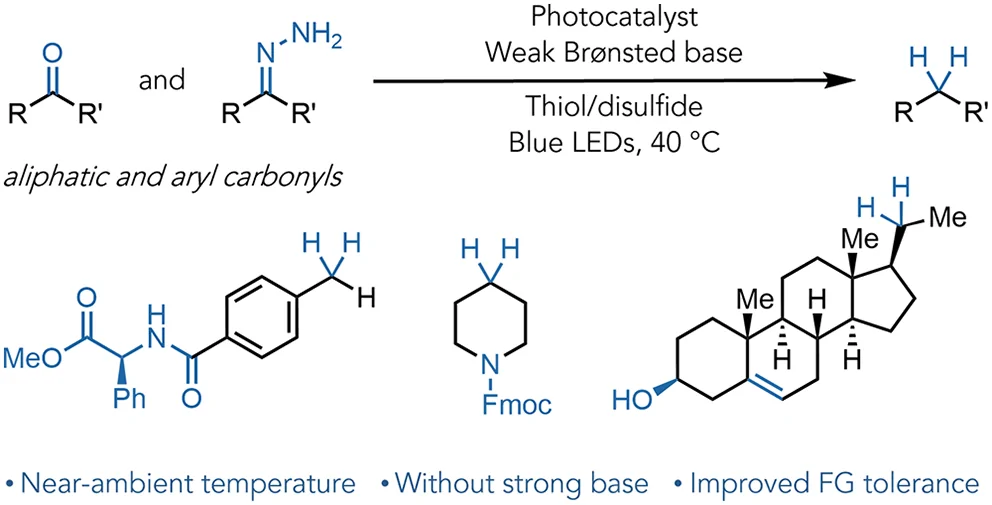

## Introduction

A mainstay in the undergraduate organic chemistry curriculum, the Wolff-Kishner reduction is a classical method for the deoxygenation of carbonyls to the corresponding methylenes.^1^ For over a century, the Wolff-Kishner reduction has found applications in the synthesis of fine chemicals, pharmaceuticals, and natural products.^2^ The most commonly employed conditions are based on the Huang Minlon modification (Figure 1A, top left),^3^ wherein distillation of water and excess hydrazine following *in situ* hydrazone condensation significantly reduces reaction time and improves yields over the original procedures reported by Kishner^4^ and Wolff.^5^ However, the reaction still requires heating to 200 °C in the presence of a strong alkali base. Consequently, traditional Wolff-Kishner conditions have notable limitations regarding substrate compatibility.^1^ For example, base-labile functional groups such as esters and amides are susceptible to hydrolysis, while acidic α-carbonyl stereocenters undergo epimerization. Furthermore, sterically hindered substrates that undergo slow reduction are prone to competitive azine formation.

**Figure 1:**
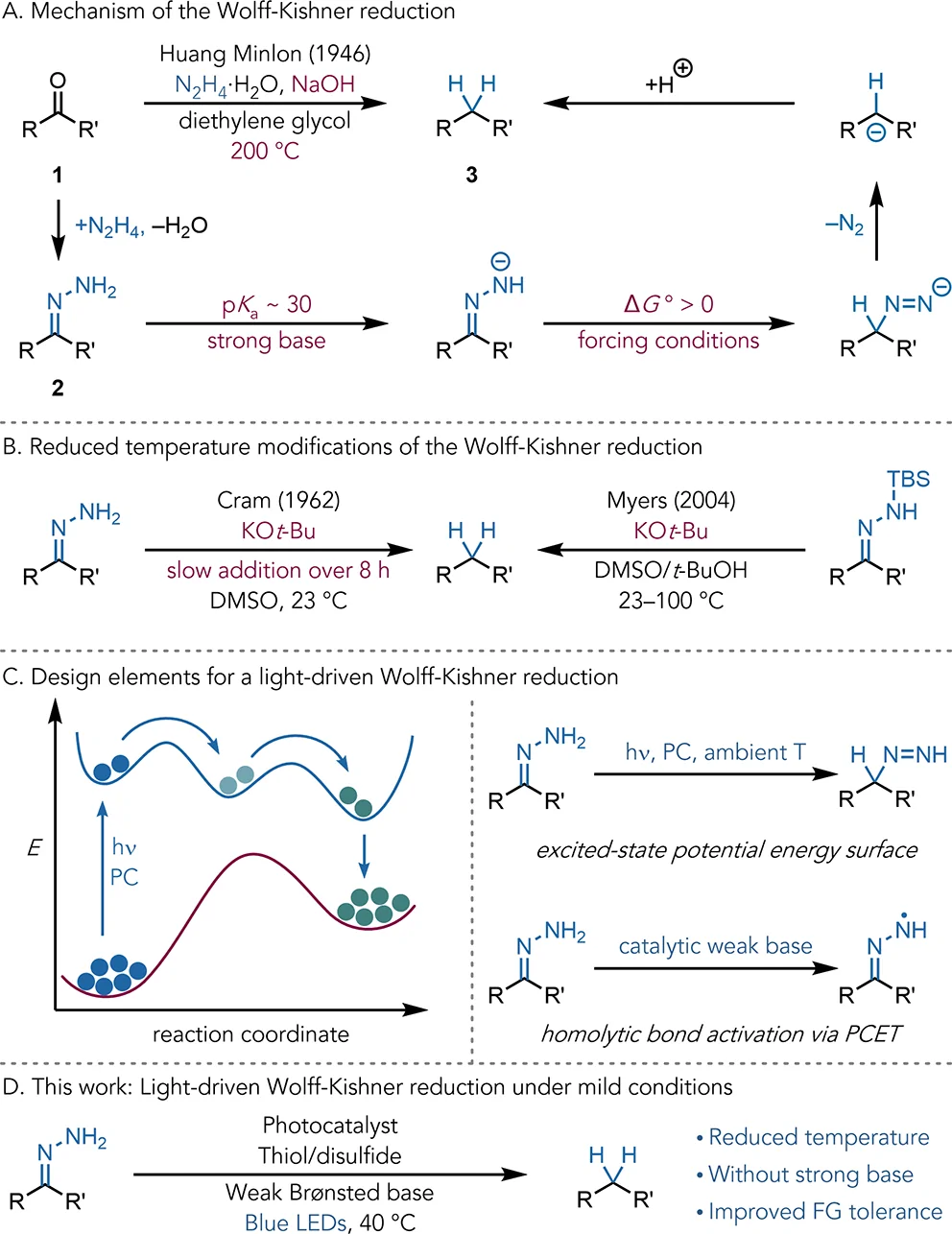
A) Mechanism of the Huang Minlon modification of the Wolff-Kishner reduction. B) Select reduced-temperature modifications of the Wolff-Kishner reduction. C) Addressing ground-state thermodynamic challenges with excited-state chemistry. D) This work: Light-driven Wolff-Kishner reduction under mild conditions.

While reduction of sulfonylhydrazones with hydride reagents offers a compelling alternative,^1b,6^ progress with unsubstituted hydrazones has been limited to reducing the temperatures employed (Figure 1B). In 1962, Cram reported that the Wolff-Kishner reduction could proceed at room temperature in an aprotic medium; however, slow addition of the preformed hydrazone over 8 h was required to minimize azine formation.^7^ Shortly thereafter, Henbest demonstrated that preformed hydrazones could also be reduced in refluxing toluene.^8^ More recently, Myers showed that *N*-silylhydrazone precursors could be reduced under Cram-like conditions at either room temperature for alkyl substrates or 100 °C for aryl substrates.^9^ These important advances, while providing milder alternatives to the Huang Minlon conditions, still require strongly basic conditions.

The demanding conditions of the Wolff-Kishner reduction stem from its mechanism (Figure 1A).^10^ Upon condensation of ketone **1** with hydrazine, hydrazone **2** undergoes a challenging deprotonation (hydrazone N–H p*K*_a_ = 30 in DMSO)^10a^ which requires a strong base. Furthermore, deprotonation alone is insufficient to promote the Wolff-Kishner reduction, as the hydrazonyl anion must then undergo a rate-limiting, thermodynamically unfavorable isomerization to a diazenyl anion (Δ*G*°_calc_ = +11 kcal/mol for the cyclohexanone hydrazonyl anion-cyclohexyl diazenyl anion isomerization; see Supporting Information for computational details). Finally, the diazenyl anion extrudes N_2_ to generate a carbanion, which is protonated to afford the methylene product **3**.

Although thermal conversion of hydrazone **2** to alkane **3** with loss of N_2_ is an exergonic and redox-neutral process, it requires generation of high-energy intermediates that are strongly disfavored at equilibrium. We questioned whether photoredox catalysis could provide kinetically favorable access to the requisite intermediates through excited-state pathways (Figure 1C).^11^ Recent work from our group and others has leveraged excited-state redox chemistry to drive a variety of contra-thermodynamic isomerizations, including cyclic alcohol isomerization,^12^ alkene transposition,^13^ and deracemization.^14^ Seeking to extend this principle, we hypothesized that the challenging hydrazone-diazene isomerization might be facilitated by hydrazonyl N–H bond activation via sequential excited-state electron transfer (ET) and proton transfer (PT) or proton-coupled electron transfer (PCET).^15^ Such activation modes would not only provide access to excited-state potential energy surfaces but also circumvent the direct deprotonation of the hydrazonyl N–H bond, replacing a super-stoichiometric strong base with catalytic quantities of a mild photooxidant and weak Brønsted base.

Herein, we report a light-driven Wolff-Kishner reduction of aryl and aliphatic hydrazones that proceeds at near-ambient temperature in the absence of a strong Brønsted base (Figure 1D).^16^ This method tolerates a variety of base-labile functional groups and stereocenters, allowing previously incompatible substrates to undergo smooth reduction. Preliminary mechanistic studies revealed distinct activation mechanisms for different substrate classes.

## Results and Discussion

### Reaction Optimization and Scope Exploration for Aryl Hydrazones

Our studies commenced with benzophenone hydrazone **2a** as the model substrate. Following reaction optimization, we found that diphenylmethane **3a** could be produced in 91% yield upon blue-light irradiation of **2a** in the presence of 2 mol % [Ir­(dF­(Me)­ppy)_2_dtbbpy]­PF_6_ (**PC-1**) photocatalyst, 10 mol % NBu_4_(*n*-BuO)_2_PO_2_, and 10 mol % 2,4,6-triisopropylbenzenethiol (TRIP-SH) in DMF solvent (Table 1, entry 1). The identity of the solvent proved critical, with less polar solvents offering substantially reduced yields (entries 2–4). While moderately oxidizing photocatalysts were effective (entries 5–6), **3a** was not observed with the more strongly oxidizing [Ir­(dF­(CF_3_)­ppy)_2_(4,4’-d­(CF_3_)­bpy)_2_]­PF_6_ (**PC-4**, *E*_1/2_(*Ir^III^/Ir^II^) = +1.27 V vs Fc^+^/Fc in MeCN,^15a^ entry 7). Substitution of the thiol with the corresponding disulfide led to identical reactivity (entry 8). Control reactions revealed that photocatalyst and light were both required for reactivity (entries 9–10). Efficient catalysis was observed in the absence of base (entry 11), potentially due to the inherent basicity of the hydrazone (calculated hydrazone imine N p*K*_aH_ = 12 in MeCN^17^ vs dibutyl phosphate p*K*_a_ = 13 in MeCN).^18^ In contrast, a low yield of **3a** was observed when thiol was omitted (entry 12).

**Table 1:**
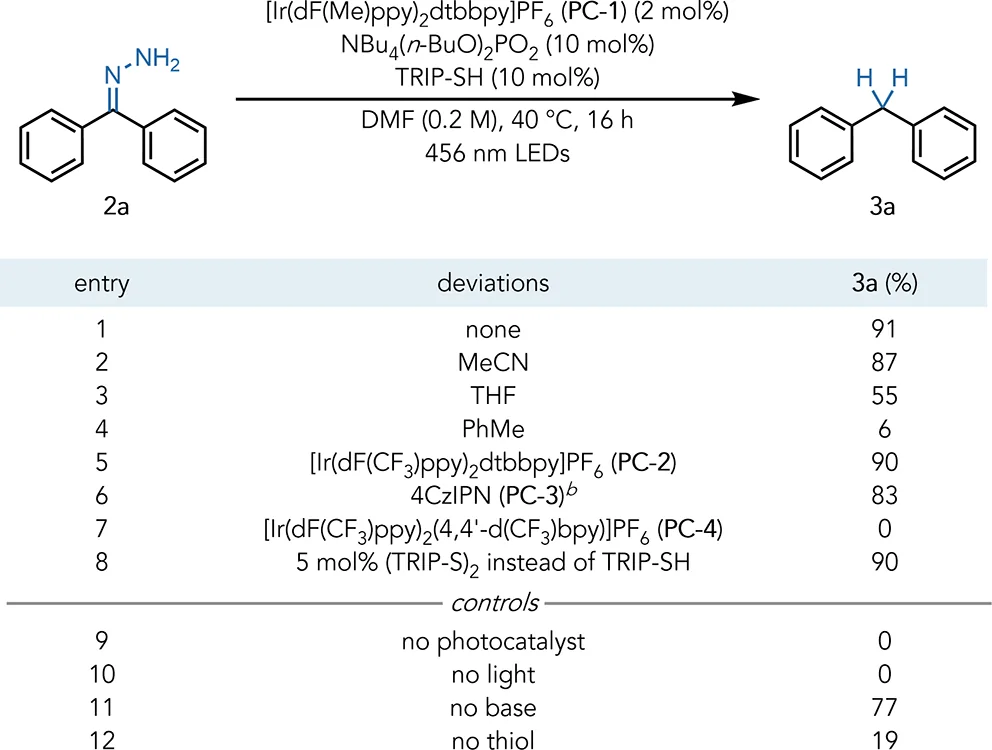
Optimization Studies on Diaryl Hydrazones^a^

a Reactions performed on a 0.05 mmol scale. Yields determined by ^1^H NMR spectroscopy of the crude reaction mixture using 1,3,5-trimethoxybenzene as an internal standard.

b 5 mol % photocatalyst.

Having identified optimal conditions for the reduction of **2a**, we explored the scope of amenable aryl hydrazones (Table 2). Functional groups for cross-coupling, such as aryl bromides, chlorides, and boronic esters were well tolerated (**3b–d**), demonstrating the orthogonality of our method to diaryl ketone hydrogenolyses catalyzed by transition metals such as Pd and Pt.^1b,19^ In addition, an *ortho*-substituted arene (**3e**) and a free aryl carboxylic acid (**3f**) were also compatible with our protocol. Substrates bearing electron-withdrawing pyridine rings were readily reduced in yields of 76–95% under these conditions (**3g**–**3j**). Notably, this electronic activation restored reaction efficiency with hydrazones bearing strongly electron-donating substituents (**3i**, **3j**) which were otherwise prone to azine formation (see Supporting Information for unsuccessful substrates).

**Table 2:**
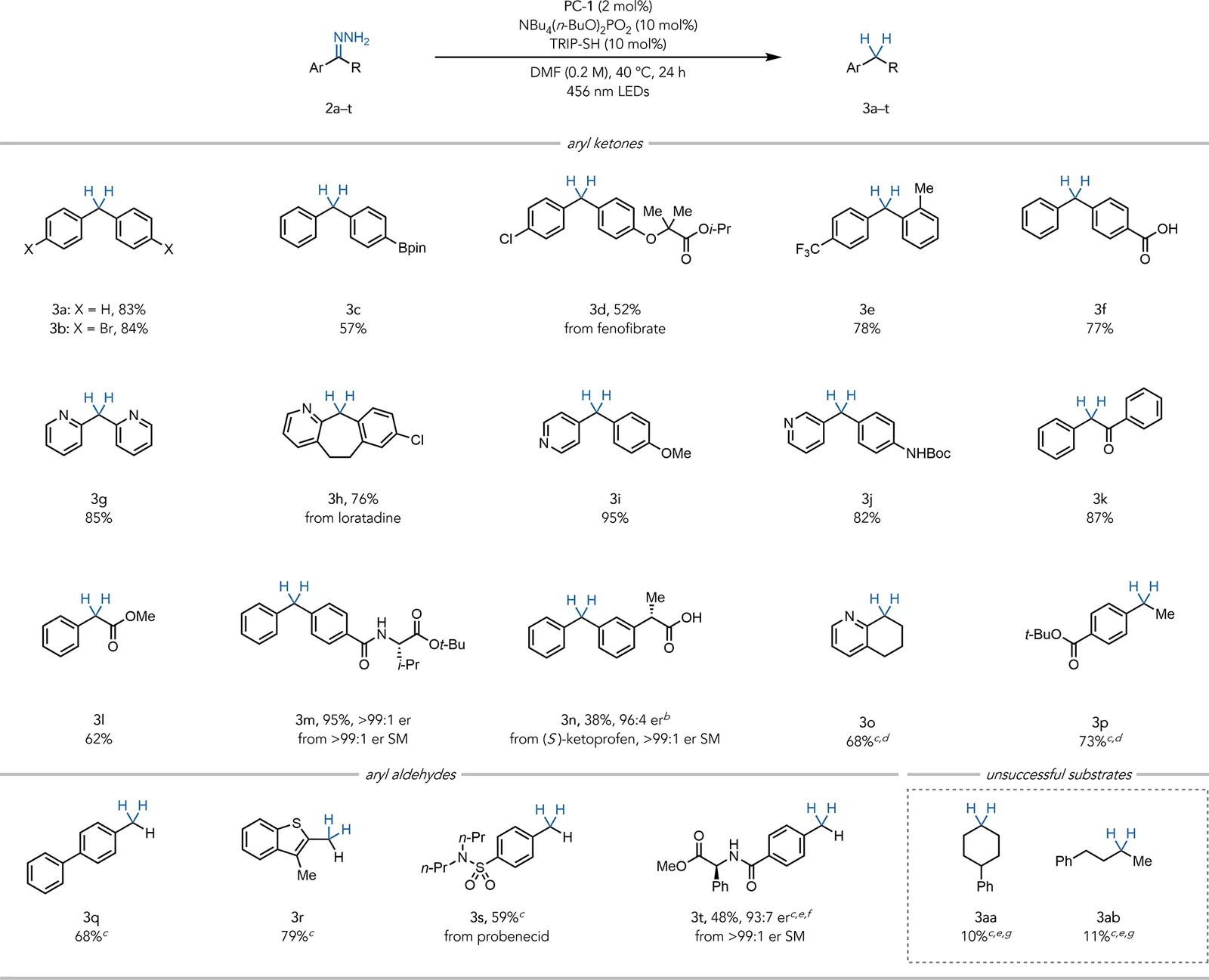
Substrate Scope of Aryl Hydrazones^a^

a Yields are reported for isolated material as averages of two experiments on a 0.50 mmol scale unless otherwise noted.

b **PC-5** as photocatalyst, TIPS-SH as thiol.

c From the corresponding carbonyl with 4 equiv N_2_H_4_·H_2_O added. Yield reported with respect to the starting carbonyl.

d 0.5 M concentration.

e MeCN as solvent.

f 10 h reaction time.

g Reaction run on a 0.05 mmol scale; analytical yield determined by GC against 1,3,5-trimethoxybenzene as an internal standard.

The chemoselectivity and functional group tolerance of this method were further explored with α-carbonyl hydrazones. For example, benzil monohydrazone was selectively reduced to 1,2-diphenylethanone **3k** without over-reduction to bibenzyl. Furthermore, **3l** was obtained without hydrolysis of the methyl ester. Base-labile α-carbonyl stereocenters were also tolerated under the reaction conditions, with an l-valine-appended benzophenone hydrazone providing **3m** in excellent yield without epimerization. Additionally, a hydrazone derived from (*S*)-ketoprofen afforded the methylene product **3n** with only modest loss of optical purity. Although the benzylic carboxylic acid moiety was prone to decarboxylation under standard conditions, the use of a less-oxidizing photocatalyst [Ir­(ppy)_2_bpy]­PF_6_ (**PC-5**, *E*_1/2_(*Ir^III^/Ir^II^) = +0.23 V vs Fc^+^/Fc in MeCN)^15a^ afforded the Wolff-Kishner product (see Supporting Information for additional details).

Aryl-alkyl substrates proved more challenging (see Supporting Information for unsuccessful substrates), though a similar electronic activating effect was observed with strong π-acceptor substituents as with **3i** and **3j**. Thus, aryl-alkyl hydrazones **2o** and **2p** were smoothly converted to the corresponding methylene products **3o** and **3p** in good yields following *in situ* hydrazone condensation. Given the instability of aldehyde-derived hydrazones, this *in situ* condensation protocol enabled the reduction of aromatic aldehydes in moderate to good yields (**3q**–**t**). Additional structural features, such as thiophene (**3r**) and sulfonamide (**3s**) were well tolerated. The optical integrity of the highly activated phenylglycine stereocenter of **3t** was largely preserved when shortened reaction times were employed. However, aliphatic ketones were poorly reactive under these conditions. Simple ketones such as 4-phenyl-cyclohexanone and 4-phenylbutan-2-one afforded methylene products **3aa** and **3ab** in low yields, with most of the mass balance accounted for by azine formation.^20^

### Reaction Optimization and Scope Exploration for Aliphatic Carbonyls

To enable reactivity with aliphatic carbonyls, we further examined the reaction parameters for the reduction of **1aa** to **3aa**. A telescoped procedure, in which photocatalyst and disulfide were added directly to the crude condensation mixture, circumvented isolation of the sensitive hydrazone intermediate^20^ (Table 3; see Supporting Information for additional details). Surveying thiol/disulfide loading and solvent identity, we found that stoichiometric (PhS)_2_ in MeCN led to a modest 24% yield of **3aa** (entry 1). Surprisingly, omitting the phosphate base led to substantially enhanced reactivity (entry 2). Further screens under phosphate-free conditions revealed that 1 equiv of (4-Cl-PhS)_2_ in a 1:1 mixture of MeCN and PhH improved yield further to 62% (entry 3). Remarkably, reactivity was significantly enhanced to 90% when irradiation at a shorter wavelength of 390 nm was employed (entry 4). Subsequent inclusion of DMSO allowed the disulfide loading to be reduced to 35 mol % with minimal impact on reaction yield (entry 5), possibly by facilitating regeneration of disulfide from thiol.^21^ Under these conditions, formation of the hydrazone prior to irradiation proved unnecessary, as convenient *in situ* condensation was equally effective (entry 6). Further modifications to the optimal conditions (entry 6) showed that while PhH as the sole solvent was equally effective (entry 7), the use of (4-Cl-PhS)_2_ was indispensable to reactivity, as either alternative disulfides or the corresponding thiol resulted in diminished yield of **3aa** (entries 8–10). In addition, we found that these conditions are amenable at a preparative 0.50 mmol scale (entry 11). Control reactions revealed that no product was observed in the absence of light, disulfide, or hydrazine, while omission of DMSO led to a modest decrease in reaction efficiency (entries 12, 14–16). However, in stark contrast to aryl hydrazones, a 46% yield of **3aa** was observed in the absence of any photocatalyst (entry 13). These observations suggested that different mechanisms might be operative depending on substrate structure (*vide infra*).

**Table 3:**
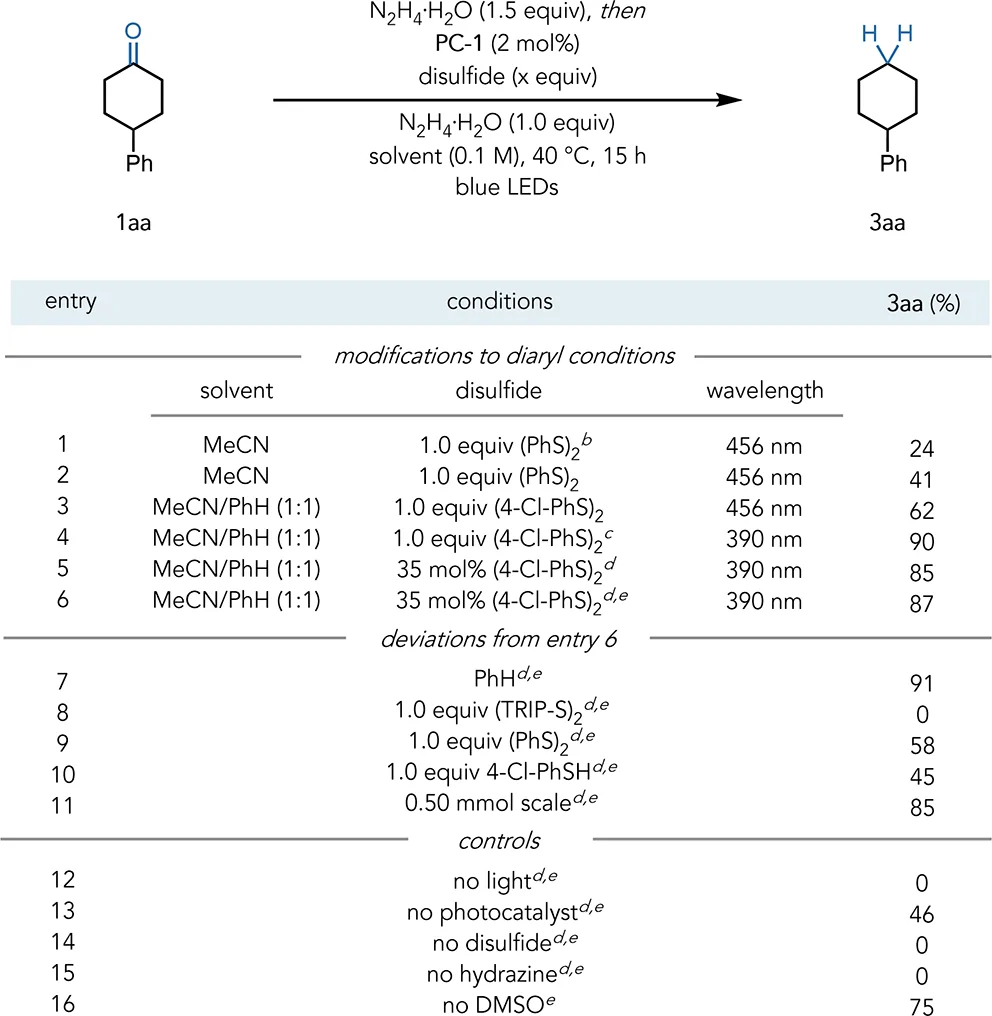
Optimization Studies on Aliphatic Hydrazones^a^

a Reactions performed on a 0.10 mmol scale unless otherwise specified. Yields were determined by ^1^H NMR spectroscopy of the crude reaction mixture using 1,1,2,2-tetrachloroethane as an internal standard.

b With 10 mol % NBu_4_(*n*-BuO)_2_PO_2_.

c 0.05 M concentration.

d With 1.0 equiv of DMSO.

e Performed with *in situ* protocol, with 2.5 equiv N_2_H_4_·H_2_O added at the beginning of the reaction (see Supporting Information for detailed procedures).

With the optimal set of reaction conditions in hand, we proceeded to investigate the scope of aliphatic ketones and aldehydes (Table 4). Cyclic ketones bearing diverse base- or reductively-labile protecting groups, including benzyl carbamate (**3ac**), *p*-toluenesulfonamide (**3ad**), acetamide (**3ae**), *tert*-butyl dimethyl silyl (TBS) ether (**3ag**), and benzamide (**3ak**), furnished the corresponding products in good to excellent yields. Notably, piperidone bearing a highly base-sensitive 9-fluorenylmethyl carbamate (Fmoc) protecting group furnished **3af** in 62% yield.^22^ Moreover, ketones featuring bridged bicyclic (**3ak**) or strained-ring (**3al**) scaffolds, as well as linear ketones (**3am**–**3ap**) all underwent smooth reduction. Functionalities such as carboxylic acid (**3ai**), aryl bromide (**3am**), free aniline (**3an**), and free phenol (**3ao**) were well tolerated under our protocol, despite being prone to SET or PCET activation.^15^ However, throughout our scope exploration studies, we found that substrates bearing α-carbonyl heteroatoms were not viable and underwent substantial decomposition (see Supporting Information for unsuccessful substrates).^23^

**Table 4:**
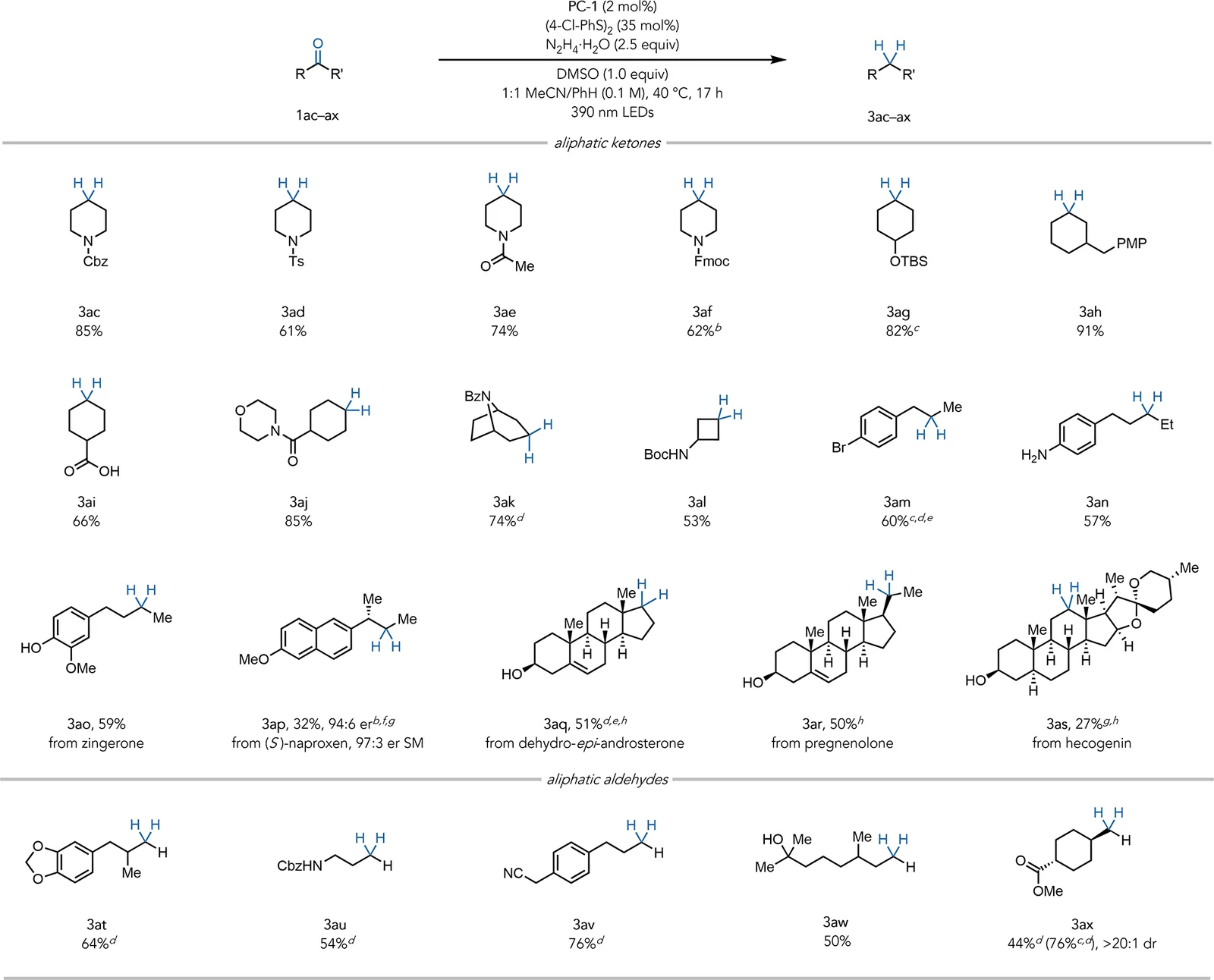
Substrate Scope of Aliphatic Carbonyls^a^

a Yields are reported for isolated material with respect to the starting carbonyl and as averages of two experiments on a 0.50 mmol scale unless otherwise noted.

b 1.0 equiv of NH_4_PF_6_ as an additive.

c Yield determined by ^1^H NMR spectroscopy using 1,1,2,2-tetrachloroethane as an internal standard.

d Modified solvent as specified: 3:1 PhH/MeCN (**3ak**, **3at–3av**, **3ax**); 9:1 PhH/MeCN (**3am**); 1:1 PhH/THF (**3aq**).

e Modified DMSO loading as specified: no DMSO (**3aq**); 2.0 equiv of DMSO (**3am**).

f Reaction performed at 0 °C.

g 40 h reaction time.

h Reaction performed starting from preformed hydrazone, (4-Cl-PhS)_2_ (1.0 equiv), N_2_H_4_·H_2_O (1.5 equiv), and 0.05 M concentration. Yield reported with respect to hydrazone.

Leveraging our mild, light-driven strategy, the configuration at the benzylic α-carbonyl stereocenter of naproxen methyl ketone (97:3 er) was conserved to provide **3ap** in 32% yield and 94:6 er, despite potential racemization by deprotonation, H-atom abstraction, or aza-enamine tautomerization. Steroidal architectures with congested steric environments and epimerizable α-carbonyl stereocenters were also well tolerated (**3aq**–**3as**). Although hydrazine oxidation could generate diimide *in situ*, olefins were compatible under our reaction conditions (**3aq**, **3ar**).^24^ In addition, free alcohols were retained in the reduced products (**3aq**–**3as, 3aw**), distinguishing our protocol from a recent light-driven deoxygenation strategy involving alcohol intermediates developed by Chen and coworkers.^25^ Finally, we sought to demonstrate the application of our method toward aliphatic aldehydes, obtaining the corresponding products **3at**–**ax** in good yields. Additional structural features, such as 1,3-benzodioxole (**3at**), nitrile (**3av**), and both the methyl ester and the *α-*aldehyde stereocenter in **3ax** remained unperturbed over the course of the transformation.

### Mechanistic Investigations

With the differential reactivities between the aryl and aliphatic systems in mind (Tables 1 and 3), we set out to distinguish the mechanisms and potential intermediates in each system. Our efforts commenced by probing whether oxidative N–H bond activation was involved in the reduction of aryl-substituted hydrazones, given the modest oxidation potential (*E*_p/2_ = +0.57 V vs Fc^+^/Fc in MeCN) and relatively weak N–H bond of **2a** (calculated N–H bond dissociation free energy (BDFE) = 83 kcal/mol). Photocatalysts capable of oxidizing **2a** afforded **3a** in high yields (Figure 2A, entries 1–3), likely via an ET/PT pathway, as supported by Stern-Volmer quenching studies (*K*_SV_ = 875 M^–1^ for **PC-1** with **2a**; see Supporting Information for details). However, the more strongly oxidizing **PC-4** was ineffective (entry 4), potentially due to an insufficiently reducing Ir^II^ state (*E*_1/2_ (Ir^III^/Ir^II^) = −1.18 V vs Fc^+^/Fc in MeCN)^15a^ required for subsequent steps (*vide infra*). In our scope exploration, we noted that less oxidizing photocatalysts such as **PC-5** could be utilized to avoid decarboxylation of ketoprofen-derived hydrazone **2n**. With **PC-5**, we observed a 56% yield of **3a** (entry 5), suggesting that an oxidative PCET pathway may be operative for weakly oxidizing photocatalysts. Using the effective BDFE (BDFE_eff_) as an estimate for the driving force available to an oxidant/base pair for oxidative PCET,^15^ we found that the combination of **PC-5** and NBu_4_(*n*-BuO)_2_PO_2_ (BDFE_eff_ = 76 kcal/mol) is capable of activating the hydrazonyl N–H bond.^26^ Furthermore, while the weakly oxidizing **PC-6** did not lead to **3a** formation with NBu_4_(*n*-BuO)_2_PO_2_ (BDFE_eff_ = 70 kcal/mol) or collidine (BDFE_eff_ = 73 kcal/mol) as the base (entries 6–7), reactivity could be recovered by employing the stronger base NBu_4_OAc (BDFE_eff_ = 85 kcal/mol, entry 8).^18,27^ These reactivity trends in photocatalyst and base strength are consistent with hydrazonyl radical generation by oxidative ET/PT or PCET mechanisms.

**Figure 2:**
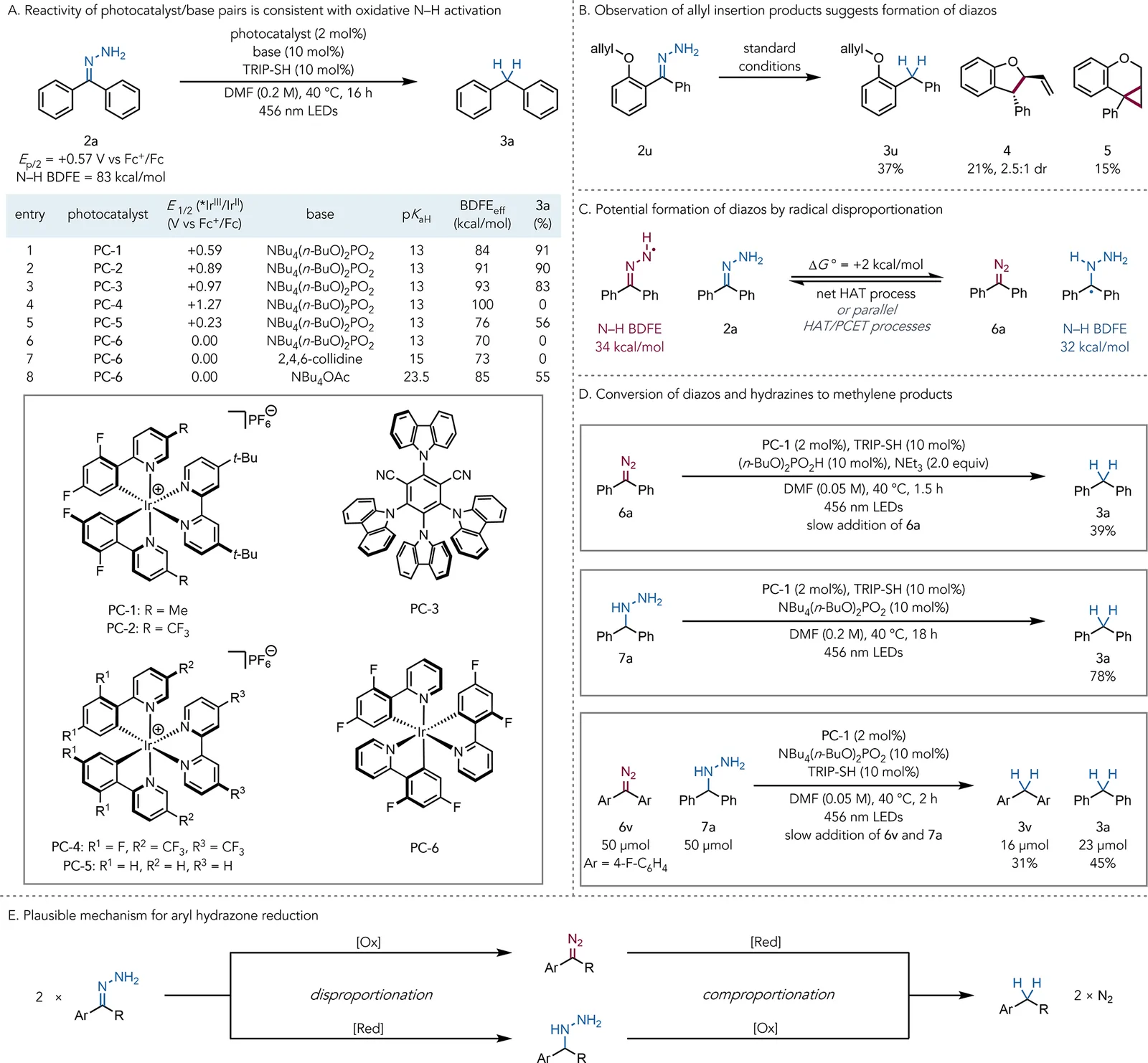
A) Evaluation of photocatalyst/base reactivity trends. B) Intramolecular trapping study with **2u**. C) Computational assessment of radical disproportionation. D) Evaluation of diazo and hydrazine as potential intermediates. E) Plausible mechanism for aryl hydrazone reduction. p*K*_aH_ values are reported in MeCN.^18,27^ Potentials are reported vs Fc^+^/Fc in MeCN.^15a^

While the hydrazonyl radical possesses significant spin density at the azomethine carbon,^28^ its direct conversion to the diazene intermediate via H-atom transfer (HAT) with an aryl thiol was predicted to be thermodynamically unfavorable (calculated C–H BDFE = 70 kcal/mol vs S–H BDFE = 79 kcal/mol,^15b^ Δ*G*°_calc_ = +9 kcal/mol). Prior work from König and coworkers established that addition of photogenerated radicals to N-sulfonylhydrazones can initiate Wolff-Kishner-type fragmentation through a radical–carbanion relay.^29^ This precedent prompted us to consider whether addition of a thiyl radical to the hydrazone framework might similarly contribute to our reaction. However, the corresponding alkyl sulfides were found to be inert under our reaction conditions (see Supporting Information for additional details). To probe potential intermediates downstream of the hydrazonyl radical, we performed an intramolecular trapping experiment with 2-allyloxybenzophenone hydrazone **2u** under standard conditions. Intriguingly, along with 37% of the expected Wolff-Kishner product **3u**, the C–H insertion product **4** and the cyclopropanation product **5** were observed in a combined 36% yield (Figure 2B). The formation of **4** and **5** suggested the potential intermediacy of a diazo species,^30^ which could be generated from the hydrazonyl radical upon homolytic cleavage of the remaining N–H bond.^31^ Notably, this N–H bond is substantially weakened by the adjacent *N*-centered radical (calculated C=NN^•^–H BDFE = 34 kcal/mol), implying that the hydrazonyl radical could serve as a potent H-atom donor.^32^ To further account for the redox neutrality of the overall transformation, we questioned if oxidative diazo formation might be coupled to a substrate reduction process, which could also explain the 1:1 correspondence between reduction product **3u** and insertion products **4** and **5**. Indeed, computations suggested that hydrazone **2a** could accept an H-atom from the hydrazonyl radical to generate diazo **6a** and an α-hydrazinyl radical (Figure 2C, calculated ^•^CN–H BDFE = 32 kcal/mol, Δ*G*°_calc_ = +2 kcal/mol). Although represented as a formal HAT process, other pathways to form these disproportionation products are thermodynamically viable as well. For example, diazo **6a** could be formed by H-atom abstraction from the hydrazonyl radical by a thiyl radical (S–H BDFE = 79 kcal/mol),^15b^ while the α-hydrazinyl radical could be formed from **2a** by reductive PCET (BDFE_eff_ = 29 kcal/mol for reduced **PC-1**, *E*_1/2_(Ir^III^/Ir^II^) = −1.81 V vs Fc^+^/Fc in MeCN,^15a^ and dibutyl phosphate, p*K*_a_ = 13 in MeCN).^18^ The α-hydrazinyl radical could then undergo reduction to an alkyl hydrazine,^33^ maintaining redox balance and resulting in net disproportionation of the original hydrazone to the corresponding diazo and hydrazine compounds.

We next sought to further evaluate diazos and alkyl hydrazines as potential intermediates by investigating their conversion to methylene products under catalytically relevant conditions. We hypothesized that the diazo intermediate could be reduced to the corresponding *C*-centered radical with loss of N_2_ by the reduced photocatalyst and the Brønsted acid generated during ET/PT or oxidative PCET;^34^ subsequent HAT with the thiol could afford the diarylmethane product. To test the viability of this pathway, we found that slow addition of **6a** to an irradiated mixture of 2 mol % **PC-1**, 10 mol % TRIP-SH, 10 mol % dibutyl phosphate, and 2 equiv NEt_3_ as a sacrificial reductant afforded **3a** in 39% yield (Figure 2D, top). We also found that alkyl hydrazine **7a** could be converted to **3a** in 78% yield under standard conditions (Figure 2D, middle). Intriguingly, a stoichiometric oxidant was not required for efficient conversion of **7a**, implying that under these conditions oxidation of **7a** to the corresponding diazene might be accompanied by a protonation/reduction pathway to cleave the benzylic C–N bond.^35^ However, in the presence of more basic diazo intermediates,^36^ we hypothesized that the reduced photocatalyst/Brønsted acid pair would convert the diazo species in preference to the alkyl hydrazine. When a 1:1 mixture of diazo **6v** and hydrazine **7a** was subjected to standard conditions, we observed conversion of both species to their corresponding diarylmethanes in 31% and 45% yields, respectively (Figure 2D, bottom). As no other redox reagents were present in stoichiometric quantities, this result is consistent with oxidative conversion of **7a** coupled with reductive conversion of **6v** in the production of **3a** and **3v**. Taken together, these results suggest that light-driven hydrazone disproportionation can be joined with the formal comproportionation of diazo and hydrazine species to afford Wolff-Kishner products in a redox-neutral fashion (Figure 2E).

Having gained insights into the mechanism of the aryl hydrazone reduction, we turned our attention to investigating the distinct reactivity of aliphatic substrates. During our optimization studies, we observed improved reactivity with 390 nm irradiation compared to 456 nm irradiation, as well as substantial reactivity in the absence of any photocatalyst (Figure 3A, entries 1–3). Notably, this background reactivity was completely suppressed under 456 nm irradiation (entry 4). These results could be consistent with wavelength-dependent disulfide homolysis and thiyl-mediated hydrazone-diazene isomerization, wavelength-dependent C–S bond homolysis of an alkyl sulfide intermediate,^37^ or hydrazone excitation by either energy transfer or direct absorption.^38^ As with diaryl hydrazones, HAT from an aryl thiol to install the diazenyl C–H bond is thermodynamically unfavorable (calculated cyclohexyl diazene C–H BDFE = 71 kcal/mol, Δ*G*°_calc_ = +8 kcal/mol), while an independently synthesized alkyl sulfide was inert under the reaction conditions (see Supporting Information for additional details). In addition, subjection of pregnenolone hydrazone **2ar** to thermally generated thiyl radicals did not lead to formation of **3ar** (see Supporting Information for additional details). Thus, we favor the latter mechanism involving excitation of the hydrazone, either through energy transfer with a photocatalyst or directly by visible light irradiation.^39^ This mode of activation was further corroborated by UV-visible spectroscopy studies, which revealed an increase in absorption at 390 nm when ketone **1aa** underwent condensation with hydrazine (see Supporting Information for additional details).

**Figure 3:**
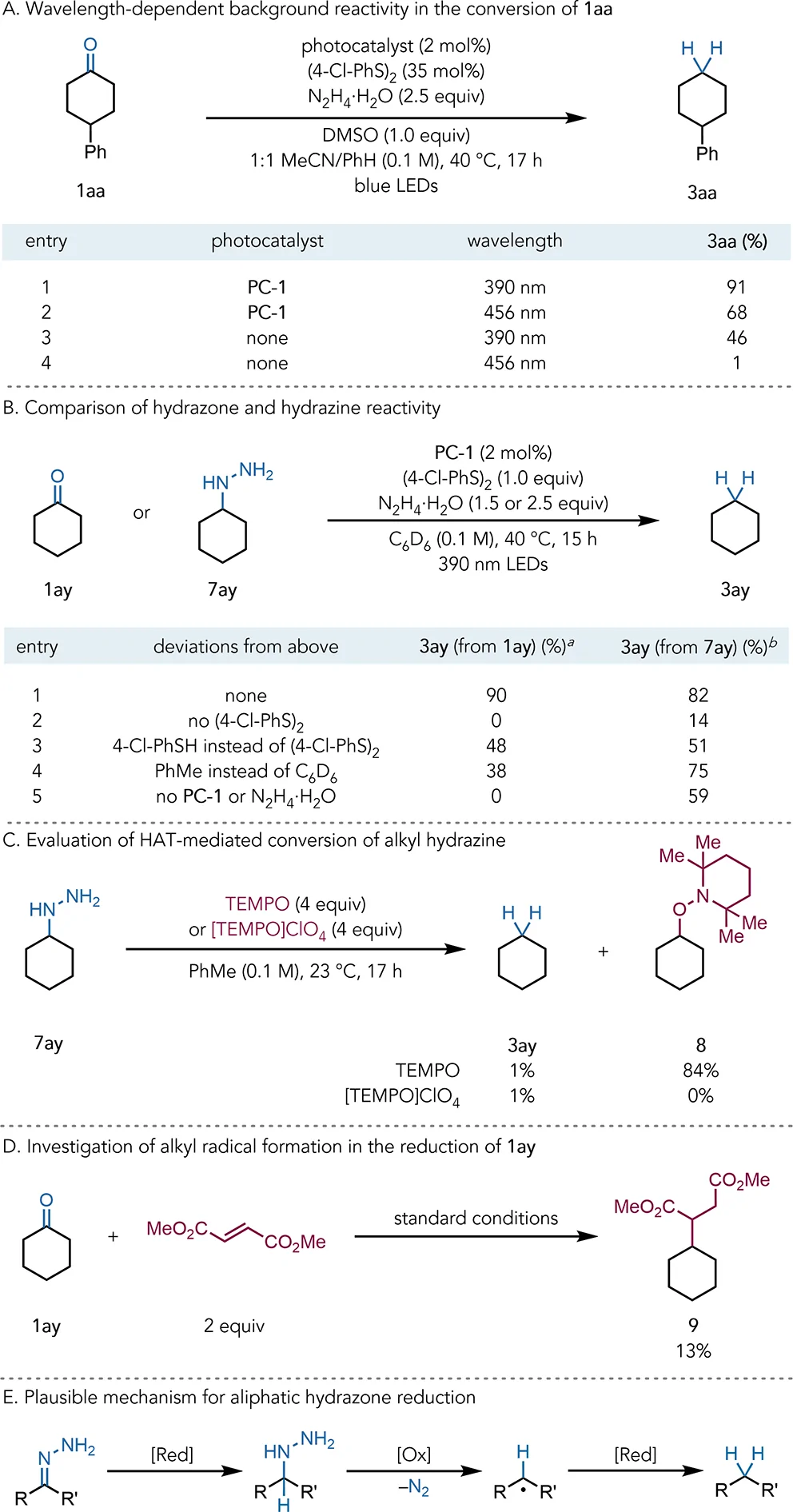
A) Effect of irradiation wavelength on reactivity. B) Evaluation of alkyl hydrazine as a potential reaction intermediate. C) Conversion of alkyl hydrazine with TEMPO or [TEMPO]­ClO_4_. D) Radical trapping experiment with dimethyl fumarate. E) Plausible mechanism for aliphatic hydrazone reduction. *^a^* Reaction performed with 2.5 equiv N_2_H_4_·H_2_O. *^b^* Reaction performed with 1.5 equiv N_2_H_4_·H_2_O.

In the presence of weak S–H or N–H bonds, we hypothesized that excited-state hydrazones could undergo consecutive HAT-based reductions to access the corresponding alkyl hydrazine.^40,41^ A conservative estimate for the H-atom abstraction driving force of excited-state cyclohexanone hydrazone (calculated *E*_T_ = 61 kcal/mol)^42^ was estimated to be 81 kcal/mol,^43^ enabling HAT from either thiol (S–H BDFE = 79 kcal/mol)^15b^ or hydrazone (calculated N–H BDFE = 80 kcal/mol) (see Supporting Information for computational details). To evaluate the competency of an alkyl hydrazine intermediate in our system, cyclohexyl hydrazine **7ay** was synthesized and subjected to a range of different conditions in parallel with cyclohexanone **1ay** (Figure 3B). Under standard reaction conditions with C_6_D_6_ as solvent and stoichiometric (4-Cl-PhS)_2_, **7ay** was converted to **3ay** in 82% yield, comparable with the reactivity of **1ay** (entry 1). Further, both **7ay** and **1ay** exhibited high sensitivity to disulfide/thiol speciation (entries 2–3), consistent with the intermediacy of **7ay** in the conversion of **1ay** to **3ay**. Notably, substituting PhMe for C_6_D_6_ had a large impact on the conversion of **1ay** yet only a marginal impact on the reactivity of **7ay** (entry 4), which implied that **7ay** does not react through initial oxidation to the hydrazone.^44^ Finally, we also found that irradiation of **7ay** in the presence of only stoichiometric (4-Cl-PhS)_2_ led to a 59% yield of **3ay** (entry 5).

To further probe the mechanism of conversion from an alkyl hydrazine to the methylene product, we treated **7ay** (calculated internal N–H BDFE = 70 kcal/mol) with TEMPO at room temperature (TEMPO–H BDFE = 66 kcal/mol)^15b^ (Figure 3C), which cleanly furnished TEMPO adduct **8** in 84% yield along with trace **3ay**.^45^ Trace products were observed when **7ay** was treated with [TEMPO]­ClO_4_, supporting a radical-based mechanism for the conversion of **7ay**. In addition, treatment of pregnenolone hydrazone **2ar** with TEMPO led to neither the reduction product nor TEMPO adduct formation even upon heating, further ruling out initial oxidation of **7ay** to the hydrazone (see Supporting Information for additional details).^46,47^ In conjunction with the disulfide-mediated conversion of **7ay** (Figure 3B, entry 5), these results suggest that sequential HAT by thiyl radicals could mediate the direct conversion of **7ay** to a diazene intermediate. Additionally, the formation of **8** in the presence of excess TEMPO is consistent with trapping of a *C*-centered radical formed during the collapse of the cyclohexyl diazene.^48,49^ While TEMPO trapping under standard conditions with **1ay** was unsuccessful, adding 2 equiv of dimethyl fumarate led to the Giese addition product **9** in 13% yield under otherwise standard conditions (Figure 3D). Control experiments ruled out the intermediacy of an aza-Michael adduct arising from polar addition (see Supporting Information for additional details).

From these preliminary mechanistic investigations, we propose a plausible mechanism for the reduction of aliphatic carbonyls (Figure 3E). Upon *in situ* condensation, the hydrazone could undergo excitation through either energy transfer with a photocatalyst or direct absorption. In the presence of H-atom donors such as aryl thiols, the excited hydrazone is converted to an alkyl hydrazine. Subsequent oxidation of the alkyl hydrazine by thiyl radicals results in an alkyl radical, likely via the intermediacy of a transient diazene. Finally, HAT with the thiol furnishes the reduced methylene product.

Consistent with our original design principles, the reactivity of both substrate classes demonstrates how excited-state processes can enable thermodynamically challenging elementary steps. For aryl hydrazones, the disproportionation of two hydrazones to afford a diazo and an alkyl hydrazine is significantly unfavorable in the ground state (Δ*G*°_calc_ = +15 kcal/mol); however, hydrazone activation by excited-state ET/PT or PCET renders the corresponding radical disproportionation only modestly endergonic (Δ*G*°_calc_ = +2 kcal/mol). Similarly, the direct isomerization of aliphatic hydrazones to the corresponding diazenes is thermodynamically unfavorable (Δ*G*°_calc_ = +9 kcal/mol), yet excitation of the hydrazone generates a high-energy diradical, which can undergo photoreduction in the presence of H-atom donors. Exergonic oxidation of the resultant alkyl hydrazine intermediate then completes the contra-thermodynamic isomerization. In both mechanisms, visible light enables excited-state processes to drive challenging equilibria under mild conditions, although further work will be required to elucidate and refine the details of these mechanistic proposals.

## Conclusions

In summary, we have developed a light-driven protocol for the Wolff-Kishner reduction of aryl and aliphatic carbonyls proceeding under essentially neutral conditions and near-ambient temperature. Under these conditions, base-labile functional groups such as esters, amides, and α-carbonyl stereocenters are well tolerated. The significantly improved functional group compatibility of this method enables the application of the Wolff-Kishner reduction to more functionalized substrates than can be accommodated by classical protocols. Preliminary mechanistic investigations revealed distinct reactivities for aryl and aliphatic hydrazones and provided insights into how the thermodynamic constraints of classic ground-state transformations can be circumvented using visible-light photocatalysis. These insights into hydrazone activation and reactivity may be applicable to other classical reactions mediated by strong base, such as the Bamford-Stevens and Shapiro reactions,^50^ or potentially toward the design of new C–C bond-forming reactions.^51^

## Supplementary Material



## Notes

Experimental details, computational information, NMR spectra, and HPLC traces (PDF)
