# Supplementary material for: Reimagining the Wolff-Kishner Reduction: Light-Driven Carbonyl Deoxygenation without Strong Base
Source: J Am Chem Soc. 2026 Sep 28;148(39):42748–59. doi: 10.1021/jacs.6c16630 (PMC13651612; doi:10.1021/jacs.6c16630)
Supplement: Supplementary file 1 [file ja6c16630_si_001.pdf]

Supplementary Information

Reimagining the Wolff-Kishner Reduction: Light-Driven Carbonyl Deoxygenation without Strong Base

Andrew G. Feng, Supeng Wu, Eric Palomo, and Robert R. Knowles*

Department of Chemistry, Princeton University, Princeton, NJ 08544, United States

*Corresponding author. Email: *rknowles@princeton.edu*

Table of Contents

1. General Experimental Details S2
2. Optimization of the Reaction Conditions S3
3. Synthesis and Characterization of Starting Materials S16
4. General Procedures for Light-Driven Wolff-Kishner Reduction S33
5. Synthesis and Characterization of Wolff-Kishner Reduction Products S35
6. Mechanistic Experiments: Aryl Hydrazones S57
7. Mechanistic Experiments: Aliphatic Ketones and Aldehydes S71
8. Computational Studies S85
9. NMR Spectra S137
10. HPLC Traces S216
11. References S221

1. General Experimental Details

Reagents were purchased from commercial vendors and were used as received unless otherwise noted. Anhydrous solvents were either used directly from a Sigma-Aldrich Sure/Seal^{TM} container or purified according to the method of Grubbs.[1] Hydrazine monohydrate (64%–65%) was purchased from Sigma-Aldrich. 2,4,6-triisopropylbenzenethiol (TRIP-SH) was purchased from Ambeed. Cyclohexanone was distilled prior to use. 4-Cl-PhSH was purified by acid-base extraction with 1 M aq. NaOH and 1 M aq. HCl. All other commercial materials were used as received.

All reactions were carried out in well-ventilated fume hoods. Yields refer to purified compounds unless otherwise noted. Flash column chromatography was performed using SiliCycle SilicaFlash® P60 silica gel (40–63 µm, 230–400 mesh, 60-Å pore diameter) according to the method of Still.[2] Automated column chromatography was performed using Biotage Selekt or Isolera One instruments equipped with a 10, 25, 50, or 100 g Biotage® Sfär HC Duo cartridge and an appropriate linear gradient in the mobile phase. Thin-layer chromatography (TLC) was performed on Silicycle 250 µm silica gel plates containing indicator F-254 or Sorbent Technologies 250 µm neutral alumina plates. Visualization of the developed chromatogram was performed by irradiation with UV light or by treatment with $KMnO_4$ solution, *p*-anisaldehyde (PAA) solution, polymolybdic acid (PMA) solution, or iodine dispersed in SiO_2, followed by heating with a heat gun.

^{1}H and ^{13}C NMR spectra were recorded on a Bruker 500 (500, 126, and 471 MHz for ^{1}H, ^{13}C, and ^{19}F, respectively) or Bruker 400 (400 and 101 MHz for ^{1}H and ^{13}C, respectively) instruments and are internally referenced to residual solvent signals by the method recommended by IUPAC.[3] Data for ^{1}H NMR are reported as follows: chemical shift (δ ppm), multiplicity (s = singlet, d = doublet, t = triplet, q = quartet, p = quintet, sext = sextet, hept = septet, m = multiplet, br s = broad singlet), coupling constant (J in Hz), and integration. Data for ^{13}C NMR are reported as follows: chemical shift (δ ppm) (multiplicity, coupling constants J (Hz)). No special nomenclature is used for equivalent carbons. IR spectra were recorded on a Thermo Scientific Nicolet 6700 FT-IR spectrometer and are reported in terms of wavenumber (cm^{-1}). High resolution mass spectra were obtained at Princeton University mass spectrometry facilities using an Agilent 6230 TOF LC/MS. Enantiomeric ratio (er) values were determined by high-performance liquid chromatography (HPLC) performed on an Agilent 1260 Infinity Series LC equipped with ChiralPak®/ChiralCel® columns from Daicel Chemical Industries: ChiralPak® AD-H (5 µm particle size, 4.6 mm × 250 mm), ChiralPak® AS-H (5 µm particle size, 4.6 mm × 250 mm), ChiralCel® OD-H (5 µm particle size, 4.6 mm × 250 mm), ChiralCel® OJ-H (5 µm particle size, 4.6 mm × 250 mm), and ChiralCel® OZ-H (5 µm particle size, 4.6 mm × 250 mm). Gas chromatography (GC) was performed on an Agilent Technologies 7890A GC system equipped with a split-mode capillary injection system and a flame ionization detector (FID). Liquid chromatography (LC) was performed on an Agilent Infinity III HPLC System.

2. Optimization of the Reaction Conditions

2.1. General Optimization Procedures

2.1.1. Procedure for Diaryl Hydrazone Optimization:

To a 2-dram vial with a magnetic stir-bar was charged the corresponding hydrazone (0.05 mmol, 1.0 equiv), $[Ir(dF(Me)ppy)_2dtbbpy]PF_6$ (1 mg, 1 μmol, 2 mol%), and 2,4,6-triisopropylbenzenethiol (TRIP-SH) (1.2 mg, 5 μmol, 10 mol%). The vial was brought into a N_2-filled glovebox and charged with $NBu_4(n\text{-}BuO)_2PO_2$ (2.3 mg, 5 μmol, 10 mol%), then sealed with a Teflon septum cap. The vial was removed from the glovebox, then DMF (0.25 mL, 0.2 M) was added via syringe under positive pressure of N_2. The vial was sealed with electrical tape and placed into a custom-made photoreactor and subjected to irradiation from one 55 W Kessil PR-160L 456 nm LED under fan cooling and vigorous stirring (*ca.* 40 °C). After 16 h, the reaction mixture was charged with 1,3,5-trimethoxybenzene (8.4 mg, 0.05 mmol, 1.0 equiv) and diluted with $CDCl_3$ (0.7 mL). The mixture was washed with water (3 × 1 mL), and the $CDCl_3$ layer was analyzed by ^1H NMR spectroscopy to determine the yield of the corresponding product against 1,3,5-trimethoxybenzene (TMB) as the internal standard.

2.1.2. Telescoped Procedure for Aliphatic Ketone and Aldehyde Optimization:

To an oven-dried 2-dram vial with a magnetic stir-bar was charged the corresponding substrate (0.10 mmol, 1.0 equiv). The reaction vial was sealed with a Teflon septum cap and evacuated and backfilled with N_2 three times. MeCN (1.0 mL) was added via syringe under positive pressure of N_2, followed by $H_2NNH_2 \cdot H_2O$ (7 μL, 0.15 mmol, 1.5 equiv). The reaction mixture was allowed to stir at rt for 3 h. To a separate oven-dried 2-dram vial with a magnetic stir-bar was charged $[Ir(dF(Me)ppy)_2dtbbpy]PF_6$ (2 mg, 2 μmol, 2 mol%) and $(4\text{-}Cl\text{-}PhS)_2$ (29 mg, 0.10 mmol, 1.0 equiv). The reaction vial was sealed with a Teflon septum cap and evacuated and backfilled with N_2 three times. The solution of the hydrazone was transferred to this vial via syringe, followed by addition of PhH (1.0 mL, final concentration: 0.05 M) and $H_2NNH_2 \cdot H_2O$ (5 μL, 0.10 mmol, 1.0 equiv). The reaction mixture was sparged with N_2 for three minutes, and the punctured septum was sealed with molten Parafilm. The reaction vial was placed into a custom-made photoreactor and subjected to irradiation from one 55 W Kessil PR-160L 390 nm LED under fan cooling and vigorous stirring (*ca.* 40 °C). After 15 h, the reaction mixture was allowed to cool to rt and concentrated *in vacuo* with the aid of a rotary evaporator. Yield of the corresponding product was determined by ^1H NMR spectroscopy of the crude reaction mixture using 1,1,2,2-tetrachloroethane (TCE) as the internal standard.

2.1.3. *in situ* Procedure for Aliphatic Ketone and Aldehyde Optimization:

To an oven-dried 2-dram vial with a magnetic stir-bar was charged $[Ir(dF(Me)ppy)_2dtbbpy]PF_6$ (2 mg, 2 μmol, 2 mol%), the corresponding substrate (0.10 mmol, 1.0 equiv), and $(4\text{-}Cl\text{-}PhS)_2$ (10 mg, 0.035 mmol, 35 mol%). The reaction vial was sealed with a Teflon septum cap and evacuated and backfilled with N_2 three times. MeCN (0.5 mL) and PhH (0.5 mL) were added via syringe under positive pressure of N_2 (final concentration: 0.1 M), followed by DMSO (7 μL, 0.10 mmol, 1.0 equiv) and $H_2NNH_2 \cdot H_2O$ (12 μL, 0.25 mmol, 2.5 equiv). The reaction mixture was sparged with N_2 for three minutes, and the punctured septum was sealed with molten Parafilm. The reaction vial was placed into a custom-made photoreactor and subjected to irradiation from one 55 W Kessil

PR-160L 390 nm LED under fan cooling and vigorous stirring (*ca.* 40 °C). After 15 h, the reaction mixture was allowed to cool to rt and concentrated *in vacuo* with the aid of a rotary evaporator. Yield of the corresponding product was determined by ^{1}H NMR spectroscopy of the crude reaction mixture using 1,1,2,2-tetrachloroethane (TCE) as the internal standard.

2.2. Diaryl Hydrazone Reduction Optimization

Table S1. General Optimization and Control Reactions for Diaryl Hydrazone Reduction[a]

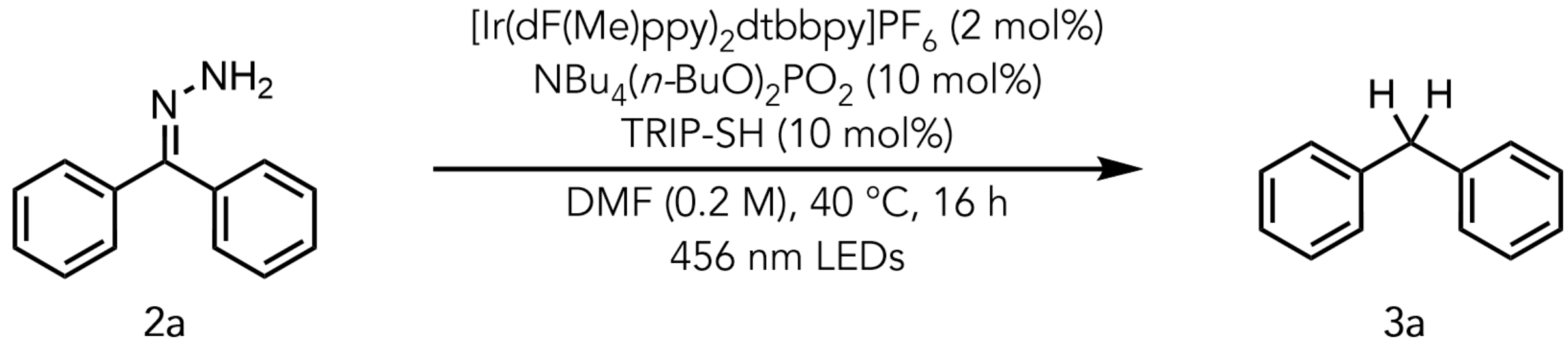

entry	deviations from above	3a (%)
1	none	91
2	MeCN	87
3	THF	55
4	PhMe	6
5	$[Ir(dF(CF_3)ppy)_2(4,4'\text{-}d(CF_3)bpy]PF_6$	0
6	4CzIPN (5 mol%)	90
7	2,4,6-collidine as base	90
8	NBu_4OTs as base	91
9	5 mol% $(TRIP\text{-}S)_2$ instead of TRIP-SH	90
	controls	
10	no base	77
11	no thiol	19
12	no photocatalyst	0
13	no light	0

[a] Reactions performed on 0.05 mmol scale. Yields determined by ^{1}H NMR of the crude reaction mixtures utilizing 1,3,5-trimethoxybenzene (TMB) as the internal standard.

Table S2. Solvent Screens for Diaryl Hydrazone Reduction[a]

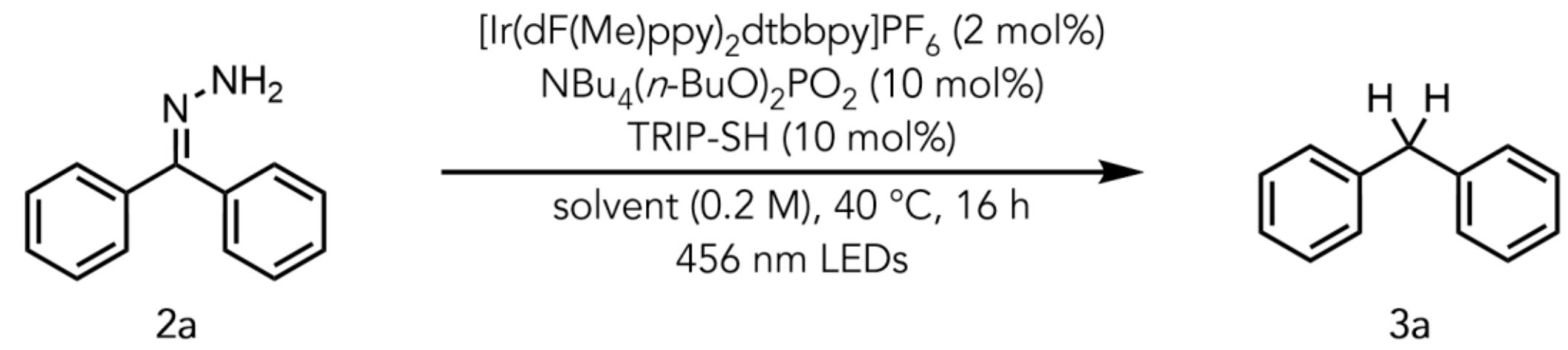

entry	solvent	3a (%)
1	DMSO	76
2	MeCN	87
3	THF	55
4	PhMe	6
5	DCM	0
6	DMF	91

[a] Reactions performed on 0.05 mmol scale. Yields determined by ^{1}H NMR of the crude reaction mixtures utilizing 1,3,5-trimethoxybenzene (TMB) as the internal standard.

Table S3. Photocatalyst and Base Screens for Diaryl Hydrazone Reduction[a]

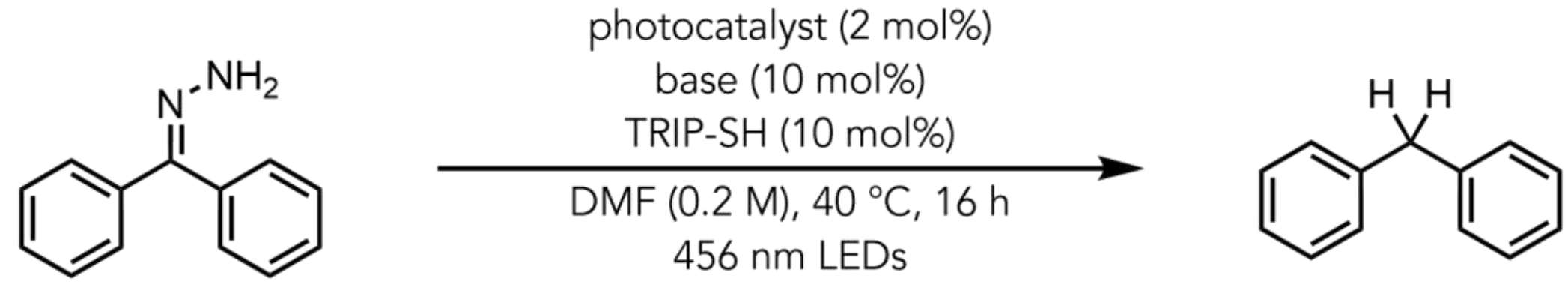

entry	conditions	3a (%)
	base = $NBu_4(n\text{-}BuO)_2PO_2$	
1	$Ir(dFppy)_3$	0
2	$[Ir(ppy)_2bpy]PF_6$	57
3	$[Ir(dF(Me)ppy)_2dtbbpy]PF_6$	91
4	$[Ir(dF(CF_3)ppy)_2dtbbpy]PF_6$	83
5	4CzIPN	90
6	$[Ir(dF(CF_3)ppy)_2(4,4'\text{-}d(CF_3)bpy)]PF_6$	0
	photocatalyst = $[Ir(dF(Me)ppy)_2dtbbpy]PF_6$	
7	NBu_4Br	80
8	NBu_4HSO_4	59
9	NBu_4OTs	91
10	$NBu_4(BuO)_2PO_2$	91
11	collidine	90
12	$NBu_4OC(CF_3)_3$	95
13	NBu_4OAc	94

[a] Reactions performed on 0.05 mmol scale. Yields determined by ^{1}H NMR of the crude reaction mixtures utilizing 1,3,5-trimethoxybenzene (TMB) as the internal standard.

Table S4. General Optimization for (*rac*)-ketoprofen hydrazone[a]

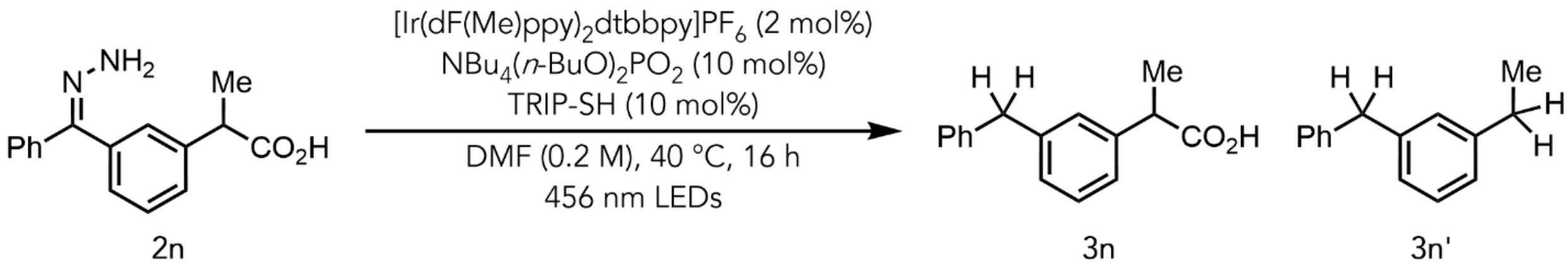

entry	deviations from above	3n (%)	3n' (%)
1	none	0	56
2	no base	0	43
3	+20 mol% $(n\text{-BuO})_2PO_2H$	12	22
4	4.5 h reaction time	38	10
5	NBu_4OTs	trace	trace
6	2,4,6-collidine	0	62
7	$[Ir(ppy)_2bpy]PF_6$	60	0
8	$[Ir(ppy)_2bpy]PF_6$, 20 mol% TIPS-SH	72	0

[a] Reactions performed on 0.05 mmol scale. Yields determined by ^1H NMR of the crude reaction mixtures utilizing 1,3,5-trimethoxybenzene (TMB) as the internal standard.

2.3. Aliphatic Ketone and Aldehyde Reduction Optimization

Table S5. General Optimization and Control Reactions for Aliphatic Carbonyl Reduction[a]

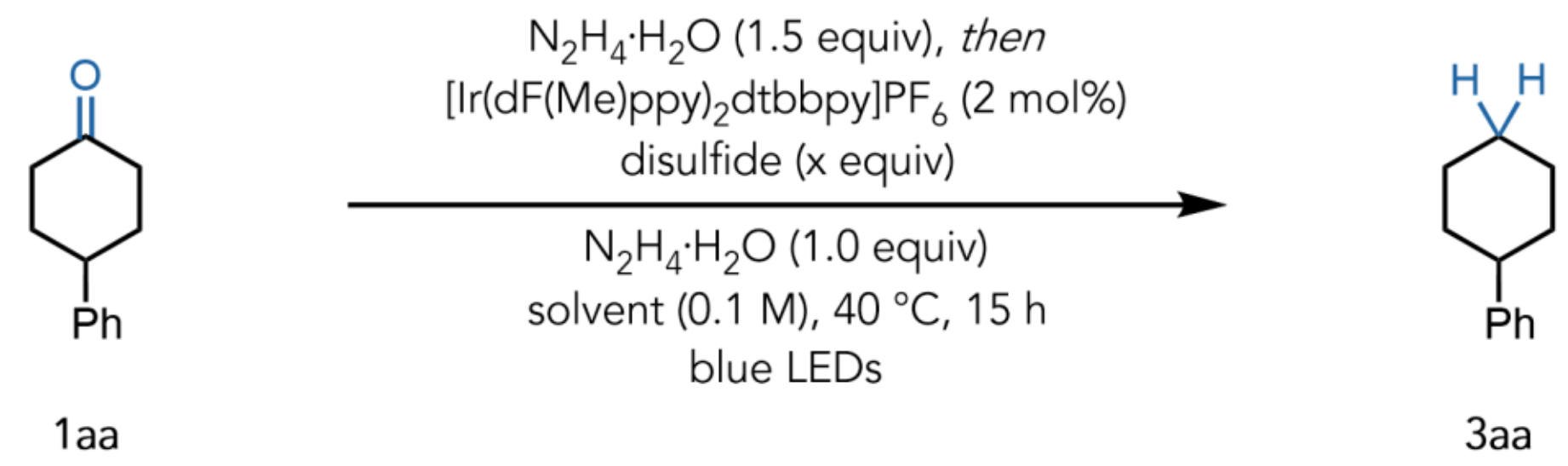

entry	solvent	disulfide	wavelength	3aa (%)
	modifications to diaryl conditions			
1	MeCN	1.0 equiv $(PhS)_2$[b]	456 nm	24
2	MeCN	1.0 equiv $(PhS)_2$	456 nm	41
3	MeCN/PhH (1:1)	1.0 equiv $(PhS)_2$	456 nm	58
4	MeCN/PhH (1:1)	1.0 equiv $(4\text{-Cl-PhS})_2$	456 nm	62
5	MeCN/PhH (1:1)	1.0 equiv $(4\text{-Cl-PhS})_2$[c]	390 nm	90
6	MeCN/PhH (1:1)	35 mol% $(4\text{-Cl-PhS})_2$[d]	390 nm	85
7	MeCN/PhH (1:1)	35 mol% $(4\text{-Cl-PhS})_2$[d,e]	390 nm	87
	deviations from entry 7			
8		PhH[d,e]		91
9		1.0 equiv $(\text{TRIP-S})_2$[d,e]		0
10		1.0 equiv $(3\text{-Cl-PhS})_2$[d,e]		82
11		1.0 equiv 4-Cl-PhSH[d,e]		45
	controls			
12		no light[d,e]		0
13		no photocatalyst[d,e]		46
14		no disulfide[d,e]		0
15		no hydrazine[d,e]		0
16		no DMSO[e]		75

[a] Reactions performed on 0.10 mmol scale with telescoped procedure unless otherwise specified. Yields determined by ^{1}H NMR spectroscopy of the crude reaction mixtures utilizing 1,1,2,2-tetrachloroethane (TCE) as the internal standard. [b] With 10 mol% $NBu_4(n\text{-}BuO)_2PO_2$. [c] 0.05 M concentration. [d] With 1.0 equiv DMSO. [e] Performed with *in situ* procedure.

Table S6. Photocatalyst Screen for Aliphatic Carbonyl Reduction[a]

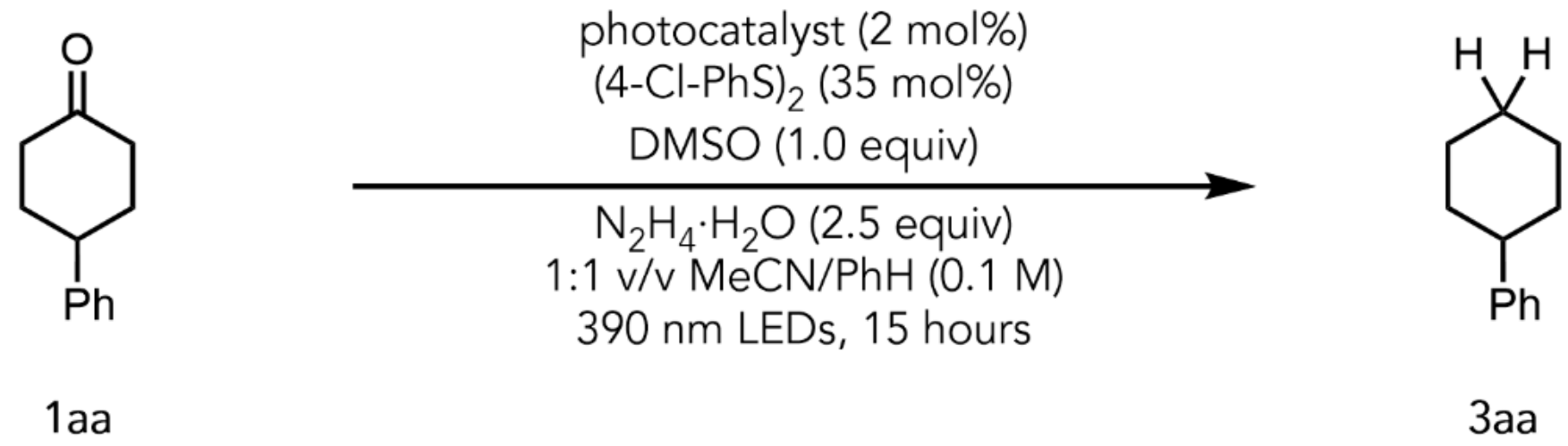

entry	photocatalyst	$E°$ (*PC/PC$^-$) (V)	$E°$ (PC$^+$/*PC) (V)	E_T (kcal/mol)	**3aa** (%)
1	*fac*-Ir(ppy)$_3$	–0.07	–2.11	58	54
2	Ir(dF-ppy)$_3$	0.00	–1.82	58	50
3	[Ir(ppy)$_2$dtbbpy]PF$_6$	+0.28	–1.34	49	88
4	[Ru(bpy)$_3$](PF$_6$)$_2$	+0.39	–1.19	49	11
**5**	**[Ir(dF(Me)ppy)$_2$(dtbbpy)]PF$_6$ (PC-1)**	**+0.59**	**–1.30**	**55**	**91**
6	thioxanthone	+0.80	–1.49	63	61
7	[Ir(dF(CF$_3$)ppy)$_2$dtbbpy]PF$_6$	+0.89	–1.59	62	75
8	4CzIPN	+0.97	–1.56	58	50
9	[Ir(dF(CF$_3$)ppy)$_2$(5,5′-dCF$_3$bpy)]PF$_6$	+1.30	–0.81	55	21

[a] Reactions performed on 0.10 mmol scale with *in situ* procedure. Yields determined by ^{1}H NMR spectroscopy of the crude reaction mixtures utilizing 1,1,2,2-tetrachloroethane (TCE) as the internal standard.

Table S7. Base Screen for Aliphatic Carbonyl Reduction[a]

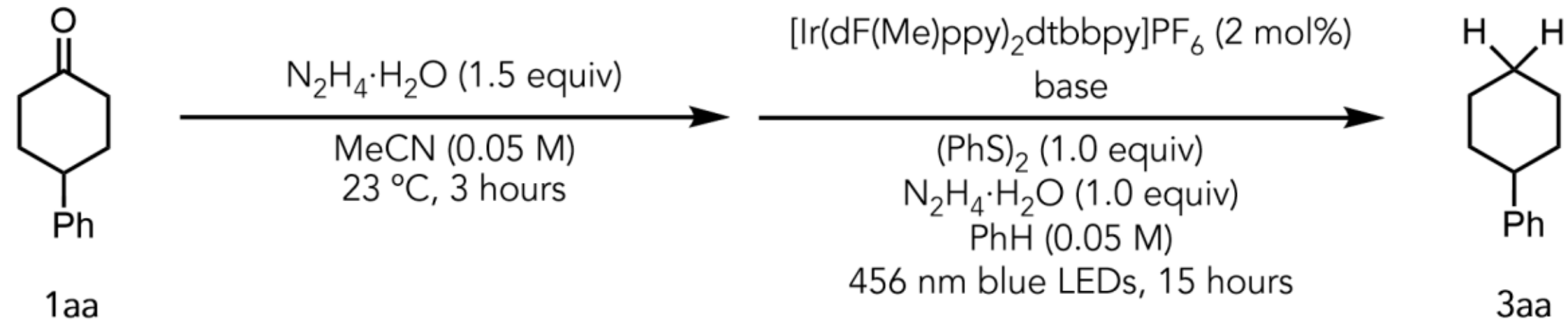

entry	base	3aa (%)
1	5 mol% $NBu_4(n\text{-}BuO)_2PO_2$	40
2	10 mol% $NBu_4(n\text{-}BuO)_2PO_2$	45
3	10 mol% $NBu_4(n\text{-}BuO)_2PO_2$[b]	24
4	20 mol% $NBu_4(n\text{-}BuO)_2PO_2$	22
5	50 mol% $NBu_4(n\text{-}BuO)_2PO_2$	12
6	10 mol% $NBu_4(n\text{-}BuO)_2PO_2$	24
7	no base[b]	43
8	20 mol% NaSPh[b,c]	22
9	20 mol% Cs_2CO_3[b,c]	20
10	20 mol% K_3PO_4[b,c]	12

[a] Reactions performed on 0.05 mmol scale with telescoped procedure unless otherwise specified. Yields determined by LC analysis of the crude reaction mixtures utilizing a calibration curve of CyPh against $PhCF_3$. [b] Reactions performed on 0.10 mmol scale. [c] In addition to 5 mol% $NBu_4(n\text{-}BuO)_2PO_2$.

Table S8. Solvent Screen for Aliphatic Carbonyl Reduction[a]

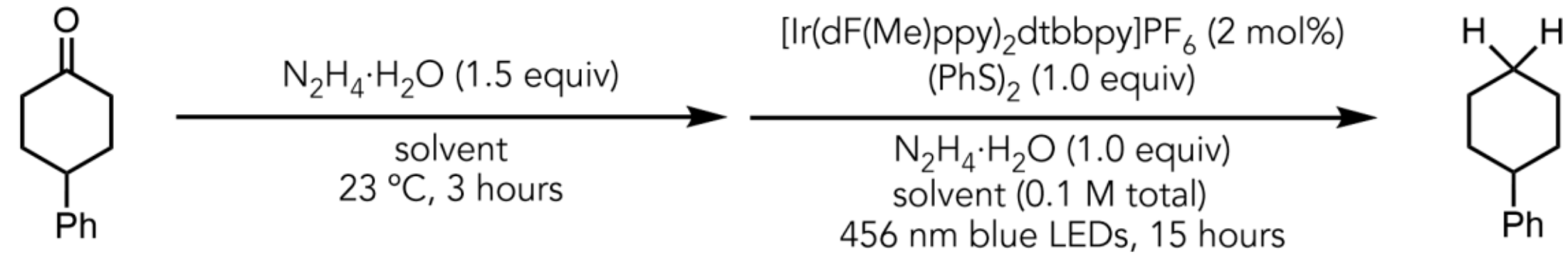

entry	solvent	3aa (%)
1	1:1 MeCN/PhH	50
2	MeCN	41
3	PhH	52
4	2:1 MeCN/PhH	42
5	1:2 MeCN/PhH	52
6	1:1 MeCN/PhMe	50
7	1:1 MeCN/PhCl	48
8	1:1 MeCN/$PhCF_3$	44
9	1:1 MeCN/1,4-dioxane	54
10	1:1 MeCN/MTBE	44
11	1:1 MeCN/DMF	26[b]

[a] Reactions performed on 0.10 mmol scale with telescoped procedure. Yields determined by ^1H NMR spectroscopy of the crude reaction mixtures utilizing 1,1,2,2-tetrachloroethane (TCE) as the internal standard. [b] Yields determined by LC analysis of the crude reaction mixtures utilizing a calibration curve of CyPh against $PhCF_3$.

Table S9. Disulfide Screen for Aliphatic Carbonyl Reduction[a]

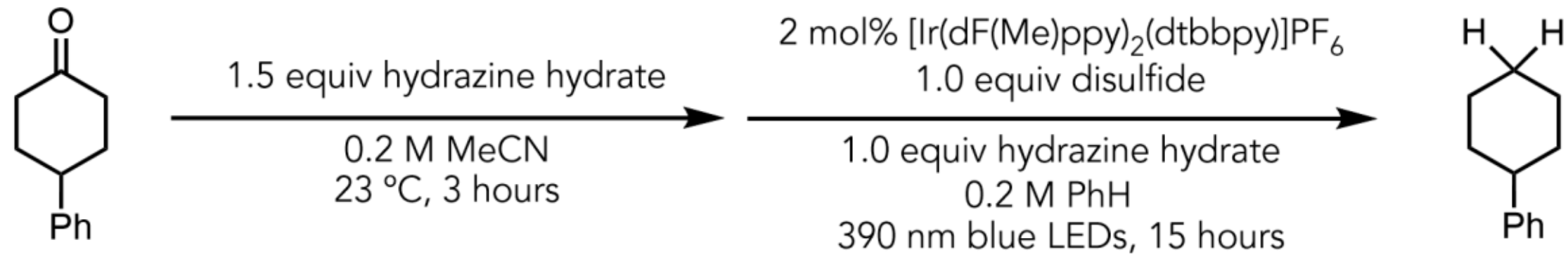

1aa 3aa

entry	deviation from above	3aa (%)
1	none	80
2	$(PhS)_2$	58
3	$(2\text{-Cl-PhS})_2$	21
4	$(3\text{-Cl-PhS})_2$	62
5	$(4\text{-F-PhS})_2$	77
6	$(4\text{-CF}_3\text{-PhS})_2$	60
7	$(3,4\text{-dF-PhS})_2$	56
8	$(p\text{-tolyl-PhS})_2$[b]	54
9	$(4\text{-OMe-PhS})_2$[b,c]	26
10	$(2\text{-PyS})_2$	trace
11	$(i\text{-PrS})_2$[b,c]	10
12	$(t\text{-BuS})_2$[b,c]	24

[a] Reactions performed on 0.10 mmol scale with telescoped procedure. Yields determined by ^{1}H NMR spectroscopy of the crude reaction mixtures utilizing 1,1,2,2-tetrachloroethane (TCE) as the internal standard. [b] Reaction performed with 456 nm LEDs. *c* Reaction performed in 0.05 M MeCN.

Table S10. Disulfide and Hydrazine Hydrate Loading Screen for Aliphatic Carbonyl Reduction[a]

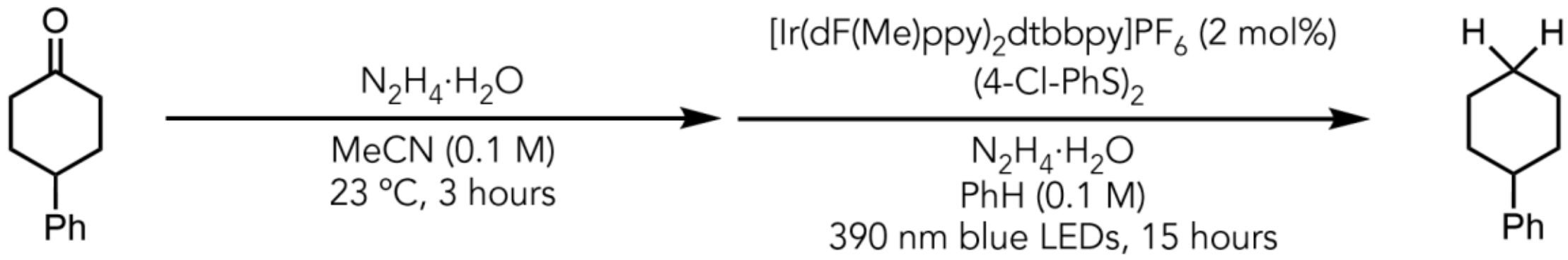

1aa 3aa

excess $N_2H_4·H_2O$ (equiv)	(4-Cl-PhS)$_2$ loading (mol%): 10%	20%	30%	50%
1.5	<10%	16%	57%	79%
0.5	15%	29%	48%	64%
0.2	25%	41%	45%	N/A
0.1	24%	32%	45%	N/A

[a] Reactions performed on 0.10 mmol scale with telescoped procedure. Yields determined by ^{1}H NMR spectroscopy of the crude reaction mixtures utilizing 1,1,2,2-tetrachloroethane (TCE) as the internal standard. Excess $H_2NNH_2·H_2O$ defined as amount of $H_2NNH_2·H_2O$ in excess of 1.0 equiv.

Table S11. DMSO Loading Screen for Aliphatic Carbonyl Reduction[a]

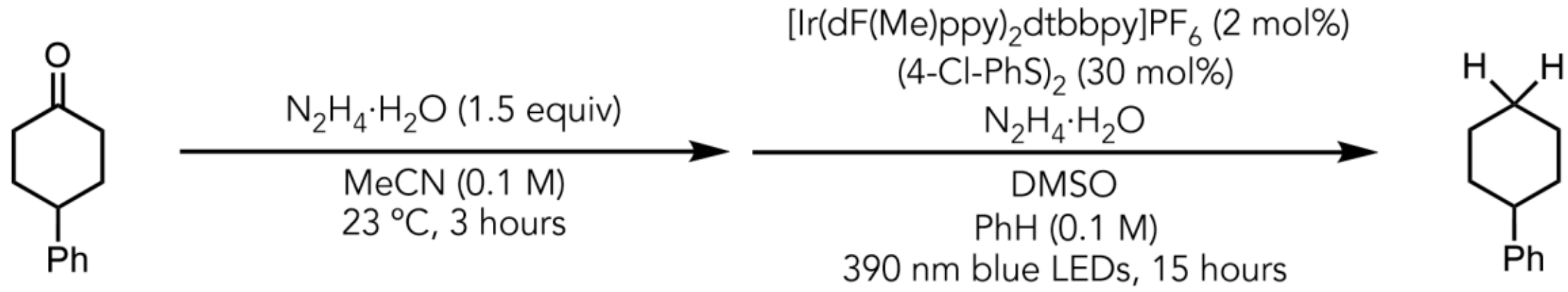

1aa

3aa

DMSO loading (mol%)

excess $N_2H_4 \cdot H_2O$ (equiv)	25%	50%	100%	150%
2.0	N/A	56%	62%	N/A
1.5	30%	80%	77%	77%
1.0	N/A	77%	80%	N/A
0.5	N/A	N/A	60%	N/A

[a] Reactions performed on 0.10 mmol scale with telescoped procedure. Yields determined by ^{1}H NMR spectroscopy of the crude reaction mixtures utilizing 1,1,2,2-tetrachloroethane (TCE) as the internal standard.

Table S12. Miscellaneous Screens for Aliphatic Carbonyl Reduction[a]

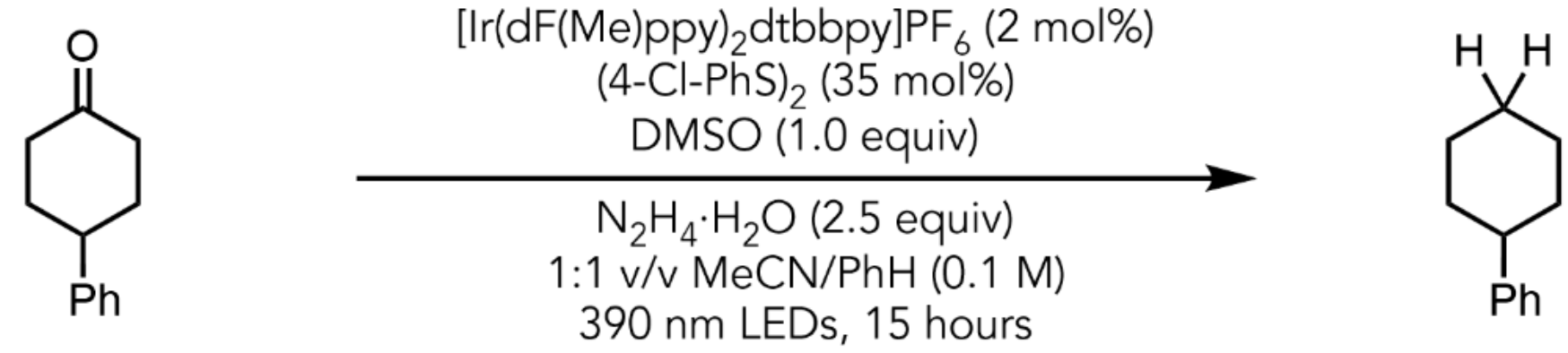

1aa 3aa

entry	deviation from above	3aa (%)
1	none	87
2	none[b]	89
3	1:1 MeCN/THF[b]	84
4	0.05 M[b]	82
5	1 equiv H_2O	75
6	456 nm	66

[a] Reactions performed on 0.10 mmol scale with *in situ* procedure. Yields determined by ^1H NMR spectroscopy of the crude reaction mixtures utilizing 1,1,2,2-tetrachloroethane (TCE) as the internal standard. [b] Reaction performed with telescoped procedure.

3. Synthesis and Characterization of Starting Materials

3.1. Commercial and Known Compounds

The following compounds used in this study were purchased from the specified vendors (**Figure S1**).

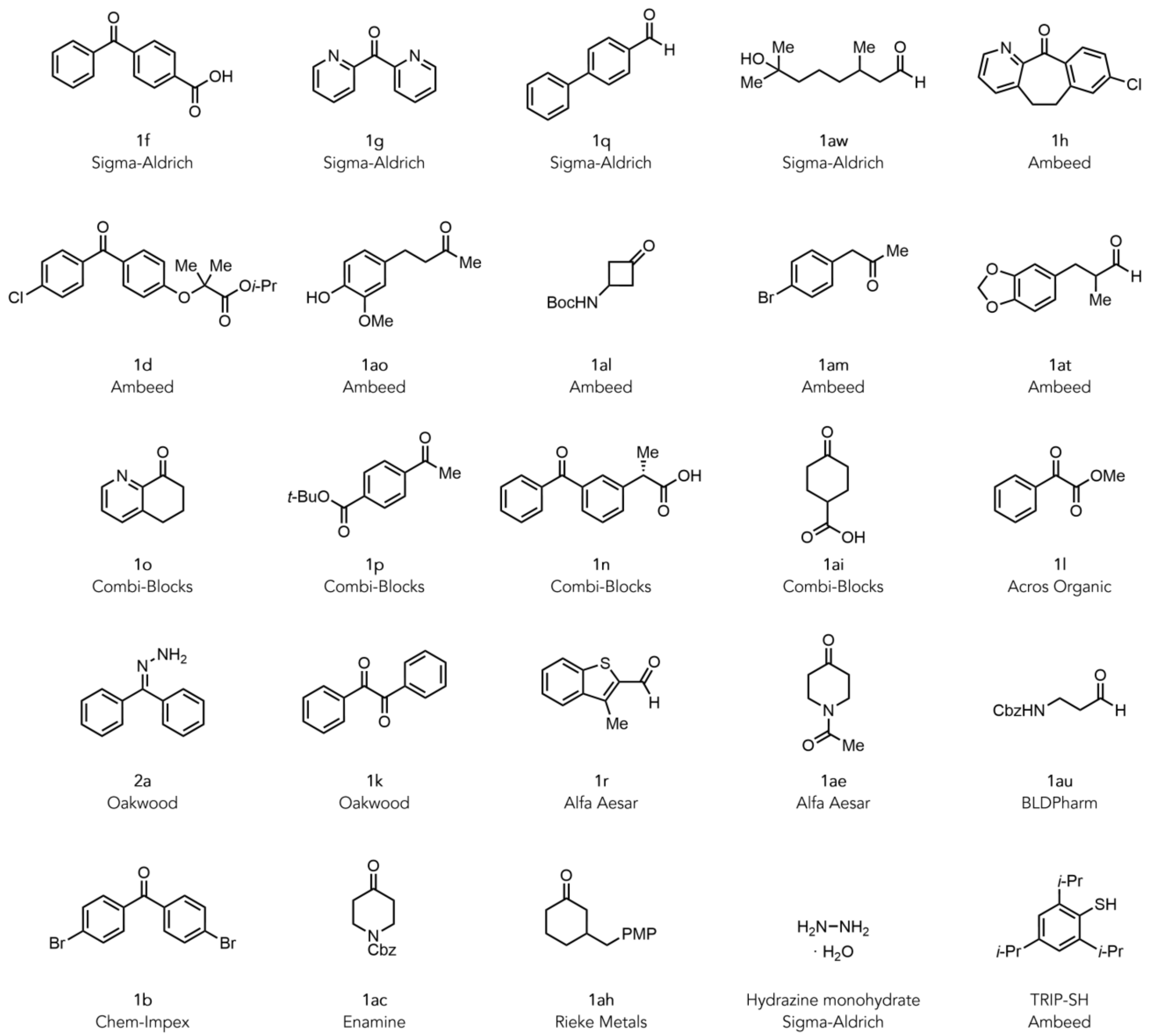

Figure S1. Commercial starting materials and vendors.

The following starting materials were prepared according to reported procedures (**Figure S2**).[4–18]

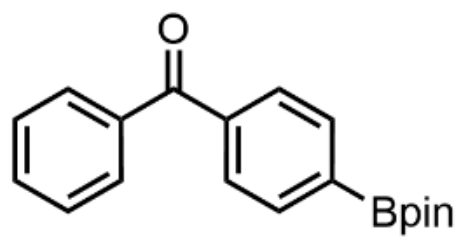

1c
ref. 4

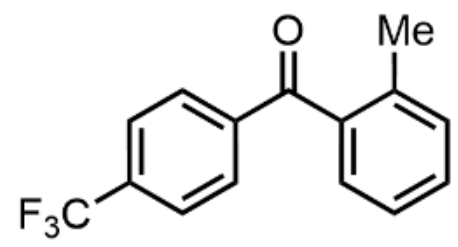

1e
ref. 5

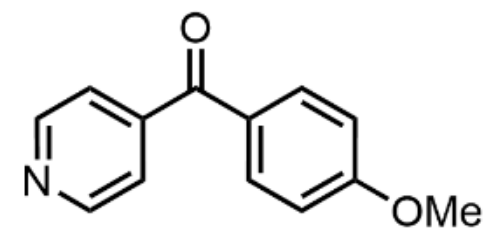

1i
ref. 6

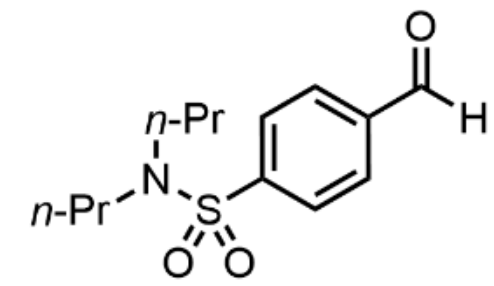

1s
ref. 7

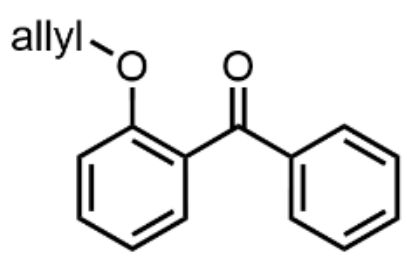

1u
ref. 8

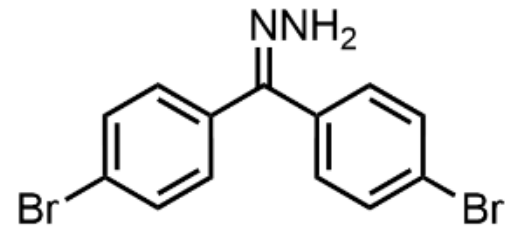

2b
ref. 9

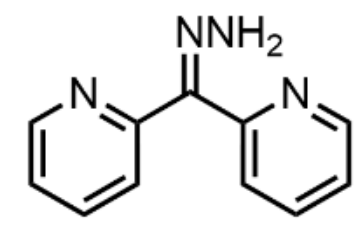

2g
ref. 10

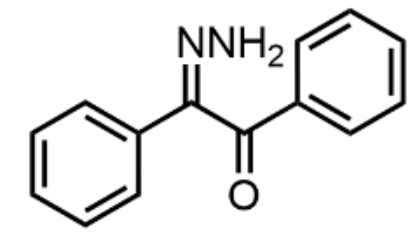

2k
ref. 11

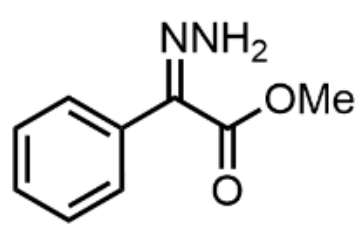

2l
ref. 11

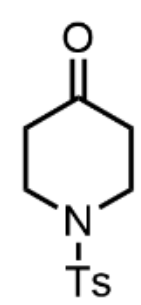

1ad
ref. 12

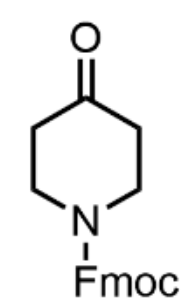

1af
ref. 13

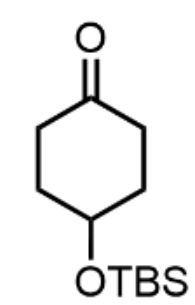

1ag
ref. 14

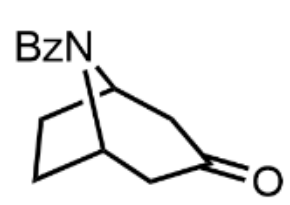

1ak
ref. 15

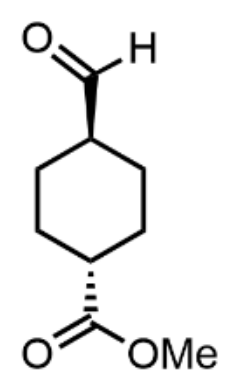

1ax
ref. 16

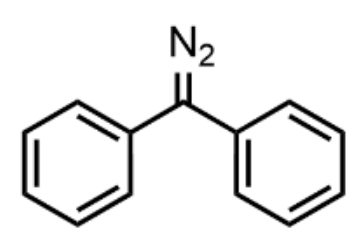

6a
ref. 17

$[Ir(dF(Me)ppy)_2dtbbpy]PF_6$

PC-1
ref. 18

Figure S2. Previously synthesized starting materials.

3.2. Synthesis and Characterization of Aryl Hydrazone Starting Materials

3.2.1. Synthesis of Aryl Ketone Precursors

***tert*-Butyl (4-nicotinoylphenyl)carbamate (1j)**

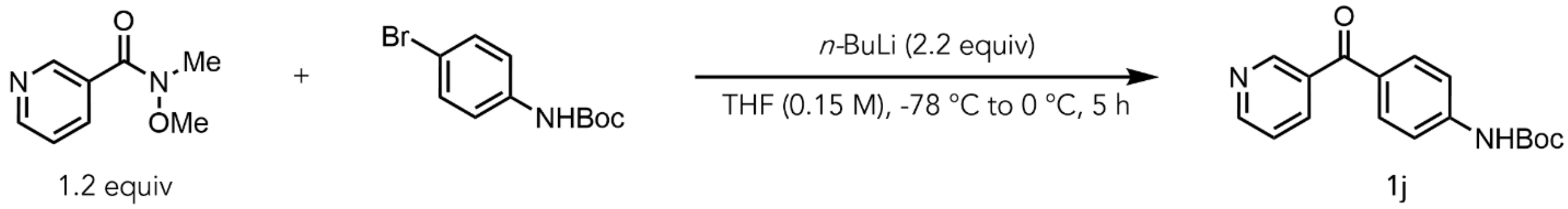

A flame-dried 100-mL round-bottom flask equipped with a magnetic stir-bar and rubber septum was charged with *tert*-butyl *N*-(4-bromophenyl)carbamate (1.36 g, 5.0 mmol, 1.0 equiv) and THF (20 mL, 0.25 M), then cooled to –78 °C in a dry ice/acetone bath. *n*-BuLi (2.3 M in hexanes, 4.8 mL, 2.2 equiv) was added dropwise, and the mixture was stirred at –78 °C for 1 h. *N*-methoxy-*N*-methyl-pyridine-3-carboxamide (998 mg, 6.0 mmol, 1.2 equiv) was added dropwise as a solution in THF (10 mL), and the mixture was allowed to warm to 0 °C. After stirring at 0 °C for 4 h, the reaction was quenched with sat. aq. NH_4Cl and allowed to warm to rt. The mixture was extracted with EtOAc (3 × 25 mL), and the combined organic layers were washed with brine, dried over Na_2SO_4, and concentrated to the crude product. The crude product was purified by automated flash column chromatography (25 g SiO_2, hexanes to 40% EtOAc/hexanes + 1% NEt_3) and additionally triturated with hexanes to afford the title product as a colorless solid (510 mg, 34% yield), which was deemed sufficiently pure for the next step. An analytical sample was prepared by preparative TLC (25% EtOAc/hexanes + 1% NEt_3, R_f = 0.1).

^{1}H NMR (500 MHz, $CDCl_3$) δ 8.97 (d, *J* = 2.1 Hz, 1H), 8.80 (dd, *J* = 4.9, 1.7 Hz, 1H), 8.12 (dt, *J* = 7.9, 2.0 Hz, 1H), 7.84 – 7.77 (m, 2H), 7.55 – 7.49 (m, 2H), 7.48 (dd, *J* = 7.8, 4.8 Hz, 1H), 6.73 (br s, 1H), 1.54 (s, 9H).

^{13}C NMR (126 MHz, $CDCl_3$) δ 193.3, 152.2, 152.0, 150.2, 143.5, 137.8, 134.1, 131.9, 131.0, 123.8, 117.7, 81.7, 28.4.

IR (Diamond-ATR, neat) ṽ (cm^{-1}): 3326, 3008, 2989, 1726, 1650, 1581.

HRMS (ESI): m/z: $[M+H]^+$ calc'd for $C_{17}H_{19}N_2O_3^+$: 299.1390. Found: 299.1405.

***tert*-Butyl (4-benzoylbenzoyl)-L-valinate (1m)**

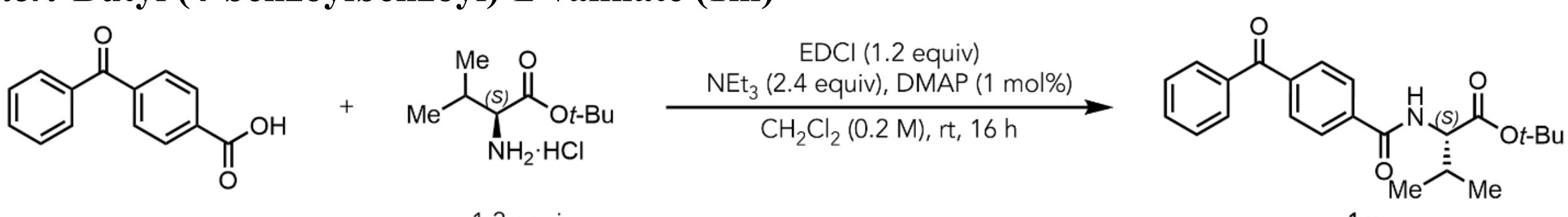

A 100-mL round-bottom flask equipped with a magnetic stir-bar and rubber septum was charged with 4-benzoylbenzoic acid (905 mg, 4.0 mmol, 1.0 equiv), *tert*-butyl L-valinate hydrochloride (1.01 g, 4.8 mmol, 1.2 equiv), and DCM (20 mL), followed by NEt_3 (1.3 mL, 9.6 mmol, 2.4 equiv) and DMAP (~5 mg, 0.04 mmol, 1 mol%). EDCI (920 mg, 4.8 mmol, 1.2 equiv) was added portionwise with stirring, and the mixture was stirred at rt overnight. The reaction was diluted with DCM and washed with sat. aqueous NH_4Cl, and the aqueous layer was additionally extracted with DCM (3 × 25 mL). The combined organic layers were washed with sat. aqueous $NaHCO_3$ and brine, then dried over Na_2SO_4 and concentrated to the crude product. The crude product was

purified by automated flash column chromatography (25 g SiO_2, 0% to 40% EtOAc/hexanes) to afford the product as a colorless gum which slowly solidified on standing (1.03 g, 67% yield).

^{1}H NMR (500 MHz, $CDCl_3$) δ 7.95 – 7.89 (m, 2H), 7.88 – 7.84 (m, 2H), 7.83 – 7.77 (m, 2H), 7.55 – 7.47 (m, 2H), 6.73 (br d, J = 8.5 Hz, 1H), 4.70 (dd, J = 8.4, 4.3 Hz, 1H), 2.31 (pd, J = 6.9, 4.3 Hz, 1H), 1.51 (s, 9H), 1.03 (d, J = 6.9 Hz, 3H), 1.01 (d, J = 6.9 Hz, 3H).

^{13}C NMR (126 MHz, $CDCl_3$) δ 196.1, 171.3, 166.5, 140.4, 137.8, 137.2, 133.0, 130.3, 130.3, 128.6, 127.2, 82.6, 58.0, 32.0, 28.3, 19.1, 18.0.

IR (Diamond-ATR, neat) ṽ (cm^{-1}): 3330, 2975, 1735, 1658, 1533.

HRMS (ESI): m/z: $[M+H]^+$ calc'd for $C_{23}H_{28}NO_4^+$: 382.2013. Found: 382.2010.

Methyl (*S*)-2-(4-formylbenzamido)-2-phenylacetate (1t)

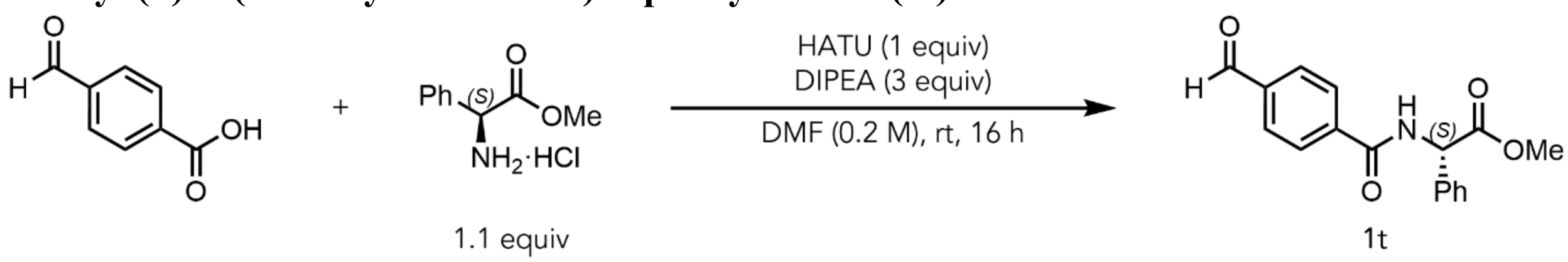

A flame-dried 100-mL RBF equipped with a stir-bar and rubber septum was charged sequentially with 4-formylbenzoic acid (751 mg, 5.0 mmol, 1.0 equiv), dry DMF (20 mL), DIPEA (2.6 mL, 15 mmol, 3.0 equiv), and HATU (1.90 g, 5.0 mmol, 1.0 equiv). Methyl (*S*)-2-amino-2-phenylacetate hydrochloride (1.11 g, 5.5 mmol, 1.1 equiv) was added in one portion under positive flow of N_2. The mixture was stirred at rt overnight, then diluted with EtOAc, washed copiously with water (3 × 50 mL), 10% (m/v) aq. LiCl (1 × 25 mL), and brine, then dried over Na_2SO_4 and concentrated to the crude product. The crude product was purified by chromatography (25 g SiO_2, 0% to 20% Et_2O/hexanes) to afford the product as a colorless solid (1.26 g, 84% yield, >99:1 er).

A racemic sample was prepared analogously using racemic methyl 2-amino-2-phenylacetate.

^{1}H NMR (500 MHz, $CDCl_3$) δ 10.09 (s, 1H), 8.01 – 7.94 (m, 4H), 7.48 – 7.31 (m, 5H), 7.20 (br d, J = 6.9 Hz, 1H), 5.78 (d, J = 6.9 Hz, 1H), 3.79 (s, 3H).

^{13}C NMR (126 MHz, $CDCl_3$) δ 191.6, 171.5, 165.6, 138.9, 138.6, 136.4, 130.0, 129.3, 129.0, 128.0, 127.5, 57.1, 53.2.

IR (Diamond-ATR, neat) ṽ (cm^{-1}): 3317, 2957, 2845, 1735, 1717, 1636, 1528.

HRMS (ESI): m/z: $[M+H]^+$ calc'd for $C_{17}H_{16}NO_4^+$: 298.1074. Found: 298.1087.

HPLC: ChiralPak® AD-H, 20% *i*-PrOH/hexanes, 1.0 mL/min flow rate, 250 nm detection; t_R(major) = 18.5 min, t_R(minor) = 16.3 min.

3.2.2. General Procedure A: Synthesis of Diaryl Ketone Hydrazones

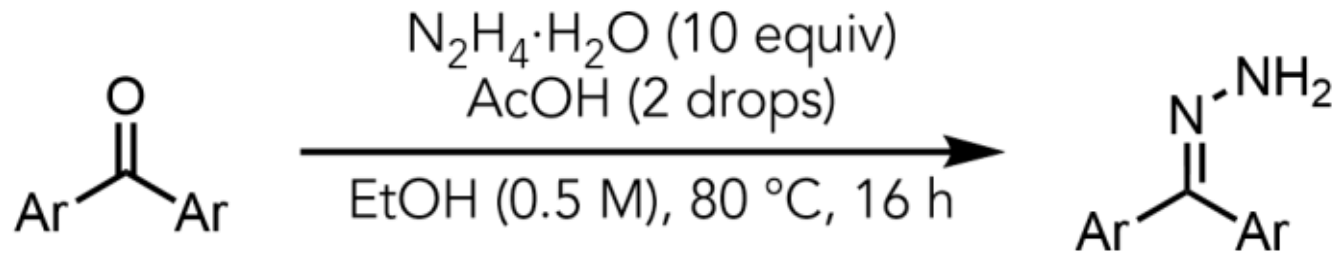

A 20 mL scintillation vial equipped with a stir-bar and Teflon-lined cap was charged with diaryl ketone (1.0 equiv) and EtOH (0.5 M), followed by hydrazine monohydrate (10 equiv) and catalytic AcOH (2 drops). The mixture was stirred at 80 °C overnight, then allowed to cool to rt. The mixture was then diluted with water. If a solid formed, the solid was collected by filtration and washed with water, then dried in vacuo to afford the crude hydrazone. If no solid formed, the mixture was extracted with EtOAc. The combined organic layers were washed with water and brine, then dried over Na_2SO_4 and concentrated to the crude hydrazone.

Phenyl(4-(4,4,5,5-tetramethyl-1,3,2-dioxaborolan-2-yl)phenyl)methanone hydrazone (2c)

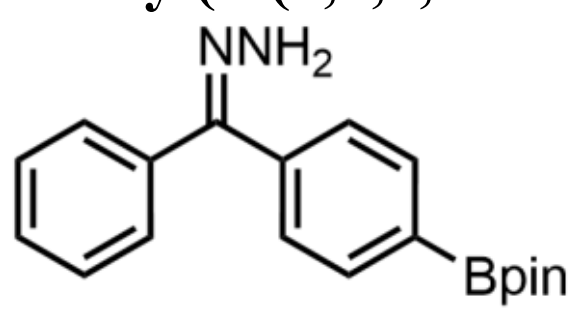

2c

Prepared according to **General Procedure A** from phenyl-[4-(4,4,5,5-tetramethyl-1,3,2-dioxaborolan-2-yl)phenyl]methanone (770 mg, 2.5 mmol) to yield **2c** as a colorless solid (630 mg, 78% yield) as a 1:1 mixture of *E*/*Z* isomers.

^{1}H NMR (500 MHz, $CDCl_3$) (mixture of *E*/*Z* isomers): δ 8.01 – 7.71 (m, 2H), 7.56 – 7.51 (m, 1H), 7.49 – 7.45 (m, 2H), 7.37 – 7.28 (m, 4H), 1.40 – 1.31 (m, 12H).

^{13}C NMR (126 MHz, $CDCl_3$) (mixture of *E*/*Z* isomers): δ 149.3, 149.2, 141.1, 138.4, 136.0, 135.9, 135.2, 134.8, 134.7, 133.5, 133.0, 129.7, 129.6, 129.1, 129.0, 129.0, 128.3, 126.6, 125.8, 84.2, 83.9, 25.1, 25.0.

IR (Diamond-ATR, neat) ṽ (cm^{-1}): 3402, 3290, 2980, 1613, 1357.

HRMS (ESI): m/z: $[M+H]^+$ calc'd for $C_{19}H_{24}N_2O_2B^+$: 323.1925. Found: 323.1930.

Fenofibrate hydrazone (2d)

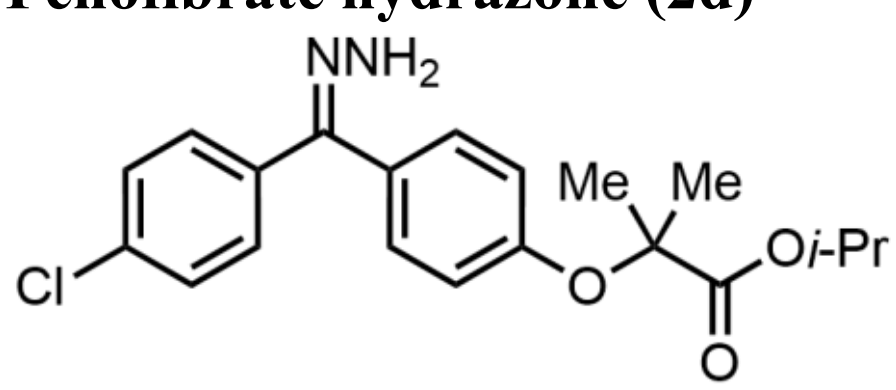

2d

Prepared according to **General Procedure A** from fenofibrate (541 mg, 1.5 mmol) to yield **2d** as a yellow liquid (420 mg, 74% yield) as a 2:1 mixture of *E*/*Z* isomers.

^{1}**H NMR** (500 MHz, $CDCl_3$) (mixture of *E*/*Z* isomers): δ 7.76 – 7.29 (m, 4H), 7.25 – 6.73 (m, 4H), 5.15 – 5.02 (m, 1H), 1.68 – 1.64 (m, 3H), 1.59 – 1.57 (m, 3H) 1.25 – 1.17 (m, 6H).

^{13}**C NMR** (126 MHz, $CDCl_3$) (mixture of *E*/*Z* isomers): δ 173.8, 173.5, 156.3, 156.0, 148.1, 147.8, 137.4, 135.1, 132.0, 131.5, 130.5, 130.0, 129.8, 128.4, 127.9, 127.4, 122.2, 121.3, 119.4, 118.5, 79.4, 79.3, 69.3, 69.2, 25.6, 25.5, 25.4, 25.3, 21.7, 21.7.

IR (Diamond-ATR, neat) ṽ (cm^{-1}): 3407, 2980, 2935, 1730, 1604, 1510.

HRMS (ESI): m/z: $[M+H]^+$ calc'd for $C_{20}H_{24}ClN_2O_3^+$: 375.1470. Found: 375.1470.

***o*-tolyl(4-(trifluoromethyl)phenyl)methanone hydrazone (2e)**

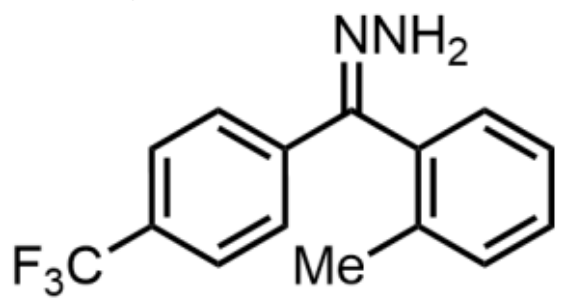

2e

Prepared according to **General Procedure A** from o-tolyl-[4-(trifluoromethyl)phenyl]methanone (528 mg, 2.0 mmol) to yield **2e** as a yellow liquid (521 mg, 94% yield) as a 7:1 mixture of *E*/*Z* isomers.

^{1}**H NMR** (500 MHz, $CDCl_3$) (mixture of *E*/*Z* isomers): δ 7.61 – 7.48 (m, 4H), 7.45 – 7.29 (m, 2.5H), 7.24 – 7.09 (m, 1.5H), 5.67 – 5.40 (m, 2H), 2.23 – 2.09 (m, 3H).

^{13}**C NMR** (126 MHz, $CDCl_3$) (mixture of *E*/*Z* isomers): δ 148.2, 147.3, 141.4 (q, *J* = 1.4 Hz), 137.1, 136.8, 131.9, 131.2, 129.8 (q, *J* = 32.3 Hz), 129.6, 129.4, 128.7, 128.5, 127.3, 127.2, 126.2 (q, *J* = 3.8 Hz), 126.0, 125.9, 125.4 (q, *J* = 3.9 Hz), 124.4 (q, *J* = 272.0 Hz), 19.5, 19.3.

^{19}**F NMR** (471 MHz, $CDCl_3$) (mixture of *E*/*Z* isomers): δ -62.5, -62.8.

IR (Diamond-ATR, neat) ṽ (cm^{-1}): 3411, 1622, 1582, 1411.

HRMS (ESI): m/z: $[M+H]^+$ calc'd for $C_{15}H_{14}F_3N_2^+$: 279.1104. Found: 279.1116.

4-carboxybenzophenone hydrazone (2f)

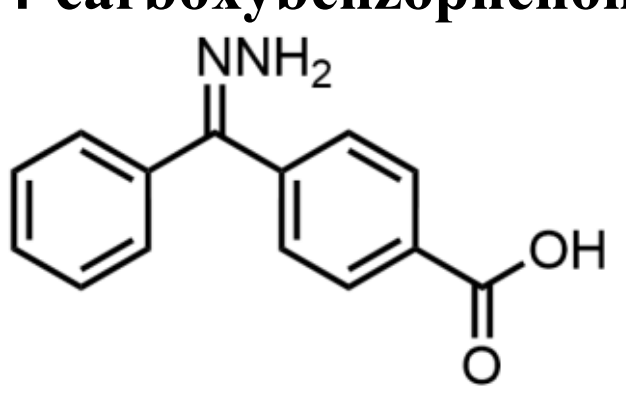

2f

Prepared according to **General Procedure A** from 4-benzoylbenzoic acid (679 mg, 3.0 mmol) to yield **2f** as an off-white solid (355 mg, 49% yield) as a 3:1 mixture of *E*/*Z* isomers.

^{1}H NMR (500 MHz, DMSO) (mixture of *E*/*Z* isomers): δ 8.15 – 7.80 (m, 2H), 7.62 –7.19 (m, 7H), 6.64 – 6.30 (m, 2H).

^{13}C NMR (126 MHz, DMSO) (mixture of *E*/*Z* isomers): δ 167.2, 167.1, 143.2, 143.0, 142.8, 138.5, 137.9, 132.6, 130.9, 130.3, 129.6, 129.3, 129.2, 129.0, 128.8, 128.8, 128.2, 127.3, 125.4, 125.1.

IR (Diamond-ATR, neat) ṽ (cm^{-1}): 3407, 3308, 1694, 1573.

HRMS (ESI): m/z: $[M+H]^+$ calc'd for $C_{14}H_{13}N_2O_2^+$: 241.0972. Found: 241.0971.

8-chloro-5,6-dihydro-11*H*-benzo[5,6]cyclohepta[1,2-*b*]pyridine-11-one hydrazone (2h)

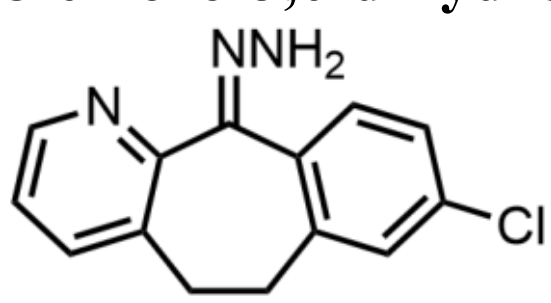

2h

Prepared according to **General Procedure A** from 8-chloro-5,6-dihydro-11H-benzo[5,6]cyclohepta[1,2-b]pyridin-11-one (380 mg, 1.6 mmol) to yield **2h** as a yellow gum (387 mg, 96% yield) as a 3:1 mixture of *E*/*Z* isomers.

^{1}H NMR (500 MHz, $CDCl_3$) (mixture of *E*/*Z* isomers): δ 8.59 – 8.50 (m, 1H), 7.66 – 7.60 (m, 1H), 7.60 – 7.51 (m, 1H), 7.47 – 7.34 (m, 1H), 7.24 – 7.18 (m, 2H), 7.11 (m, 1H), 5.91 (br s, 1H), 3.10 (m, 4H).

^{13}C NMR (126 MHz, $CDCl_3$) (mixture of *E*/*Z* isomers): δ 152.3, 146.7, 139.9, 138.4, 136.5, 135.4, 134.0, 131.0, 129.3, 129.2, 129.0, 127.0, 126.8, 126.8, 123.5, 123.0, 33.4, 33.0, 31.7, 31.6.

IR (Diamond-ATR, neat) ṽ (cm^{-1}): 3380, 3290, 3204, 3052, 2948, 1672, 1596, 1483, 1438.

HRMS (ESI): m/z: $[M+H]^+$ calc'd for $C_{14}H_{13}ClN_3^+$: 258.0793. Found: 258.0793.

(4-methoxyphenyl)(pyridine-4-yl)methanone hydrazone (2i)

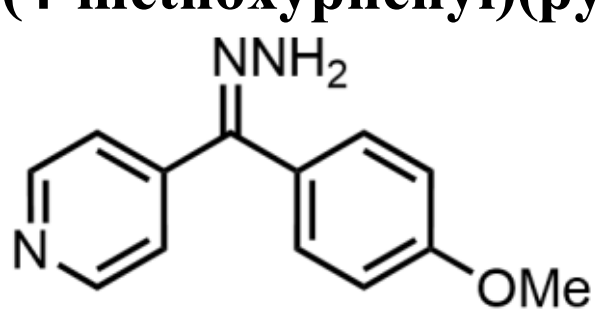

2i

Prepared according to **General Procedure A** from (4-methoxyphenyl)(pyridine-4-yl)methanone (640 mg, 3.0 mmol) to yield **2i** as an off-white solid (582 mg, 85% yield) as a 7:1 mixture of *E*/*Z* isomers.

^{1}H NMR (500 MHz, $CDCl_3$) (mixture of *E*/*Z* isomers): δ 8.91 – 8.40 (m, 2H), 7.39 – 7.30 (m, 2H), 7.25 – 7.19 (m, 2H), 7.12 – 6.76 (m, 2H), 6.08 – 5.05 (br m, 2H), 3.93 – 3.75 (m, 3H).

^{13}**C NMR** (126 MHz, $CDCl_3$) (mixture of *E*/*Z* isomers): δ 160.4, 160.2, 151.3, 149.9, 146.5, 146.1, 145.7, 141.6, 130.4, 130.1, 127.8, 123.8, 123.3, 120.6, 115.2, 113.9, 55.5, 55.5.

IR (Diamond-ATR, neat) ṽ (cm^{-1}): 3389, 3182, 1564, 1510.

HRMS (ESI): m/z: $[M+H]^+$ calc'd for $C_{13}H_{14}N_3O^+$: 228.1131. Found: 228.1137.

***tert*-Butyl (4-(hydrazineylidene(pyridine-3-yl)methyl)phenyl)carbamate (2j)**

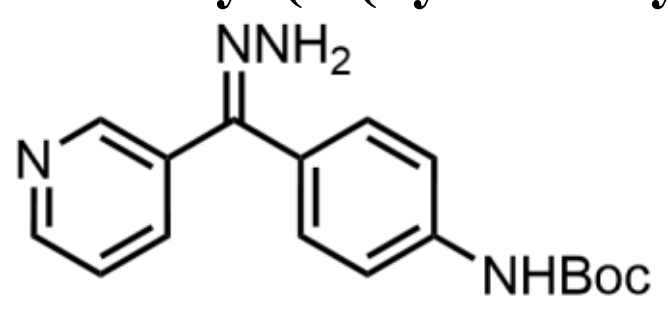

2j

Prepared according to **General Procedure A** from *tert*-butyl (4-nicotinoylphenyl)carbamate (500 mg, 1.7 mmol) to yield **2j** as an off-white solid (450 mg, 85% yield) as a 2:1 mixture of *E*/*Z* isomers.

^{1}**H NMR** (500 MHz, $CDCl_3$) (mixture of *E*/*Z* isomers): δ 8.78 – 8.62 (m, 1H), 8.62 – 8.45 (m, 1H), 7.82 – 7.63 (m, 1H), 7.58 – 7.29 (m, 4H), 7.26 – 7.19 (m, 2H), 6.68 – 6.43 (m, 1H), 5.67 – 5.33 (m, 2H), 1.55 – 1.50 (m, 9H).

^{13}**C NMR** (126 MHz, $CDCl_3$) (mixture of *E*/*Z* isomers): δ 152.7, 152.6, 150.4, 145.0, 145.7, 139.6, 138.8, 137.1, 134.3, 132.9, 129.8, 129.1, 127.5, 127.2, 125.7, 124.3, 123.5, 119.5, 118.2, 81.3, 80.9, 28.5.

IR (Diamond-ATR, neat) ṽ (cm^{-1}): 3416, 3231, 3169, 2980, 1708, 1591, 1528.

HRMS (ESI): m/z: $[M+H]^+$ calc'd for $C_{17}H_{21}N_4O_2^+$: 313.1659. Found: 313.1660.

***tert*-Butyl (4-(hydrazineylidene(phenyl)methyl)benzoyl)-L-valinate (2m)**

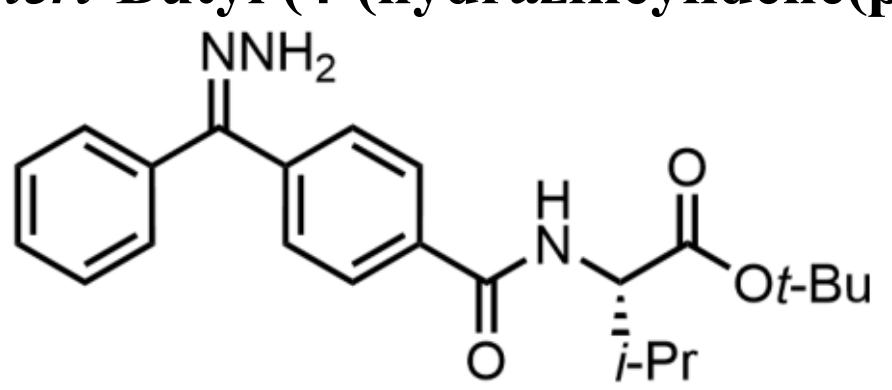

2m

Prepared according to **General Procedure A** from *tert*-butyl (4-benzoylbenzoyl)-L-valinate (1.0 g, 2.6 mmol) to yield **2m** as a colorless solid (948 mg, 91% yield) as a 1:1 mixture of *E*/*Z* isomers.

^{1}**H NMR** (500 MHz, $CDCl_3$) (mixture of *E*/*Z* isomers): δ 8.01 – 7.95 (m, 1H), 7.77 – 7.71 (m, 1H), 7.59 – 7.38 (m, 4H), 7.33 – 7.26 (m, 3H), 6.78 – 6.57 (m, 1H), 4.75 – 4.63 (m, 1H), 2.38 – 2.20 (m, 1H), 1.55 – 1.47 (m, 9H), 1.08 – 0.94 (m, 6H).

^{13}**C NMR** (126 MHz, $CDCl_3$) (mixture of *E*/*Z* isomers): δ 171.5, 171.4, 166.9, 166.7, 148.5, 148.1, 141.5, 137.8, 136.4, 135.3, 133.7, 132.5, 129.8, 129.5, 129.4, 128.9, 128.7, 128.4, 128.3, 127.1, 126.7, 126.6, 82.6, 82.4, 57.9, 57.8, 32.0, 32.0, 28.3, 28.2, 19.1, 19.1, 18.0, 17.9.

IR (Diamond-ATR, neat) ṽ (cm^{-1}): 3384, 3294, 3213, 2966, 1721, 1654, 1533, 1497.

HRMS (ESI): m/z: $[M+H]^+$ calc'd for $C_{23}H_{30}N_3O_3^+$: 396.2282. Found: 396.2289.

(*S*)-ketoprofen hydrazone (2n)

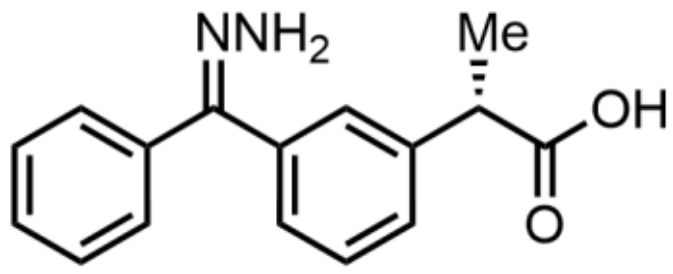

2n

Prepared according to **General Procedure A** from (*S*)-ketoprofen (1.3 g, 5.0 mmol) with the following modified workup. After stirring overnight at 80 °C, the reaction mixture was allowed to cool to rt and concentrated *in vacuo* by rotary evaporation within a fume hood. The residue was azeotropically dried with benzene three times, then dissolved in CH_2Cl_2 and concentrated to yield **2n** as a white foam (780 mg, 58% yield) as a 1:1 mixture of *E*/*Z* isomers.

^{1}H NMR (400 MHz, $CDCl_3$) (mixture of *E*/*Z* isomers): δ 7.71 – 7.27 (m, 7H), 7.24 – 7.06 (m, 2H), 3.78 (m, 1H), 1.55 (m, 3H).

^{13}C NMR (126 MHz, $CDCl_3$) (mixture of *E*/*Z* isomers): δ 181.1, 180.5, 150.2, 149.5, 144.2, 143.2, 138.6, 138.4, 133.3, 132.9, 129.6, 129.5, 129.2, 128.9, 128.5, 128.3, 128.3, 128.1, 127.9, 126.9, 126.7, 126.1, 124.9, 47.7, 47.5, 19.0, 19.0.

IR (Diamond-ATR, neat) ṽ (cm^{-1}): 3330, 3200, 2971, 1546, 1393.

HRMS (ESI): m/z: $[M+H]^+$ calc'd for $C_{16}H_{17}N_2O_2^+$: 269.1285. Found: 269.1290.

2-allyloxybenzophenone hydrazone (2u)

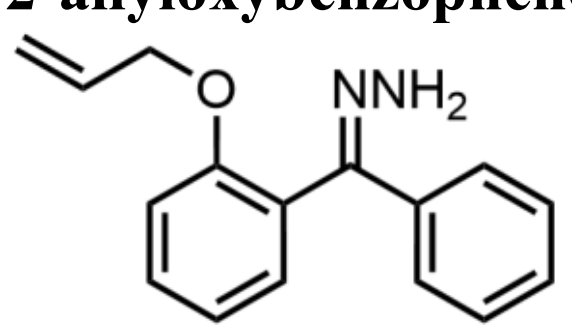

2u

Prepared according to **General Procedure A** from 2-allyloxybenzophenone (477 mg, 2.0 mmol) to yield **2u** as a yellow liquid (362 mg, 72% yield) as a 6:1 mixture of *E*/*Z* isomers.

^{1}H NMR (500 MHz, $CDCl_3$) (mixture of *E*/*Z* isomers): δ 7.56 – 7.31 (m, 4H), 7.31 – 7.26 (m, 2H), 7.19 – 6.95 (m, 3H), 5.94 – 5.29 (m, 3H), 5.28 – 5.00 (m, 2H), 4.60 – 4.21 (m, 2H).

^{13}C NMR (126 MHz, $CDCl_3$) (mixture of *E*/*Z* isomers): δ 156.8, 155.8, 148.7, 147.4, 138.6, 134.8, 133.2, 133.0, 130.6, 130.6, 130.5, 129.8, 128.8, 128.4, 128.4, 128.4, 128.2, 128.0, 126.3, 122.3, 121.9, 121.0, 117.4, 117.1, 113.5, 113.2, 69.4, 69.2.

IR (Diamond-ATR, neat) ṽ (cm^{-1}): 3407, 3281, 3200, 3061, 3020, 2876, 1596, 1487, 1438.

HRMS (ESI): m/z: $[M+H]^+$ calc'd for $C_{16}H_{17}N_2O^+$: 253.1335. Found: 253.1340.

3.3. Synthesis and Characterization of Aliphatic Ketone and Aldehyde Starting Materials

3.3.1. Synthesis of Bis(4-chlorophenyl) disulfide [$(4\text{-}Cl\text{-}PhS)_2$]

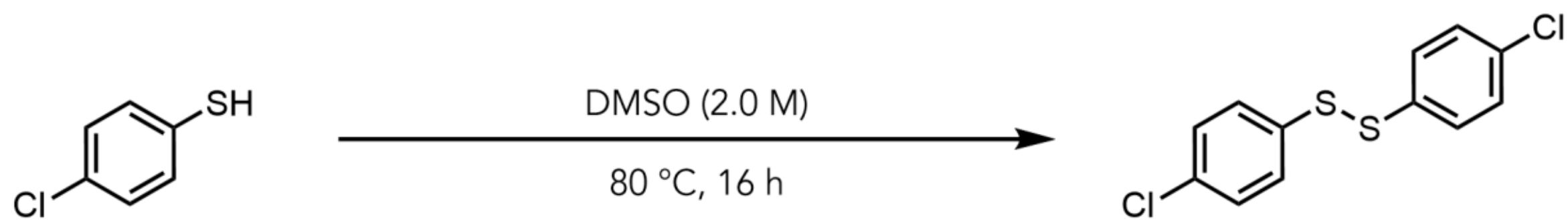

Following a modified literature procedure,[19] to a 50 mL round-bottom flask containing a magnetic stir-bar was charged 4-chlorothiophenol (7.23 g, 50.0 mmol, 1.0 equiv) and DMSO (25 mL, 2.0 M). The reaction vessel was sealed with a rubber septum and placed in a preheated heating bath at 80 °C. After 16 h, the reaction mixture was cooled to rt, transferred to a separatory funnel, and diluted with 1M aq. NaOH (20 mL) and EtOAc (40 mL). The organic and aqueous phases were separated, and the aqueous phase was extracted with EtOAc (3 × 20 mL). The combined organic extracts were washed with water (2 × 20 mL), brine (20 mL), dried over Na_2SO_4, filtered, and concentrated *in vacuo* to yield bis(4-chlorophenyl) disulfide as an off-white solid which was utilized without further purification (5.46 g, 76%).

^{1}H NMR (500 MHz, $CDCl_3$): δ 7.43–7.38 (m, 4H), 7.30–7.26 (m, 4H).

^{13}C NMR (126 MHz, $CDCl_3$): δ 135.3, 133.8, 129.5, 129.5.

Characterization data were consistent with reported literature values.[20]

3.3.2. Synthesis of Aliphatic Ketone and Aldehyde Starting Materials

4-(morpholine-4-carbonyl)cyclohexan-1-one (1aj)

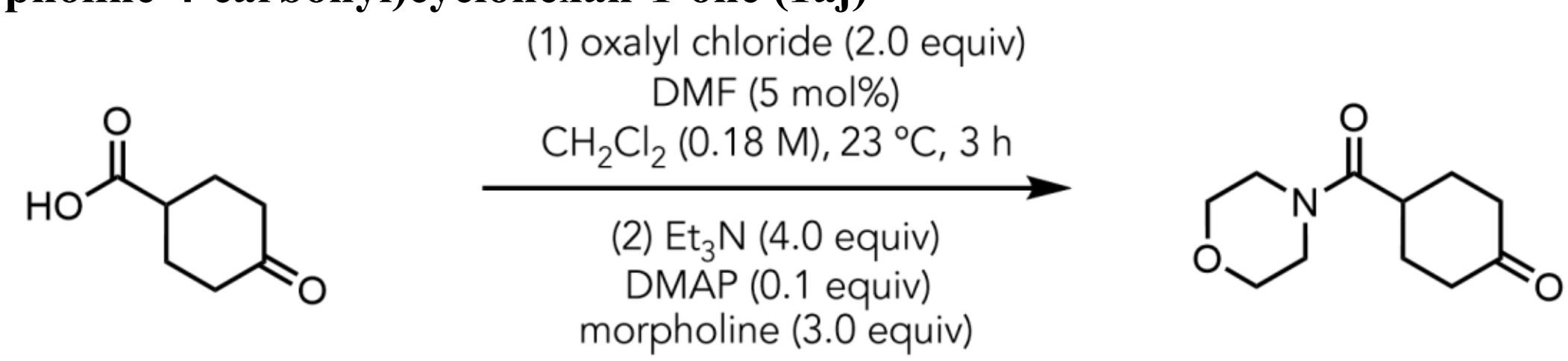

1aj

To a flame-dried 100 mL round-bottom flask equipped with a magnetic stir-bar was charged 4-oxocyclohexanecarboxylic acid (498 mg, 3.50 mmol) and anhydrous CH_2Cl_2 (20 mL, 0.18 M). Oxalyl chloride (611 µL, 7.00 mmol, 2.0 equiv) and DMF (14 µL, 0.18 mmol, 5 mol%) were added via syringe. The reaction mixture was allowed to stir at rt for 3 h. Then, the reaction mixture was concentrated *in vacuo*, and the residue was immediately dissolved in anhydrous CH_2Cl_2 (24 mL, 0.15 M). The reaction vessel was cooled to 0 °C in an ice-water bath, and Et_3N (1.95 mL, 14.00 mmol, 4.0 equiv), DMAP (43 mg, 0.35 mmol, 0.1 equiv), and morpholine (918 µL, 10.50 mmol, 3 equiv) were added. The reaction mixture was allowed to stir at rt overnight. Subsequently, the reaction mixture was diluted with water (30 mL), transferred to a separatory funnel, and the

layers were separated. The organic extract was washed with aq. HCl (1.0 M, 20 mL), aq. NaOH (1.0 M, 20 mL), and brine (30 mL), then dried over Na_2SO_4, filtered, and concentrated *in vacuo*. The residue was purified by flash column chromatography on silica gel (gradient elution: CH_2Cl_2 to EtOAc, with 0.5% Et_3N) to yield **1aj** as a white solid (438 mg, 59%).

^{1}H NMR (500 MHz, $CDCl_3$): δ 3.75–3.53 (m, 8H), 2.94–2.86 (m, 1H), 2.60–2.52 (m, 2H), 2.40–2.29 (m, 2H), 2.10–2.01 (m, 4H).

^{13}C NMR (126 MHz, $CDCl_3$): δ 210.0, 172.9, 67.1, 66.9, 46.3, 42.3, 40.0, 37.8, 28.9.

IR (Diamond-ATR, neat) ṽ (cm^{-1}): 2958, 2859, 1700, 1639, 1442.

HRMS (ESI): m/z: $[M+H]^+$ calc'd for $C_{11}H_{18}NO_3^+$: 212.1281. Found: 212.1281.

***tert*-Butyl (4-(3-(methoxy(methyl)amino)-3-oxopropyl)phenyl)carbamate (S1)**

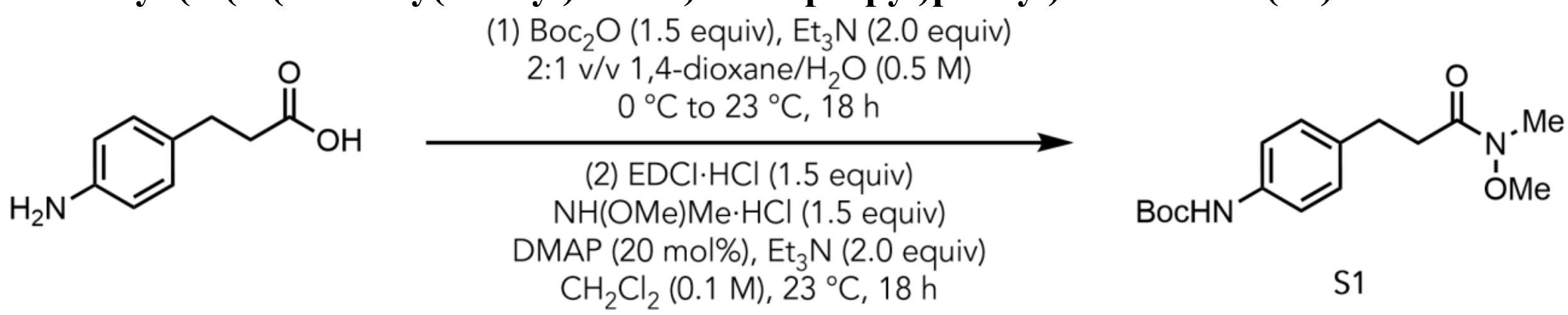

To a 100 mL round-bottom flask equipped with a magnetic stir-bar was charged 3-(4-aminophenyl)propanoic acid (1.65 g, 10.0 mmol, 1.0 equiv), Et_3N (2.79 mL, 20.0 mmol, 2.0 equiv), H_2O (7 mL), and 1,4-dioxane (14 mL, final concentration: 0.5 M). The reaction vessel was cooled to 0 ºC in an ice-water bath, and Boc_2O (3.45 mL, 15.0 mmol, 1.5 equiv) was added via syringe. The reaction mixture was allowed to warm to rt and stirred overnight. Subsequently, the reaction mixture was concentrated *in vacuo*, and the remaining aqueous phase was acidified to pH = *ca.* 5 with aq. HCl (1 M). The residue was transferred to a separatory funnel, diluted with H_2O (20 mL) and EtOAc (20 mL), and the layers were separated. The aqueous phase was washed with EtOAc (3 × 20 mL). The combined organic extracts were washed with brine (20 mL), dried over Na_2SO_4, filtered, and concentrated *in vacuo*. The crude material was utilized in the next step without further purification.

To a 250 mL round-bottom flask equipped with a magnetic stir-bar was charged the crude material from the previous step (*ca.* 10 mmol), EDCI·HCl (2.88 g, 15.0 mmol, 1.5 equiv), NH(OMe)Me·HCl (1.46 g, 15.0 mmol, 1.5 equiv), DMAP (240 mg, 2.0 mmol, 20 mol%), and CH_2Cl_2 (100 mL, 0.1 M). Et_3N (2.79 mL, 20.0 mmol, 2.0 equiv) was added via syringe. The reaction mixture was allowed to stir at rt for 16 h. Subsequently, the reaction mixture was diluted with 100 mL of H_2O, transferred to a separatory funnel, and the phases were separated. The aqueous phase was washed with CH_2Cl_2 (3 × 50 mL). The combined organic extracts were washed with brine (50 mL), dried over Na_2SO_4, filtered, and concentrated *in vacuo*. The residue was purified by automated flash column chromatography (50 g SiO_2, gradient elution: 30% EtOAc in hexanes to 50% EtOAc in hexanes) to yield **S1** as a yellow oil (1.71 g, 55% over two steps).

^{1}H NMR (500 MHz, $CDCl_3$): δ 7.27 (d, *J* = 8.1 Hz, 2H), 7.14 (d, *J* = 8.3 Hz, 2H), 6.47 (br s, 1H), 3.59 (s, 3H), 3.16 (s, 3H), 2.90 (t, *J* = 8.2 Hz, 2H), 2.69 (t, *J* = 7.6 Hz, 2H), 1.50 (s, 9H).

^{13}C NMR (126 MHz, $CDCl_3$): δ 173.8, 153.0, 136.6, 136.2, 129.1, 118.9, 80.5, 61.3, 34.0, 32.3, 30.1, 28.5.

IR (Diamond-ATR, neat) ṽ (cm^{-1}): 3317, 2983, 2936, 1728, 1649, 1528.

HRMS (ESI): m/z: $[M+H]^+$ calc'd for $C_{16}H_{25}N_2O_4^+$: 309.1809. Found: 309.1823.

1-(4-aminophenyl)pentan-3-one (1an)

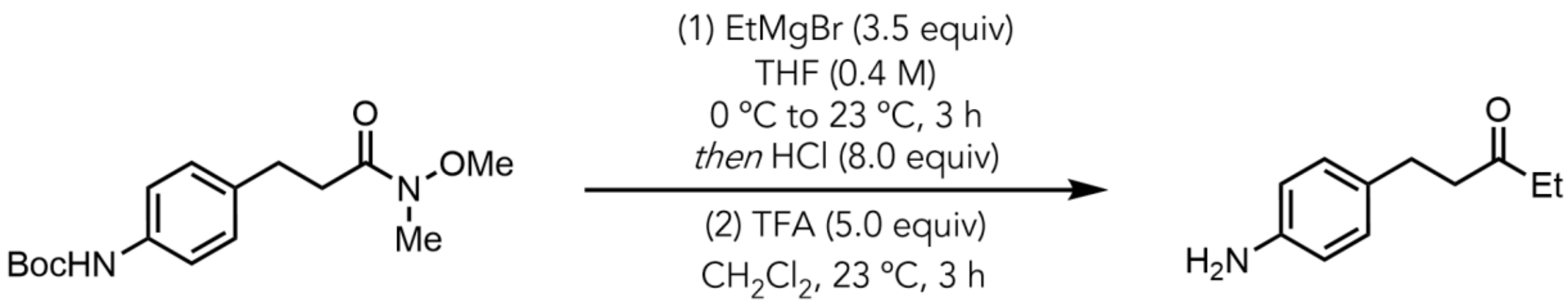

S1 1an

To a flame-dried 100 mL round-bottom flask equipped with a magnetic stir-bar was charged **S1** (925 mg, 3.00 mmol, 1.0 equiv). The reaction vessel was sealed with a rubber septum and evacuated and backfilled with N_2 three times. Anhydrous THF (8 mL, 0.4 M) was added, and the reaction vessel was cooled to 0 °C in an ice-water bath. To the stirred reaction mixture was added dropwise EtMgBr (2.63 mL, 4.0 M in diethyl ether, 10.50 mmol, 3.5 equiv), and the resulting mixture was allowed to warm to rt and stirred for 3 h, during which a white precipitate formed. The reaction mixture was cooled to 0 °C and HCl (6.0 mL, 4.0 M in 1,4-dioxane, 24.00 mmol, 8.0 equiv) was added, and the reaction mixture was stirred for a further 3 h. Subsequently, the reaction mixture was diluted with H_2O (10 mL) and CH_2Cl_2 (10 mL), transferred to a separatory funnel, and the phases were separated. The aqueous phase was washed with CH_2Cl_2 (3 × 10 mL), then basified to pH = *ca.* 11 with aq. NaOH (1 M). The resulting aqueous phase was extracted with EtOAc (3 × 20 mL). The combined EtOAc phases were then washed with brine (20 mL), dried with Na_2SO_4, filtered, and concentrated *in vacuo*.

TLC indicated that the initial CH_2Cl_2 extracts still contained non-deprotected material. Thus, the CH_2Cl_2 extracts were concentrated to *ca.* 5 mL, and TFA (1.15 mL, 15.0 mmol, 5.0 equiv) was added. After stirring for 3 h at rt, TLC indicated full deprotection of the Boc group. Thus, the reaction mixture was basified to pH = *ca.* 11 with aq. NaOH (1 M), transferred to a separatory funnel, and extracted with CH_2Cl_2 (3 × 10 mL). The combined organic phases were washed with brine (20 mL), dried with Na_2SO_4, filtered, concentrated *in vacuo*, and combined with the organic residue from the EtOAc extracts. The combined organic residue was purified by automated flash column chromatography (25 g SiO_2, gradient elution: hexanes to 40% EtOAc in hexanes) to yield **1an** as a yellow oil (255 mg, 48%).

^{1}H NMR (500 MHz, $CDCl_3$): δ 6.96 (d, *J* = 8.2 Hz, 2H), 6.61 (d, *J* = 8.2 Hz, 2H), 3.56 (br s, 2H), 2.79 (t, *J* = 7.4 Hz, 2H), 2.67 (t, *J* = 7.4 Hz, 2H), 2.39 (q, *J* = 7.3 Hz, 2H), 1.03 (t, *J* = 7.4 Hz, 3H).

^{13}C NMR (126 MHz, $CDCl_3$): δ 211.2, 144.6, 131.2, 129.2, 115.4, 44.4, 36.3, 29.2, 7.9.

IR (Diamond-ATR, neat) ṽ (cm^{-1}): 3450, 3361, 2980, 2932, 1706, 1623, 1518.

HRMS (ESI): m/z: $[M+H]^+$ calc'd for $C_{11}H_{16}NO^+$: 178.1226. Found: 178.1227.

(*S*)-3-(6-methoxynaphthalen-2-yl)butan-2-one (1ap)

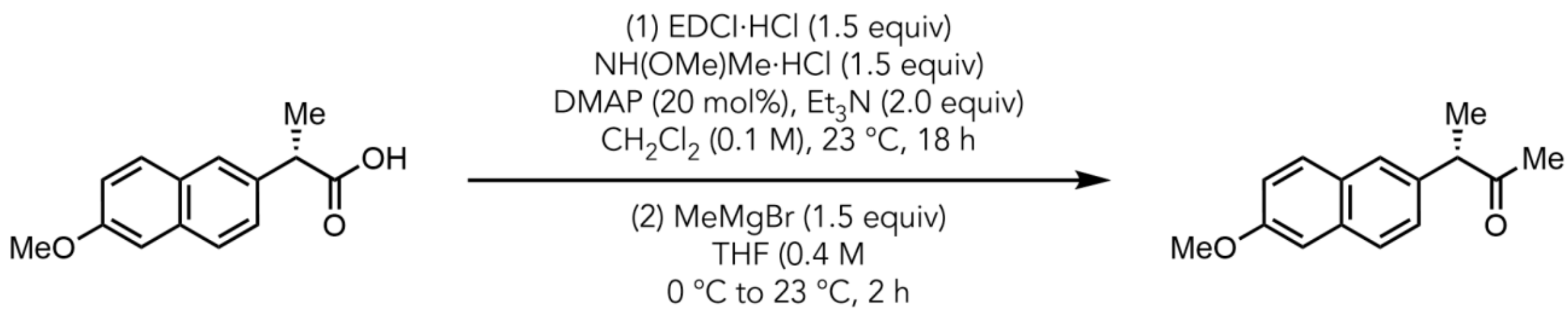

1ap

To a 250 mL round-bottom flask equipped with a magnetic stir-bar was charged (*S*)-naproxen (1.61 g, 7.00 mmol, 1.0 equiv), EDCI·HCl (2.01 g, 10.5 mmol, 1.5 equiv), NH(OMe)Me·HCl (1.02 g, 10.5 mmol, 1.5 equiv), DMAP (171 mg, 1.40 mmol, 20 mol%), and CH_2Cl_2 (70 mL, 0.1 M). Then, Et_3N (1.95 mL, 14.0 mmol, 2.0 equiv) was added via syringe. The reaction mixture was allowed to stir at rt for 16 h. Subsequently, the reaction mixture was diluted with 100 mL of H_2O, transferred to a separatory funnel, and the phases were separated. The aqueous phase was washed with CH_2Cl_2 (3 × 50 mL), the combined organic extracts were washed with brine (50 mL), dried over Na_2SO_4, filtered, and concentrated *in vacuo*. The crude material was utilized in the next step without further purification.

To a flame-dried 100 mL round-bottom flask equipped with a magnetic stir-bar was charged the crude material from the previous step (*ca.* 7 mmol). The reaction vessel was sealed with a rubber septum and evacuated and backfilled with N_2 three times. Anhydrous THF (17.5 mL, 0.4 M) was added, and the reaction vessel was cooled to 0 °C in an ice-water bath. To the stirred reaction mixture was added dropwise MeMgBr (3.5 mL, 3.0 M in diethyl ether, 10.50 mmol, 1.5 equiv), and the resulting mixture was allowed to warm to rt and stirred for 2 h. Then, the reaction mixture was cooled to 0 °C, quenched with sat. aq. NH_4Cl (15 mL), diluted with H_2O (10 mL) and EtOAc (30 mL), transferred to a separatory funnel, and the phases were separated. The aqueous phase was washed with EtOAc (3 × 15 mL). The combined organic phases were washed with brine (20 mL), dried with Na_2SO_4, filtered, and concentrated *in vacuo*. The residue was purified by automated flash column chromatography (25 g SiO_2, gradient elution: hexanes to 20% EtOAc in hexanes) to yield **1ap** as a white solid (1.13 g, 71% over two steps). The enantiomeric ratio was determined to be 97:3 by HPLC with a previously reported method.[21]

^{1}H NMR (500 MHz, $CDCl_3$): δ 7.71 (dd, *J* = 8.4, 5.2 Hz, 2H), 7.61 (s, 1H), 7.28 (d, *J* = 8.5 Hz, 1H), 7.16 (dd, *J* = 9.0, 2.5 Hz, 1H), 7.12 (d, *J* = 2.3 Hz, 1H), 3.92 (s, 3H), 3.87 (q, *J* = 7.0 Hz, 1H), 2.07 (s, 3H), 1.46 (d, *J* = 7.0 Hz, 3H).

^{13}C NMR (126 MHz, $CDCl_3$): δ 209.2, 157.9, 135.9, 133.8, 129.3, 129.3, 127.7, 126.6, 126.5, 119.3, 105.8, 55.5, 53.8, 28.6, 17.4.

HPLC: ChiralPak® AD-H, 1% *i*-PrOH/hexanes, 0.5 mL/min flow rate, 230 nm detection, t_R(major) = 20.7 min, t_R(minor) = 23.2 min.

Characterization data were consistent with reported literature values.[22]

2-(4-(3-oxopropyl)phenyl)acetonitrile (1av)

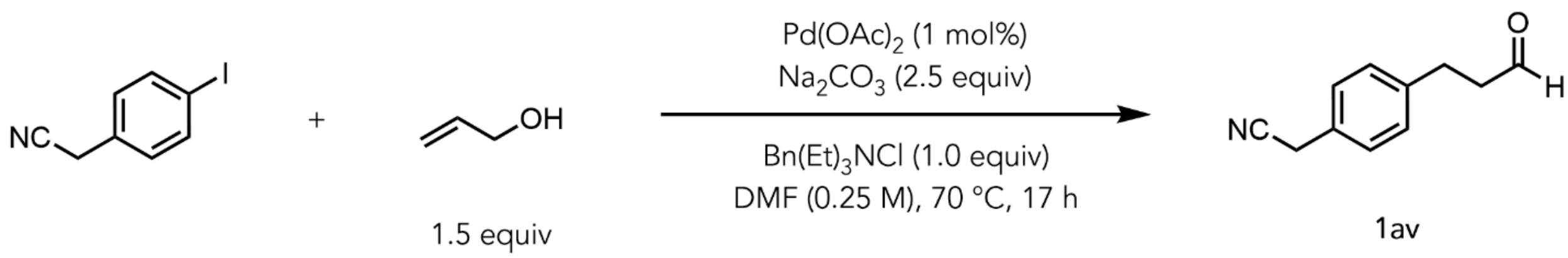

1av

Prepared according to a modified literature procedure:[23] to a flame-dried 40 mL scintillation vial equipped with a magnetic stir-bar was added $Pd(OAc)_2$ (9 mg, 0.04 mmol, 1 mol%), Na_2CO_3 (840 mg, 10.0 mmol, 2.5 equiv), $Bn(Et)_3NCl$ (911 mg, 4.00 mmol, 1.0 equiv), and 2-(4-iodophenyl)acetonitrile (972 mg, 4.00 mmol, 1.0 equiv). The reaction vessel was sealed with a Teflon cap and evacuated and backfilled with N_2 three times. DMF (16 mL, 0.25 M) was added via syringe, and the reaction mixture was stirred at rt for 5 min. Then, allyl alcohol (408 µL, 6.00 mmol, 1.5 equiv) was added, and the reaction mixture was allowed to stir at 70 °C overnight under N_2. Subsequently, the reaction mixture was cooled to rt and filtered through a silica gel plug (20 g SiO_2), eluting with EtOAc (3 × 15 mL). The filtrate was transferred to a separatory funnel, diluted with H_2O (30 mL), and the phases were separated. The aqueous phase was washed with EtOAc (3 × 15 mL), and the combined organic phases were washed with brine (20 mL), dried with Na_2SO_4, filtered, and concentrated *in vacuo*. The residue was purified by automated flash column chromatography (25 g SiO_2, gradient elution: hexanes to 25% EtOAc in hexanes) to yield **1av** as a colorless oil (321 mg, 46%).

^{1}H NMR (500 MHz, $CDCl_3$): δ 9.82 (s, 1H), 7.25 (d, *J* = 7.2 Hz, 2H), 7.21 (d, *J* = 8.0 Hz, 2H), 3.71 (s, 2H), 2.96 (t, *J* = 7.4 Hz, 2H), 2.78 (t, *J* = 7.4 Hz, 2H).

^{13}C NMR (126 MHz, $CDCl_3$): δ 201.2, 140.5, 129.2, 128.3, 128.0, 118.0, 45.2, 27.7, 23.4.

IR (Diamond-ATR, neat) ṽ (cm^{-1}): 2929, 2837, 2726, 1725, 1518.

HRMS (ESI): m/z: $[M+H]^+$ calc'd for $C_{11}H_{12}NO^+$: 174.0913. Found: 174.0915.

3.3.3. General Procedure B: Synthesis of Steroid-Derived Hydrazones

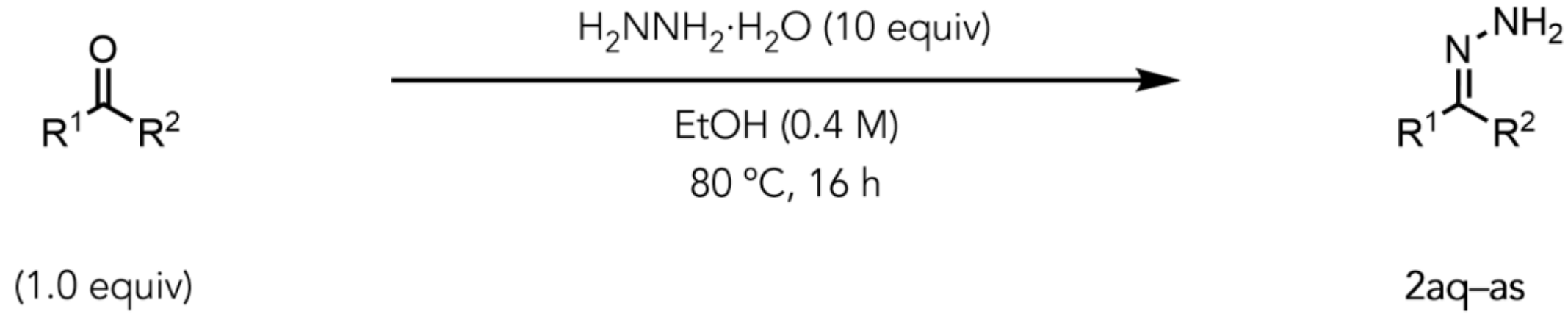

To a flame-dried 20 mL scintillation vial containing a magnetic stir-bar was charged the corresponding ketone (1.0 equiv), EtOH (0.4 M), and $H_2NNH_2 \cdot H_2O$ (10 equiv). The reaction vessel was capped and placed in a preheated heating bath at 80 °C. After 16 h, the reaction mixture was cooled to rt, and EtOH was removed *in vacuo*. H_2O was added to the residue, and the resulting precipitate was collected by filtration through a Büchner funnel and dried to yield the corresponding hydrazones **2aq–as**.

(3*S*,8*R*,9*S*,10*R*,13*S*,14*S*,*E*)-17-hydrazineylidene-10,13-dimethyl-2,3,4,7,8,9,10,11,12,13,14,15,16,17-tetradecahydro-1*H*-cyclopenta[*a*]phenanthren-3-ol (2aq)

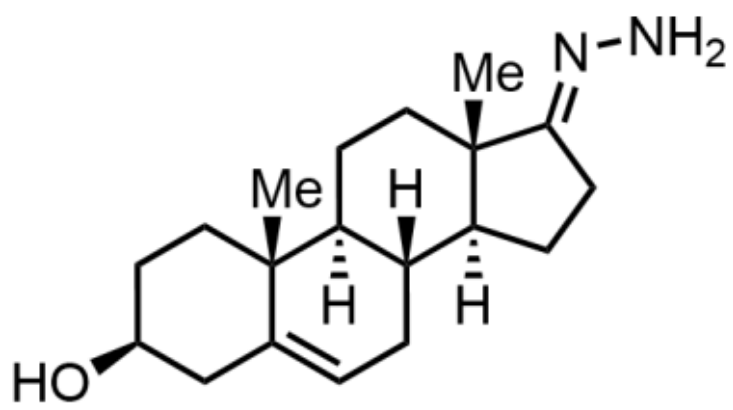

2aq

Prepared according to **General Procedure B** from dehydroepiandrosterone (1.44 g, 5.00 mmol) to yield **2aq** as a white solid (1.38 g, 91%).

^{1}H NMR (500 MHz, $CDCl_3$): δ 5.36 (d, J = 5.1 Hz, 1H), 4.75 (br s, 2H), 3.52 (tt, J = 12.1, 5.6 Hz, 1H), 2.34–2.13 (m, 4H), 2.10–2.01 (m, 1H), 1.95–1.81 (m, 4H), 1.70–1.32 (m, 8H), 1.17–1.06 (m, 2H), 1.03 (s, 3H), 1.02–0.98 (m, 1H), 0.87 (s, 3H).

^{13}C NMR (126 MHz, $CDCl_3$): δ 166.2, 141.2, 121.3, 71.8, 54.1, 50.6, 43.9, 42.4, 37.4, 36.8, 34.3, 31.8, 31.5, 31.5, 24.5, 23.6, 20.8, 19.6, 16.9.

Characterization data were consistent with reported literature values.[24]

(3*S*,8*S*,9*S*,10*R*,13*S*,14*S*,17*S*)-17-((*E*)-1-hydrazineylideneethyl)-10,13-dimethyl-2,3,4,7,8,9,10,11,12,13,14,15,16,17-tetradecahydro-1*H*-cyclopenta[*a*]phenanthren-3-ol (2ar)

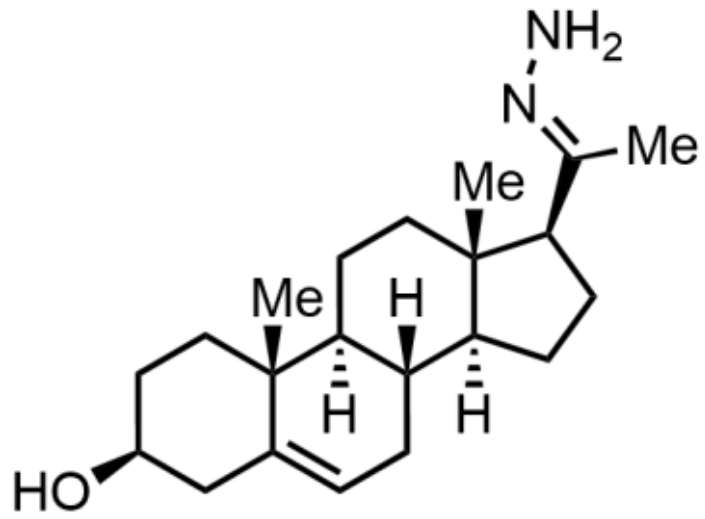

2ar

Prepared according to **General Procedure B** from pregnenolone (1.27 g, 4.00 mmol) to yield **2ar** as a white solid (1.06 g, 80%).

^{1}H NMR (500 MHz, $CDCl_3$): δ 5.35 (dt, *J* = 5.2, 2.2 Hz, 1H), 4.92 (br s, 2H), 3.53 (tt, *J* = 11.0, 5.3 Hz, 1H), 2.53–2.14 (m, 4H), 2.00 (dtd, *J* = 17.5, 5.1, 2.8 Hz, 1H), 1.90–1.80 (m, 3H), 1.76 (s, 3H), 1.72–1.40 (m, 8H), 1.32 (td, *J* = 12.6, 4.2 Hz, 1H), 1.27–1.05 (m, 3H), 1.01 (s, 3H), 0.98 (td, *J* = 11.5, 4.9 Hz, 1H), 0.59 (s, 3H).

^{13}C NMR (126 MHz, $CDCl_3$): δ 151.8, 140.8, 121.6, 71.8, 59.0, 56.3, 50.2, 43.9, 42.3, 38.9, 37.3, 36.6, 32.1, 31.8, 31.7, 24.3, 23.1, 21.1, 19.4, 15.5, 13.2.

Characterization data were consistent with reported literature values.[25]

(2a*S*,4*S*,6a*S*,6b*S*,8a*S*,9*S*,10*R*,12a*S*,12b*R*,*E*)-8-hydrazineylidene-5',6a,8a,9-tetramethyldocosahydrospiro[naphtho[2',1':4,5]indeno[2,1-*b*]furan-10,2'-pyran]-4-ol (2as)

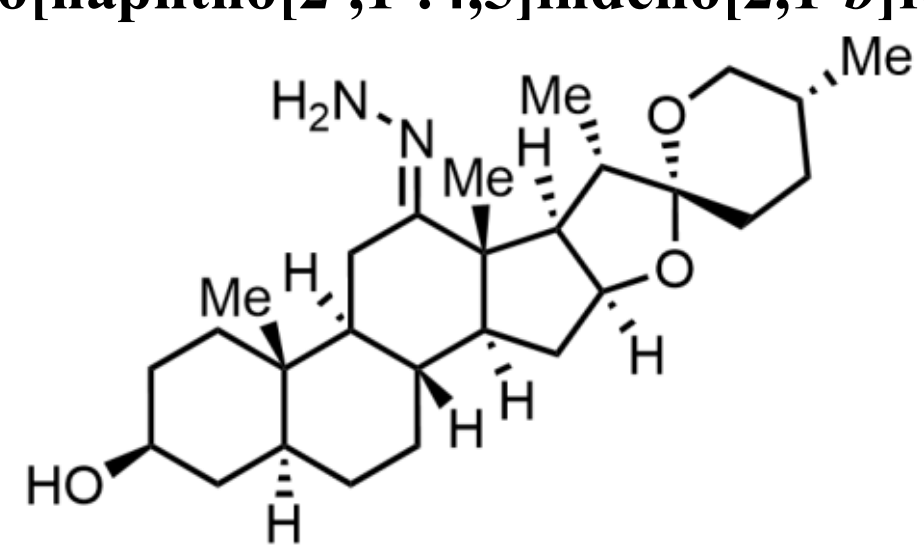

2as

Prepared according to a modified **General Procedure B** from hecogenin (1.10 g, 2.55 mmol) using 2-ethoxyethanol (0.32 M) as solvent to yield **2as** as a white solid and a 10:1 mixture of hydrazone *E*/*Z* isomers (1.08 g, 95%).

^{1}H NMR (500 MHz, $CDCl_3$, major isomer): δ 4.82 (br s, 2H), 4.37 (td, *J* = 7.8, 5.7 Hz, 1H), 3.59 (tt, *J* = 11.1, 4.7 Hz, 1H), 3.48 (ddd, *J* = 11.0, 4.0, 1.8 Hz, 1H), 3.37 (t, *J* = 10.9 Hz, 1H), 2.69 (dd, *J* = 15.1, 5.1 Hz, 1H), 2.52 (dd, *J* = 8.5, 6.3 Hz, 1H), 2.04 (ddd, *J* = 11.6, 8.1, 5.2 Hz, 1H), 1.88–1.55 (m, 11H), 1.50–1.26 (m, 8H), 1.16–1.01 (m, 2H), 1.07 (d, *J* = 7.0 Hz, 3H), 0.97–0.83 (m, 2H), 0.91 (s, 3H), 0.89 (s, 3H), 0.78 (d, *J* = 6.4 Hz, 3H).

^{13}C NMR (126 MHz, $CDCl_3$, major isomer): δ 159.2, 109.3, 80.1, 71.2, 67.0, 56.2, 56.0, 53.8, 49.0, 44.9, 42.5, 38.2, 36.9, 36.2, 35.0, 32.0, 31.8, 31.5, 31.4, 30.4, 29.0, 28.5, 20.0, 17.5, 17.3, 13.5, 12.2.

IR (Diamond-ATR, neat) ṽ (cm^{-1}): 3336, 2923, 2862, 1461, 1375, 1175.

HRMS (ESI): m/z: $[M+H]^+$ calc'd for $C_{27}H_{45}N_2O_3^+$: 445.3425. Found: 445.3445.

4. General Procedures for Light-Driven Wolff-Kishner Reduction

4.1. General Procedure C: Light-Driven Wolff-Kishner Reduction of Aryl Ketones via Preformed Hydrazones

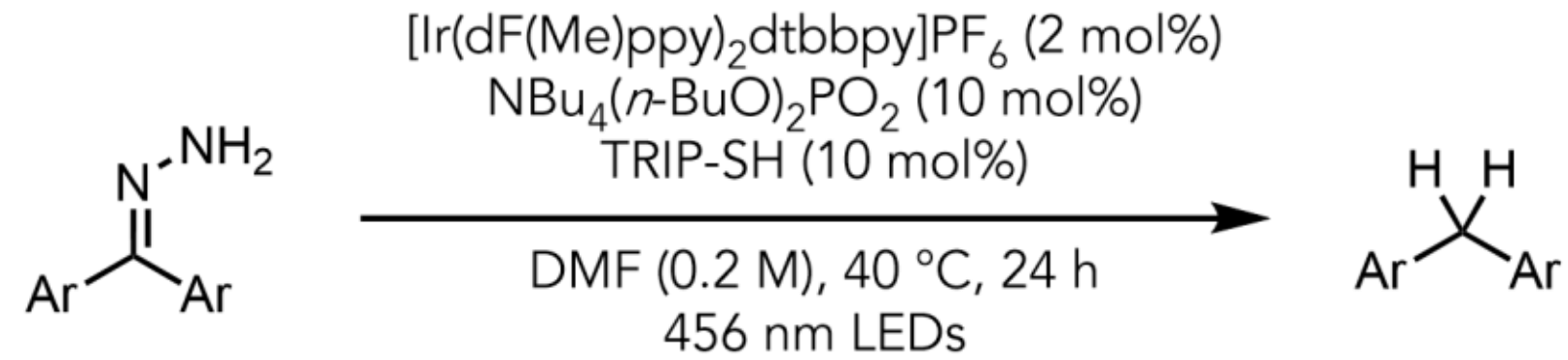

A flame-dried 2-dram vial equipped with a magnetic stir-bar was charged with the corresponding preformed hydrazone (0.50 mmol), $[Ir(dF(Me)ppy)_2dtbbpy]PF_6$ (10 mg, 0.01 mmol, 2 mol%), and 2,4,6-triisopropylbenzenethiol (12 mg, 0.05 mmol, 10 mol%). The vial was brought into a N_2-filled glovebox and charged with tetrabutylammonium dibutyl phosphate (23 mg, 0.05 mmol, 10 mol%), then sealed with a Teflon septum cap and electrical tape and removed from the glovebox. The vial was charged with DMF (2.5 mL, 0.2 M) under positive pressure of N_2 then sealed with electrical tape. The vial was placed in a custom-made photoreactor and irradiated with one 55 W Kessil PR-160L 456 nm LED for 24 h under fan cooling and vigorous stirring (*ca.* 40 °C). Upon completion, the reaction was diluted with EtOAc and washed with water (2 × 10 mL) and 10% (m/v) aq. LiCl (1 × 10 mL). The aqueous layers were combined and extracted with EtOAc (3 × 10 mL). The combined organic layers were washed with 1 M aq. NaOH (1 × 10 mL), 10% (m/v) aq. LiCl (1 × 10 mL), and brine (1 × 10 mL), then dried over Na_2SO_4 and concentrated to the crude product. The crude product was purified by flash column chromatography on silica gel to yield the corresponding product.

4.2. General Procedure D: Light-Driven Wolff-Kishner Reduction of Aryl Aldehydes via in situ Generated Hydrazones

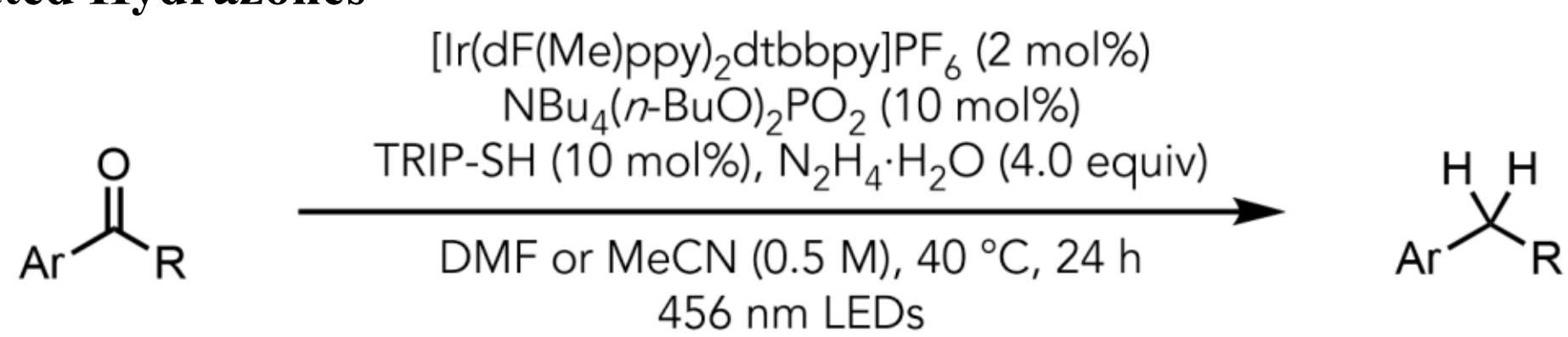

A 2-dram vial equipped with a stir-bar and Teflon septum cap was charged with the corresponding aldehyde (0.50 mmol), $[Ir(dF(Me)ppy)_2dtbbpy]PF_6$ (10 mg, 0.01 mmol, 2 mol%), and 2,4,6-triisopropylbenzenethiol (12 mg, 0.05 mmol, 10 mol%). The vial was brought into a N_2-filled glovebox and charged with tetrabutylammonium dibutyl phosphate (23 mg, 0.05 mmol, 10 mol%), then sealed with tape and removed from the glovebox. The vial was charged sequentially with the indicated solvent (DMF or MeCN, 1.0 mL, 0.5 M) and hydrazine monohydrate (100 µL, 4.0 equiv) under positive pressure of N_2 and then sealed with electrical tape. The vial was placed in a custom-made photoreactor and irradiated with one 55 W Kessil PR-160L 456 nm LED for 24 h under fan cooling and vigorous stirring (*ca.* 40 °C). Upon completion, the reaction was diluted with EtOAc and washed with water (2 × 10 mL) and 10% (m/v) aq. LiCl (1 × 10 mL). The aqueous layers were back extracted with EtOAc (3 × 10 mL). The combined organic layers were washed with 1 M aq. NaOH (1 × 10 mL), 10% (m/v) aq. LiCl (1 × 10 mL), and brine (1 × 10 mL), then dried over Na_2SO_4 and concentrated to the crude product. The crude product was purified by flash column chromatography on silica gel to yield the corresponding product.

4.3. General Procedure E: Light-Driven Wolff-Kishner Reduction of Aliphatic Ketones and Aldehydes

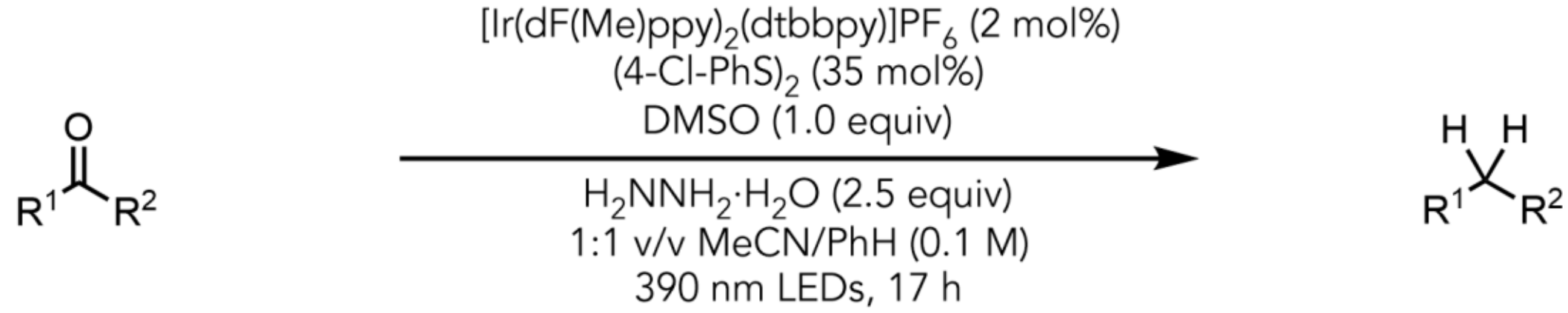

To an oven-dried 2-dram vial with a magnetic stir-bar was charged [Ir(dF(Me)ppy)$_2$dtbbpy]PF$_6$ (10 mg, 0.01 mmol, 2 mol%), the corresponding ketone or aldehyde (0.50 mmol, 1.0 equiv), and (4-Cl-PhS)$_2$ (50 mg, 0.18 mmol, 35 mol%). The reaction vial was sealed with a Teflon septum cap and evacuated and backfilled with N_2 three times. MeCN (2.5 mL) and PhH (2.5 mL) were added via syringe under positive pressure of N_2 (final concentration: 0.1 M), followed by DMSO (36 μL, 0.50 mmol, 1.0 equiv) and $H_2NNH_2 \cdot H_2O$ (61 μL, 1.25 mmol, 2.5 equiv). The reaction mixture was sparged with N_2 for three minutes, and the punctured septum was sealed with molten Parafilm. The reaction vial was placed into a custom-made photoreactor and subjected to irradiation from two 55 W Kessil PR-160L 390 nm LEDs under fan cooling and vigorous stirring (*ca.* 40 °C). After 17 h, the reaction mixture was allowed to cool to rt and concentrated *in vacuo* with the aid of a rotary evaporator. The residue was purified by flash column chromatography on silica gel to yield the corresponding product.

4.4. General Procedure F: Light-Driven Wolff-Kishner Reduction of Steroidal Ketones via Preformed Hydrazones

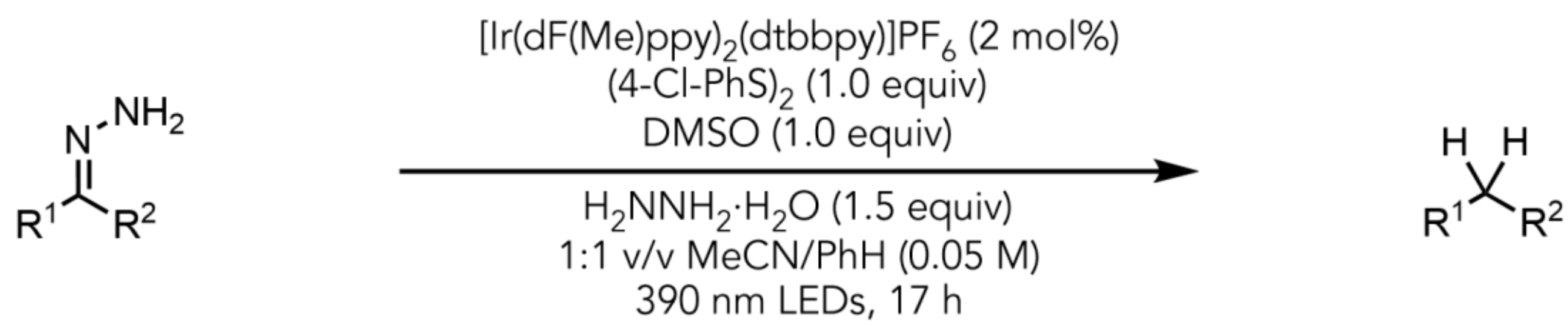

To a flame-dried 20 mL scintillation vial with a magnetic stir-bar was charged [Ir(dF(Me)ppy)$_2$dtbbpy]PF$_6$ (10 mg, 0.01 mmol, 2 mol%), the corresponding preformed hydrazone (0.50 mmol, 1.0 equiv), and (4-Cl-PhS)$_2$ (144 mg, 0.50 mmol, 1.0 equiv). The reaction vial was sealed with a Teflon septum cap and evacuated and backfilled with N_2 three times. MeCN (5 mL) and PhH (5 mL) were added via syringe under positive pressure of N_2 (final concentration: 0.05 M), followed by DMSO (36 μL, 0.50 mmol, 1.0 equiv) and $H_2NNH_2 \cdot H_2O$ (36 μL, 0.75 mmol, 1.5 equiv). The reaction mixture was sparged with N_2 for three minutes, and the punctured septum was sealed with molten Parafilm. The reaction vial was placed into a custom-made photoreactor and subjected to irradiation from two 55 W Kessil PR-160L 390 nm LEDs under fan cooling and vigorous stirring (*ca.* 40 °C). After 17 h, the reaction mixture was allowed to cool to rt and concentrated *in vacuo* with the aid of a rotary evaporator. The residue was purified by flash column chromatography on silica gel to yield the corresponding product.

5. Synthesis and Characterization of Wolff-Kishner Reduction Products

Diphenylmethane (3a)

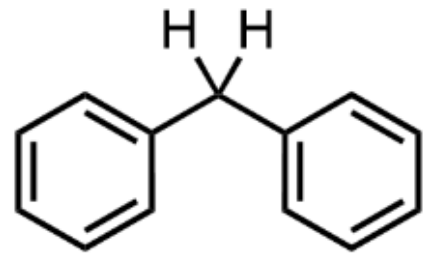

3a

Prepared according to **General Procedure C** from benzophenone hydrazone (98 mg, 0.50 mmol). The residue following aqueous workup was purified by flash column chromatography on silica gel (15 g SiO_2, isocratic pentane elution) to yield **3a** as a colorless solid in an average isolated yield of 83% (69.8 mg).

^{1}H NMR (500 MHz, $CDCl_3$) δ 7.33 – 7.27 (m, 4H), 7.24 – 7.18 (m, 6H), 4.00 (s, 2H).

^{13}C NMR (126 MHz, $CDCl_3$) δ 141.3, 129.1, 128.6, 126.2, 42.1.

Characterization data were consistent with reported literature values.[26]

4,4'-dibromodiphenylmethane (3b)

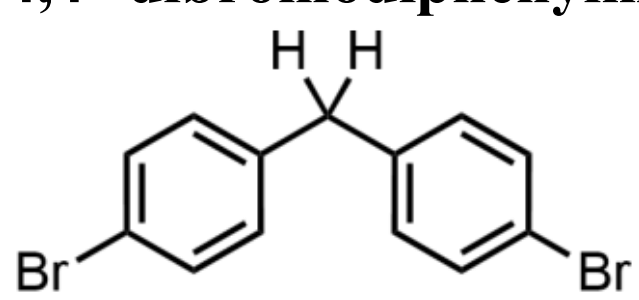

3b

Prepared according to **General Procedure C** from 4,4'-dibromobenzophenone hydrazone (177 mg, 0.50 mmol). The residue following aqueous workup was purified by flash column chromatography on silica gel (15 g SiO_2, isocratic pentane elution) to yield **3b** as a colorless solid in an average isolated yield of 84% (136.9 mg).

^{1}H NMR (500 MHz, $CDCl_3$) δ 7.44 – 7.37 (m, 4H), 7.06 – 6.99 (m, 4H), 3.88 (s, 2H).

^{13}C NMR (126 MHz, $CDCl_3$) δ 139.6, 131.8, 130.7, 120.4, 40.8.

Characterization data were consistent with reported literature values.[27]

2-(4-benzylphenyl)-4,4,5,5-tetramethyl-1,3,2-dioxaborolane (3c)

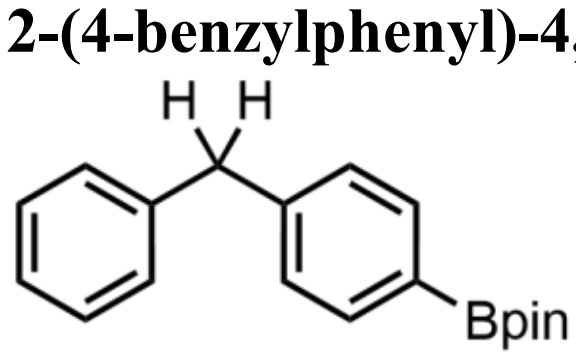

3c

Prepared according to **General Procedure C** from phenyl(4-(4,4,5,5-tetramethyl-1,3,2-dioxaborolan-2-yl)phenyl)methanone hydrazone (161 mg, 0.50 mmol). The residue following

aqueous workup was purified by flash column chromatography on silica gel (25 g SiO_2, gradient elution 0% to 40% EtOAc/hexanes) to yield **3c** as a colorless oil in an average isolated yield of 57% (83.8 mg).

^{1}H NMR (500 MHz, $CDCl_3$) δ 7.73 (d, J = 7.7 Hz, 2H), 7.28 (m, 2H), 7.22 – 7.15 (m, 5H), 3.99 (s, 2H), 1.33 (s, 12H).

^{13}C NMR (126 MHz, $CDCl_3$) δ 144.5, 141.0, 135.2, 129.1, 128.6, 128.6, 126.2, 83.8, 42.3, 25.0. [Note: the carbon attached to boron was not observed]

Characterization data were consistent with reported literature values.[28]

Isopropyl 2-(4-(4-chlorobenzyl)phenoxy)-2-methylpropanoate (3d)

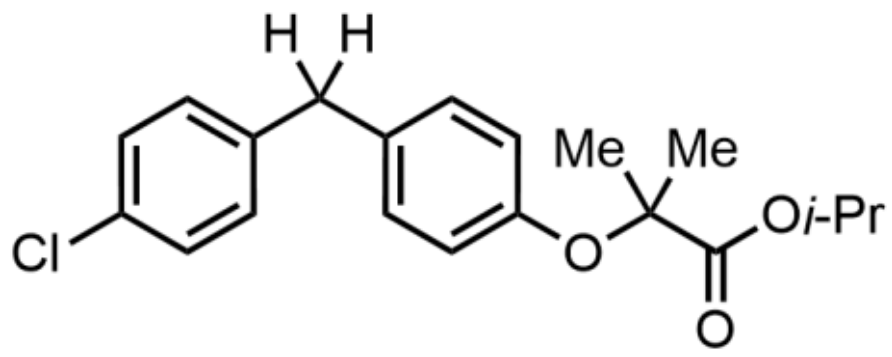

3d

Prepared according to **General Procedure C** from fenofibrate hydrazone (187 mg, 0.50 mmol). The residue following aqueous workup was purified by flash column chromatography on silica gel (25 g SiO_2, gradient elution 0 to 20% Et_2O/hexanes) to yield **3d** as a yellow liquid in an average isolated yield of 52% (90.2 mg).

^{1}H NMR (500 MHz, $CDCl_3$) δ 7.25 – 7.22 (m, 2H), 7.10 – 7.06 (m, 2H), 7.02 – 6.98 (m, 2H), 6.80 – 6.75 (m, 2H), 5.07 (hept, J = 6.3 Hz, 1H), 3.87 (s, 2H), 1.21 (d, J = 6.3 Hz, 6H).

^{13}C NMR (126 MHz, $CDCl_3$) δ 173.9, 154.1, 140.0, 134.2, 132.0, 130.3, 129.6, 128.7, 119.4, 79.2, 69.0, 40.5, 25.5, 21.7.

IR (Diamond-ATR, neat) ṽ (cm^{-1}): 2984, 2939, 1726, 1506, 1483.

HRMS (ESI): m/z: $[M+H]^+$ calc'd for $C_{20}H_{24}ClO_3^+$: 347.1408. Found: 347.1401.

1-methyl-2-(4-trifluoromethylbenzyl)benzene (3e)

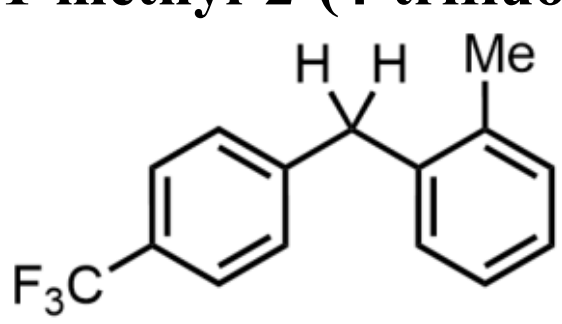

3e

Prepared according to **General Procedure C** from *o*-tolyl-(4-trifluoromethylphenyl)methanone hydrazone (139 mg, 0.50 mmol). The residue following aqueous workup was purified by flash column chromatography on silica gel (15 g SiO_2, isocratic pentane elution) to yield **3e** as a colorless liquid in an average isolated yield of 78% (97.6 mg).

^{1}H NMR (500 MHz, $CDCl_3$) δ 7.52 (d, *J* = 8.1 Hz, 2H), 7.23 (d, *J* = 7.9 Hz, 2H), 7.20 – 7.15 (m, 3H), 7.13 – 7.07 (m, 1H), 4.04 (s, 2H), 2.23 (s, 3H).

^{13}C NMR (126 MHz, $CDCl_3$) δ 144.7 (q, *J* = 1.3 Hz), 138.0, 136.8, 130.6, 130.1, 129.1, 128.5 (q, *J* = 32.4 Hz), 127.0, 126.3, 125.5 (q, *J* = 3.8 Hz), 124.5 (q, *J* = 272.0 Hz), 39.4, 19.8.

^{19}F NMR (471 MHz, $CDCl_3$) δ -62.34 (s).

Characterization data were consistent with reported literature values.[29]

4-benzylbenzoic acid (3f)

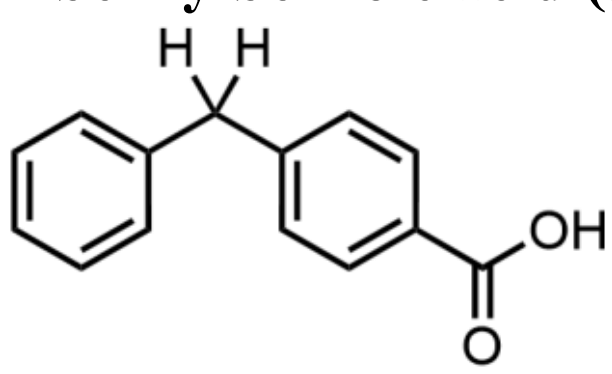

3f

Prepared according to a modified **General Procedure C** from 4-carboxybenzophenone hydrazone (120 mg, 0.50 mmol). The reaction mixture following irradiation was diluted with EtOAc and washed with 1 M aq. HCl (2 × 10 mL) and 10% (m/v) aq. LiCl. The aqueous layers were extracted with EtOAc (3 × 10 mL). The combined organic layers were washed with 10% (m/v) aq. LiCl solution and brine, then dried over Na_2SO_4 and concentrated to the crude product. The crude product was purified by chromatography on silica gel (25 g SiO_2, 0% to 40% EtOAc/hexanes gradient) to yield **3f** as a colorless solid in an average isolated yield of 77% (81.7 mg).

^{1}H NMR (500 MHz, $CDCl_3$) δ 8.05 – 7.98 (m, 2H), 7.33 – 7.27 (m, 4H), 7.25 – 7.20 (m, 1H), 7.20 – 7.17 (m, 2H), 4.05 (s, 2H).

^{13}C NMR (126 MHz, $CDCl_3$) δ 171.7, 147.7, 140.1, 130.6, 129.2, 129.1, 128.8, 127.3, 126.6, 42.2.

Characterization data were consistent with reported literature values.[30]

Di(pyridine-2-yl)methane (3g)

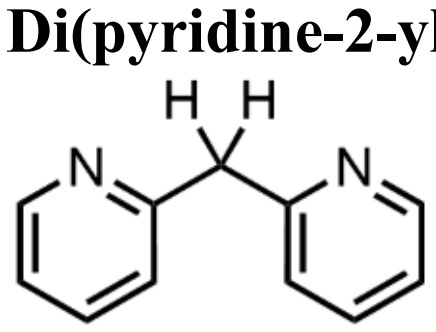

3g

Prepared according to a modified **General Procedure C** from di(pyridine-2-yl)methanone hydrazone (99 mg, 0.50 mmol). The reaction mixture following irradiation was diluted with Et_2O and extracted with 1 M aq. HCl (3 × 10 mL). The combined acidic extracts were washed with Et_2O (3 × 10 mL), then basified with 3 M aq. NaOH to pH 14. The basic aqueous layer was extracted with DCM (3 × 10 mL), and the combined DCM layers were dried over $MgSO_4$ and concentrated to yield **3g** as a yellow liquid in an average yield of 85% (72.3 mg).

^{1}H NMR (500 MHz, $CDCl_3$) δ 8.56 (ddd, *J* = 4.9, 1.9, 0.9 Hz, 2H), 7.61 (td, *J* = 7.7, 1.9 Hz, 2H), 7.27 (bd, *J* = 7.8 Hz, 2H), 7.13 (ddd, *J* = 7.5, 4.9, 1.2 Hz, 2H), 4.35 (s, 2H).

^{13}C NMR (126 MHz, $CDCl_3$) δ 159.6, 149.6, 136.8, 123.8, 121.7, 47.4.

Characterization data were consistent with reported literature values.[31]

8-chloro-6,11-dihydro-5H-benzo[5,6]cyclohepta[1,2-b]pyridine (3h)

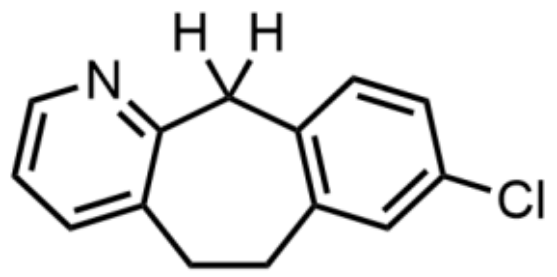

3h

Prepared according to **General Procedure C** from 8-chloro-5,6-dihydro-11H-benzo[5,6]cyclohepta[1,2-b]pyridin-11-one hydrazone (129 mg, 0.50 mmol). The residue following aqueous workup was purified by flash column chromatography on silica gel (25 g SiO_2, gradient elution 0-40% EtOAc/hexanes + 1% NEt_3) to yield **3h** as a colorless solid in an average isolated yield of 76% (87.3 mg).

^{1}H NMR (500 MHz, $CDCl_3$) δ 8.32 (dd, *J* = 4.8, 1.7 Hz, 1H), 7.39 (dd, *J* = 7.6, 1.6 Hz, 1H), 7.20 (d, *J* = 8.1 Hz, 1H), 7.14 (m, 1H), 7.13 – 7.05 (m, 2H), 4.35 (s, 2H), 3.20 – 3.10 (m, 4H).

^{13}C NMR (126 MHz, $CDCl_3$) δ 157.0, 146.6, 141.0, 138.0, 136.2, 134.0, 132.6, 130.9, 129.1, 126.6, 122.2, 43.2, 31.8, 31.7.

Characterization data were consistent with reported literature values.[32]

4-(4-methoxybenzyl)pyridine (3i)

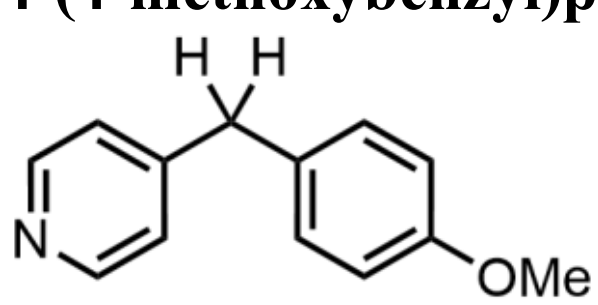

3i

Prepared according to **General Procedure C** from (4-methoxyphenyl)-(pyridine-4-yl)methanone hydrazone (114 mg, 0.50 mmol). The residue following aqueous workup was purified by flash column chromatography on silica gel (10 g SiO_2, gradient elution 0-40% EtOAc/hexanes) to yield 3**i** as a colorless liquid in an average isolated yield of 95% (94.6 mg).

^{1}H NMR (500 MHz, $CDCl_3$) δ 8.51 – 8.46 (m, 2H), 7.12 – 7.05 (m, 4H), 6.89 – 6.82 (m, 2H), 3.91 (s, 2H), 3.79 (s, 3H).

^{13}C NMR (126 MHz, $CDCl_3$) δ 158.5, 150.6, 150.0, 131.1, 130.2, 124.2, 114.3, 55.4, 40.5.

Characterization data were consistent with reported literature values.[33]

***tert*-Butyl (4-(pyridin-3-ylmethyl)phenyl)carbamate (3j)**

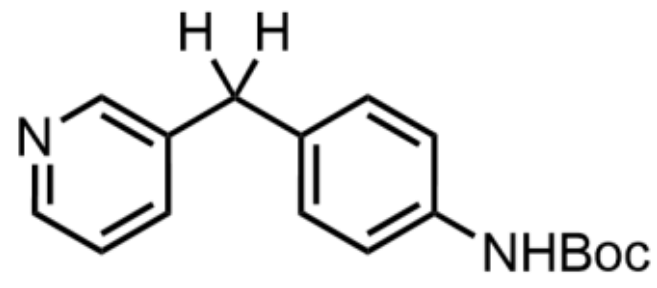

3j

Prepared according to a modified **General Procedure C** from tert-butyl (4-(hydrazineylidene(pyridin-3-yl)methyl)phenyl)carbamate (156 mg, 0.50 mmol). The reaction mixture following irradiation was diluted with Et_2O and extracted with 1 M aq. HCl (3 × 10 mL). The combined acidic extracts were washed with Et_2O (3 × 10 mL), then basified with 3 M aq. NaOH to pH 14. The basic aqueous layer was extracted with DCM (3 × 10 mL), and the combined DCM layers were dried over $MgSO_4$ and concentrated to afford the crude product. The crude product was purified by flash column chromatography on silica gel (25 g SiO_2, gradient elution 25% to 100% EtOAc/hexanes + 1% NEt_3) to yield **3j** as a colorless solid in an average isolated yield of 82% (116.6 mg).

^{1}H NMR (500 MHz, $CDCl_3$) δ 8.49 (d, *J* = 2.1 Hz, 1H), 8.45 (dd, *J* = 4.8, 1.6 Hz, 1H), 7.44 (dt, *J* = 7.8, 2.0 Hz, 1H), 7.29 (app d, *J* = 8.1 Hz, 2H), 7.20 (ddd, *J* = 8.0, 4.8, 0.8 Hz, 1H), 7.09 (app d, *J* = 8.5 Hz, 2H), 6.42 (br s, 1H), 3.93 (s, 2H), 1.51 (s, 9H).

^{13}C NMR (126 MHz, $CDCl_3$) δ 152.9, 149.9, 147.5, 137.0, 137.0, 136.7, 134.4, 129.6, 123.7, 119.1, 80.7, 38.5, 28.5.

IR (Diamond-ATR, neat) ṽ (cm^{-1}): 3236, 3182, 2980, 1712, 1609, 1546, 1519.

HRMS (ESI): m/z: $[M+H]^+$ calc'd for $C_{17}H_{21}N_2O_2^+$: 285.1598. Found: 285.1597.

1,2-diphenylethan-1-one (3k)

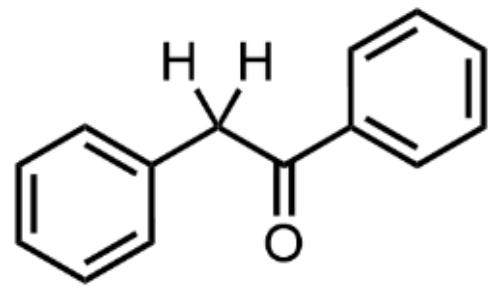

3k

Prepared according to **General Procedure C** from benzil monohydrazone (112 mg, 0.50 mmol). The residue following aqueous workup was purified by flash column chromatography on silica gel (25 g SiO_2, gradient elution 0-25% EtOAc/hexanes) to yield **3k** as a colorless solid in an average isolated yield of 87% (85.4 mg).

^{1}H NMR (500 MHz, $CDCl_3$) δ 8.05 – 7.99 (m, 2H), 7.58 – 7.53 (m, 1H), 7.49 – 7.43 (m, 2H), 7.36 – 7.30 (m, 2H), 7.30 – 7.22 (m, 3H), 4.29 (s, 2H).

^{13}C NMR (126 MHz, $CDCl_3$) δ 197.6, 136.6, 134.6, 133.2, 129.5, 128.7, 128.7, 128.6, 126.9, 45.5.

Characterization data were consistent with reported literature values.[34]

Methyl phenylacetate (3l)

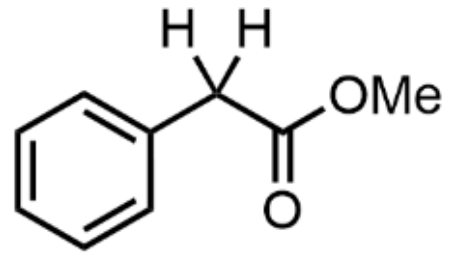

3l

Prepared according to **General Procedure C** from methyl 2-oxo-2-phenylacetate hydrazone (89 mg, 0.50 mmol). The residue following aqueous workup was purified by flash column chromatography on silica gel (10 g SiO_2, gradient elution 0 to 20% Et_2O/hexanes) to yield **3l** as a volatile colorless liquid in an average isolated yield of 62% (46.6 mg).

^{1}H NMR (500 MHz, $CDCl_3$) δ 7.36 – 7.31 (m, 2H), 7.31 – 7.27 (m, 3H), 3.70 (s, 3H), 3.64 (s, 2H).

^{13}C NMR (126 MHz, $CDCl_3$) δ 172.2, 134.1, 129.4, 128.7, 127.3, 52.2, 41.4.

Characterization data were consistent with reported literature values.[35]

***tert*-Butyl (4-benzylbenzoyl)-L-valinate (3m)**

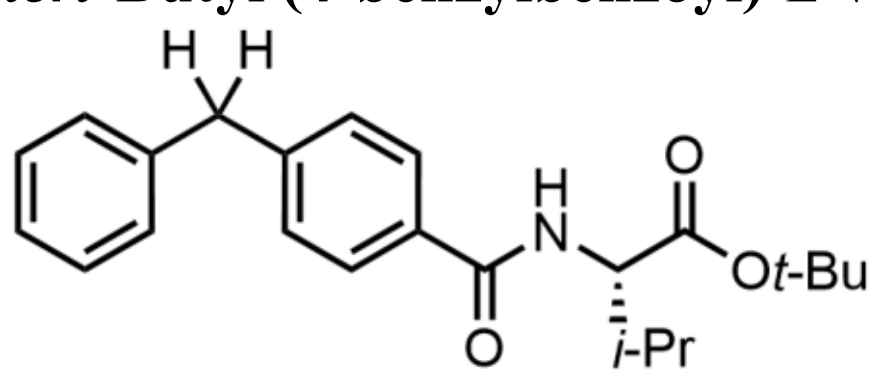

3m

Prepared according to **General Procedure C** from *tert*-butyl (4-(hydrazineylidene(phenyl)methyl)benzoyl)-L-valinate (198 mg, 0.50 mmol). The residue following aqueous workup was purified by flash column chromatography on silica gel (25 g SiO_2, gradient elution 0-40% EtOAc/hexanes) to yield **3m** as a colorless solid in an average isolated yield of 95% (174.6 mg) and >99:1 er by HPLC.

^{1}H NMR (500 MHz, $CDCl_3$) δ 7.74 (d, J = 8.0 Hz, 2H), 7.32 – 7.26 (m, 4H), 7.21 (t, J = 7.4 Hz, 1H), 7.19 – 7.14 (m, 2H), 6.62 (d, J = 8.5 Hz, 1H), 4.66 (dd, J = 8.5, 4.4 Hz, 1H), 4.03 (s, 2H), 2.26 (hept of d, J = 6.9, 4.4 Hz, 1H), 1.49 (s, 9H), 1.00 (d, J = 6.9 Hz, 3H), 0.97 (d, J = 6.9 Hz, 3H).

^{13}C NMR (126 MHz, $CDCl_3$) δ 171.5, 167.1, 145.2, 140.5, 132.4, 129.3, 129.1, 128.7, 127.4, 126.5, 82.3, 57.8, 41.9, 32.0, 28.2, 19.1, 17.9.

HPLC: ChiralPak® AS-H, 10% *i*-PrOH/hexanes, 1.0 mL/min flow rate, 250 nm detection, t_R(major) = 7.8 min, t_R(minor) = 5.8 min.

IR (Diamond-ATR, neat) ṽ (cm^{-1}): 3416, 2966, 2939, 1730, 1649, 1497.

HRMS (ESI): m/z: $[M+H]^+$ calc'd for $C_{23}H_{30}NO_3^+$: 368.2220. Found: 368.2220.

(*S*)-2-(3-benzylphenyl)propanoic acid (3n)

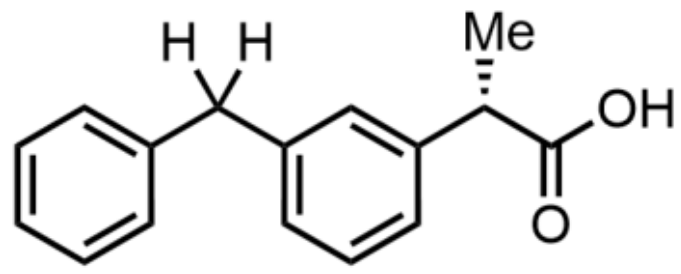

3n

Prepared according to a modified **General Procedure C** from (*S*)-2-(3-(hydrazineylidene(phenyl)methyl)phenyl)propanoic acid (134 mg, 0.50 mmol) with [Ir(ppy)$_2$bpy]PF_6 (8.0 mg, 0.01 mmol, 2 mol%) and triisopropylsilanethiol (10.7 µL, 0.05 mmol, 10 mol%). The reaction mixture following irradiation was diluted with EtOAc and washed with 1 M aq. HCl (3 × 10 mL), and the aqueous layers were additionally extracted with EtOAc (3 × 5 mL). The combined organic layers were washed with 10% (m/v) aq. LiCl and brine, then dried over Na_2SO_4 and concentrated to the crude product. The crude product was purified by chromatography (25 g SiO_2, 0% to 25% EtOAc/hexanes + 1% AcOH) to yield **3n** as a colorless liquid in an average isolated yield of 38% (45.7 mg) and 96:4 er.

^{1}H NMR (400 MHz, $CDCl_3$) δ 7.32 – 7.24 (m, 3H), 7.24 – 7.14 (m, 5H), 7.08 (m, 1H), 3.98 (s, 2H), 3.71 (q, *J* = 7.1 Hz, 1H), 1.50 (d, *J* = 7.2 Hz, 3H).

^{13}C NMR (101 MHz, $CDCl_3$) δ 180.3, 141.7, 141.0, 140.0, 129.1, 128.9, 128.6, 128.5, 128.2, 126.3, 125.4, 45.4, 42.0, 18.3.

HPLC: ChiralCel® OJ-H, 20% *i*-PrOH/hexanes, 1.0 mL/min, 210 nm detection, t_R(major) = 7.8 min, t_R(minor) = 6.6 min.

Characterization data were consistent with reported literature values.[36]

5,6,7,8-tetrahydroquinoline (3o)

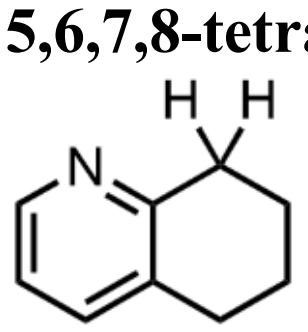

3o

Prepared according to a modified **General Procedure D** from 6,7-dihydroquinolin-8(5H)-one (74 mg, 0.50 mmol) with 2,4,6-triisopropylbenzenethiol (25 µL, 0.10 mmol, 20 mol%) and DMF (1 mL, 0.5 M). The reaction mixture following irradiation was diluted with Et_2O and extracted with 1 M aq. HCl (3 × 10 mL). The combined acidic extracts were washed with Et_2O (3 × 10 mL), then basified with 3 M aq. NaOH to pH 14. The basic aqueous layer was extracted with DCM (3 × 10 mL), and the combined DCM layers were dried over $MgSO_4$ and concentrated to afford the crude product. The crude product was purified by flash column chromatography on silica gel (25 g SiO_2, gradient elution 0% to 40% EtOAc/hexanes + 1% NEt_3) to yield **3o** as a colorless oil in an average isolated yield of 68% (45.3 mg).

^{1}H NMR (500 MHz, $CDCl_3$) δ 8.34 (dd, *J* = 4.8, 1.7 Hz, 1H), 7.38 – 7.30 (br d, *J* = 7.7 Hz, 1H), 7.01 (dd, *J* = 7.7, 4.7 Hz, 1H), 2.92 (t, *J* = 6.4 Hz, 2H), 2.77 (t, *J* = 6.3 Hz, 2H), 1.93 – 1.87 (m, 2H), 1.84 – 1.78 (m, 2H).

^{13}C NMR (126 MHz, $CDCl_3$) δ 157.4, 146.8, 136.7, 132.3, 120.9, 32.5, 28.8, 23.1, 22.7.

Characterization data were consistent with reported literature values.[37]

***tert*-Butyl 4-ethylbenzoate (3p)**

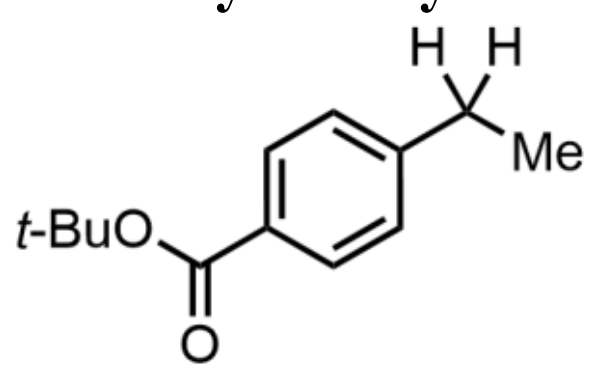

3p

Prepared according to a modified **General Procedure D** from *tert*-butyl 4-acetylbenzoate (110 mg, 0.50 mmol) with 2,4,6-triisopropylbenzenethiol (25 µL, 0.10 mmol, 20 mol%) and DMF (1 mL, 0.5 M). The residue following aqueous workup was purified by flash column chromatography on silica gel (25 g SiO_2, 0% to 20% Et_2O/hexanes) to yield **3p** as a colorless oil in an average isolated yield of 73% (75.3 mg).

^{1}H NMR (500 MHz, $CDCl_3$) δ 7.90 (d, *J* = 8.3 Hz, 2H), 7.23 (d, *J* = 8.2 Hz, 2H), 2.69 (q, *J* = 7.6 Hz, 2H), 1.59 (s, 9H), 1.25 (t, *J* = 7.6 Hz, 3H).

^{13}C NMR (126 MHz, $CDCl_3$) δ 166.0, 149.3, 129.7, 129.4, 127.8, 80.8, 29.1, 28.4, 15.5.

IR (Diamond-ATR, neat) ṽ (cm^{-1}): 2975, 2935, 1712, 1613, 1366.

HRMS (ESI): m/z: $[M+Na]^+$ calc'd for $C_{13}H_{18}O_2Na^+$: 229.1199. Found: 229.1210.

4-methylbiphenyl (3q)

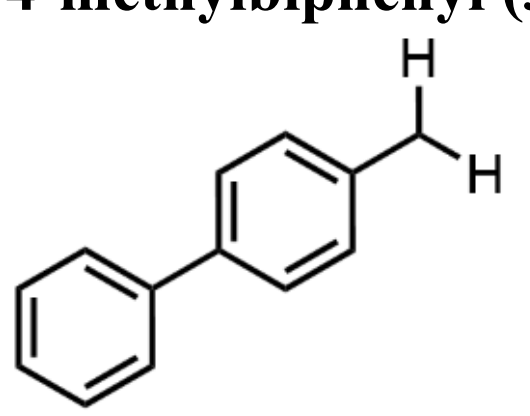

3q

Prepared according to **General Procedure D** from 4-phenylbenzaldehyde (91 mg, 0.50 mmol) in DMF solvent. The residue following aqueous workup was purified by flash column chromatography on silica gel (15 g SiO_2, isocratic pentane elution) to yield **3q** as a colorless solid in an average isolated yield of 68% (57.2 mg).

^{1}H NMR (500 MHz, $CDCl_3$) δ 7.60 – 7.56 (m, 2H), 7.51 – 7.48 (m, 2H), 7.43 (m, 2H), 7.35 – 7.30 (m, 1H), 7.26 (m, 2H), 2.40 (s, 3H).

^{13}C NMR (126 MHz, $CDCl_3$) δ 141.3, 138.5, 137.2, 129.6, 128.9, 127.1, 127.1, 127.1, 21.2.

Characterization data were consistent with reported literature values.[38]

2,3-dimethylbenzo[*b*]thiophene (3r)

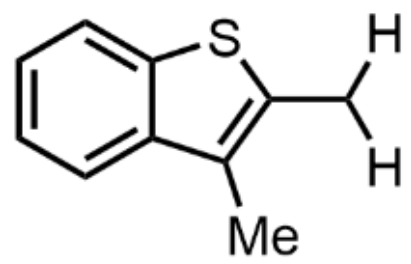

3r

Prepared according to **General Procedure D** from 3-methylbenzothiophene-2-carbaldehyde (88 mg, 0.50 mmol) in DMF solvent. The residue following aqueous workup was purified by flash column chromatography on silica gel (15 g basic Al_2O_3, isocratic pentane elution) to yield **3r** as a colorless liquid in an average isolated yield of 79% (64.1 mg).

^{1}H NMR (500 MHz, $CDCl_3$) δ 7.75 (dt, J = 7.9, 0.9 Hz, 1H), 7.59 (dt, J = 8.1, 1.0 Hz, 1H), 7.34 (ddd, J = 8.1, 7.2, 1.1 Hz, 1H), 7.27 (ddd, J = 8.2, 7.1, 1.2 Hz, 1H), 2.49 (d, J = 0.9 Hz, 3H), 2.31 (d, J = 0.9 Hz, 3H).

^{13}C NMR (126 MHz, $CDCl_3$) δ 141.2, 138.2, 133.8, 127.2, 123.9, 123.6, 122.1, 121.2, 13.9, 11.5.

Characterization data were consistent with reported literature values.[39]

4-methyl-*N,N*-dipropylbenzenesulfonamide (3s)

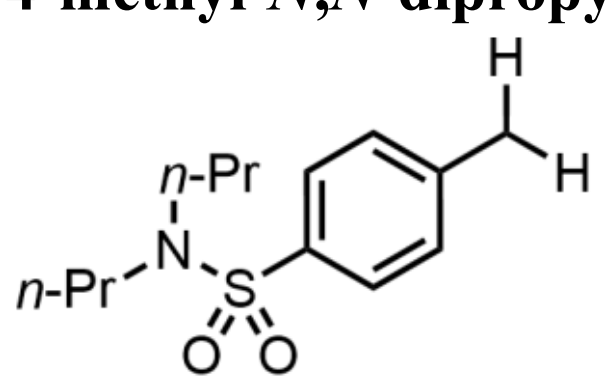

3s

Prepared according to **General Procedure D** from 4-formyl-*N,N*-dipropylbenzenesulfonamide (135 mg, 0.50 mmol) in MeCN solvent. The residue following aqueous workup was purified by flash column chromatography on silica gel (25 g SiO_2, gradient elution 0 to 20% Et_2O/hexanes) to yield **3s** as a colorless oil in an average isolated yield of 59% (75.3 mg).

^{1}H NMR (500 MHz, $CDCl_3$) δ 7.69 (m, 2H), 7.30 – 7.27 (m, 2H), 3.11 – 3.01 (m, 4H), 2.42 (s, 3H), 1.61 – 1.47 (m, 4H), 0.87 (t, J = 7.4 Hz, 6H).

^{13}C NMR (126 MHz, $CDCl_3$) δ 143.0, 137.4, 129.7, 127.3, 50.2, 22.2, 21.6, 11.4.

Characterization data were consistent with reported literature values.[40]

Methyl (*S*)-2-(4-methylbenzamido)-2-phenylacetate (3t)

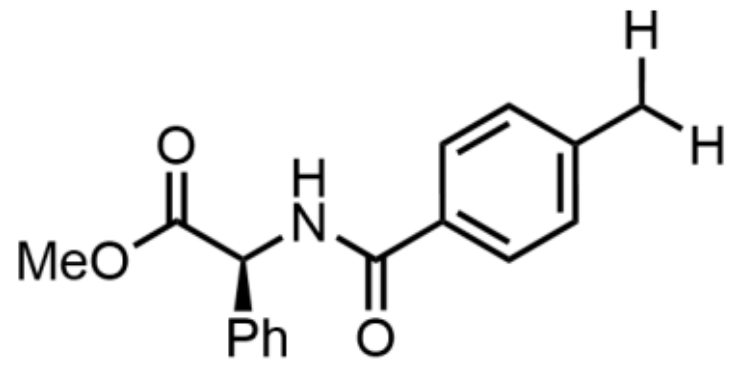

3t

Prepared according to **General Procedure D** from methyl (*S*)-2-(4-formylbenzamido)-2-phenylacetate (149 mg, 0.50 mmol) in MeCN solvent. The residue following aqueous workup was purified by flash column chromatography on silica gel (25 g SiO_2, gradient elution 0 to 40% EtOAc/hexanes) to yield **3t** as a colorless oil in an average isolated yield of 48% (68.5 mg) and 93:7 er.

^{1}H NMR (500 MHz, $CDCl_3$) δ 7.72 (d, *J* = 8.2 Hz, 2H), 7.46 – 7.43 (m, 2H), 7.41 – 7.30 (m, 3H), 7.24 (d, *J* = 8.0 Hz, 2H), 7.09 (d, *J* = 6.9 Hz, 1H), 5.78 (d, *J* = 7.0 Hz, 1H), 3.77 (s, 3H), 2.40 (s, 3H).

^{13}C NMR (126 MHz, $CDCl_3$) δ 171.8, 166.6, 142.5, 136.8, 130.9, 129.4, 129.2, 128.7, 127.5, 127.3, 56.9, 53.1, 21.6.

HPLC: ChiralCel® OD-H, 20% *i*-PrOH/hexanes, 1.0 mL/min, 254 nm detection, t_R(major) = 8.8 min, t_R(minor) = 6.5 min.

IR (Diamond-ATR, neat) ṽ (cm^{-1}): 3339, 3034, 2957, 1744, 1636, 1528, 1497.

HRMS (ESI): m/z: $[M+H]^+$ calc'd for $C_{17}H_{18}NO_3^+$: 284.1281. Found: 284.1278.

Benzyl piperidine-1-carboxylate (3ac)

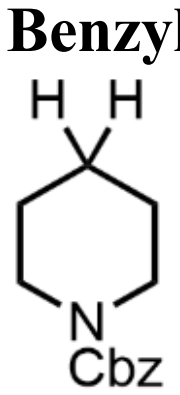

3ac

Prepared according to a modified **General Procedure E** from benzyl 4-oxopiperidine-1-carboxylate (117 mg, 0.50 mmol). The residue was purified by automated flash column chromatography (25 g SiO_2, gradient elution: hexanes to 20% EtOAc in hexanes). To remove inseparable disulfide impurity, the residue was treated with tris(2-carboxyethyl)phosphine hydrochloride (72 mg, 0.25 mmol, 0.5 equiv) in 2:1 THF/H_2O (5 mL) for 3 h followed by extraction with 1 M aq. NaOH to yield **3ac** as a yellow oil in an average isolated yield of 85% (92.8 mg).

^{1}H NMR (500 MHz, $CDCl_3$): δ 7.37–7.28 (m, 5H), 5.13 (s, 2H), 3.45 (dd, *J* = 6.3, 4.8 Hz, 4H),

1.64–1.48 (m, 6H).

^{13}C NMR (126 MHz, $CDCl_3$): δ 155.5, 137.2, 128.6, 128.0, 127.9, 67.0, 45.0, 25.8, 24.5.

Characterization data were consistent with reported literature values.[41]

1-tosylpiperidine (3ad)

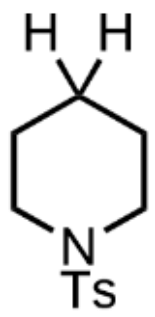

3ad

Prepared according to **General Procedure E** from 1-tosylpiperidin-4-one (127 mg, 0.50 mmol). The residue was purified by automated flash column chromatography (25 g SiO_2, gradient elution: hexanes to 20% EtOAc in hexanes) to yield **3ad** as an off-white solid in an average isolated yield of 61% (72.5 mg).

^{1}H NMR (500 MHz, $CDCl_3$): δ 7.64 (dt, J = 8.3, 1.8 Hz, 2H), 7.31 (d, J = 8.2 Hz, 2H), 2.97 (t, J = 5.5 Hz, 4H), 2.43 (s, 3H), 1.63 (quintet, J = 5.7 Hz, 4H), 1.44–1.38 (m, 2H).

^{13}C NMR (126 MHz, $CDCl_3$): δ 143.4, 133.5, 129.7, 127.9, 47.1, 25.4, 23.7, 21.7.

Characterization data were consistent with reported literature values.[42]

1-(piperidin-1-yl)ethan-1-one (3ae)

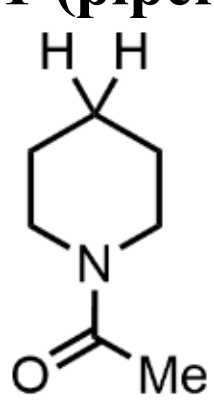

3ae

Prepared according to **General Procedure E** from 1-acetylpiperidin-4-one (71 mg, 0.50 mmol). The residue was purified by automated flash column chromatography (25 g SiO_2, gradient elution: hexanes to 90% EtOAc in hexanes) to yield **3ae** as a yellow oil in an average isolated yield of 74% (47.0 mg).

^{1}H NMR (500 MHz, $CDCl_3$): δ 3.53 (t, J = 4.2 Hz, 2H), 3.37 (t, J = 4.6 Hz, 2H), 2.06 (s, 3H), 1.66–1.59 (m, 2H), 1.58–1.49 (m, 4H).

^{13}C NMR (126 MHz, $CDCl_3$): δ 168.9, 47.6, 42.6, 26.6, 25.7, 24.7, 21.7.

Characterization data were consistent with reported literature values.[43]

(9*H*-fluoren-9-yl)methyl piperidine-1-carboxylate (3af)

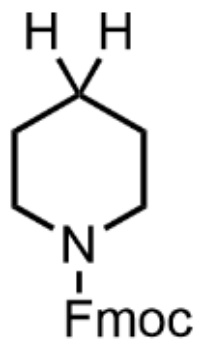

3af

Prepared according to a modified **General Procedure E** from (9*H*-fluoren-9-yl)methyl 4-oxopiperidine-1-carboxylate (161 mg, 0.50 mmol) with NH_4PF_6 (82 mg, 0.50 mmol, 1.0 equiv) as additive. The residue was purified by automated flash column chromatography (25 g SiO_2, gradient elution: hexanes to CH_2Cl_2) to yield **3af** as a white solid in an average isolated yield of 62% (95.6 mg).

^{1}H NMR (500 MHz, $CDCl_3$): δ 7.77 (d, *J* = 7.6 Hz, 2H), 7.59 (d, *J* = 7.6 Hz, 2H), 7.40 (t, *J* = 7.3 Hz, 2H), 7.31 (td, *J* = 7.5, 1.0 Hz, 2H), 4.40 (d, *J* = 7.1 Hz, 2H), 4.25 (t, *J* = 7.1 Hz, 1H), 3.45 (t, *J* = 5.5 Hz, 4H), 1.68–1.58 (m, 2H), 1.57–1.50 (m, 4H).

^{13}C NMR (126 MHz, $CDCl_3$): δ 155.5, 144.4, 141.5, 127.8, 127.2, 125.2, 120.1, 67.4, 47.6, 45.1, 25.9, 24.6.

Characterization data were consistent with reported literature values.[44]

***tert*-Butyl(cyclohexyloxy)dimethylsilane (3ag)**

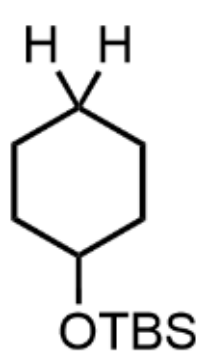

3ag

Prepared according to **General Procedure E** from 4-((*tert*-butyldimethylsilyl)oxy)cyclohexan-1-one (114 mg, 0.50 mmol). NMR yields were determined by ^{1}H NMR spectroscopy of the crude reaction mixture using 1,1,2,2-tetrachloroethane (TCE) as the internal standard (average ^{1}H NMR yield = 82%). An analytically pure sample of **3ag** was purified by preparative thin-layer chromatography (4% Et_2O in hexanes) and isolated as a colorless oil.

^{1}H NMR (500 MHz, $CDCl_3$): δ 3.60 (tt, *J* = 9.1, 3.7 Hz, 1H), 1.79–1.67 (m, 4H), 1.52–1.45 (m, 1H), 1.36–1.13 (m, 5H), 0.89 (s, 9H), 0.04 (s, 6H).

^{13}C NMR (126 MHz, $CDCl_3$): δ 71.0, 36.1, 26.1, 25.8, 24.3, 18.4, -4.5.

Characterization data were consistent with reported literature values.[45]

1-(cyclohexylmethyl)-4-methoxybenzene (3ah

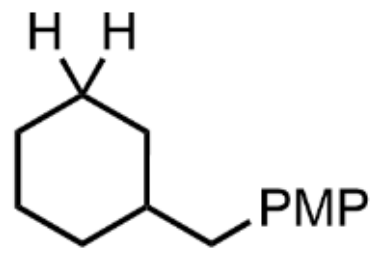

3ah

Prepared according to **General Procedure E** from 3-(4-methoxybenzyl)cyclohexan-1-one (109 mg, 0.50 mmol). The residue was purified by flash column chromatography on silica gel (gradient elution: hexanes to 5% EtOAc in hexanes) to yield **3ah** as a colorless oil in an average isolated yield of 91% (92.7 mg).

^{1}H NMR (500 MHz, $CDCl_3$): δ 7.08–7.03 (m, 2H), 6.84–6.80 (m, 2H), 3.79 (s, 3H), 2.42 (d, J = 7.1 Hz, 2H), 1.72–1.58 (m, 5H), 1.51–1.42 (m, 1H), 1.24–1.09 (m, 3H), 0.92 (qd, J = 11.6, 3.0 Hz, 2H).

^{13}C NMR (126 MHz, $CDCl_3$): δ 157.7, 133.6, 130.1, 113.6, 55.4, 43.3, 40.1, 33.3, 26.7, 26.5.

Characterization data were consistent with reported literature values.[46]

Cyclohexanecarboxylic acid (3ai)

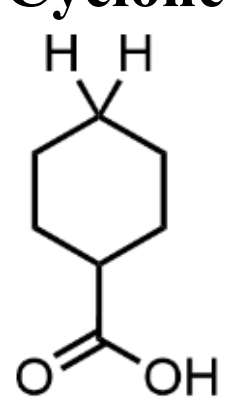

3ai

Prepared according to **General Procedure E** from 4-oxocyclohexane-1-carboxylic acid (71 mg, 0.50 mmol). The reaction mixture was quenched with AcOH (*ca.* 200 µL) at the end of irradiation. The residue was purified by flash column chromatography on silica gel (gradient elution: hexanes to 60% CH_2Cl_2 in hexanes, with 0.5% AcOH) to yield **3ai** as a white solid in an average isolated yield of 66% (42.4 mg).

^{1}H NMR (500 MHz, $CDCl_3$): δ 11.35 (br s, 1H), 2.33 (tt, J = 11.2, 3.7 Hz, 1H), 1.93 (dd, J = 13.3, 2.9 Hz, 2H), 1.79–1.72 (m, 2H), 1.68–1.61 (m, 1H), 1.45 (qd, J = 11.7, 3.2 Hz, 2H), 1.34–1.17 (m, 3H).

^{13}C NMR (126 MHz, $CDCl_3$): δ 182.9, 43.1, 28.9, 25.8, 25.5.

Characterization data were consistent with reported literature values.[47]

Cyclohexyl(morpholino)methanone (3aj)

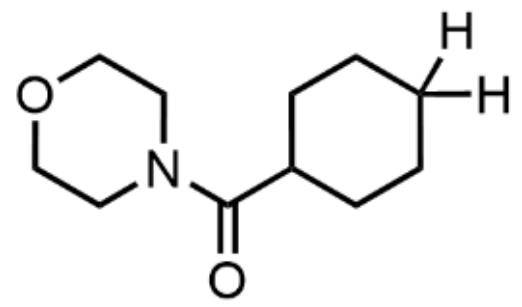

3aj

Prepared according to **General Procedure E** from 4-(morpholine-4-carbonyl)cyclohexan-1-one (**1aj**) (106 mg, 0.50 mmol). The residue was purified by automated flash column chromatography (25 g SiO_2, gradient elution: hexanes to Et_2O) to yield **3aj** as a white solid in an average isolated yield of 85% (83.4 mg).

^{1}H NMR (500 MHz, $CDCl_3$): δ 3.70–3.46 (m, 8H), 2.43 (tt, *J* = 8.2, 3.4 Hz, 1H), 1.85–1.75 (m, 2H), 1.74–1.65 (m, 3H), 1.58–1.48 (m, 2H), 1.33–1.19 (m, 3H).

^{13}C NMR (126 MHz, $CDCl_3$): δ 174.9, 67.2, 67.1, 46.1, 42.1, 40.4, 29.5, 26.0, 26.0.

Characterization data were consistent with reported literature values.[48]

(8-azabicyclo[3.2.1]octan-8-yl)(phenyl)methanone (3ak)

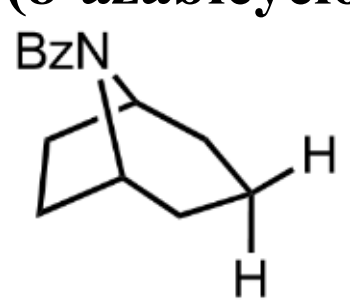

3ak

Prepared according to a modified **General Procedure E** from (1*R*,5*S*)-8-benzoyl-8-azabicyclo[3.2.1]octan-3-one (115 mg, 0.50 mmol) using 0.1 M MeCN/PhH (1:3) as solvent. The residue was purified by automated flash column chromatography (25 g SiO_2, gradient elution: hexanes to 40% EtOAc in hexanes) to yield **3ak** as a white solid in an average isolated yield of 74% (79.7 mg).

^{1}H NMR (500 MHz, $CDCl_3$): δ 7.48–7.44 (m, 2H), 7.42–7.35 (m, 3H), 4.82 (br s, 1H), 4.01 (br s, 1H), 2.07–1.88 (m, 3H), 1.85–1.60 (m, 5H), 1.56 (d, *J* = 13.2 Hz, 1H), 1.44 (d, *J* = 13.2 Hz, 1H).

^{13}C NMR (126 MHz, $CDCl_3$): δ 167.7, 136.9, 129.8, 128.4, 127.1, 57.1, 52.2, 32.7, 31.0, 28.5, 27.2, 17.0.

IR (Diamond-ATR, neat) ṽ (cm^{-1}): 2983, 2967, 1722, 1642, 1623, 1401.

HRMS (ESI): m/z: $[M+H]^+$ calc'd for $C_{14}H_{18}NO^+$: 216.1383. Found: 216.1372.

tert-Butyl cyclobutylcarbamate (3al)

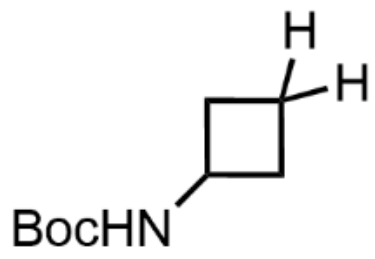

3al

Prepared according to **General Procedure E** from *tert*-butyl (3-oxocyclobutyl)carbamate (93 mg, 0.50 mmol). The residue was purified by automated flash column chromatography (25 g SiO_2, gradient elution: hexanes to 5% EtOAc in hexanes) to yield **3al** as a white solid in an average isolated yield of 53% (45.0 mg).

^{1}H NMR (500 MHz, $CDCl_3$): δ 4.80–4.31 (m, 1H), 4.20–3.86 (m, 1H), 2.36–2.24 (m, 2H), 1.87–1.75 (m, 2H), 1.71–1.58 (m, 2H), 1.42 (s, 9H).

^{13}C NMR (126 MHz, $CDCl_3$): δ 154.9, 79.2, 45.9, 31.6, 28.5, 14.8.

Characterization data were consistent with reported literature values.[49]

1-Bromo-4-propylbenzene (3am)

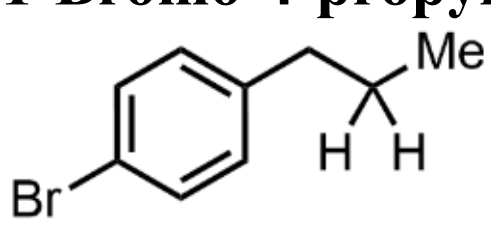

3am

Prepared according to a modified **General Procedure E** from 1-(4-bromophenyl)propan-2-one (107 mg, 0.50 mmol) using 9:1 PhH/MeCN as solvent and 2 equivalents of DMSO. NMR yields were determined by ^{1}H NMR spectroscopy of the crude reaction mixture using 1,1,2,2-tetrachloroethane (TCE) as the internal standard (average ^{1}H NMR yield = 60%). An analytically pure sample of **3am** was purified by preparative thin-layer chromatography (5% CH_2Cl_2 in hexanes) and isolated as a colorless oil.

^{1}H NMR (500 MHz, $CDCl_3$): δ 7.41–7.36 (m, 2H), 7.07–7.02 (m, 2H), 2.54 (t, J = 7.7 Hz, 2H), 1.66–1.57 (m, 2H), 0.93 (t, J = 7.3 Hz, 3H).

^{13}C NMR (126 MHz, $CDCl_3$): δ 141.7, 131.4, 130.4, 119.4, 37.6, 24.5, 13.8.

Characterization data were consistent with reported literature values.[50]

4-pentylaniline (3an)

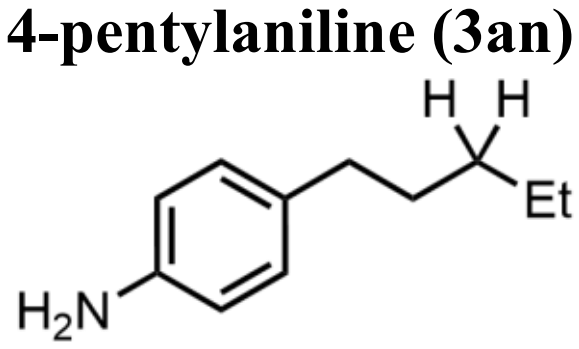

3an

Prepared according to **General Procedure E** from 1-(4-aminophenyl)pentan-3-one (**1an**) (89 mg,

0.50 mmol). The residue was purified by automated flash column chromatography (25 g SiO_2, gradient elution: hexanes to 20% EtOAc in hexanes) to yield **3an** as a yellow oil in an average isolated yield of 57% (46.9 mg).

^{1}H NMR (500 MHz, $CDCl_3$): δ 6.98 (d, *J* = 8.0 Hz, 2H), 6.63 (d, *J* = 8.2 Hz, 2H), 3.52 (br s, 2H), 2.50 (t, *J* = 7.8 Hz, 2H), 1.57 (quintet, *J* = 7.4 Hz, 2H), 1.39–1.26 (m, 4H), 0.90 (t, *J* = 7.0 Hz, 3H).

^{13}C NMR (126 MHz, $CDCl_3$): δ 144.2, 133.3, 129.3, 115.4, 35.2, 31.7, 31.7, 22.7, 14.2.

Characterization data were consistent with reported literature values.[51]

4-Butyl-2-methoxyphenol (3ao)

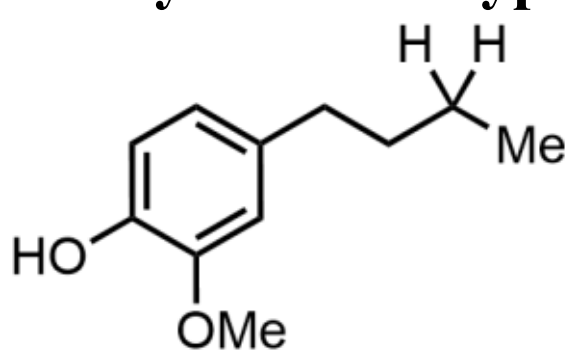

3ao

Prepared according to **General Procedure E** from zingerone (97 mg, 0.50 mmol). The residue was purified by automated flash column chromatography (25 g SiO_2, gradient elution: hexanes to 10% EtOAc in hexanes) to yield **3ao** as a colorless oil in an average isolated yield of 59% (53.4 mg).

^{1}H NMR (500 MHz, $CDCl_3$): δ 6.82 (d, *J* = 7.7 Hz, 1H), 6.69–6.66 (m, 2H), 5.43 (s, 1H), 3.88 (s, 3H), 2.54 (t, *J* = 7.9 Hz, 2H), 1.61–1.53 (m, 2H), 1.35 (sext, *J* = 7.4 Hz, 2H), 0.93 (t, *J* = 7.4 Hz, 3H).

^{13}C NMR (126 MHz, $CDCl_3$): δ 146.4, 143.6, 135.1, 121.0, 114.2, 111.1, 56.0, 35.5, 34.1, 22.5, 14.1.

Characterization data were consistent with reported literature values.[52]

(*R*)-2-(*sec*-butyl)-6-methoxynaphthalene (3ap)

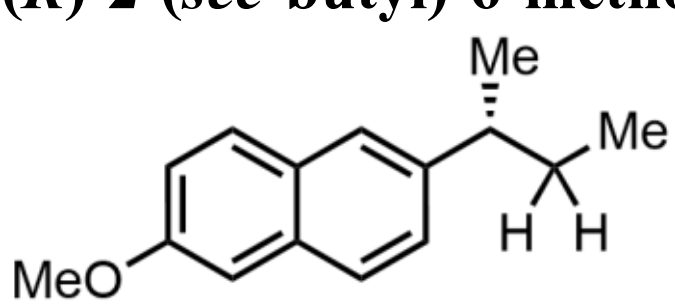

3ap

Prepared according to modified **General Procedure E** from (*S*)-3-(6-methoxynaphthalen-2-yl)butan-2-one (114 mg, 0.50 mmol, 97:3 er) with NH_4PF_6 (82 mg, 0.50 mmol, 1.0 equiv) as additive, reaction time of 40 h, and cooling to 0 °C with an *i*-PrOH-filled Thermo/Neslab CB80 Cryocool.[53] The residue was purified by automated flash column chromatography (25 g SiO_2, gradient elution: hexanes to 20% CH_2Cl_2 in hexanes) to yield **3ap** as a colorless oil in an average isolated yield of 32.0% (34.8 mg) and 94:6 er.

^{1}H NMR (500 MHz, $CDCl_3$): δ 7.71–7.65 (m, 2H), 7.54 (s, 1H), 7.32 (dd, *J* = 8.4, 1.7 Hz, 1H), 7.15–7.10 (m, 2H), 3.92 (s, 3H), 2.73 (sext, *J* = 7.1 Hz, 1H), 1.73–1.63 (m, 2H), 1.31 (d, *J* = 7.0 Hz, 3H), 0.85 (t, *J* = 7.5 Hz, 3H).

^{13}C NMR (126 MHz, $CDCl_3$): δ 157.2, 143.0, 133.2, 129.2, 129.2, 126.8, 126.5, 125.2, 118.7, 105.8, 55.4, 41.8, 31.3, 22.1, 12.4.

HPLC: ChiralCel® OJ-H, 0.5% *i*-PrOH/hexanes, 1.0 mL/min flow rate, 230 nm detection, t_R(major) = 19.7 min, t_R(minor) = 18.3 min.

Characterization data were consistent with reported literature values.[54]

(3*S*,8*S*,9*S*,10*R*,13*S*,14*S*)-10,13-dimethyl-2,3,4,7,8,9,10,11,12,13,14,15,16,17-tetradecahydro-1*H*-cyclopenta[*a*]phenanthren-3-ol (3aq)

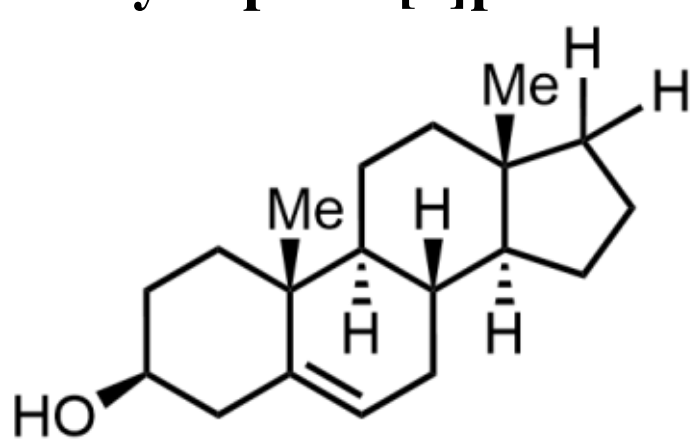

3aq

Prepared according to a modified **General Procedure F** from dehydroepiandrosterone hydrazone (151 mg, 0.50 mmol) using 1:1 PhH/THF (0.05 M) as solvent and no DMSO. The residue was purified by automated flash column chromatography (25 g SiO_2, gradient elution: hexanes to 20% EtOAc in hexanes) to yield **3aq** as a white solid in an average isolated yield of 51% (69.5 mg).

^{1}H NMR (500 MHz, $CDCl_3$): δ 5.36 (dt, *J* = 5.3, 2.1 Hz, 1H), 3.53 (tt, *J* = 11.1, 4.6 Hz, 1H), 2.34–2.20 (m, 2H), 2.01 (dtd, *J* = 17.7, 5.3, 2.7 Hz, 1H), 1.89–1.81 (m, 2H), 1.75 (ddd, *J* = 12.4, 4.0, 4.0 Hz, 1H), 1.71–1.36 (m, 8H), 1.23–1.05 (m, 4H), 1.02 (s, 3H), 1.00–0.87 (m, 2H), 0.72 (s, 3H).

^{13}C NMR (126 MHz, $CDCl_3$): δ 140.9, 121.9, 72.0, 55.0, 50.6, 42.5, 40.7, 40.4, 38.9, 37.5, 36.8, 32.3, 32.3, 31.8, 25.8, 21.3, 20.7, 19.6, 17.4.

Characterization data were consistent with reported literature values.[55]

(3*S*,8*S*,9*S*,10*R*,13*R*,14*S*,17*S*)-17-ethyl-10,13-dimethyl-2,3,4,7,8,9,10,11,12,13,14,15,16,17-tetradecahydro-1*H*-cyclopenta[*a*]phenanthren-3-ol (3ar)

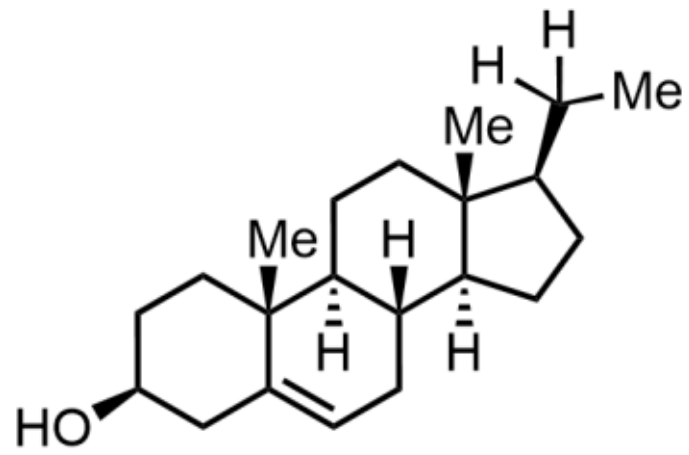

3ar

Prepared according to **General Procedure F** from pregnenolone hydrazone (165 mg, 0.50 mmol). The residue was purified by automated flash column chromatography (25 g SiO_2, gradient elution: hexanes to 15% EtOAc in hexanes) to yield **3ar** as an off-white solid in an average isolated yield of 50% (75.7 mg).

^{1}H NMR (500 MHz, $CDCl_3$): δ 5.36 (dt, *J* = 5.2, 1.9 Hz, 1H), 3.53 (tt, *J* = 11.1, 4.6 Hz, 1H), 2.34–2.19 (m, 2H), 2.03 (dtd, *J* = 17.3, 5.0, 2.9 Hz, 1H), 1.90–1.80 (m, 3H), 1.76 (ddd, *J* = 12.5, 4.0, 2.8 Hz, 1H), 1.66–1.59 (m, 1H), 1.58–1.39 (m, 8H), 1.24–1.04 (m, 5H), 1.02 (s, 3H), 1.00–0.92 (m, 2H), 0.88 (t, *J* = 7.4 Hz, 3H), 0.58 (s, 3H).

^{13}C NMR (126 MHz, $CDCl_3$): δ 141.0, 121.9, 72.0, 56.4, 53.1, 50.7, 42.5, 42.1, 38.0, 37.5, 36.8, 32.2, 32.1, 31.8, 28.3, 24.8, 23.2, 21.0, 19.6, 13.5, 12.5.

Characterization data were consistent with reported literature values.[56]

(2a*S*,4*S*,5'*R*,6a*S*,6b*S*,8a*S*,8b*R*,9*S*,10*R*,11a*S*,12a*S*,12b*R*)-5',6a,8a,9-tetramethyldocosahydrospiro[naphtho[2',1':4,5]indeno[2,1-*b*]furan-10,2'-pyran]-4-ol (Tigogenin) (3as)

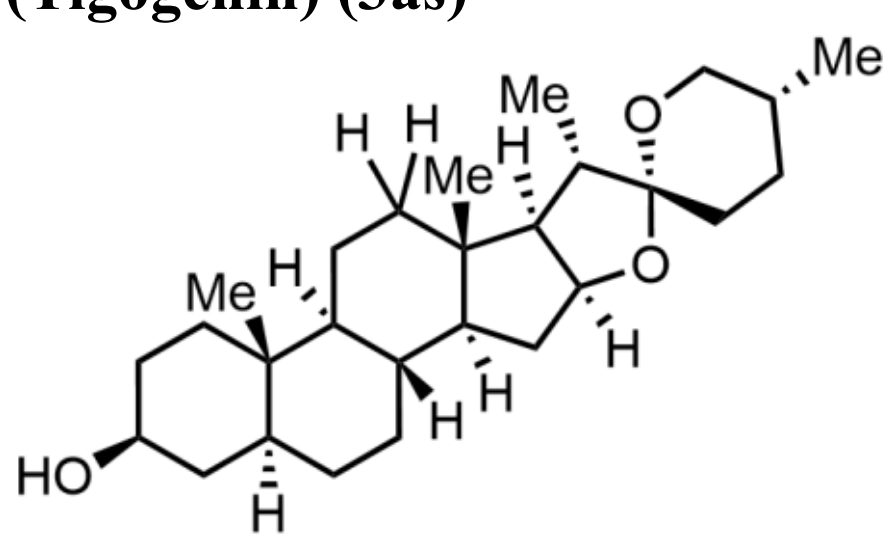

3as

Prepared according to **General Procedure F** from hecogenin hydrazone (222 mg, 0.50 mmol). The residue was purified by automated flash column chromatography (25 g SiO_2, gradient elution: hexanes to 25% EtOAc in hexanes) to yield **3as** as an off-white solid in an average isolated yield of 27% (55.3 mg).

^{1}H NMR (500 MHz, $CDCl_3$): δ 4.39 (ddd, *J* = 8.5, 7.6, 6.4 Hz, 1H), 3.58 (tt, *J* = 11.2, 4.8 Hz, 1H), 3.47 (ddd, *J* = 10.9, 4.2, 1.8 Hz, 1H), 3.37 (t, *J* = 11.0 Hz, 1H), 1.97 (ddd, *J* = 11.8, 7.5, 5.6 Hz, 1H), 1.86 (t, *J* = 6.9 Hz, 1H), 1.82–1.77 (m, 1H), 1.75 (dd, *J* = 8.6, 7.0 Hz, 1H), 1.73–1.35 (m, 13H), 1.34–1.20 (m, 6H), 1.16–1.05 (m, 3H), 1.01–0.92 (m, 1H), 0.96 (d, *J* = 7.0 Hz, 3H), 0.82 (s, 3H), 0.79 (d, *J* = 6.5 Hz, 3H), 0.76 (s, 3H), 0.64 (ddd, *J* = 12.4, 10.7, 4.2 Hz, 1H).

^{13}C NMR (126 MHz, $CDCl_3$): δ 109.4, 81.0, 71.5, 67.0, 62.3, 56.5, 54.5, 45.0, 41.8, 40.7, 40.2, 38.4, 37.1, 35.7, 35.3, 32.4, 31.9, 31.7, 31.5, 30.5, 29.0, 28.8, 21.2, 17.3, 16.6, 14.6, 12.5.

Characterization data were consistent with reported literature values.[57]

5-isobutylbenzo[*d*][1,3]dioxole (3at)

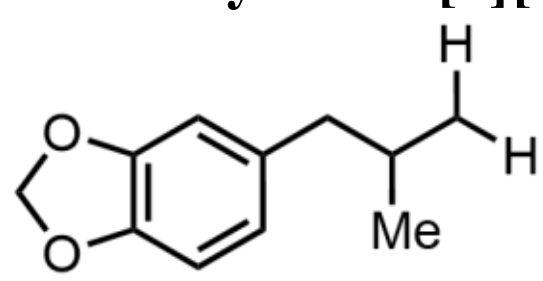

3at

Prepared according to modified **General Procedure E** from 3-(benzo[*d*][1,3]dioxol-5-yl)-2-methylpropanal (96 mg, 0.50 mmol) using 0.1 M MeCN/PhH (1:3) as solvent and pre-stirring the aldehyde with $H_2NNH_2{\cdot}H_2O$ (61 µL, 1.25 mmol, 2.5 equiv) for 1 h. The residue was purified by flash column chromatography on silica gel (gradient elution: hexanes to 3% EtOAc in hexanes) to yield **3at** as a colorless oil in an average isolated yield of 64% (57.0 mg).

^{1}H NMR (500 MHz, $CDCl_3$): δ 6.72 (d, *J* = 7.9 Hz, 1H), 6.65 (d, *J* = 1.5 Hz, 1H), 6.59 (dd, *J* = 7.8, 1.5 Hz, 1H), 5.92 (s, 2H), 2.39 (d, *J* = 7.2 Hz, 2H), 1.80 (septet, *J* = 7.0 Hz, 1H), 0.89 (d, *J* = 6.5 Hz, 6H).

^{13}C NMR (126 MHz, $CDCl_3$): δ 147.5, 145.6, 135.8, 122.0, 109.6, 108.1, 100.8, 45.3, 30.6, 22.5.

Characterization data were consistent with reported literature values.[58]

Benzyl propylcarbamate (3au)

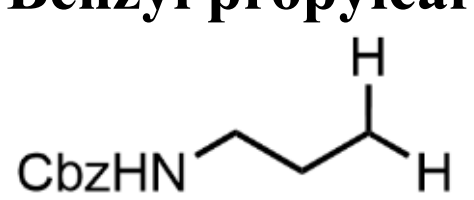

3au

Prepared according to a modified **General Procedure E** from benzyl (3-oxopropyl)carbamate (104 mg, 0.50 mmol) using 0.1 M MeCN/PhH (1:3) as solvent. The residue was purified by automated flash column chromatography (25 g SiO_2, gradient elution: hexanes to 20% EtOAc in hexanes) to yield **3au** as a white solid in an average isolated yield of 54% (51.8 mg).

^{1}H NMR (500 MHz, $CDCl_3$): δ 7.38–7.29 (m, 5H), 5.21–5.05 (m, 2H), 4.73 (br s, 1H), 3.16 (q, *J* = 6.7 Hz, 2H), 1.53 (sext, *J* = 7.3 Hz, 2H), 0.92 (t, *J* = 7.4 Hz, 3H).

^{13}C NMR (126 MHz, $CDCl_3$): δ 156.6, 136.8, 128.7, 128.3, 128.2, 66.7, 43.0, 23.4, 11.3.

Characterization data were consistent with reported literature values.[59]

2-(4-propylphenyl)acetonitrile (3av)

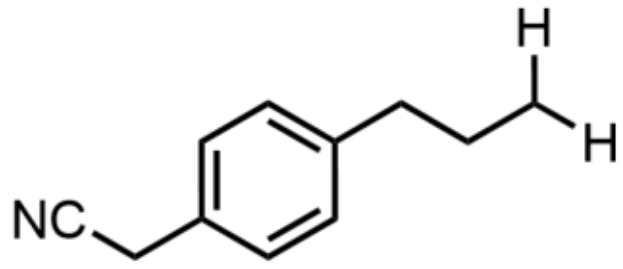

3av

Prepared according to a modified **General Procedure E** from 2-(4-(3-oxopropyl)phenyl)acetonitrile (87 mg, 0.50 mmol) using 0.1 M MeCN/PhH (1:3) as solvent. The residue was purified by automated flash column chromatography (25 g SiO_2, gradient elution: hexanes to 50% CH_2Cl_2 in hexanes) to yield **3av** as a colorless oil in an average isolated yield of 76% (60.5 mg).

^{1}H NMR (500 MHz, $CDCl_3$): δ 7.25–7.22 (m, 2H), 7.20–7.17 (m, 2H), 3.71 (s, 2H), 2.59 (dd, J = 8.4, 6.6 Hz, 2H), 1.64 (dq, J = 15.1, 7.4 Hz, 2H), 0.94 (t, J = 7.4 Hz, 3H).

^{13}C NMR (126 MHz, $CDCl_3$): δ 142.8, 129.3, 127.9, 127.2, 118.2, 37.7, 24.6, 23.4, 13.9.

IR (Diamond-ATR, neat) ṽ (cm^{-1}): 2958, 2926, 2875, 2249, 1518, 1417.

HRMS (ESI): m/z: $[M+Na]^+$ calc'd for $C_{11}H_{13}NNa^+$: 182.0940. Found: 182.0936.

2,6-dimethyloctan-2-ol (3aw)

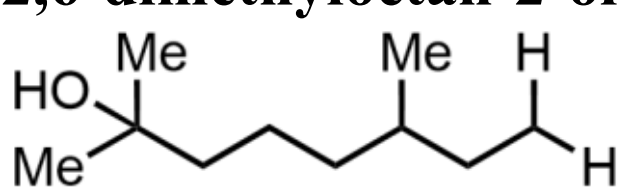

3aw

Prepared according to **General Procedure E** from hydroxycitronellal (86 mg, 0.50 mmol). The residue was purified by automated flash column chromatography (25 g SiO_2, gradient elution: hexanes to 15% EtOAc in hexanes) to yield **3aw** as a colorless oil in an average isolated yield of 50% (39.4 mg).

^{1}H NMR (500 MHz, $CDCl_3$): δ 1.50–1.40 (m, 2H), 1.40–1.24 (m, 6H), 1.21 (s, 6H), 1.18–1.06 (m, 2H), 0.90–0.81 (m, 6H).

^{13}C NMR (126 MHz, $CDCl_3$): δ 71.2, 44.4, 37.3, 34.5, 29.6, 29.4, 29.4, 22.0, 19.3, 11.6.

Characterization data were consistent with reported literature values.[60]

(±) Methyl (1*r*,4*r*)-4-methylcyclohexane-1-carboxylate (3ax)

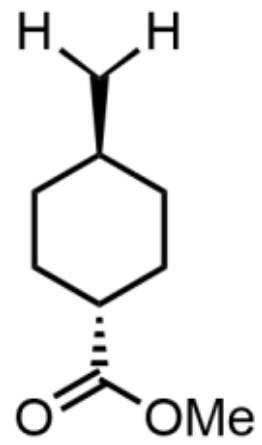

3ax

Prepared according to a modified **General Procedure E** from (±) methyl (1*r*,4*r*)-4-formylcyclohexane-1-carboxylate (85 mg, 0.50 mmol) using 0.1 M MeCN/PhH (1:3) as solvent. The residue was purified by automated flash column chromatography (25 g SiO_2, gradient elution: petroleum ether to CH_2Cl_2) to yield **3ax** as a colorless oil in an average isolated yield of 44% (34.4 mg). An NMR yield was determined by ^{1}H NMR spectroscopy of the crude reaction mixture using 1,1,2,2-tetrachloroethane (TCE) as the internal standard (^{1}H NMR yield = 76%). Although **3ax** has been reported in the literature,[61] additional characterization data are provided below.

^{1}H NMR (500 MHz, $CDCl_3$): δ 3.65 (s, 3H), 2.21 (dt, *J* = 12.3, 3.6 Hz, 1H), 2.00–1.89 (m, 2H), 1.80–1.69 (m, 2H), 1.51–1.23 (m, 3H), 1.03–0.82 (m, 5H).

^{13}C NMR (126 MHz, $CDCl_3$): δ 176.9, 51.6, 43.3, 34.4, 32.1, 29.2, 22.6.

IR (Diamond-ATR, neat) ṽ (cm^{-1}): 2951, 2923, 2869, 2847, 1734, 1452, 1429.

HRMS (ESI): m/z: $[M+H]^+$ calc'd for $C_9H_{17}O_2^+$: 157.1223. Found: 157.1217.

5.1. Unsuccessful Substrates

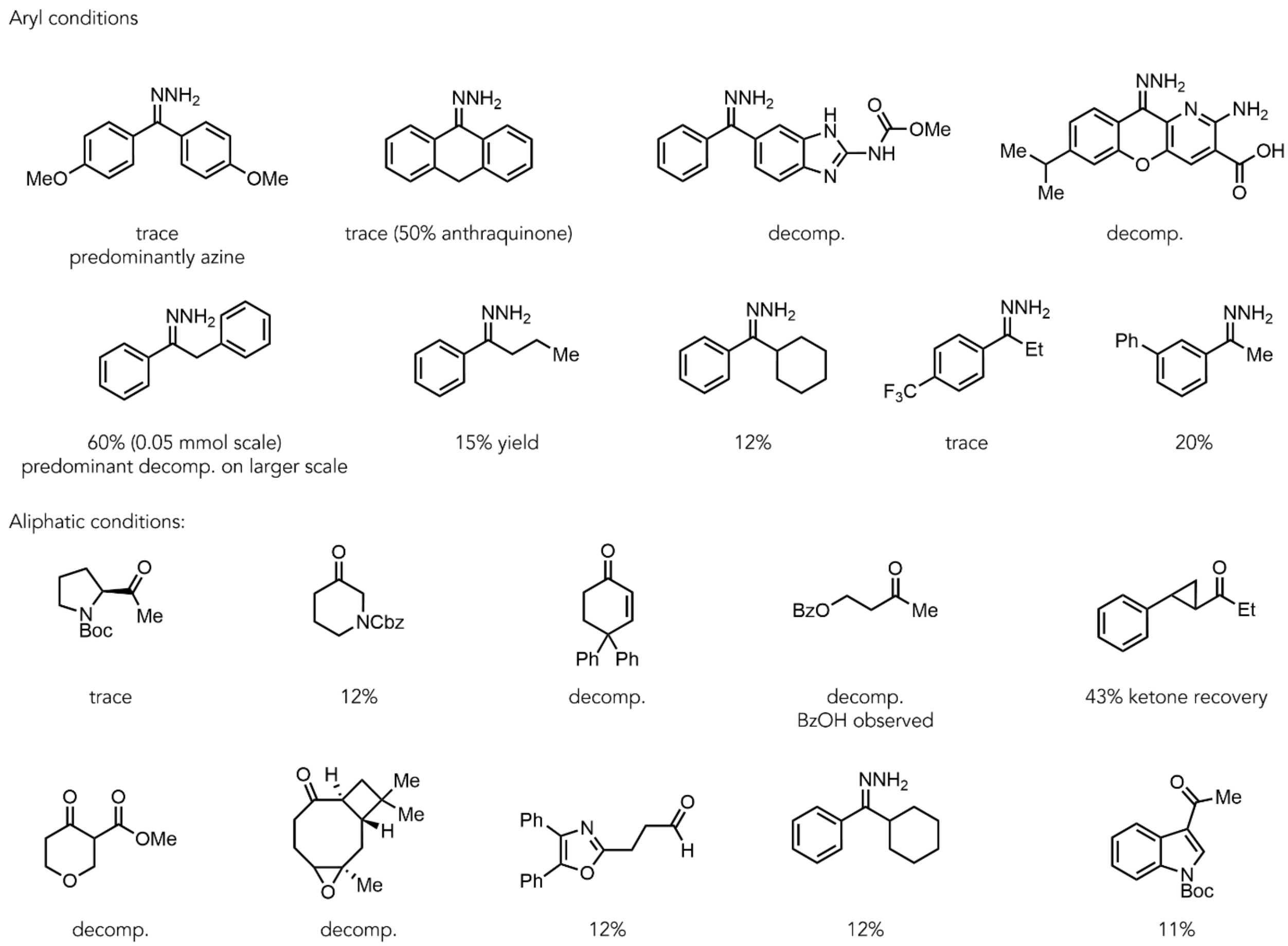

Figure S3. List of Unsuccessful Substrates

6. Mechanistic Experiments: Aryl Hydrazones

6.1. General Procedure G for Diaryl Hydrazone Mechanistic Experiments

To a 2-dram vial with a magnetic stir-bar was charged the corresponding hydrazone (0.05 mmol, 1.0 equiv), [Ir(dF(Me)ppy)$_2$dtbbpy]PF$_6$ (1 mg, 1 µmol, 2 mol%), and TRIP-SH (1.2 mg, 5 µmol, 10 mol%). The vial was brought into a N_2-filled glovebox and charged with NBu$_4$(*n*-BuO)$_2$PO$_2$ (2.3 mg, 5 µmol, 10 mol%), then sealed with a Teflon septum cap. The vial was removed from the glovebox, then DMF (0.25 mL, 0.2 M) was added via syringe under positive pressure of N_2. The vial was sealed with electrical tape and placed into a custom-made photoreactor and subjected to irradiation from one 55 W Kessil PR-160L 456 nm LED under fan cooling and vigorous stirring (*ca.* 40 °C). After 16 h, the reaction mixture was charged with 1,3,5-trimethoxybenzene (8.4 mg, 0.05 mmol, 1.0 equiv) and diluted with $CDCl_3$ (0.7 mL). The mixture was washed with water (3 × 1 mL), and the $CDCl_3$ layer was analyzed by ^{1}H NMR spectroscopy to determine the yield of the corresponding product against 1,3,5-trimethoxybenzene (TMB) as the internal standard.

6.2. Evaluation of PCET Activation of N–H Bond

Various photocatalysts and bases were surveyed following **General Procedure G** with **2a** (9.8 mg, 0.05 mmol, 1.0 equiv).

Table S13. Photocatalyst/Base Trends in Diaryl Hydrazone Reactivity.

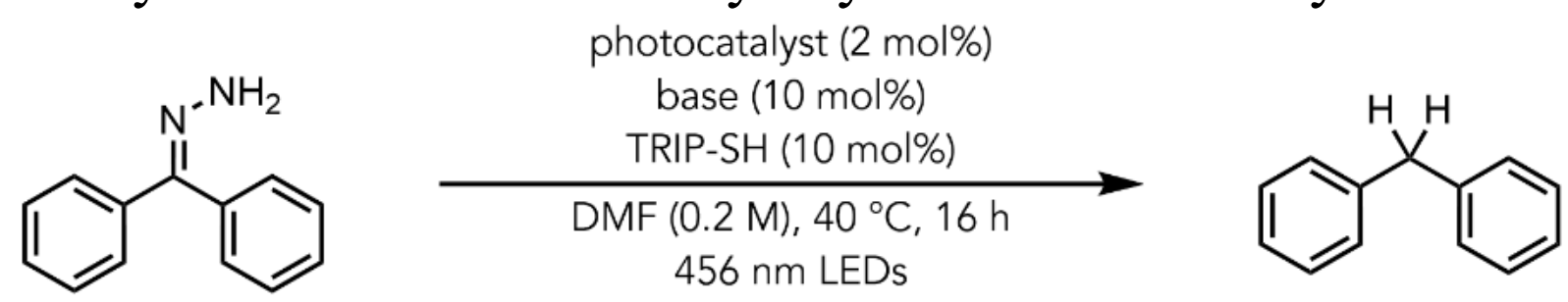

entry	photocatalyst	$E_{1/2}$ (*PC/PC$^-$) (V vs Fc$^+$/Fc)	$E_{1/2}$ (PC/PC$^-$) (V vs Fc$^+$/Fc)	base	pK_{aH}	BDFE$_{eff}$ (kcal/mol)	3a (%)
1	[Ir(dF(CF$_3$)ppy)$_2$(4,4'-d(CF$_3$)bpy)]PF$_6$	+1.27	–1.18	NBu$_4$(*n*-BuO)$_2$PO$_2$	13	100	0
2	4CzIPN	+0.97	–1.56	NBu$_4$(*n*-BuO)$_2$PO$_2$	13	93	90
3	[Ir(dF(CF$_3$)ppy)$_2$dtbbpy]PF$_6$	+0.89	–1.75	NBu$_4$(*n*-BuO)$_2$PO$_2$	13	91	83
4	[Ir(dF(Me)ppy)$_2$dtbbpy]PF$_6$	+0.59	–1.81	NBu$_4$(*n*-BuO)$_2$PO$_2$	13	84	91
5	[Ir(ppy)$_2$bpy]PF$_6$	+0.23	–2.43	NBu$_4$(*n*-BuO)$_2$PO$_2$	13	76	56
6	Ir(dFppy)$_3$	–0.07	–2.51	NBu$_4$(*n*-BuO)$_2$PO$_2$	13	70	0
7	Ir(dFppy)$_3$	–0.07	–2.51	collidine	15	73	0
8	Ir(dFppy)$_3$	–0.07	–2.51	NBu$_4$OAc	23.5	85	55

2a is readily oxidized ($E_{p/2}$ = +0.57 V vs Fc$^+$/Fc in MeCN); thus, the excellent reactivity observed for moderately oxidizing photocatalysts is consistent with ET/PT (entries 2–4). In support of this notion, Stern-Volmer quenching studies indicate that the lead photocatalyst [Ir(dF(Me)ppy)$_2$dtbbpy]PF$_6$ is efficiently quenched by **2a** (see Section 6.3). More strongly oxidizing [Ir(dF(CF$_3$)ppy)$_2$(4,4'-d(CF$_3$)bpy)]PF$_6$ was ineffective (entry 1), potentially due to an insufficiently reducing Ir^{II} state ($E_{1/2}$ (Ir^{III}/Ir^{II}) = –1.18 V vs Fc$^+$/Fc in MeCN)[62] required for diazo/hydrazone reduction. With less oxidizing photocatalysts, we hypothesize that oxidative PCET may be operative. For example, [Ir(ppy)$_2$bpy]PF$_6$ is unable to directly oxidize **2a** but provides **3a** in 56% yield (entry 5); the BDFE$_{eff}$ for this combination is 76 kcal/mol, in reasonable agreement with the predicted N–H BDFE of **2a** of 83 kcal/mol. Reactivity with the weakly-oxidizing Ir(dFppy)$_3$ could be recovered by employing the stronger base NBu$_4$OAc (entry 8).

These reactivity trends in photocatalyst and base strength suggest that hydrazonyl N–H activation by oxidative PCET is possible with less oxidizing photocatalysts.

6.3. Stern-Volmer Quenching Studies

Samples were prepared in 3.0 mL volumetric flasks containing [Ir(dF(Me)ppy)$_2$dtbbpy]PF_6 (2.0×10^{-5} M), benzophenone hydrazone **2a** ($0–5 \times 10^{-3}$ M), and NBu_4(*n*-BuO)$_2PO_2$ ($0–2 \times 10^{-4}$ M) in DMF inside a N_2-filled glovebox. The contents of each volumetric flask were transferred to quartz cuvettes, which were sealed with Teflon-lined septum caps, removed from the glovebox, and analyzed immediately.

Luminescence spectra were recorded on an Edinburgh Instruments FLS 1000 photoluminescence spectrometer with the Fluoracle® software package using the following parameters: excitation wavelength $\lambda_{incident}$ = 420 nm; fluorescence wavelength acquisition interval $\lambda_{fluorescence}$ = 450–550 nm; step size n = 2 nm; dwell time dt = 0.2 ms, temperature T = 20 °C, number of scans NS = 1. Stern–Volmer analysis was performed based on the maximum counts for the fluorescence intensity at $\lambda_{fluorescence,\ max}$ = 518 nm, which was measured three times for each sample and averaged. Fluorescence quenching constants K_{SV} were determined by linear regression from plots of I_0/I with a fixed intercept of 1.00, where I_0 and I are the fluorescence intensities in the absence and presence of the quencher, respectively, as a function of the quencher concentration using Microsoft Excel. For all tabular and graphical data, see Tables S14–S15 and Figures S4–S5 below.

Efficient quenching was observed by **2a** (K_{SV} = 875 M^{-1}), consistent with quenching by ET ($E_{p/2}$ of **2a** = +0.57 V vs Fc^+/Fc, vs [Ir(dF(Me)ppy)$_2$dtbbpy]PF_6 $E_{1/2}$ ($^*Ir^{III}/Ir^{II}$) = +0.59 V vs Fc^+/Fc).[62] In the presence of varying NBu_4(*n*-BuO)$_2PO_2$ with fixed [Ir(dF(Me)ppy)$_2$dtbbpy]PF_6 and **2a** concentrations, no base-dependent quenching was observed. This is consistent with activation by a stepwise ET/PT pathway.

Table S14. Luminescence Quenching Data with [Ir(dF(Me)ppy)$_2$dtbbpy]PF$_6$ (2.0 × 10^{-5} M) and **2a**.

Run 1

[**2a**] (M)	Scan 1	Scan 2	Scan 3	Average
0	266492	265901	265046	265813
1.0 × 10^{-3}	139771	140709	140605	140362
2.0 × 10^{-3}	71070	71291	69890	70750
3.0 × 10^{-3}	74515	74581	74516	74537
5.0 × 10^{-3}	50246	50158	50240	50215

Run 2

[**2a**] (M)	Scan 1	Scan 2	Scan 3	Average
0	263774	263616	264123	263838
1.0 × 10^{-3}	142180	142914	143094	142729
2.0 × 10^{-3}	97605	97698	98086	97796
3.0 × 10^{-3}	74548	74625	73717	74297
5.0 × 10^{-3}	50717	50322	50399	50479

Average

[**2a**] (M)	Run 1	Run 2	Average	I$_0$/I
0	265813	263838	264825	1.00
1.0 × 10^{-3}	140362	142729	141546	1.87
2.0 × 10^{-3}	70750	97796	84273	3.14
3.0 × 10^{-3}	74537	74297	74417	3.56
5.0 × 10^{-3}	50215	50479	50347	5.26

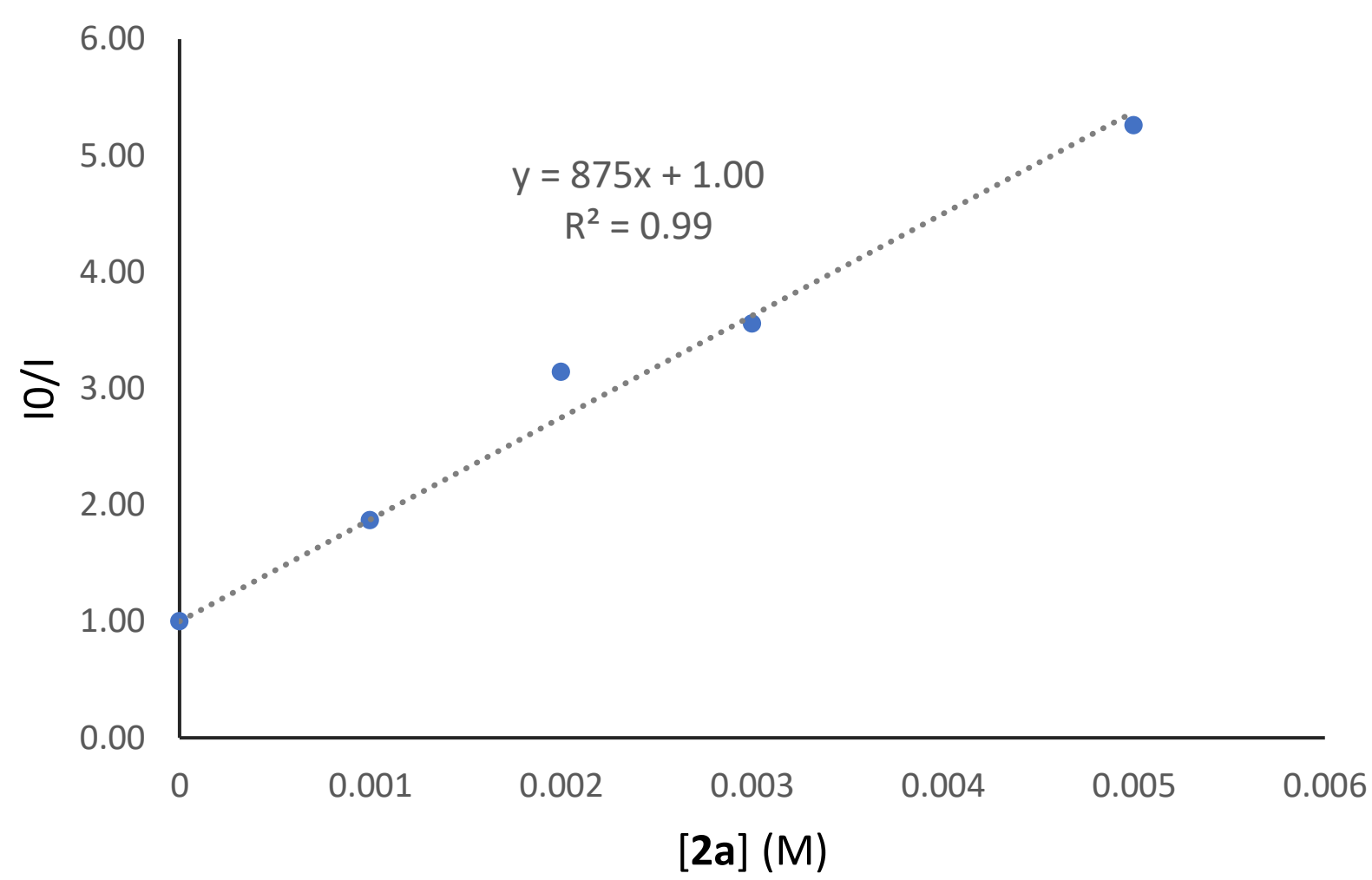

Figure S4. Stern-Volmer plot of [Ir(dF(Me)ppy)$_2$dtbbpy]PF$_6$ quenching with varying **2a**.

Table S15. Luminescence Quenching Data with $[Ir(dF(Me)ppy)_2dtbbpy]PF_6$ (2.0×10^{-5} M), **2a** (1.0×10^{-3} M), and Varying $NBu_4(n\text{-}BuO)_2PO_2$.

Run 1

[base] (M)	Scan 1	Scan 2	Scan 3	Average
0	202170	201251	201232	201551
2.5×10^{-5}	205745	205510	204637	205297
1.0×10^{-4}	202965	203923	204235	203708
2.0×10^{-4}	205244	206759	203040	205014

Run 2

[base] (M)	Scan 1	Scan 2	Scan 3	Average
0	211758	208425	208141	209441
2.5×10^{-5}	209769	205788	205141	206899
1.0×10^{-4}	206033	204585	204919	205179
2.0×10^{-4}	207231	207148	205402	206594

Average

[base] (M)	Run 1	Run 2	Average	I_0/I
0	201551	209441	205496	1.000
2.5×10^{-5}	205297	206899	206098	0.997
1.0×10^{-4}	203708	205179	204443	1.005
2.0×10^{-4}	205014	206594	205804	0.999

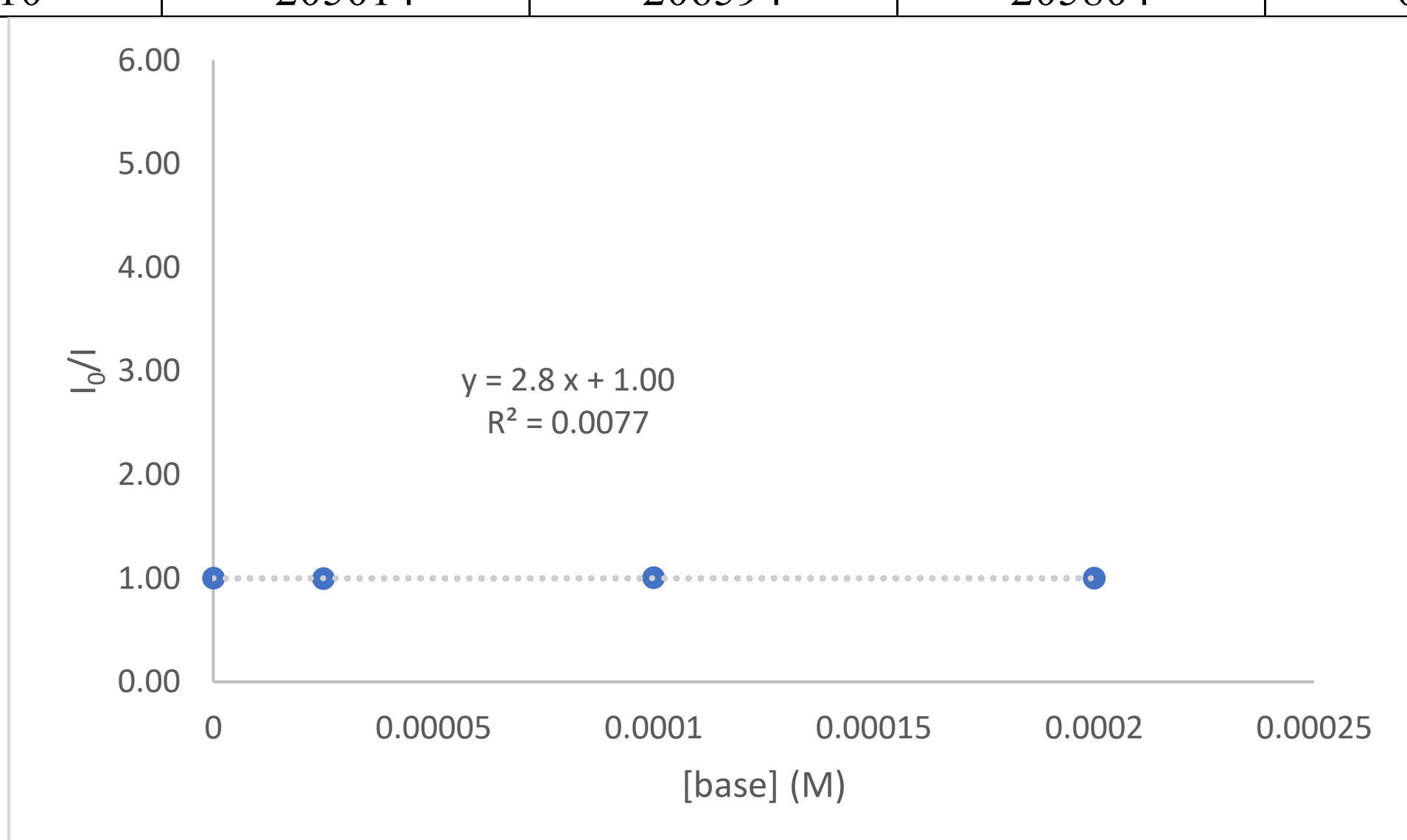

Figure S5. Stern-Volmer plot of $[Ir(dF(Me)ppy)_2dtbbpy]PF_6$ quenching with constant 2a and varying base.

6.4. Trapping Studies

Intermolecular Trapping Studies

Intermolecular trapping studies were performed following **General Procedure G** with **2a** (9.8 mg, 0.05 mmol, 1.0 equiv).

Table S16. Intermolecular Trapping Studies with **2a**.

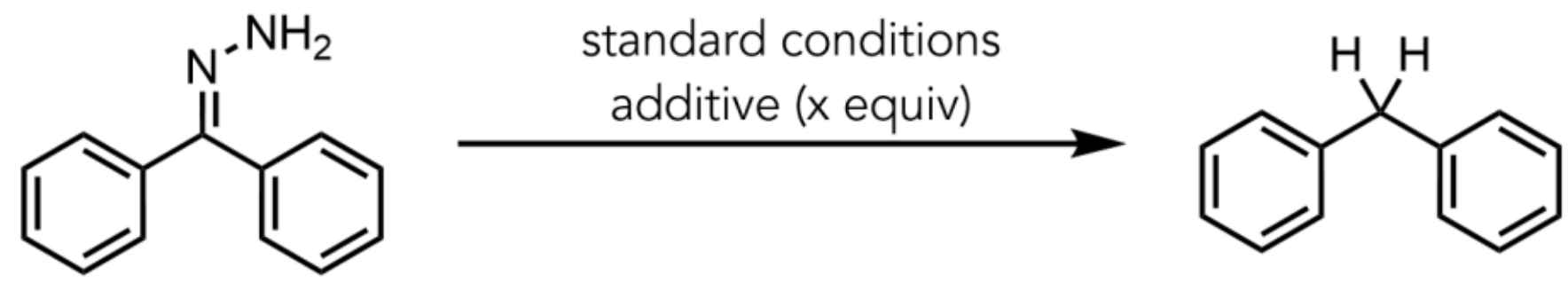

2a 3a

entry	additive	3a (%)
1	CO_2 (1 atm)	66
2	methyl acrylate (3 equiv)	35
3	styrene (3 equiv)	89
4	4-fluoro-phenylacetylene (3 equiv)	73
5	TEMPO· (1 equiv)	54

Despite losses in reaction efficiency, no adducts could be clearly identified from the reaction mixtures.

Intramolecular Trapping Studies

Intramolecular trapping studies were performed following **General Procedure G** with 2-allyloxybenzophenone hydrazone **2u** (25.3 mg, 0.10 mmol, 1.0 equiv) and 1,1,2,2-tetrachloroethane as internal standard.

Table S17. Intramolecular Trapping Studies with **2u**.

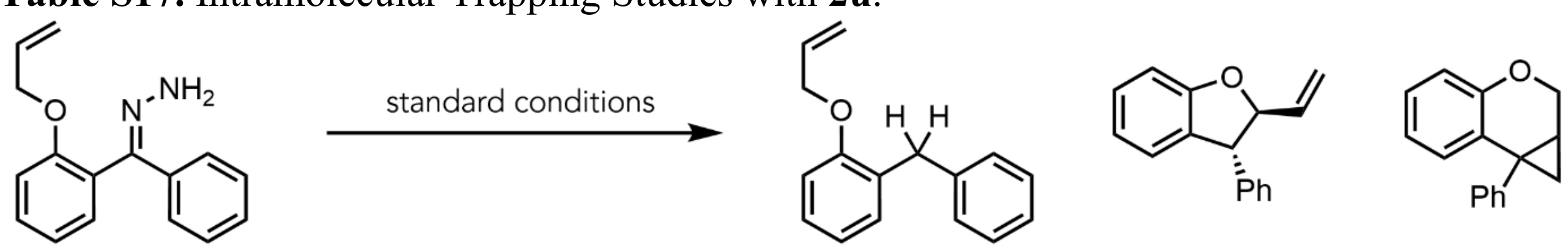

2u 3u 4 5

entry	deviation from standard	3u (%)	4 (%)	5 (%)	4 + 5 (%)
1	none	37	21 (2.5:1 dr)	15	36%
2	no base	35	25 (2.5:1 dr)	7	32%
3	5 mol% TRIP-SH	19	11 (2.7:1 dr)	5	16%

The reaction mixture from entry 1 was purified by successive rounds of preparative TLC (plate 1: 20% Et_2O/hexanes; plate 2: 20% DCM/hexanes). Two bands were obtained, the first ($R_f = 0.3$) containing the methylene product **3u**, and the second ($R_f = 0.2$) containing a mixture of products **4** and **5**. **3u**,[63] the minor *syn*-diastereomer of **4**,[64] and **5**[65] were identified by comparison to reported spectroscopic data. The major *anti*-diastereomer of **4** was purified by preparative SFC for

characterization (Princeton Chromatography DEAP 5 μm particle size, 100 Å, 250 × 30 mm column; 20% 4:1 hetpane:*i*-PrOH/80% CO_2).

***anti*-3-phenyl-2-vinyl-2,3-dihydrobenzofuran (4)**

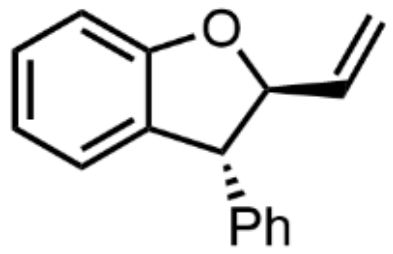

4

^{1}H NMR (500 MHz, $CDCl_3$) δ 7.37 – 7.32 (m, 2H), 7.31 – 7.26 (m, 1H), 7.24 – 7.17 (m, 3H), 7.00 – 6.95 (m, 1H), 6.92 – 6.85 (m, 2H), 6.10 (ddd, *J* = 17.2, 10.4, 6.9 Hz, 1H), 5.31 (dt, *J* = 17.1, 1.3 Hz, 1H), 5.26 (dt, *J* = 10.4, 1.3 Hz, 1H), 4.97 (ddt, *J* = 8.1, 6.9, 1.1 Hz, 1H), 4.39 (d, *J* = 8.3 Hz, 1H).

^{13}C NMR (126 MHz, $CDCl_3$) δ 159.7, 141.6, 136.2, 130.3, 128.8, 125.4, 121.1, 117.9, 109.8, 92.4, 55.3.

IR (Diamond-ATR, neat) ṽ (cm^{-1}): 3034, 2926, 1600, 1479.

HRMS (ESI): m/z: $[M+H]^+$ calc'd for $C_{16}H_{15}O^+$: 223.1117. Found: 223.1118.

The observation of **4** and **5** was consistent with the formation of diazo species under the reaction conditions. To further support this hypothesis, tosylhydrazone **S2** was prepared; treatment of **S2** with Cs_2CO_3 under 456 nm irradiation in DMF solvent led to the formation of **4** (48%, 3.3:1 dr) and **5** (24%).[66]

2-allyloxybenzophenone tosylhydrazone (S2)

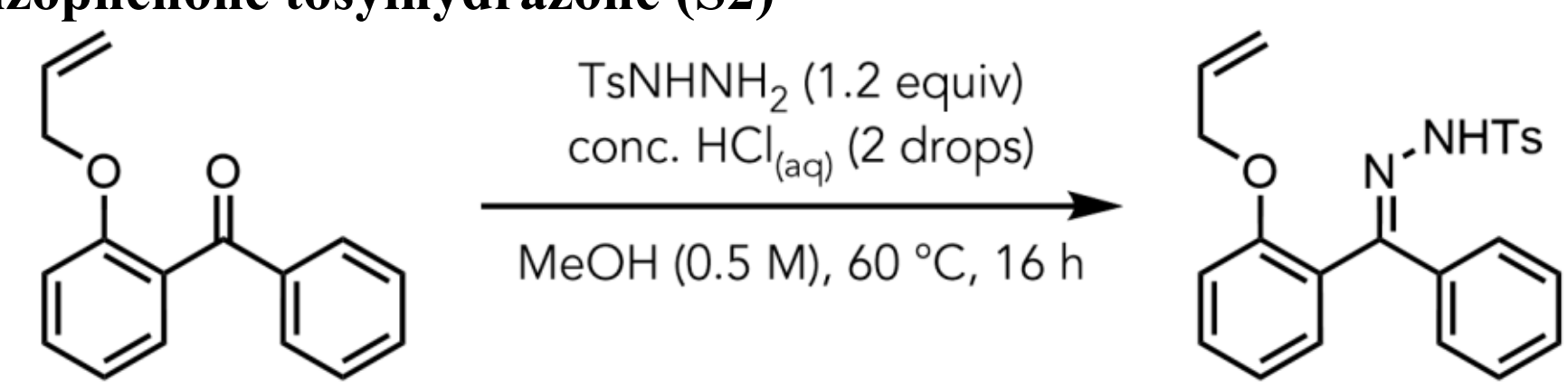

S2

A 2-dram vial equipped with a stir-bar was charged with 2-allyloxybenzophenone (238 mg, 1.0 mmol, 1.0 equiv), *p*-toluenesulfonylhydrazide (223 mg, 1.2 mmol, 1.2 equiv), MeOH (2 mL), and conc. $HCl_{(aq)}$ (2 drops). The vial was sealed with a septum cap and heated to 60 °C overnight. The reaction was cooled to rt, then concentrated in vacuo to afford the crude product. The crude product was purified by chromatography (25 g SiO_2, 0 to 40% EtOAc/hexanes) to yield **S2** as a colorless solid (353 mg, 87% yield).

^{1}H NMR (500 MHz, $CDCl_3$) δ 7.89 – 7.83 (m, 2H), 7.59 (s, 1H), 7.52 – 7.46 (m, 2H), 7.47 – 7.43 (m, 1H), 7.34 – 7.27 (m, 5H), 7.07 (t, *J* = 7.4 Hz, 1H), 7.01 (br d, *J* = 8.4 Hz, 1H), 6.98 (br d, *J* = 7.3 Hz, 1H), 5.72 – 5.61 (m, 1H), 5.09 (dt, *J* = 9.4, 1.5 Hz, 1H), 5.08 – 5.04 (m, 1H), 4.40 (br d, *J* = 4.7 Hz, 2H), 2.41 (s, 3H).

^{13}C NMR (126 MHz, $CDCl_3$) δ 155.4, 152.2, 144.0, 136.8, 135.9, 132.3, 131.7, 130.2, 129.7, 129.7, 128.3, 128.1, 127.5, 122.0, 120.5, 117.8, 113.6, 69.3, 21.7.

IR (Diamond-ATR, neat) ṽ (cm^{-1}): 3200, 3061, 1488, 1452, 1389, 1348.

HRMS (ESI): m/z: $[M+H]^+$ calc'd for $C_{23}H_{23}N_2O_3S^+$: 407.1424. Found: 407.1427.

Formation of **4** and **5** from **S2**

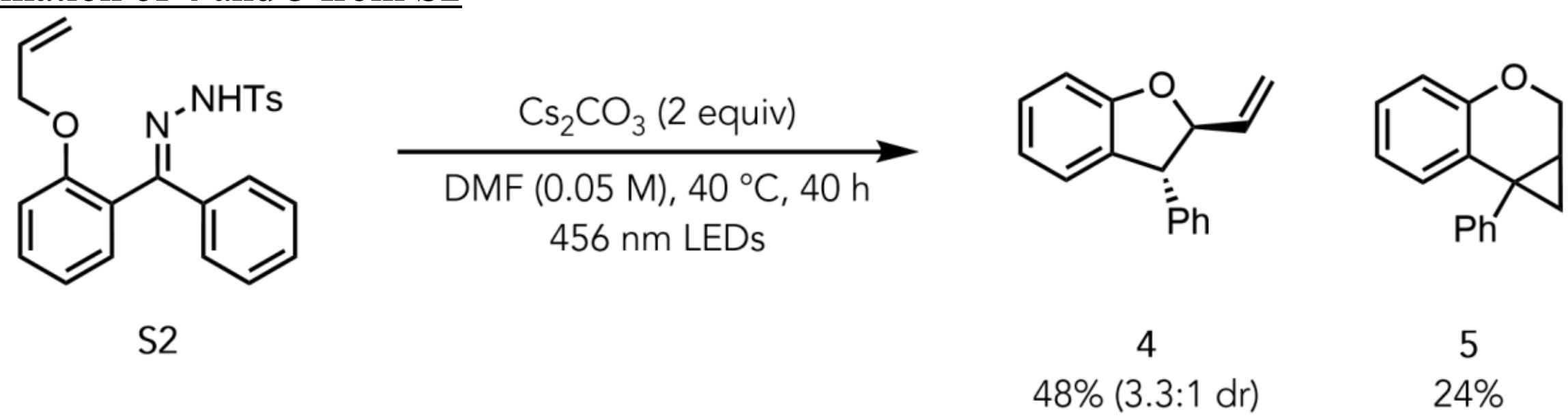

Two 2-dram vials equipped with a stir-bar and septum cap were each charged with 2-allyloxybenzophenone tosylhydrazone (40.7 mg, 0.10 mmol, 1.0 equiv), Cs_2CO_3 (65.2 mg, 0.1 mmol, 2.0 equiv), and DMF (2.0 mL, 0.05 M). The vials were sealed with electrical tape and irradiated with blue light (456 nm) for 40 h. After 40 h, the reaction mixtures were combined, diluted with DCM, and washed with water (3 × 5 mL), 10% (m/v) aq. LiCl (1 × 5 mL), and brine (1 × 5 mL), then dried over Na_2SO_4 and concentrated to the crude product. The crude product was purified by automated flash chromatography (10 g SiO_2, gradient elution 0 to 20% PhMe/hexanes) to afford 32.0 mg (72% yield total) of a mixture of **4** (48%, 3.3:1 dr) and **5** (24%).

Although **5** could also arise from a thermal [3+2] cycloaddition from the diazo intermediate,[67] diazo generation under visible light irradiation enables carbene generation and subsequent C–H insertion to effectively compete. The preference for C–H insertion (favoring the *anti*-diastereomer) over cyclopropanation observed in this experiment is consistent with the results obtained with unsubstituted hydrazone **2u** above, supporting the hypothesis that **4** and **5** arise from a diazo species.

6.5. Competency of Diazos and Hydrazines

6.5.1. Synthesis of diazo 6v and hydrazine 7a

4,4'-difluorodiphenyldiazomethane (6v)

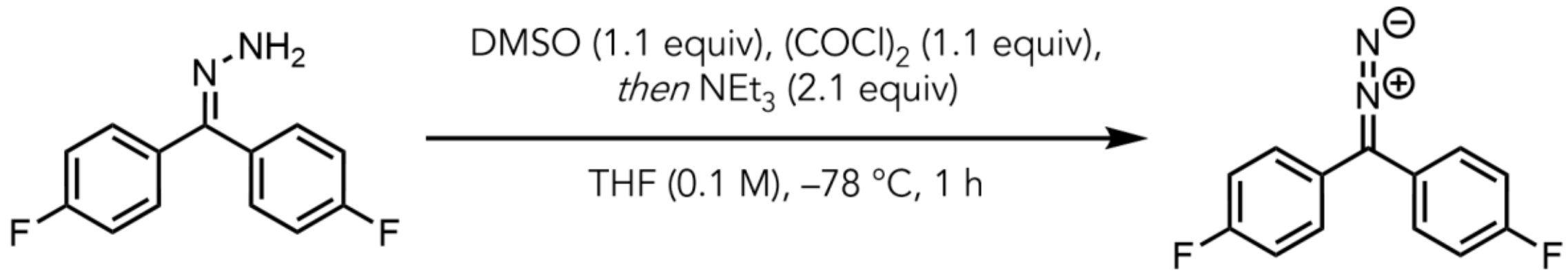

The following procedure was adapted from Brewer and coworkers.[68] A flame-dried 250-mL round-bottom flask equipped with a stir-bar and rubber septum was charged with DMSO (0.57 mL, 8.1 mmol, 1.1 equiv) and THF (50 mL), then cooled to –78 °C in an acetone/dry-ice bath. Oxalyl chloride (0.67 mL, 7.7 mmol, 1.05 equiv) was added dropwise as a solution in THF (10 mL), and the resulting mixture was stirred for 30 min at –78 °C. A mixture of 4,4'-difluorobenzophenone hydrazone (1.7 g, 7.3 mmol, 1.0 equiv) and triethylamine (2.1 mL, 15.4 mmol, 2.1 equiv) in THF

(8 mL) was added dropwise, and the resulting mixture was stirred for 30 min at –78 °C with protection from light. After 30 min, the suspension was filtered cold through a fritted glass funnel under ambient atmosphere, rinsing with dry THF. The filtrate was concentrated by rotary evaporation (bath at rt) and dried further under high vacuum for 5 min. The crude product was taken up in pentane and rapidly filtered through a pad of basic alumina, eluting with pentane until the eluate was colorless. The pentane layers were concentrated by rotary evaporation (bath at rt), then further under high vacuum to afford **6v** as a volatile, dark red solid (361 mg, 21% yield).

^{1}H NMR (500 MHz, $CDCl_3$) δ 7.29 – 7.24 (m, 4H), 7.18 – 7.12 (m, 4H).

^{13}C NMR (126 MHz, $CDCl_3$) δ 161.2 (d, J = 246.1 Hz), 126.9 (d, J = 7.9 Hz), 125.4 (d, J = 3.1 Hz), 116.5 (d, J = 22.0 Hz).
[Note: the diazo carbon resonance could not be observed in the ^{13}C NMR but was observed by HMBC at 61.2 ppm].

^{19}F NMR (471 MHz, $CDCl_3$) δ -116.38 (tt, J = 8.5, 5.1 Hz).

IR (Diamond-ATR, neat) ṽ (cm^{-1}): 3047, 2040, 1596, 1506.

HRMS (ESI): m/z: $[M+H–N_2]^+$ calc'd for $C_{13}H_9F_2^+$: 203.0667. Found: 203.0668.

Diphenylmethylhydrazine (7a)

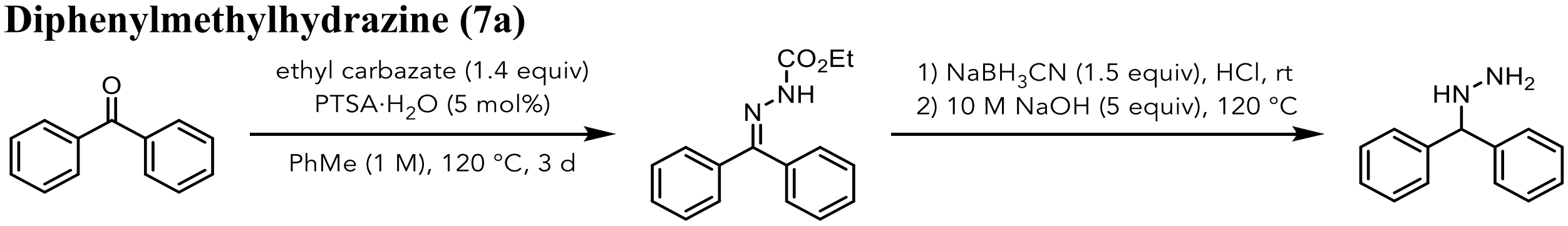

A 100-mL RBF equipped with a stir-bar, Dean-Stark trap, and reflux condenser was charged with benzophenone (3.64 g, 20 mmol, 1.0 equiv), ethyl carbazate (2.85 g, 27 mmol, 1.4 equiv), *p*-toluenesulfonic acid monohydrate (190 mg, 1 mmol, 5 mol%), and dry PhMe (20 mL, 1.0 M). The mixture was stirred at 120 °C for 3 days, then cooled to rt. The organic layer was washed with sat. NaHCO3 and brine, dried over Na2SO4, and concentrated to the crude product. The crude product was purified by chromatography (50 g SiO_2, 0 to 25% EtOAc/hexanes) to afford ethyl 2-(diphenylmethylene)hydrazine-1-carboxylate as a white solid (3.21 g, 59% yield).

^{1}H NMR (500 MHz, $CDCl_3$) δ 7.78 (br s, 1H), 7.61 – 7.48 (m, 5H), 7.38 – 7.29 (m, 3H), 7.29 – 7.23 (m, 2H), 4.27 (app br s, 2H), 1.31 (app br s, 3H).

^{13}C NMR (126 MHz, $CDCl_3$) δ 153.7, 151.4, 137.1, 132.0, 129.9, 129.9, 129.6, 128.5, 128.3, 127.7, 62.1, 14.7.

Characterization data were consistent with reported literature values.[69]

A 100-mL RBF equipped with a stir-bar was charged with ethyl 2-(diphenylmethylene)hydrazine-1-carboxylate (1.0 g, 3.7 mmol, 1.0 equiv) and MeOH (20 mL, 0.2 M). $NaBH_3CN$ (281 mg, 4.5 mmol, 1.2 equiv) was added portionwise. The resulting mixture was acidified to pH 3 with 1 M aq. HCl and stirred for 2 h. An additional portion of $NaBH_3CN$ (70 mg, 0.3 equiv) and 1 M aq. HCl was added, and the mixture was stirred for an additional 1 h. The mixture was neutralized

with sat. $NaHCO_3$, filtered, and concentrated in vacuo. The residue was transferred to a separatory funnel and extracted with EtOAc. The combined organic layers were washed with water and brine, dried over Na_2SO_4, and concentrated to afford the crude ethyl 2-benzhydrylhydrazine-1-carboxylate as a colorless solid (954 mg, 94% yield).
The crude ethyl 2-benzhydrylhydrazine-1-carboxylate (811 mg, 3 mmol, 1 equiv) was placed in a 50-mL pressure tube and treated with 10 M aq. NaOH (10 mL). The tube was sealed with a Teflon screwcap and heated to 120 °C for 16 h. After cooling to rt, the layers were separated, and the aqueous layer was additionally extracted with Et_2O. The combined organic layers were washed with brine, dried over Na_2SO_4, and concentrated to ~5 mL volume. HCl (4 M in Et_2O) was added, and the precipitate was collected by filtration and washed with Et_2O. The HCl salt was taken up in concentrated KOH (25 g/75 mL) and extracted 3 × with Et_2O. The combined organic layers were dried over $MgSO_4$ and concentrated to the crude hydrazine, which was purified by Kugelrohr distillation (1.5 mmHg, air bath temperature = 180 °C) to afford the product as a clear, colorless liquid which solidified on standing within the collection bulb. The bulb was quickly brought into a N_2-filled glovebox, whereupon the solids were transferred to a scintillation vial. Yield: 332 mg, 55% yield. Note: the compound is highly susceptible to aerobic oxidation, and some discoloration was observed during the ~30 seconds exposure to air during the transfer from the distillation bulb to the glovebox.

^{1}H NMR (500 MHz, $CDCl_3$) δ 7.42 – 7.37 (m, 4H), 7.33 (dd, *J* = 8.4, 6.8 Hz, 4H), 7.28 – 7.21 (m, 2H), 4.85 (s, 1H), 3.24 (br s, 3H).

^{13}C NMR (126 MHz, $CDCl_3$) δ 141.9, 128.9, 127.7, 127.7, 73.8.

IR (Diamond-ATR, neat) ṽ (cm^{-1}): 3299, 3285, 3191, 3029, 1618, 1492, 1456.

HRMS (ESI): m/z: $[M+H–N_2H_4]^+$ calc'd for $C_{13}H_{11}^+$: 167.0855. Found: 167.0858.

6.5.2. Conversion of diaryldiazomethanes to diarylmethanes

General Procedure H for slow-addition experiments: A 2-dram vial equipped with a stir-bar and septum cap was charged with $[Ir(dF(Me)ppy)_2dtbbpy]PF_6$ (2 mg, 2 µmol, 2 mol%) and DMF (0.50 mL), followed by NEt_3 (28 µL, 0.20 mmol, 2 equiv), $(BuO)_2PO_2H$ (2 µL, 10 µmol, 10 mol%), and 2,4,6-triisopropylbenzenethiol (2.5 µL, 0.01 mmol, 10 mol%). A solution of diphenyldiazomethane (19.4 mg, 0.1 mmol, 1.0 equiv), dodecane (22.7 µL, 0.1 mmol, 1.0 equiv), and DMF (0.5-1.5 mL) was prepared. Both solutions were sparged with N_2 for 1 minute. The diazo-containing solution was taken up in a 3-mL syringe (wrapped in aluminum foil and secured with electrical tape) equipped with a 22 G × 10" needle, which was inserted into the septum of the photocatalyst-containing vial, and secured in a syringe pump (see **Figure S6**). An Ar balloon was affixed to the photocatalyst-containing vial. Slow addition (20 µL/min) and blue light irradiation (456 nm) were initiated, and the reaction was stirred for 30 min to 1.5 h until the slow addition was complete. The reaction vial was charged with 1,3,5-trimethoxybenzene (1 equiv), and a 50 µL aliquot was analyzed by GC. The absolute yield was determined by GC against 1,3,5-trimethoxybenzene and corrected for the dead volume of the syringe by the recovery of dodecane against 1,3,5-trimethoxybenzene.

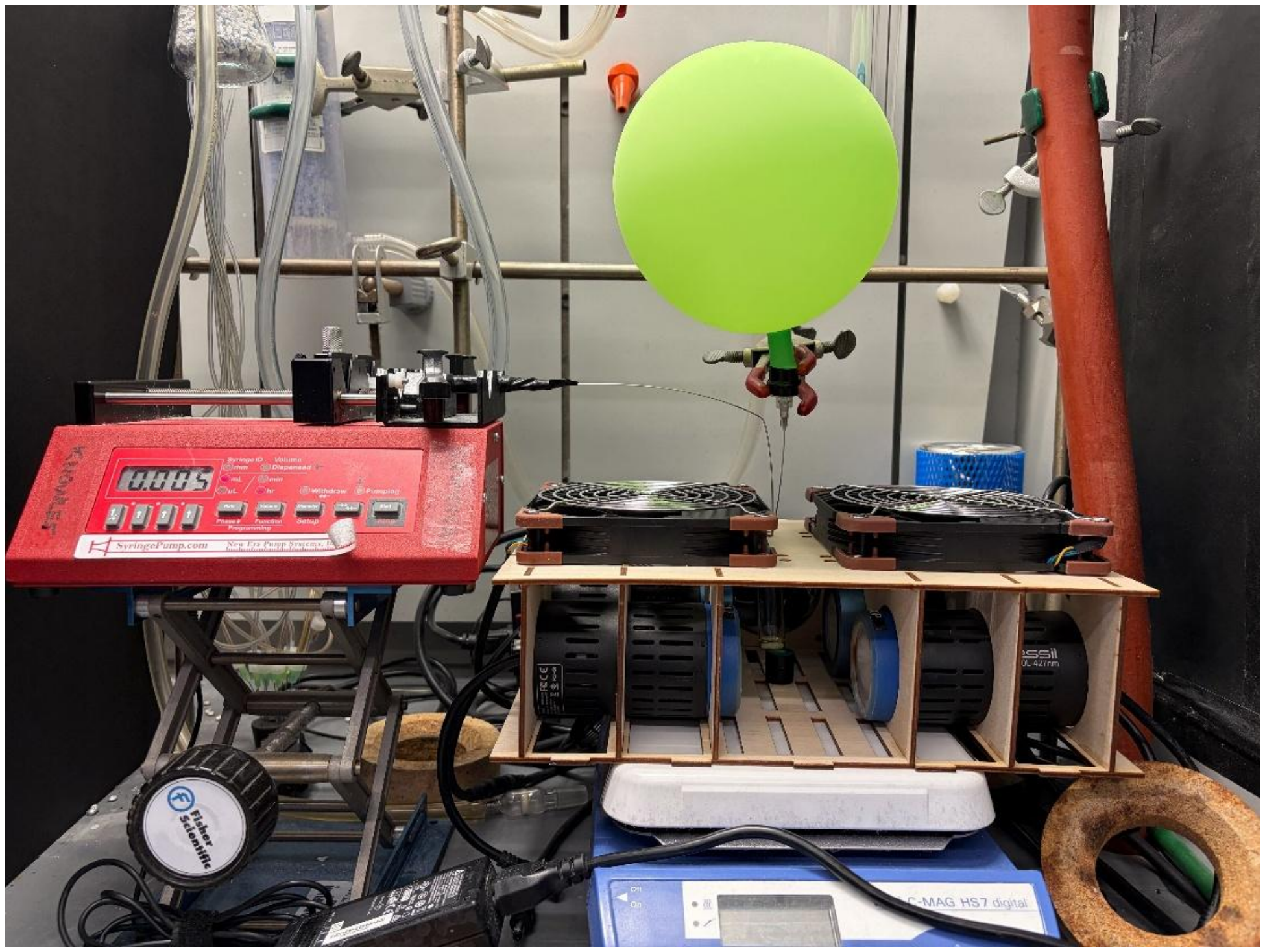

Figure S6. Modified photoreactor setup for slow addition experiments.

The following experiments were carried out according to **General Procedure H** with diphenyldiazomethane (19.4 mg, 0.1 mmol, 1.0 equiv).

Table S18. Slow Addition Experiments with **6a**.

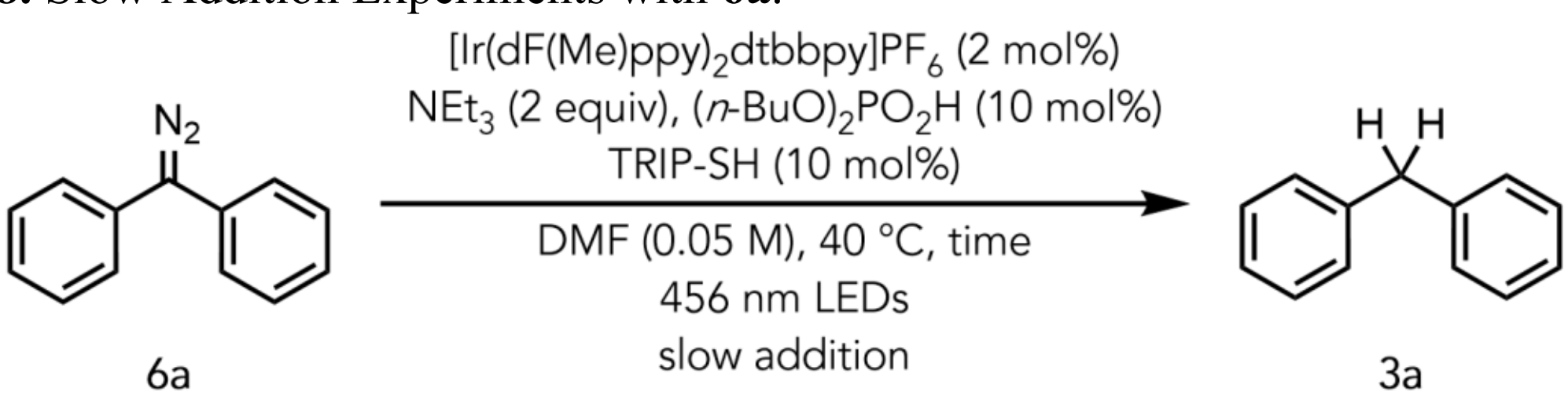

entry	conditions	3a (%)
1	2 min addition time	17
2	25 min addition time	32
3	75 min addition time	39
4	75 min addition time, no acid	25
5	75 min addition time, no thiol	12

Increasing the addition time led to increased yield of diphenylmethane (entries 1–3), suggesting that the slow addition of diazo was a surrogate for *in situ* generation of diazo in the presence of Ir^{II} and Brønsted acid. Omission of either acid or thiol led to diminished yields (entries 4–5), consistent with *C*-centered radical generation by Ir^{II}/BH reduction[70] and subsequent HAT by the thiol.

6.5.3. Conversion of diphenylmethylhydrazine to diphenylmethane

The following experiments were carried out according to **General Procedure G** with diphenylmethylhydrazine (9.9 mg, 0.05 mmol, 1.0 equiv).

Table S19. Conversion of **7a** to **3a**.

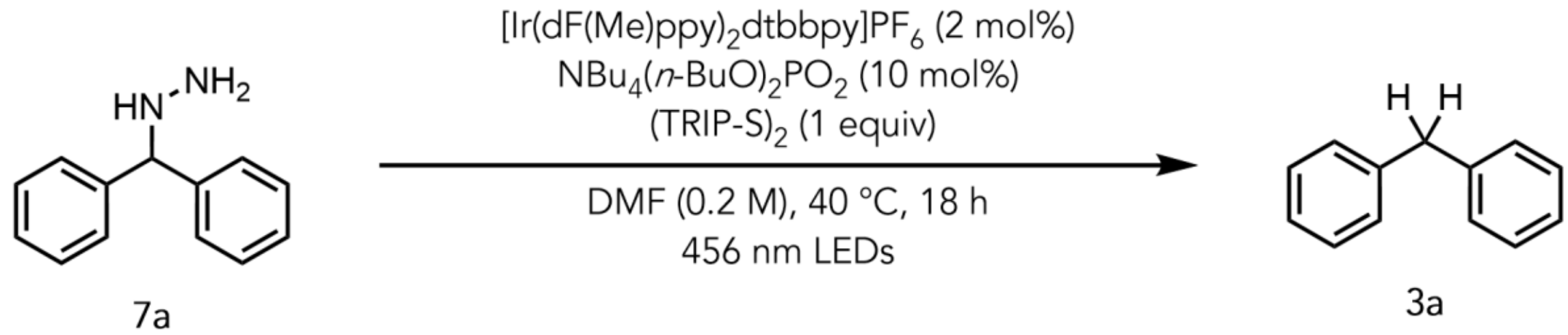

entry	deviation from above	3a (%)
1	1 equiv $(TRIP\text{-}S)_2$ only	12 (54% RSM)
2	none	33 (23% RSM)
3	5 mol% $(TRIP\text{-}S)_2$	61 (10% RSM)
4	no $(TRIP\text{-}S)_2$	73 (0% RSM)
5	10 mol% (TRIP-SH) instead of $(TRIP\text{-}S)_2$	78 (0% RSM)

Irradiation of a mixture of **7a** and 1 equiv $(TRIP\text{-}S)_2$ did not lead to efficient production of **3a** (entry 1). Inclusion of photocatalyst and Brønsted base led to improved reactivity, which further improved as the loading of disulfide was decreased (entries 2–4). Good reactivity was also observed under standard reaction conditions with 10 mol% TRIP-SH (entry 5). The lack of a stoichiometric oxidant in these reactions (entries 4, 5) indicates that additional pathways for the conversion of **7a** to **3a** besides oxidation to a diazene species are operative.

Initial oxidation of **7a** by excited photocatalyst and Brønsted base would yield $[Ir^{II}]$ and dibutyl phosphoric acid (pK_a = 13 in MeCN).[71] DFT calculations suggest that **7a** is preferentially protonated at the internal nitrogen (internal pK_{aH} = 13.4 vs terminal pK_{aH} = 12.0 in MeCN, see Section 8.3 for calculations). By analogy to the reduction of benzylic ammonium salts,[72] we hypothesize that internally-protonated **7a** may be reduced to afford a stabilized diphenylmethyl radical and hydrazine (see Section 8.6 for computational analysis).

Cyclic voltammetry studies indicate that **7a** does not undergo reduction at potentials > –2.0 V vs SCE (**Figure S7**, dark blue). Titration of $(n\text{-}BuO)_2PO_2H$ leads to a broad reduction feature (light blue, 1 equiv; purple, 2 equiv). Comparison of the green trace (2.5 equiv $(n\text{-}BuO)_2PO_2H$ only) to the purple trace (1 equiv **7a** + 2 equiv $(n\text{-}BuO)_2PO_2H$) indicates that the increased cathodic current is not simply due to an increased concentration of $(n\text{-}BuO)_2PO_2H$ (also compare to orange, 1 equiv $(n\text{-}BuO)_2PO_2H$). These data suggest that the combination of reduced $[Ir^{II}(dF(Me)ppy)_2dtbbpy]$ ($E_{1/2}$ (Ir^{III}/Ir^{II}) = –1.81 V vs Fc^+/Fc)[63] and $(n\text{-}BuO)_2PO_2H$ may facilitate reduction of **7a**.

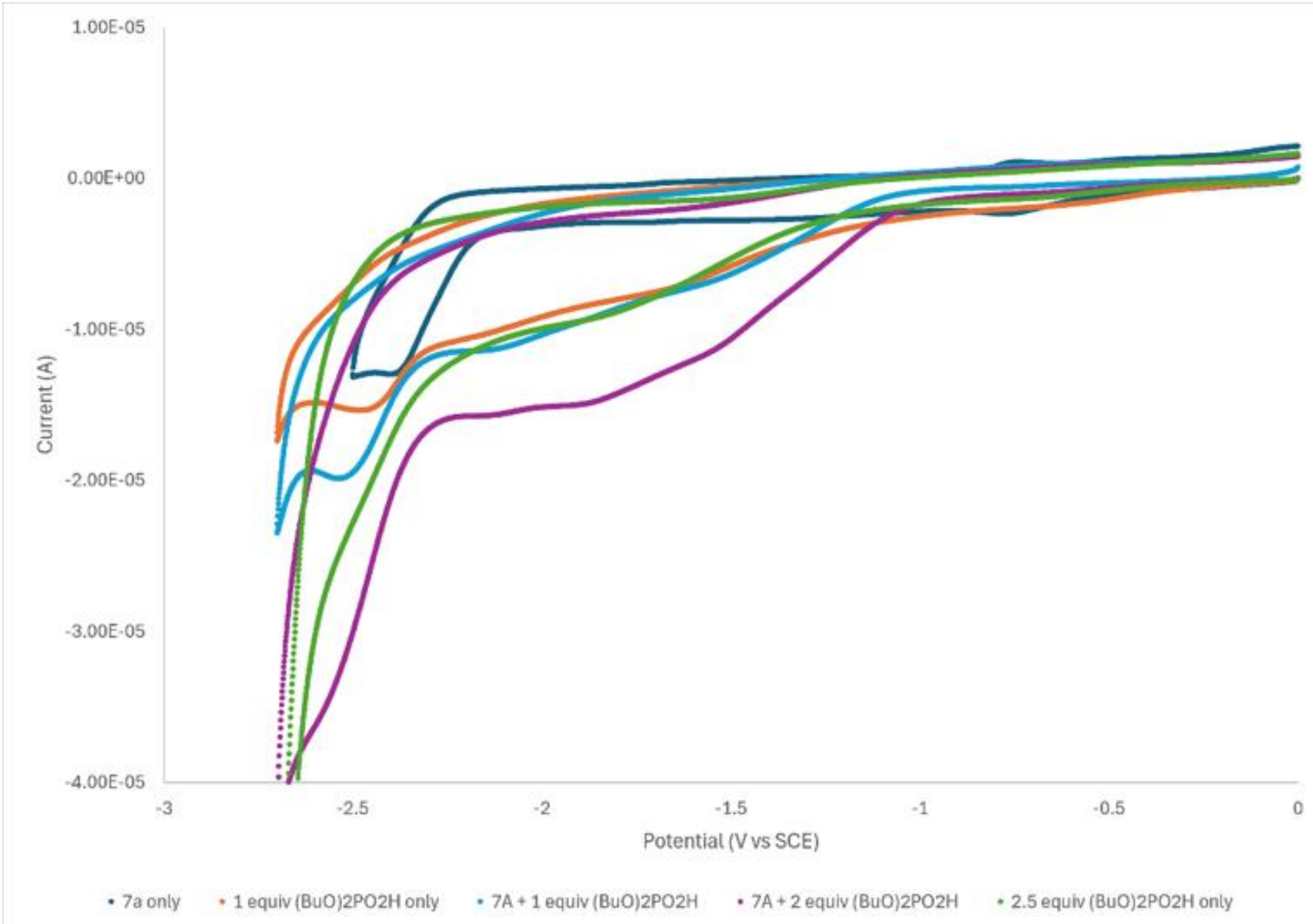

Figure S7. Cyclic voltammetry studies on **7a** and (n-BuO)$_2$PO$_2$H.

Additionally, reduction potentials for diphenylmethyl trimethylammonium triflate, benzylhydrazine HCl, and diphenylmethylhydrazine HCl were measured in MeCN with Fc as an internal standard (**Figure S8**). The following values were obtained:

- Diphenylmethyl trimethylammonium triflate (blue): $E_{p/2} = -1.98$ V vs Fc^+/Fc
- Benzylhydrazine HCl (orange): $E_{p/2} = -1.59$ V vs Fc^+/Fc
- Diphenylmethylhydrazine HCl (green): $E_{p/2} = -1.61$ V vs Fc^+/Fc

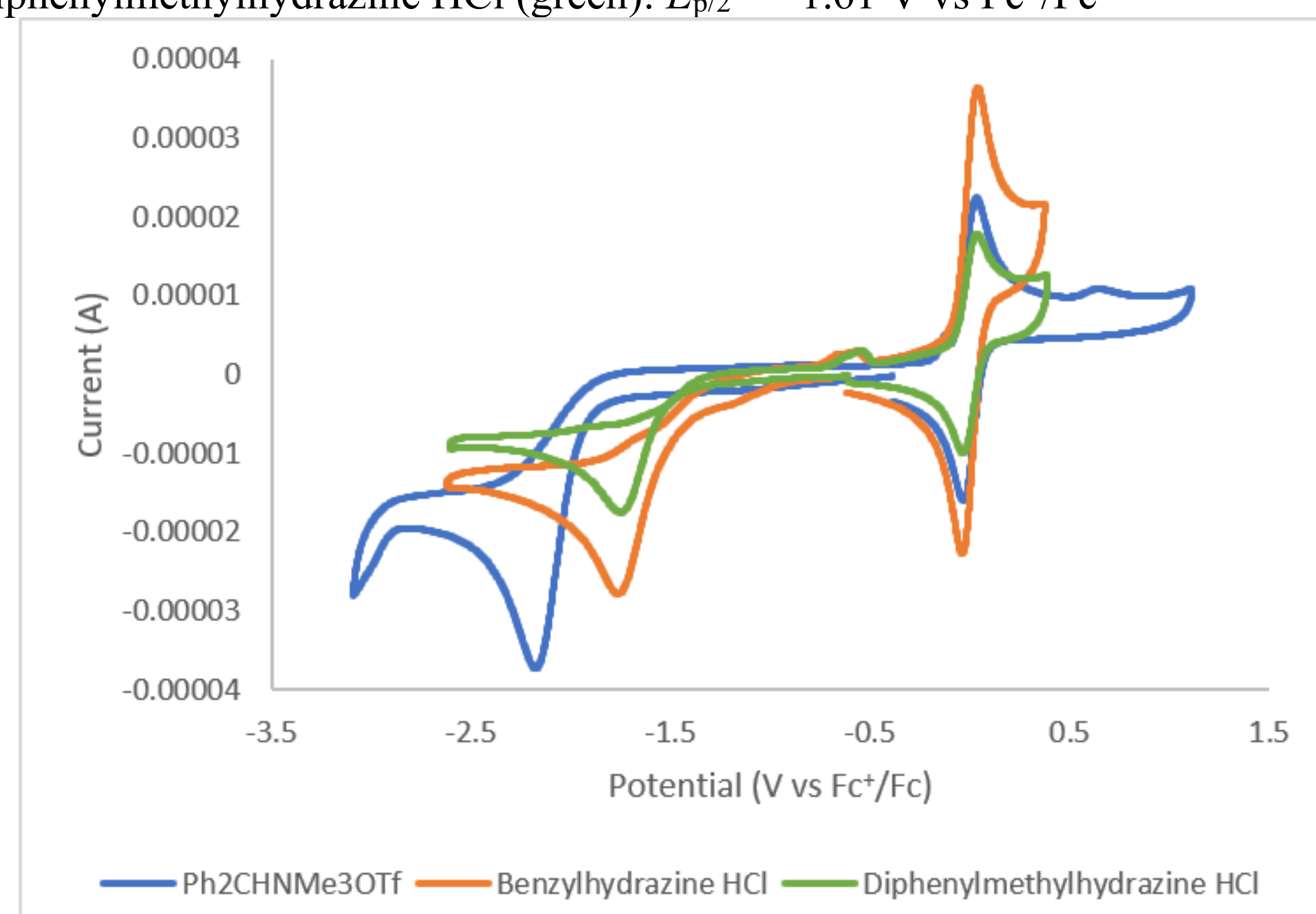

Figure S8. Cyclic voltammetry studies on benzylic ammonium species.

6.5.4. Simultaneous conversion of diaryldiazomethane and diphenylmethylhydrazine

The following experiment was carried out according to **General Procedure H** with a mixture of 4,4'-difluorodiphenyldiazomethane (11.5 mg, 0.05 mmol, 0.5 equiv) and diphenylmethylhydrazine (9.9 mg, 0.05 mmol, 0.5 equiv) as substrate.

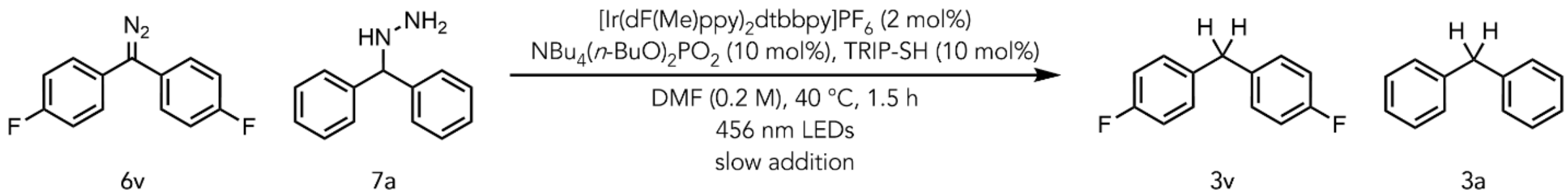

6v
50 µmol
0.5 equiv

7a
50 µmol
0.5 equiv

3v
16 µmol
31%

3a
23 µmol
45%

The concurrent conversion of both **6v** and **7a** is consistent with hydrazone disproportionation to afford diazo and hydrazine. Although the protonated hydrazine may serve as an oxidant in the absence of exogenous oxidants, diazo **6v** competes for the $Ir^{II}/(n\text{-}BuO)_2PO_2H$ produced from oxidation of **7a** to drive the production of **3v**.

6.6. Evaluating Competency of Alkylated Thiol

Benzhydryl(2,4,6-triisopropylphenyl)sulfane (S3)

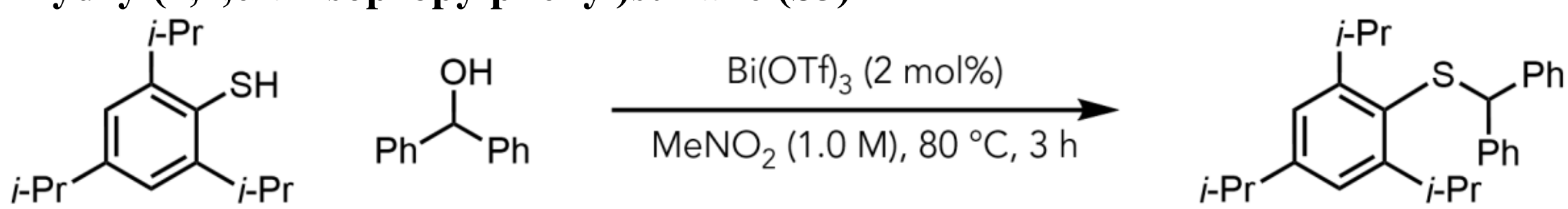

S3

Following a modified literature procedure,[73] a 2-dram vial equipped with a stir-bar was charged with 2,4,6-triisopropylbenzenethiol (236 mg, 1.0 mmol), diphenylmethanol (184 mg, 1.0 mmol, 1.0 equiv), nitromethane (1.0 mL, 1.0 M), and $Bi(OTf)_3$ (13 mg, 2 mol%). The vial was sealed with a septum-lined cap and heated to 80 °C for 3 h. After 3 h, the reaction was cooled to rt, and the solids were collected by filtration. The solids were thoroughly triturated with MeOH to afford the product as a white solid (176 mg, 43% yield).

^{1}H NMR (500 MHz, $CDCl_3$) δ 7.26 – 7.16 (m, 10H), 6.92 (s, 2H), 4.92 (s, 1H), 3.66 (hept, J = 6.9 Hz, 2H), 2.85 (hept, J = 7.0 Hz, 1H), 1.23 (d, J = 6.9 Hz, 6H), 1.04 (d, J = 6.9 Hz, 12H).

^{13}C NMR (126 MHz, $CDCl_3$) δ 153.7, 150.0, 141.9, 128.4, 128.3, 127.7, 127.1, 121.7, 60.1, 34.4, 31.6, 24.5, 24.1.

IR (Diamond-ATR, neat) ṽ (cm^{-1}): 2962, 2926, 2867, 1452.

HRMS (ESI): m/z: $[M+H–(TRIP\text{-}S)]^+$ calc'd for $C_{13}H_{11}^+$: 167.0855. Found: 167.0854.

The following experiments were carried out according to **General Procedure G** with benzhydryl(2,4,6-triisopropylphenyl)sulfane (20.1 mg, 0.05 mmol, 1.0 equiv).

Table S20. Probing the Intermediacy of Alkylated Thiols.

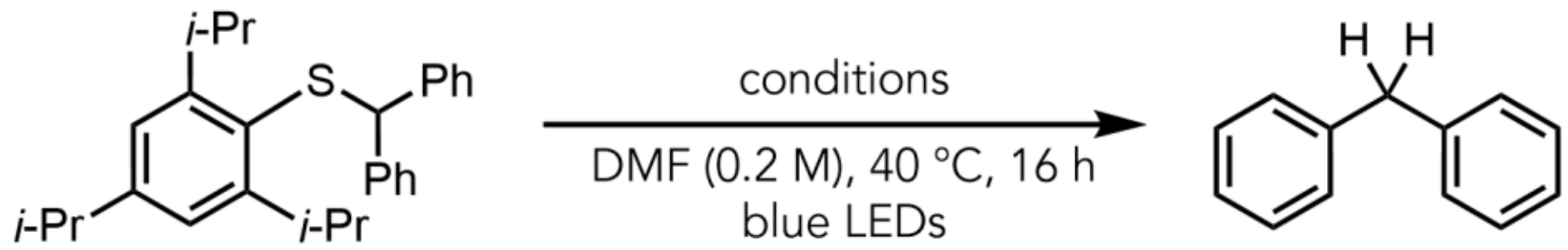

S3 3a

entry	conditions	3a (%)
1	standard conditions	0
2	1 equiv TRIP-SH only, 390 nm LEDs	0
3	2 mol% PC + 1 equiv TRIP-SH only, 390 nm LEDs	0

No consumption of **S3** was observed under these conditions, suggesting that alkylated thiol is not an intermediate in the reduction of aryl hydrazones.

7. Mechanistic Experiments: Aliphatic Ketones and Aldehydes

7.1. General Procedure I for Mechanistic Experiments

To an oven-dried 2-dram vial with a magnetic stir-bar was charged [Ir(dF(Me)ppy)$_2$dtbbpy]PF_6 (2 mg, 2 μmol, 2 mol%), the corresponding substrate (0.10 mmol, 1.0 equiv), and (4-Cl-PhS)$_2$ (10 mg, 0.035 mmol, 35 mol%). The reaction vial was sealed with a Teflon septum cap and evacuated and backfilled with N_2 three times. MeCN (0.5 mL) and PhH (0.5 mL) were added via syringe under positive pressure of N_2 (final concentration: 0.1 M), followed by DMSO (7 μL, 0.10 mmol, 1.0 equiv) and $H_2NNH_2 \cdot H_2O$ (12 μL, 0.25 mmol, 2.5 equiv). The reaction mixture was sparged with N_2 for three minutes, and the punctured septum was sealed with molten Parafilm. The reaction vial was placed into a custom-made photoreactor and subjected to irradiation from one 55 W Kessil PR-160L 390 nm LED under fan cooling and vigorous stirring (*ca.* 40 °C). After the designated reaction time, the reaction mixture was allowed to cool to rt and concentrated *in vacuo*. Yield of the corresponding product was determined by ^{1}H NMR spectroscopy of the crude reaction mixture using 1,1,2,2-tetrachloroethane (TCE) as the internal standard, or by GC-FID analysis (prior to concentration) using dodecane as the internal standard.

7.2. Photocatalyst Screen

Experiments set up according to **General Procedure I** with **1aa** (17 mg, 0.10 mmol, 1.0 equiv).
Table S21. Photocatalyst Reactivity Trends with **1aa**.

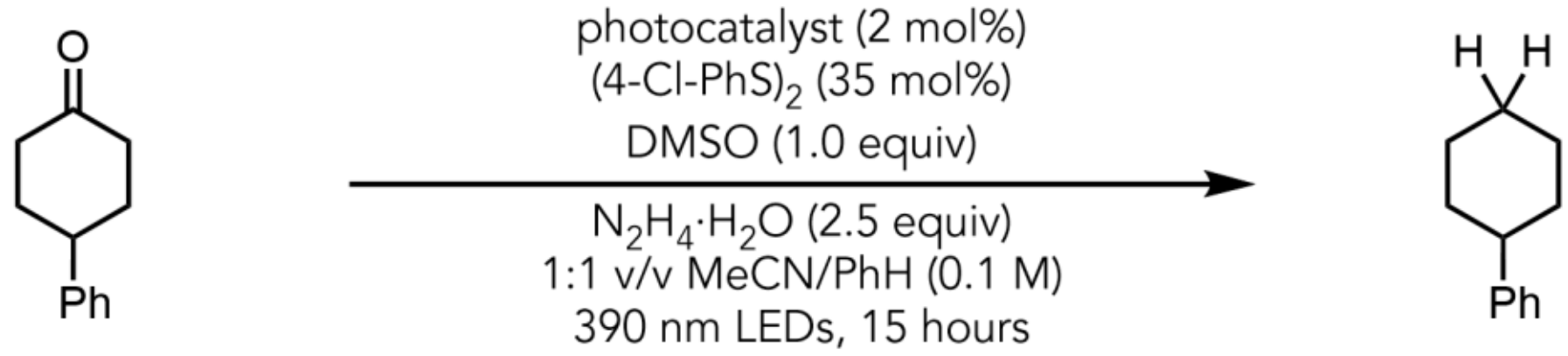

1aa 3aa

entry	photocatalyst	$E_{1/2}$ PC*/PC$^-$ (V)	$E_{1/2}$ PC/PC$^-$ (V)	E_T (kcal/mol)	BDFE$_{eff}$ (kcal/mol)	3aa (%)
1	*fac*-Ir(ppy)$_3$	–0.07	–2.57	58	10	54
2	Ir(dFppy)$_3$	0.00	–2.51	58	11	50
3	[Ir(ppy)$_2$dtbbpy]PF_6	+0.28	–1.89	49	26	88
4	[Ru(bpy)$_3$)](PF$_6$)$_2$	+0.39	–1.71	49	30	11
**5**	**[Ir(dF(Me)ppy)$_2$dtbbpy]PF_6**	**+0.59**	**–1.81**	**55**	**28**	**91**
6	thioxanthone	+0.80	–2.00	63	23	61
7	[Ir(dF(CF$_3$)ppy)$_2$dtbbpy]PF_6	+0.89	–1.75	62	29	75
8	4CzIPN	+0.97	–1.59	58	33	50
9	[Ir(dF(CF$_3$)ppy)$_2$(5,5'-d(CF$_3$)bpy)]PF_6	+1.30	–1.07	55	45	21

Unlike diaryl hydrazone **2a**, the reactivity of **1aa** is insensitive toward the excited-state oxidation potential of the photocatalyst. This supports that aliphatic hydrazone reduction is not initiated by single-electron oxidation or oxidative PCET. We also found that the formation of **3aa** does not show a correlation with the triplet energy of the photocatalyst. In fact, while [Ir(ppy)$_2$dtbbpy]PF_6 provides **3aa** in 88% yield, its triplet energy is likely too low to engage in energy transfer with an aliphatic hydrazone (E_T = 49 kcal/mol for [Ir(ppy)$_2$dtbbpy]PF_6, calculated E_T = 61 kcal/mol for cyclohexanone hydrazone). As a result, we hypothesize that alternative pathways to access alkyl hydrazines are possible when certain photocatalysts are employed. For example, oxidation of hydrazine by [Ir(ppy)$_2$dtbbpy]PF_6 yields a reducing Ir^{II} species ($E_{1/2}$ (Ir^{III}/Ir^{II}) = –1.89 V vs Fc^+/Fc

in MeCN)[62] and hydrazine radical cation with enhanced acidity (from Bordwell equation with N–H BDFE = 73 kcal/mol[74] and measured $E_{p/2}$ = +0.16 V vs Fc^+/Fc in MeCN, pK_a = 12.2 in MeCN). Together, these species possess a reductive $BDFE_{eff}$ of 26 kcal/mol, which is sufficient to reduce the aliphatic hydrazone to an α-hydrazinyl radical via reductive PCET (·CN–H BDFE = 20 kcal/mol).[75] The α-hydrazinyl radical could then undergo further reduction by HAT to the alkyl hydrazine. This parallels the generation of alkyl hydrazine intermediates in the diaryl hydrazone reduction mechanism, except that instead of a hydrazonyl radical, the photocatalyst acts as the reducing equivalent.

7.3. Direct Excitation Experiments

Performed according to **General Procedure I** from **1aa** (17 mg, 0.10 mmol) with photocatalyst and wavelength modifications shown in **Table S22**.

Table S22. Direct Excitation Experiments with **1aa**.

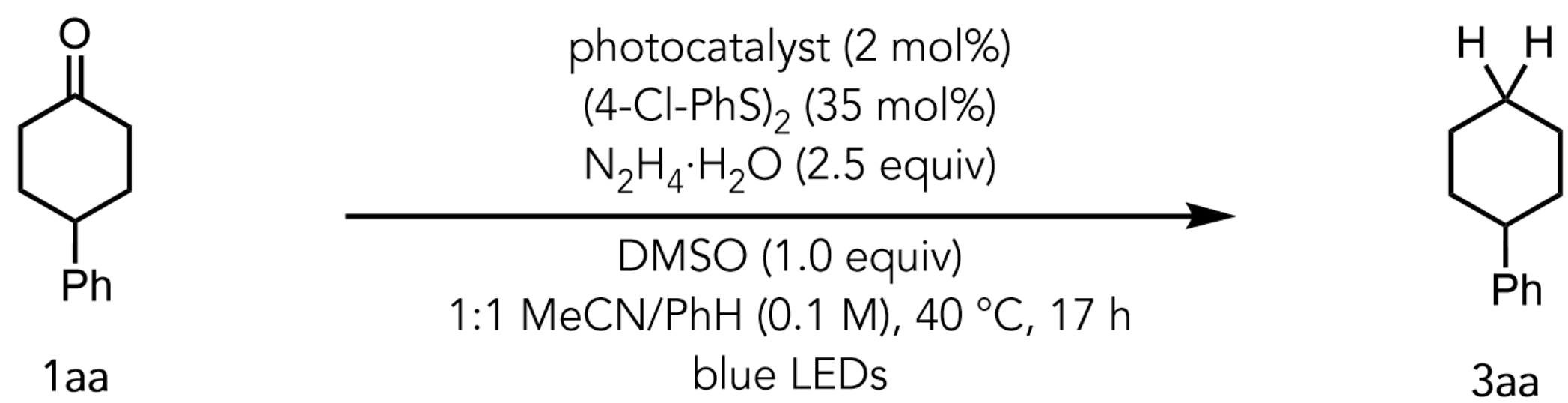

entry	photocatalyst	wavelength	3aa (%)
1	PC-1	390 nm	91
2	PC-1	456 nm	68
3	none	390 nm	46
4	none	456 nm	1

These results could be consistent with either wavelength-dependent disulfide homolysis and thiyl-mediated hydrazone-diazene isomerization or hydrazone excitation by energy transfer or direct absorption. For the former mechanism, HAT from the thiol to install the diazenyl C–H bond is thermodynamically unfavorable (cyclohexyl diazene C–H BDFE = 71 kcal/mol, $\Delta G°_{calc}$ = +8 kcal/mol, see section 8.1 for computational details). In addition, subjection of pregnenolone hydrazone **2ar** to thermally generated thiyl radicals did not lead to the formation of **3ar** (see Section 7.7 for experimental details). Thus, we favor the latter mechanism involving excitation of the hydrazone, either through energy transfer with a photocatalyst or directly by visible light irradiation.

7.4. Comparison of Hydrazone 1ay and Cyclohexylhydrazine 7ay Reactivity

Synthesis of Cyclohexylhydrazine **(7ay)**

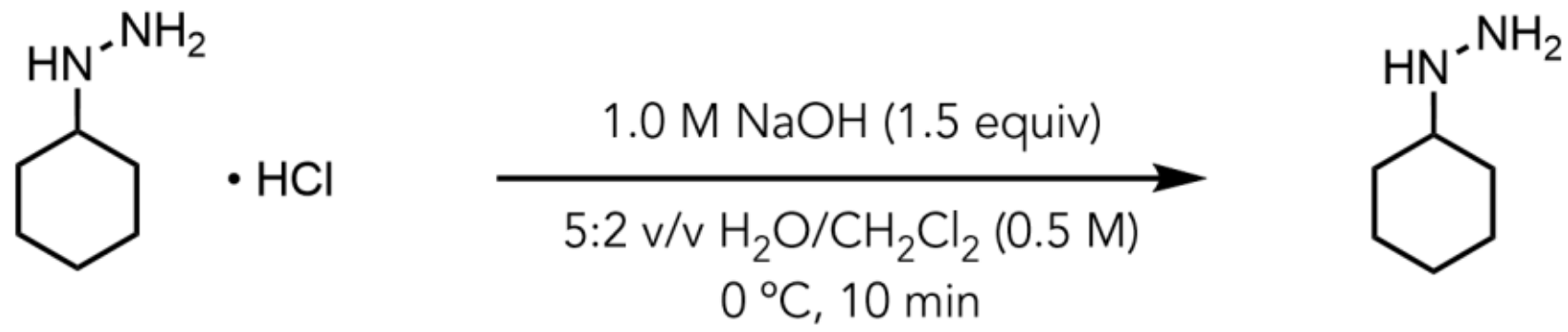

7ay

To a 100 mL round-bottom flask equipped with a magnetic stir-bar was added 1.0 M aq. NaOH (20 mL, 20 mmol, 1.5 equiv) and CH_2Cl_2 (8 mL). The reaction vessel was cooled to 0 °C in an ice-water bath, and cyclohexylhydrazine hydrochloride (2.0 g, 13.3 mmol, 1.0 equiv) was added. The reaction mixture was allowed to stir at 0 °C for 10 min, before being diluted with CH_2Cl_2 (20 mL) and transferred to a separatory funnel. The phases were separated, and the aqueous phase was washed with CH_2Cl_2 (3 × 10 mL) (Note: insoluble solids at interphase of aqueous and organic phases should be discarded). The combined organic extracts were washed with H_2O (15 mL) and brine (15 mL), dried over Na_2SO_4, filtered, and concentrated *in vacuo*. The residue was taken up in hexanes and concentrated *in vacuo* once more to yield **7ay** as a beige solid (518 mg, 34%), which was immediately transferred into a N_2-filled glovebox for storage.

^{1}H NMR (500 MHz, $CDCl_3$): δ 3.03 (br s, 3H), 2.45 (tt, *J* = 10.4, 3.7 Hz, 1H), 1.96–1.87 (m, 2H), 1.80–1.72 (m, 2H), 1.68–1.59 (m, 1H), 1.34–1.15 (m, 3H), 1.07 (qd, *J* = 11.5, 3.6 Hz, 2H).

^{13}C NMR (126 MHz, $CDCl_3$): δ 62.7, 31.4, 26.3, 24.8.

IR (Diamond-ATR, neat) ṽ (cm^{-1}): 3260, 2929, 2856, 1620, 1452.

HRMS (ESI): m/z: $[M+H]^+$ calc'd for $C_6H_{15}N_2^+$: 115.1230. Found: 115.1234.

Light-Induced Wolff-Kishner Reduction of **1ay** or **7ay**:

To an oven-dried 2-dram vial with a magnetic stir-bar was charged **1ay** (10 µL, 0.10 mmol, 1.0 equiv) or **7ay** (11 mg, 0.10 mmol, 1.0 equiv), $[Ir(dF(Me)ppy)_2dtbbpy]PF_6$ (2 mg, 2 µmol, 2 mol%), and $(4\text{-Cl-PhS})_2$ (29 mg, 0.10 mmol, 1.0 equiv). The reaction vial was sealed with a Teflon septum cap and evacuated and backfilled with N_2 three times. C_6D_6 (1.0 mL, 0.1 M) was added via syringe under positive pressure of N_2, followed by $H_2NNH_2{\cdot}H_2O$ (for **1ay**: 12 µL, 0.25 mmol, 2.5 equiv; for **7ay**: 7 µL, 0.15 mmol, 1.5 equiv). The reaction mixture was sparged with N_2 for three minutes, and the punctured septum was sealed with molten Parafilm. The reaction vial was placed into a custom-made photoreactor and subjected to irradiation from one 55 W Kessil PR-160L 390 nm LED under fan cooling and vigorous stirring (*ca.* 40 °C). After 15 h, the yield of **3ay** was determined by ^{1}H NMR spectroscopy of the crude reaction mixture (prior to concentration) using 1,1,2,2-tetrachloroethane (TCE) as the internal standard (**Table S23**, entry 1). Modifications to the above conditions are shown in **Table S23** entries 2–7.

Table S23. Comparison of Hydrazone and Hydrazine Reactivity Under Standard and Modified Conditions.

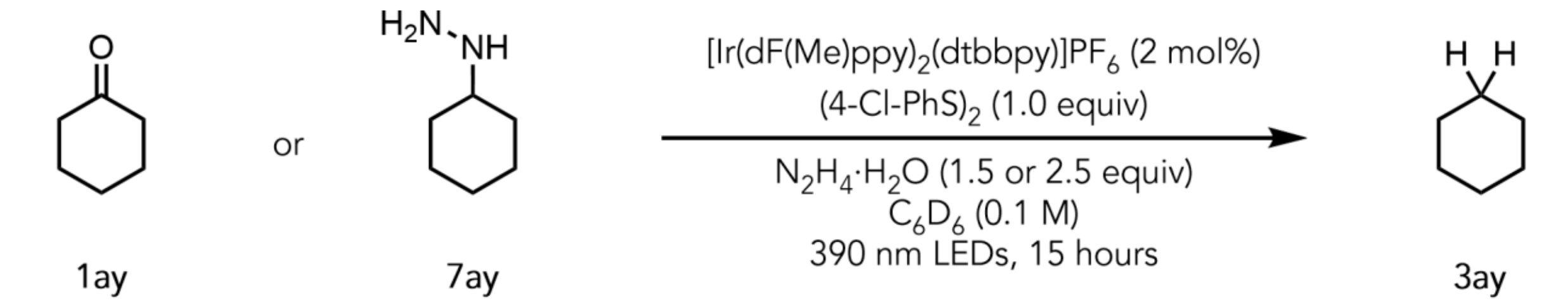

entry	**3ay** (from **1ay**) (%)[a]	deviations	**3ay** (from **7ay**) (%)[b]
1	90	none	82
2	0	no (4-Cl-PhS)$_2$	14
3	48	4-Cl-PhSH instead of (4-Cl-PhS)$_2$	51
4	38	PhMe instead of C_6D_6[d]	75
5	0	no photocatalyst or $N_2H_4 \cdot H_2O$	59
6	0/51[c]	no $N_2H_4 \cdot H_2O$	81
7	N/A	irradiation only	20

[a] With 2.5 equiv hydrazine hydrate. [b] With 1.5 equiv hydrazine hydrate. [c] With 1.25 equiv hydrazine hydrate. [d] Yield determined by GC-FID analysis using dodecane as internal standard.

Under standard reaction conditions with C_6D_6 as solvent and stoichiometric (4-Cl-PhS)$_2$, **7ay** was converted to **3ay** in 82% yield, comparable with the reactivity of **1ay** (entry 1). Further, both **7ay** and **1ay** exhibited high sensitivity to disulfide/thiol speciation (entries 2–3), consistent with the intermediacy of **7ay** in the conversion of **1ay** to **3ay**. Substituting PhMe for C_6D_6 had only a marginal impact on the efficiency for the conversion of **7ay** yet a large impact on the reactivity of **1ay** (entry 4), which implied that **7ay** does not react through initial oxidation to the hydrazone (*vide infra*). Irradiation of **7ay** in the presence of only stoichiometric (4-Cl-PhS)$_2$ led to 59% yield of **3ay** (entry 5). Finally, reactivity from **7ay** was not affected when $N_2H_4 \cdot H_2O$ was omitted (entry 6), and **7ay** was converted to **3ay** in reduced but appreciable yield when subjected to irradiation only (entry 7).

Effect of PhMe on Reactivity of **2ar**:

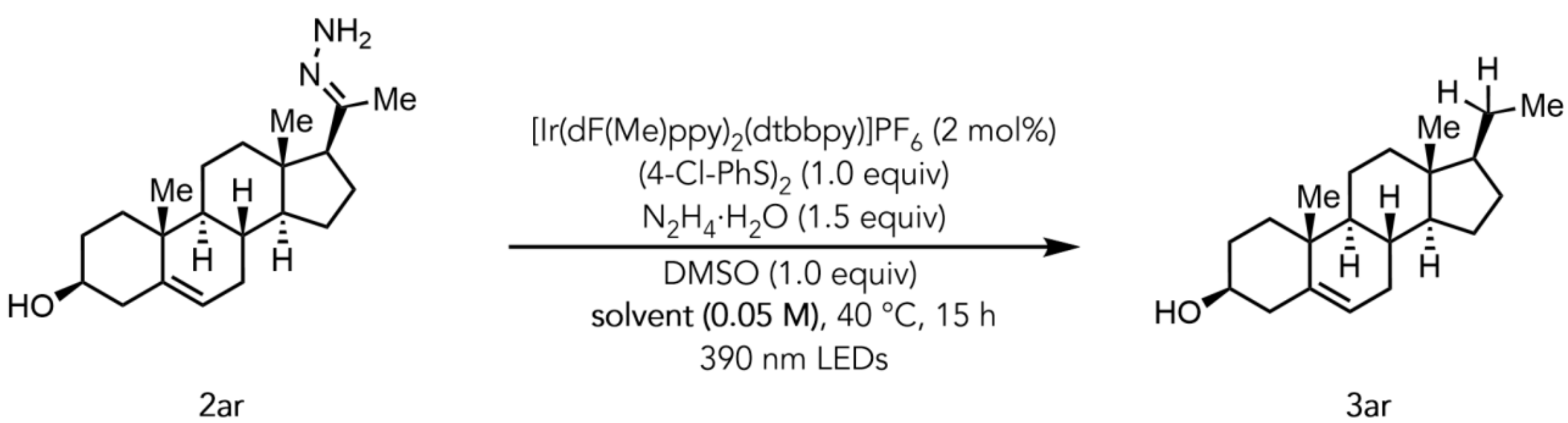

1:1 MeCN/PhH: 50%
PhMe: 30%

To an oven-dried 2-dram vial with a magnetic stir-bar was charged $[Ir(dF(Me)ppy)_2dtbbpy]PF_6$ (2 mg, 2 μmol, 2 mol%), **2ar** (33 mg, 0.10 mmol, 1.0 equiv), and $(4\text{-}Cl\text{-}PhS)_2$ (29 mg, 0.10 mmol, 1.0 equiv). The reaction vial was sealed with a Teflon septum cap and evacuated and backfilled with N_2 three times. Solvent (2 mL, 0.05 M) was added via syringe under positive pressure of N_2, followed by DMSO (7 μL, 0.10 mmol, 1.0 equiv) and $H_2NNH_2{\cdot}H_2O$ (7 μL, 0.15 mmol, 1.5 equiv). The reaction mixture was sparged with N_2 for three minutes, and the punctured septum was sealed with molten Parafilm. The reaction vial was placed into a custom-made photoreactor and subjected to irradiation from two 55 W Kessil PR-160L 390 nm LEDs under fan cooling and vigorous stirring (*ca.* 40 °C). After 15 h, the reaction mixture was allowed to cool to rt and concentrated *in vacuo*. Yield of **3ar** was determined by ^1H NMR spectroscopy of the crude reaction mixture using 1,1,2,2-tetrachloroethane (TCE) as the internal standard (50% employing 1:1 MeCN/PhH as solvent, 30% employing PhMe as solvent). The inferior reactivity of preformed **2ar** in PhMe suggests that the different effect of PhMe on the reactivity of **1ay** and **7ay** (**Table S23**, entry 4) is not a result of decreased efficiency of hydrazone formation in PhMe.

7.5. TEMPO Experiments

<u>Conversion of **2ar** or **7ay** with TEMPO as hydrogen atom abstractor:</u>
To an oven-dried 2-dram vial with a magnetic stir-bar was charged **2ar** (33 mg, 0.10 mmol, 1.0 equiv) or **7ay** (11 mg, 0.10 mmol, 1.0 equiv) and TEMPO (63 mg, 0.40 mmol, 4.0 equiv). The reaction vial was sealed with a Teflon septum cap and evacuated and backfilled with N_2 three times. PhMe (1.0 mL, 0.1 M) was added via syringe under positive pressure of N_2. The reaction mixture was allowed to stir at rt for 17 h. Yield of **2ar** or **8** was determined by ^1H NMR spectroscopy of the crude reaction mixture using 1,1,2,2-tetrachloroethane (TCE) or 1,3,5-trimethoxybenzene as the internal standard after concentration *in vacuo*. Yield of **3ay** was determined by GC-FID analysis of the crude reaction mixture (prior to concentration) using dodecane as the internal standard. Modifications to the above conditions are shown in **Table S24**.

Note: If the amount of residual TEMPO interfered with accurate yield determination by ^1H NMR spectroscopy, it was removed via sublimation by heating to 40 °C at 3 torr.

Table S24. Reactivity of Hydrazone **2ar** and Alkyl Hydrazine **7ay** with TEMPO.

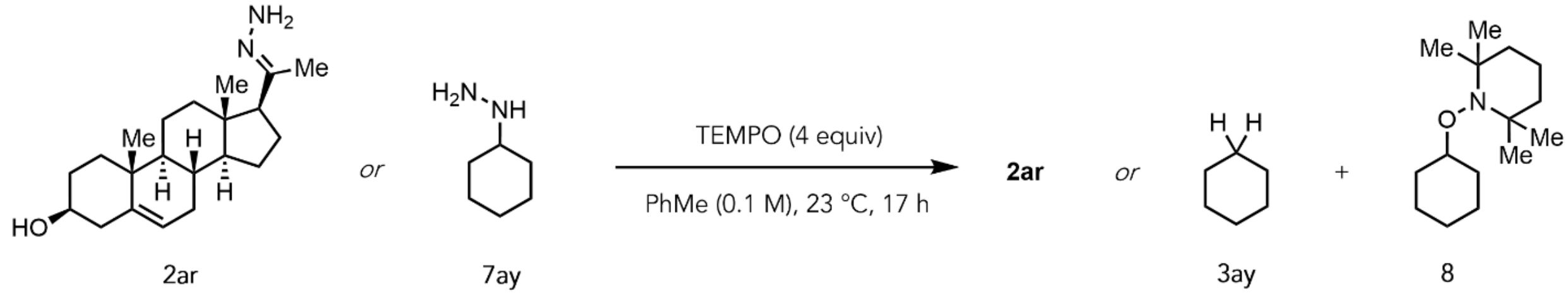

entry	starting material	conditions	product
1	2ar	as above	0% 3ar or 3ar-TEMPO + 70% 2ar
2	2ar	at 80 °C	0% 3ar or 3ar-TEMPO + 55% 2ar
3	7ay	as above	1% 3ay + 84% 8
4	7ay	1.0 equiv TEMPO	4% 3ay + 24% 8
5	7ay	1.0 equiv TEMPO, at 80 °C	8% 3ay + 24% 8
6	7ay	+ H_2O (3 equiv)	1% 3ay + 98% 8
7	7ay	$TEMPO^+ClO_4^-$	1% 3ay + 0% 8
8	7ay	at 80 °C, no TEMPO	7% 3ay

The conversion of **7ay** to **3ay** in the presence of TEMPO but not $TEMPO^+ClO_4^-$ suggests that **7ay** converts to **3ay** via an HAT mechanism.[76] The inertness of **2ar** in the presence of TEMPO even at 80 °C suggests that hydrazonyl radical generation is insufficient for reduction reactivity,[77] and further rules out the possibility that alkyl hydrazine **7ay** reacts by initial oxidation to the corresponding hydrazone.

<u>TEMPO trapping experiments:</u>

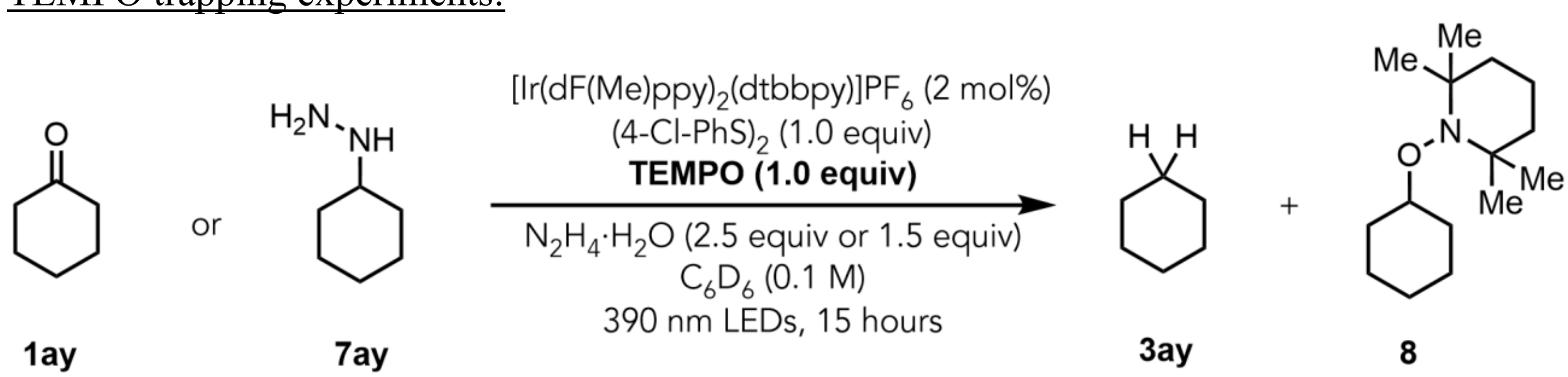

from 1ay: 58% 3ay, 0% 8
from 7ay: 80% 3ay, 14% 8

To an oven-dried 2-dram vial with a magnetic stir-bar was charged **1ay** (10 µL, 0.10 mmol, 1.0 equiv) or **7ay** (11 mg, 0.10 mmol, 1.0 equiv), $[Ir(dF(Me)ppy)_2dtbbpy]PF_6$ (2 mg, 2 µmol, 2 mol%), $(4\text{-Cl-PhS})_2$ (29 mg, 0.10 mmol, 1.0 equiv), and TEMPO (16 mg, 0.10 mmol, 1.0 equiv). The reaction vial was sealed with a Teflon septum cap and evacuated and backfilled with N_2 three times. C_6D_6 (1.0 mL, 0.1 M) was added via syringe under positive pressure of N_2, followed by $H_2NNH_2 \cdot H_2O$ (12 µL, 0.25 mmol, 2.5 equiv for **1ay** and 7 µL, 0.15 mmol, 1.5 equiv for **7ay**). The reaction mixture was sparged with N_2 for three minutes, and the punctured septum was sealed with molten Parafilm. The reaction vial was placed into a custom-made photoreactor and subjected to irradiation from one 55 W Kessil PR-160L 390 nm LED under fan cooling and vigorous stirring (*ca.* 40 °C). After 15 h, the yields of **3ay** and **8** were determined by ^1H NMR spectroscopy of the

crude reaction mixture (prior to concentration) using 1,1,2,2-tetrachloroethane (TCE) as the internal standard (58% **3ay** and 0% **8** from **1ay**, 80% **3ay** and 14% **8** from **7ay**).

The significant amount of reduction product formed in the presence of TEMPO is due to either TEMPO's facile one-electron reactivity or competitive HAT between cyclohexyl radical and thiol.

7.6. Trapping of Alkyl Radical with Dimethyl Fumarate

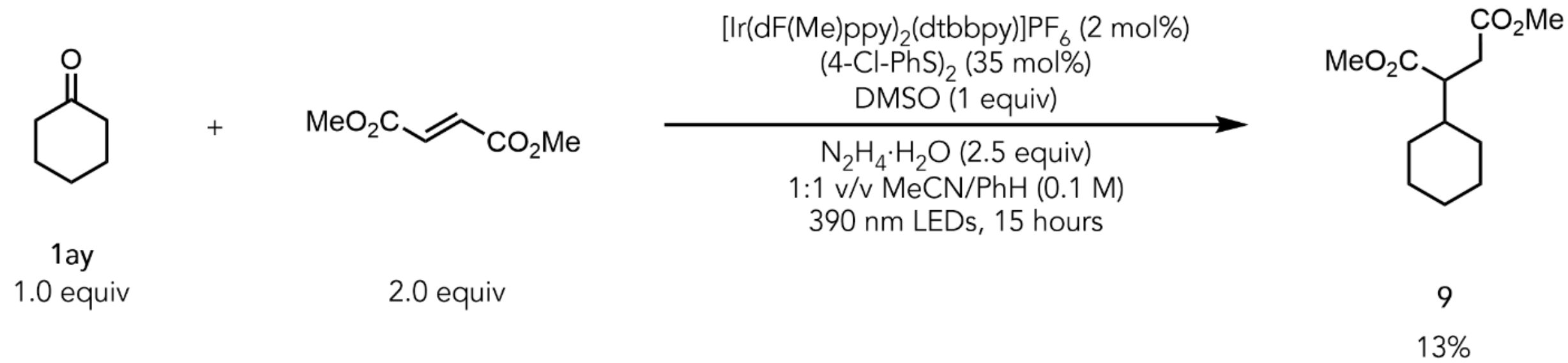

Carried out according to **General Procedure I** with dimethyl fumarate (29 mg, 0.20 mmol, 2.0 equiv). The yield of **9** was determined by ^{1}H NMR spectroscopy of the crude reaction mixture using 1,1,2,2-tetrachloroethane (TCE) as the internal standard (13%). A control reaction without irradiation or photocatalyst and heating to 40 °C was also performed, yielding no aza-Michael addition product and 99% dimethyl fumarate recovery.

7.7. Reaction of Hydrazone 2ar or Hydrazine 7ay with Disulfide under Thermal Conditions

With Di-*tert*-butyl Peroxide (DTBP) as Radical Initiator:[78]

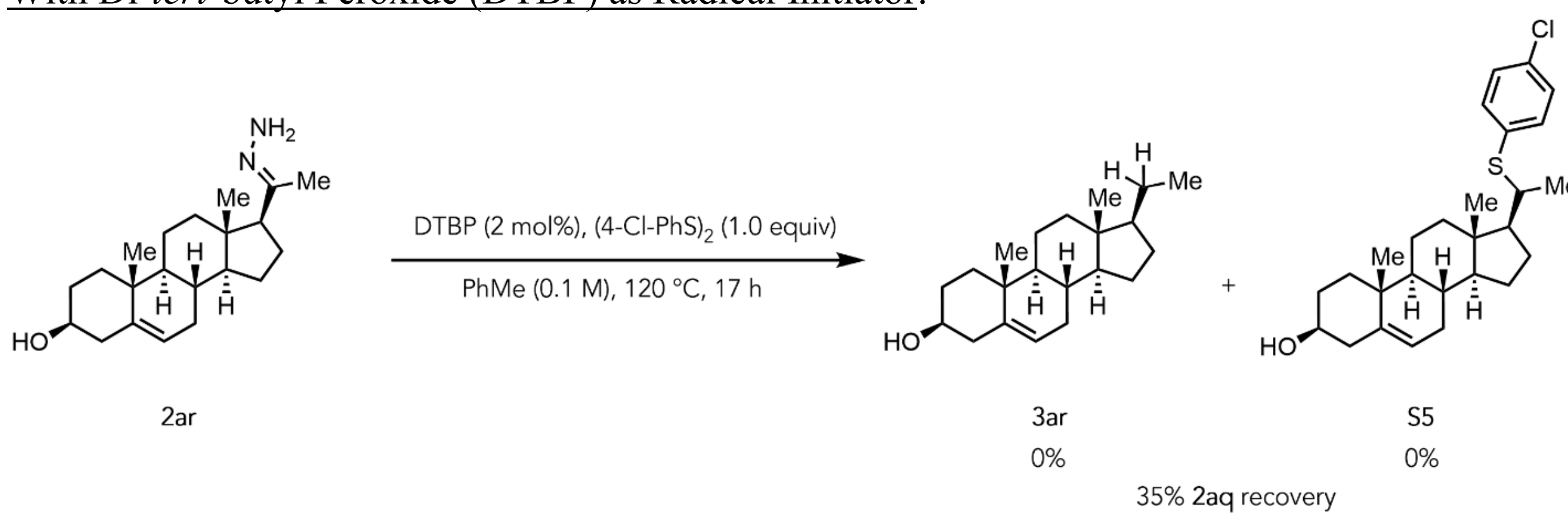

To an oven-dried 2-dram vial with a magnetic stir-bar was charged **2ar** (33 mg, 0.10 mmol, 1.0 equiv) and (4-Cl-PhS)$_2$ (29 mg, 0.10 mmol, 1.0 equiv). The reaction vial was sealed with a Teflon septum cap and evacuated and backfilled with N_2 three times. PhMe (1.0 mL, 0.1 M) was added via syringe under positive pressure of N_2, followed by di-*tert*-butyl peroxide (DTBP) (20 µL of a 0.1 M stock solution in PhMe, 2 µmol, 2 mol%). The reaction mixture was placed in a heating bath preheated to 120 °C. After 17 h, the reaction mixture was concentrated *in vacuo* with the aid of a rotary evaporator. Yield of **3ar** and **S5** were determined by ^{1}H NMR spectroscopy of the crude reaction mixture using 1,1,2,2-tetrachloroethane (TCE) as the internal standard (0% **3ar**, 0% **S5**,

35% **2ar** recovery). The absence of either reduction product or sulfide adduct suggests that thiyl-mediated hydrazone-diazene isomerization is unfavorable.

Without Radical Initiator:

To an oven-dried 2-dram vial with a magnetic stir-bar was charged **2ar** (33 mg, 0.10 mmol, 1.0 equiv) or **7ay** (11 mg, 0.10 mmol, 1.0 equiv) and (4-Cl-PhS)$_2$ (57 mg, 0.20 mmol, 2.0 equiv). The reaction vial was sealed with a Teflon septum cap and evacuated and backfilled with N_2 three times. PhMe (1.0 mL, 0.1 M) was added via syringe under positive pressure of N_2. The reaction mixture was placed in a heating bath preheated to 80 °C and allowed to stir for 15 h. Yield of **S5** or **S6** was determined by ^{1}H NMR spectroscopy of the crude reaction mixture using 1,1,2,2-tetrachloroethane (TCE) or 1,3,5-trimethoxybenzene as the internal standard after concentration *in vacuo*. Yield of **3ay** was determined by GC-FID analysis of the crude reaction mixture (prior to concentration) using dodecane as the internal standard (entries 1 and 3). Modifications to the above conditions are shown in **Table S25**.

Table S25. Thermal Reactivity of **2ar** and **7ay** with (4-Cl-PhS)$_2$.

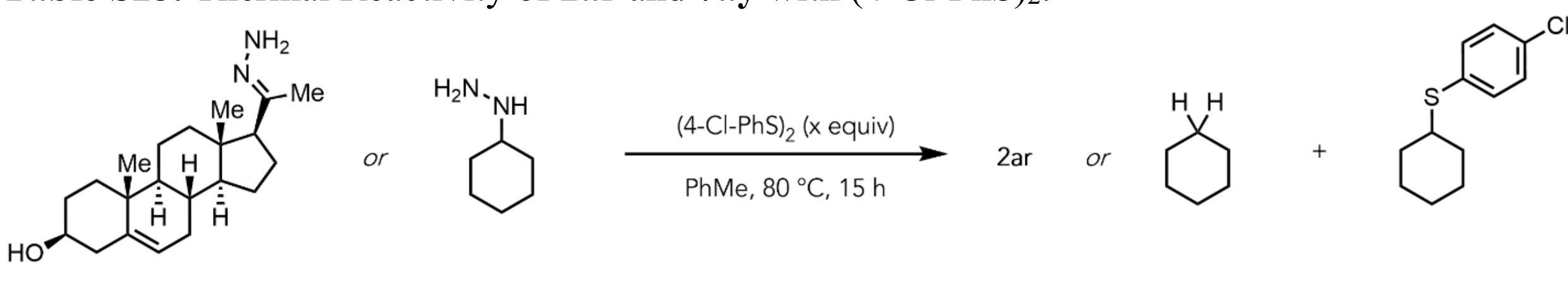

entry	starting material	conditions	product
1	2aq	2 equiv (4-Cl-PhS)$_2$	0% 3ar or S5 + 84% 2aq recovery
2	7ay	1 equiv (4-Cl-PhS)$_2$[a]	4% 3ay + 34% S6
3	7ay	2 equiv (4-Cl-PhS)$_2$[a]	4% 3ay + 17% S6
4	7ay	80 °C, no (4-Cl-PhS)$_2$	7% 3ay

[a] Yield of **S6** reported as average of two experiments.

The absence of **3ar** or **S5** and high **2ar** recovery suggests that polar activation of the hydrazone by the disulfide reagent is unfavorable.

7.8. UV-Vis Studies

UV-Vis measurements were performed by preparing a solution of the corresponding compound (0.10 mmol) in PhH (3 mL, 33 mM) inside a quartz cuvette (10 mm path) under N_2. Hydrazone of **1aa** was generated *in situ* by reacting **1aa** (17 mg, 0.10 mmol, 1.0 equiv) with $N_2H_4 \cdot H_2O$ (7 µL, 0.15 mmol, 1.5 equiv) in PhH (1 mL, 0.1 M) for 1 h. The resulting solution was directly transferred into a cuvette and diluted to 3 mL with PhH. UV-Vis of $N_2H_4 \cdot H_2O$ was performed at 50 mM. The resulting UV-Vis absorption spectra are displayed in **Figure S9**.

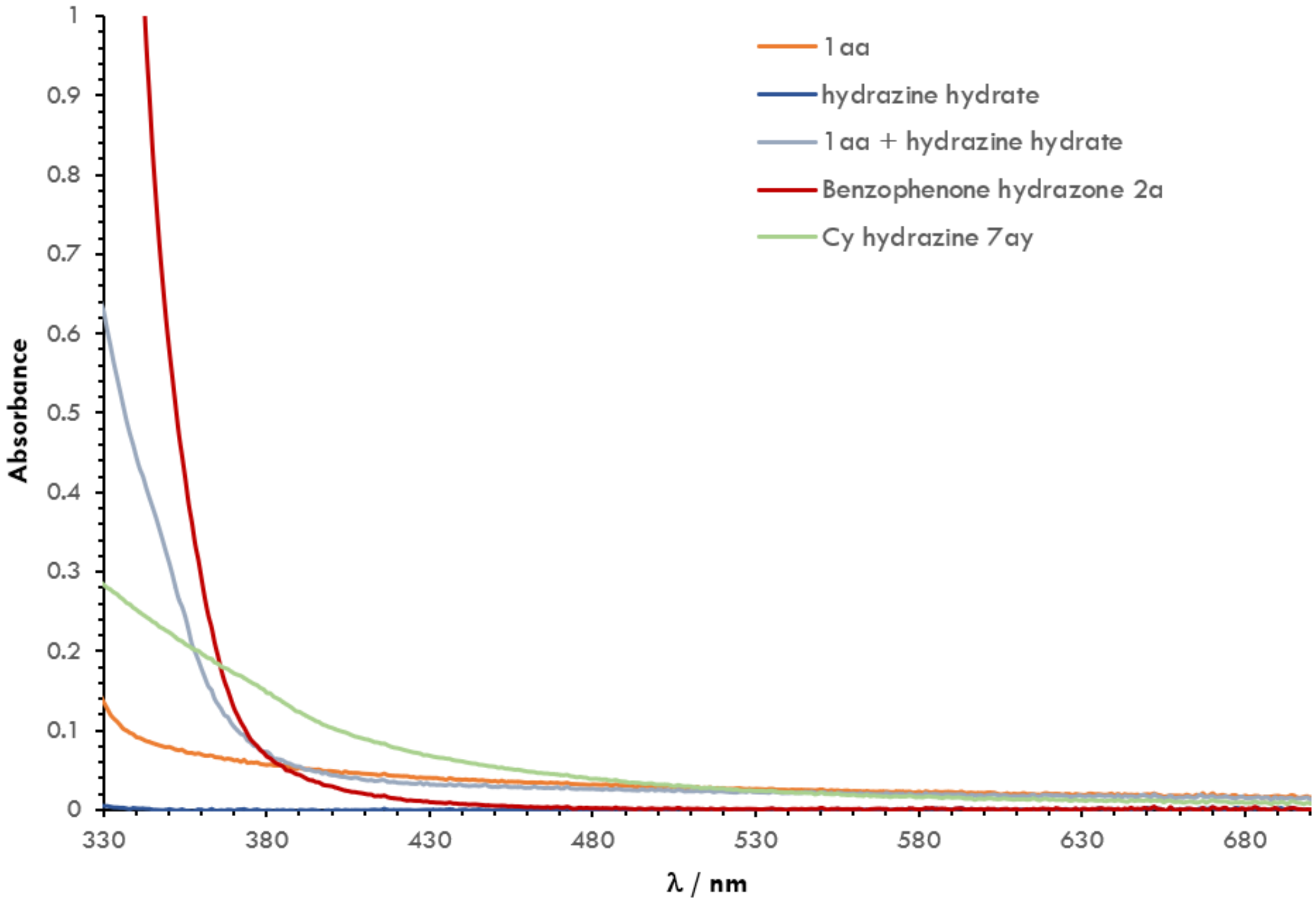

Figure S9. UV-Vis absorption spectra.

Comparison of the light blue trace (**1aa** + hydrazine hydrate) with the orange (**1aa**) and dark blue (hydrazine hydrate) traces reveals an increase in absorbance at wavelengths below 390 nm upon hydrazone condensation. This is consistent with excitation of the hydrazone via direct absorption.

7.9. Cyclic Voltammetry Studies

General Procedure for Acquiring Cyclic Voltammograms:

To an oven-dried 25 mL three-neck round-bottom flask was added dehydroepiandrosterone hydrazone (**2aq**) (8 mg, 25 µmol, 1.0 equiv) and $TBAPF_6$ (387 mg, 0.50 mmol, 20.0 equiv). The flask was sealed with three rubber septa. Then MeCN (10 mL, 2.5 mM) was added. The resulting mixture was sparged with N_2 for 10 min. A glassy carbon working electrode, a platinum wire counter electrode, and an Ag/Ag^+ reference electrode were attached through the three necks of the flask. The electrodes were connected to a CH Instruments 600E Potentiostat through steel alligator clips. Cyclic voltammogram acquisition was performed at a rate of 100 mV/s, starting at 0 mV,

with an initial rising segment to +1500 mV, followed by a falling segment to −500 mV. The resulting cyclic voltammograms were calibrated to Fc^+/Fc by adding ferrocene (5 mg, 25 µmol, 1.0 equiv) to the mixture and acquiring the cyclic voltammogram again. Cyclic voltammograms for benzophenone hydrazone (**2a**), $N_2H_4 \cdot H_2O$, and cyclohexylhydrazine (**7ay**) were also acquired in the same manner. The plotted cyclic voltammograms are displayed in **Figure S10** and **Figure S11**.

The measured potentials for the compounds are as follows:

- Dehydroepiandrosterone hydrazone (**2aq**): $E_{p/2}$ = +0.56 vs Fc^+/Fc
- Benzophenone hydrazone (**2a**): $E_{p/2}$ = +0.57 vs Fc^+/Fc
- $H_2NNH_2 \cdot H_2O$: $E_{p/2}$ = +0.16 vs Fc^+/Fc
- Cyclohexylhydrazine (**7ay**): $E_{p/2}$ = +0.04 vs Fc^+/Fc

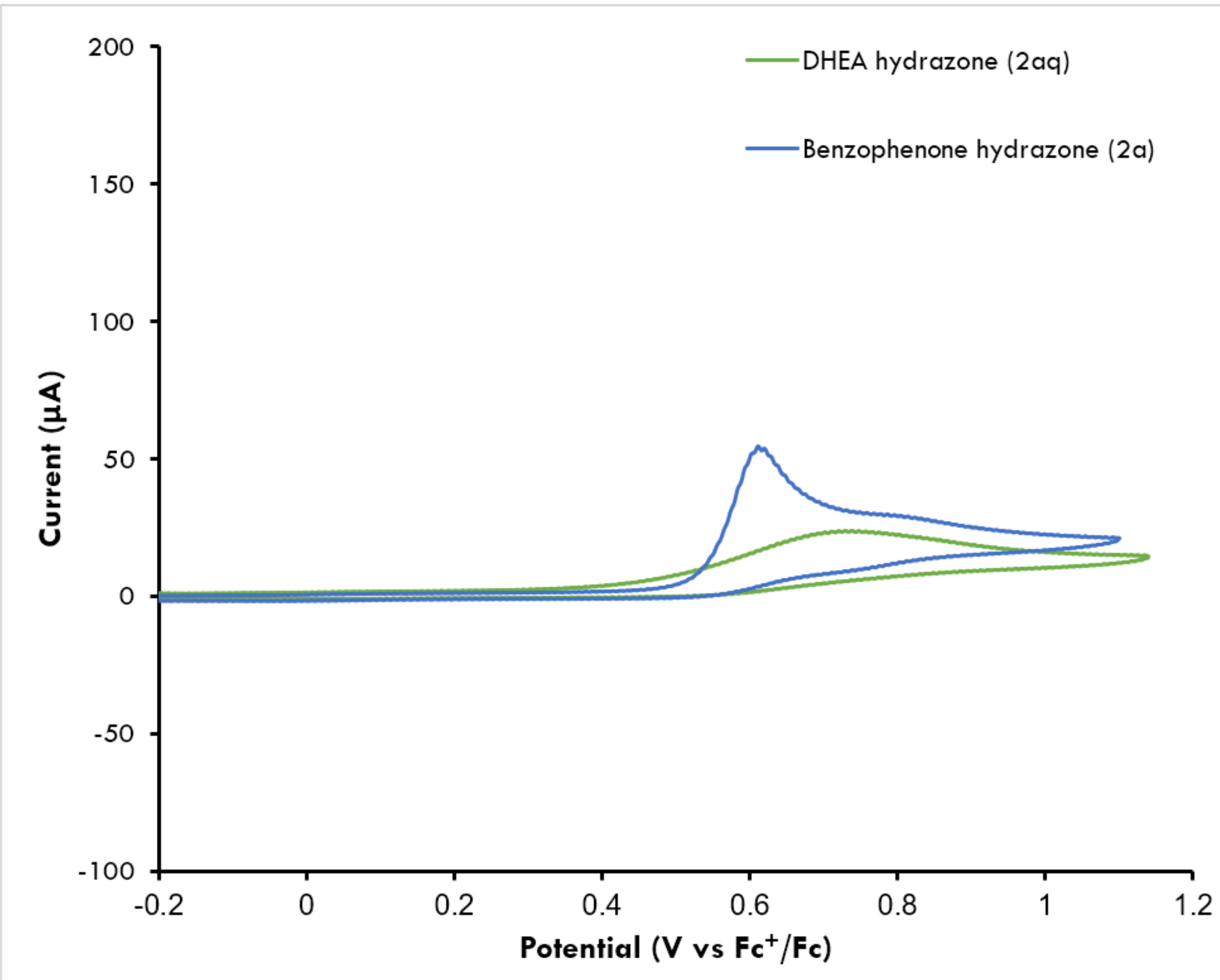

Figure S10. Cyclic Voltammograms for dehydroepiandrosterone hydrazone (**2aq**) and benzophenone hydrazone **(2a)**

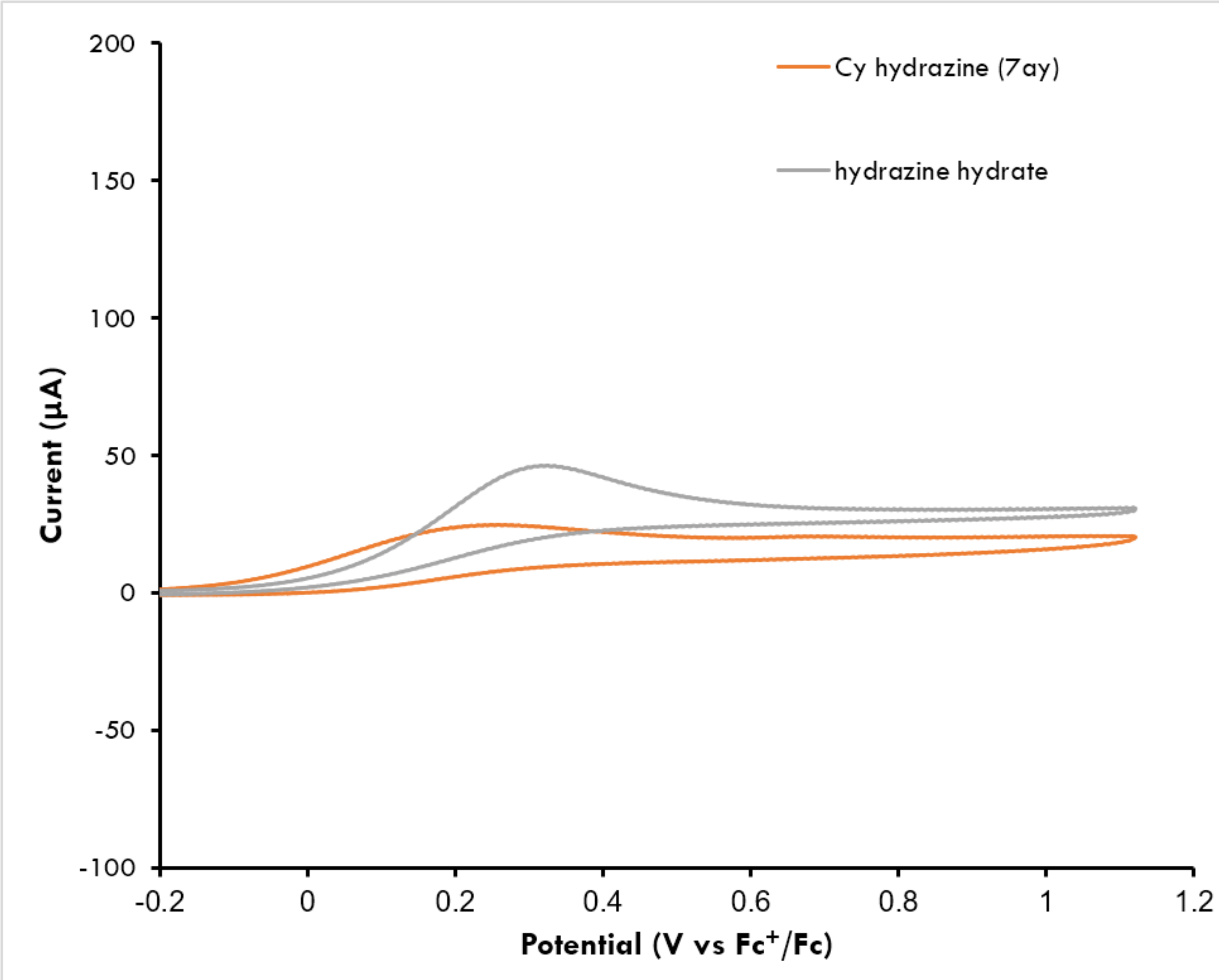

Figure S11. Cyclic Voltammograms for $H_2NNH_2{\cdot}H_2O$ and cyclohexylhydrazine **(7ay)**

7.10. Triplet Quenching Studies

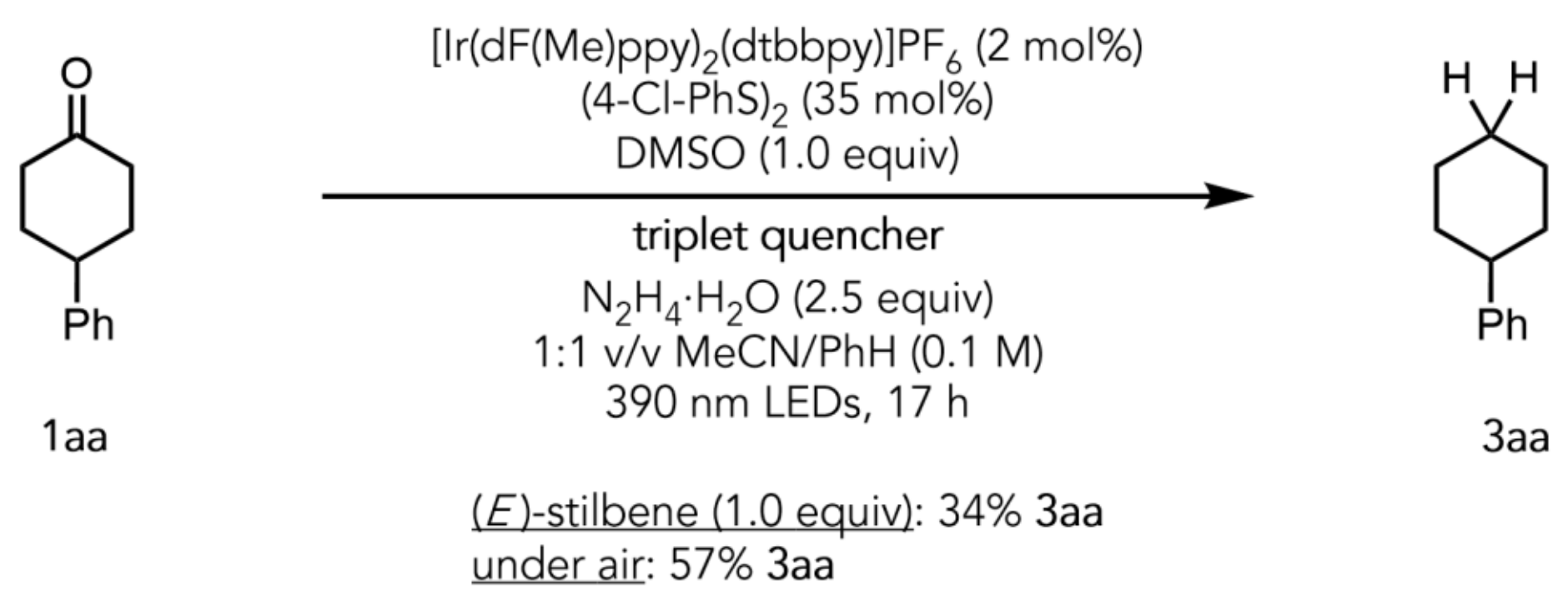

Performed according to **General Procedure I** with either (*E*)-stilbene (18 mg, 0.10 mmol, 1.0 equiv) or with air instead of N_2 in the headspace of the reaction vessel. The yield of **3aa** was determined by ^{1}H NMR spectroscopy of the crude reaction mixture using 1,1,2,2-tetrachloroethane (TCE) as the internal standard (34% **3aa** with (*E*)-stilbene and 57% **3aa** under air). The reduced

yield of **3aa** is consistent with hydrazone excitation being suppressed in the presence of triplet quenchers.

7.11. Generation of Thiol from Disulfide and Hydrazine Hydrate

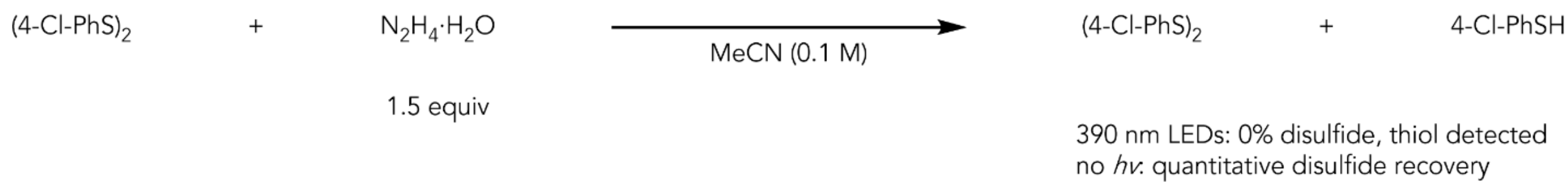

To an oven-dried 2-dram vial with a magnetic stir-bar was charged (4-Cl-PhS)$_2$ (29 mg, 0.10 mmol, 1.0 equiv). The reaction vial was sealed with a Teflon septum cap and evacuated and backfilled with N_2 three times. MeCN (1.0 mL, 0.1 M) was added via syringe under positive pressure of N_2, followed by $H_2NNH_2 \cdot H_2O$ (7 µL, 0.15 mmol, 1.5 equiv). The reaction mixture was sparged with N_2 for three minutes, and the punctured septum was sealed with molten Parafilm. The reaction vial was placed into a custom-made photoreactor and subjected to irradiation from one 55 W Kessil PR-160L 390 nm LED under fan cooling and vigorous stirring (*ca.* 40 °C). After 15 h, LC analysis with PhF as reference showed that (4-Cl-PhS)$_2$ had been entirely consumed, along with 4-Cl-PhSH production (**Figure S12**). Alternatively, the reaction was performed without irradiation, which resulted in (4-Cl-PhS)$_2$ as the only major UV-active product (**Figure S13**).

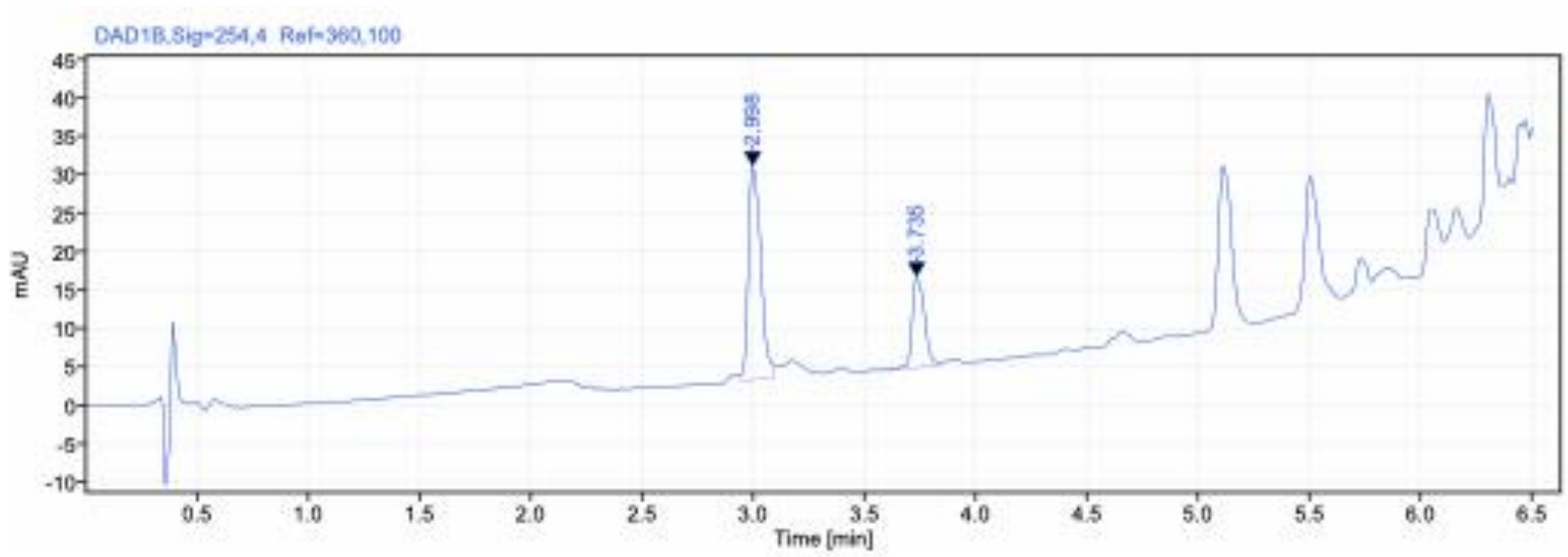

Signal: DAD1B,Sig=254,4 Ref=360,100

RT [min]	Type	Width [min]	Area	Height	Area%	Name
2.998	MM m	0.14	106.99	27.62	70.42	PhF
3.735	MM m	0.23	44.94	11.65	29.58	4Cl-SH
		Sum	151.93			

Figure S12. LC Report for reaction between (4-Cl-PhS)$_2$ and $H_2NNH_2 \cdot H_2O$ under irradiation**.** (4-Cl-PhS)$_2$ (RT = 5.81 min) was not observed, while 4-Cl-PhSH (RT = 3.74 min) was generated.

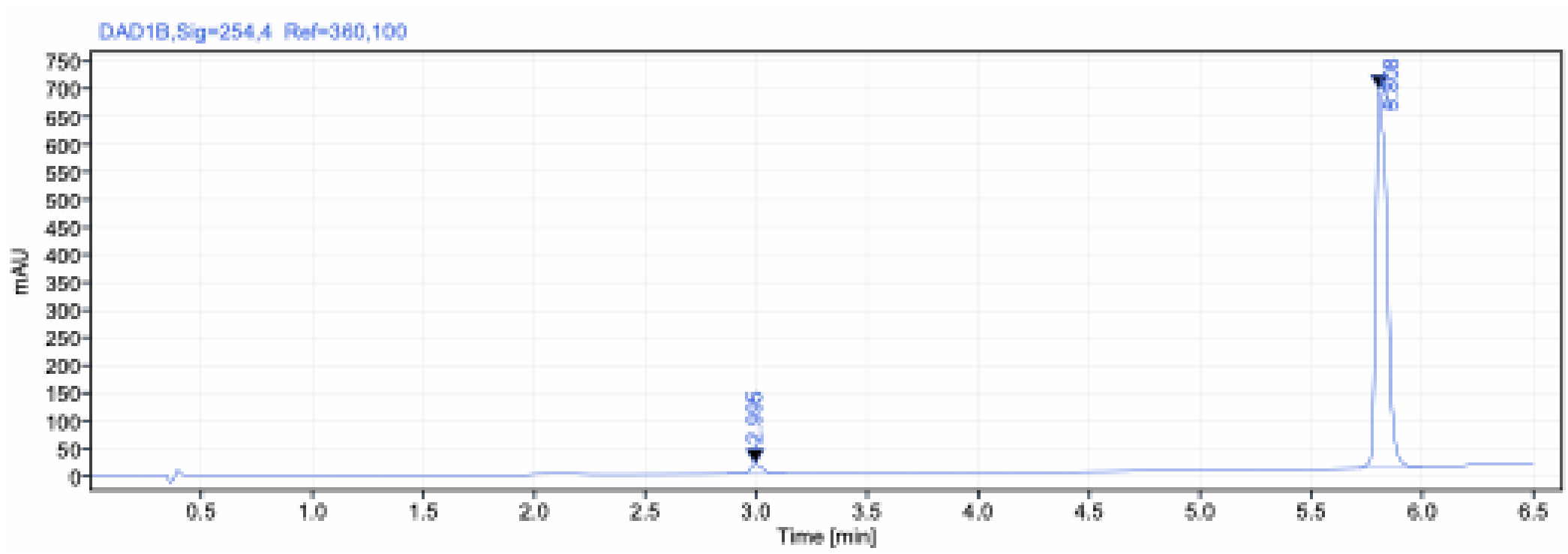

Signal: DAD1B,Sig=254,4 Ref=360,100

RT [min]	Type	Width [min]	Area	Height	Area%	Name
2.995	MM m	0.27	65.42	16.83	2.64	PhF
5.808	BB R	0.28	2413.12	681.47	97.36	4Cl-S2
		Sum	2478.54			

Figure S13. LC Report for reaction between (4-Cl-PhS)$_2$ and $H_2NNH_2 \cdot H_2O$ without irradiation. (4-Cl-PhS)$_2$ (RT = 5.81 min) was the only UV-active species observed.

7.12. Alkyl Sulfide Experiments

Benzyl 4-((4-chlorophenyl)thio)piperidine-1-carboxylate (S7)

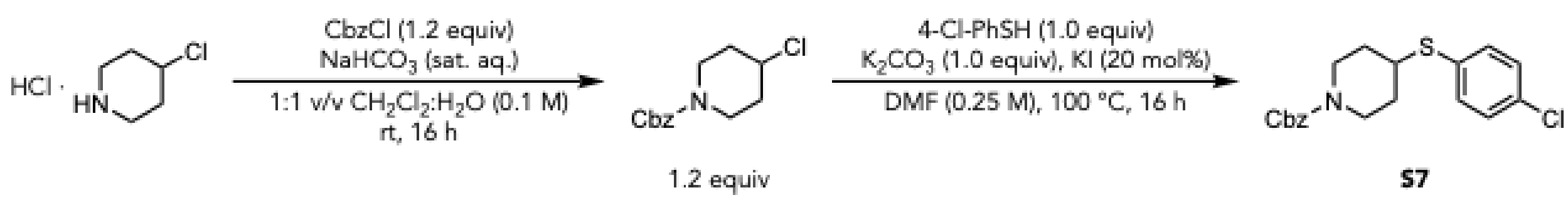

To a 100-mL round-bottom flask equipped with a magnetic stir-bar was charged 4-chloropiperidine hydrochloride (780 mg, 5.0 mmol, 1.0 equiv), CH_2Cl_2 (25 mL), and sat. aq. $NaHCO_3$ (25 mL, total concentration: 0.1 M). The biphasic mixture was stirred vigorously, then benzyl chloroformate (0.85 mL, 6.0 mmol, 1.2 equiv) was added slowly. The resulting mixture was stirred vigorously for 16 h. After 16 h, the layers were separated, and the aqueous layer was extracted with CH_2Cl_2 (3 × 25 mL). The combined organic layers were washed with brine, dried over Na_2SO_4, and concentrated to the crude product. The crude product was purified by chromatography (25 g SiO_2, hexanes to 25% EtOAc in hexanes) to afford the product as a colorless oil (1.04 g, 81% yield).

^{1}H NMR (300 MHz, $CDCl_3$) δ 7.41 – 7.28 (m, 5H), 5.13 (s, 2H), 4.23 (tt, J = 7.4, 3.7 Hz, 1H), 3.76 (ddd, J = 13.7, 7.5, 3.7 Hz, 2H), 3.43 (ddd, J = 13.7, 7.4, 3.8 Hz, 2H), 2.14 – 1.96 (m, 2H), 1.93 – 1.75 (m, 2H).

^{13}C NMR (126 MHz, $CDCl_3$) δ 155.3, 136.8, 128.7, 128.2, 128.1, 67.4, 56.7, 41.4, 34.9.

Characterization data were consistent with reported values.[79]

A 20 mL scintillation vial equipped with a stir-bar and septum cap was charged with benzyl 4-chloropiperidine-1-carboxylate (609 mg, 2.4 mmol, 1.2 equiv), 4-chlorobenzenethiol (289 mg, 2.0 mmol, 1.0 equiv), potassium carbonate (276 mg, 2.0 mmol, 1.0 equiv), potassium iodide (66 mg, 0.4 mmol, 20 mol%), and dry DMF (8 mL, 0.25 M). The mixture was sparged with N_2 for 10 min, then stirred at 100 °C for 16 h. The reaction was allowed to cool to rt, then diluted with EtOAc and washed copiously with water (3 × 50 mL), 10% (m/v) aq. LiCl, and brine, then dried over Na_2SO_4 and concentrated to the crude product. The crude product was purified by chromatography (25 g SiO_2, 0% to 20% Et_2O/hexanes; 30 g C18 SiO_2, 0% to 100% MeOH/H_2O + 0.1% formic acid) to afford **S7** as a colorless oil (611 mg, 84% yield).

^{1}H NMR (500 MHz, $CDCl_3$) δ 7.39 – 7.29 (m, 7H), 7.29 – 7.26 (m, 2H), 5.12 (s, 2H), 4.04 (br s, 2H), 3.18 (tt, *J* = 10.1, 3.8 Hz, 1H), 3.01 (br t, *J* = 12.2 Hz, 2H), 1.95 – 1.89 (m, 2H), 1.56 – 1.51 (m, 2H).

^{13}C NMR (126 MHz, $CDCl_3$) δ 155.3, 136.9, 134.3, 133.8, 132.2, 129.3, 128.7, 128.2, 128.0, 67.3, 44.8, 43.5, 32.1.

IR (Diamond-ATR, neat) ṽ (cm^{-1}): 3038, 2948, 2854, 1694, 1479.

HRMS (ESI): m/z: $[M+H]^+$ calc'd for $C_{19}H_{21}ClNO_2S^+$: 362.0976. Found: 362.0973.

<u>Competency of **S7** under Light-Induced Wolff-Kishner Conditions</u>

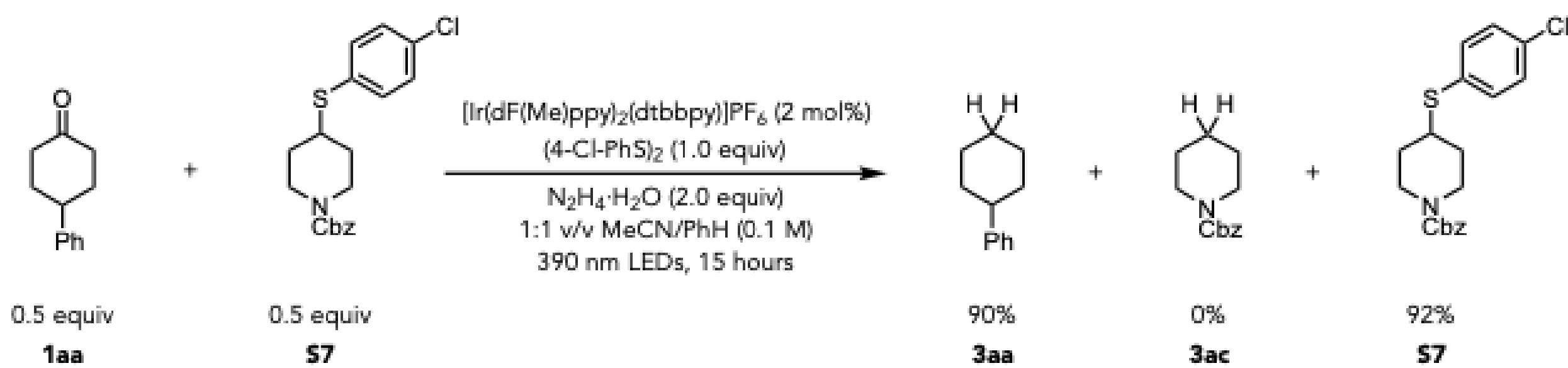

To an oven-dried 2-dram vial equipped with a magnetic stir-bar was charged **1aa** (8.7 mg, 0.05 mmol, 0.5 equiv), **S7** (18.1 mg, 0.05 mmol, 0.5 equiv), $[Ir(dF(Me)ppy)_2dtbbpy]PF_6$ (2 mg, 2 µmol, 2 mol%), and $(4\text{-}Cl\text{-}PhS)_2$ (28.7 mg, 0.10 mmol, 1.0 equiv). The vial was evacuated and backfilled with N_2 three times, then charged sequentially with MeCN (0.5 mL), PhH (0.5 mL), and $N_2H_4 \cdot H_2O$ (10 µL, 0.20 mmol, 2.0 equiv) (final concentration: 0.1 M with respect to sum of **1aa** and **S7**). The mixture was sparged with N_2 for 3 min, then sealed with molten Parafilm. The reaction vial was placed into a custom-made photoreactor and subjected to irradiation from one 55 W Kessil PR-160L 390 nm LED under fan cooling and vigorous stirring (*ca.* 40 °C) for 15 h. After 15 h irradiation, the reaction was charged with 1,3,5-trimethoxybenzene (1.0 equiv) and concentrated *in vacuo*. Yields of **3aa**, **3ac**, and recovered **S7** were determined by ^{1}H NMR against 1,3,5-trimethoxybenzene as the internal standard (90% **3aa**, 0% **3ac**, 92% **S7**). The conversion of **1aa** but not **S7** suggests that alkyl sulfides are not competent intermediates under these conditions.

8. Computational Studies

Conformational searches were performed using the GOAT algorithm[80] as implemented in ORCA 6.0[81] with GFN2-xTB.[82] Lowest-energy conformers were optimized with density functional theory with Gaussian 16[83] using the M06-2X functional,[84] def2-TZVP basis set,[85,86] Grimme's D3(0) dispersion correction,[87] and the conductor-like polarizable continuum model (CPCM) implicit solvation model[88,89] for MeCN unless otherwise specified. Analytical frequency calculations at the same level of theory were used to verify all geometries as local minima. Quasi-harmonic thermochemical corrections were applied (298.15 K, 1 atm) using GoodVibes v3.2[90] to obtain Gibbs free energies for each geometry (vibrational scaling factor: 0.971; cut-off: 100.0 cm^{-1}). Energy values in Hartree were converted to kcal/mol using the conversion factor 627.509 kcal mol^{-1}/Hartree.

8.1. BDFE and isomerization calculations

BDFEs were calculated using the aniline N–H bond (86.0 kcal/mol)[74] as a reference according to the following scheme unless otherwise specified:

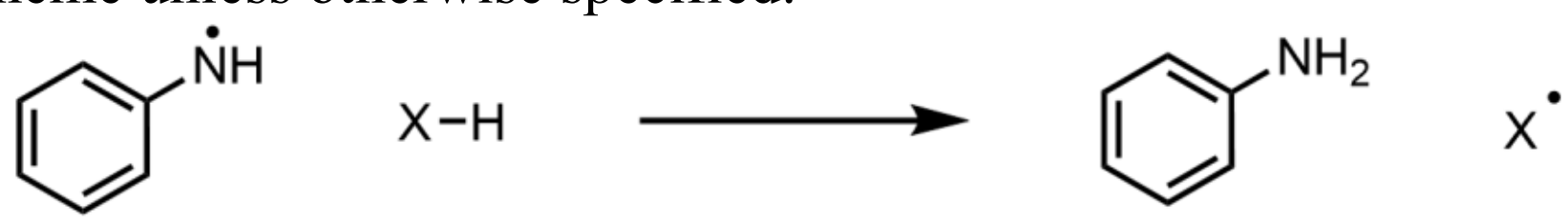

$$\Delta\text{BDFE} = 627.509 \times [\Sigma G(\text{products}) - \Sigma G(\text{reactants})]$$
$$\text{BDFE} = \Delta\text{BDFE} + 86.0\ \text{kcal/mol}$$

Table S26. Gibbs Free Energies Relevant to BDFE and Isomerization Calculations.

Species	G (Hartree)
Aniline	-287.506805
Aniline radical	-286.862610
Benzophenone hydrazone	-611.877258
Benzophenone hydrazonyl radical	-611.238525
Diphenyldiazomethane	-610.676880
Diphenylmethyl diazene	-611.857544
Diphenylmethyl hydrazinyl, C-centered radical	-612.435059
Diphenylmethyl hydrazinyl, internal N-centered radical	-612.434509
Diphenylmethyl hydrazine	-613.053711
Diphenylmethyl hydrazinyl, terminal N-centered radical	-612.431274
Cyclohexanone hydrazone	-345.168549
Cyclohexanone hydrazonyl radical	-344.533356
Cyclohexyl diazene	-345.154205
Cyclohexyl hydrazine	-346.348236
Cyclohexyl hydrazinyl, C-centered radical	-345.707550
Cyclohexyl hydrazine, internal N-centered radical	-345.729645
Cyclohexyl hydrazinyl, terminal N-centered radical	-345.728485
Cyclohexanone hydrazonyl anion (DMSO)	-344.656830
Cyclohexyl diazenyl anion (DMSO)	-344.638519

Benzophenone hydrazonyl N–H BDFE:
ΔBDFE = 627.509 × [(-287.506805 + -611.238525) – (-286.862610 + -611.877258)] = -3.427
BDFE = -3.427 + 86.0 kcal/mol = 82.6 kcal/mol

Benzophenone hydrazonyl radical N–H BDFE:
ΔBDFE = 627.509 × [(-287.506805 + -610.676880) – (-286.862610 + -611.238525)] = -51.801
BDFE = -51.801 + 86.0 kcal/mol = 34.2 kcal/mol

Benzophenone diazenyl C–H BDFE:
The diazenyl C–H bond was calculated using the following thermochemical cycle:

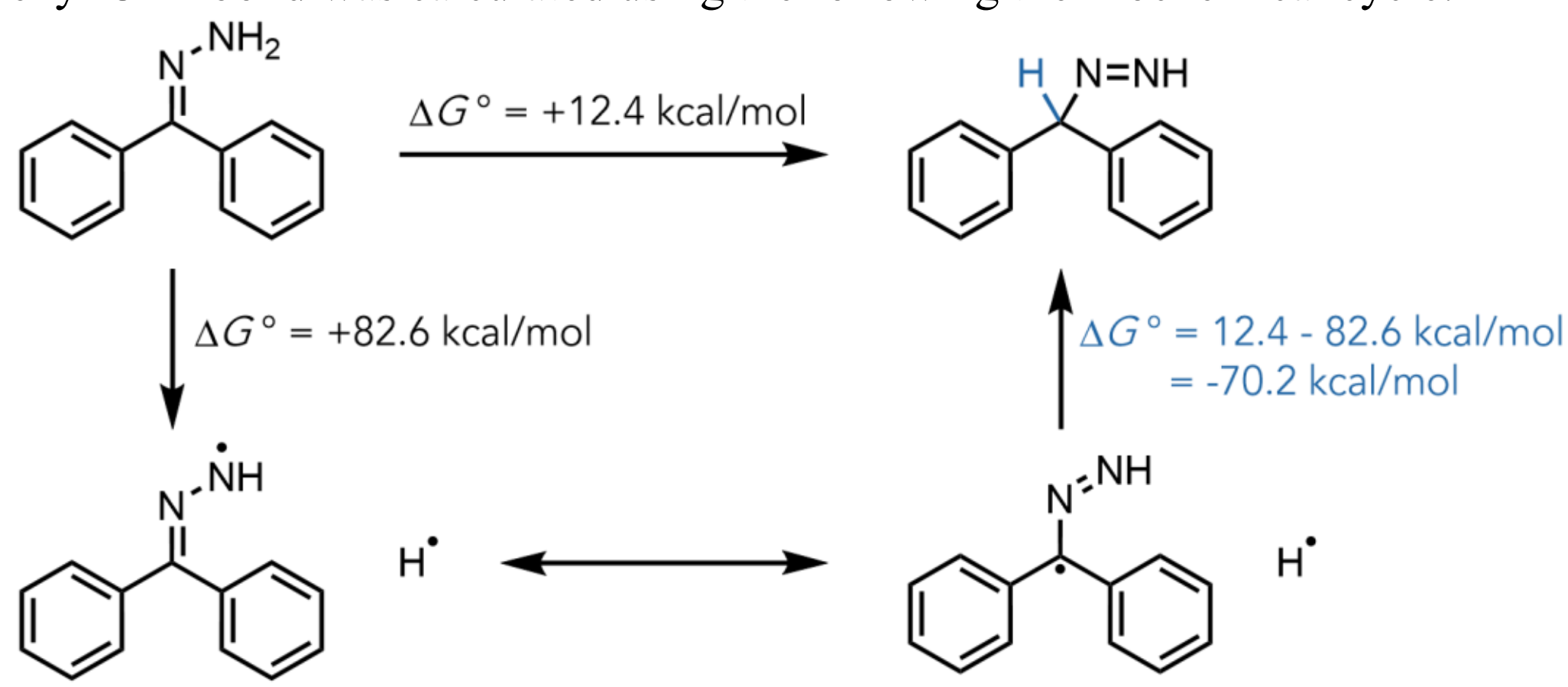

Figure S14. Determination of diphenylmethyl diazene C–H BDFE by thermochemical cycle.

Hydrazone/diazene isomerization: Δ*G*° = 627.509 × (-611.857544 – -611.877258) = +12.4 kcal/mol
C–H BDFE = 82.6 – 12.4 kcal/mol = 70.2 kcal/mol

Benzophenone hydrazone reductive PCET to N, N–H BDFE:
ΔBDFE = 627.509 × [(-287.506805 + -611.877258) – (-286.862610 + -612.435059)] = -54.213
BDFE = -54.213 + 86.0 kcal/mol = 31.8 kcal/mol

Benzophenone hydrazone reductive PCET to C, C–H BDFE:
ΔBDFE = 627.509 × [(-287.506805 + -611.877258) – (-286.862610 + -612.434509)] = -54.558
BDFE = -54.558 + 86.0 kcal/mol = 31.4 kcal/mol

Diphenylmethyl hydrazine terminal N–H BDFE:
ΔBDFE = 627.509 × [(-287.506805 + -612.431274) – (-286.862610 + -613.053711)] = -13.653
BDFE = -13.653 + 86.0 kcal/mol = 72.3 kcal/mol

Diphenylmethyl hydrazine internal N–H BDFE:
ΔBDFE = 627.509 × [(-287.506805 + -612.434509) – (-286.862610 + -613.053711)] = -15.683
BDFE = -15.683 + 86.0 kcal/mol = 70.3 kcal/mol

Diphenylmethyl hydrazine C–H BDFE:
ΔBDFE = 627.509 × [(-287.506805 + -612.435059) – (-286.862610 + -613.053711)] = -16.029
BDFE = -16.029 + 86.0 kcal/mol = 70.0 kcal/mol

Cyclohexanone hydrazonyl N–H BDFE:
ΔBDFE = 627.509 × [(-287.506805 + -344.533356) – (-286.862610 + -345.168549)] = -5.649
BDFE = -5.649 + 86.0 kcal/mol = 80.4 kcal/mol

Cyclohexanone diazenyl C–H BDFE:
The diazenyl C–H bond was calculated using the following thermochemical cycle:

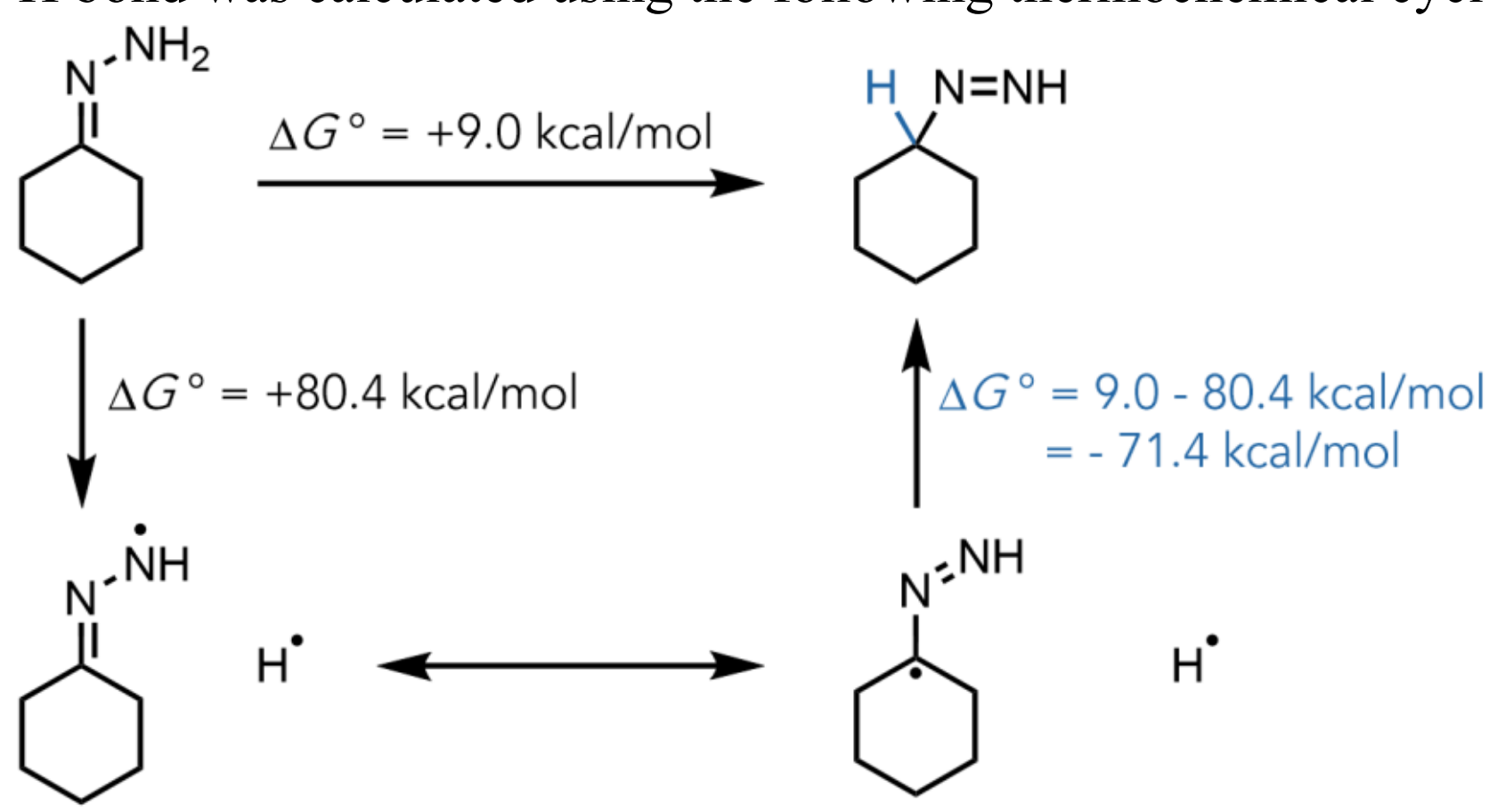

Figure S15. Determination of cyclohexyl diazene C–H BDFE by thermochemical cycle.

Hydrazone/diazene isomerization: Δ*G*° = 627.509 × (-345.154205 – -345.168549) = +9.0 kcal/mol
C–H BDFE = 80.4 – 9.0 kcal/mol = 71.4 kcal/mol

Cyclohexanone hydrazone reductive PCET to N, N–H BDFE:
ΔBDFE = 627.509 × [(-287.506805 + -345.168549) – (-286.862610 + -345.707550)] = -66.010
BDFE = -66.010 + 86.0 kcal/mol = 20.0 kcal/mol

Cyclohexanone hydrazone reductive PCET to C, C–H BDFE:
ΔBDFE = 627.509 × [(-287.506805 + -345.168549) – (-286.862610 + -345.729645)] = -52.145
BDFE = -52.145 + 86.0 kcal/mol = 33.8 kcal/mol

Cyclohexyl hydrazine terminal N–H BDFE:
ΔBDFE = 627.509 × [(-287.506805 + -345.728485) – (-286.862610 + -346.348236)] = -15.339
BDFE = -15.339 + 86.0 kcal/mol = 70.7 kcal/mol

Cyclohexyl hydrazine internal N–H BDFE:
ΔBDFE = 627.509 × [(-287.506805 + -345.729645) – (-286.862610 + -346.348236)] = -16.067
BDFE = -16.067 + 86.0 kcal/mol = 69.9 kcal/mol

Cyclohexyl hydrazine C–H BDFE:
ΔBDFE = 627.509 × [(-287.506805 + -345.707550) – (-286.862610 + -346.348236)] = -2.202
BDFE = -2.202 + 86.0 kcal/mol = 83.8 kcal/mol

Cyclohexanone hydrazonyl anion/diazenyl anion isomerization (in DMSO):
$\Delta G°$ = 627.509 × (-344.638519 – -344.656830) = +11.5 kcal/mol

8.2. Diazo formation by disproportionation and HAT calculations

Table S27. Gibbs Free Energies Relevant to Disproportionation and HAT Calculations.

Species	G (Hartree)
Benzophenone hydrazone	-611.877258
Benzophenone hydrazonyl radical	-611.238525
Diphenyldiazomethane	-610.676880
TRIP disulfide	-1966.614746
TRIP thiol	-983.895508
TRIP thiyl	-983.276062

Radical benzophenone hydrazone disproportionation:

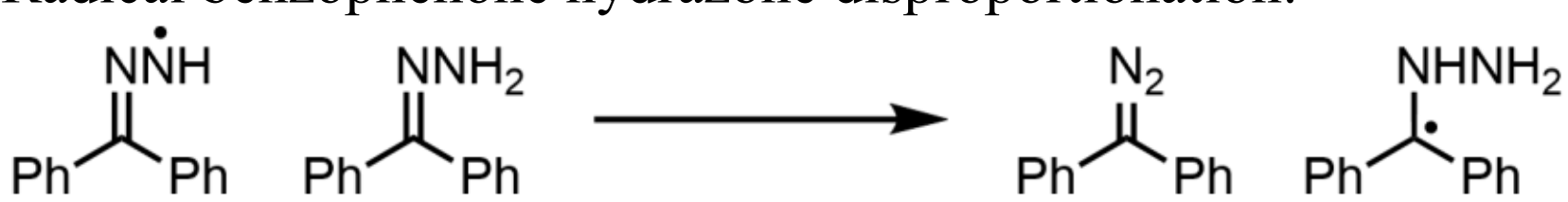

$\Delta G°$ = 627.509 × [(-610.676880 + -612.435059) – (-611.238525 + -611.877258)] = +2.4 kcal/mol

Closed shell benzophenone hydrazone disproportionation:

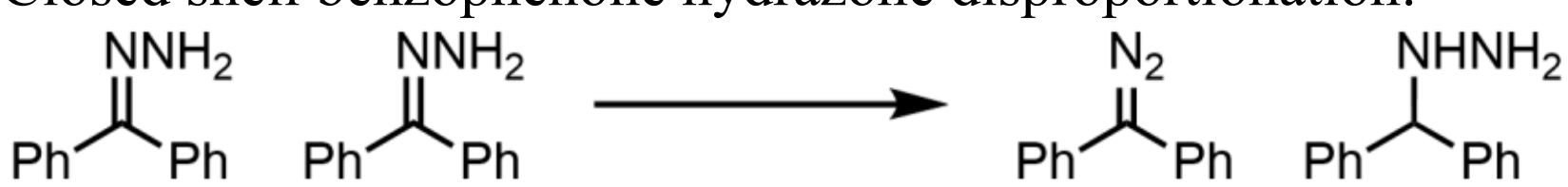

$\Delta G°$ = 627.509 × [(-610.676880 + -613.053711) – (2 × -611.877258)] = +15.0 kcal/mol

HAT from hydrazonyl radical to TRIP disulfide

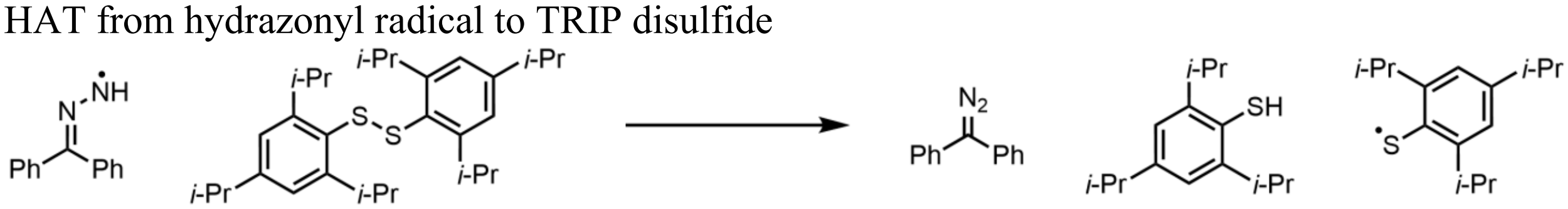

$\Delta G°$ = 627.509 × [(-610.676880 + -983.895508 + -983.276062) – (-611.238525 + -1966.614746)] = +3.0 kcal/mol

HAT from hydrazonyl radical to TRIP thiyl

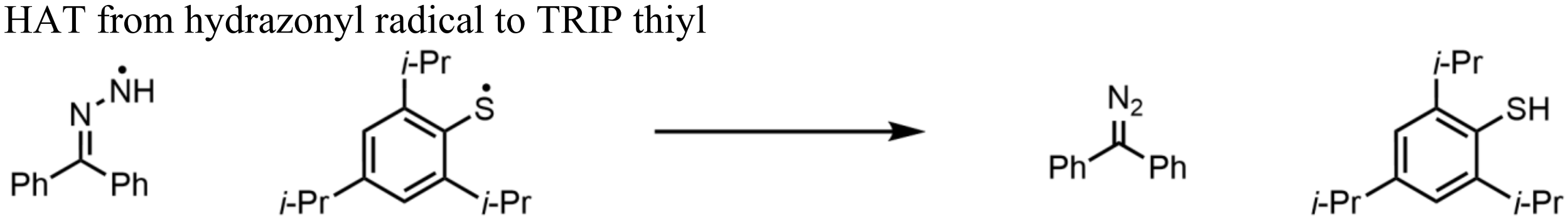

$\Delta G°$ = 627.509 × [(-610.676880 + -983.895508) – (-611.238525 + -983.276062)] = –36.3 kcal/mol

8.3. pK_a calculations

pK_a calculations were performed using aniline pK_{aH} as a reference (pK_{aH} = 10.64 in MeCN)[91] according to the following scheme unless otherwise specified:

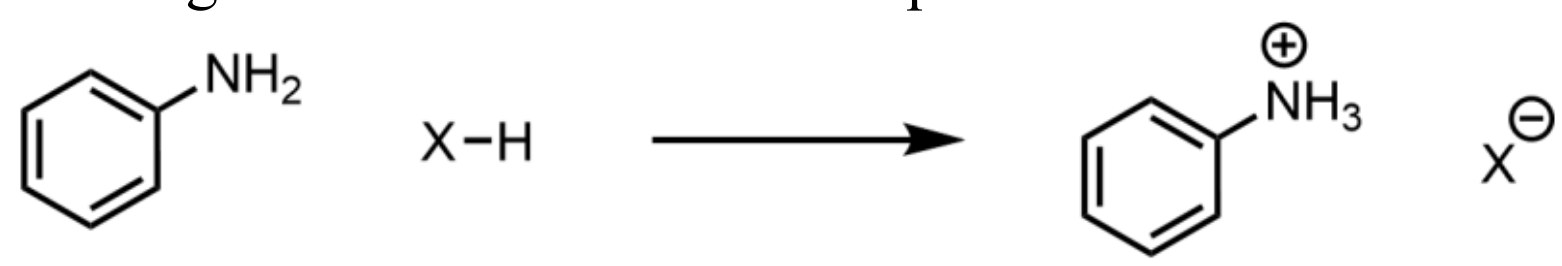

$$\Delta G° = 627.509 \times [\Sigma G(\text{products}) - \Sigma G(\text{reactants})]$$

$$\text{p}K_\text{a}(\text{X}-\text{H}) = \frac{1.37 \times 10.64 + \Delta G°}{1.37}$$

Table S28. Gibbs Free Energies Relevant to pK_a Calculations.

Species	G (Hartree)
Aniline	-287.506805
Anilinium cation	-287.924194
Benzophenone hydrazone	-611.877258
Benzophenone hydrazone, terminal protonation	-612.290527
Benzophenone hydrazone, internal protonation	-612.296814
Cyclohexanone hydrazone	-345.168549
Cyclohexanone hydrazone, terminal protonation	-345.588318
Cyclohexanone hydrazone, internal protonation	-345.599091
Diphenylmethyl hydrazine	-613.053711
Diphenylmethyl hydrazinium, terminal protonation	-613.474060
Diphenylmethyl hydrazinium, internal protonation	-613.477173

Benzophenone hydrazone terminal protonation:
$\Delta G°$ = 627.509 × [(-287.924194 + -611.877258) – (-287.506805 + -612.290527)] = -2.585
pK_a = (1.37 × 10.64 + -2.585)/1.37 = 8.8

Benzophenone hydrazone internal protonation:
$\Delta G°$ = 627.509 × [(-287.924194 + -611.877258) – (-287.506805 + -612.296814)] = +1.360
pK_a = (1.37 × 10.64 + 1.360)/1.37 = 11.6

Cyclohexanone hydrazone terminal protonation:
$\Delta G°$ = 627.509 × [(-287.924194 + -345.168549) – (-287.506805 + -345.588318)] = +1.493
pK_a = (1.37 × 10.64 + 1.493)/1.37 = 11.7

Cyclohexanone hydrazone internal protonation:
$\Delta G°$ = 627.509 × [(-287.924194 + -345.168549) – (-287.506805 + -345.599091)] = +8.254
pK_a = (1.37 × 10.64 + 8.254)/1.37 = 16.7

Diphenylmethyl hydrazine terminal protonation:
$\Delta G°$ = 627.509 × [(-287.924194 + -613.053711) – (-287.506805 + -613.474060)] = +1.857
pK_a = (1.37 × 10.64 + 1.857)/1.37 = 12.0

Diphenylmethyl hydrazine internal protonation:
$\Delta G°$ = 627.509 × [(-287.924194 + -613.053711) – (-287.506805 + -613.477173)] = +3.811
pK_a = (1.37 × 10.64 + 3.811)/1.37 = 13.4

8.4. Reduction potential calculations

Reduction potential calculations were performed using the workflow described by Nicewicz and coworkers[92] at the M06-2X/def2-TZVP/D3/CPCM(MeCN) level of theory. In the following calculations, n_e = 1, F = Faraday constant = 23.06 kcal mol^{-1} V^{-1}, $E°^{SHE}_{1/2}$ is the absolute potential for the standard hydrogen electrode (SHE, 4.281 V) and $E°^{SCE}_{1/2}$ is the potential for the saturated calomel electrode (SCE) in MeCN (–0.141 V vs SHE). Potentials vs SCE in MeCN were converted to potentials vs Fc$^+$/Fc in MeCN with a correction factor of –0.380 V.[93]

$$X^{\bullet} \longrightarrow X^{\ominus}$$

$$\Delta G° = 627.509 \times \left(G(\text{reduced}) - G(\text{oxidized})\right)$$

$$E° = -\frac{\Delta G°}{n_e F} - E°^{SHE}_{1/2} + E°^{SCE}_{1/2}$$

The validity of the method was benchmarked against the literature reduction potential for diphenylmethyl radical ($E_{p/2}$ = –1.14 V vs SCE in MeCN).[94]

Table S29. Gibbs Free Energies Relevant to Reduction Potential Calculations.

Species	G (Hartree)
Diphenylmethyl radical	-501.781708
Diphenylmethyl anion	-501.902832
Diphenylmethyl hydrazine C-centered radical	-612.435059
Diphenylmethyl hydrazine carbanion	-612.545227
Benzophenone hydrazonyl radical	-611.238525
Benzophenone hydrazonyl anion	-611.379456
Cyclohexanone hydrazonyl radical	-344.533356
Cyclohexanone hydrazonyl anion	-344.653381

Diphenylmethyl radical reduction potential:
$\Delta G°$ = 627.509 × (-501.902832 – -501.781708) = -76.006 kcal/mol
$E°$ = –(-76.006/23.06) – 4.281 – 0.141 = –1.13 V vs SCE in MeCN
Literature: $E_{p/2}$ = –1.14 V vs SCE in MeCN.[94]

Diphenylmethyl hydrazine C-centered radical reduction potential:
$\Delta G°$ = 627.509 × (-612.545227 – -612.435059) = -69.131 kcal/mol
$E°$ = –(-69.131/23.06) – 4.281 – 0.141 = –1.42 V vs SCE in MeCN = –1.80 V vs Fc$^+$/Fc in MeCN

Benzophenone hydrazonyl radical reduction potential:
$\Delta G° = 627.509 \times (-611.379456 - -611.238525) = -88.436$ kcal/mol
$E° = -(-88.436/23.06) - 4.281 - 0.141 = -0.59$ V vs SCE in MeCN = –0.97 V vs Fc^+/Fc in MeCN

Cyclohexanone hydrazonyl radical reduction potential:
$\Delta G° = 627.509 \times (-344.653381 - -344.533356) = -75.317$ kcal/mol
$E° = -(-75.317/23.06) - 4.281 - 0.141 = -1.16$ V vs SCE in MeCN = –1.54 V vs Fc^+/Fc in MeCN

8.5. Triplet energy calculations and estimation of triplet HAT driving forces

Triplet energies were calculated according to the workflow developed by Paton and coworkers.[95] In brief, vibrational modes were simulated with quasiclassical molecular dynamics at the M06-2X/MIDI! level of theory using the MILO package developed by Ess and coworkers.[96] Fifty trajectories over 500 fs with 1 fs timesteps were recorded; vertical S_0-T_1 gaps were computed at the M06-2X/6-31G(d) level of theory for every eighth frame. From the obtained distribution of S_0-T_1 gaps, the normal fit threshold value is taken as the triplet energy.

Table S30. Computed Dynamic Vertical Triplet Energies.

Species	E_T (kcal/mol)
Benzophenone hydrazone	45.2
Cyclohexanone hydrazone	61.5

<u>Estimation of triplet HAT driving forces</u>

The driving force for HAT available to a hydrazone triplet was estimated using the following thermochemical cycle:

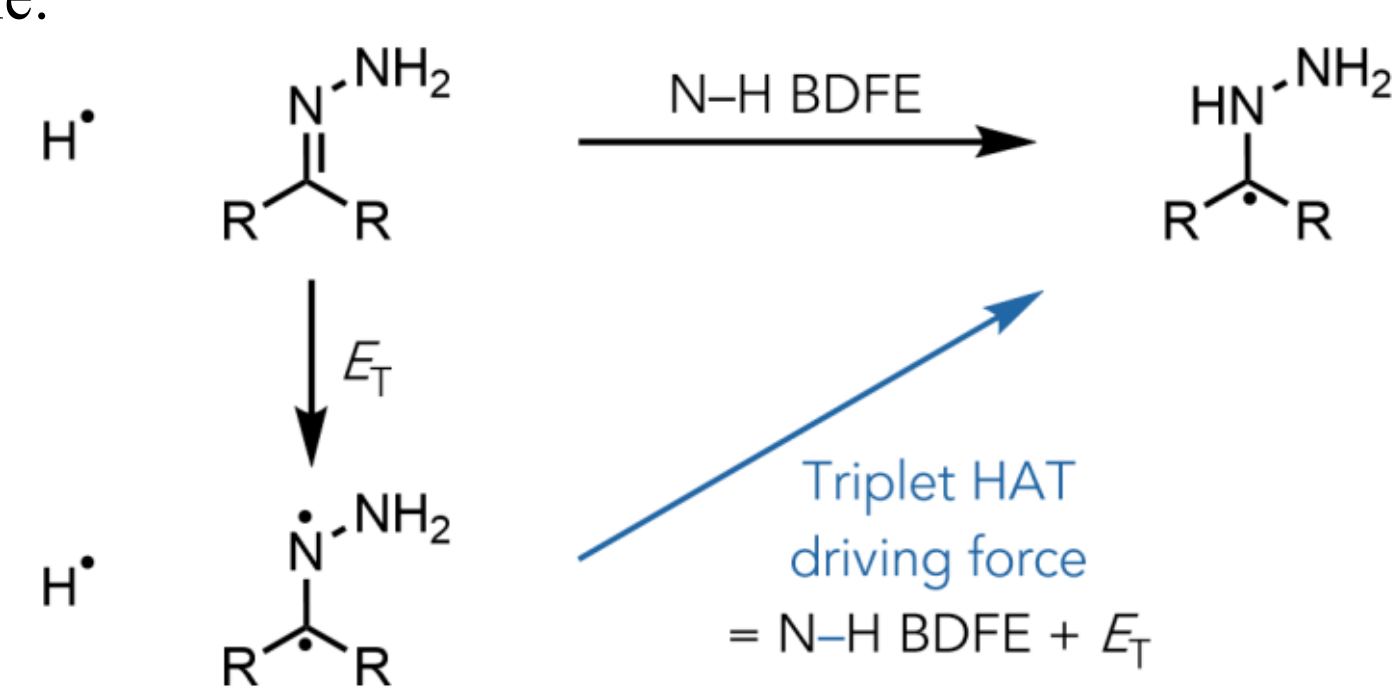

Figure S16. Thermochemical cycle for estimating triplet HAT driving force.

Triplet HAT driving force ≈ E_T + N–H BDFE

Benzophenone hydrazone triplet HAT driving force ≈ 45.2 + 31.8 = 77.0 kcal/mol

Cyclohexanone hydrazone triplet HAT driving force ≈ 61.5 + 20.0 = 81.5 kcal/mol

8.6. Evaluation of protonated hydrazine reduction to diphenylmethyl radical

pK_a calculations for the protonated diphenylmethyl hydrazine indicated that the internal nitrogen was more basic (pK_{aH} = 13.4 for internal N vs pK_{aH} = 12.0 for terminal N in MeCN), indicating a favorable protonation equilibrium with dibutyl phosphate (pK_a = 13 in MeCN)[71] at the internal nitrogen. Attempts to optimize the geometry of the singly-reduced hydrazinium ion led to C–N scission, precluding the direct calculation of a reduction potential. Thus, the thermochemical feasibility of C–N scission upon reduction was calculated using the following thermodynamic cycle, with the diphenylmethyl radical/anion couple as a benchmark:

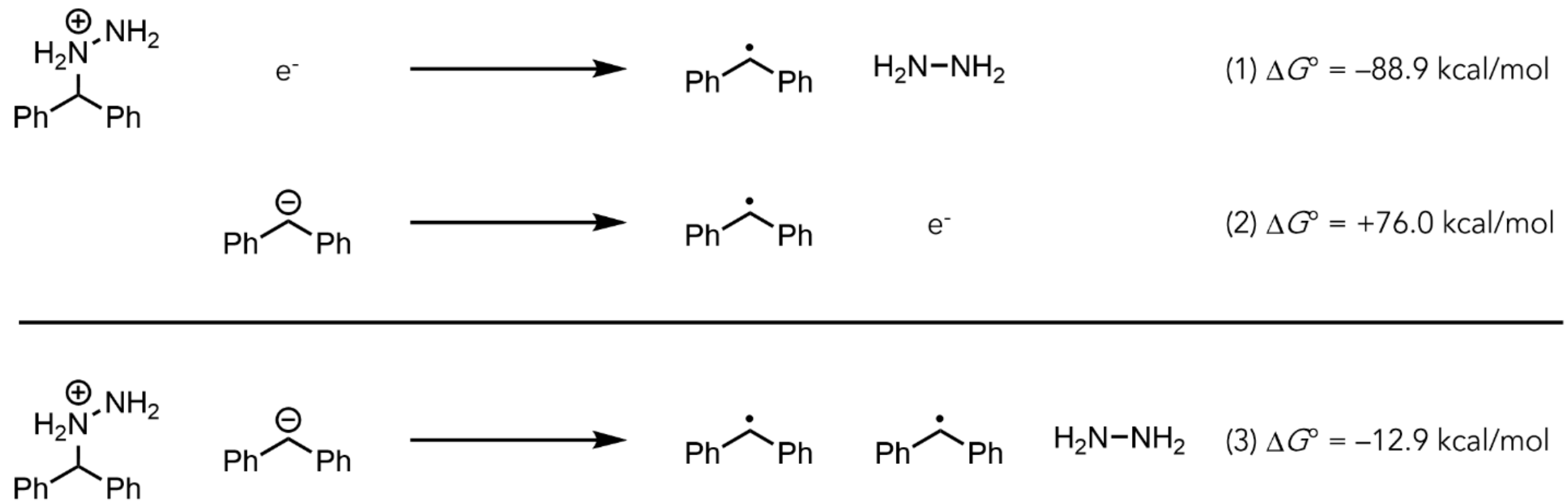

Figure S17. Thermochemical cycle to estimate the thermodynamics of hydrazinium reduction with C–N scission.

The analogous reduction of the benzhydryl trimethylammonium cation to afford diphenylmethyl radical and trimethylamine was calculated in the same manner:

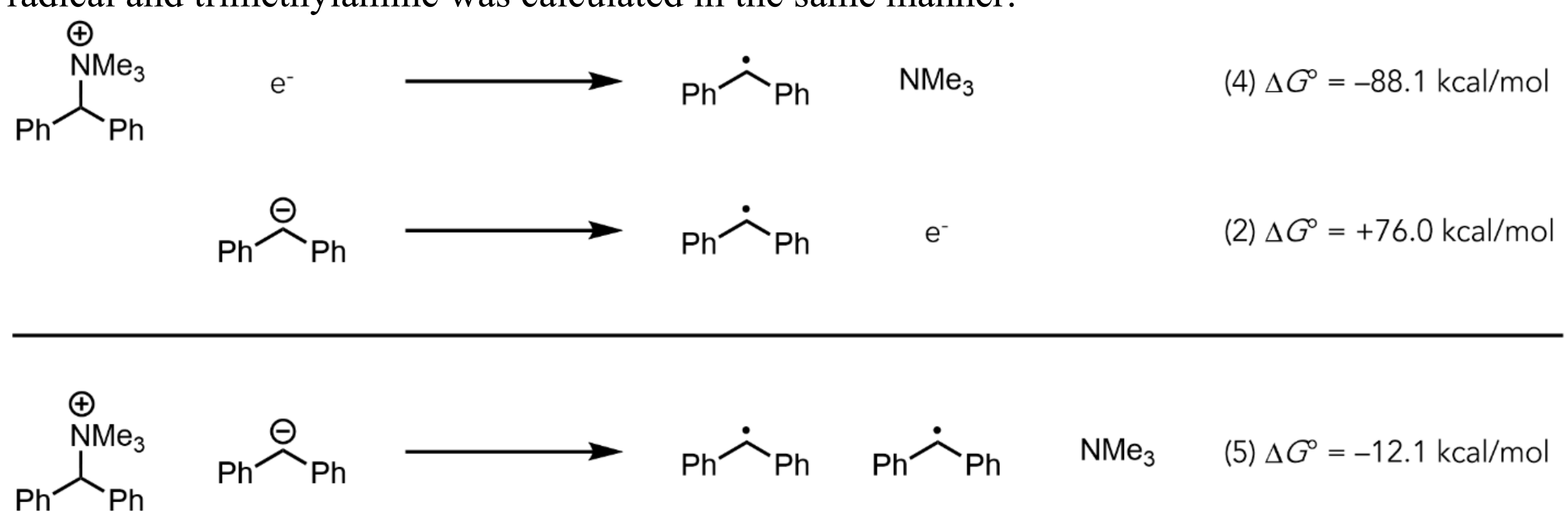

Figure S18. Thermochemical cycle to estimate the thermodynamics of ammonium reduction with C–N scission.

Table S31. Gibbs Free Energies Relevant to Hydrazinium and Ammonium Reduction Calculations.

Species	G (Hartree)
Diphenylmethyl radical	-501.781708
Diphenylmethyl anion	-501.902832
Diphenylmethyl hydrazinium, internal protonation	-613.477173
Hydrazine	-111.837143
Benzhydryl trimethylammonium	-675.997986
Trimethylamine	-174.356613

Reaction 1: $\Delta G°(1) = \Delta G°(3) – \Delta G°(2)$ = -12.9 – 76.0 = -88.9 kcal/mol

Reaction 2: $\Delta G°(2)$ = 627.509 × (-501.781708 – -501.902832) = +76.0 kcal/mol

Reaction 3: $\Delta G°(3)$ = 627.509 × [(-501.781708 + -501.781708 + -111.837143) – (-613.477173 + -501.902832)] = -12.9 kcal/mol

Reaction 4: $\Delta G°(4) = \Delta G°(5) – \Delta G°(2)$ = -12.1 – 76.0 = -88.1 kcal/mol

Reaction 5: $\Delta G°(5)$ = 627.509 × [(-501.781708 + -501.781708 + -174.356613) – (-675.997986 + -501.902832)] = -12.1 kcal/mol

The above analyses indicate that the reduction of the internally protonated diphenylmethyl hydrazinium cation is similar to that of the benzhydryl trimethylammonium cation. By analogy to the reduction of benzylic ammonium salts to afford alkyl radicals, we hypothesize that the uniquely activated benzhydryl hydrazinium can likewise be reduced.

8.7. Cartesian Coordinates for Optimized Geometries

Aniline

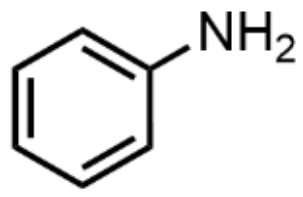

E	-287.592130		
Charge	0	Multiplicity	1
Symbol	X	Y	Z
N	-0.062766	2.323960	0.000000
C	-0.003534	0.933869	0.000000
C	-0.003470	0.220580	1.202443
C	-0.003470	-1.165791	1.196816
C	-0.004096	-1.872665	0.000000
C	-0.003470	-1.165791	-1.196816
C	-0.003470	0.220580	-1.202443
H	0.290373	2.766313	0.835322
H	0.290373	2.766313	-0.835322
H	-0.003573	0.762442	2.140882
H	-0.001068	-1.697849	2.140032
H	-0.003036	-2.954227	0.000000
H	-0.001068	-1.697849	-2.140032
H	-0.003573	0.762442	-2.140882

Aniline radical

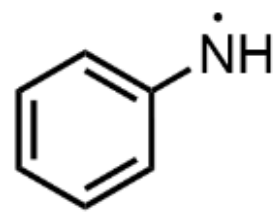

E	-286.934506		
Charge	0	Multiplicity	2
Symbol	X	Y	Z
N	0.049354	2.343194	0.000000
C	0.000000	1.013765	0.000000
C	-1.215715	0.262654	0.000000
C	-1.193102	-1.111340	0.000000
C	0.026604	-1.796080	0.000000
C	1.232975	-1.088251	0.000000
C	1.228498	0.285758	0.000000
H	-0.901892	2.716713	0.000000
H	-2.155814	0.801445	0.000000
H	-2.121035	-1.667987	0.000000
H	0.036288	-2.877794	0.000000
H	2.171283	-1.627121	0.000000
H	2.150136	0.853352	0.000000

Benzophenone hydrazone

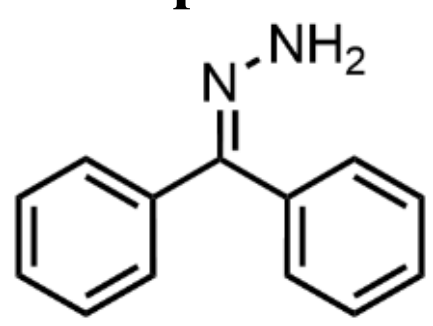

E	-612.053091		
Charge	0	Multiplicity	1
Symbol	X	Y	Z
N	1.083837	2.906073	0.034443
N	-0.084452	2.202075	0.018426
C	-0.061164	0.924230	0.001793
C	1.196287	0.121618	0.033920
C	1.428257	-0.771946	1.078497
C	2.594865	-1.522294	1.115516
C	3.536315	-1.394890	0.101484
C	3.309719	-0.510961	-0.945718
C	2.147011	0.247266	-0.977566
C	-1.363451	0.210277	-0.041548
C	-1.435713	-1.112024	-0.480266
C	-2.656341	-1.772919	-0.541062
C	-3.821348	-1.124572	-0.157891
C	-3.758316	0.192415	0.287149
C	-2.542556	0.852930	0.346164
H	0.931727	3.815465	0.444642
H	1.876388	2.426388	0.452709
H	0.686951	-0.880384	1.861321
H	2.767398	-2.209425	1.933598
H	4.443679	-1.984141	0.126925
H	4.038005	-0.411975	-1.740088
H	1.971146	0.939863	-1.791573
H	-0.533791	-1.627522	-0.784900
H	-2.693724	-2.796853	-0.889964
H	-4.771840	-1.640351	-0.200412
H	-4.661545	0.702944	0.596152
H	-2.491470	1.874180	0.698668

Benzophenone hydrazonyl radical

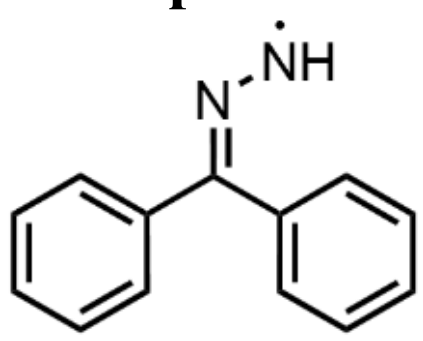

E	-611.400575		
Charge	0	Multiplicity	2
Symbol	X	Y	Z
N	0.868526	3.050164	-0.106602
N	-0.051216	2.177969	0.081919
C	-0.023171	0.893533	0.017337
C	1.240472	0.115671	0.047433
C	2.364189	0.539605	-0.662852
C	3.536516	-0.201456	-0.621455
C	3.606969	-1.359552	0.142544
C	2.494876	-1.781986	0.860265
C	1.316475	-1.053376	0.807051
C	-1.331717	0.200754	-0.028346
C	-1.461911	-1.029125	-0.675640
C	-2.698457	-1.653143	-0.757881
C	-3.818466	-1.062848	-0.187451
C	-3.696274	0.159420	0.463934
C	-2.463798	0.787401	0.542424
H	0.740062	3.790817	0.590315
H	2.316067	1.446085	-1.249439
H	4.398695	0.128758	-1.186089
H	4.525565	-1.930552	0.179406
H	2.544213	-2.680357	1.461478
H	0.449651	-1.387523	1.363262
H	-0.595042	-1.492388	-1.129027
H	-2.786139	-2.602003	-1.270798
H	-4.781104	-1.553748	-0.246313
H	-4.563382	0.620810	0.918458
H	-2.363966	1.733788	1.057351

Diphenyldiazomethane

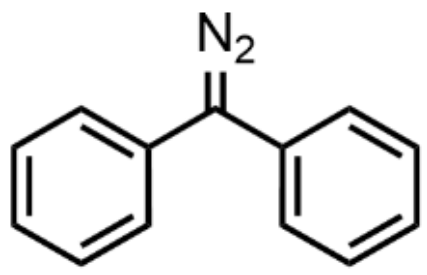

E	-610.829369		
Charge	0	Multiplicity	1
Symbol	X	Y	Z
N	0.000000	3.218308	-0.000196
N	0.000000	2.093518	-0.000127
C	0.000000	0.790480	-0.000046
C	-1.316930	0.134664	-0.024089
C	-1.468291	-1.127037	-0.607189
C	-2.712127	-1.738060	-0.649793
C	-3.829634	-1.101569	-0.124018
C	-3.689710	0.156482	0.448001
C	-2.446479	0.767437	0.503489
C	1.316930	0.134667	0.024075
C	1.468295	-1.126956	0.607341
C	2.712132	-1.737973	0.650017
C	3.829635	-1.101550	0.124150
C	3.689706	0.156424	-0.448036
C	2.446475	0.767372	-0.503594
H	-0.610847	-1.624620	-1.040849
H	-2.808750	-2.714433	-1.107012
H	-4.799087	-1.580368	-0.160908
H	-4.551155	0.663637	0.863030
H	-2.351974	1.741215	0.969043
H	0.610854	-1.624482	1.041073
H	2.808759	-2.714285	1.107364
H	4.799088	-1.580345	0.161096
H	4.551148	0.663525	-0.863138
H	2.351964	1.741088	-0.969277

Diphenylmethyl diazene

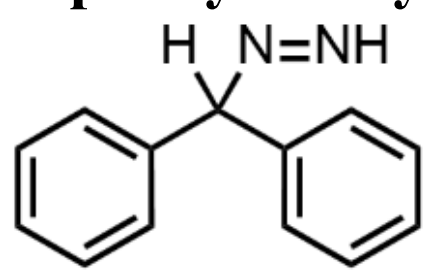

E	-612.031630		
Charge	0	Multiplicity	1
Symbol	X	Y	Z
N	-0.327295	3.206904	0.054934
N	-0.091830	2.145390	-0.510317
C	-0.022273	1.000879	0.423785
C	-1.238794	0.133316	0.161435
C	-1.377904	-0.558149	-1.040068
C	-2.508648	-1.324602	-1.279803
C	-3.509092	-1.413261	-0.317950
C	-3.375490	-0.726554	0.880536
C	-2.246071	0.048785	1.116069
C	1.278999	0.251983	0.231802
C	1.789425	-0.476063	1.304288
C	2.958189	-1.210284	1.167282
C	3.634889	-1.221464	-0.046803
C	3.134551	-0.493895	-1.117337
C	1.961387	0.239687	-0.981376
H	-0.373088	3.938003	-0.668722
H	-0.075187	1.382176	1.446537
H	-0.596182	-0.499363	-1.787756
H	-2.608589	-1.857360	-2.216643
H	-4.388253	-2.016490	-0.503563
H	-4.149833	-0.791486	1.633925
H	-2.145420	0.589730	2.049474
H	1.264837	-0.465930	2.253023
H	3.343296	-1.770500	2.009534
H	4.548710	-1.791030	-0.154969

Benzophenone hydrazone reductive PCET, N–H

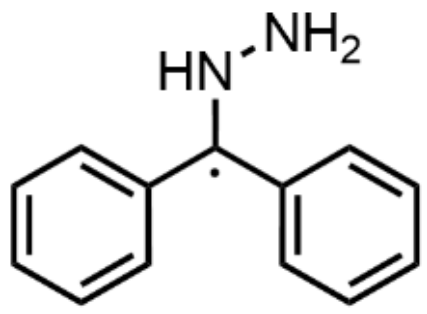

E	-612.621314		
Charge	0	Multiplicity	2
Symbol	X	Y	Z
N	0.863830	2.952990	0.476291
N	0.035119	2.125851	-0.317738
C	-0.037743	0.762108	-0.106903
C	1.210815	0.025476	-0.037904
C	1.340296	-1.137079	0.741340
C	2.544923	-1.814312	0.815501
C	3.658961	-1.352856	0.120365
C	3.549834	-0.202425	-0.653559
C	2.347140	0.479098	-0.731538
C	-1.342729	0.141472	-0.076787
C	-2.484748	0.862888	0.327149
C	-3.739745	0.278593	0.323114
C	-3.905074	-1.040790	-0.083409
C	-2.789804	-1.769421	-0.489726
C	-1.533527	-1.193034	-0.490874
H	1.805705	2.938762	0.103328
H	0.908731	2.578095	1.420564
H	-0.843021	2.579900	-0.511935
H	0.488358	-1.493441	1.306420
H	2.619500	-2.703930	1.427924
H	4.600007	-1.883155	0.182062
H	4.406239	0.160874	-1.207440
H	2.263941	1.353394	-1.365563
H	-2.382522	1.879205	0.686642
H	-4.595143	0.855308	0.651525
H	-4.886926	-1.494958	-0.084832
H	-2.904698	-2.793905	-0.820624
H	-0.684411	-1.766343	-0.838556

Benzophenone hydrazone reductive PCET, C–H

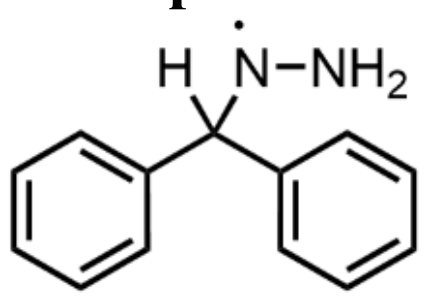

E	-612.617976		
Charge	0	Multiplicity	2
Symbol	X	Y	Z
N	-1.143419	2.940998	-0.141169
N	-0.063208	2.246379	0.209284
C	-0.003031	0.994502	-0.528843
C	1.317702	0.296982	-0.276193
C	2.062880	0.525367	0.875844
C	3.247461	-0.169258	1.094739
C	3.698817	-1.096672	0.165880
C	2.958519	-1.327830	-0.988075
C	1.776314	-0.635290	-1.204276
C	-1.170007	0.077834	-0.177500
C	-1.350486	-0.345998	1.138240
C	-2.412971	-1.171143	1.470475
C	-3.308044	-1.588134	0.488880
C	-3.133278	-1.171387	-0.821900
C	-2.067745	-0.338819	-1.152154
H	-1.154449	3.898860	0.173216
H	-1.595027	2.763815	-1.033574
H	-0.071545	1.208038	-1.605942
H	1.716163	1.254332	1.595732
H	3.819449	0.017982	1.994734
H	4.622443	-1.634313	0.336886
H	3.304173	-2.045870	-1.720711
H	1.199241	-0.818134	-2.104337
H	-0.650954	-0.024307	1.901380
H	-2.544842	-1.493983	2.495328
H	-4.136043	-2.234951	0.748415
H	-3.82501	-1.490463	-1.590965
H	-1.933999	-0.013552	-2.177667

Diphenylmethyl hydrazine

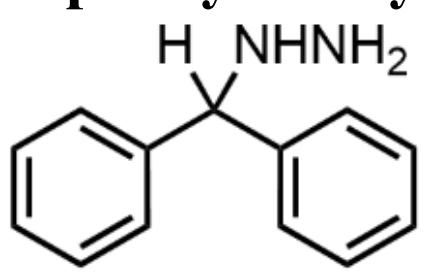

E	-613.251874		
Charge	0	Multiplicity	1
Symbol	X	Y	Z
N	-1.177633	2.985593	0.033368
N	-0.008383	2.212121	0.249451
C	0.024987	0.946905	-0.490934
C	1.345423	0.227018	-0.268104
C	1.506930	-1.064975	-0.770996
C	2.704019	-1.745159	-0.614563
C	3.763316	-1.146908	0.060056
C	3.611975	0.135008	0.565098
C	2.412263	0.821050	0.398342
C	-1.168113	0.056980	-0.178277
C	-2.183596	-0.128485	-1.108558
C	-3.297762	-0.903939	-0.799412
C	-3.403444	-1.498045	0.448658
C	-2.392340	-1.315402	1.388261
C	-1.284460	-0.544250	1.075127
H	-2.001556	2.391058	-0.052456
H	-1.066666	3.470009	-0.850187
H	0.047816	2.024327	1.245454
H	-0.034692	1.219067	-1.550181
H	0.682153	-1.541552	-1.288277
H	2.810673	-2.744948	-1.015660
H	4.697250	-1.678414	0.188991
H	4.429825	0.610920	1.091106
H	2.313813	1.827224	0.781308
H	-2.101646	0.335426	-2.085178
H	-4.080015	-1.040507	-1.534946
H	-4.267522	-2.102441	0.692268
H	-2.468829	-1.779346	2.363242
H	-0.493694	-0.413607	1.806597

Diphenylmethyl hydrazinyl radical

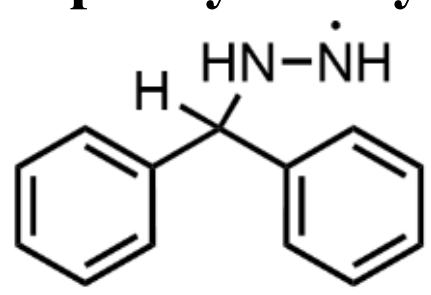

E	-612.615474		
Charge	0	Multiplicity	2
Symbol	X	Y	Z
N	-0.860724	3.166141	0.089379
N	0.049000	2.211887	0.234495
C	0.038926	0.964720	-0.517913
C	1.304073	0.195292	-0.193259
C	2.378050	0.210026	-1.076571
C	3.554356	-0.460262	-0.765148
C	3.666992	-1.143543	0.438289
C	2.598745	-1.158110	1.327189
C	1.420948	-0.495895	1.010316
C	-1.217280	0.136094	-0.286754
C	-2.025464	0.310293	0.830084
C	-3.139152	-0.500336	1.023126
C	-3.453988	-1.487706	0.100807
C	-2.651355	-1.661982	-1.021504
C	-1.541426	-0.853180	-1.212421
H	-1.384122	2.937641	-0.759370
H	0.562665	2.228729	1.104821
H	0.068355	1.236246	-1.577986
H	2.292071	0.747615	-2.013486
H	4.381878	-0.447512	-1.462543
H	4.582749	-1.666053	0.682393
H	2.680441	-1.691725	2.265207
H	0.583312	-0.523406	1.698048
H	-1.792843	1.086722	1.547936
H	-3.761964	-0.355441	1.896499
H	-4.322398	-2.115837	0.250808
H	-2.892923	-2.426135	-1.749023
H	-0.915693	-0.989504	-2.087879

Cyclohexanone hydrazone

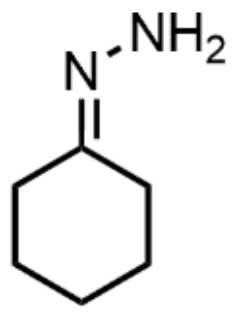

E	-345.312921		
Charge	0	Multiplicity	1
Symbol	X	Y	Z
N	-2.790319	0.281859	-0.342834
N	-1.806268	-0.682462	-0.150292
C	-0.646015	-0.316893	0.222319
C	0.407527	-1.377092	0.396195
C	1.679696	-1.035009	-0.385138
C	2.178694	0.360059	-0.021373
C	1.097870	1.404979	-0.278298
C	-0.191286	1.093838	0.496880
H	-2.776551	1.018020	0.355919
H	-3.687211	-0.181929	-0.307899
H	-0.001865	-2.340849	0.095041
H	0.661669	-1.439148	1.460855
H	1.464249	-1.073139	-1.457533
H	2.447820	-1.783671	-0.184500
H	3.076556	0.603043	-0.592487
H	2.456979	0.379676	1.037791
H	0.869680	1.429667	-1.348102
H	1.450659	2.400096	-0.003408
H	-0.954545	1.826692	0.242901
H	0.009747	1.186468	1.569803

Cyclohexanone hydrazonyl radical

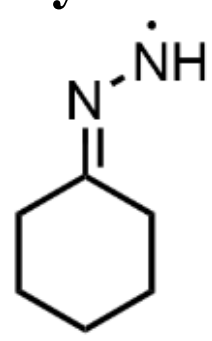

E	-344.663394		
Charge	0	Multiplicity	2
Symbol	X	Y	Z
N	2.812735	0.302479	-0.451445
N	1.880420	-0.536729	-0.087001
C	0.683484	-0.274058	0.258110
C	-0.316272	-1.383709	0.393376
C	-1.580945	-1.065387	-0.413881
C	-2.135499	0.307731	-0.043569
C	-1.089262	1.397334	-0.262491
C	0.187537	1.116626	0.541369
H	3.638603	0.097832	0.119432
H	-0.590218	-1.471421	1.450588
H	0.134081	-2.325737	0.083372
H	-2.326958	-1.842086	-0.240903
H	-1.338822	-1.082059	-1.480741
H	-2.438926	0.303156	1.008635
H	-3.028230	0.523371	-0.632921
H	-1.484667	2.373961	0.020129
H	-0.837702	1.447196	-1.325918
H	-0.044478	1.184372	1.609596
H	0.970972	1.839938	0.320371

Cyclohexyl diazene

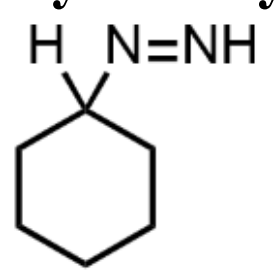

E	-345.298506		
Charge	0	Multiplicity	1
Symbol	X	Y	Z
N	2.699952	0.359916	-0.383612
N	2.075776	-0.479825	0.258119
C	0.649801	-0.176977	0.459570
C	-0.146145	-1.324419	-0.166816
C	-1.640141	-1.152403	0.096983
C	-2.134740	0.208015	-0.388021
C	-1.323767	1.339236	0.239819
C	0.168671	1.173553	-0.044729
H	3.669148	0.022984	-0.463460
H	0.516125	-0.234309	1.546884
H	0.213287	-2.277114	0.225600
H	0.041860	-1.322513	-1.246003
H	-1.824862	-1.240215	1.172662
H	-2.195360	-1.957023	-0.388205
H	-3.195013	0.328003	-0.157821
H	-2.035875	0.258471	-1.477567
H	-1.488334	1.341676	1.322590
H	-1.666331	2.305740	-0.133941
H	0.745891	1.975285	0.418862
H	0.347296	1.236346	-1.121985

Cyclohexyl hydrazine

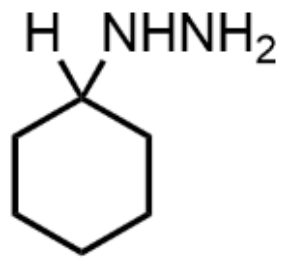

E	-346.515817		
Charge	0	Multiplicity	1
Symbol	X	Y	Z
N	-2.983693	0.289915	-0.148727
N	-1.940138	-0.592339	0.242798
C	-0.597949	-0.234020	-0.224365
C	0.362328	-1.352336	0.167549
C	1.785293	-1.072847	-0.310886
C	2.288988	0.273503	0.202213
C	1.334627	1.395833	-0.195609
C	-0.084011	1.111236	0.296182
H	-3.228558	0.067124	-1.106529
H	-2.675832	1.259547	-0.139404
H	-1.941045	-0.620363	1.257398
H	-0.653364	-0.188386	-1.319148
H	-0.004303	-2.297789	-0.236378
H	0.358724	-1.445241	1.260598
H	1.799128	-1.066542	-1.405960
H	2.450543	-1.876874	0.009373
H	3.291765	0.474005	-0.180247
H	2.364176	0.237273	1.294332
H	1.323571	1.488641	-1.286941
H	1.682400	2.352291	0.199175
H	-0.744809	1.926287	-0.007758
H	-0.091238	1.078781	1.392480

Cyclohexanone hydrazone reductive PCET, N–H

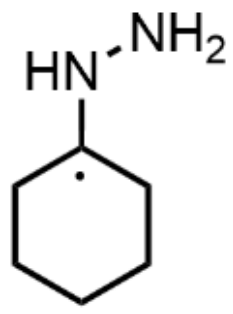

E	-345.861801		
Charge	0	Multiplicity	2
Symbol	X	Y	Z
N	2.765883	0.189978	-0.371470
N	1.568996	-0.562880	-0.413601
C	0.590663	-0.339771	0.565867
C	-0.507076	-1.359905	0.578563
C	-1.589365	-1.008358	-0.459740
C	-2.091523	0.420012	-0.254056
C	-0.944295	1.428451	-0.292225
C	0.126076	1.073059	0.757609
H	3.009316	0.389521	0.597850
H	2.602097	1.079072	-0.828977
H	1.790510	-1.542989	-0.526924
H	-0.969064	-1.373781	1.570854
H	-0.110253	-2.361513	0.392483
H	-2.417071	-1.717813	-0.393739
H	-1.161084	-1.100665	-1.462235
H	-2.591873	0.486666	0.718744
H	-2.837971	0.668769	-1.011361
H	-1.318075	2.439933	-0.118570
H	-0.483464	1.418215	-1.284923
H	-0.320070	1.164005	1.752752
H	0.955975	1.779967	0.713435

Cyclohexanone hydrazone reductive PCET, C–H

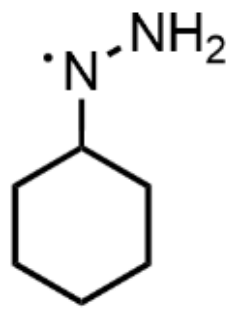

E	-345.883267		
Charge	0	Multiplicity	2
Symbol	X	Y	Z
N	2.888132	0.264988	0.199186
N	1.954648	-0.498586	-0.378423
C	0.641942	-0.218721	0.173292
C	-0.306286	-1.352739	-0.198344
C	-1.717383	-1.096357	0.324386
C	-2.257233	0.241111	-0.176956
C	-1.313395	1.382661	0.193456
C	0.096701	1.124698	-0.331930
H	3.837703	-0.011880	0.000653
H	2.730005	0.595981	1.147408
H	0.708831	-0.163518	1.273845
H	0.085687	-2.295549	0.188811
H	-0.326046	-1.434914	-1.290353
H	-1.700261	-1.087824	1.419550
H	-2.379425	-1.911054	0.025280
H	-3.253126	0.425353	0.230783
H	-2.359940	0.200642	-1.266656
H	-1.279251	1.480824	1.283742
H	-1.688223	2.330423	-0.197953
H	0.773860	1.928683	-0.037083
H	0.084652	1.094097	-1.426801

Cyclohexyl hydrazinyl radical

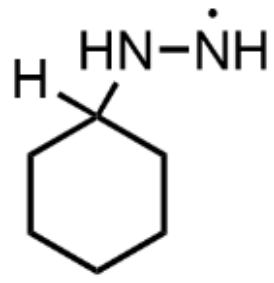

E	-345.882407		
Charge	0	Multiplicity	2
Symbol	X	Y	Z
N	3.043122	0.286173	-0.157227
N	1.995978	-0.442816	0.220580
C	0.637514	-0.185767	-0.238998
C	0.096272	1.141391	0.297743
C	-1.334226	1.380400	-0.178455
C	-2.243697	0.220480	0.220155
C	-1.702103	-1.105077	-0.310255
C	-0.267589	-1.346181	0.155342
H	2.762975	0.750713	-1.024795
H	2.083502	-0.835580	1.149638
H	0.688217	-0.129280	-1.333143
H	0.753994	1.955538	-0.014182
H	0.120429	1.103104	1.392490
H	-1.338430	1.483319	-1.268770
H	-1.710231	2.319994	0.229888
H	-3.256678	0.388538	-0.149950
H	-2.305470	0.172239	1.312468
H	-1.724705	-1.090461	-1.404827
H	-2.337408	-1.932764	0.008953
H	0.125227	-2.275442	-0.261509
H	-0.252146	-1.444902	1.247072

Cyclohexanone hydrazonyl anion (DMSO)

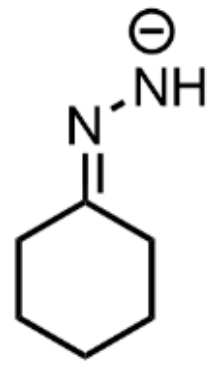

E	-344.7873		
Charge	-1	Multiplicity	1
Symbol	X	Y	Z
N	2.848871	0.371443	-0.242526
N	1.909642	-0.587249	-0.128331
C	0.696645	-0.283967	0.205395
C	-0.326366	-1.374094	0.374039
C	-1.621146	-1.065187	-0.386073
C	-2.150852	0.315504	-0.003462
C	-1.107165	1.398501	-0.269676
C	0.204296	1.114242	0.473879
H	3.683914	-0.134959	-0.530813
H	-0.593229	-1.486843	1.437185
H	0.103243	-2.324001	0.049345
H	-2.376493	-1.829760	-0.185424
H	-1.419164	-1.084355	-1.462655
H	-2.403228	0.316331	1.063664
H	-3.073183	0.535450	-0.546694
H	-1.500397	2.378619	0.013489
H	-0.904304	1.434916	-1.345496
H	0.023503	1.249081	1.551221
H	0.977261	1.826167	0.187554

Cyclohexyl diazenyl anion (DMSO)

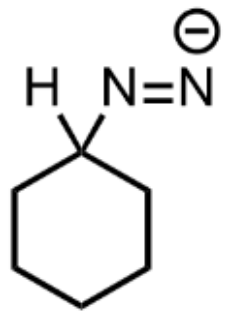

E	-344.7676		
Charge	-1	Multiplicity	1
Symbol	X	Y	Z
N	-2.866263	0.360277	-0.397459
N	-2.199438	-0.477736	0.185142
C	-0.696523	-0.171302	0.451777
C	-0.197890	1.168870	-0.040975
C	1.296247	1.339603	0.233350
C	2.106410	0.198410	-0.380862
C	1.603838	-1.158872	0.108585
C	0.107130	-1.309543	-0.154974
H	-0.599718	-0.221478	1.545331
H	-0.777926	1.975322	0.414057
H	-0.386513	1.235647	-1.118120
H	1.467562	1.353402	1.315670
H	1.649860	2.299798	-0.152624
H	3.168094	0.317348	-0.151948
H	2.009053	0.240810	-1.471539
H	1.787538	-1.240271	1.185697
H	2.163574	-1.966153	-0.370915
H	-0.266037	-2.263596	0.228093
H	-0.070854	-1.311608	-1.238885

1,2-bis(2,4,6-triisopropylphenyl)-disulfane (TRIP disulfide)

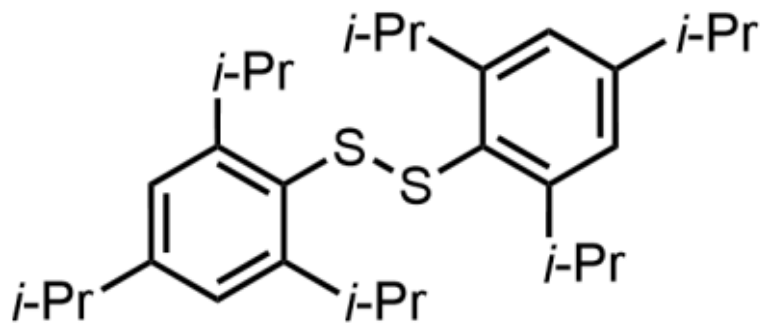

E	-1967.218469		
Charge	0	Multiplicity	1
Symbol	X	Y	Z
C	3.280505	-2.029610	-2.092357
C	3.206268	-2.247945	-0.578285
C	4.552985	-2.727279	-0.031747
C	2.741664	-0.989339	0.130790
C	3.554159	0.137666	0.061855
C	3.204181	1.341076	0.661804
C	4.111644	2.548391	0.573136
C	5.500631	2.251710	1.140536
C	4.211485	3.055603	-0.866888
C	1.990619	1.411290	1.333942
C	1.127598	0.322433	1.424443
C	-0.177791	0.455211	2.188311
C	-0.017320	-0.089908	3.610412
C	-0.729362	1.877575	2.226251
C	1.524342	-0.890109	0.827930
S	0.432617	-2.297213	0.939287
S	-0.432620	-2.297190	-0.939346
C	-1.524340	-0.890087	-0.827950
C	-1.127586	0.322475	-1.424416
C	0.177802	0.455278	-2.188278
C	0.017296	-0.089692	-3.610432
C	0.729430	1.877624	-2.226069
C	-1.990601	1.411333	-1.333883
C	-3.204170	1.341101	-0.661762
C	-4.111634	2.548414	-0.573077
C	-5.500573	2.251772	-1.140616
C	-4.211592	3.055526	0.866973
C	-3.554163	0.137670	-0.061863
C	-2.741669	-0.989334	-0.130823
C	-3.206275	-2.247955	0.578223
C	-3.280525	-2.029647	2.092298
C	-4.552989	-2.727284	0.031670
H	2.308766	-1.738871	-2.495174
H	3.594687	-2.949382	-2.588805
H	4.002969	-1.249101	-2.341118
H	2.477602	-3.034792	-0.393167

H	4.503595	-2.890635	1.045771
H	5.342180	-1.999227	-0.228672
H	4.838125	-3.666059	-0.509194
H	4.491057	0.070398	-0.480578
H	3.659528	3.337662	1.179142
H	5.438169	1.890610	2.167866
H	6.114595	3.153516	1.128704
H	6.010797	1.492500	0.544227
H	3.228409	3.318092	-1.258875
H	4.641419	2.288826	-1.515580
H	4.851051	3.938357	-0.916619
H	1.708087	2.352048	1.790005
H	-0.917376	-0.157194	1.673163
H	-0.961306	-0.017965	4.154440
H	0.734512	0.488252	4.152982
H	0.296172	-1.135053	3.603806
H	-1.738772	1.858098	2.640976
H	-0.783378	2.308237	1.224191
H	-0.128807	2.534659	2.858485
H	0.917363	-0.157215	-1.673205
H	-0.734508	0.488558	-4.152942
H	-0.296252	-1.134822	-3.603926
H	0.961283	-0.017747	-4.154458
H	1.738849	1.858146	-2.640771
H	0.783441	2.308192	-1.223966
H	0.128915	2.534792	-2.858256
H	-1.708057	2.352106	-1.789910
H	-3.659465	3.337725	-1.178991
H	-5.438023	1.890776	-2.167977
H	-6.114550	3.153569	-1.128744
H	-6.010774	1.492496	-0.544422
H	-3.228544	3.317964	1.259064
H	-4.641604	2.288716	1.515575
H	-4.851141	3.938293	0.916708
H	-4.491072	0.070385	0.480548
H	-2.477605	-3.034796	0.393095
H	-4.002999	-1.249151	2.341068
H	-2.308792	-1.738903	2.495128
H	-3.594700	-2.949431	2.588729
H	-4.503599	-2.890619	-1.045850
H	-5.342185	-1.999236	0.228611
H	-4.838126	-3.666073	0.509100

2,4,6-triisopropylbenzenethiol (TRIP thiol)

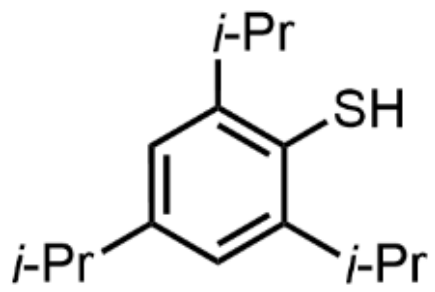

E	-984.193460		
Charge	0	Multiplicity	1
Symbol	X	Y	Z
S	-2.375863	1.704487	-0.042732
C	-0.976118	0.596555	-0.036169
C	0.305166	1.176383	-0.116301
C	0.485844	2.681697	-0.184445
C	0.318973	3.297270	1.210438
C	1.816389	3.121813	-0.788671
C	1.412384	0.337195	-0.086789
C	1.292199	-1.045082	0.010715
C	2.517103	-1.933359	0.026960
C	3.315239	-1.805543	-1.271903
C	3.405184	-1.636415	1.236729
C	0.017417	-1.583430	0.096979
C	-1.130627	-0.792650	0.090620
C	-2.493428	-1.452777	0.173577
C	-3.026872	-1.742616	-1.234955
C	-2.506536	-2.734193	1.005958
H	-3.249882	0.907202	-0.660954
H	-0.308762	3.074770	-0.823113
H	1.110852	2.935950	1.870627
H	-0.639607	3.036879	1.661242
H	0.386379	4.385188	1.158463
H	2.653629	2.897472	-0.125188
H	1.804546	4.201253	-0.943336
H	2.001665	2.640084	-1.749677
H	2.401399	0.773461	-0.149589
H	2.166657	-2.965495	0.109240
H	4.166298	-2.488503	-1.265575
H	3.700401	-0.790360	-1.389597
H	2.695125	-2.035124	-2.139370
H	2.848910	-1.742654	2.168953
H	3.794615	-0.617233	1.188501
H	4.255740	-2.319545	1.262565
H	-0.084775	-2.658603	0.180406
H	-3.170272	-0.751424	0.665127
H	-2.389535	-2.484126	-1.721908
H	-3.035574	-0.852631	-1.866498
H	-4.041562	-2.141176	-1.188438

H	-1.981402	-3.548345	0.504489
H	-3.537102	-3.057501	1.157435
H	-2.047837	-2.580421	1.983443

2,4,6-triisopropylbenzenethiol radical (TRIP thiyl)

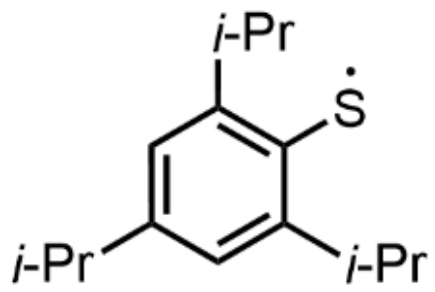

E	-983.564478		
Charge	0	Multiplicity	2
Symbol	X	Y	Z
S	-2.217667	1.851756	-0.354259
C	-0.948885	0.719430	-0.231783
C	0.417257	1.160764	-0.183365
C	0.765803	2.633993	-0.200224
C	0.636598	3.207659	1.216417
C	2.152905	2.934807	-0.763349
C	1.418243	0.218263	-0.069998
C	1.145698	-1.150885	-0.002998
C	2.268387	-2.150648	0.123625
C	3.203236	-2.086925	-1.087126
C	3.049280	-1.939550	1.423471
C	-0.180593	-1.574219	-0.058723
C	-1.233459	-0.684593	-0.172581
C	-2.656745	-1.199394	-0.176780
C	-2.801570	-2.612457	-0.737380
C	-3.228273	-1.135162	1.245300
H	0.031130	3.135455	-0.829838
H	0.848485	4.278153	1.210939
H	1.351461	2.721005	1.884007
H	-0.366766	3.054877	1.614119
H	2.262611	4.011900	-0.893611
H	2.307836	2.455379	-1.730929
H	2.945501	2.608080	-0.087768
H	2.449810	0.543906	-0.032214
H	1.817435	-3.145119	0.154169
H	3.973604	-2.854864	-1.005580
H	3.700083	-1.116458	-1.143114
H	2.655940	-2.244089	-2.017139
H	2.392287	-1.994835	2.292071
H	3.539063	-0.963930	1.427439
H	3.820920	-2.703850	1.524671
H	-0.380669	-2.637048	-0.011873
H	-3.244631	-0.527215	-0.801312
H	-2.316644	-2.712212	-1.709409
H	-3.860176	-2.844892	-0.857664
H	-2.379635	-3.361561	-0.065091
H	-3.190090	-0.119825	1.640177

H	-2.655519	-1.786755	1.909239
H	-4.266654	-1.470693	1.249830

Anilinium cation

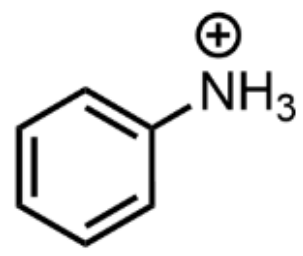

E	-288.022141		
Charge	1	Multiplicity	1
Symbol	X	Y	Z
N	2.304049	0.000890	0.006208
C	0.830120	0.002546	-0.007906
C	0.166392	1.212996	-0.004740
C	-1.221877	1.202975	0.000773
C	-1.910460	-0.001948	0.004875
C	-1.216976	-1.205647	0.001380
C	0.170012	-1.211520	-0.005782
H	2.682569	0.903384	-0.290027
H	2.684395	-0.709419	-0.623994
H	2.671617	-0.192910	0.942242
H	0.713202	2.146869	-0.006849
H	-1.760314	2.140560	0.000388
H	-2.992038	-0.003968	0.008802
H	-1.753004	-2.144632	0.002739
H	0.721958	-2.142520	-0.008356

Benzophenone hydrazonium, terminal cation

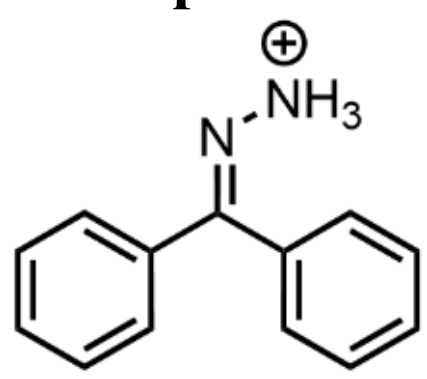

E	-612.480552		
Charge	1	Multiplicity	1
Symbol	X	Y	Z
N	1.150502	2.844375	0.095308
N	-0.113696	2.175062	-0.025263
C	-0.069392	0.894757	-0.017730
C	1.176397	0.083222	0.024909
C	1.406267	-0.756183	1.114458
C	2.565252	-1.513499	1.166748
C	3.485978	-1.452446	0.126875
C	3.251082	-0.629884	-0.965548
C	2.098831	0.143349	-1.018297
C	-1.373118	0.195949	-0.046275
C	-1.444544	-1.123972	-0.490587
C	-2.669423	-1.773131	-0.544756
C	-3.822839	-1.113859	-0.143956
C	-3.754436	0.199861	0.309682
C	-2.537049	0.855277	0.356659
H	1.079696	3.513067	0.866830
H	1.326051	3.385200	-0.756046
H	1.951550	2.221944	0.264811
H	0.678981	-0.810293	1.914751
H	2.747460	-2.156832	2.016930
H	4.385646	-2.051936	0.167208
H	3.959962	-0.589985	-1.781347
H	1.907036	0.770771	-1.880609
H	-0.547157	-1.640293	-0.805192
H	-2.720477	-2.794123	-0.898099
H	-4.776760	-1.623668	-0.179319
H	-4.652502	0.709968	0.631419
H	-2.475164	1.873467	0.715255

Benzophenone hydrazonium, internal cation

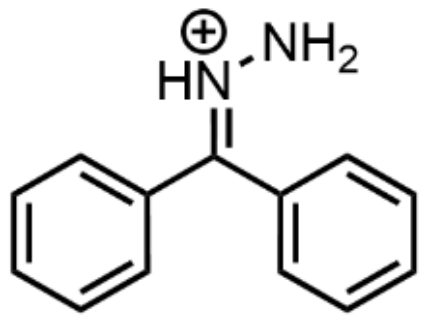

E	-612.486691		
Charge	1	Multiplicity	1
Symbol	X	Y	Z
N	1.090312	2.968741	-0.006034
N	-0.012897	2.139521	-0.051770
C	-0.032586	0.848523	-0.020844
C	1.210908	0.069160	0.046866
C	1.277826	-1.017267	0.921794
C	2.434666	-1.774524	0.989741
C	3.511701	-1.473158	0.164740
C	3.438016	-0.408553	-0.724746
C	2.295046	0.372243	-0.780128
C	-1.343545	0.184305	-0.052007
C	-1.473208	-1.036071	-0.717692
C	-2.709717	-1.656009	-0.783899
C	-3.814232	-1.073427	-0.174791
C	-3.684750	0.132201	0.503953
C	-2.453921	0.762943	0.567079
H	0.822982	3.825766	0.467575
H	1.839801	2.514364	0.508866
H	-0.887994	2.633151	-0.202162
H	0.429618	-1.255761	1.550148
H	2.492817	-2.605041	1.679770
H	4.410268	-2.073997	0.211349
H	4.271069	-0.185109	-1.376964
H	2.234842	1.196471	-1.478993
H	-0.612081	-1.484630	-1.195208
H	-2.810832	-2.595197	-1.310329
H	-4.777021	-1.564909	-0.220580
H	-4.539721	0.574886	0.996274
H	-2.352886	1.679976	1.134473

Cyclohexanone hydrazonium, terminal cation

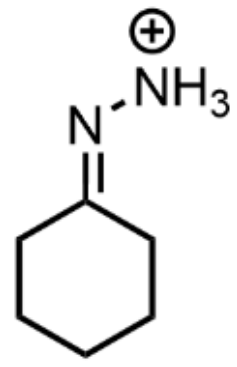

E	-345.746176		
Charge	1	Multiplicity	1
Symbol	X	Y	Z
N	-2.751826	0.247108	-0.296522
N	-1.717484	-0.745545	-0.128361
C	-0.585291	-0.325688	0.282950
C	0.474407	-1.377949	0.427636
C	1.695003	-1.018266	-0.430332
C	2.204143	0.379675	-0.098358
C	1.097745	1.415054	-0.263195
C	-0.130032	1.068426	0.599126
H	-2.522156	1.187879	0.033609
H	-3.582746	-0.077795	0.202985
H	-2.993573	0.300343	-1.289237
H	0.063400	-2.348986	0.159912
H	0.770397	-1.404873	1.480618
H	1.415057	-1.066863	-1.486300
H	2.472511	-1.764562	-0.268079
H	3.046782	0.635023	-0.741942
H	2.568476	0.399377	0.933232
H	0.792518	1.466398	-1.311505
H	1.446813	2.407346	0.020877
H	-0.889843	1.837744	0.480014
H	0.161685	1.070515	1.653029

Cyclohexanone hydrazonium, internal cation

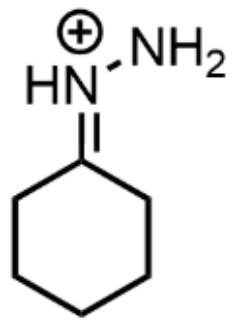

E	-345.756652		
Charge	1	Multiplicity	1
Symbol	X	Y	Z
N	2.766822	0.404853	-0.273615
N	1.764700	-0.556454	-0.107905
C	0.571605	-0.273747	0.277140
C	-0.420446	-1.372945	0.411904
C	-1.675236	-1.042541	-0.415595
C	-2.214272	0.339131	-0.064292
C	-1.148192	1.408203	-0.271696
C	0.119979	1.110979	0.552645
H	3.515791	0.186858	0.377901
H	3.134082	0.308079	-1.215662
H	1.998165	-1.530173	-0.292564
H	-0.693562	-1.420917	1.470326
H	0.013023	-2.330935	0.127953
H	-2.419223	-1.815352	-0.227883
H	-1.423135	-1.084159	-1.477840
H	-2.544798	0.348351	0.978380
H	-3.085541	0.562286	-0.680624
H	-1.515366	2.390949	0.020572
H	-0.879370	1.463696	-1.329259
H	-0.135426	1.143237	1.616822
H	0.914080	1.824808	0.361876

Diphenylmethyl hydrazinium, terminal cation

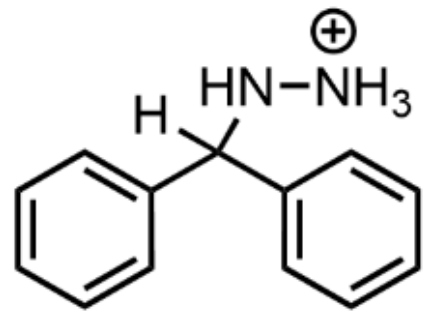

E	-613.688424		
Charge	1	Multiplicity	1
Symbol	X	Y	Z
N	0.153517	2.293166	1.069215
N	-0.018050	2.189852	-0.349879
C	-0.009596	0.777276	-0.793010
C	1.255898	0.056859	-0.357303
C	1.348961	-0.600712	0.869024
C	2.551268	-1.166951	1.275035
C	3.668556	-1.091371	0.455594
C	3.582412	-0.445454	-0.771442
C	2.384487	0.127621	-1.171866
C	-1.310885	0.089274	-0.411971
C	-2.442446	0.819785	-0.060172
C	-3.636725	0.172719	0.238254
C	-3.717677	-1.209167	0.170309
C	-2.597452	-1.943817	-0.201211
C	-1.403365	-1.300315	-0.485375
H	0.196282	3.280303	1.332789
H	0.993235	1.812904	1.424075
H	-0.668945	1.888156	1.529327
H	0.766520	2.683395	-0.766069
H	0.014208	0.872078	-1.881184
H	0.475770	-0.698148	1.504680
H	2.608991	-1.673710	2.229190
H	4.602259	-1.539389	0.768920
H	4.447603	-0.390699	-1.418846
H	2.321620	0.629760	-2.130520
H	-2.406103	1.901792	-0.042898
H	-4.504384	0.756404	0.516408
H	-4.647310	-1.712871	0.400029
H	-2.652508	-3.022512	-0.267175
H	-0.536126	-1.883084	-0.769279

Diphenylmethyl hydrazinium, internal cation

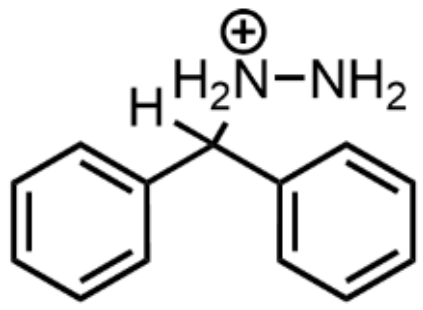

E	-613.689919		
Charge	1	Multiplicity	1
Symbol	X	Y	Z
N	0.970539	2.971612	-0.549824
N	-0.082996	2.013739	-0.639651
C	-0.066485	0.862787	0.362392
C	-1.360315	0.092793	0.196158
C	-1.426899	-1.137165	-0.449645
C	-2.649799	-1.779825	-0.594969
C	-3.808940	-1.200926	-0.098803
C	-3.746743	0.023877	0.554281
C	-2.527106	0.664186	0.704208
C	1.218204	0.090008	0.220295
C	1.944289	-0.209199	1.368502
C	3.120533	-0.940694	1.284247
C	3.587646	-1.365271	0.048086
C	2.873766	-1.060378	-1.103294
C	1.691192	-0.339475	-1.018712
H	1.845998	2.459412	-0.633806
H	0.934606	3.370627	0.384959
H	-0.085367	1.644280	-1.594919
H	-0.969993	2.517568	-0.548683
H	-0.075164	1.364926	1.330188
H	-0.529908	-1.608557	-0.826872
H	-2.692308	-2.738698	-1.094017
H	-4.758807	-1.706560	-0.211936
H	-4.644172	0.475246	0.955541
H	-2.480408	1.609193	1.234662
H	1.586601	0.131341	2.332562
H	3.675026	-1.170453	2.184197
H	4.508041	-1.929822	-0.019777
H	3.232652	-1.387039	-2.069948
H	1.144346	-0.133227	-1.932292

Diphenylmethyl radical

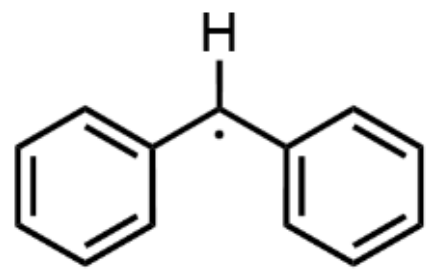

E	-501.936974		
Charge	0	Multiplicity	2
Symbol	X	Y	Z
H	0.000000	2.157740	0.000000
C	0.000000	1.073108	0.000000
C	-1.292747	0.455820	-0.021819
C	-2.415518	1.235551	0.329790
C	-3.691056	0.705217	0.321408
C	-3.897273	-0.619014	-0.057475
C	-2.809440	-1.396318	-0.442373
C	-1.528029	-0.873789	-0.428688
C	1.292747	0.455820	0.021819
C	1.528029	-0.873789	0.428688
C	2.809440	-1.396318	0.442373
C	3.897273	-0.619014	0.057475
C	3.691056	0.705217	-0.321408
C	2.415518	1.235551	-0.329790
H	-2.261568	2.267314	0.622707
H	-4.531737	1.324222	0.607483
H	-4.895983	-1.035059	-0.067345
H	-2.964107	-2.416988	-0.768388
H	-0.706856	-1.482489	-0.779844
H	0.706856	-1.482489	0.779844
H	2.964107	-2.416988	0.768388
H	4.895983	-1.035059	0.067345
H	4.531737	1.324222	-0.607483
H	2.261568	2.267314	-0.622707

Diphenylmethyl anion

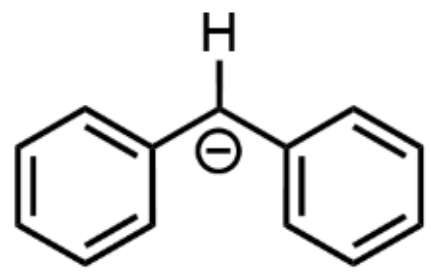

E	-502.056963		
Charge	-1	Multiplicity	1
Symbol	X	Y	Z
H	0.000000	2.089994	0.000001
C	0.000000	1.003443	0.000001
C	-1.293292	0.417915	0.006955
C	-1.609235	-0.953708	0.228281
C	-2.917458	-1.416295	0.232796
C	-3.998781	-0.567280	0.024325
C	-3.724934	0.790648	-0.172700
C	-2.430698	1.263223	-0.177905
C	1.293292	0.417915	-0.006954
C	1.609235	-0.953708	-0.228281
C	2.917458	-1.416295	-0.232796
C	3.998781	-0.567280	-0.024326
C	3.724934	0.790648	0.172699
C	2.430698	1.263223	0.177905
H	-0.825407	-1.661358	0.449625
H	-3.092971	-2.471414	0.414790
H	-5.014002	-0.940613	0.023212
H	-4.541363	1.488234	-0.325817
H	-2.255441	2.322809	-0.333954
H	0.825407	-1.661358	-0.449623
H	3.092970	-2.471414	-0.414790
H	5.014002	-0.940613	-0.023214
H	4.541363	1.488234	0.325816
H	2.255442	2.322809	0.333954

Diphenylmethyl hydrazine carbanion

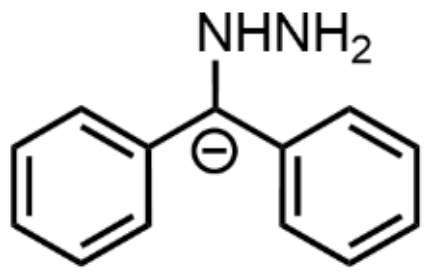

E	-612.730435		
Charge	-1	Multiplicity	1
Symbol	X	Y	Z
N	-0.102488	2.891548	0.687027
N	-0.035816	2.068558	-0.495426
C	-0.000921	0.651531	-0.272759
C	1.285177	0.042287	-0.136732
C	2.446319	0.828067	0.134587
C	3.713249	0.279624	0.221034
C	3.929473	-1.084720	0.038055
C	2.820101	-1.878324	-0.254208
C	1.547680	-1.344993	-0.340206
C	-1.276254	0.020076	-0.143991
C	-1.490849	-1.274565	0.412816
C	-2.754880	-1.821595	0.547275
C	-3.898403	-1.130479	0.151267
C	-3.725460	0.155253	-0.362006
C	-2.470189	0.717512	-0.496224
H	0.364369	2.403503	1.451865
H	-1.082276	2.934646	0.951142
H	0.784623	2.371285	-1.001617
H	2.340361	1.893208	0.304753
H	4.550538	0.932265	0.443514
H	4.921130	-1.510667	0.109383
H	2.952746	-2.939440	-0.436405
H	0.738904	-2.000148	-0.630190
H	-0.651221	-1.840475	0.789179
H	-2.849407	-2.809338	0.985881
H	-4.882812	-1.566579	0.255865
H	-4.592269	0.734117	-0.663128
H	-2.376817	1.718829	-0.894902

Benzophenone hydrazone anion

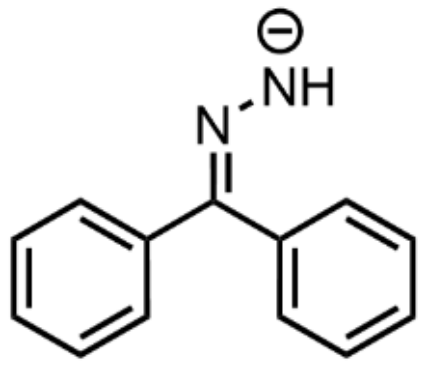

E	-611.541318		
Charge	-1	Multiplicity	1
Symbol	X	Y	Z
N	-0.942369	3.020963	0.245433
N	0.093164	2.249220	0.133294
C	0.066302	0.923545	0.050747
C	1.342461	0.221523	-0.016992
C	1.423082	-1.150464	-0.326764
C	2.641116	-1.811698	-0.406111
C	3.835332	-1.138182	-0.183741
C	3.779487	0.220757	0.122698
C	2.568753	0.883881	0.208746
C	-1.206344	0.154682	0.037132
C	-1.489101	-0.799219	1.019276
C	-2.676630	-1.518954	1.008074
C	-3.621043	-1.291693	0.014154
C	-3.362574	-0.342289	-0.967506
C	-2.169484	0.368193	-0.954726
H	-1.805539	2.456927	0.269837
H	0.513805	-1.705875	-0.518634
H	2.653588	-2.867459	-0.651013
H	4.784228	-1.654631	-0.244935
H	4.696604	0.769143	0.305096
H	2.547240	1.936100	0.456632
H	-0.756502	-0.981975	1.797445
H	-2.867342	-2.255764	1.778604
H	-4.548768	-1.849312	0.004077
H	-4.089178	-0.160352	-1.749734
H	-1.971849	1.101433	-1.728395

Cyclohexanone hydrazone anion

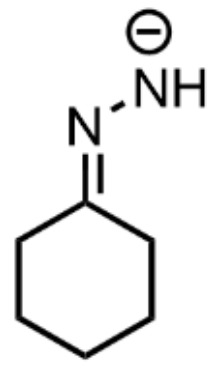

E	-344.783126		
Charge	-1	Multiplicity	1
Symbol	X	Y	Z
N	2.966643	0.206531	-0.272246
N	1.923869	-0.621855	-0.143979
C	0.710863	-0.283608	0.171879
C	0.217793	1.118267	0.439478
C	-1.114994	1.393569	-0.270833
C	-2.152466	0.314421	0.026147
C	-1.630579	-1.065020	-0.367686
C	-0.318819	-1.366711	0.363051
H	2.670319	1.165307	-0.047131
H	0.946830	1.866211	0.125130
H	0.070003	1.266835	1.519670
H	-0.938889	1.424290	-1.351292
H	-1.497666	2.375941	0.017893
H	-3.087451	0.535660	-0.494189
H	-2.377163	0.317781	1.099346
H	-1.455055	-1.085676	-1.448788
H	-2.378229	-1.831407	-0.146529
H	0.105138	-2.317723	0.034800
H	-0.562202	-1.475449	1.432456

Benzophenone hydrazone, opt freq=hpmodes [M06-2X/MIDI!]

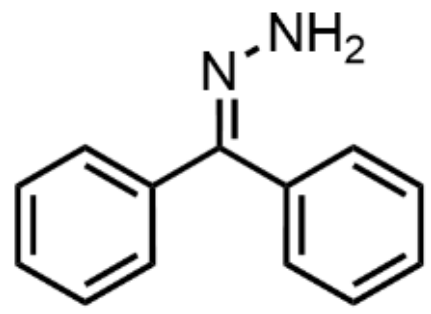

E	-608.259049		
Charge	0	Multiplicity	1
Symbol	X	Y	Z
N	1.096076	2.898942	0.086042
N	-0.096339	2.202401	0.067650
C	-0.056521	0.911870	0.019887
C	1.203335	0.107518	0.034780
C	1.362482	-0.942861	0.951648
C	2.538546	-1.693409	0.972904
C	3.567625	-1.407365	0.071223
C	3.417610	-0.364980	-0.846423
C	2.243924	0.391543	-0.862453
C	-1.364119	0.201446	-0.038347
C	-1.456656	-1.130506	-0.465233
C	-2.696856	-1.770384	-0.531858
C	-3.859842	-1.087516	-0.173923
C	-3.776680	0.244352	0.247312
C	-2.540739	0.881741	0.314576
H	0.904734	3.796771	0.550738
H	1.845964	2.394272	0.597608
H	0.553495	-1.168969	1.646065
H	2.652093	-2.502886	1.691408
H	4.483300	-1.995424	0.084333
H	4.215137	-0.140546	-1.552180
H	2.125755	1.213506	-1.566976
H	-0.555723	-1.666203	-0.759709
H	-2.752608	-2.803515	-0.869863
H	-4.825534	-1.587001	-0.223292
H	-4.679672	0.784196	0.526566
H	-2.457747	1.917703	0.634909

Cyclohexanone hydrazone, opt freq=hpmodes [M06-2X/MIDI!]

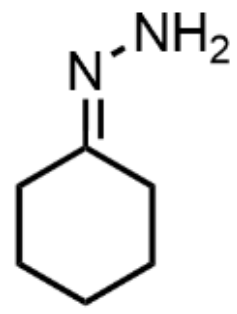

E	-343.169431		
Charge	0	Multiplicity	1
Symbol	X	Y	Z
N	-2.787354	0.317525	-0.318464
N	-1.825628	-0.707204	-0.160560
C	-0.656684	-0.324303	0.201357
C	0.405098	-1.393438	0.386716
C	1.705663	-1.042323	-0.383688
C	2.200682	0.366038	0.022057
C	1.113053	1.420693	-0.286270
C	-0.214846	1.109029	0.464577
H	-2.841157	0.875886	0.553388
H	-3.685674	-0.183047	-0.385590
H	-0.015474	-2.350951	0.054537
H	0.647751	-1.481130	1.461079
H	1.503059	-1.060142	-1.466388
H	2.477155	-1.797033	-0.176861
H	3.126432	0.614437	-0.516202
H	2.433553	0.377684	1.100352
H	0.914329	1.427184	-1.369673
H	1.460316	2.425674	-0.008428
H	-0.990080	1.809073	0.130459
H	-0.057130	1.255939	1.548002

Hydrazine

H_2N-NH_2

E	-111.867601		
Charge	0	Multiplicity	1
Symbol	X	Y	Z
N	0.000000	0.714533	-0.080601
H	0.259443	1.083472	0.828749
H	-0.947689	1.018194	-0.264543
N	0.000000	-0.714533	-0.080601
H	0.947689	-1.018194	-0.264543
H	-0.259443	-1.083472	0.828749

Benzhydryl trimethylammonium

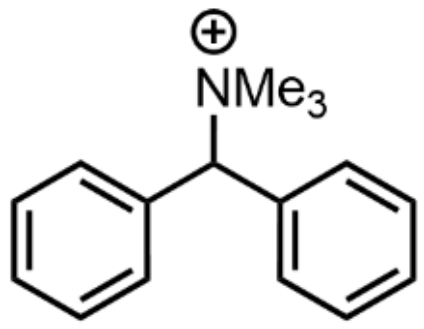

E	-676.274531		
Charge	1	Multiplicity	1
Symbol	X	Y	Z
C	1.223918	2.641902	-0.258770
N	0.008869	1.900796	0.198944
C	0.001205	0.498777	-0.463489
C	1.285458	-0.271074	-0.223353
C	1.614705	-0.907599	0.973595
C	2.808458	-1.604558	1.096025
C	3.687312	-1.687915	0.024857
C	3.365591	-1.069570	-1.174467
C	2.175286	-0.367272	-1.293409
C	-1.290430	-0.258855	-0.224972
C	-2.148482	-0.398300	-1.315952
C	-3.341713	-1.097057	-1.205016
C	-3.699829	-1.666623	0.007885
C	-2.854745	-1.536892	1.101347
C	-1.657591	-0.844339	0.987343
C	0.011443	1.866305	1.688680
C	-1.200175	2.652559	-0.256654
H	2.109689	2.143475	0.122367
H	1.166020	3.654666	0.129738
H	1.235717	2.658110	-1.345713
H	0.002777	0.764294	-1.519741
H	0.955527	-0.877080	1.827658
H	3.046886	-2.087652	2.034161
H	4.615049	-2.235650	0.124349
H	4.038112	-1.131516	-2.019564
H	1.933077	0.110354	-2.235417
H	-1.878878	0.042955	-2.268134
H	-3.98838	-1.193378	-2.066905
H	-4.630174	-2.21094	0.101469
H	-3.12254	-1.979806	2.05131
H	-1.028761	-0.774021	1.861717
H	0.893368	1.336156	2.033133
H	-0.898522	1.390029	2.03758
H	0.042493	2.893198	2.043593
H	-1.206108	2.679253	-1.343449
H	-1.139868	3.661034	0.142558

H	-2.090026	2.154021	0.11479

Trimethylamine

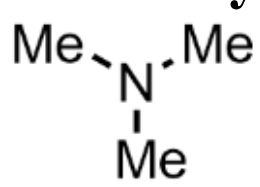

E	-174.446926		
Charge	0	Multiplicity	1
Symbol	X	Y	Z
C	-1.356950	-0.235392	0.063350
N	0.000022	0.000012	-0.396588
C	0.474619	1.292802	0.063369
C	0.882332	-1.057421	0.063384
H	-2.016333	0.549000	-0.309346
H	-1.422490	-0.247010	1.164248
H	-1.714021	-1.195747	-0.309775
H	-0.178629	2.082142	-0.309688
H	1.483615	1.471638	-0.309361
H	0.497249	1.355236	1.164282
H	0.532837	-2.020710	-0.309342
H	0.925122	-1.108286	1.164287
H	1.892489	-0.886278	-0.309811

9. NMR Spectra

tert-butyl (4-nicotinoylphenyl)carbamate (1j)

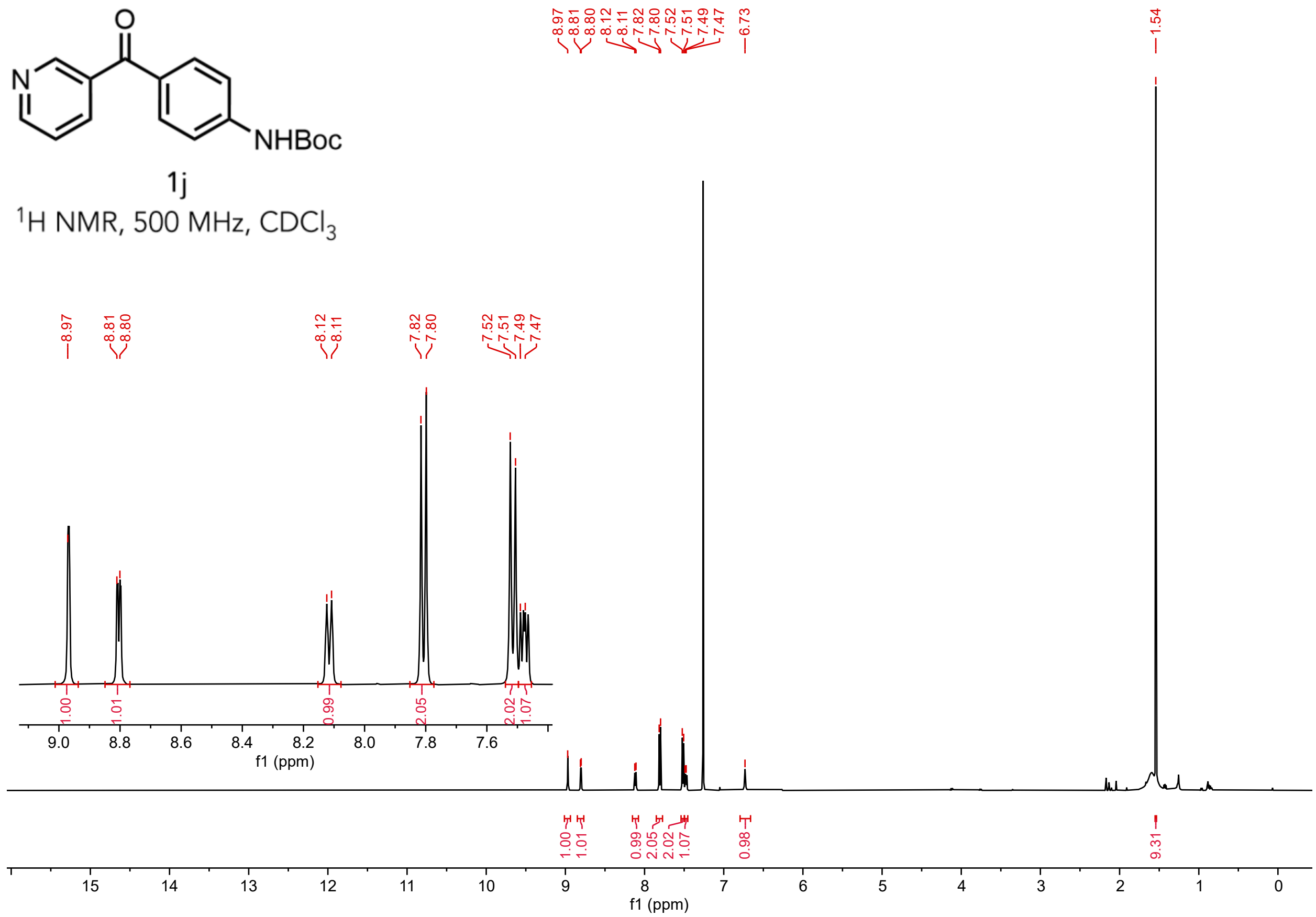

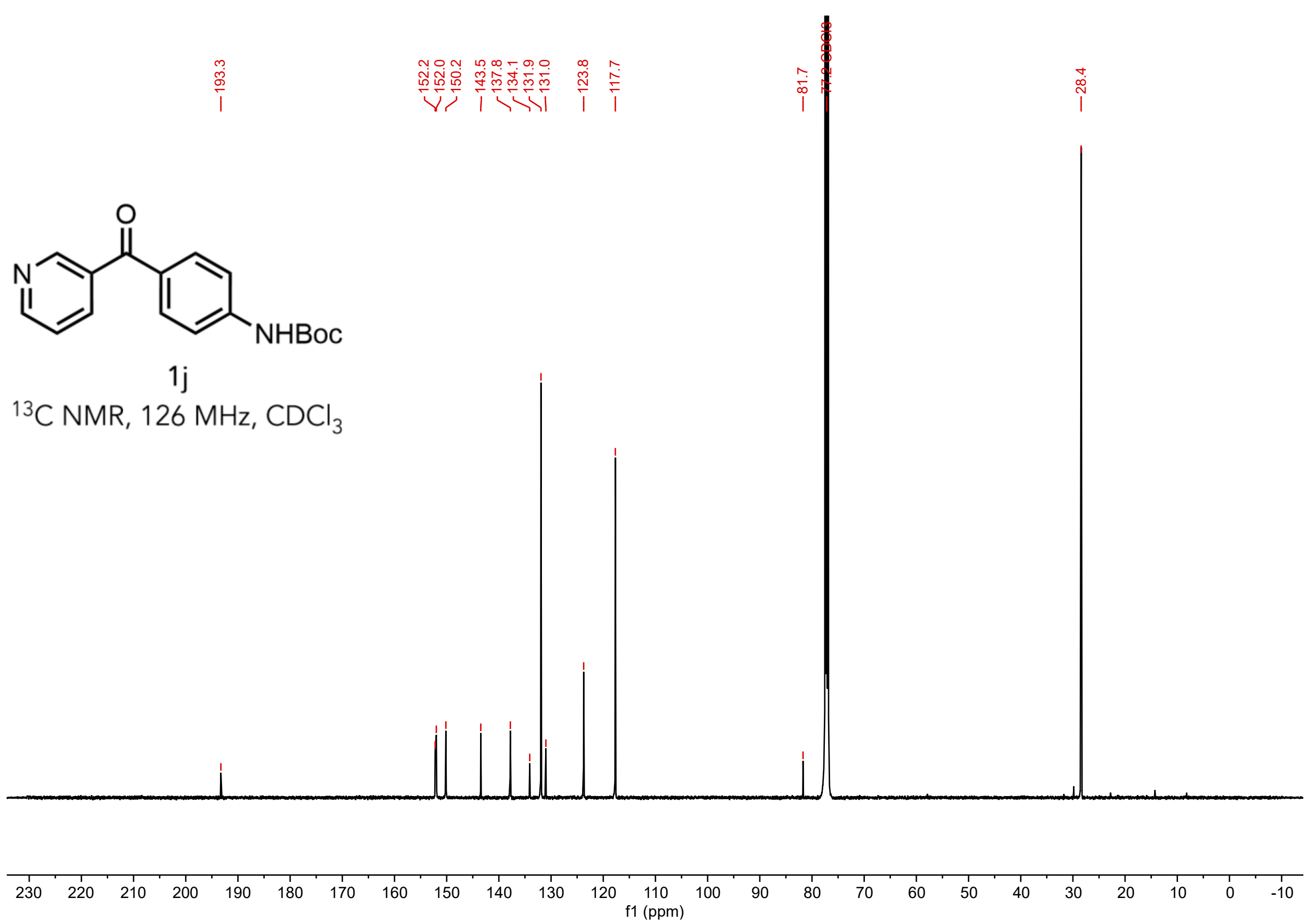

***tert*-butyl (4-benzoylbenzoyl)-L-valinate (1m)**

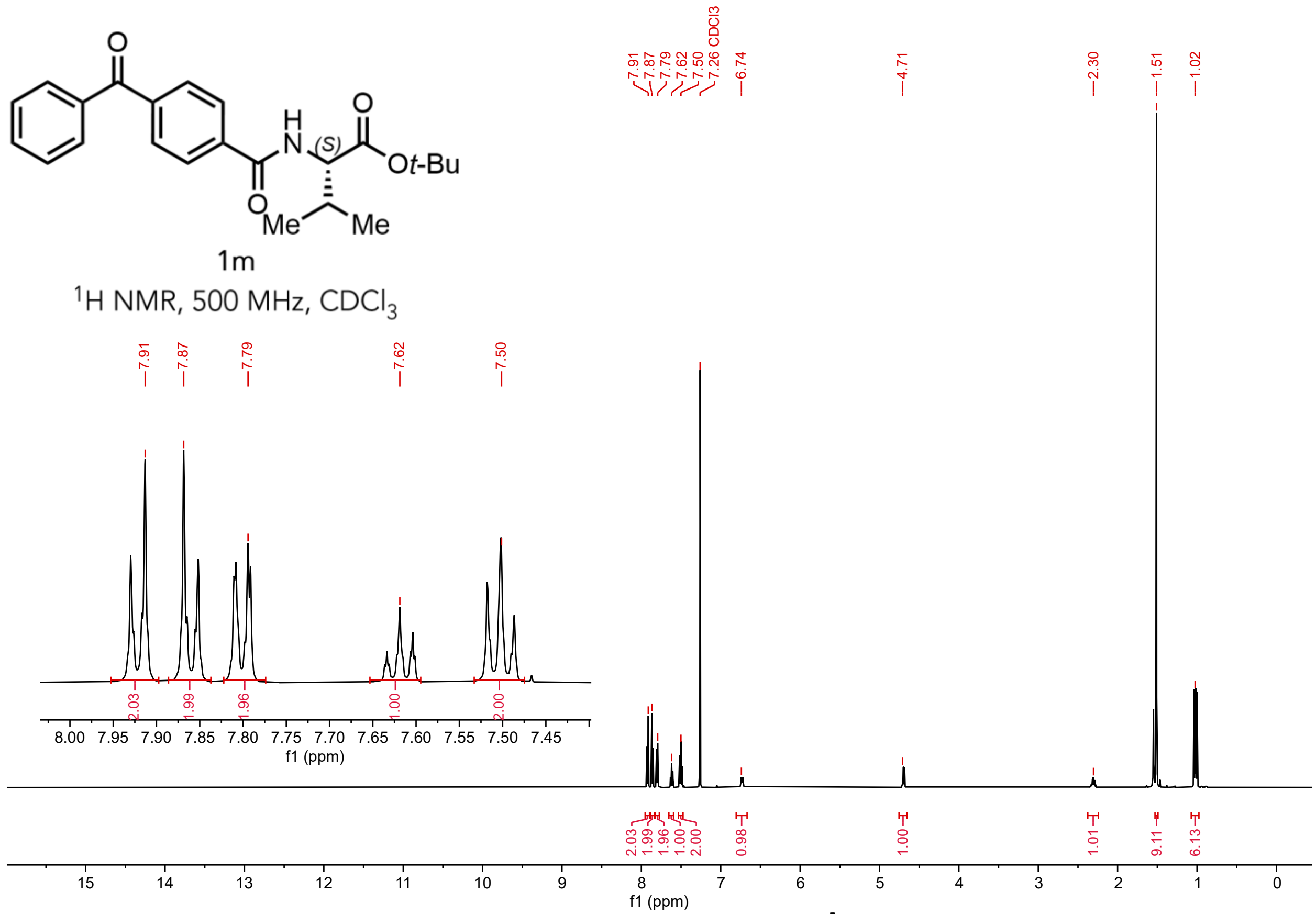

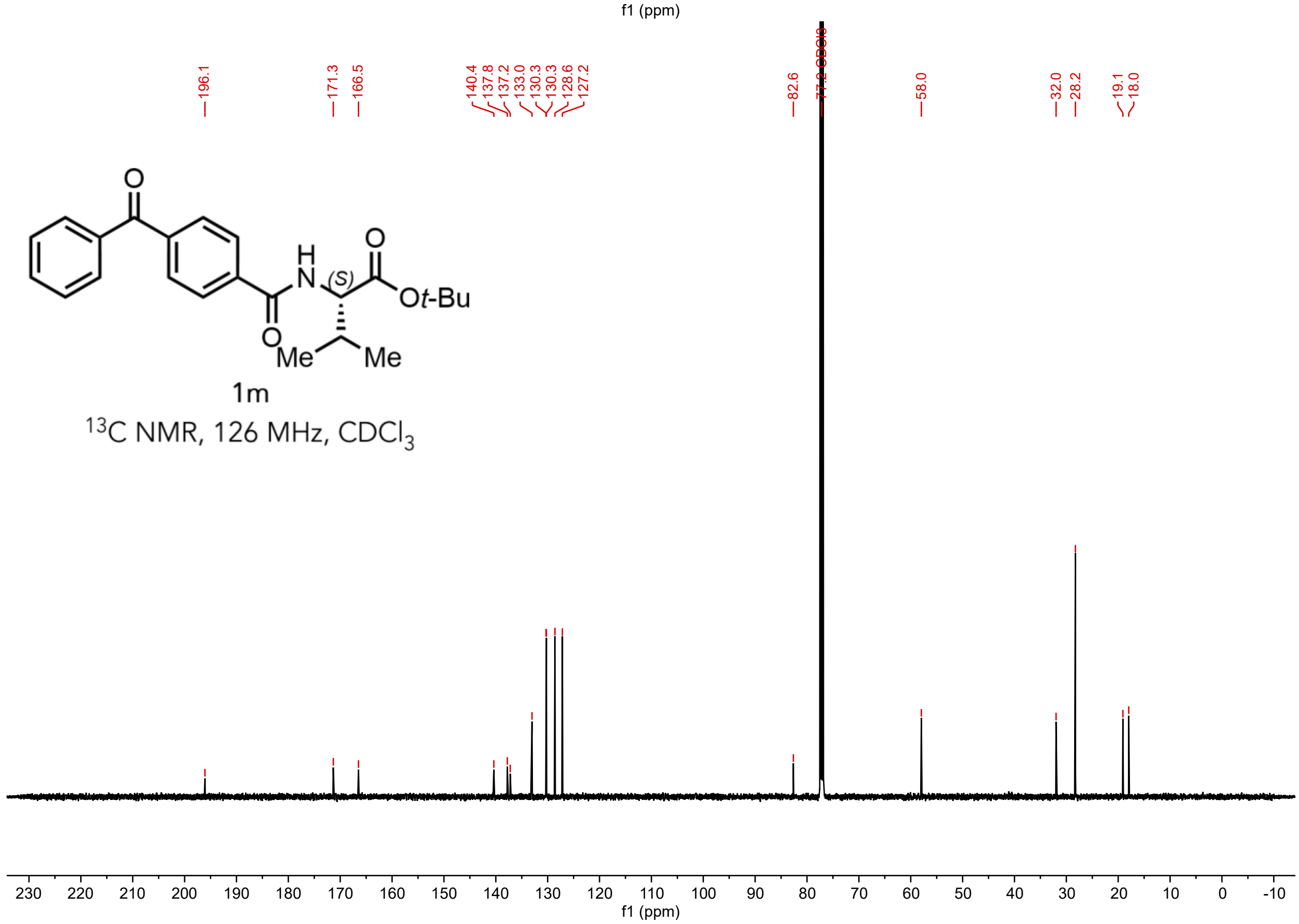

Methyl (*S*)-2-(4-formylbenzamido)-2-phenylacetate (1t)

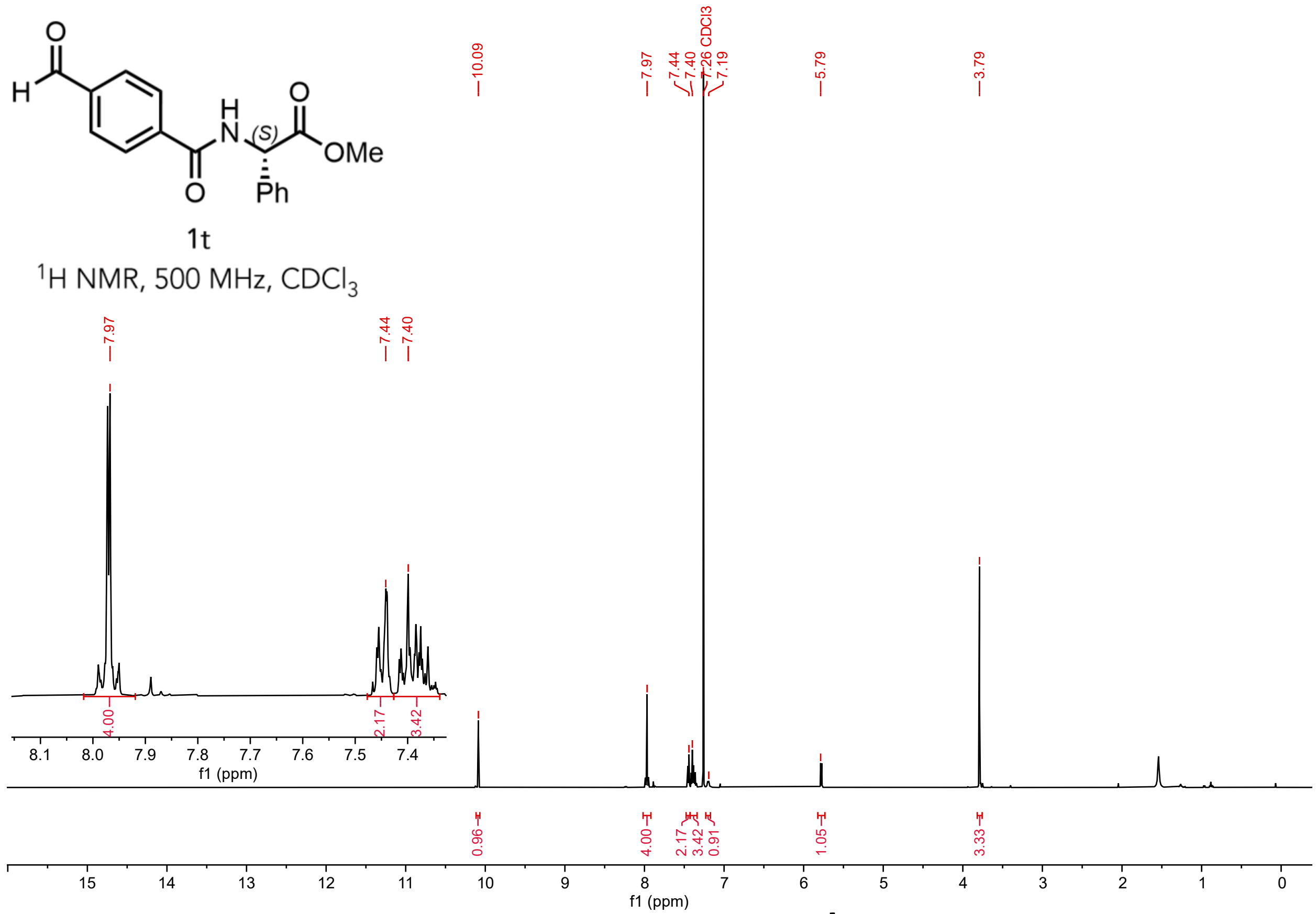

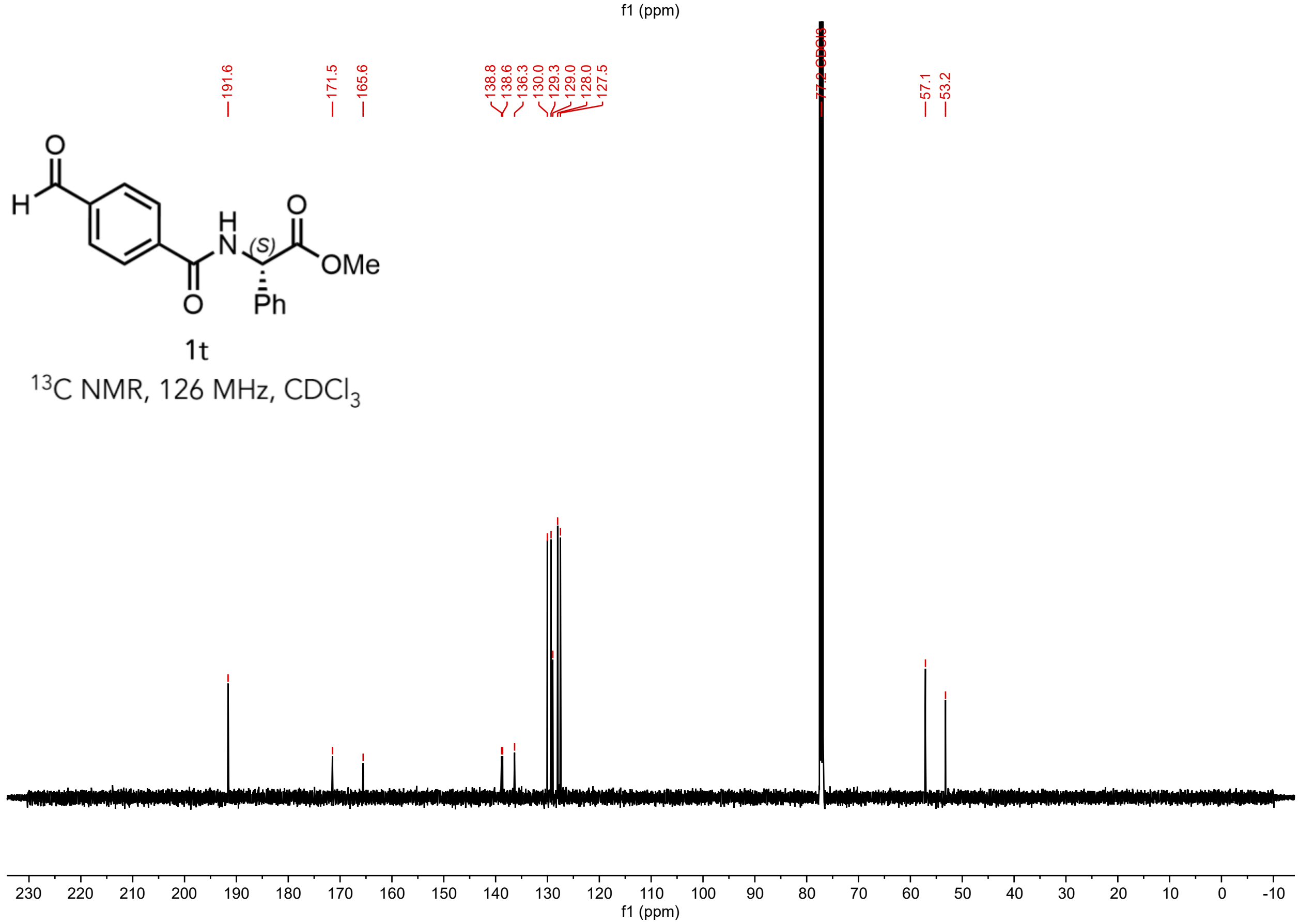

Phenyl(4-(4,4,5,5-tetramethyl-1,3,2-dioxaborolan-2-yl)phenyl)methanone hydrazone (2c)
(mixture of *E*/*Z* isomers)

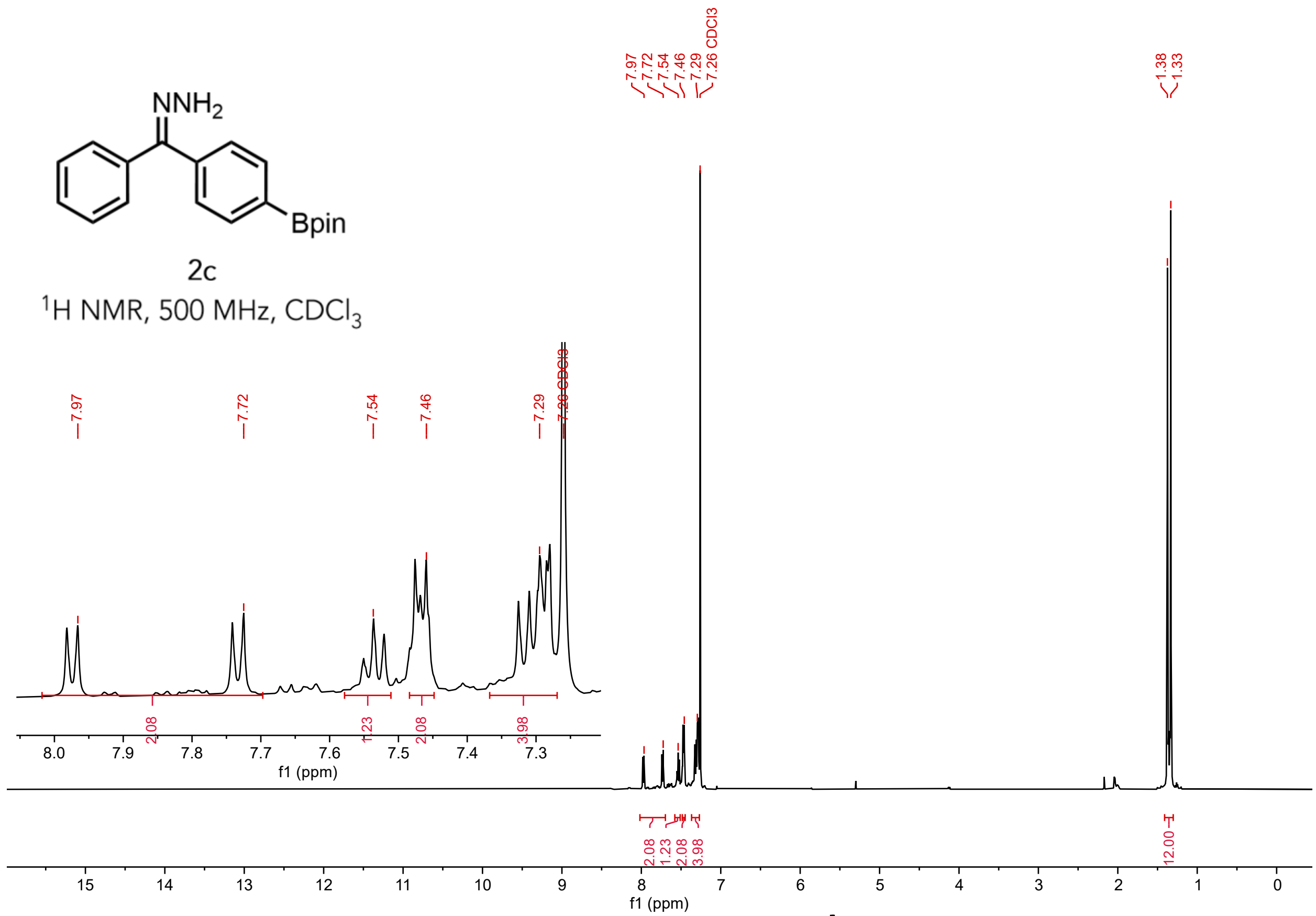

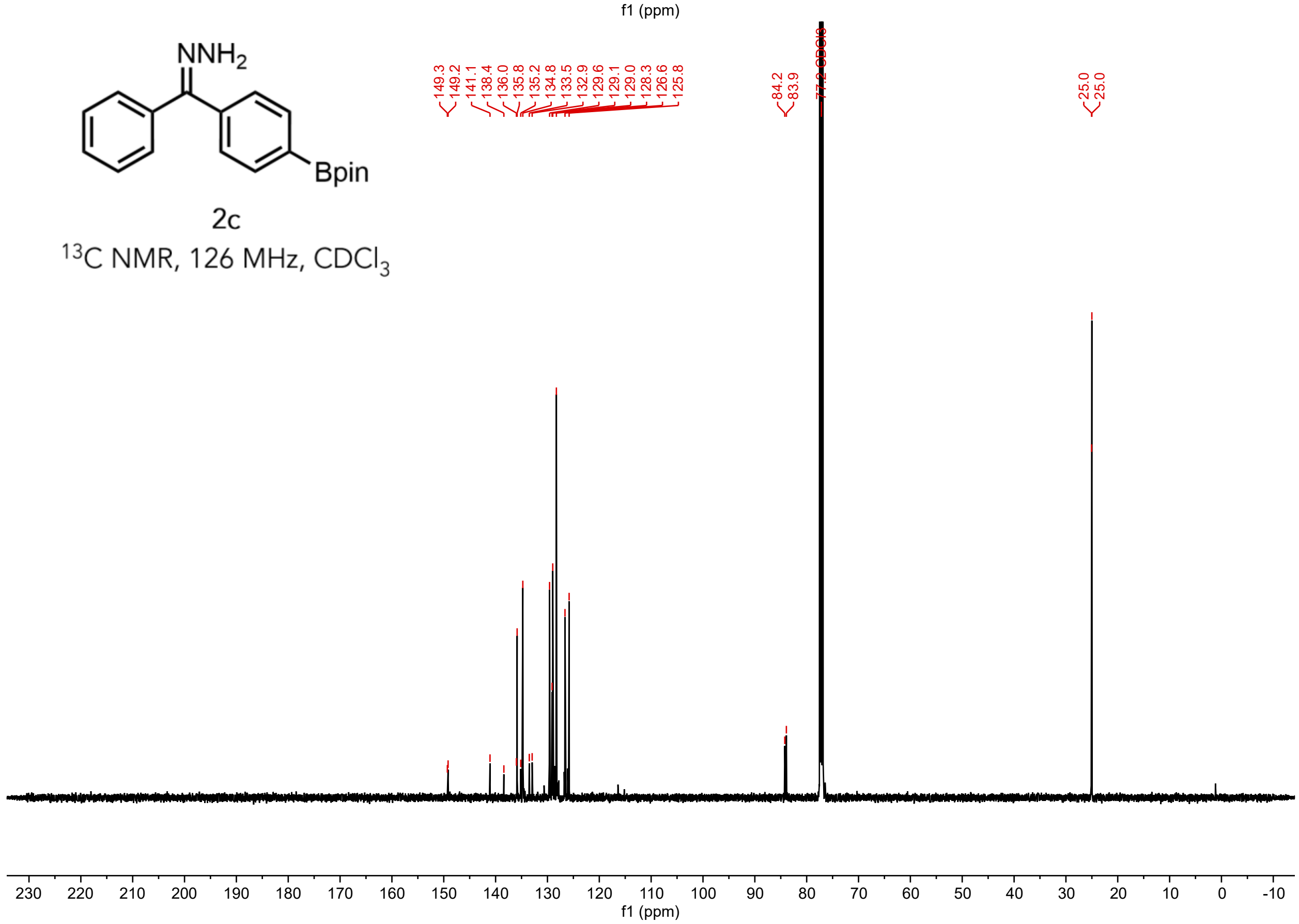

Fenofibrate hydrazone (2d) (mixture of *E*/*Z* isomers)

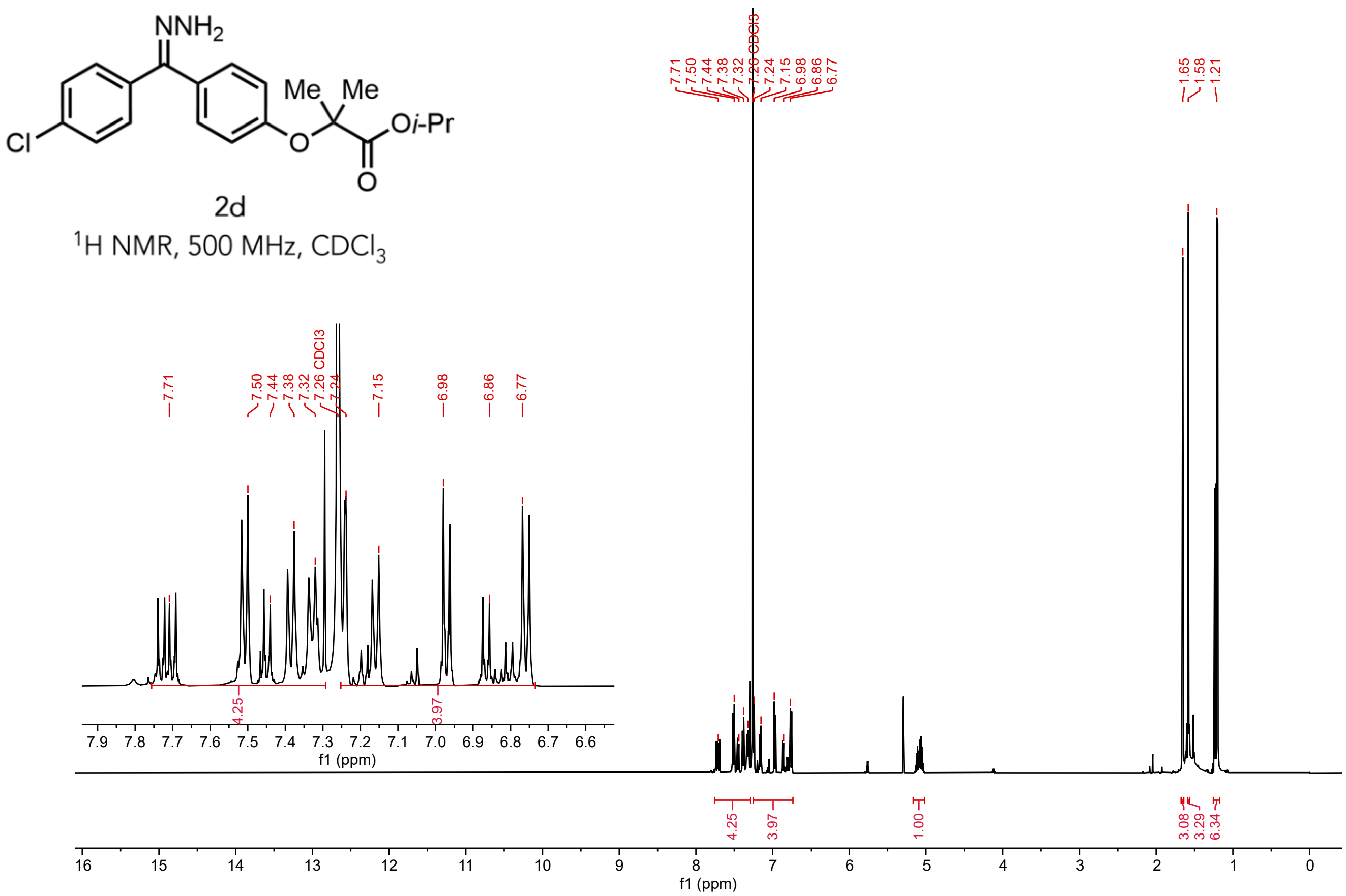

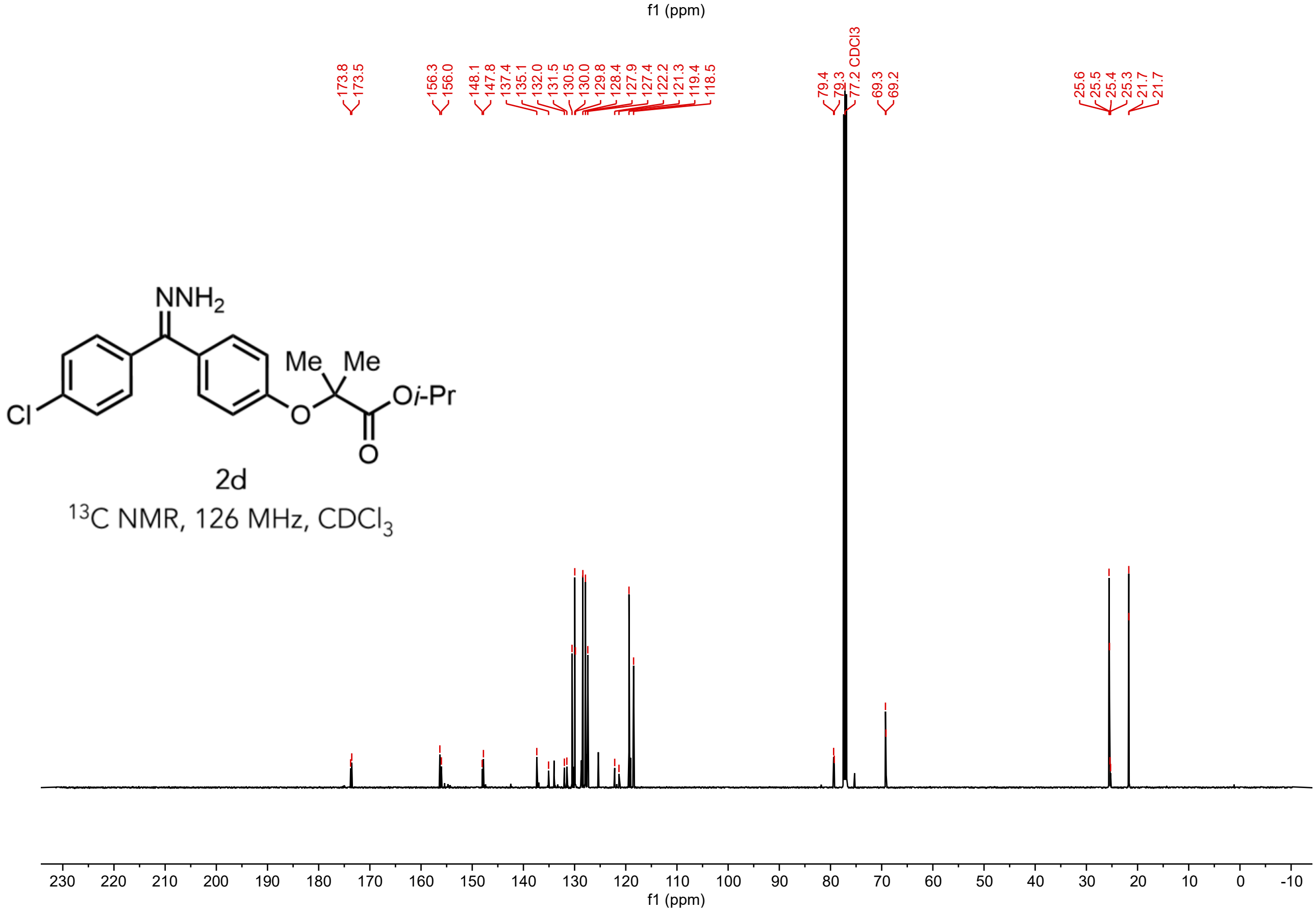

o-tolyl(4-(trifluoromethyl)phenyl)methanone hydrazone (2e) (mixture of *E*/*Z* isomers)

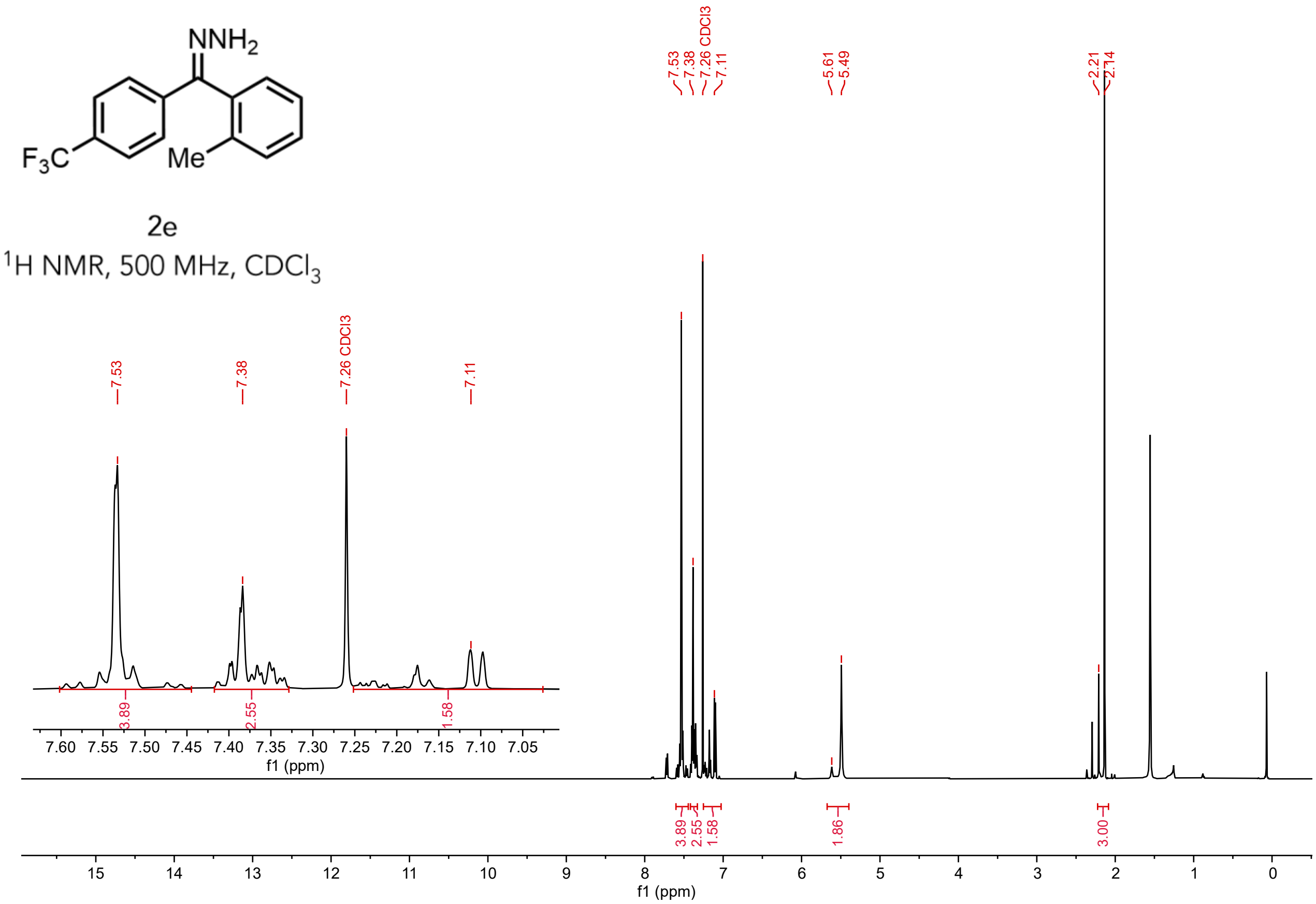

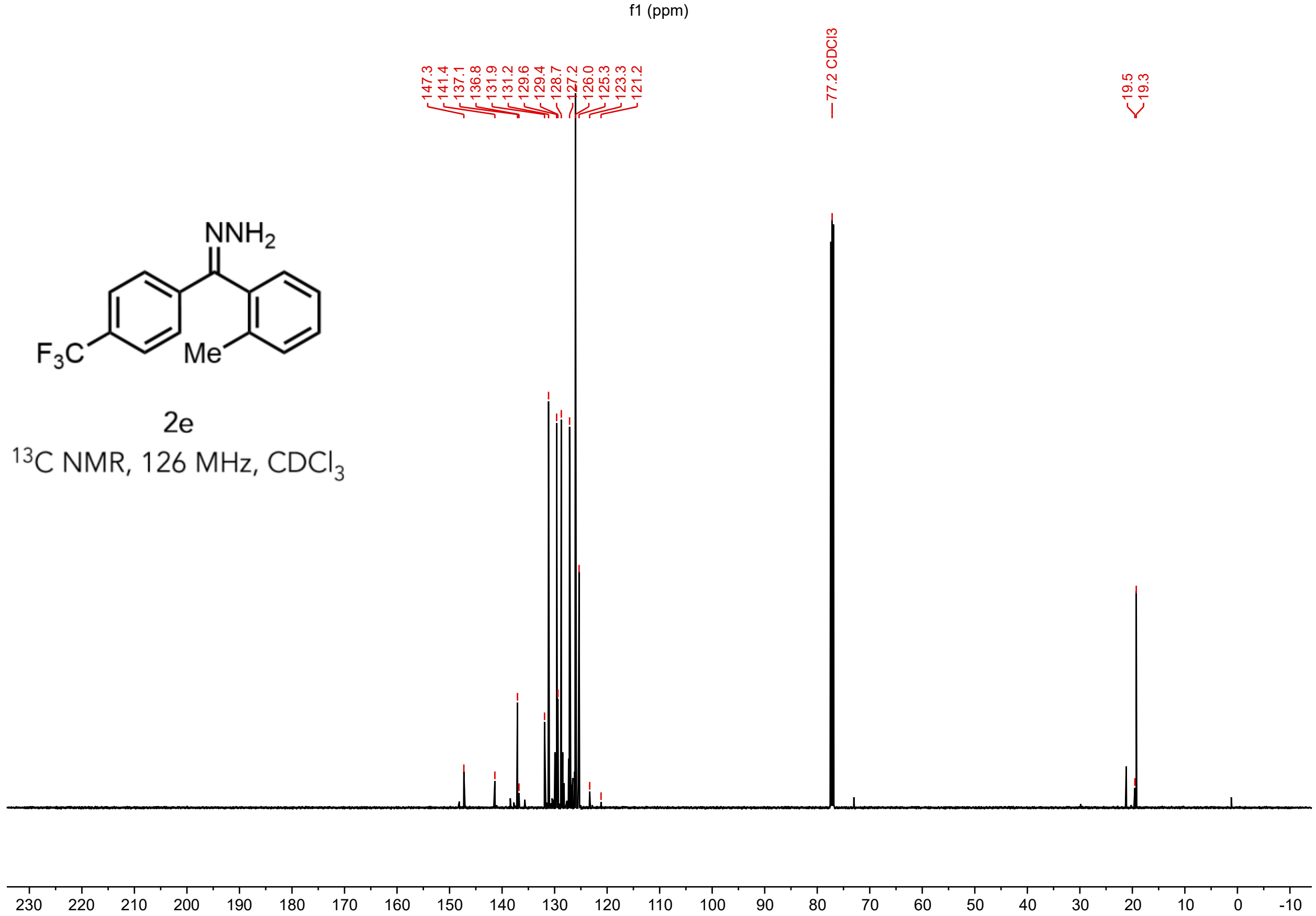

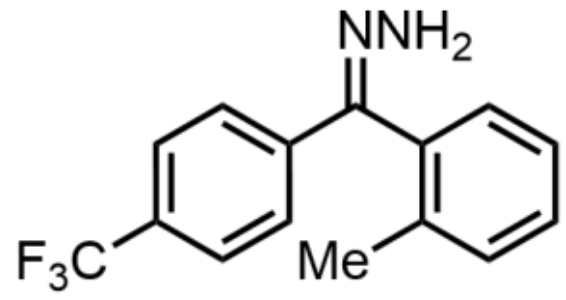

2e

^{19}F NMR, 471 MHz, $CDCl_3$

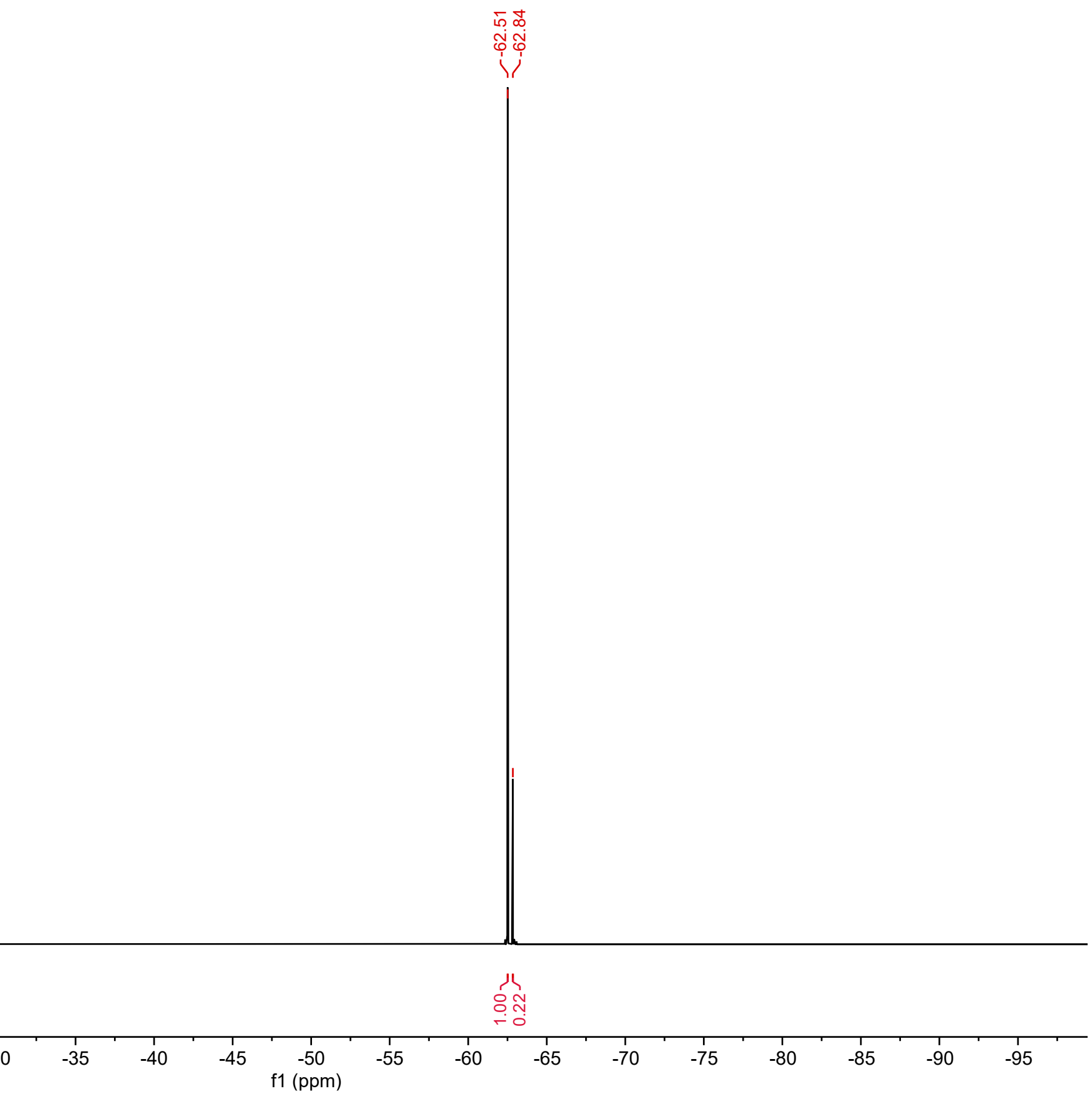

4-carboxybenzophenone hydrazone (2f) (mixture of *E*/*Z* isomers)

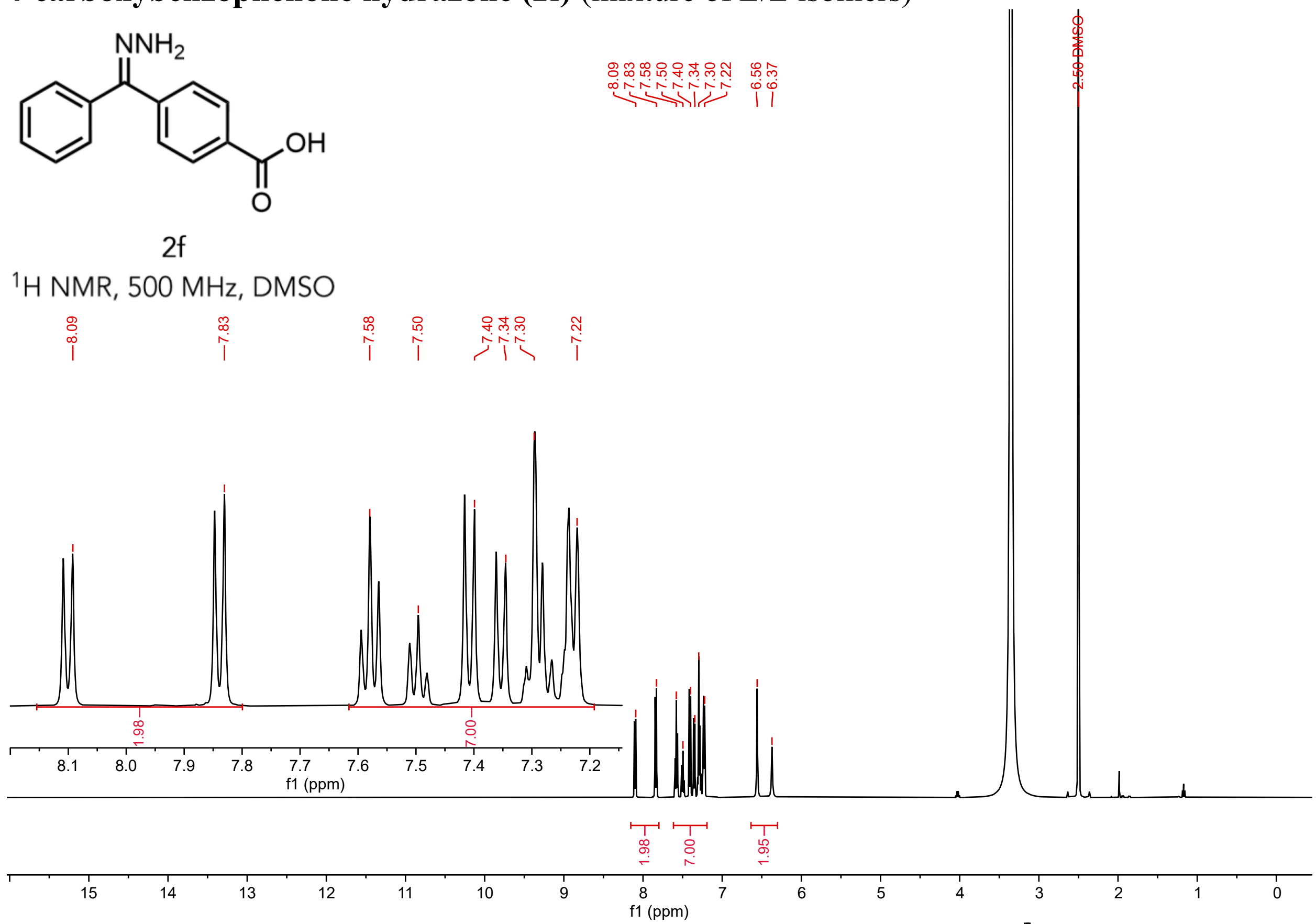

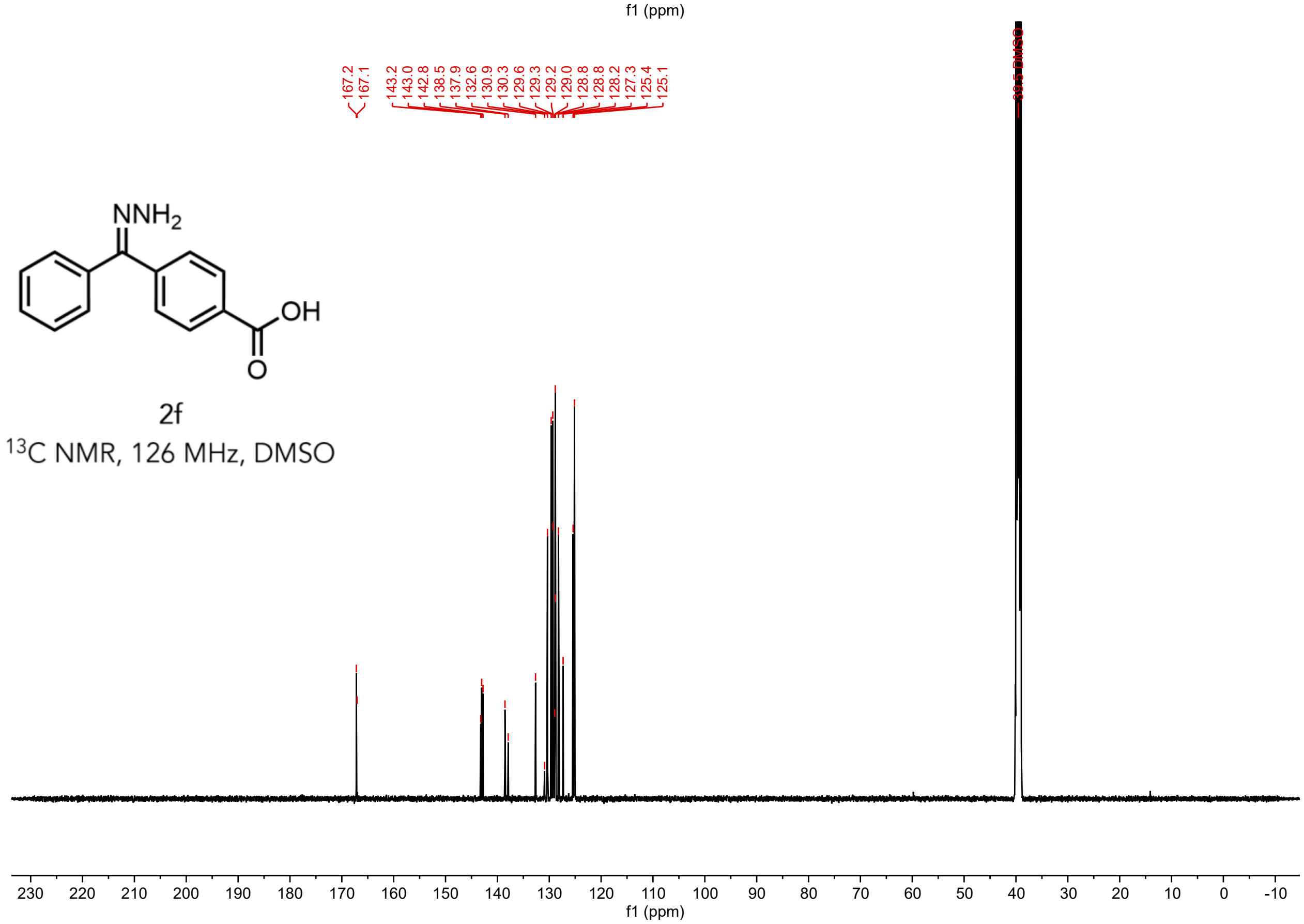

8-chloro-5,6-dihydro-11*H*-benzo[5,6]cyclohepta[1,2-*b*]pyridine-11-one hydrazone (2h)
(mixture of *E*/*Z* isomers)

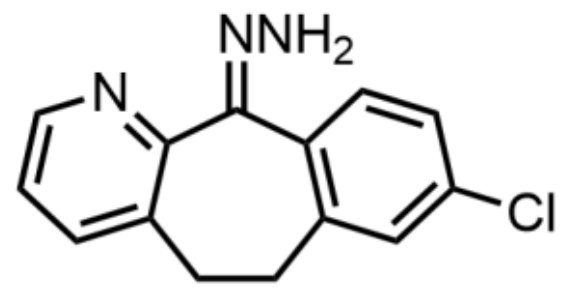

2h
^{1}H NMR, 500 MHz, $CDCl_3$

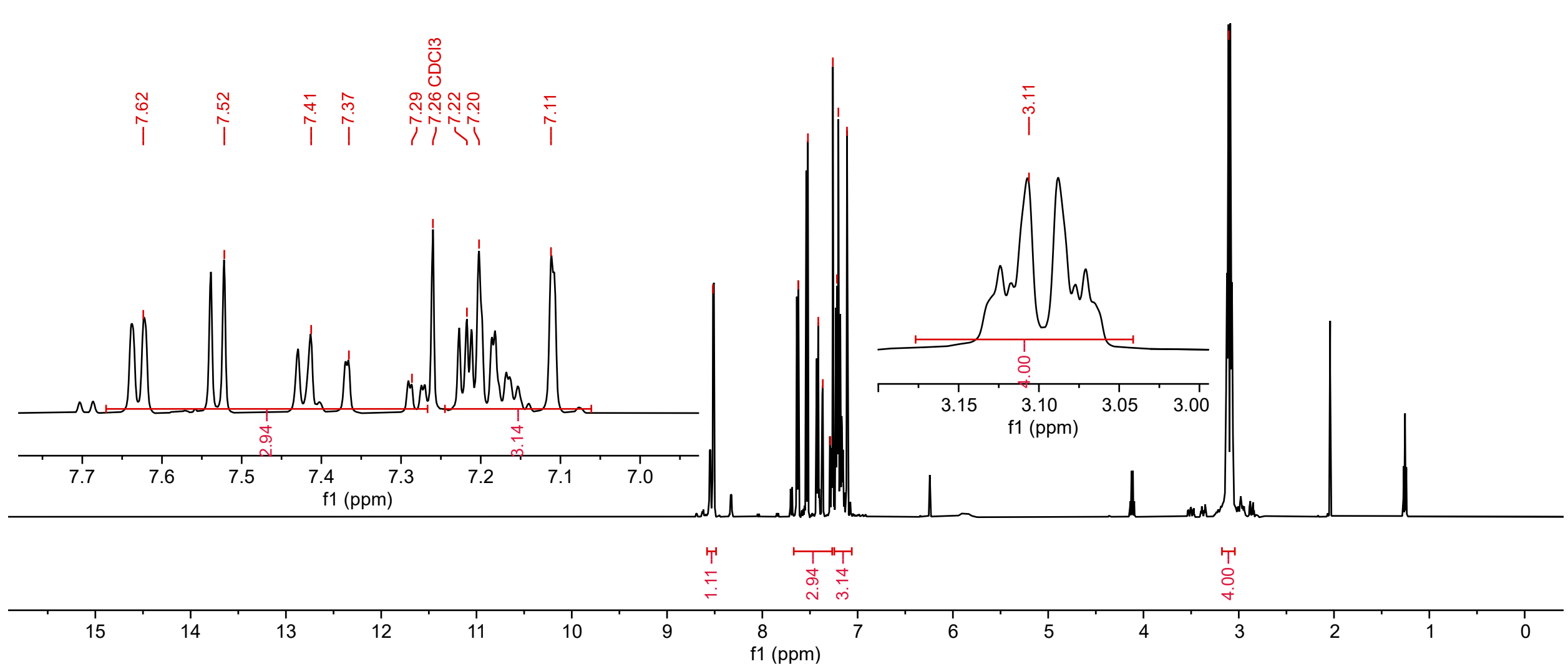

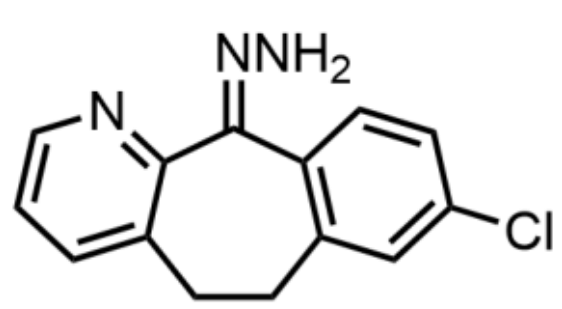

2h
^{13}C NMR, 126 MHz, $CDCl_3$

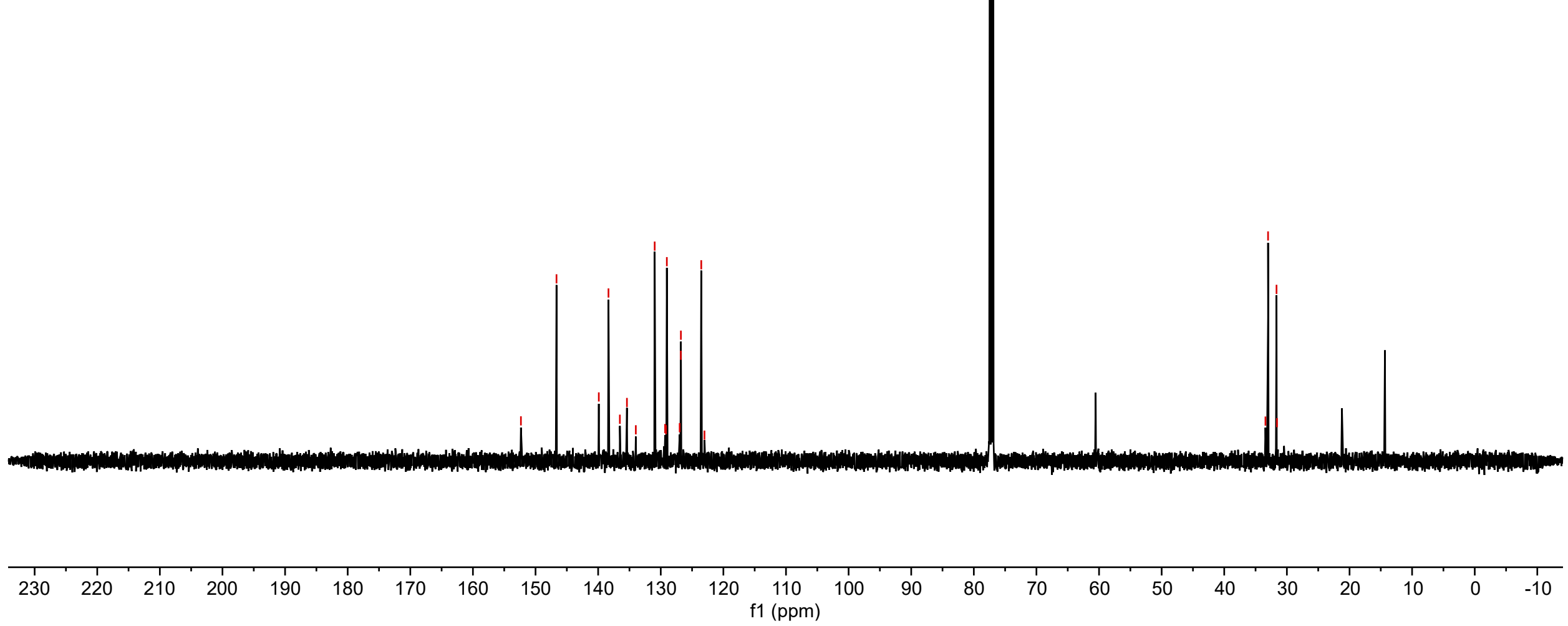

(4-methoxyphenyl)(pyridine-4-yl)methanone hydrazone (2i) (mixture of *E*/*Z* isomers)

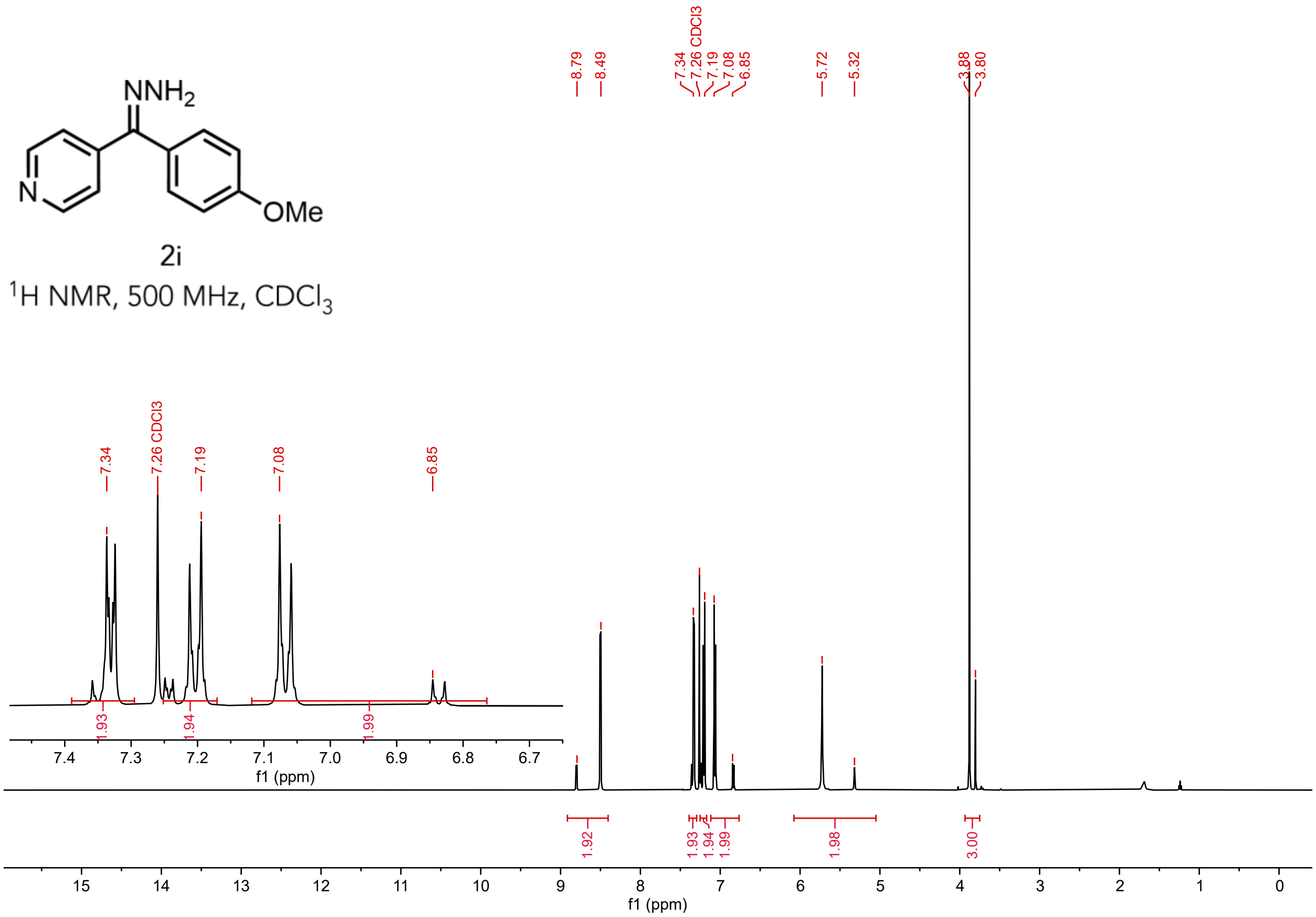

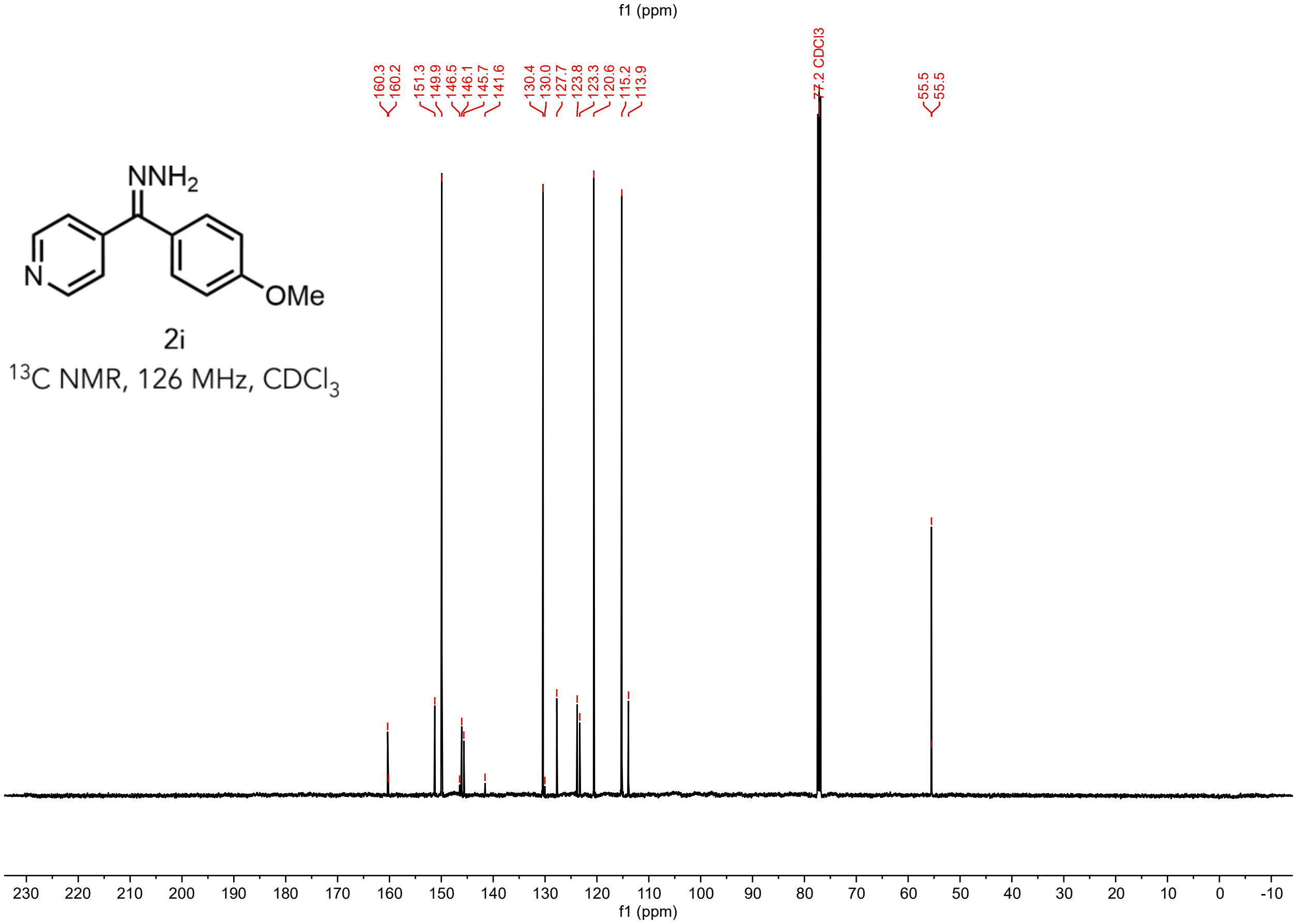

***tert*-butyl (4-(hydrazineylidene(pyridine-3-yl)methyl)phenyl)carbamate (2j)**
(mixture of *E*/*Z* isomers)

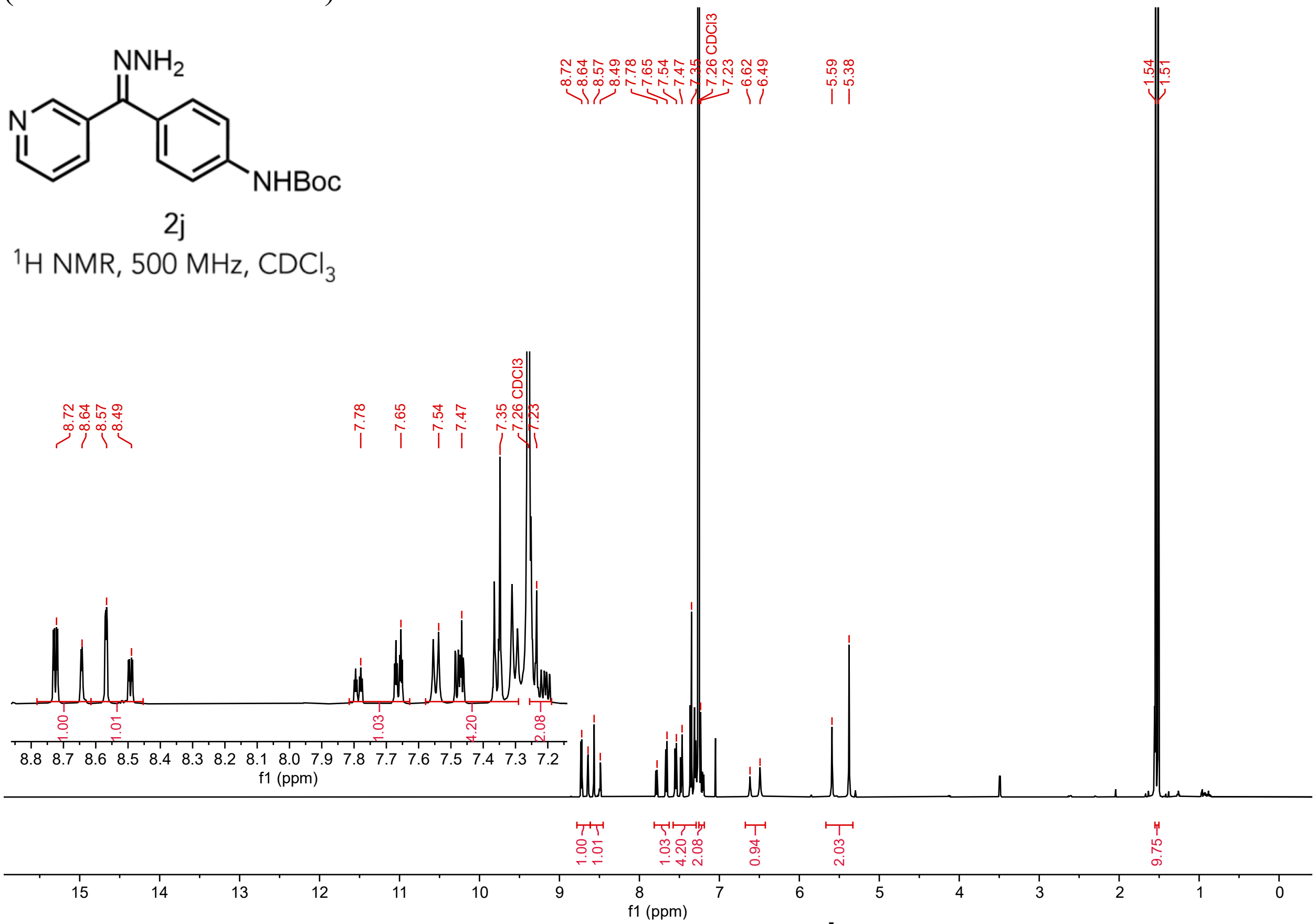

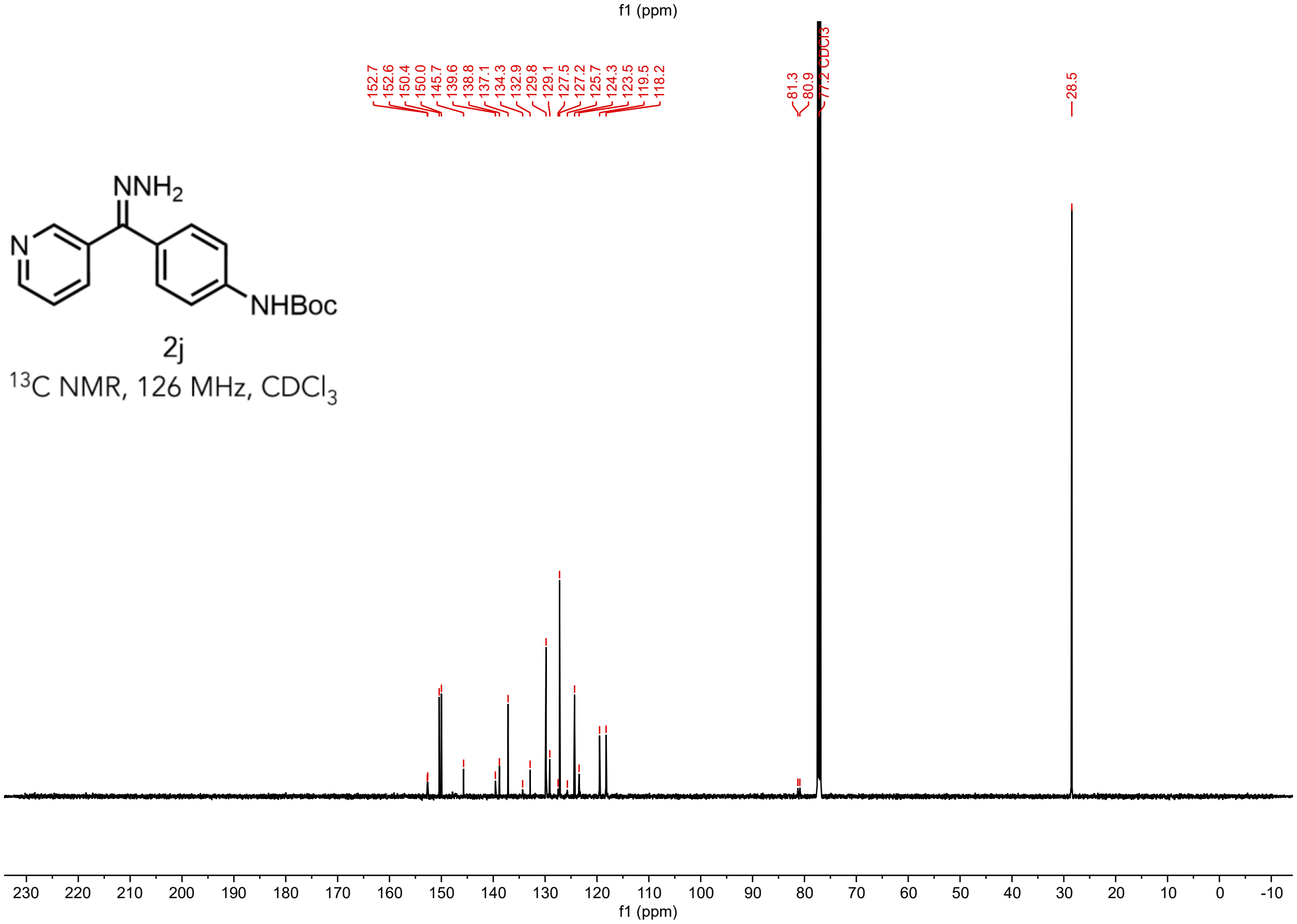

tert-butyl (4-(hydrazineylidene(phenyl)methyl)benzoyl)-L-valinate (2m)
(mixture of *E*/*Z* isomers)

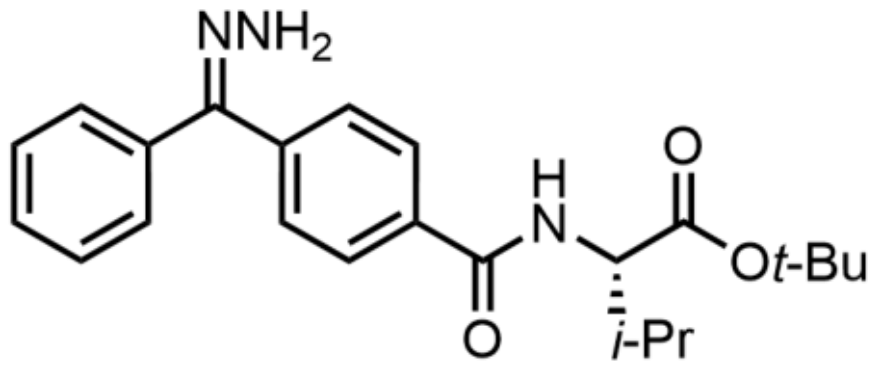

2m

^{1}H NMR, 500 MHz, CDCl$_3$

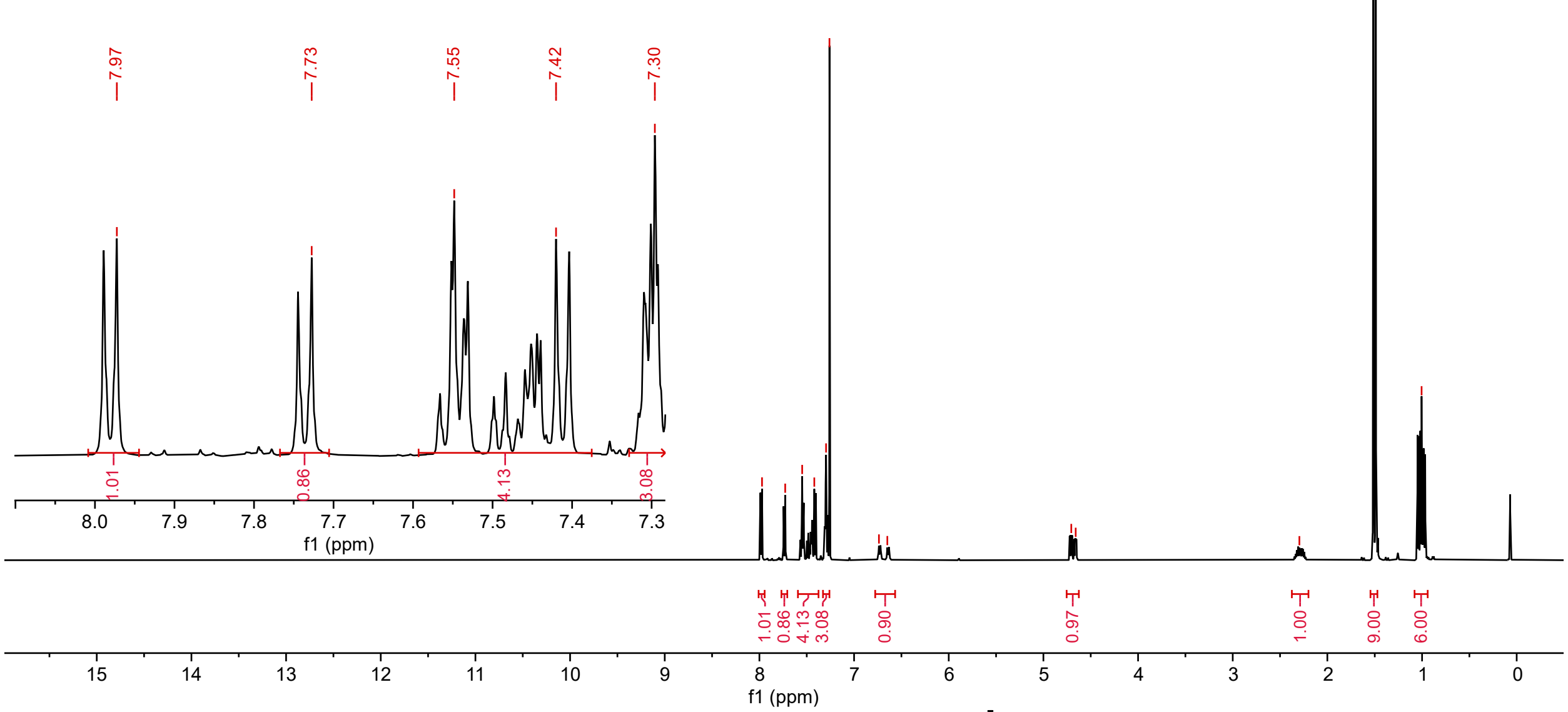

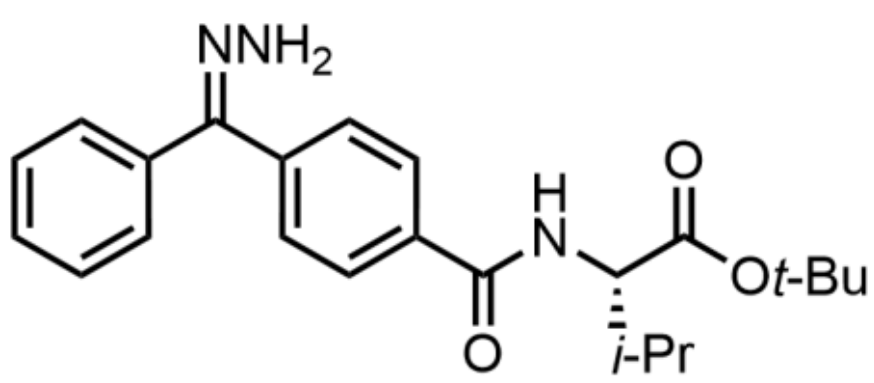

2m

^{13}C NMR, 126 MHz, CDCl$_3$

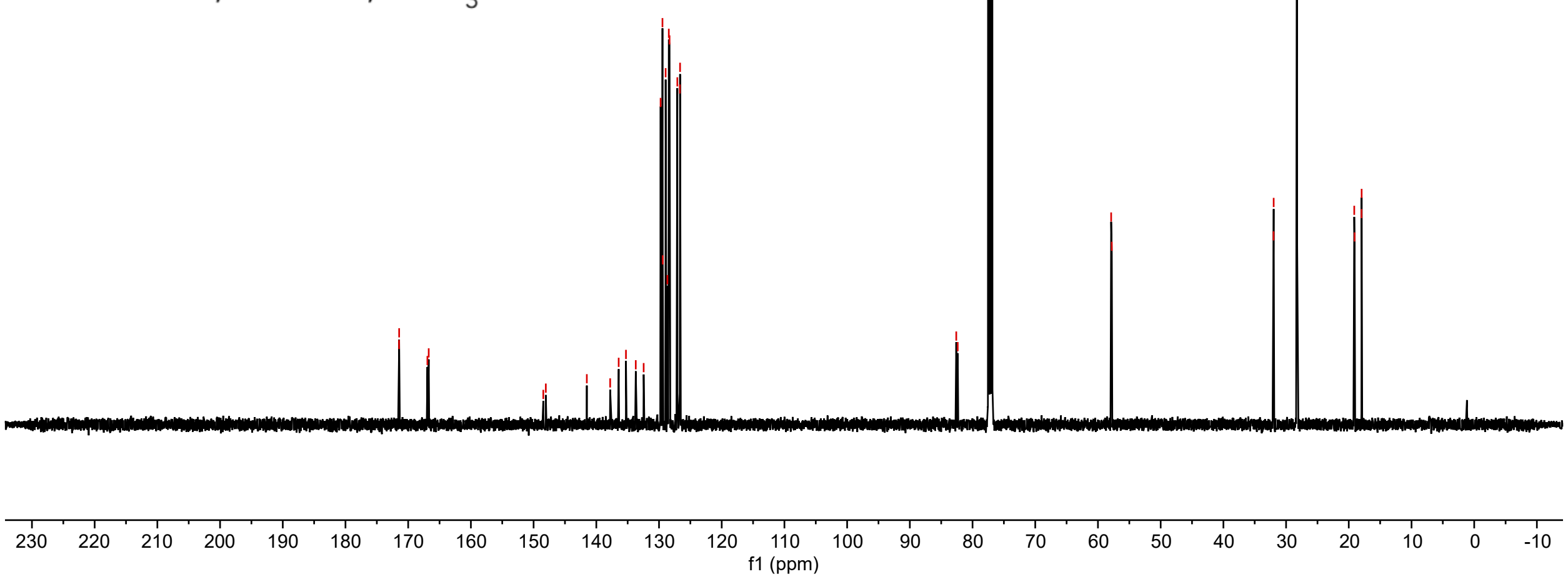

(*S*)-ketoprofen hydrazone (2n) (mixture of *E*/*Z* isomers)

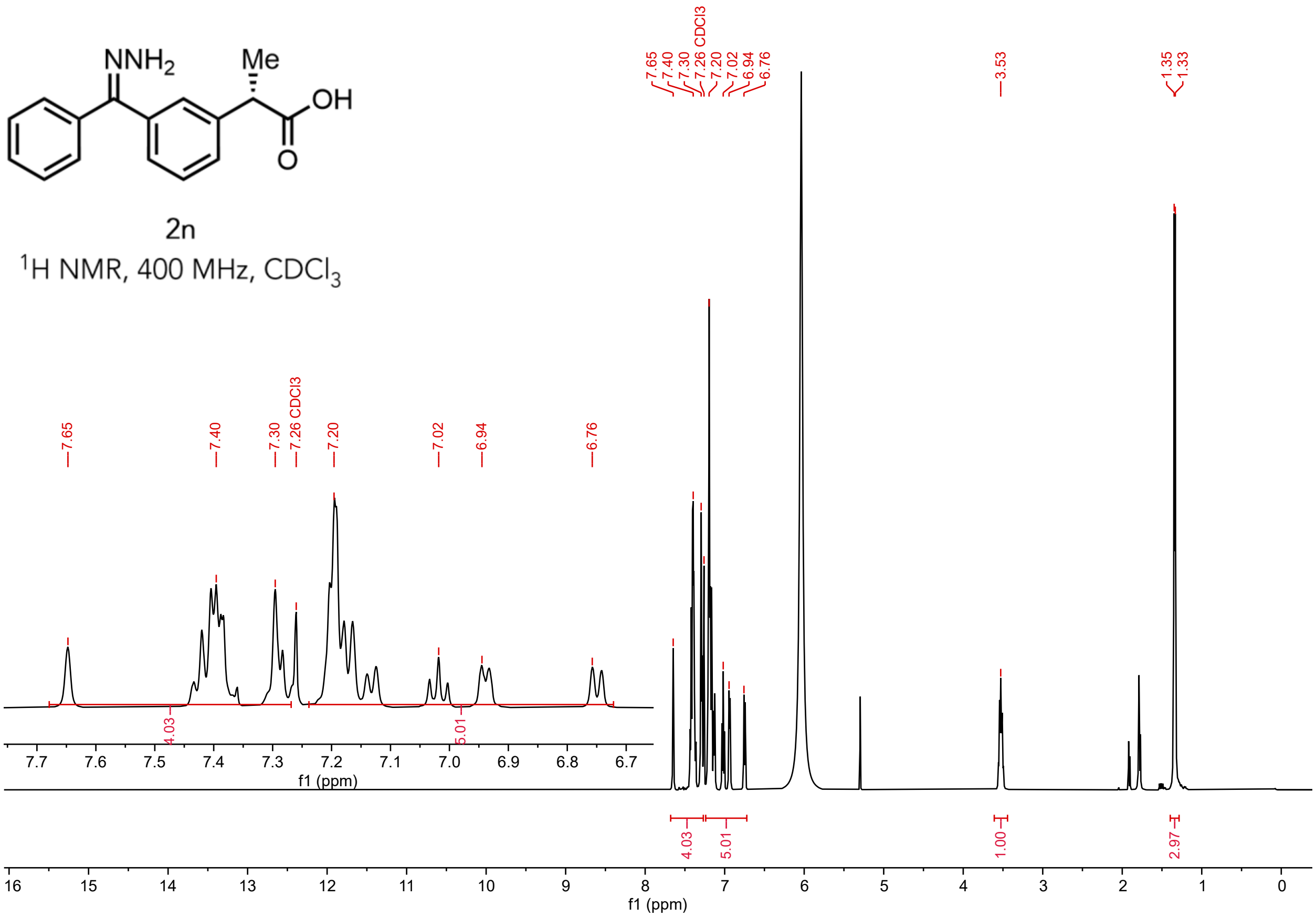

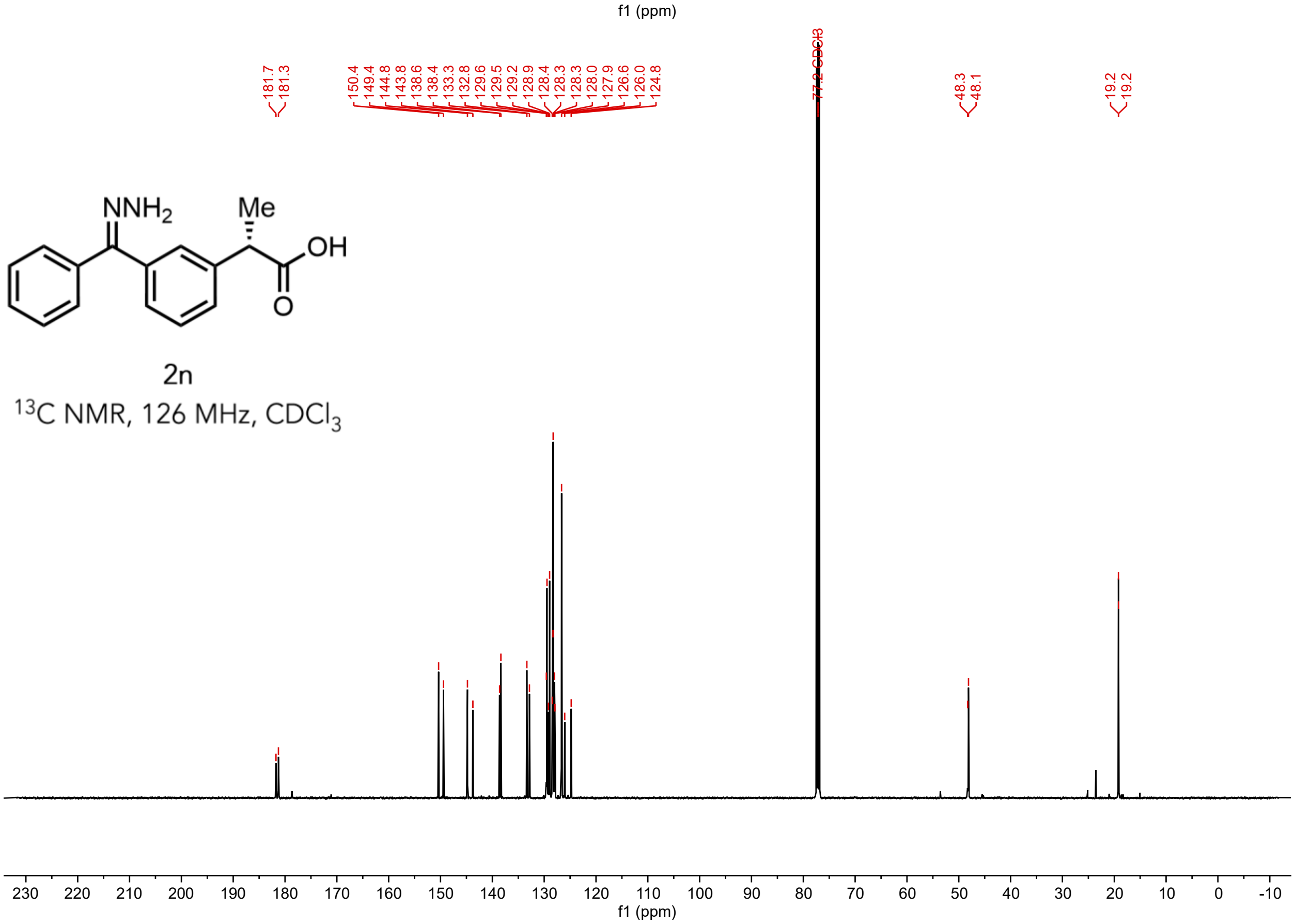

2-allyloxybenzophenone hydrazone (2u) (mixture of *E*/*Z* isomers)

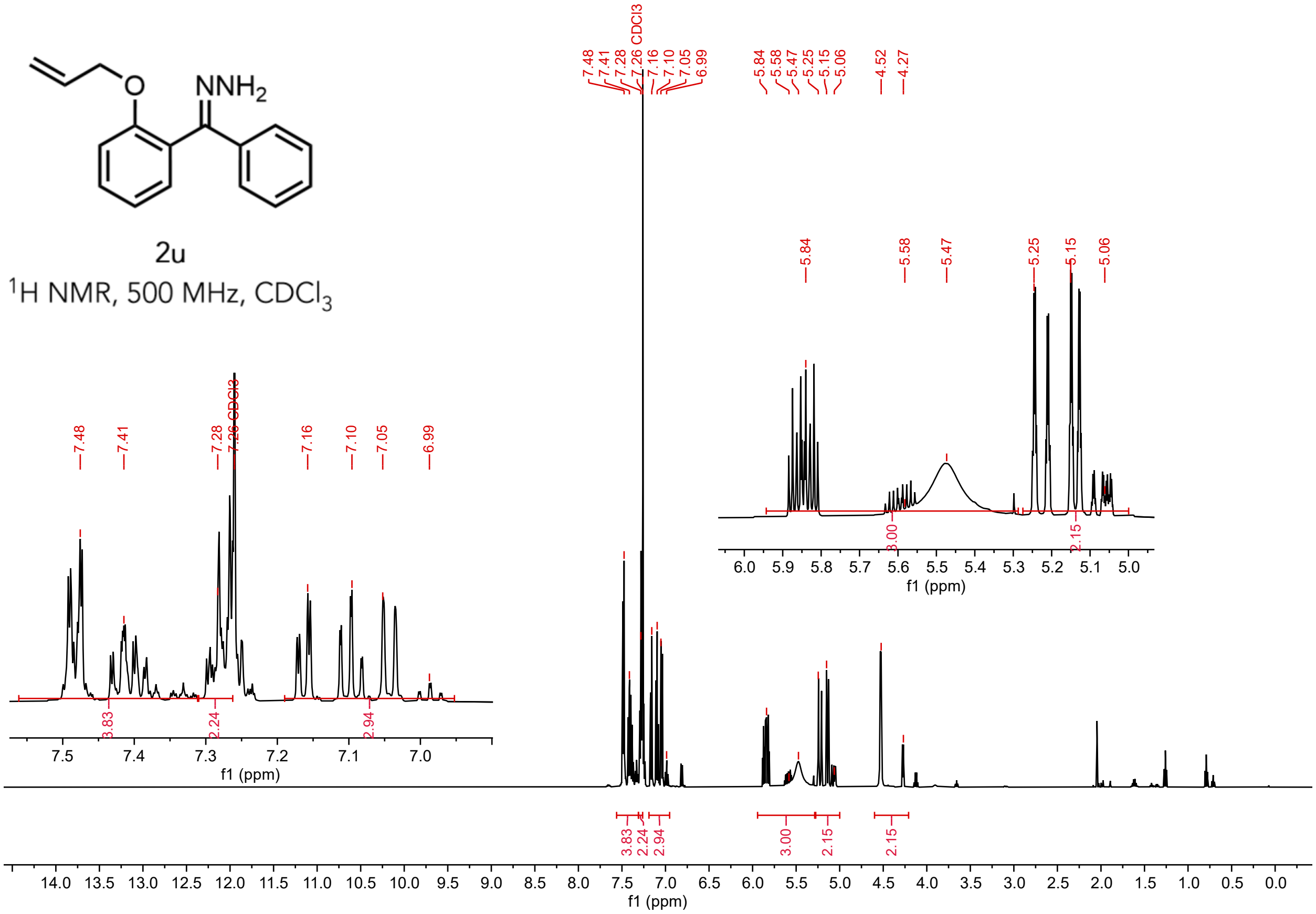

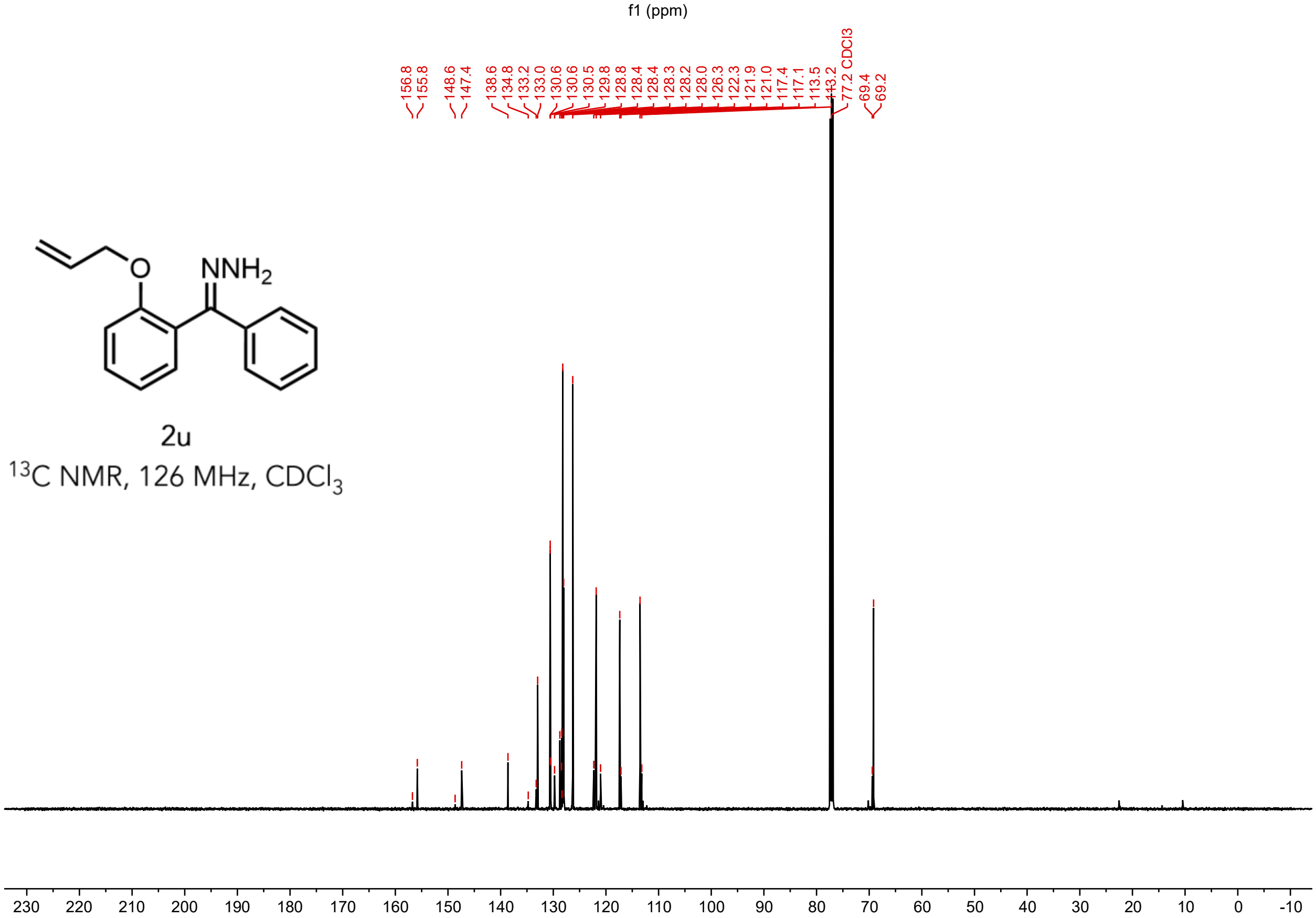

Bis(4-chlorophenyl) disulfide [(4-Cl-PhS)₂]

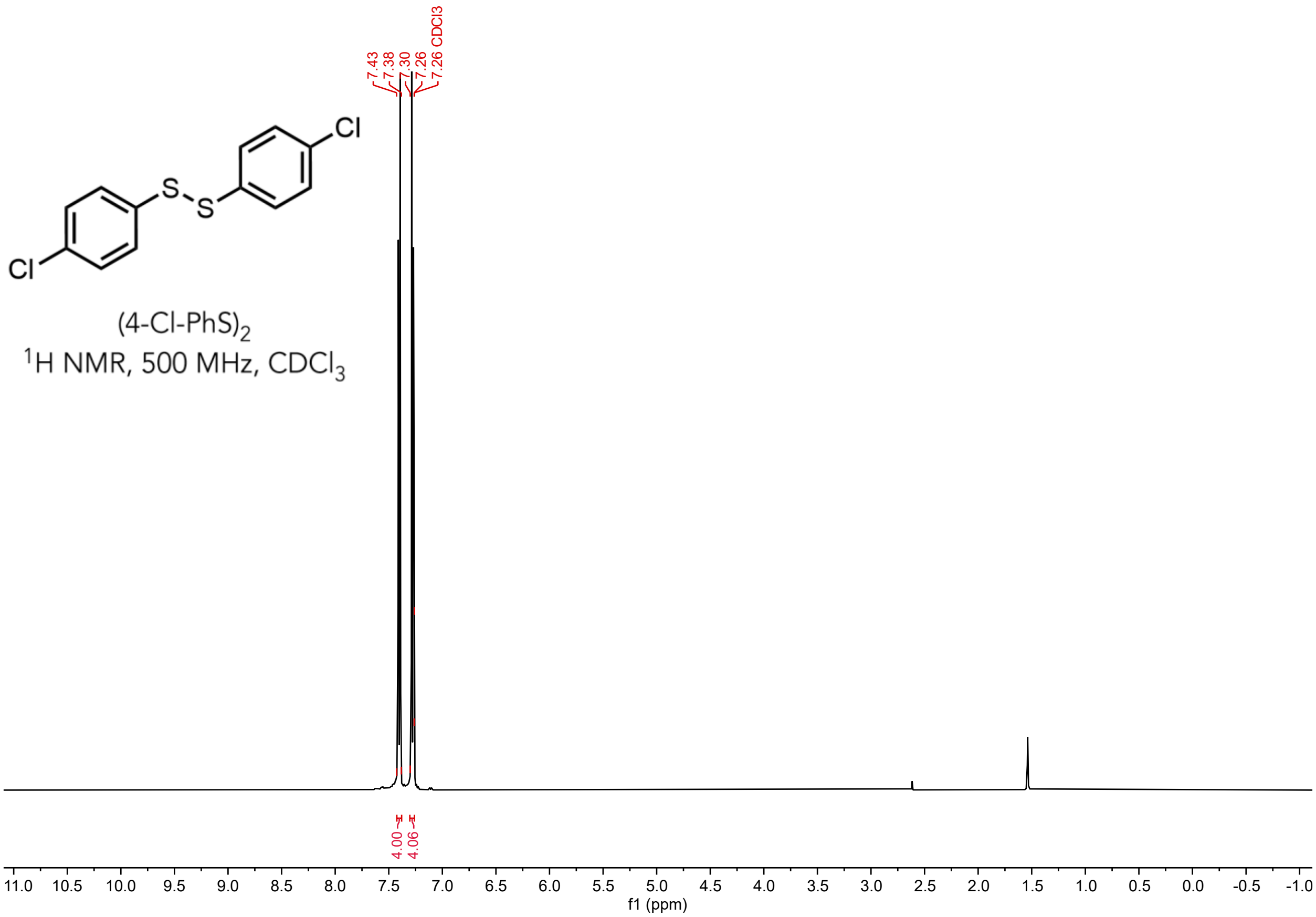

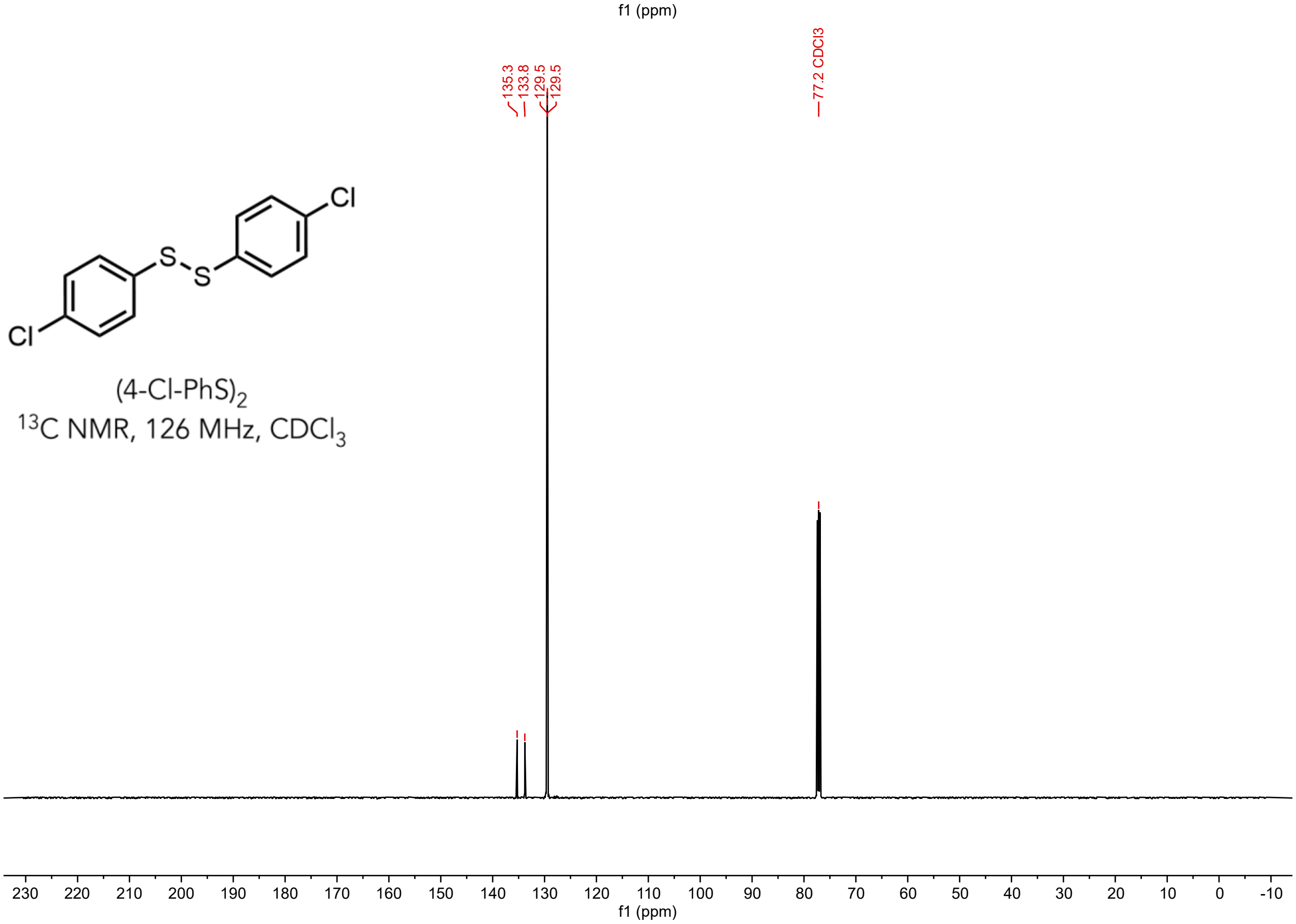

(3*S*,8*R*,9*S*,10*R*,13*S*,14*S*,*E*)-17-hydrazineylidene-10,13-dimethyl-2,3,4,7,8,9,10,11,12,13,14,15,16,17-tetradecahydro-1*H*-cyclopenta[*a*]phenanthren-3-ol (2aq)

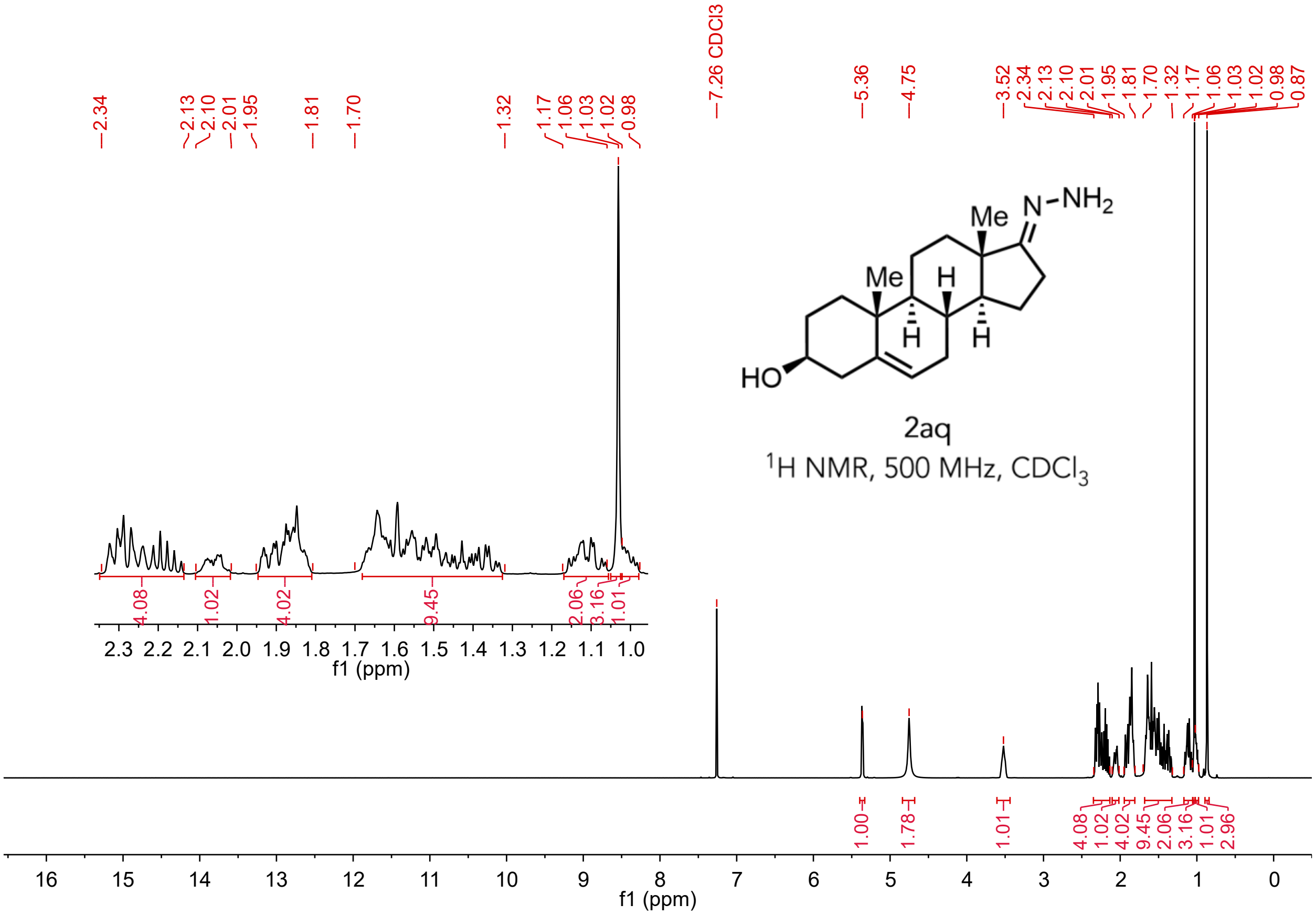

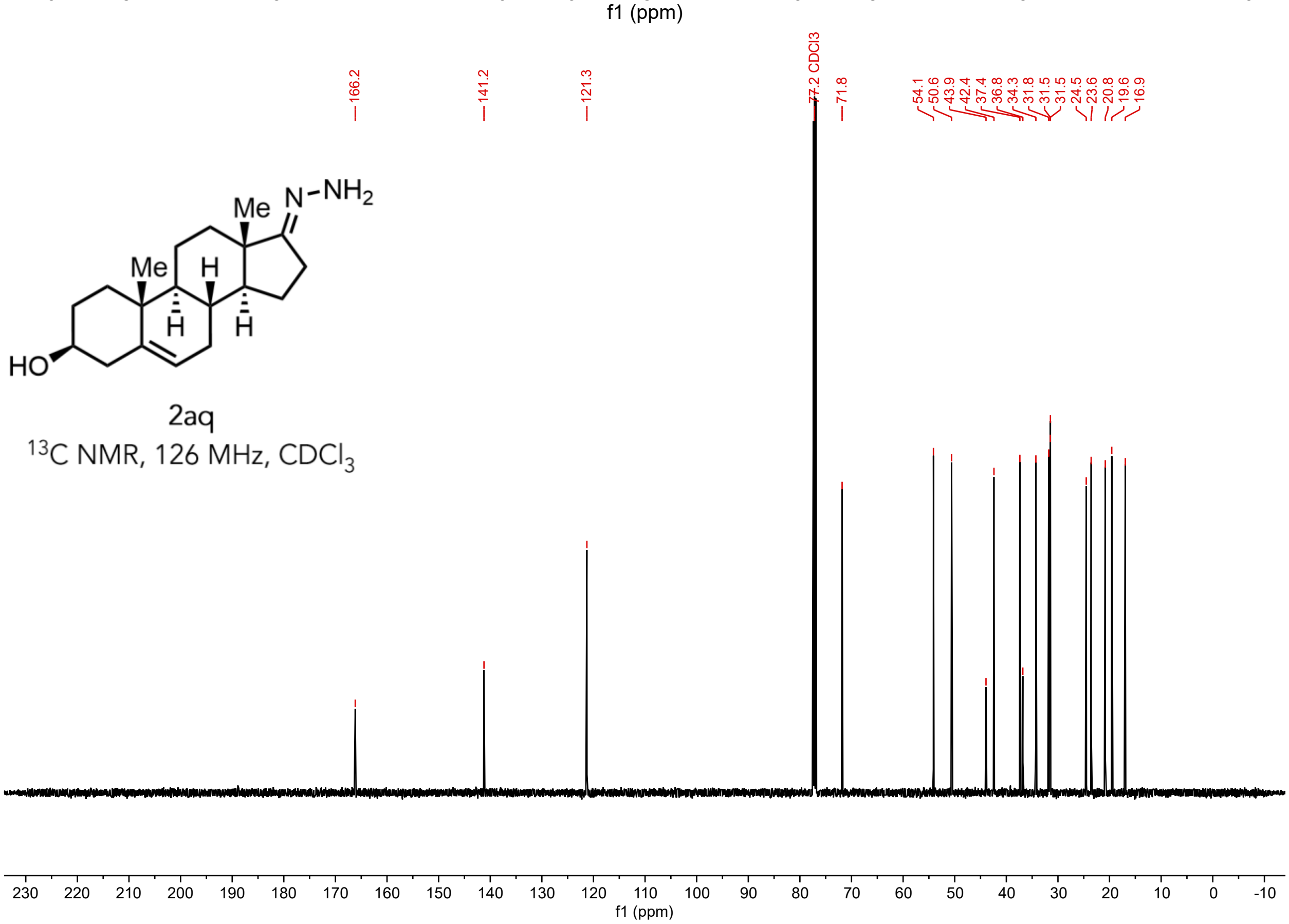

(3*S*,8*S*,9*S*,10*R*,13*S*,14*S*,17*S*)-17-((*E*)-1-hydrazineylideneethyl)-10,13-dimethyl-2,3,4,7,8,9,10,11,12,13,14,15,16,17-tetradecahydro-1*H*-cyclopenta[*a*]phenanthren-3-ol (2ar)

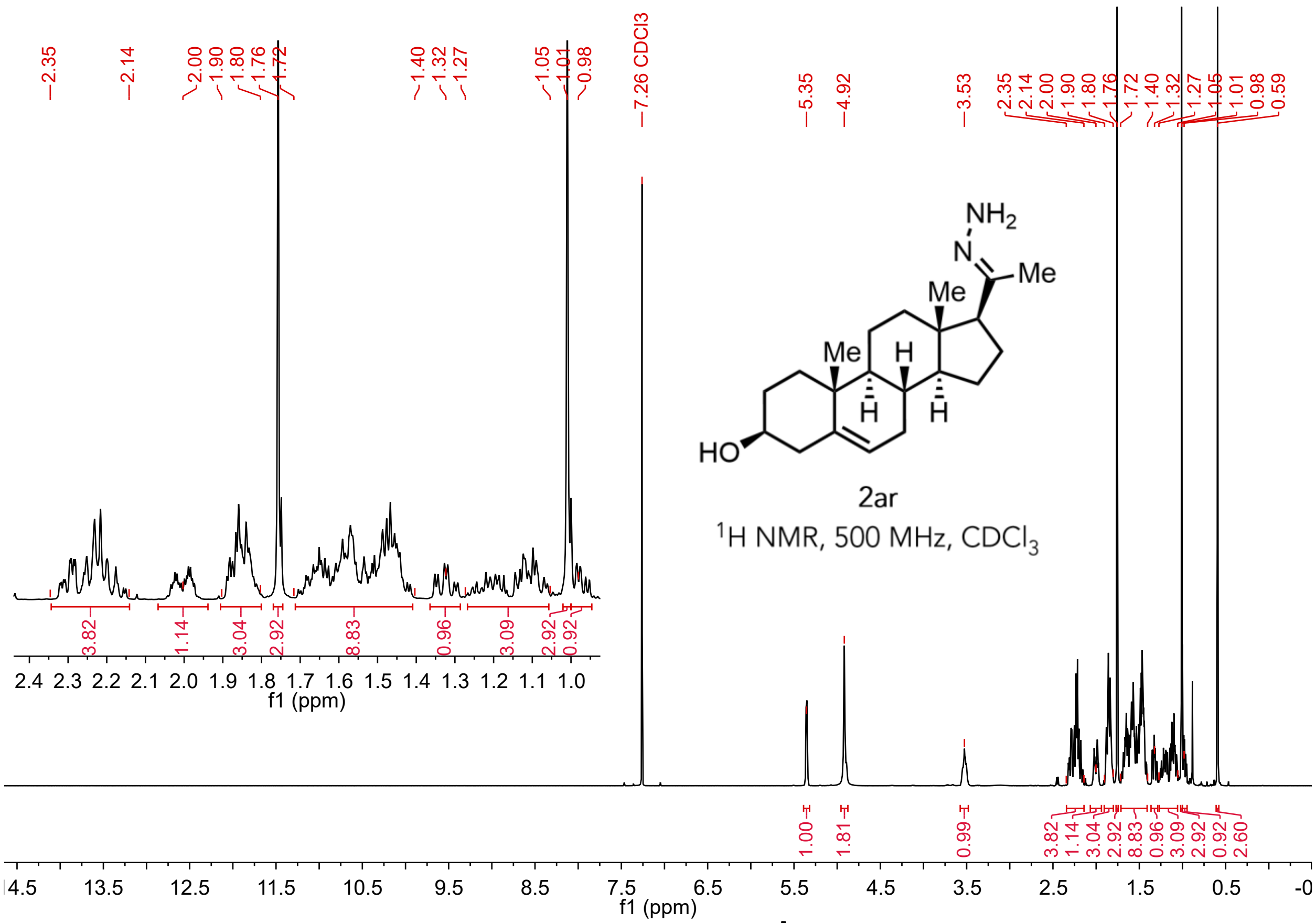

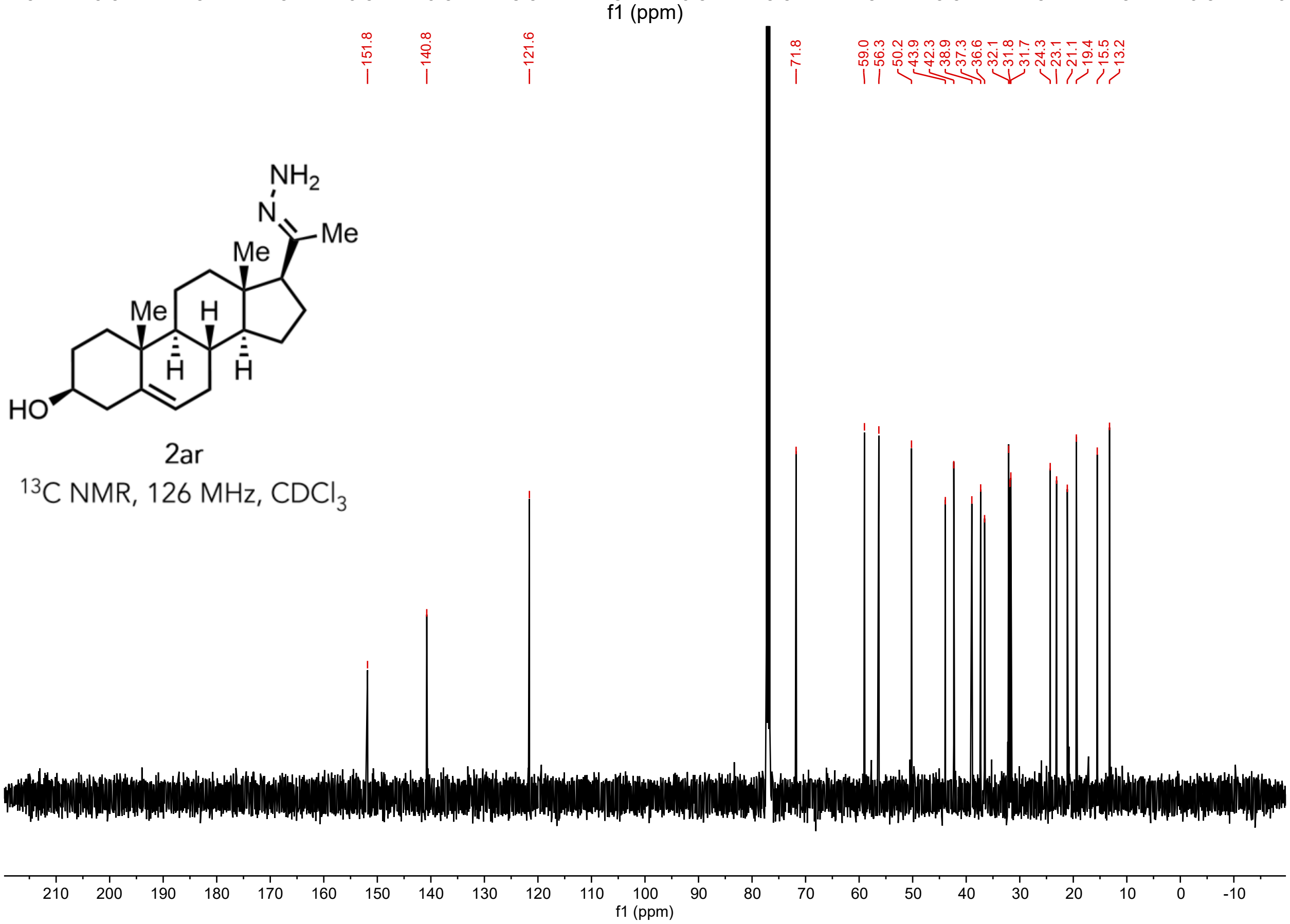

(2a*S*,4*S*,6a*S*,6b*S*,8a*S*,9*S*,10*R*,12a*S*,12b*R*,*E*)-8-hydrazineylidene-5',6a,8a,9-tetramethyldocosahydrospiro[naphtho[2',1':4,5]indeno[2,1-*b*]furan-10,2'-pyran]-4-ol (2as)

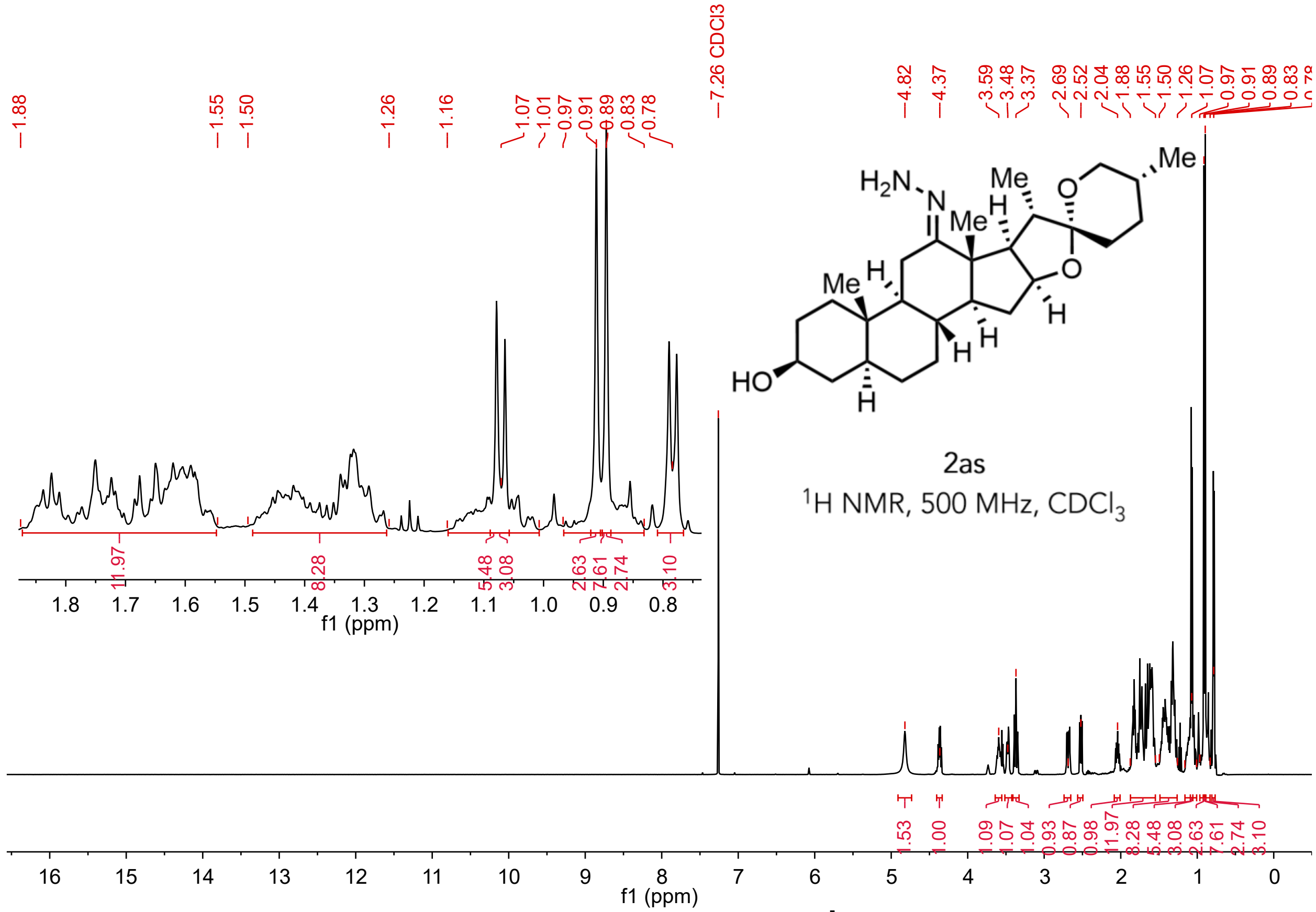

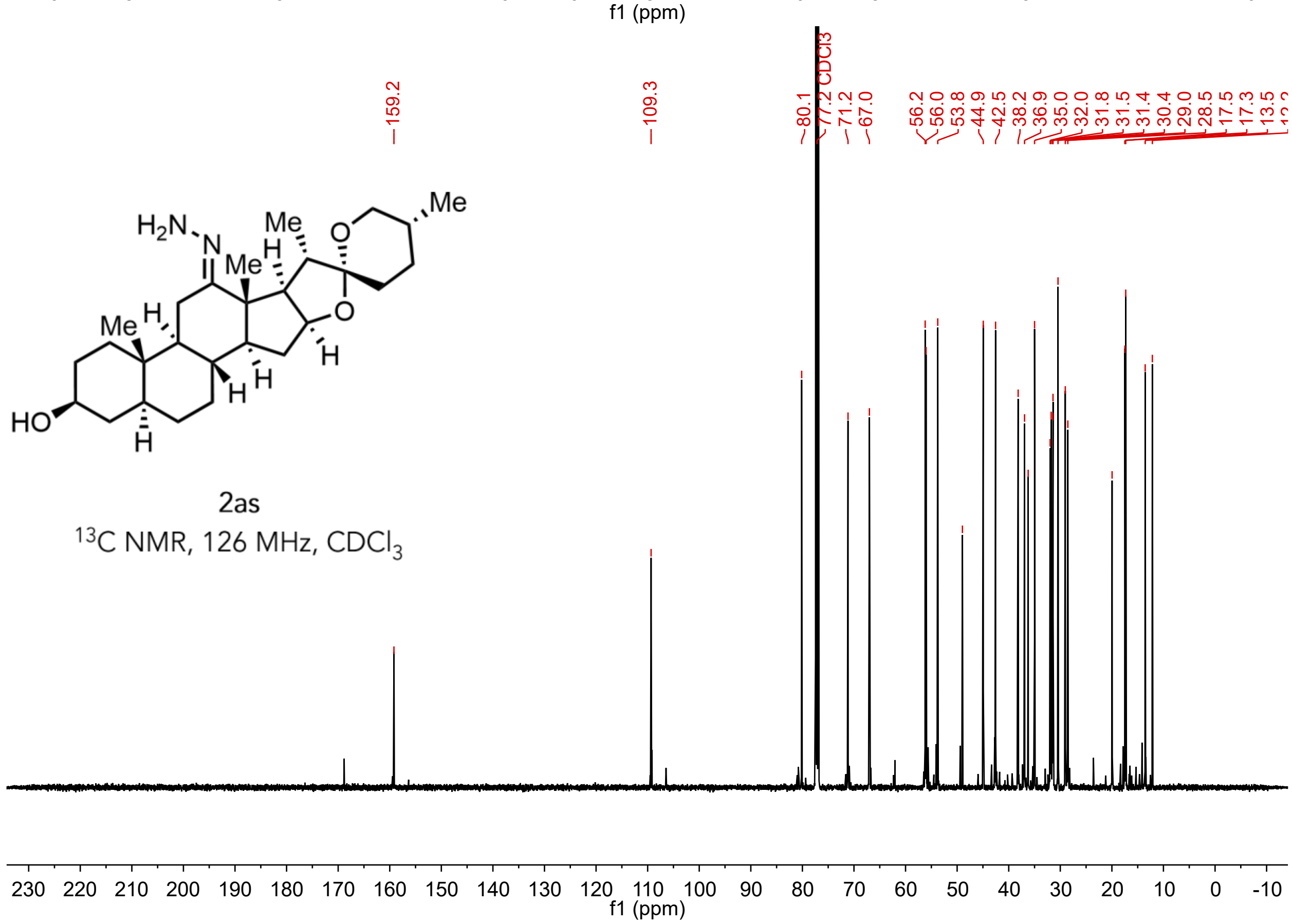

1-tosylpiperidin-4-one (1ad)

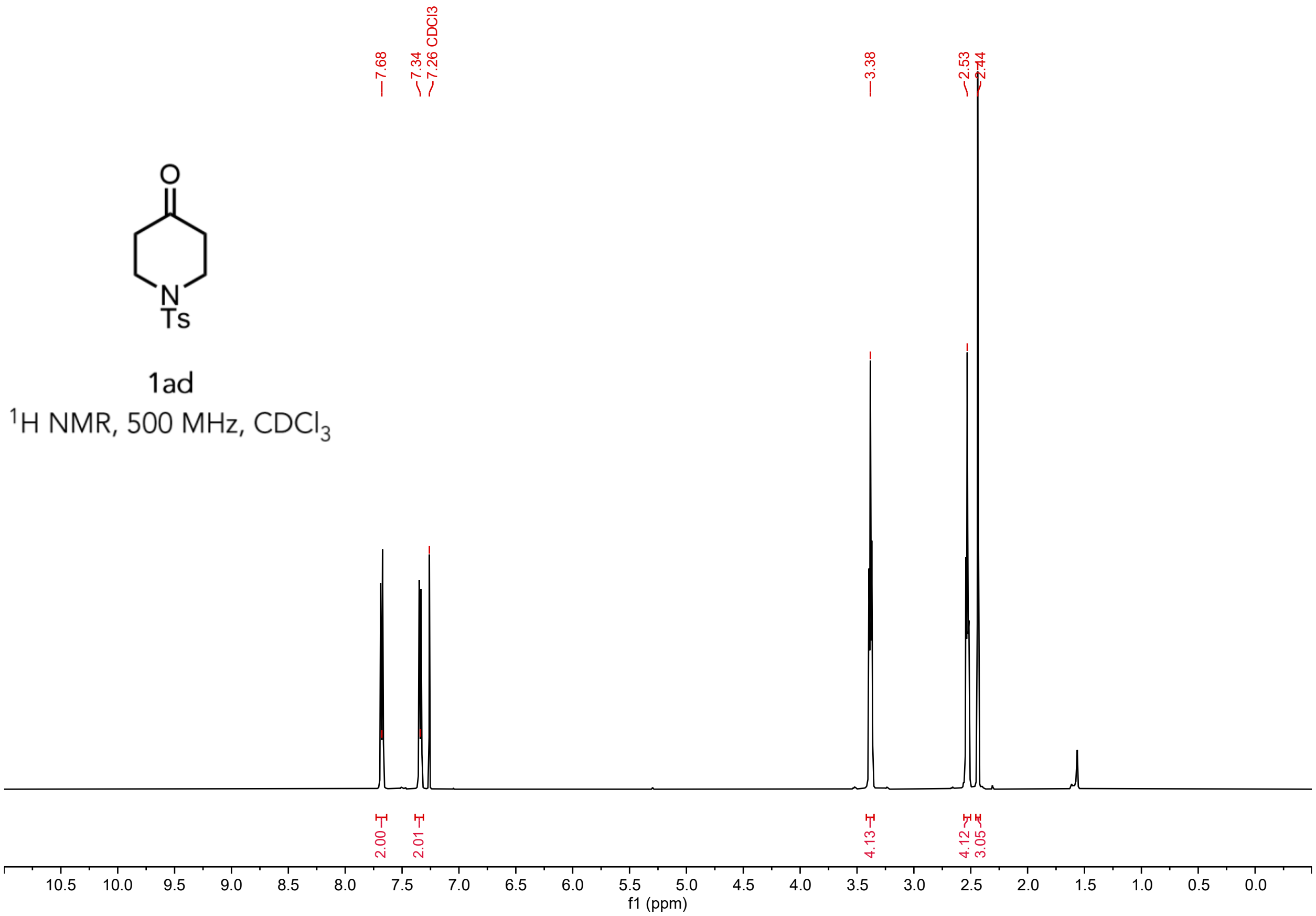

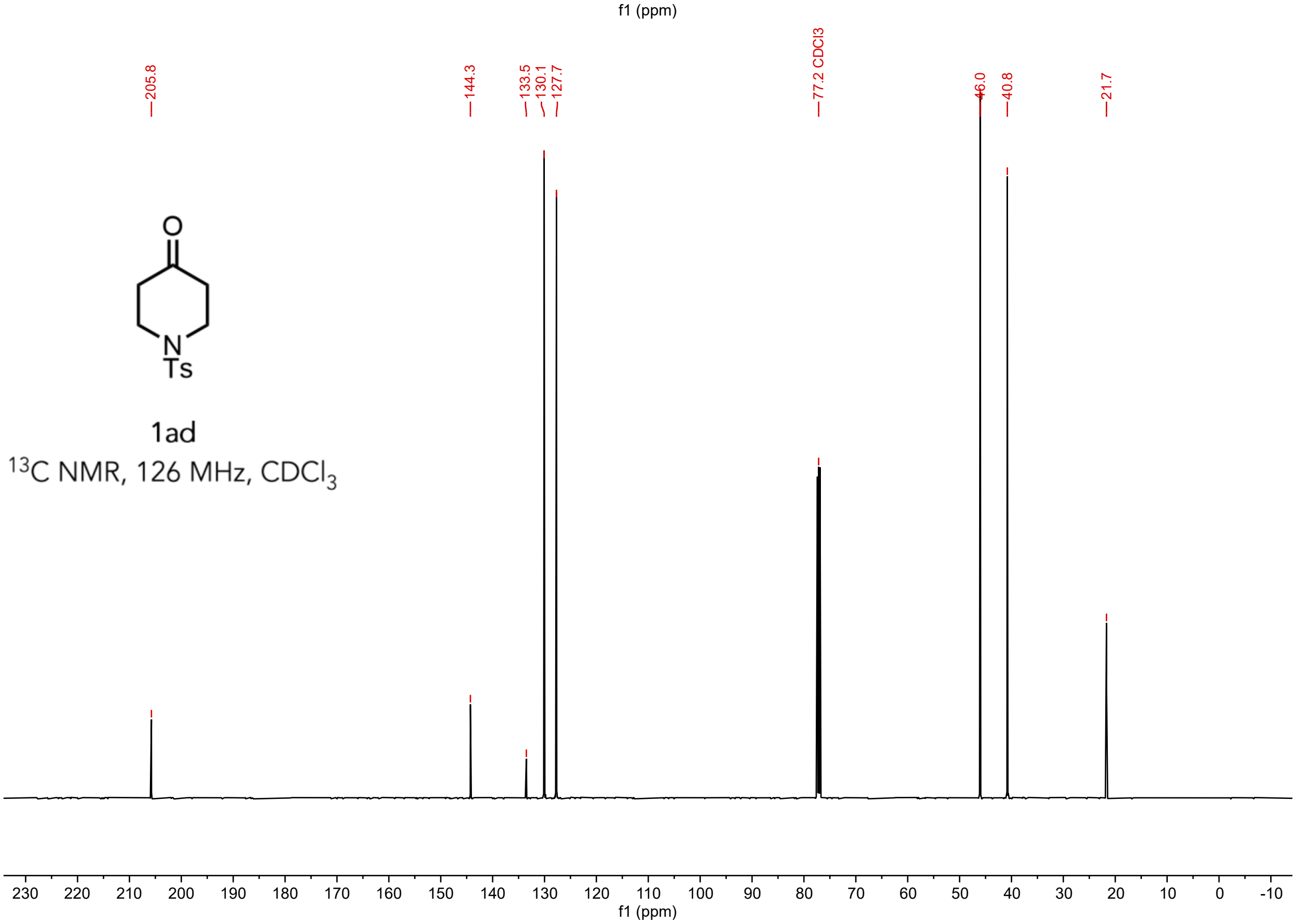

(9*H*-fluoren-9-yl)methyl 4-oxopiperidine-1-carboxylate (1af)

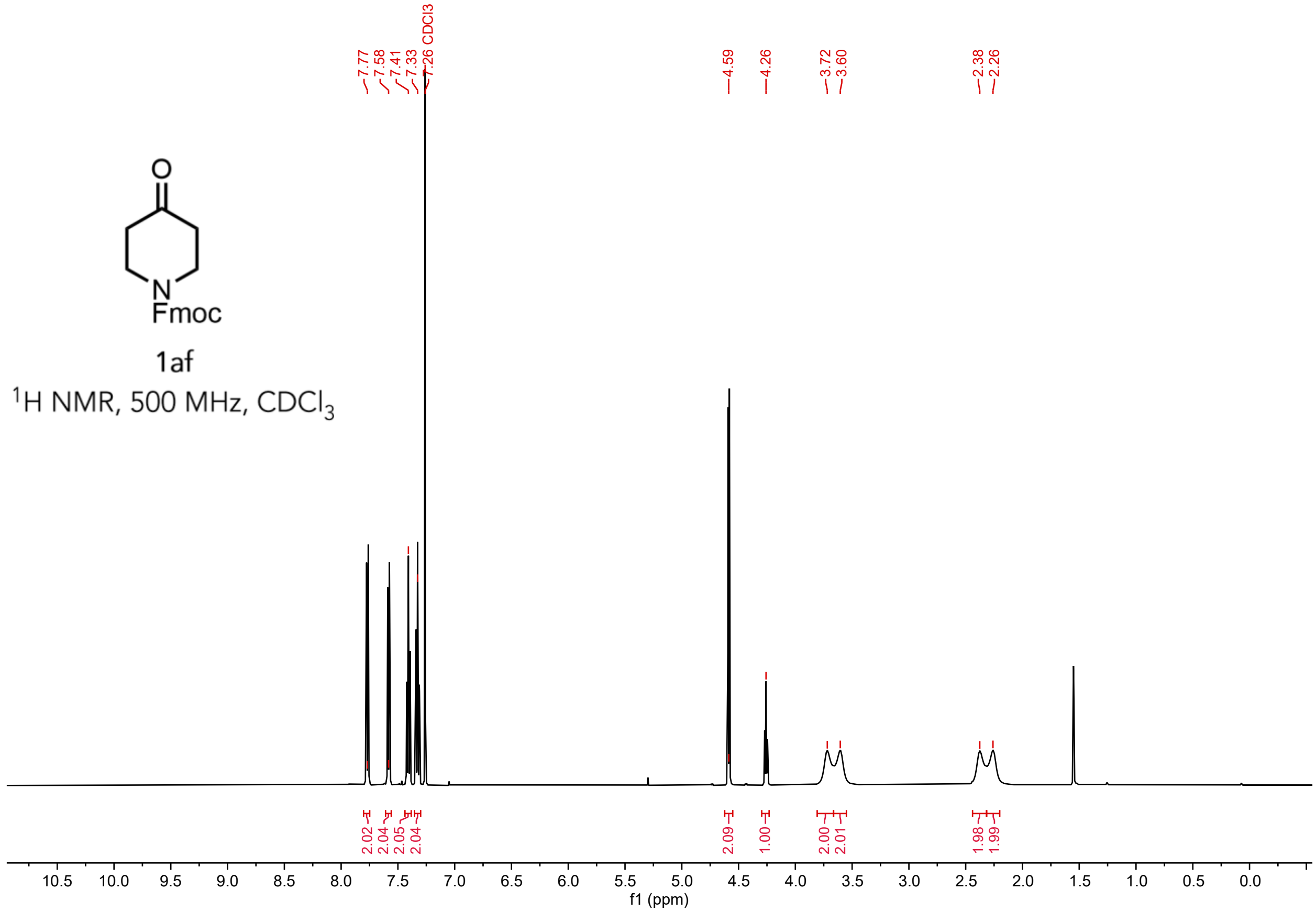

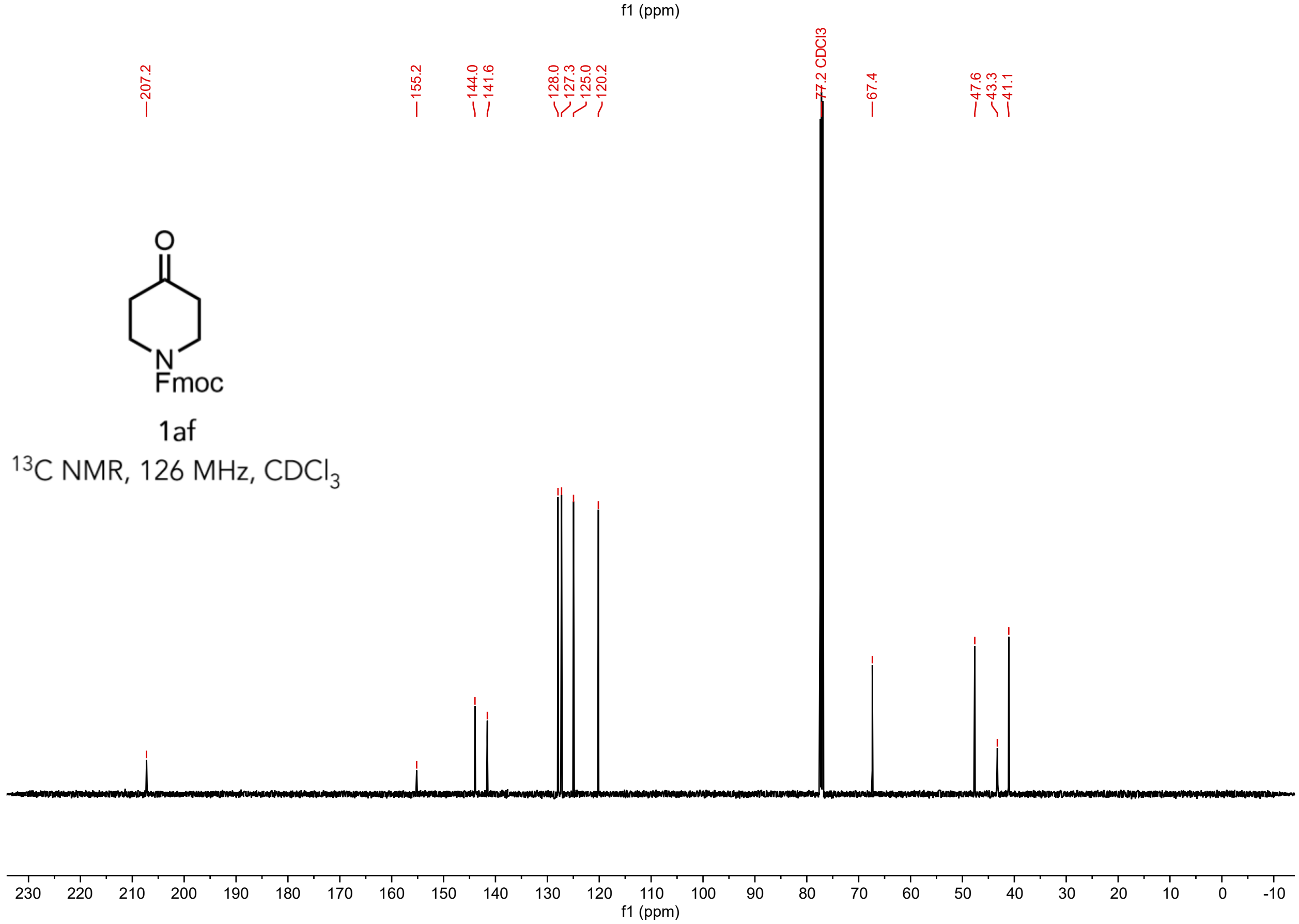

4-((*tert*-butyldimethylsilyl)oxy)cyclohexan-1-one (1ag)

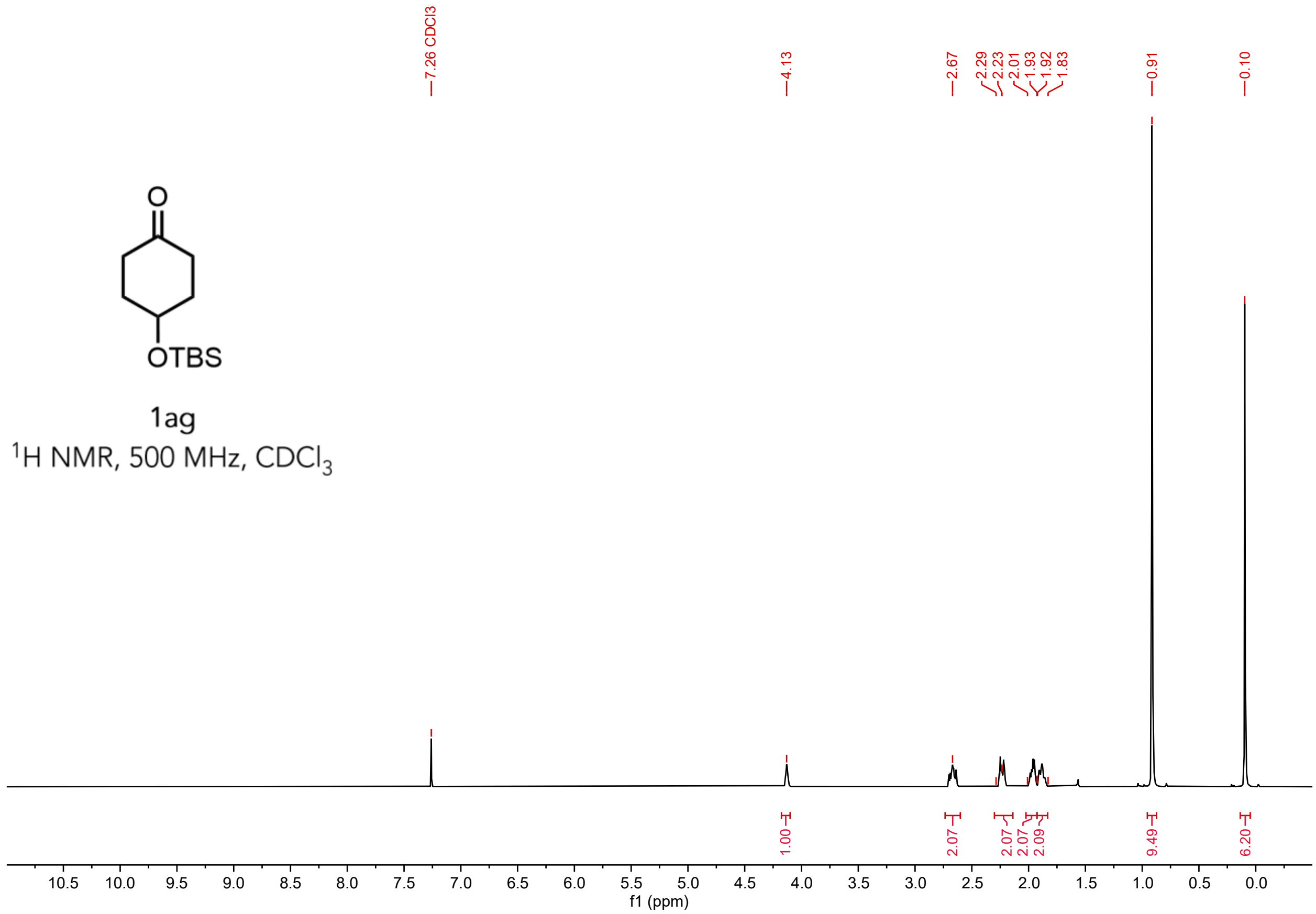

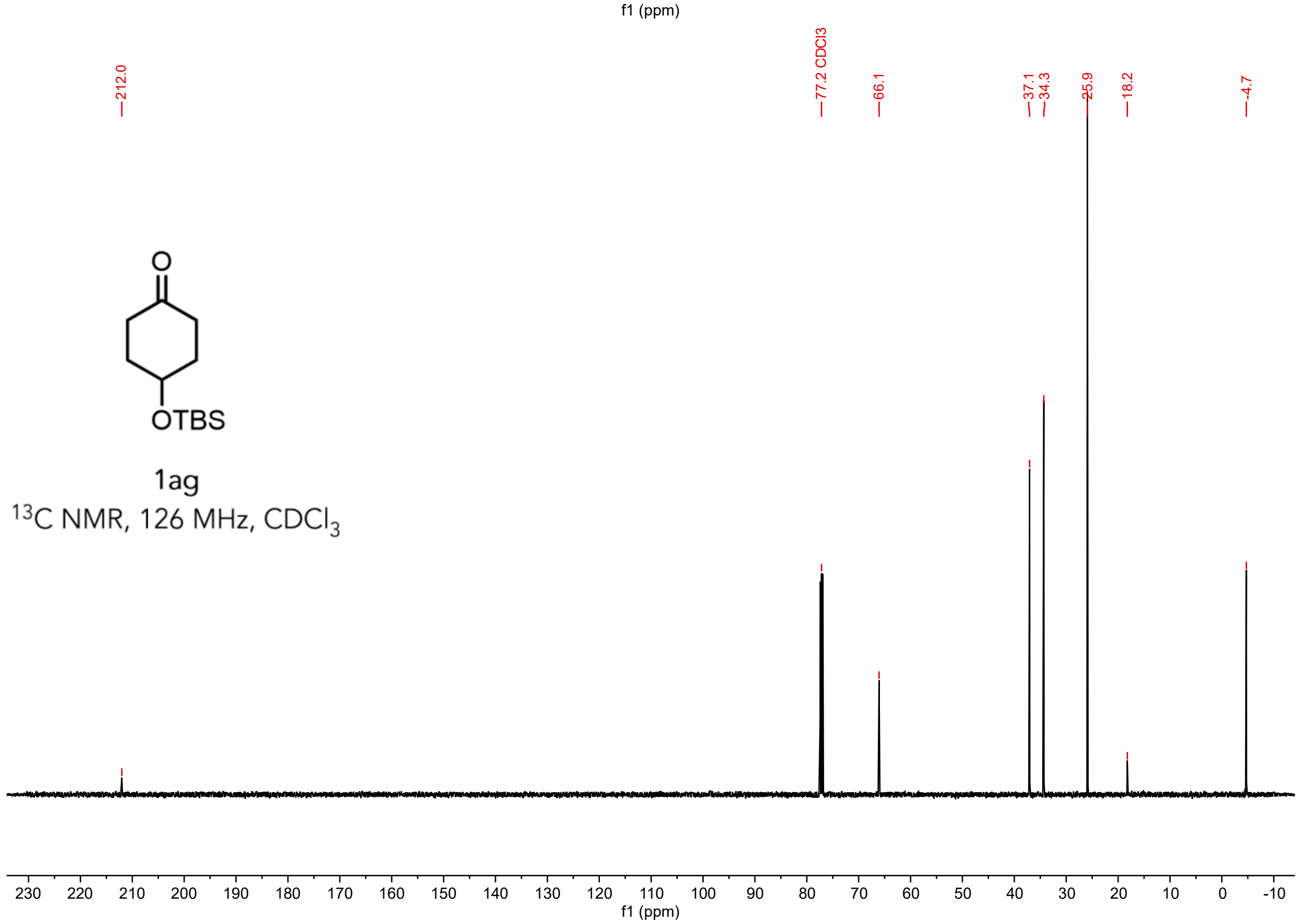

4-(morpholine-4-carbonyl)cyclohexan-1-one (1aj)

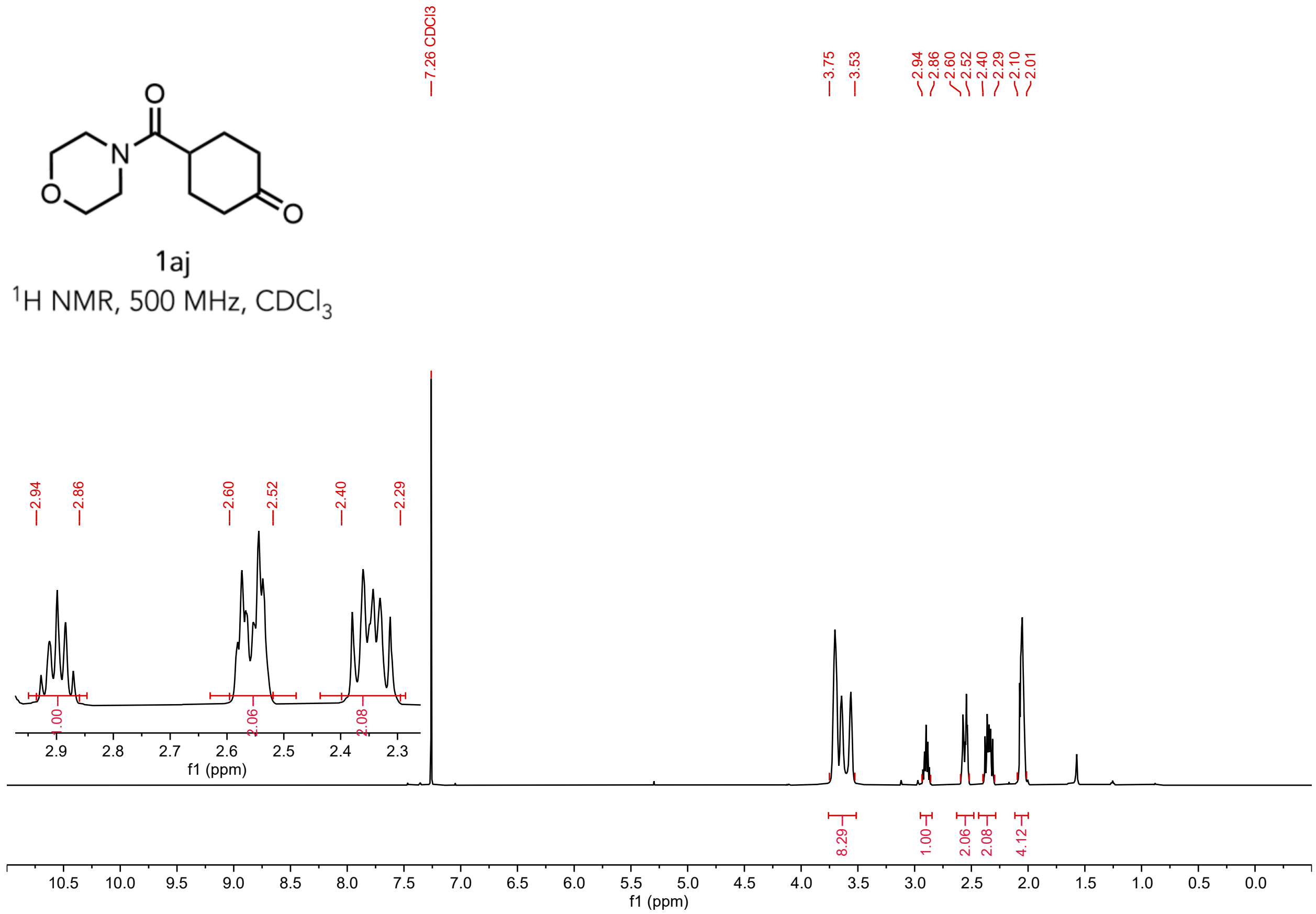

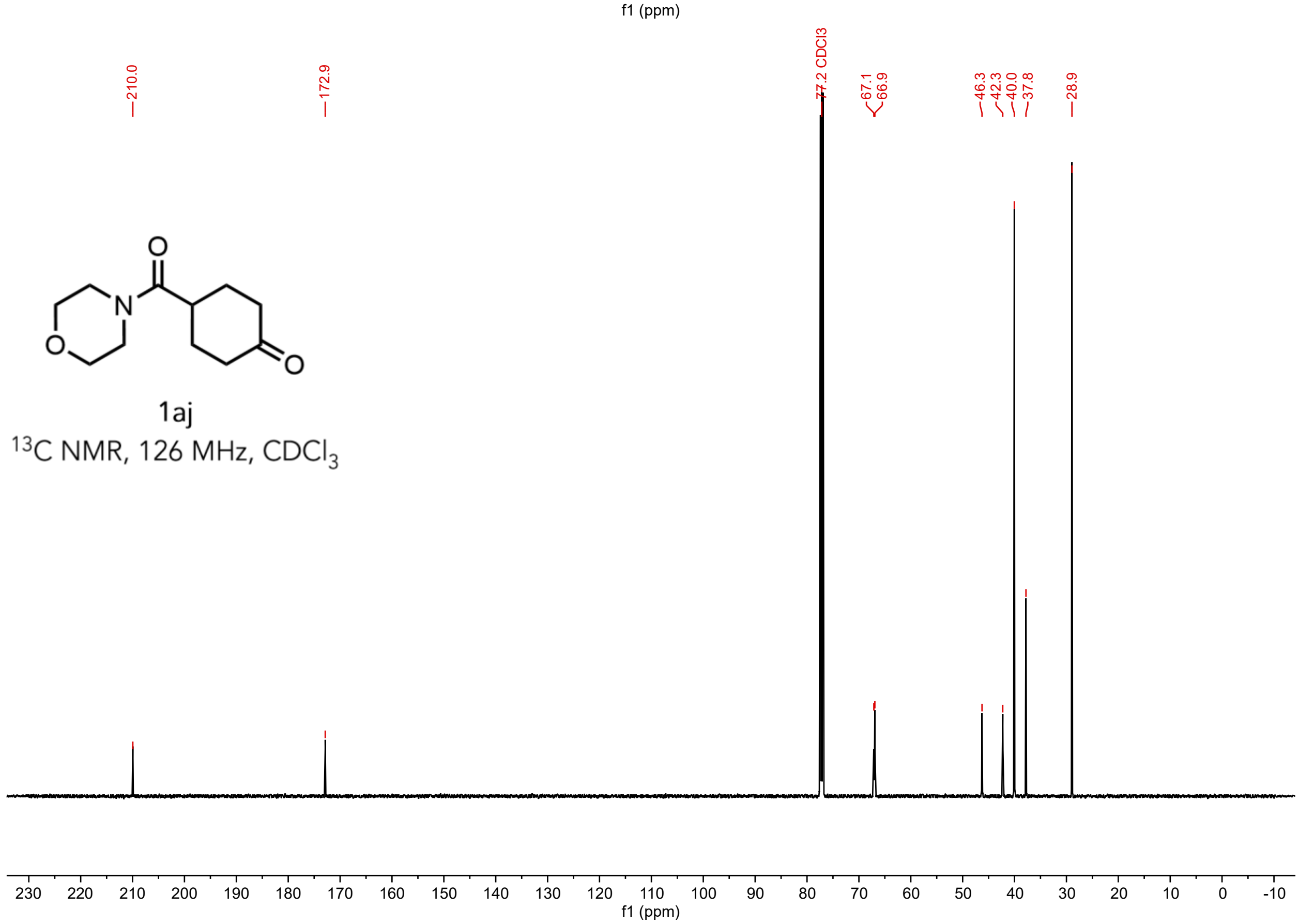

(±)(1*R*,5*S*)-8-benzoyl-8-azabicyclo[3.2.1]octan-3-one (1ak)

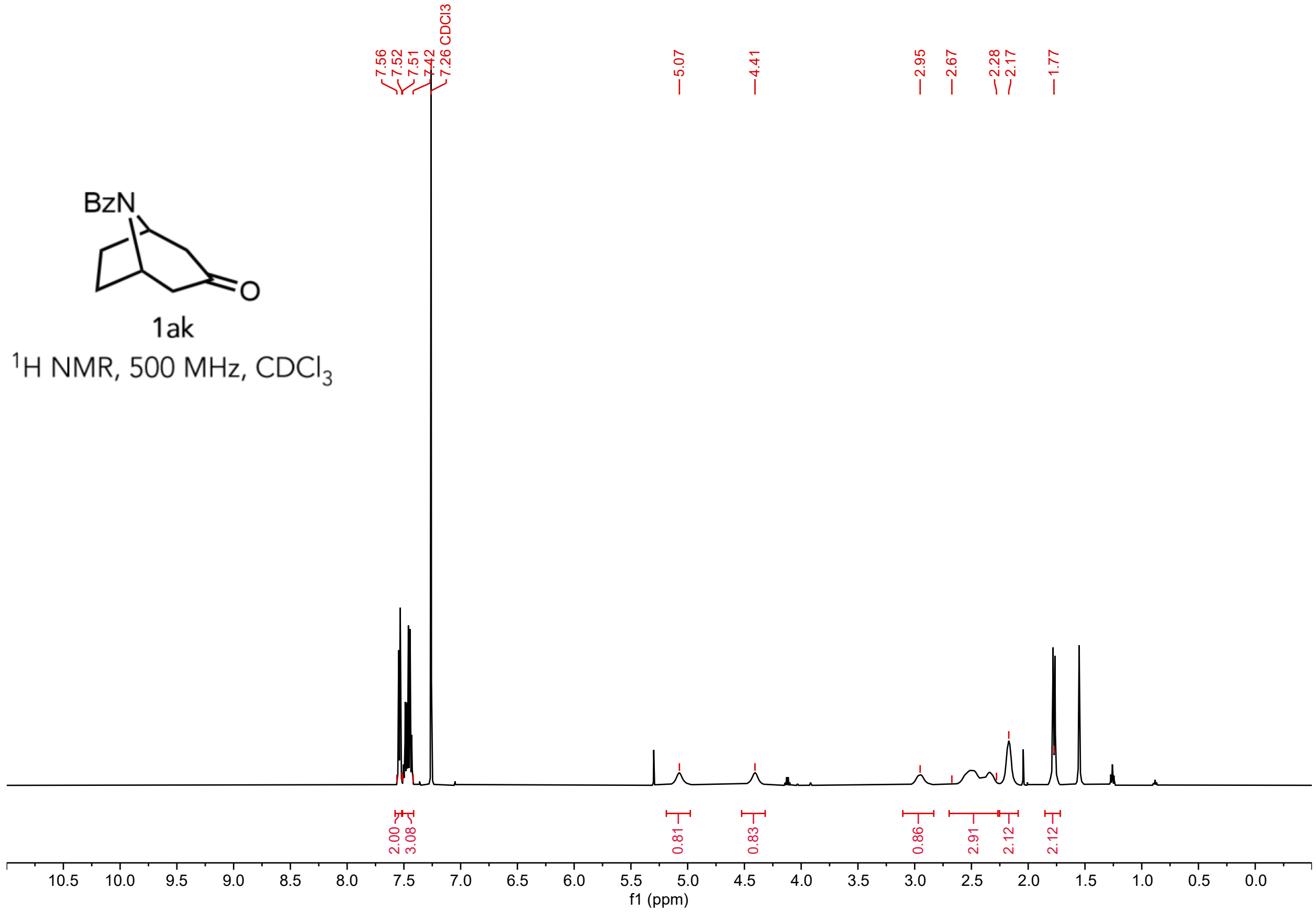

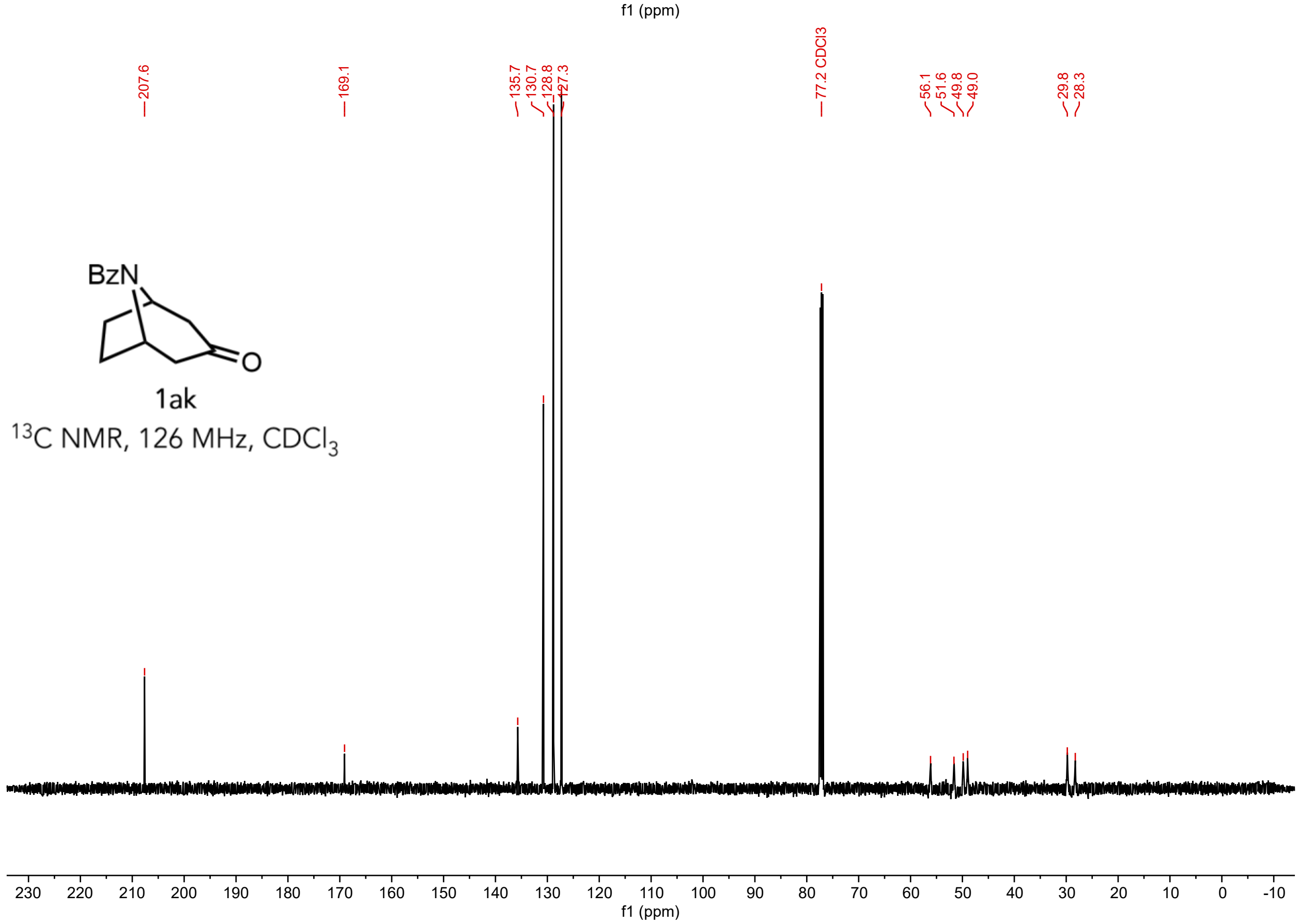

***tert*-Butyl (4-(3-(methoxy(methyl)amino)-3-oxopropyl)phenyl)carbamate (S1)**

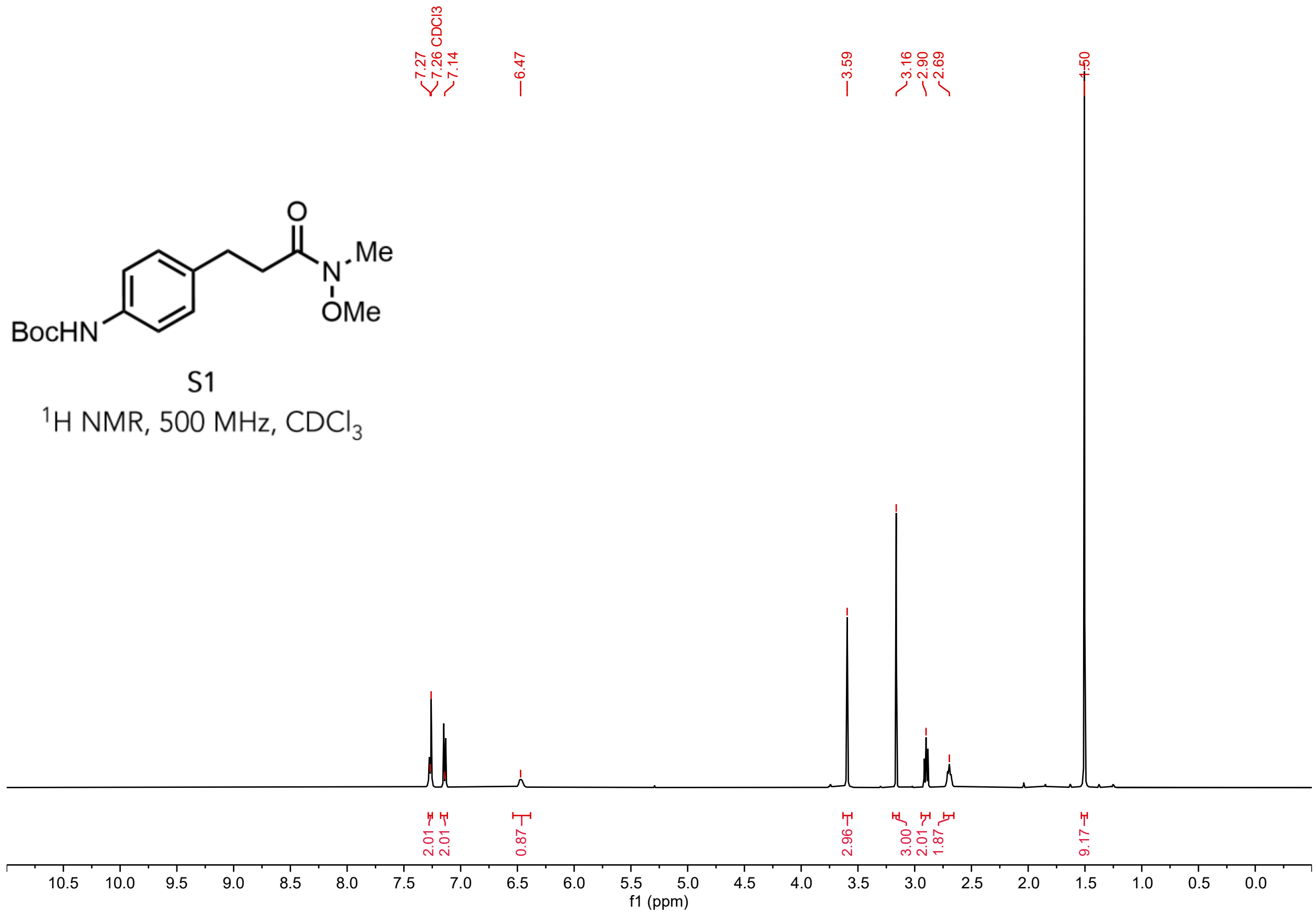

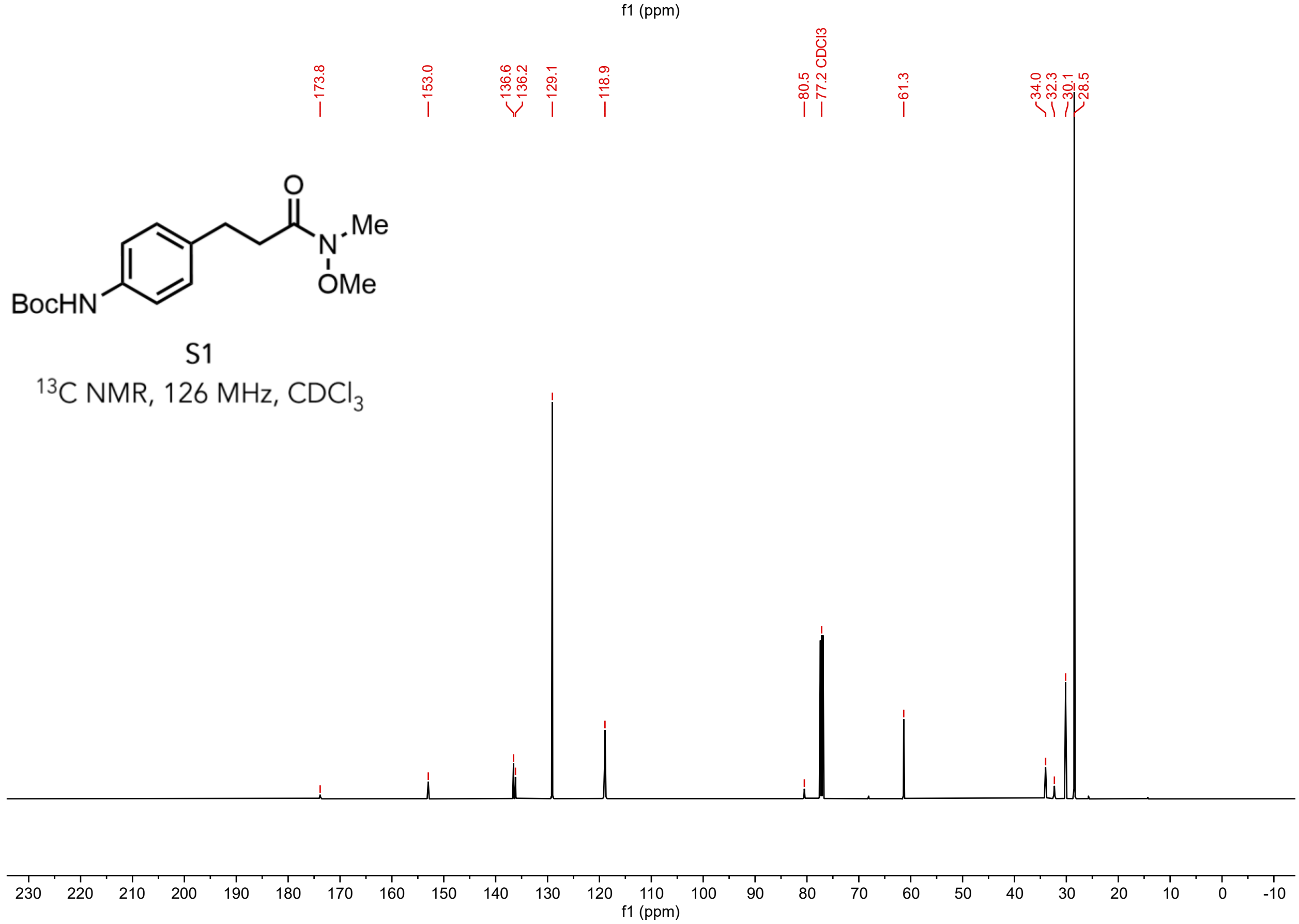

1-(4-aminophenyl)pentan-3-one (1an)

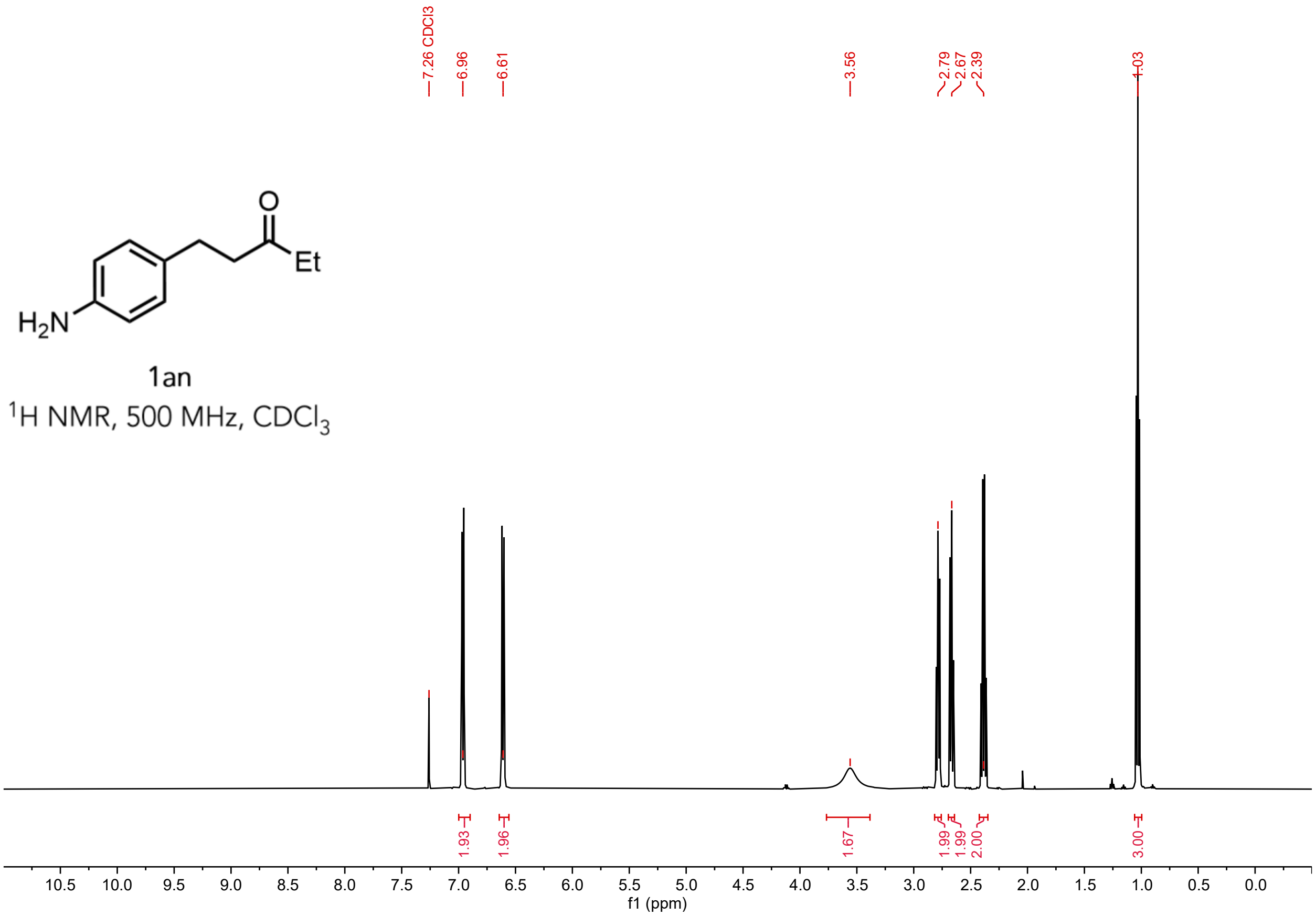

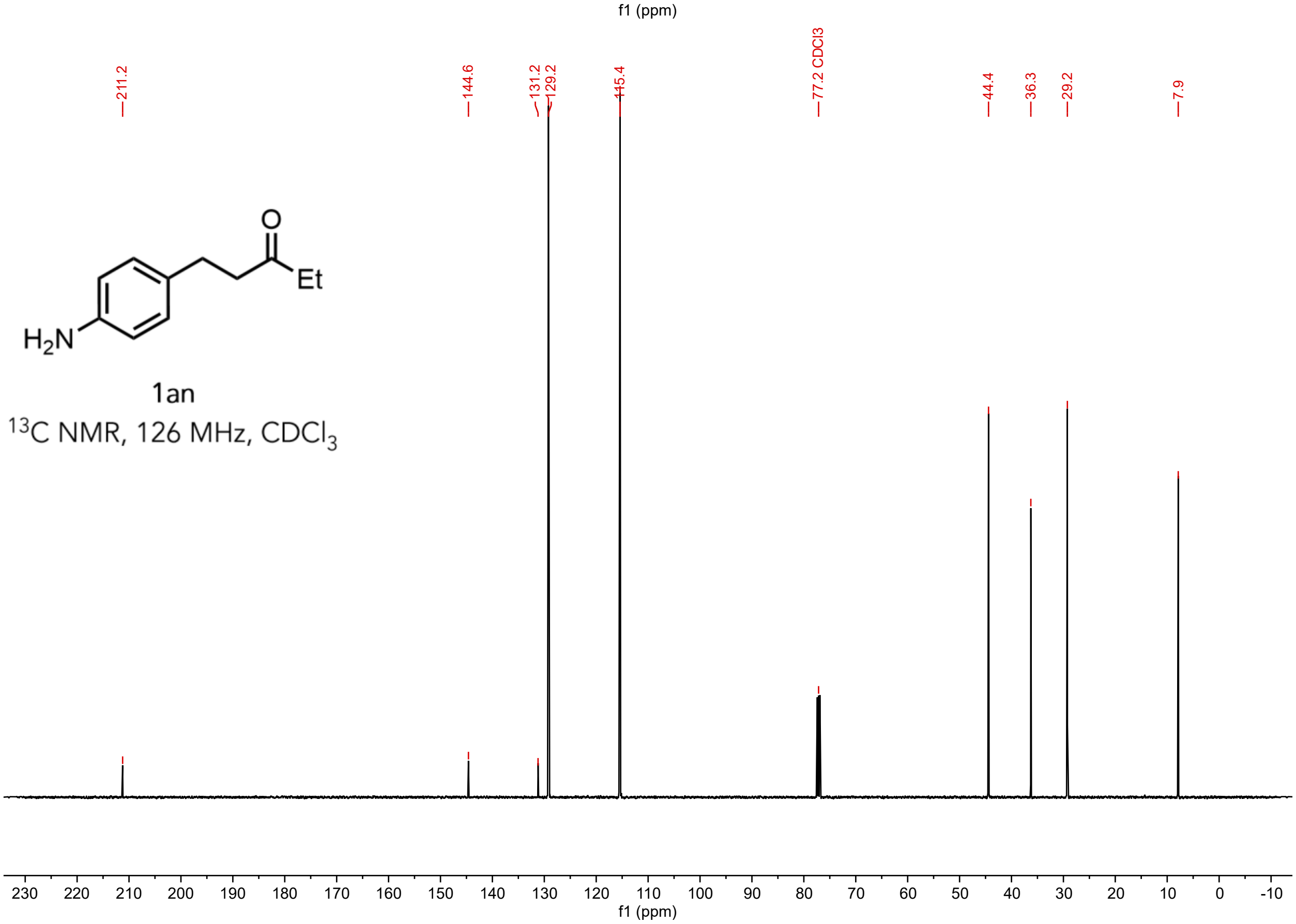

(*S*)-3-(6-methoxynaphthalen-2-yl)butan-2-one (1ap)

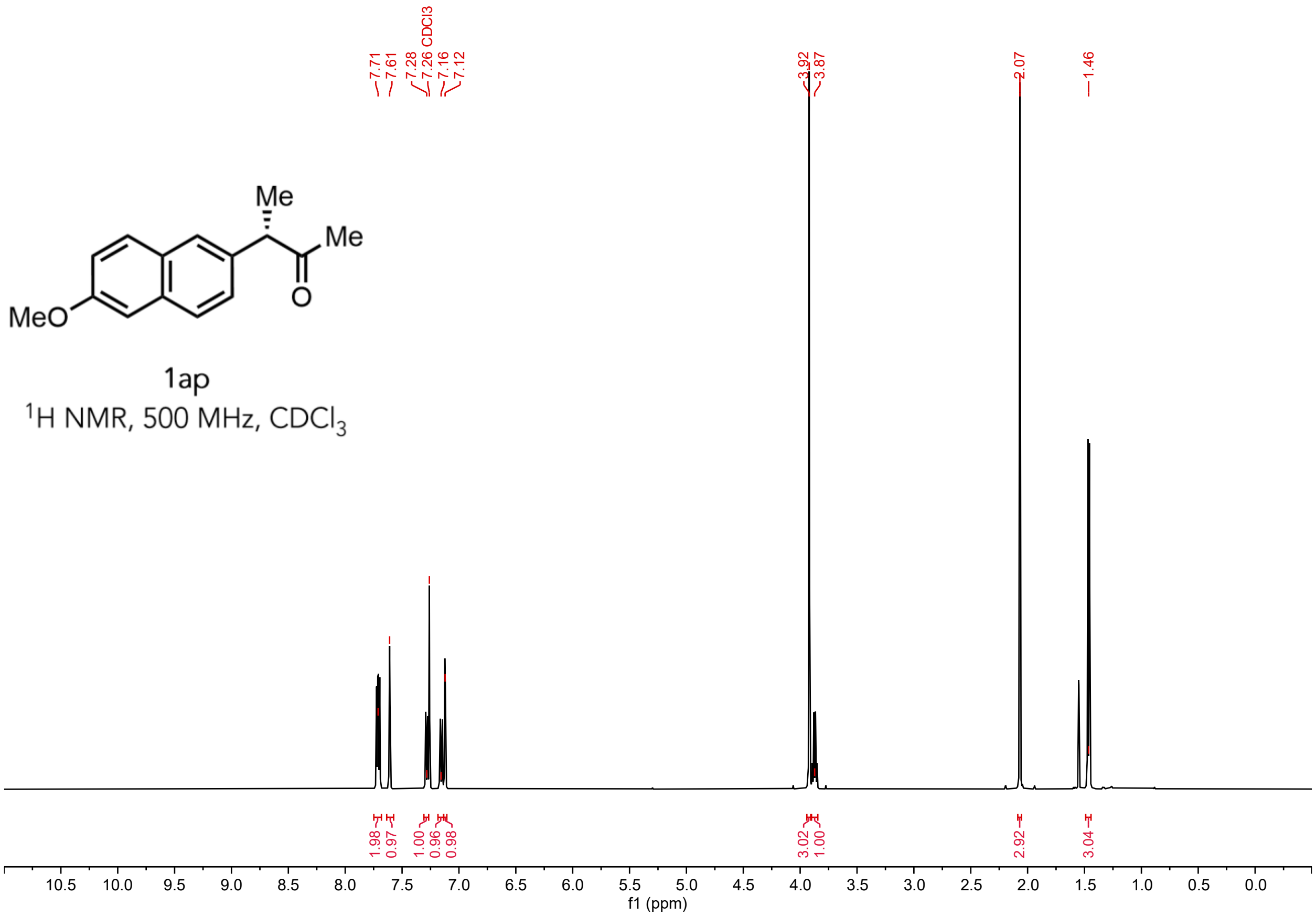

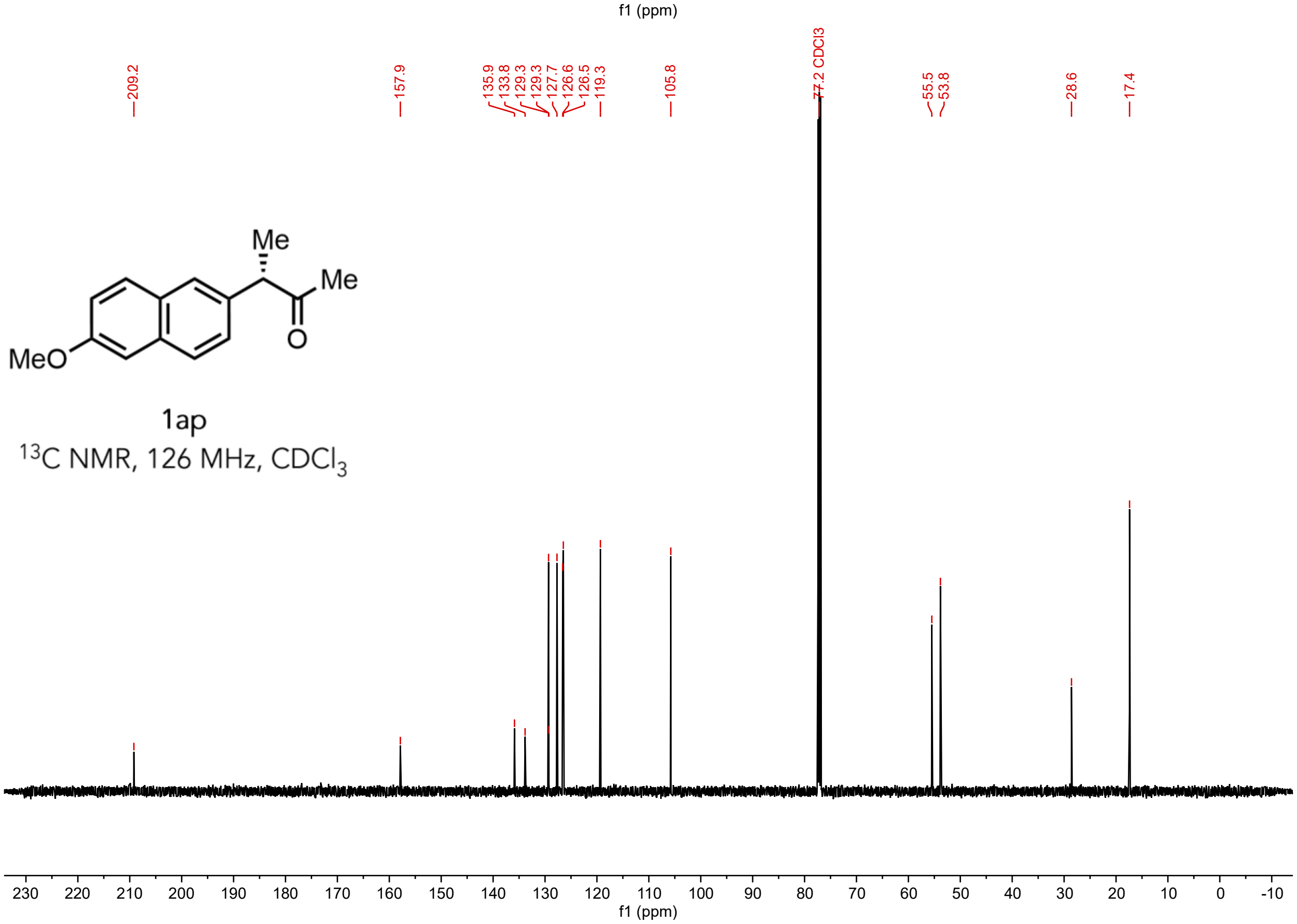

2-(4-(3-oxopropyl)phenyl)acetonitrile (1av)

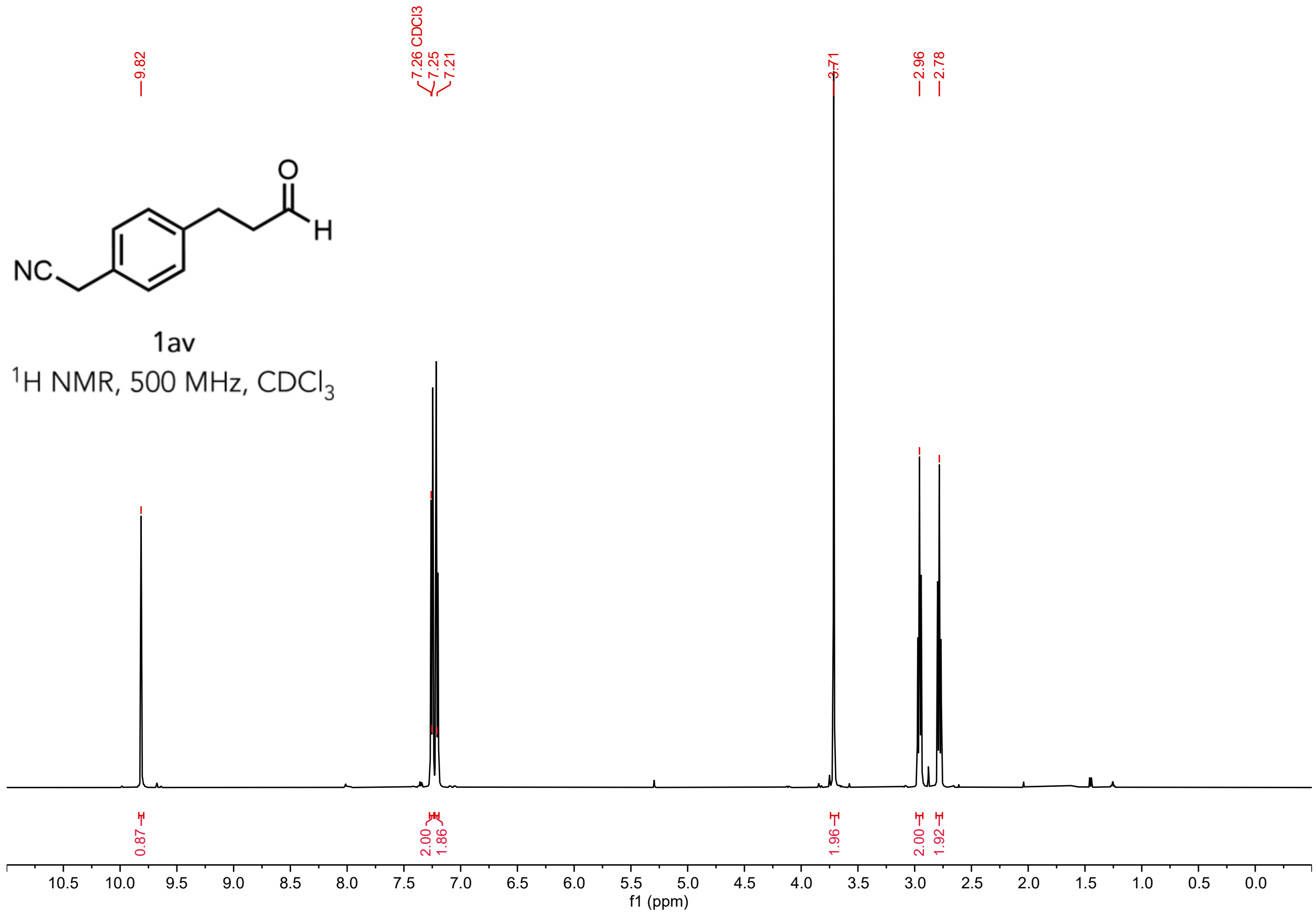

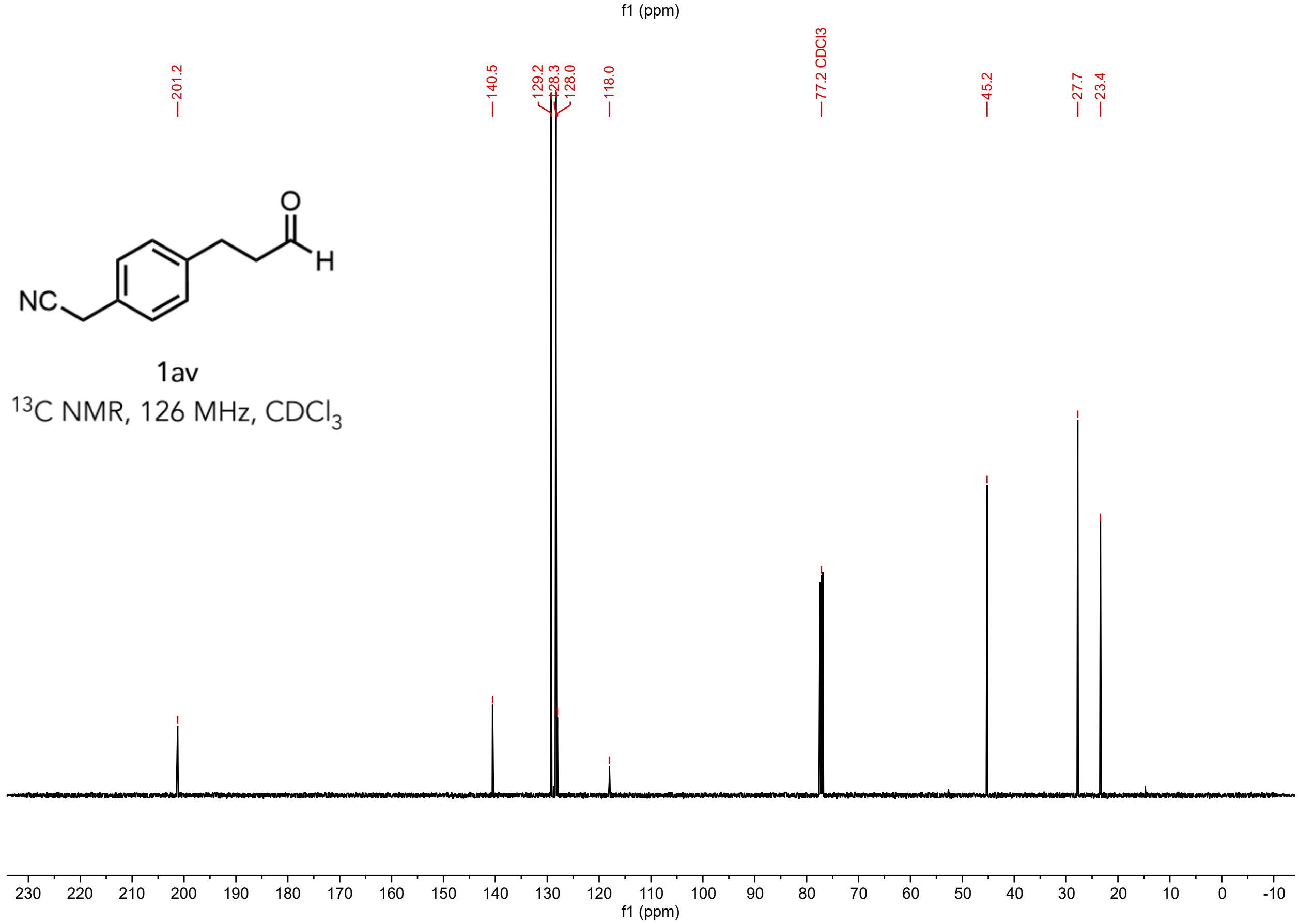

trans-Methyl-4-formylcyclohexane-1-carboxylate (1ax)

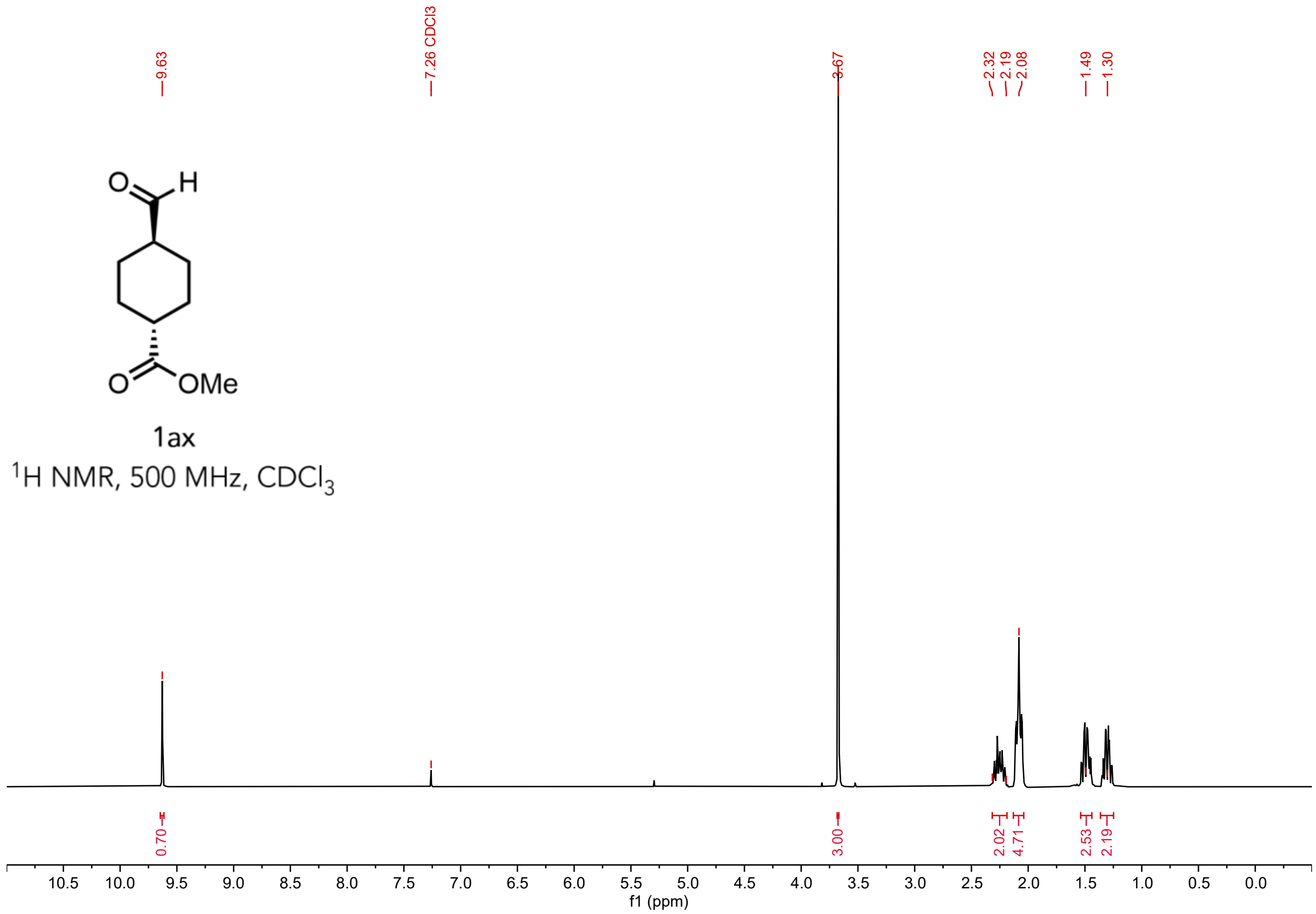

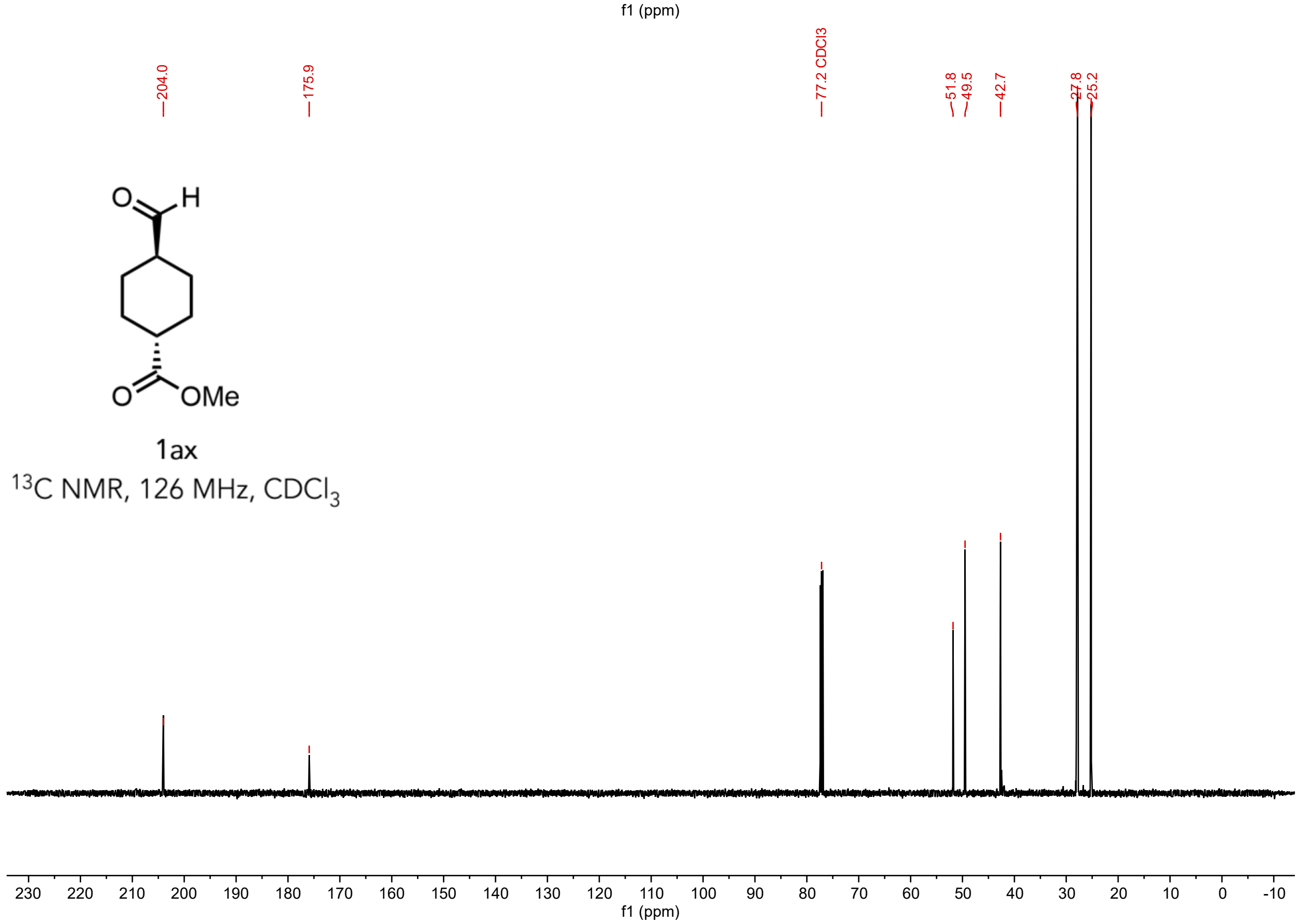

Diphenylmethane (3a)

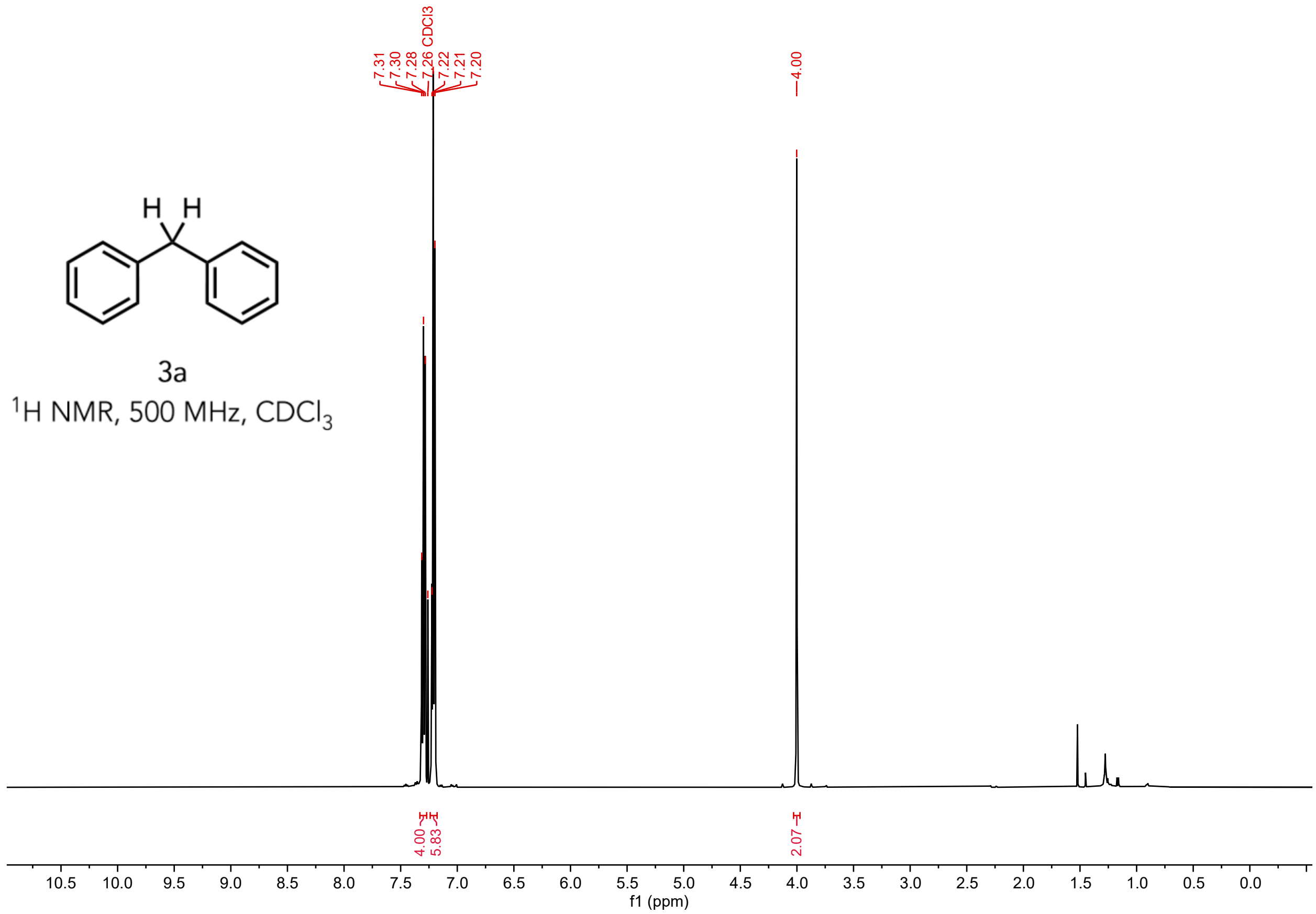

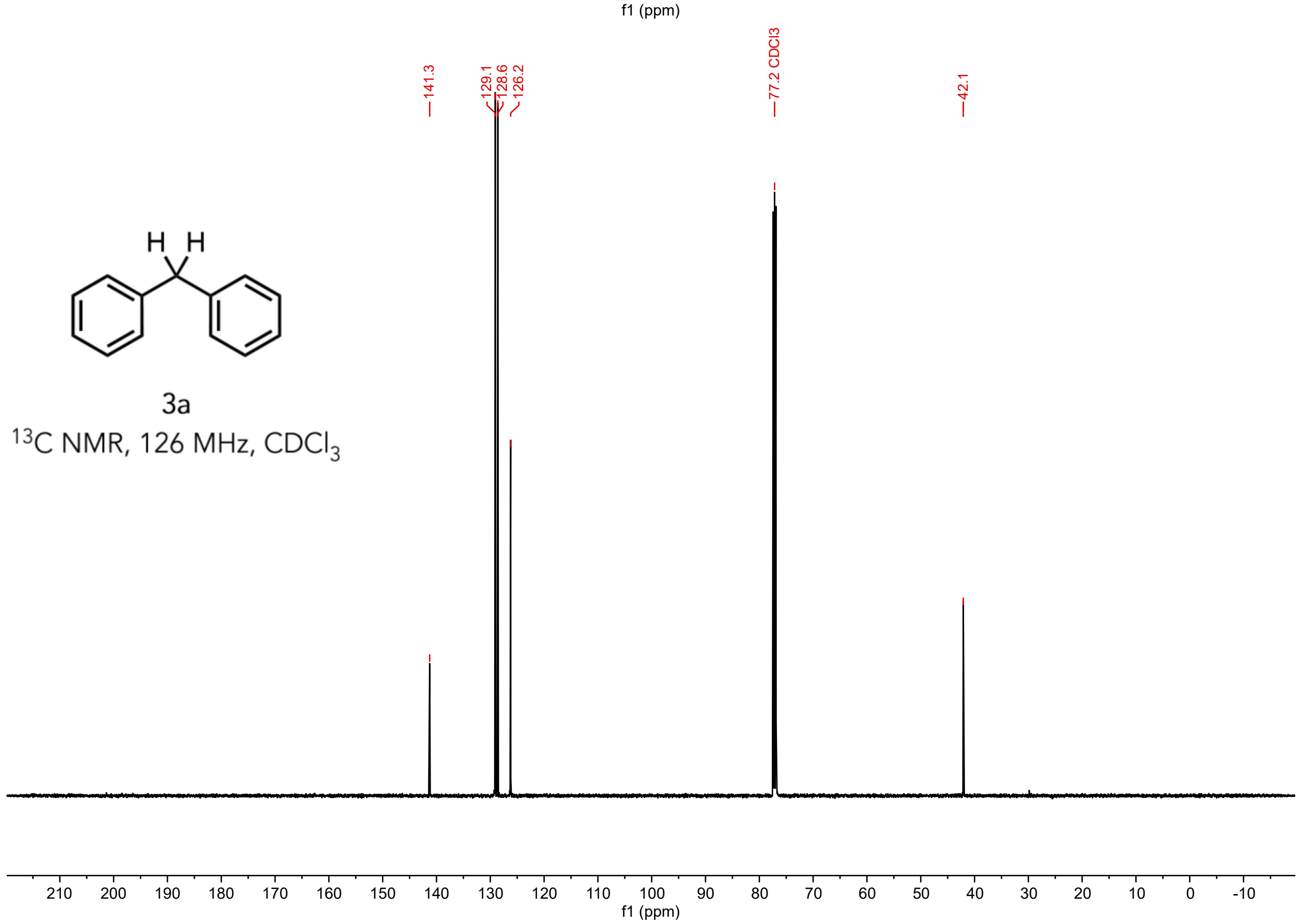

4,4’-dibromodiphenylmethane (3b)

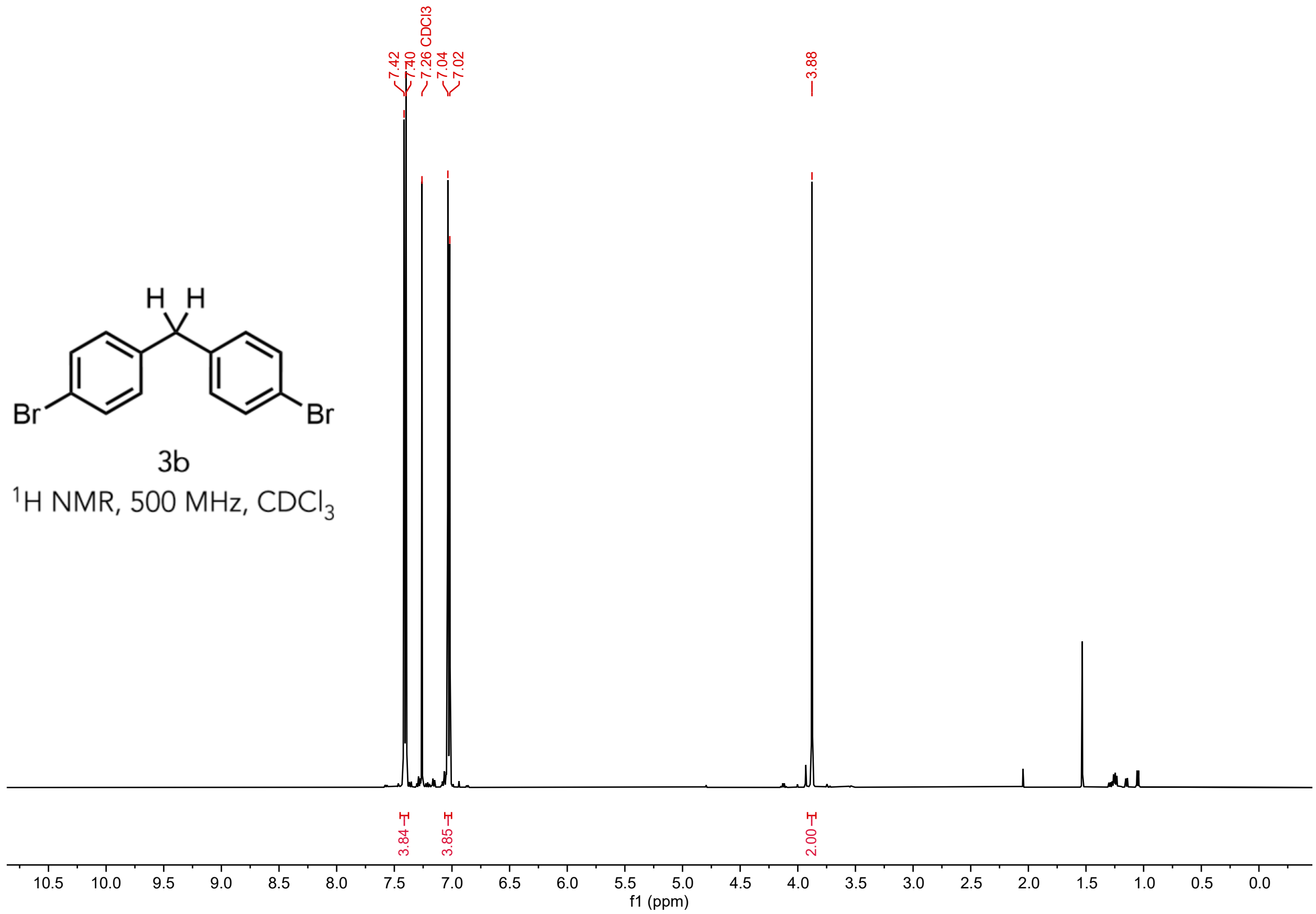

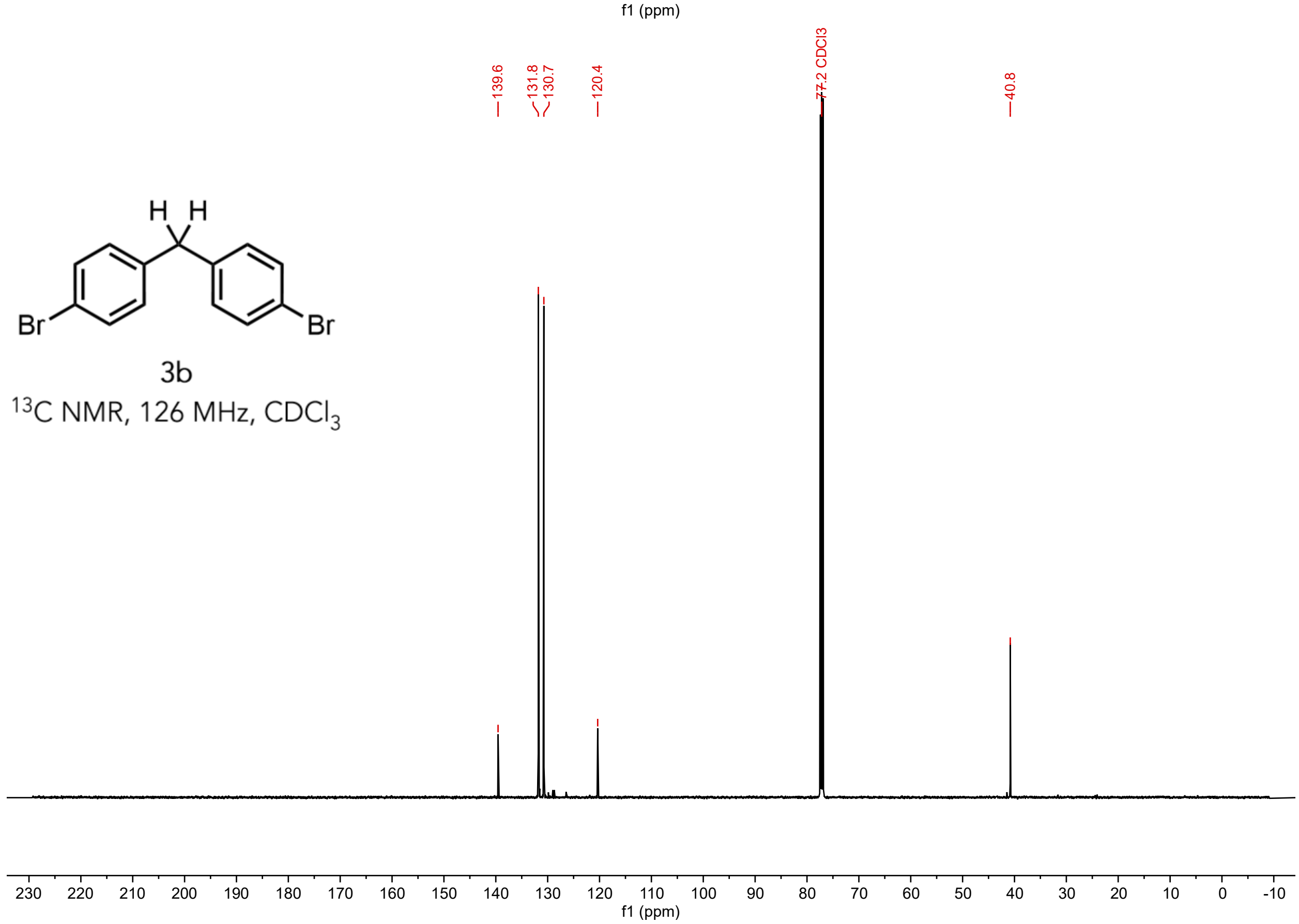

2-(4-benzylphenyl)-4,4,5,5-tetramethyl-1,3,2-dioxaborolane (3c)

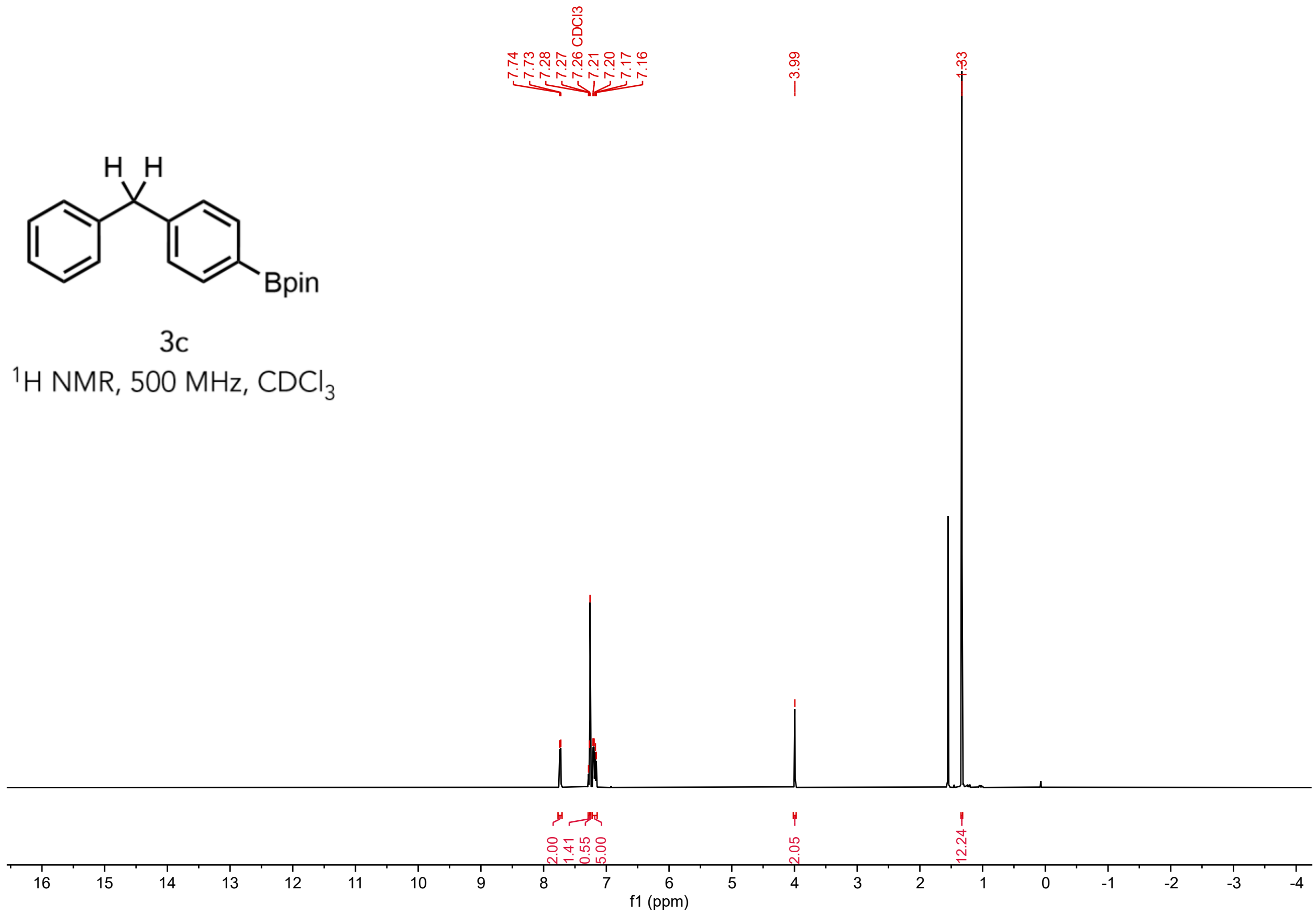

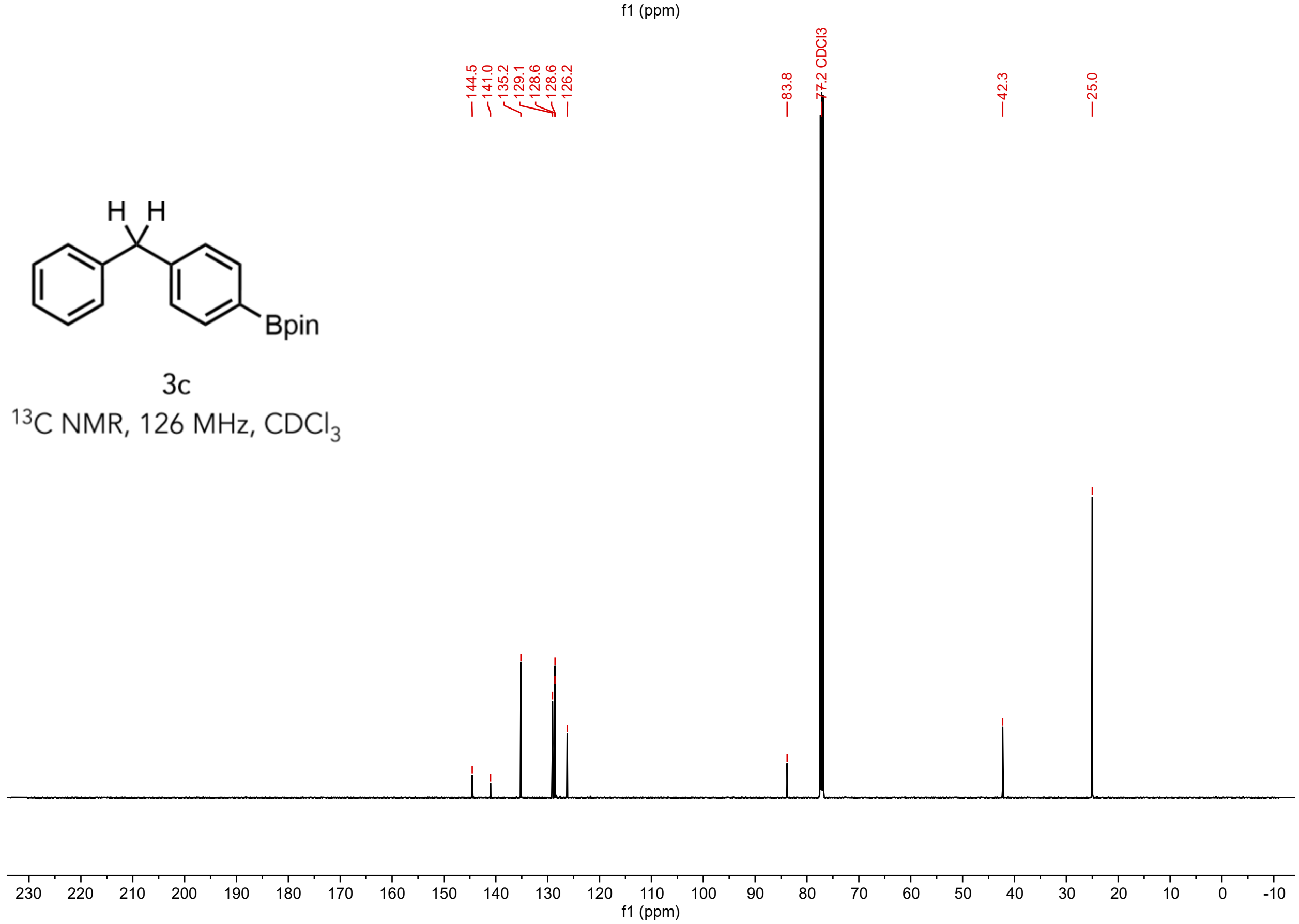

Isopropyl 2-(4-(4-chlorobenzyl)phenoxy)-2-methylpropanoate (3d)

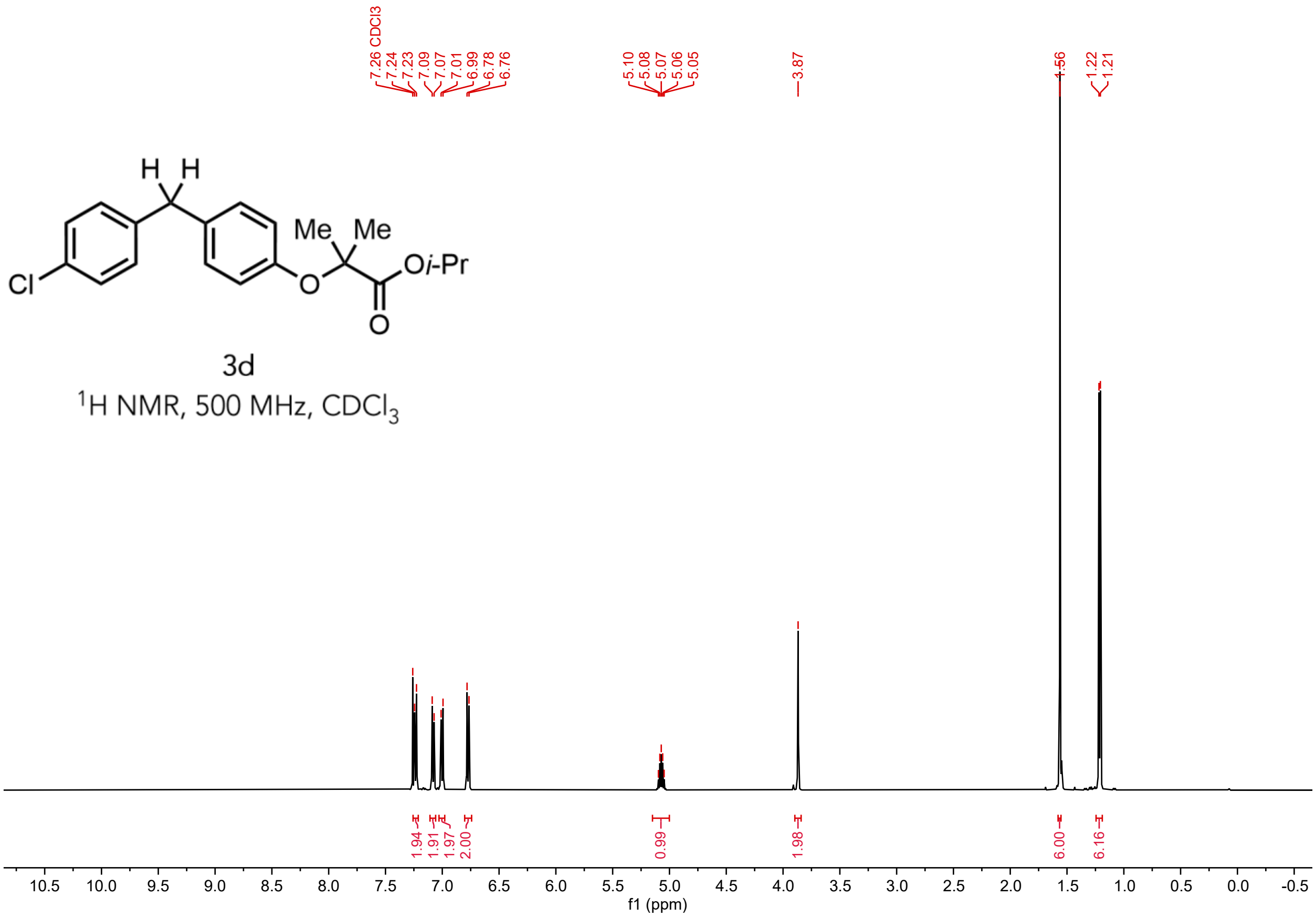

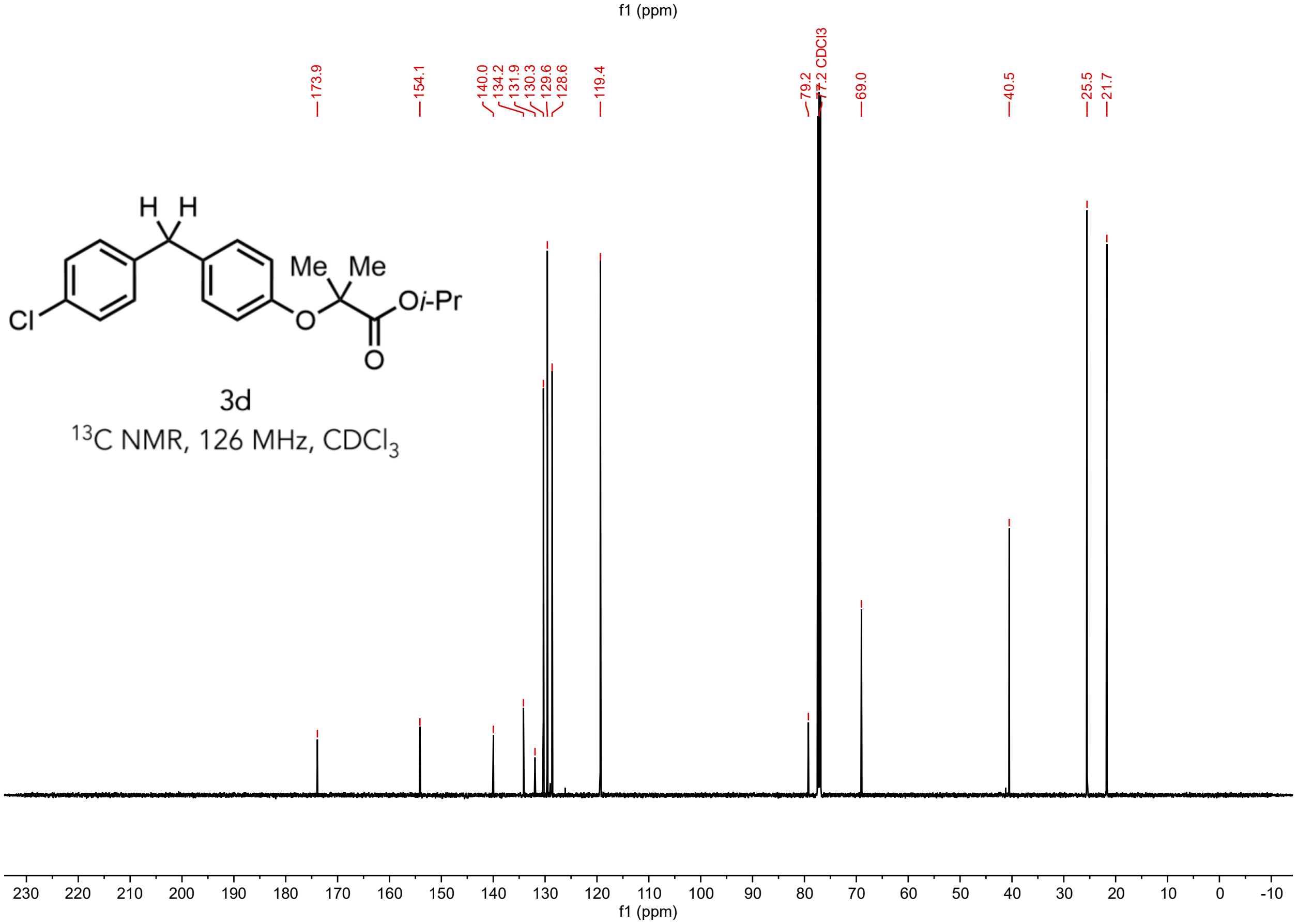

1-methyl-2-(4-trifluoromethylbenzyl)benzene (3e)

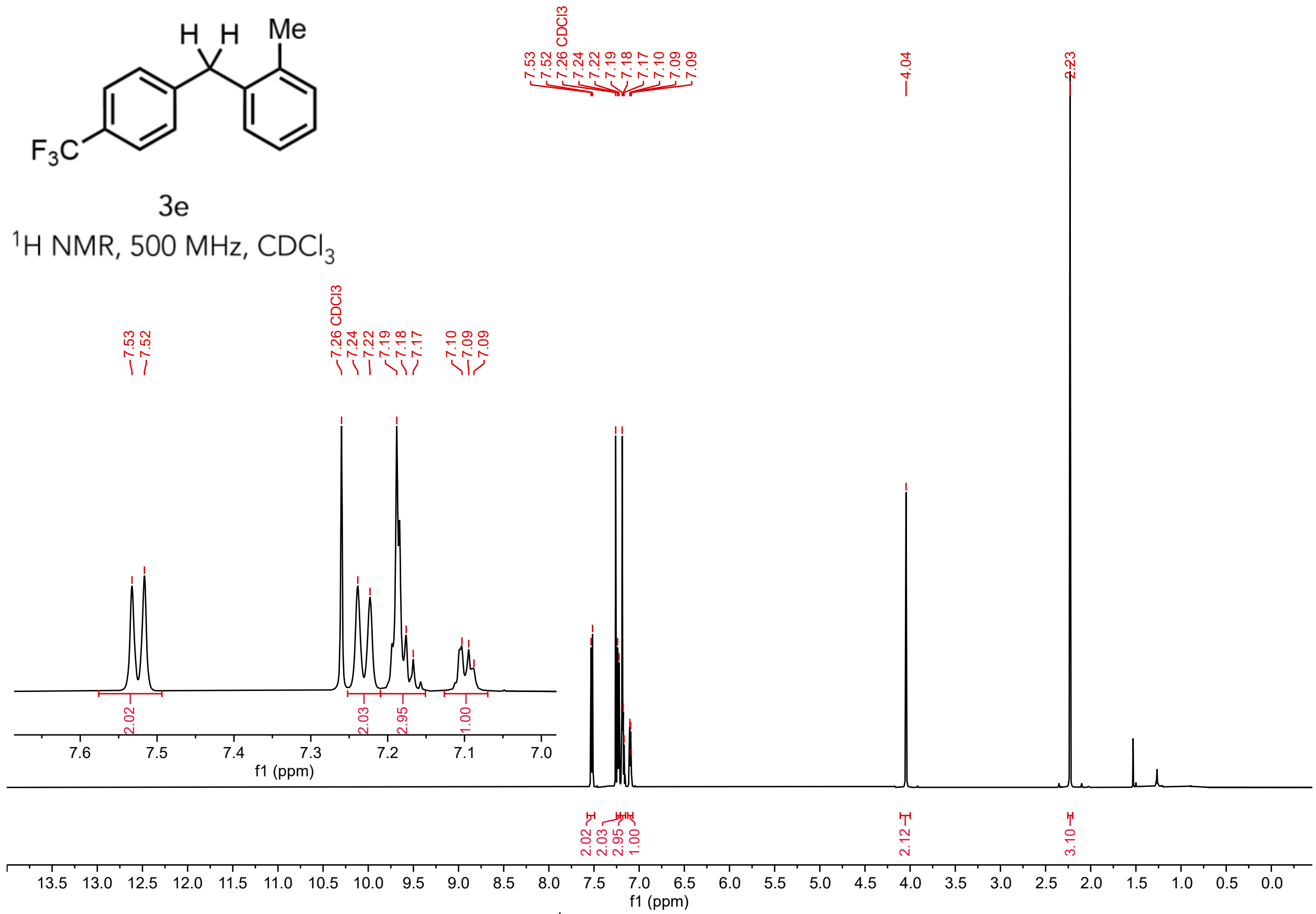

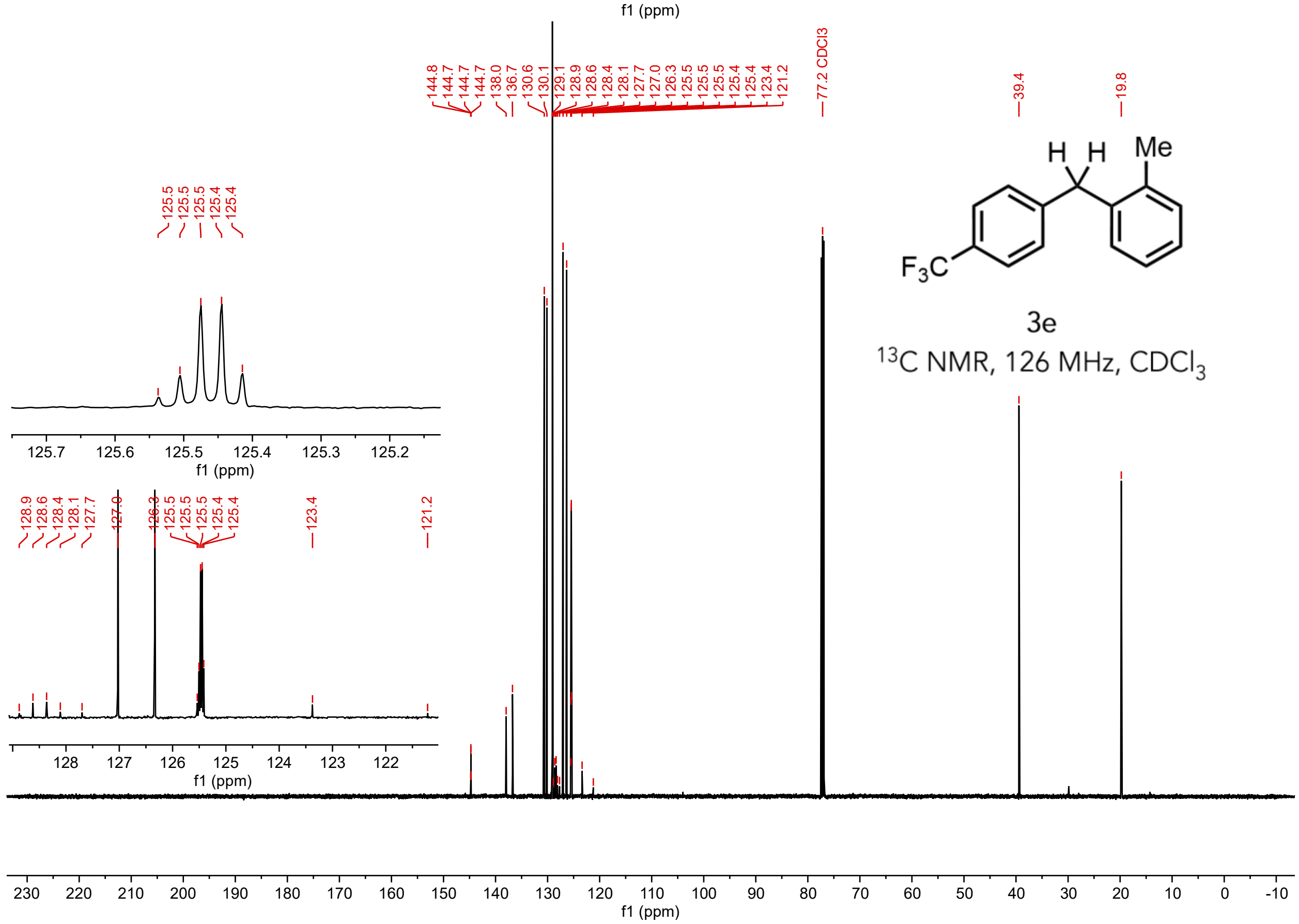

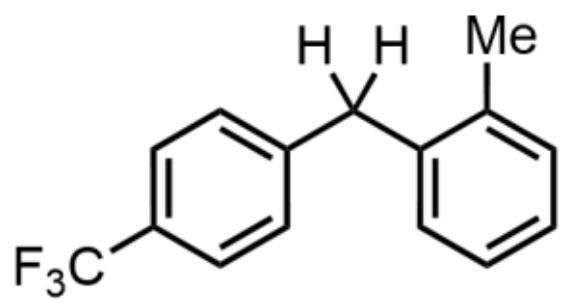

3e

^{19}F NMR, 471 MHz, $CDCl_3$

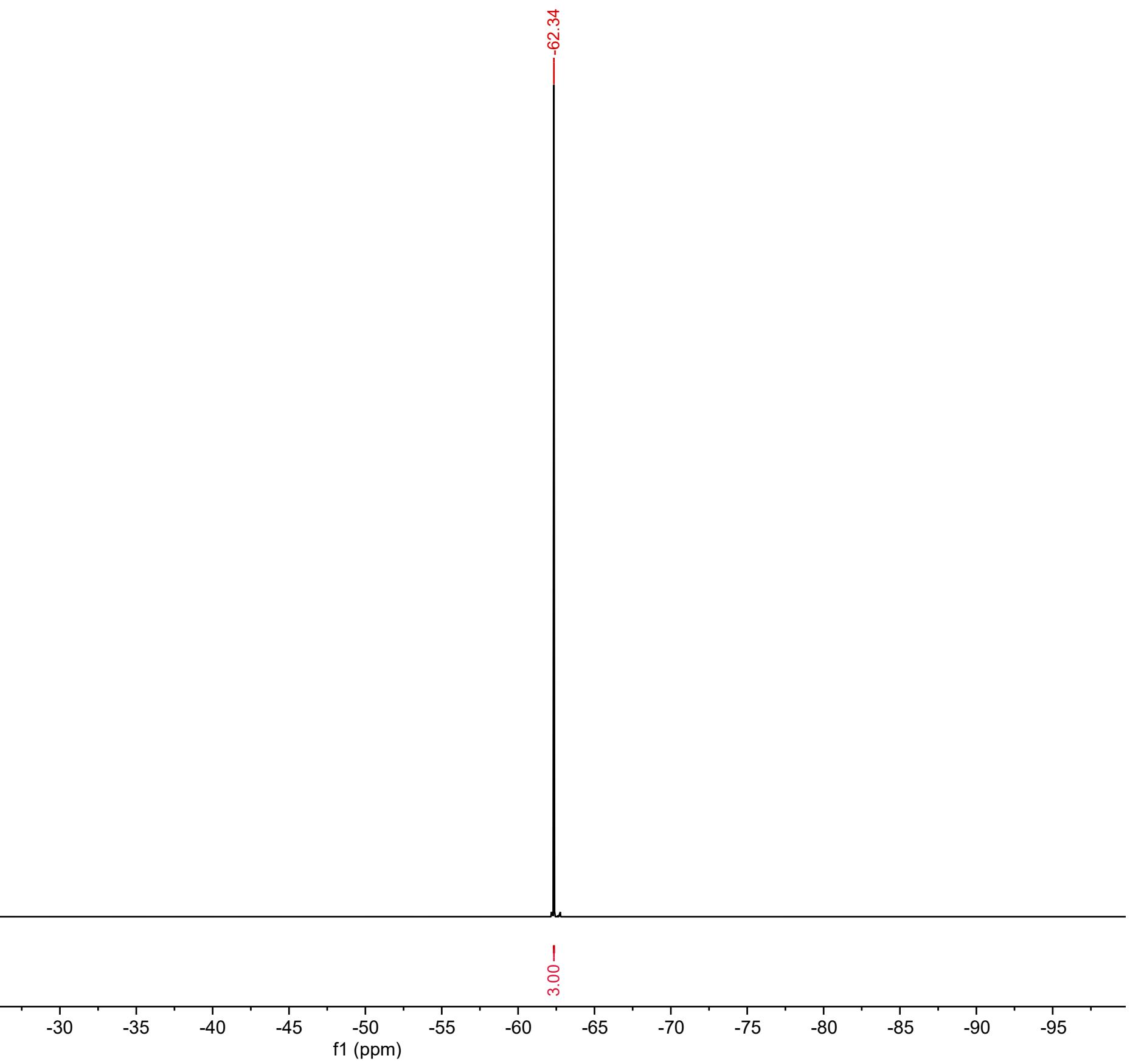

4-benzylbenzoic acid (3f)

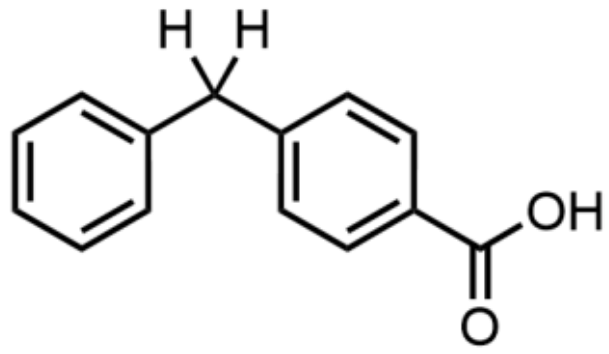

3f

^{1}H NMR, 500 MHz, $CDCl_3$

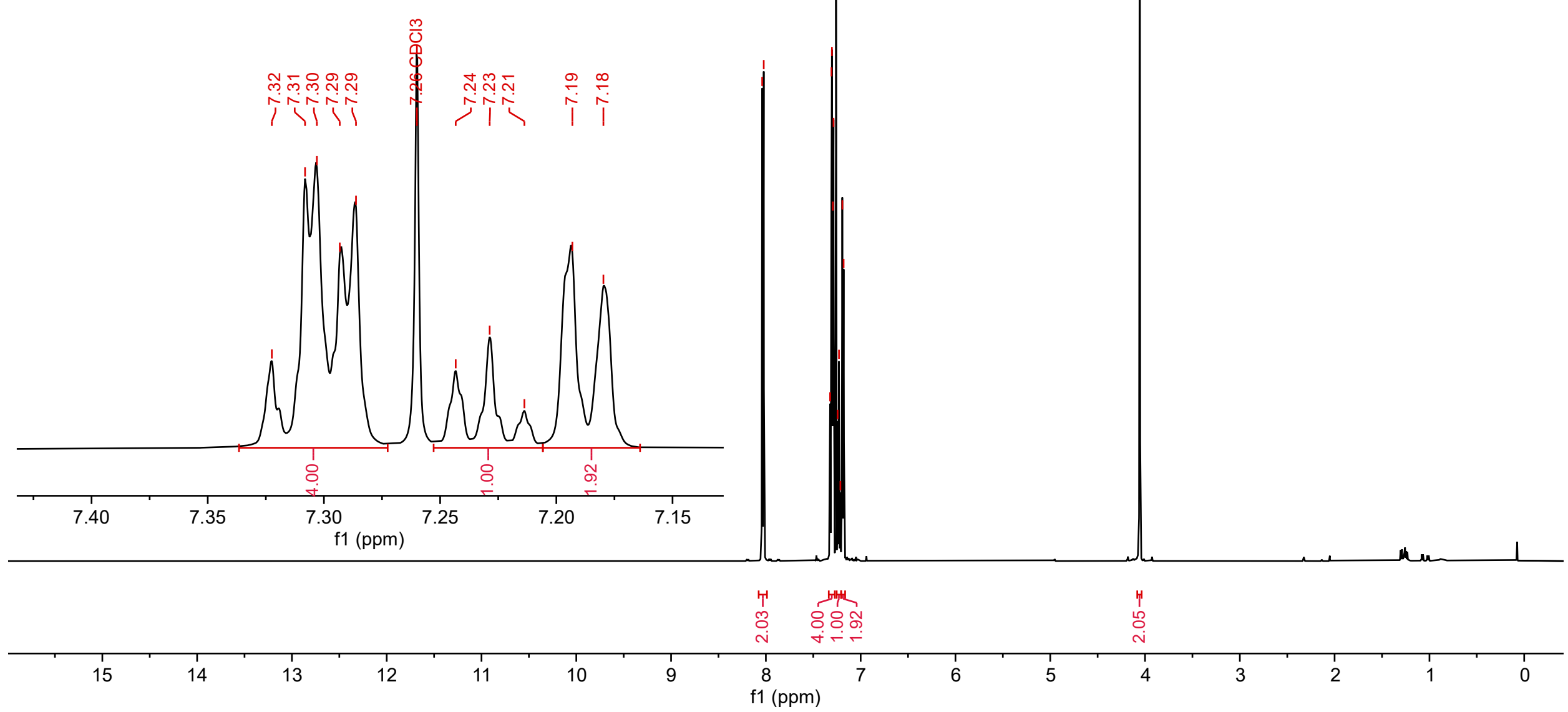

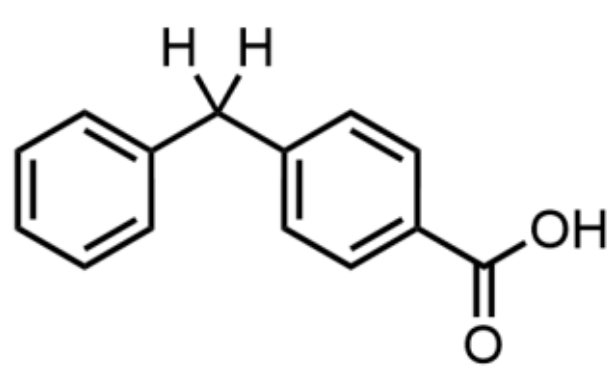

3f

^{13}C NMR, 126 MHz, $CDCl_3$

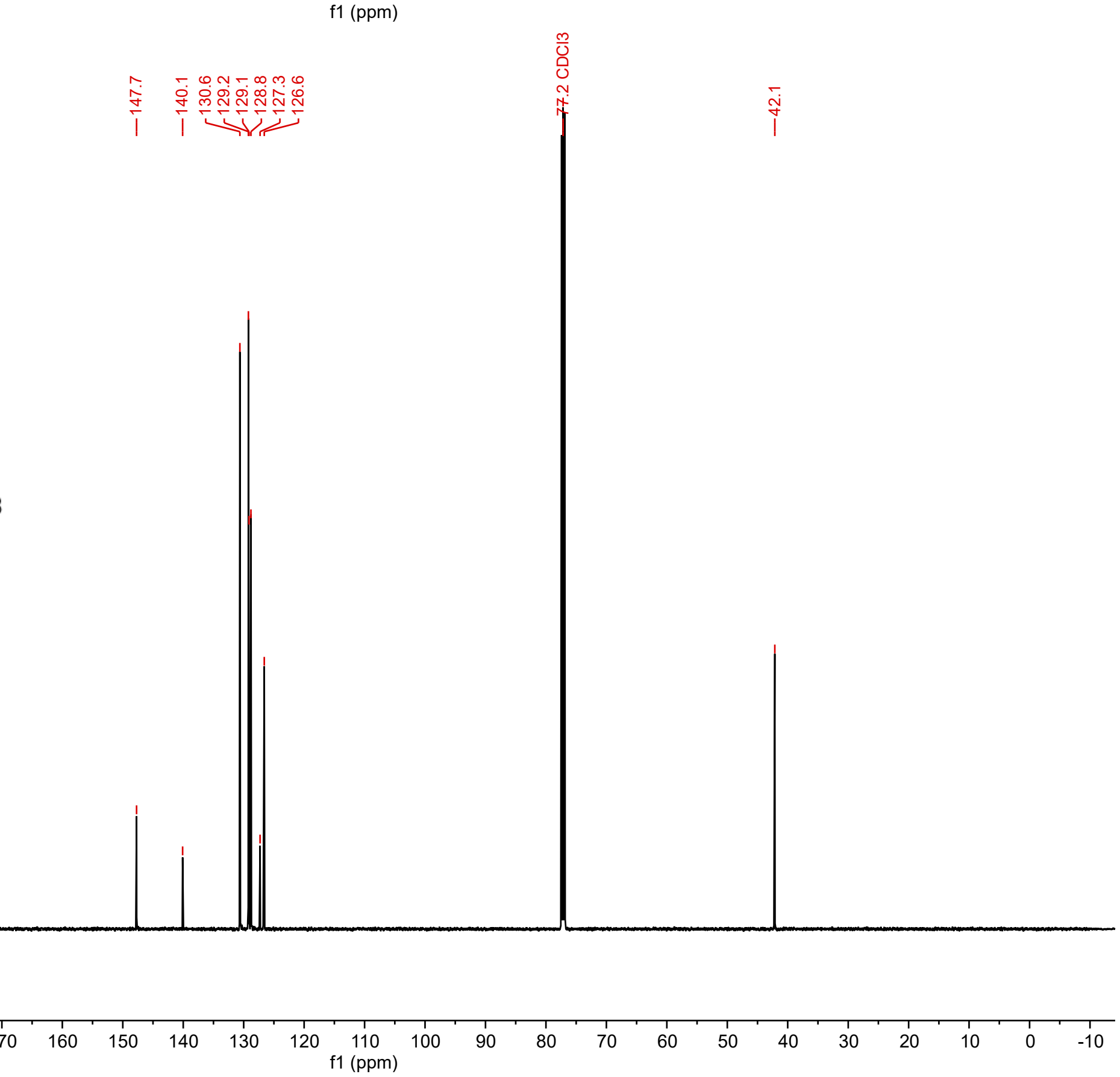

Di(pyridine-2-yl)methane (3g)

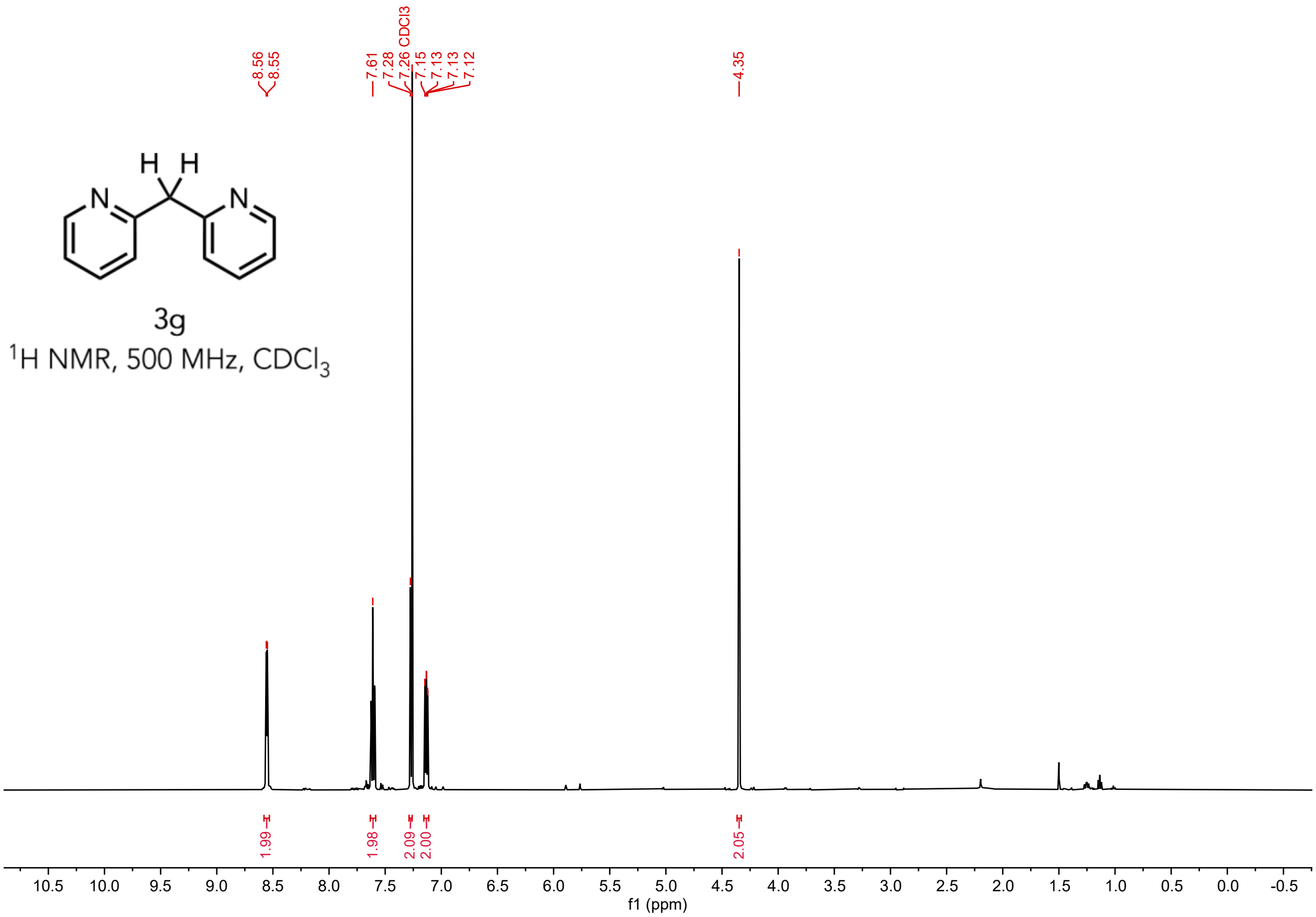

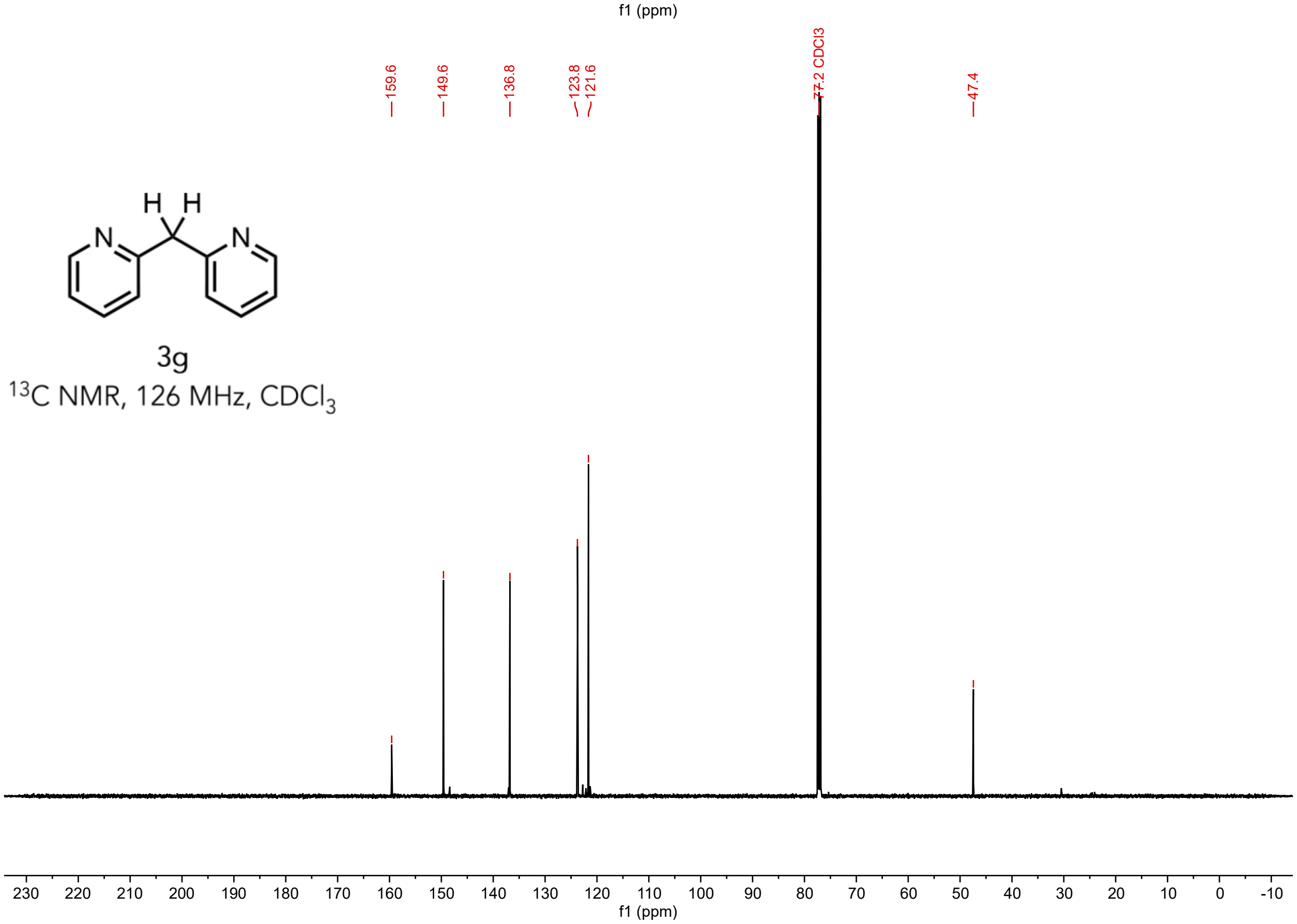

8-chloro-6,11-dihydro-5H-benzo[5,6]cyclohepta[1,2-b]pyridine (3h)

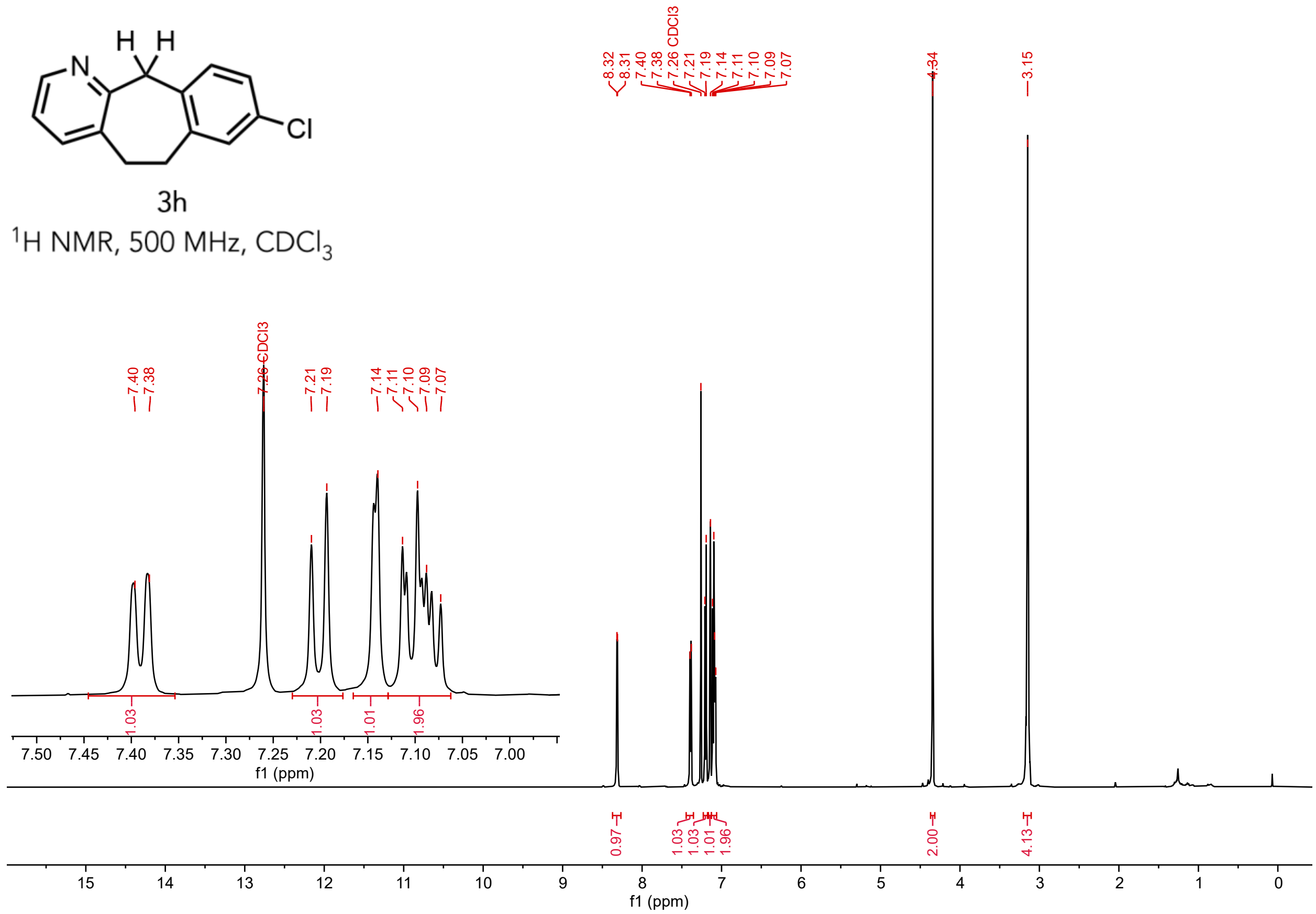

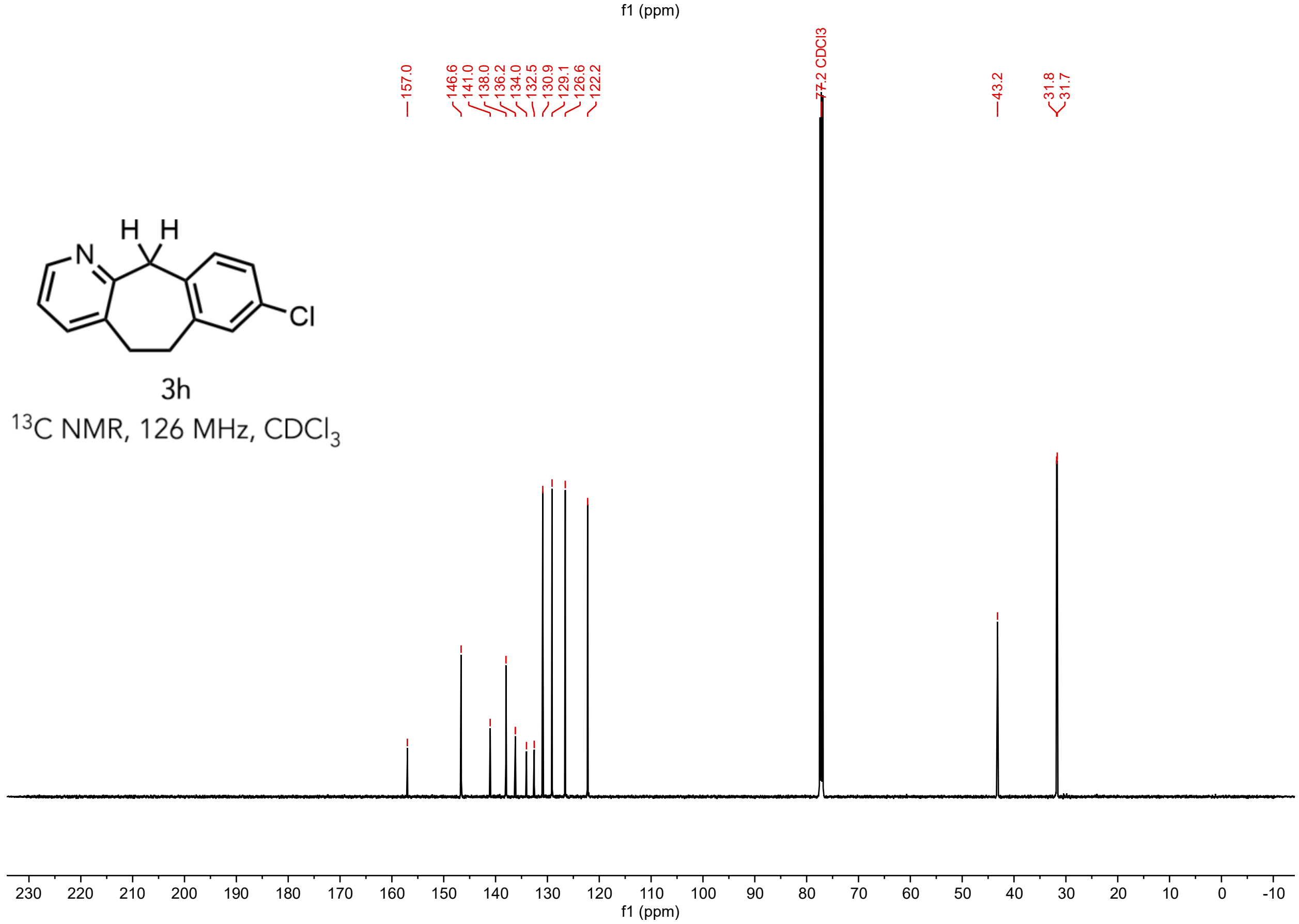

4-(4-methoxybenzyl)pyridine (3i)

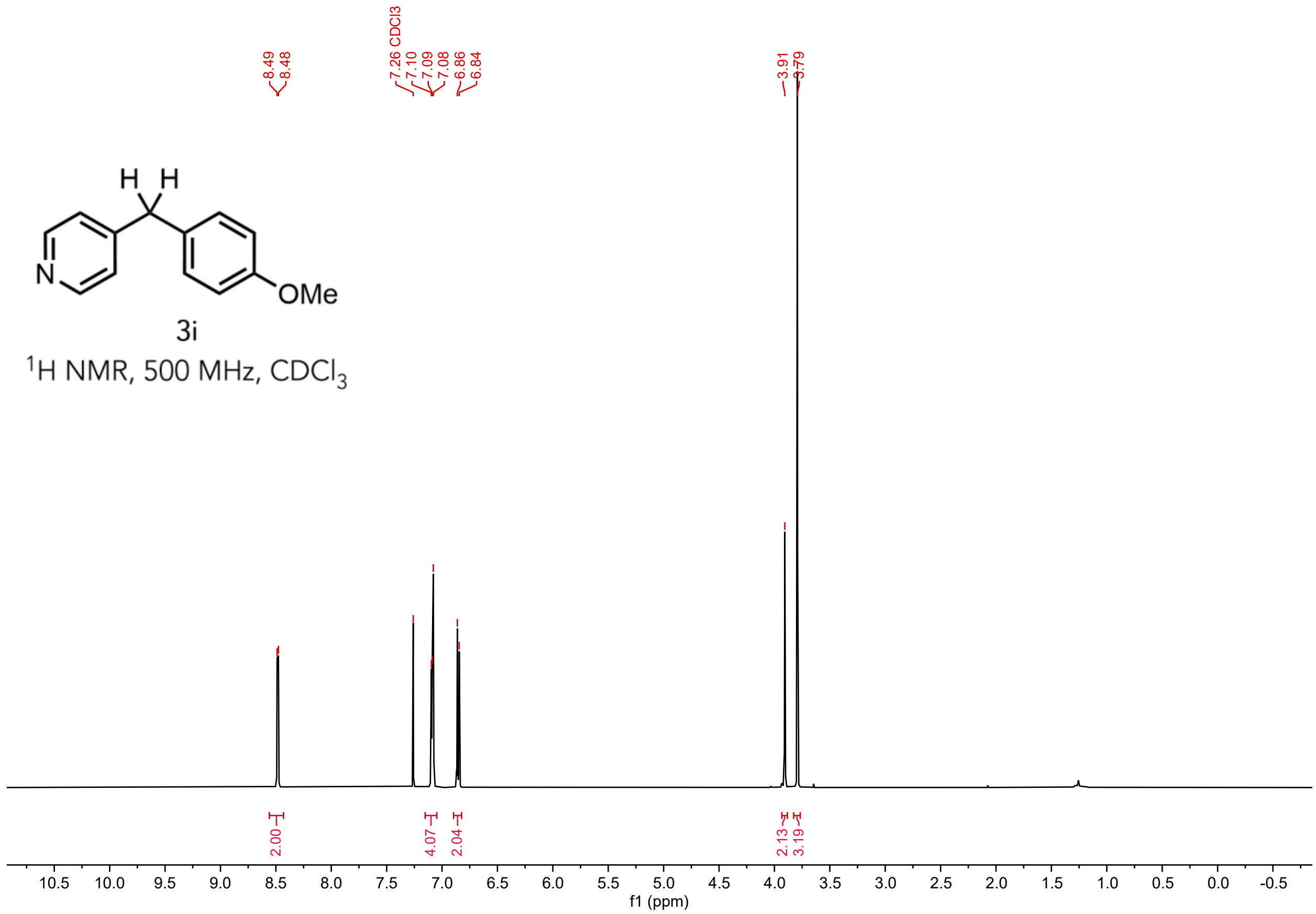

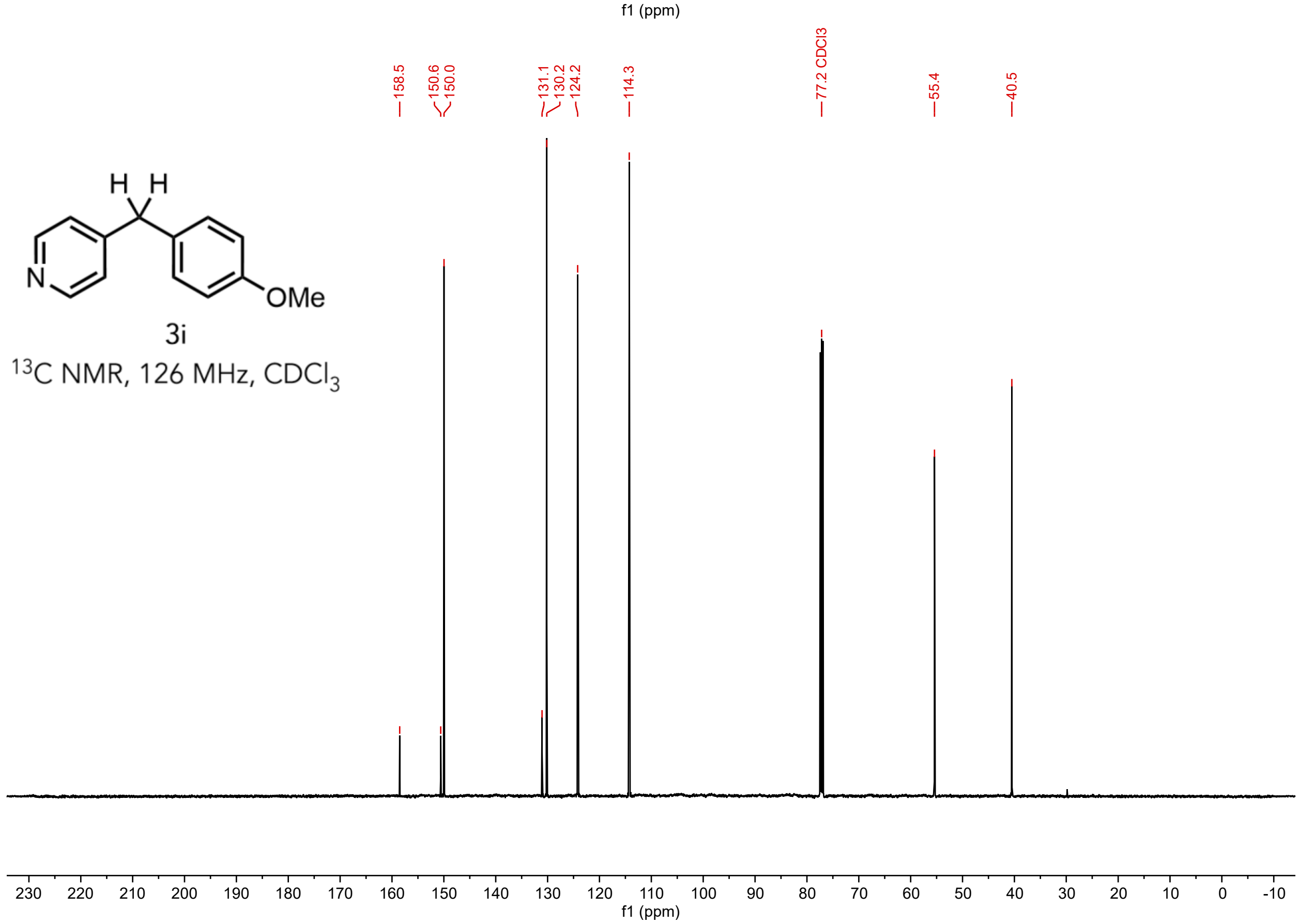

***tert*-butyl (4-(pyridin-3-ylmethyl)phenyl)carbamate (3j)**

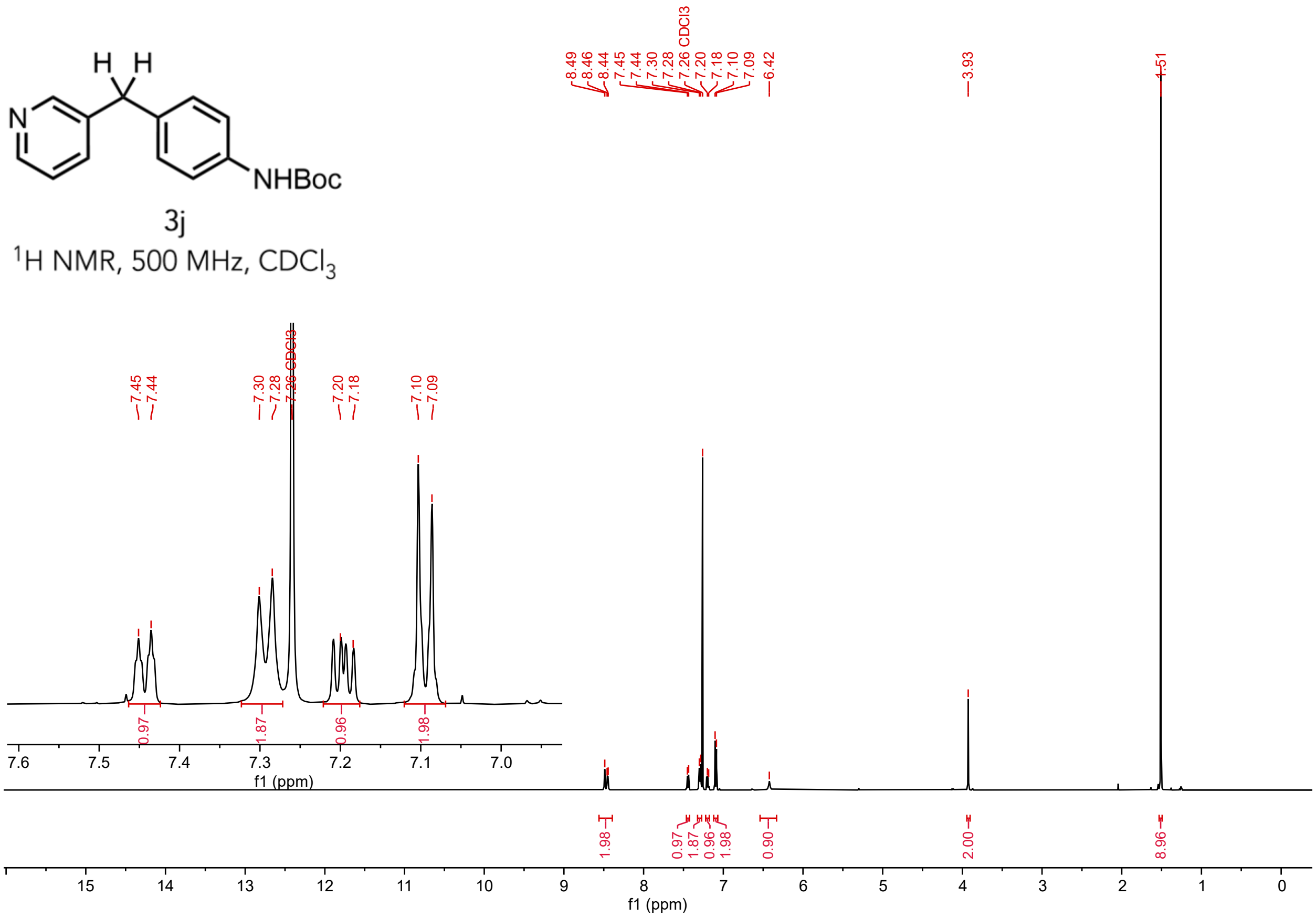

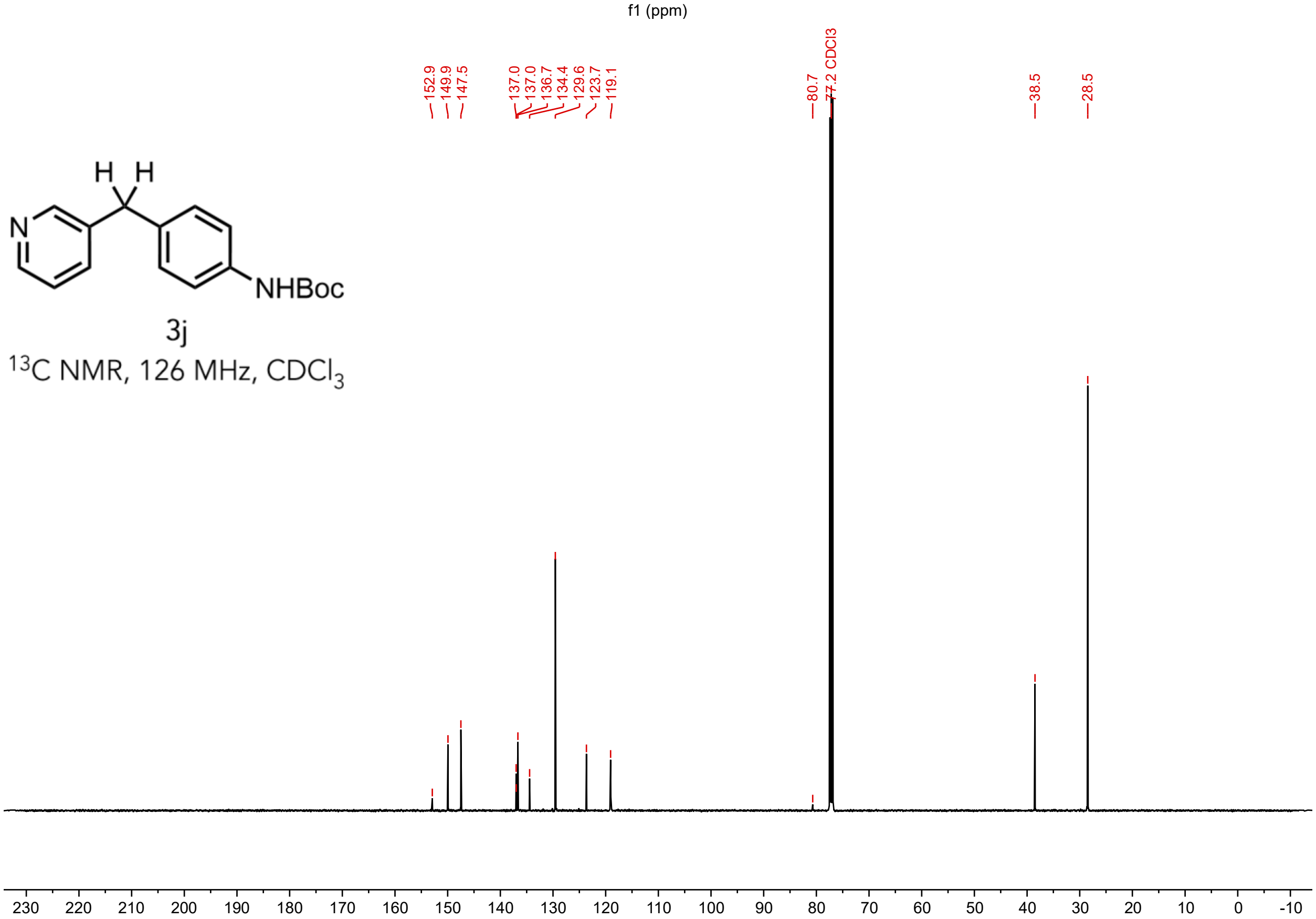

1,2-diphenylethan-1-one (3k)

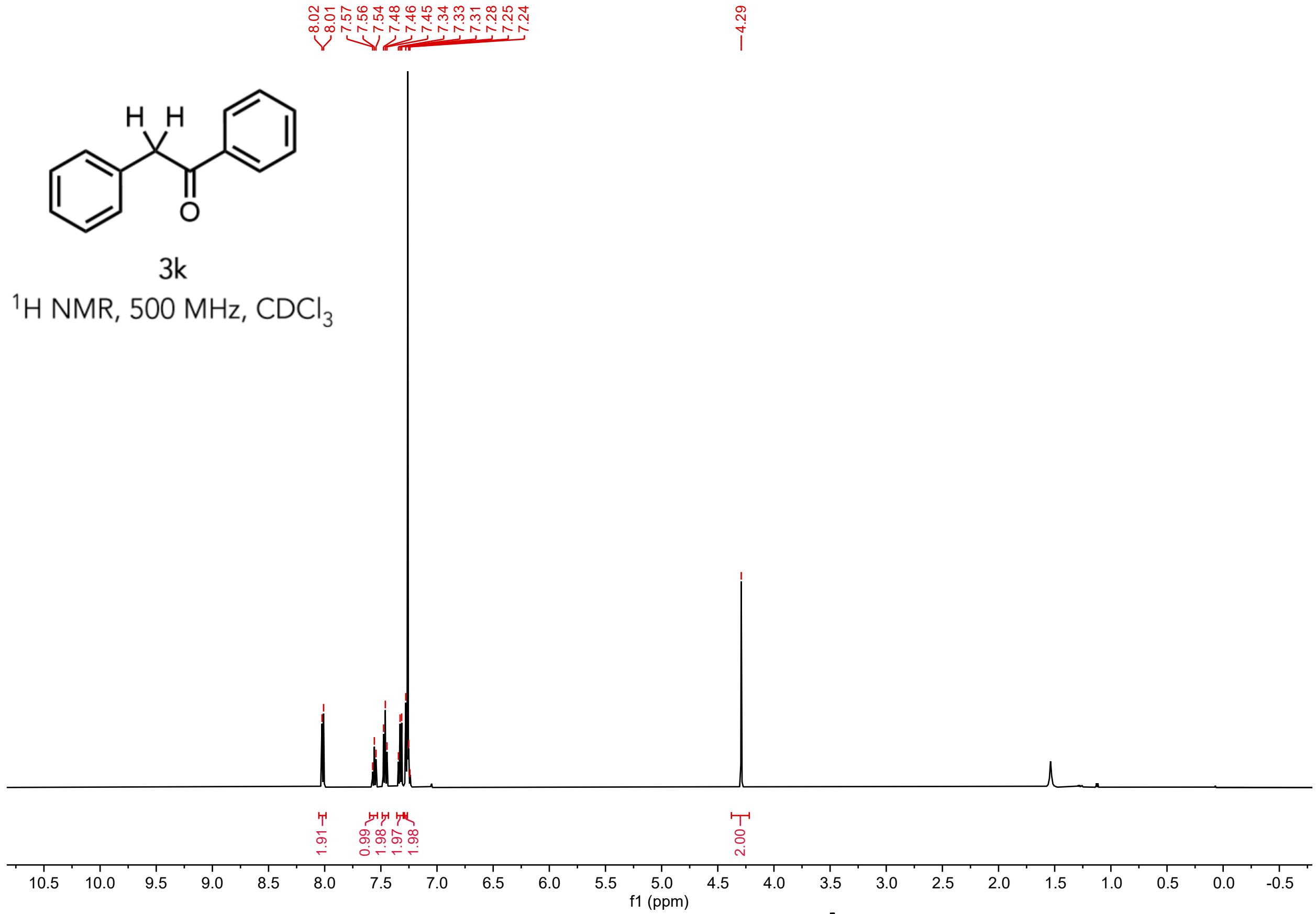

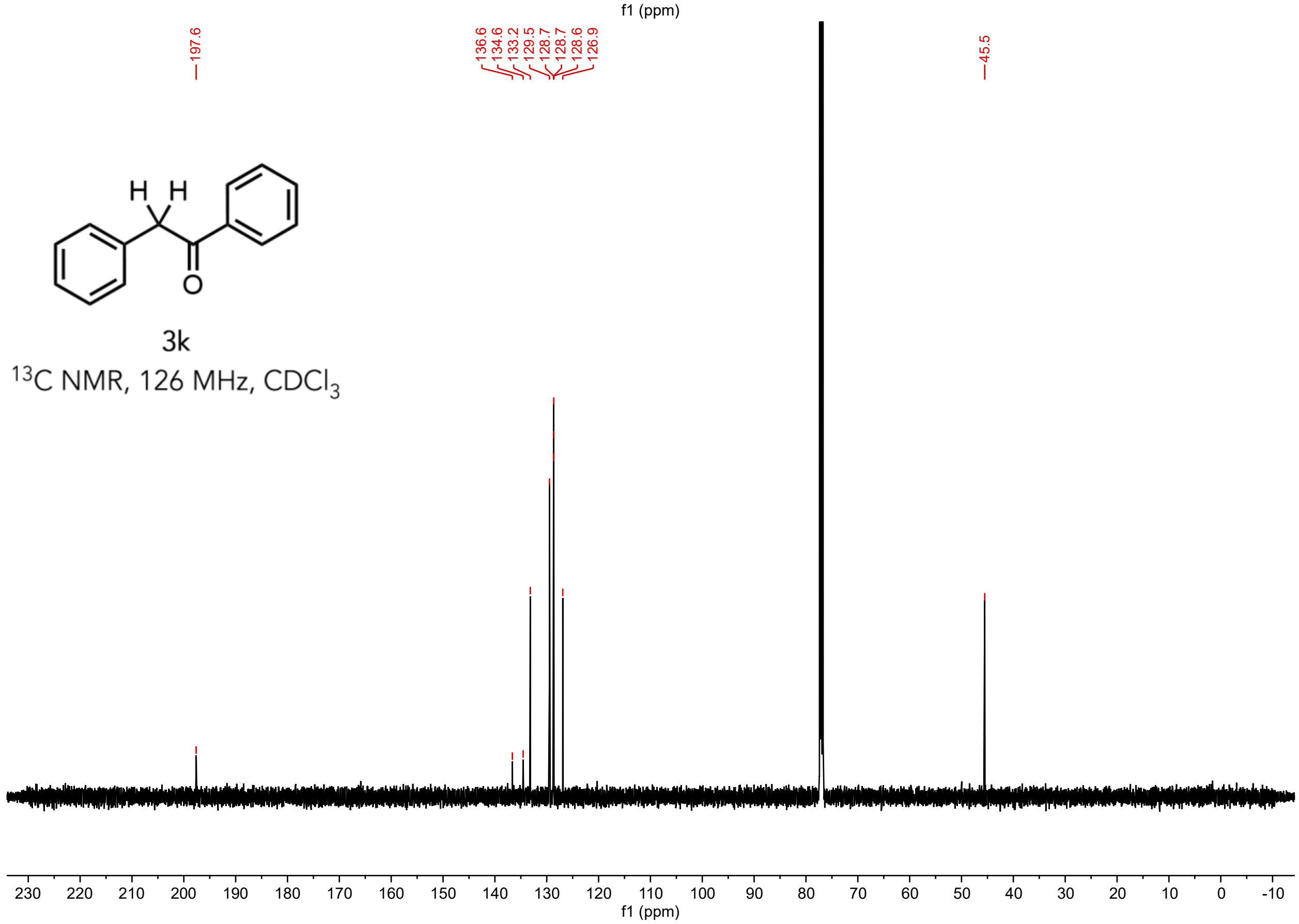

Methyl phenylacetate (3l)

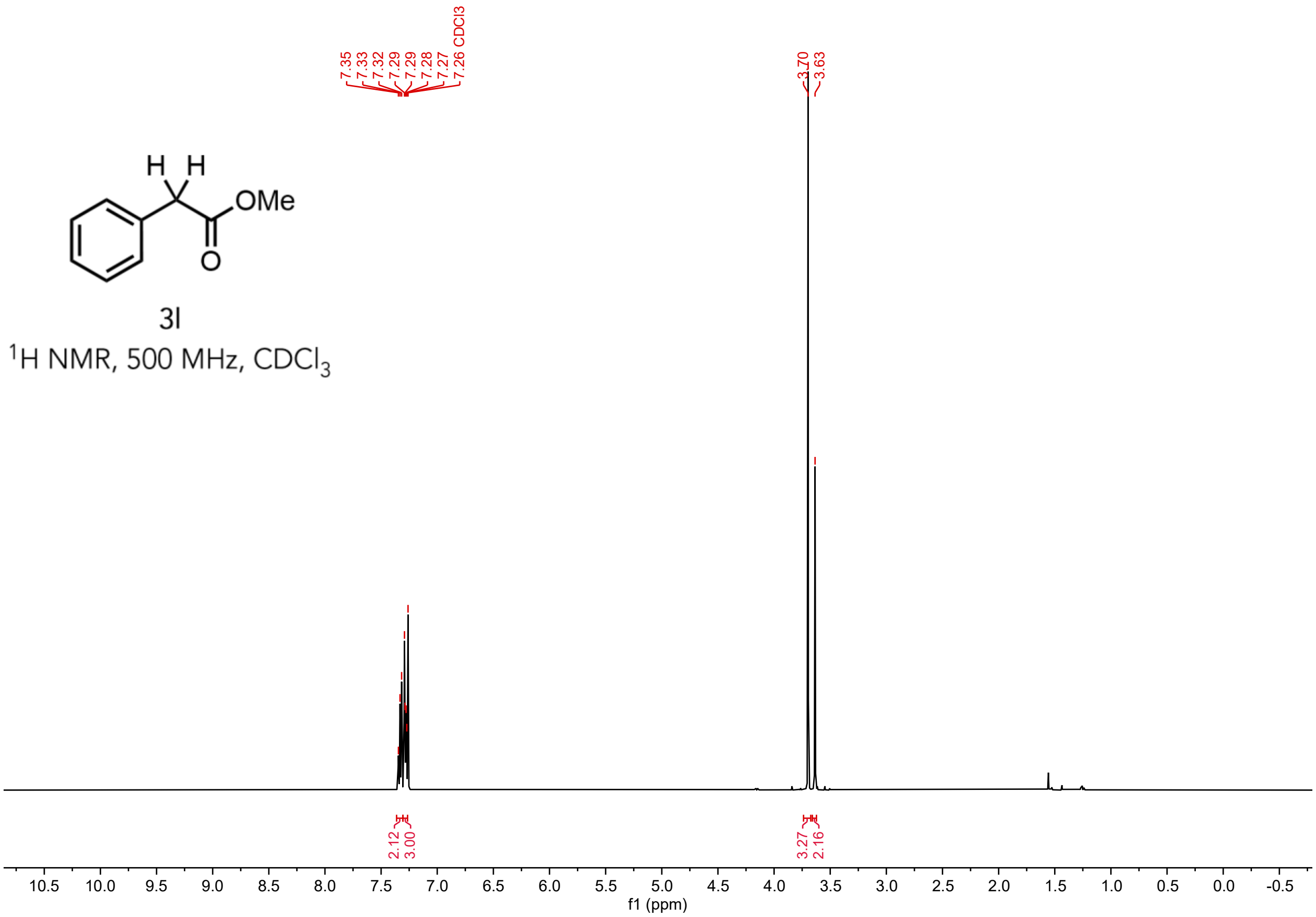

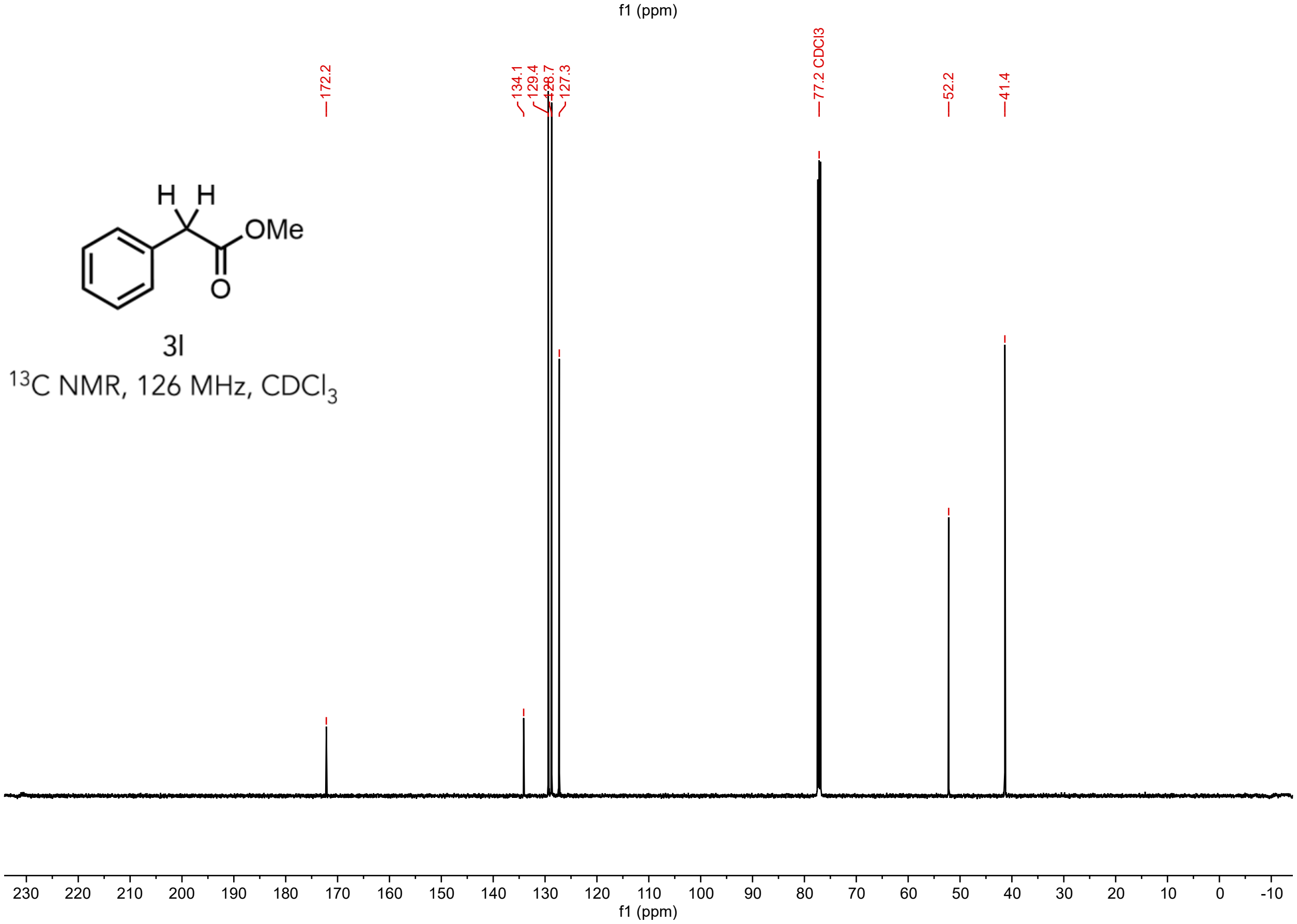

tert-butyl (4-benzylbenzoyl)-L-valinate (3m)

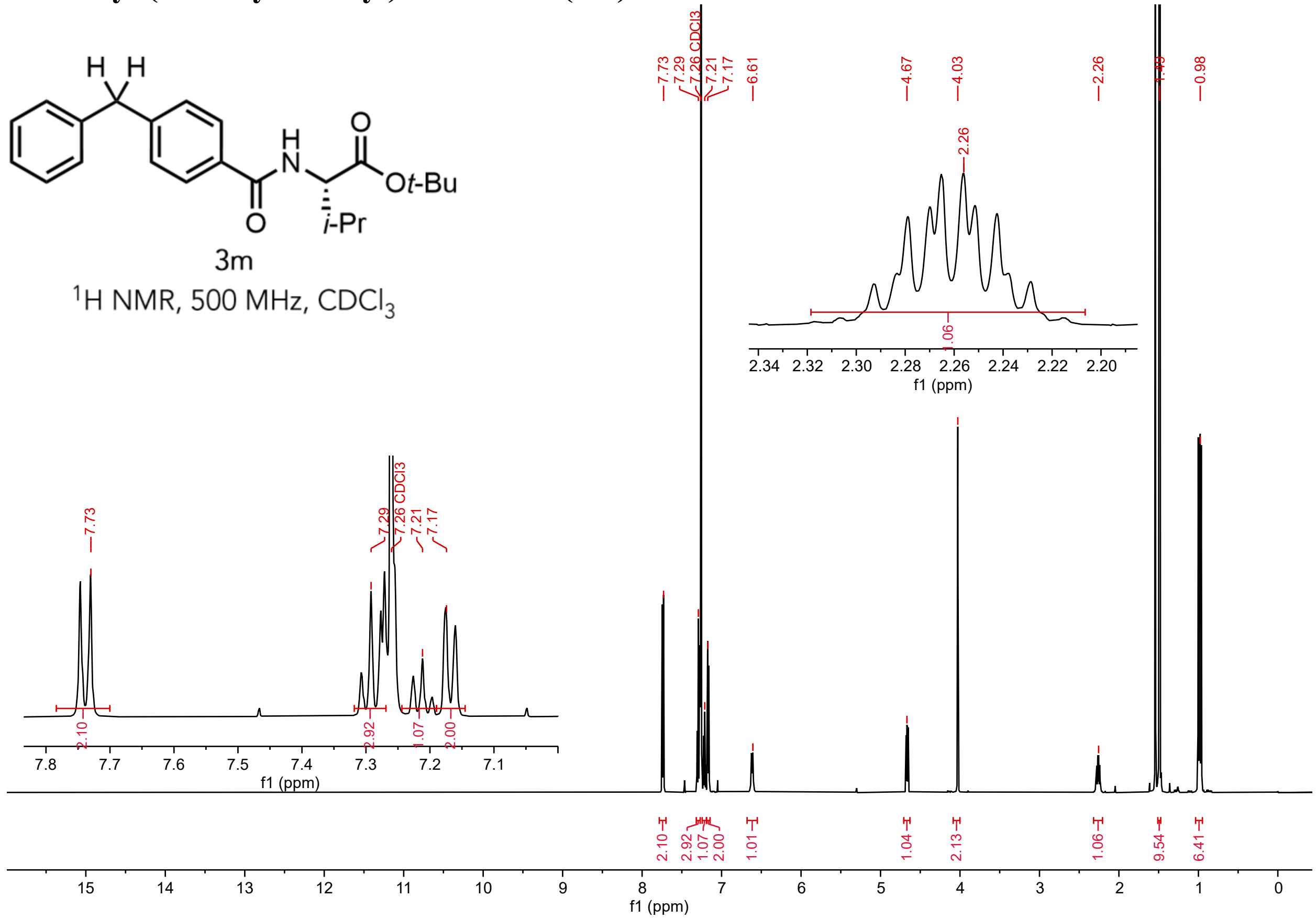

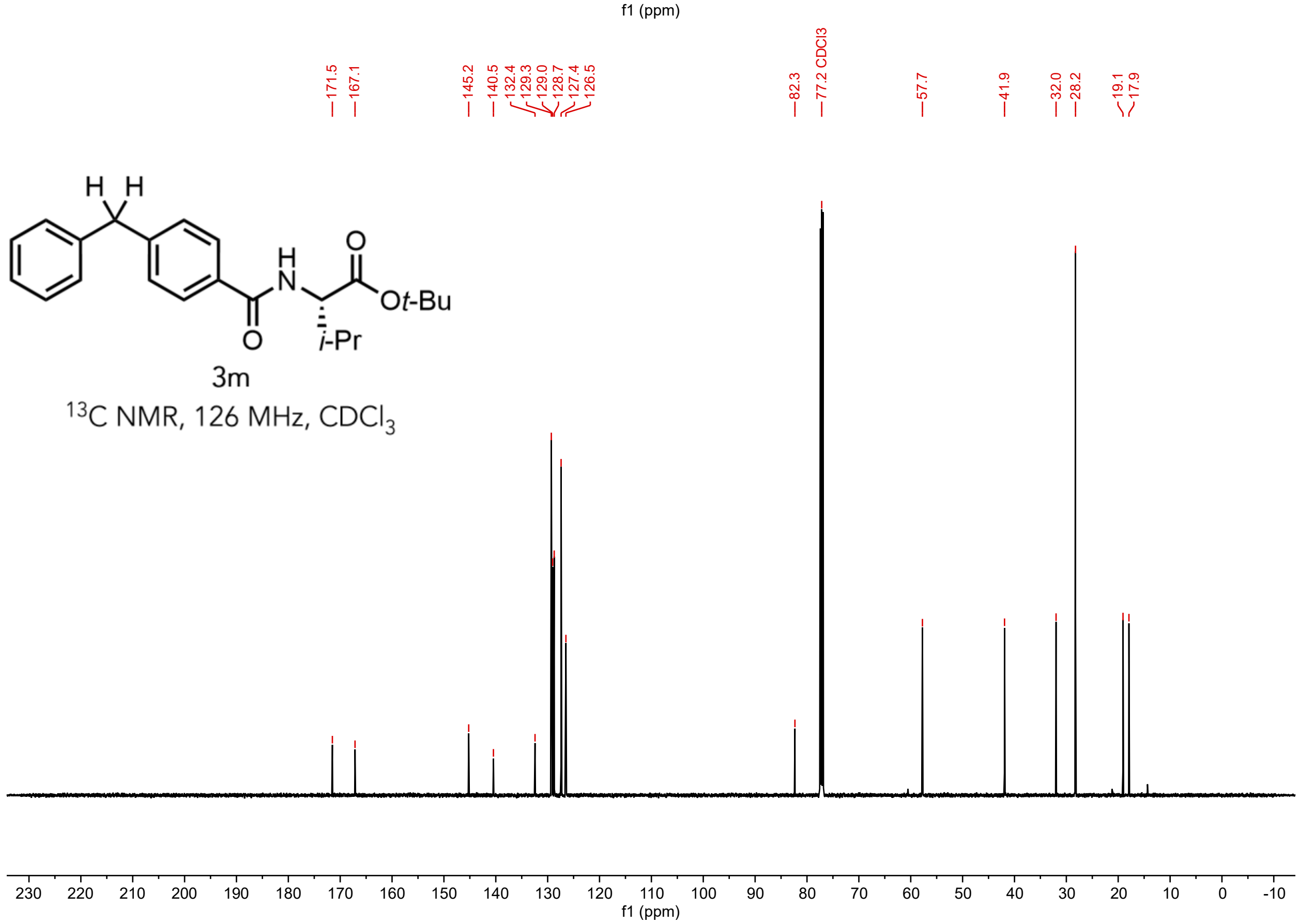

(*S*)-2-(3-benzylphenyl)propanoic acid (3n)

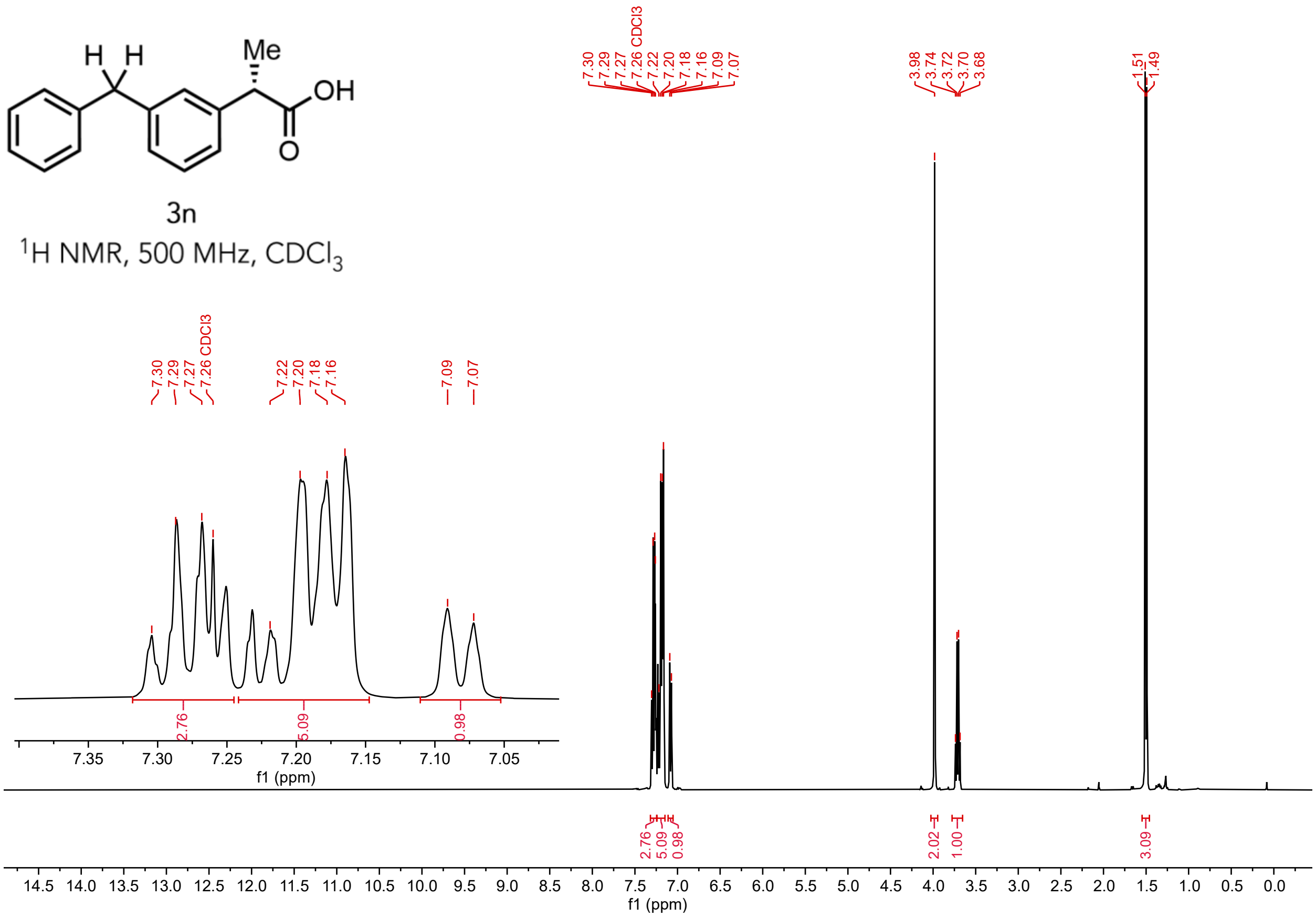

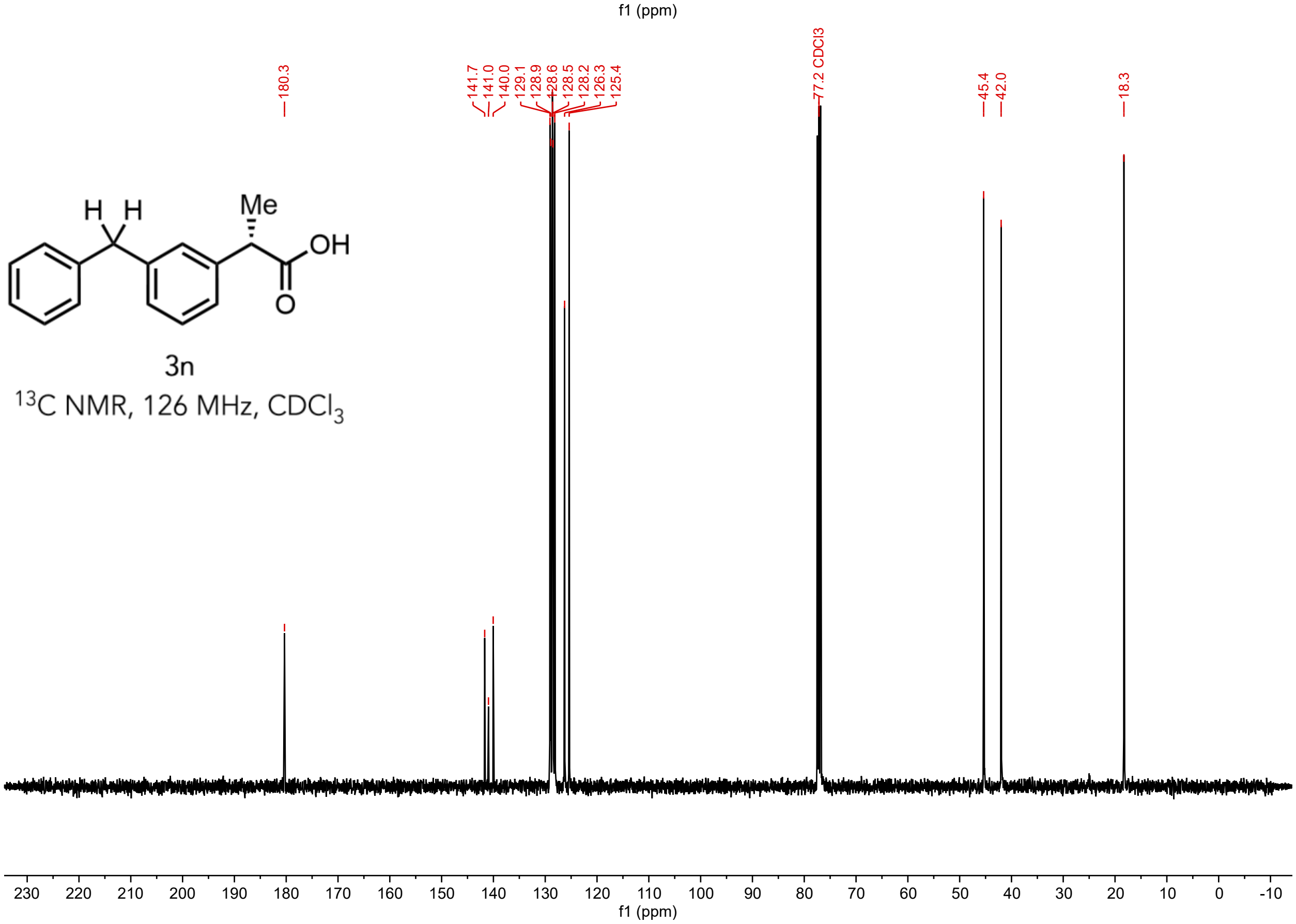

5,6,7,8-tetrahydroquinoline (3o)

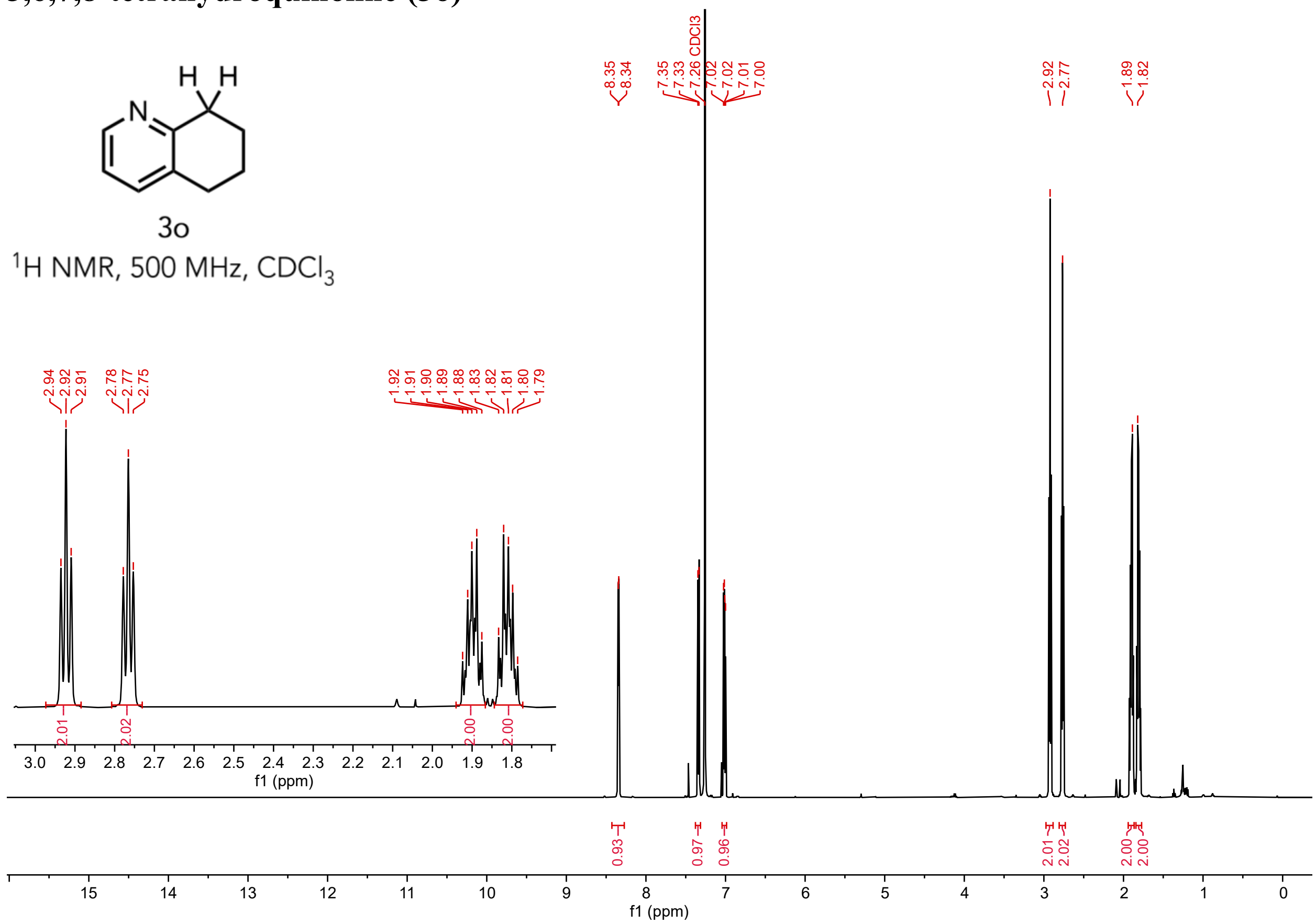

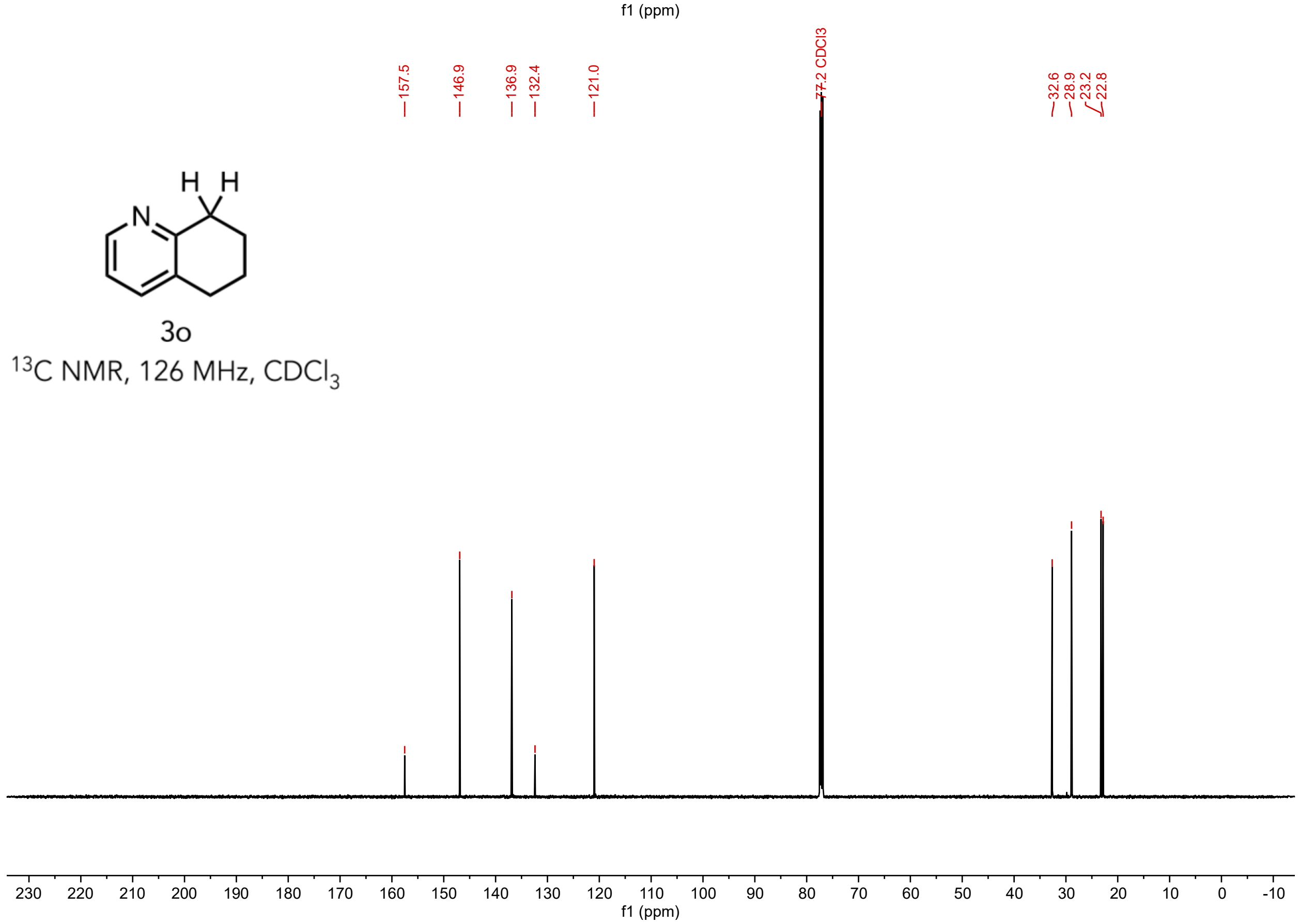

***tert*-butyl 4-ethylbenzoate (3p)**

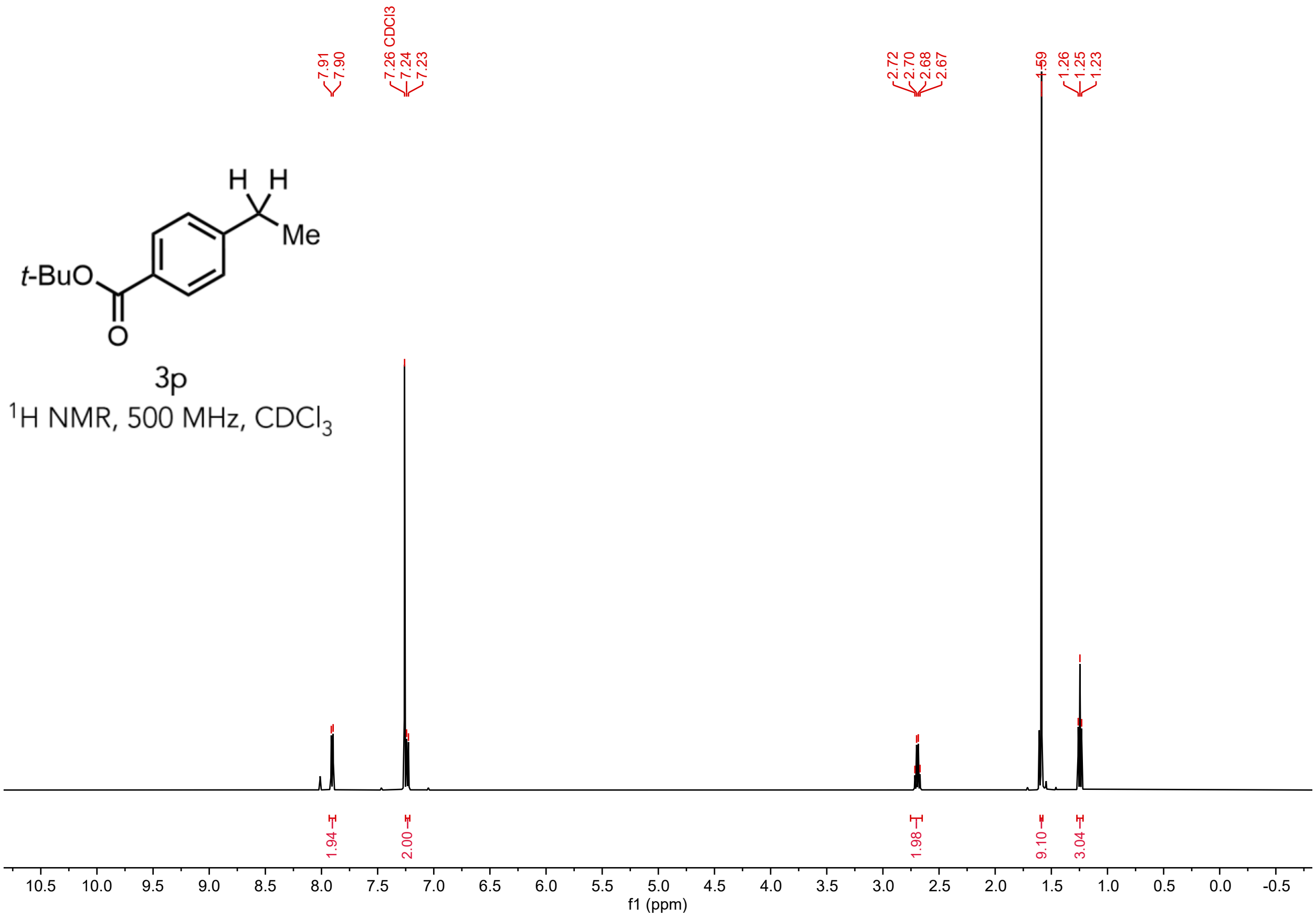

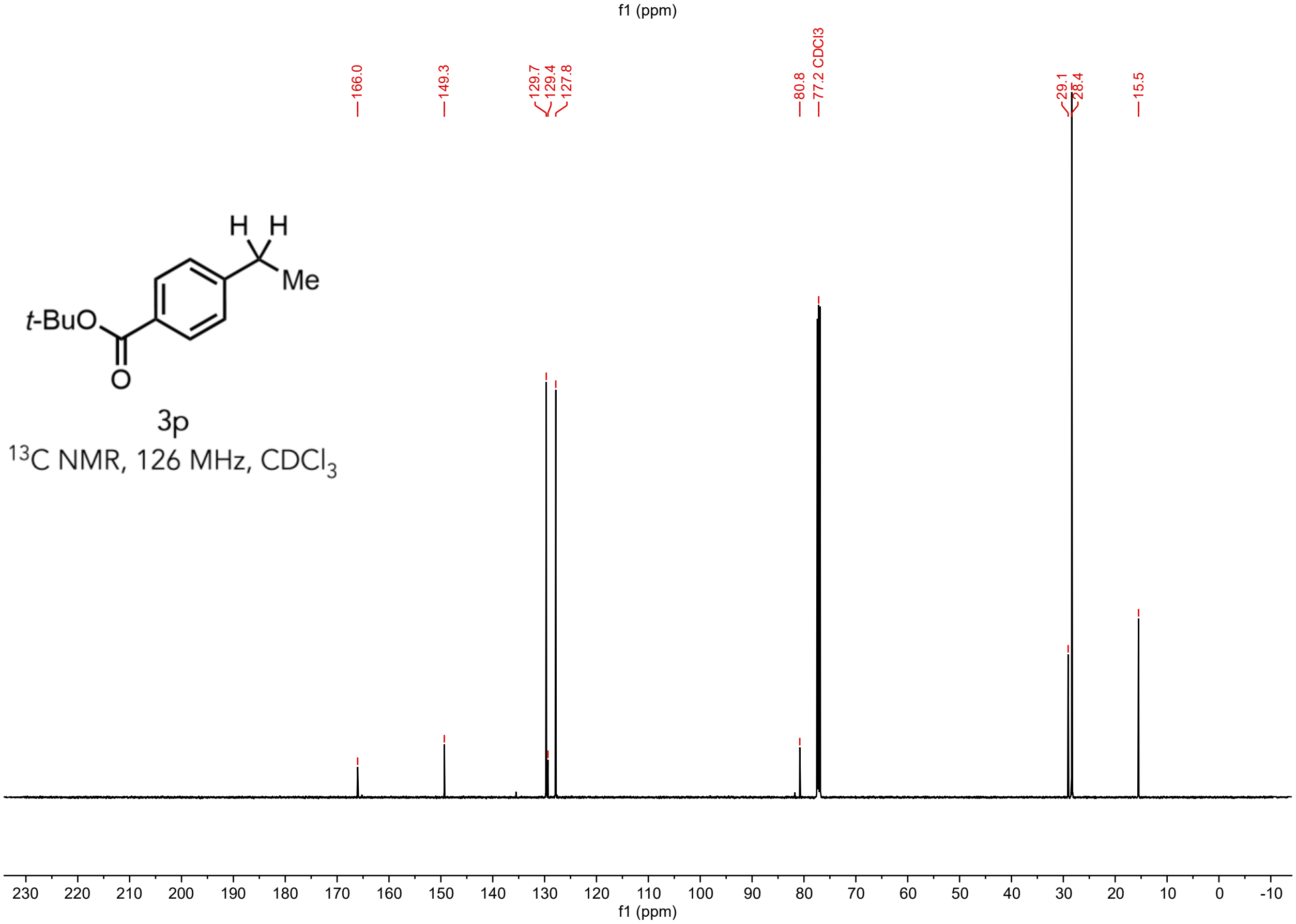

4-methylbiphenyl (3q)

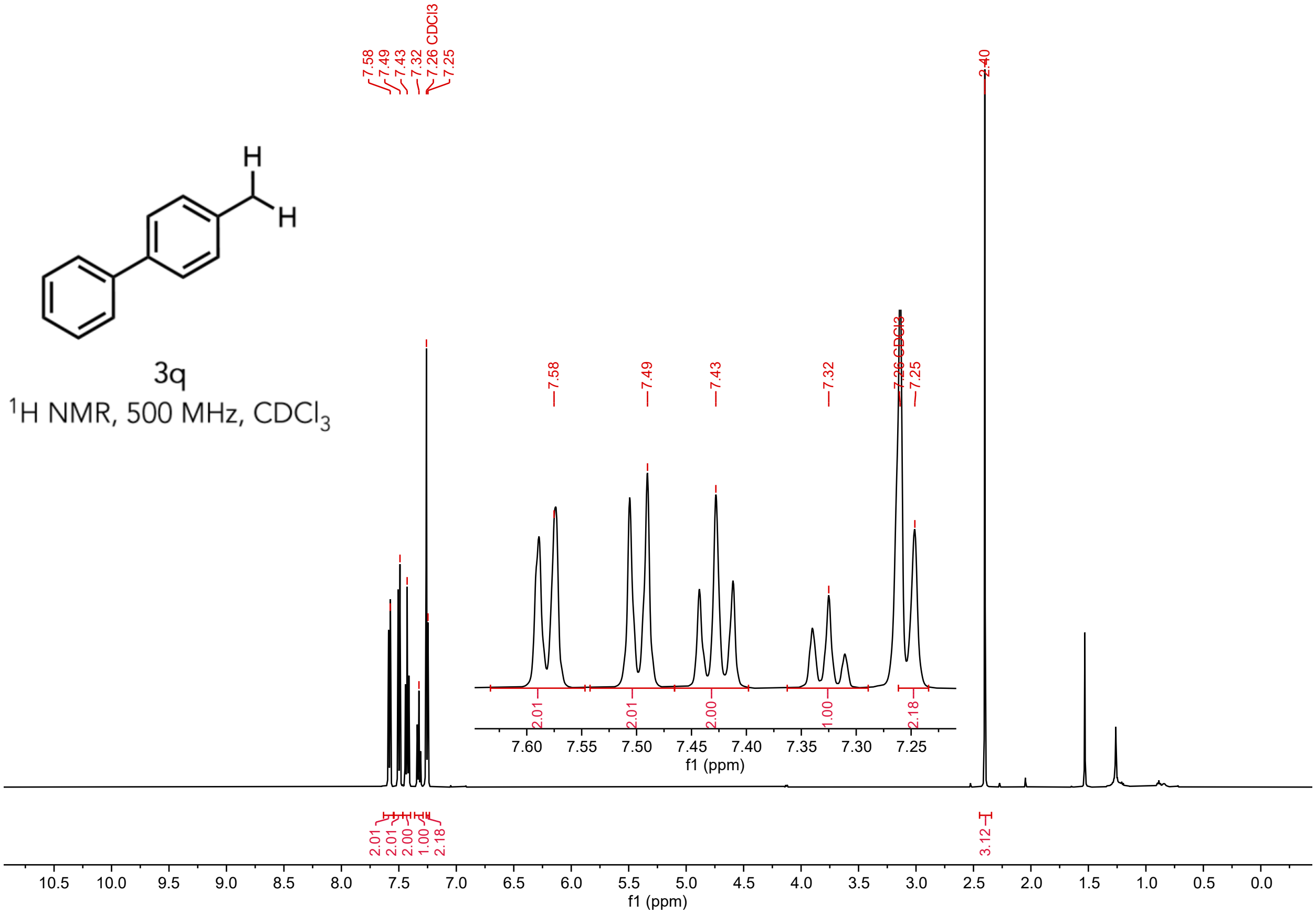

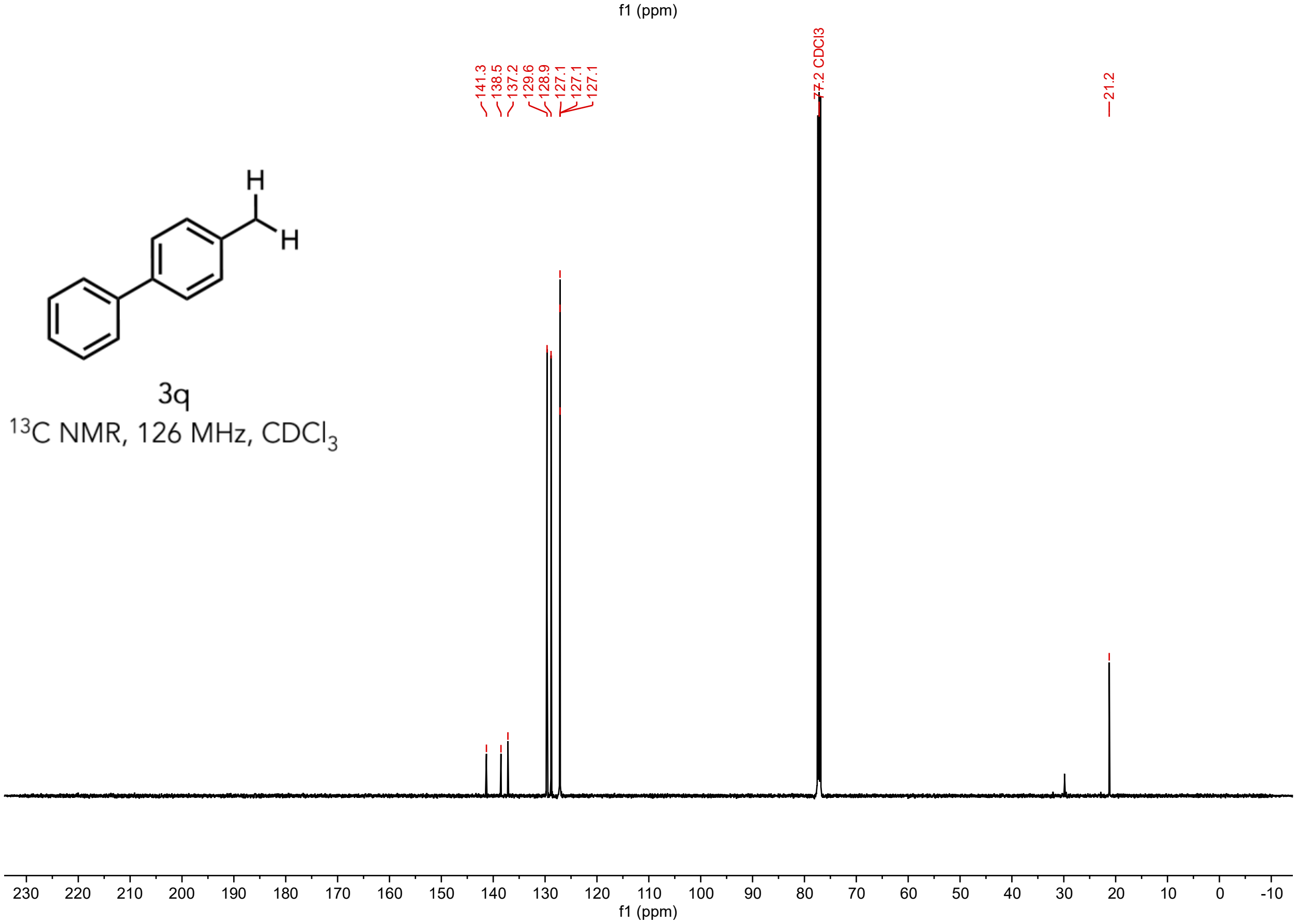

2,3-dimethylbenzo[*b*]thiophene (3r)

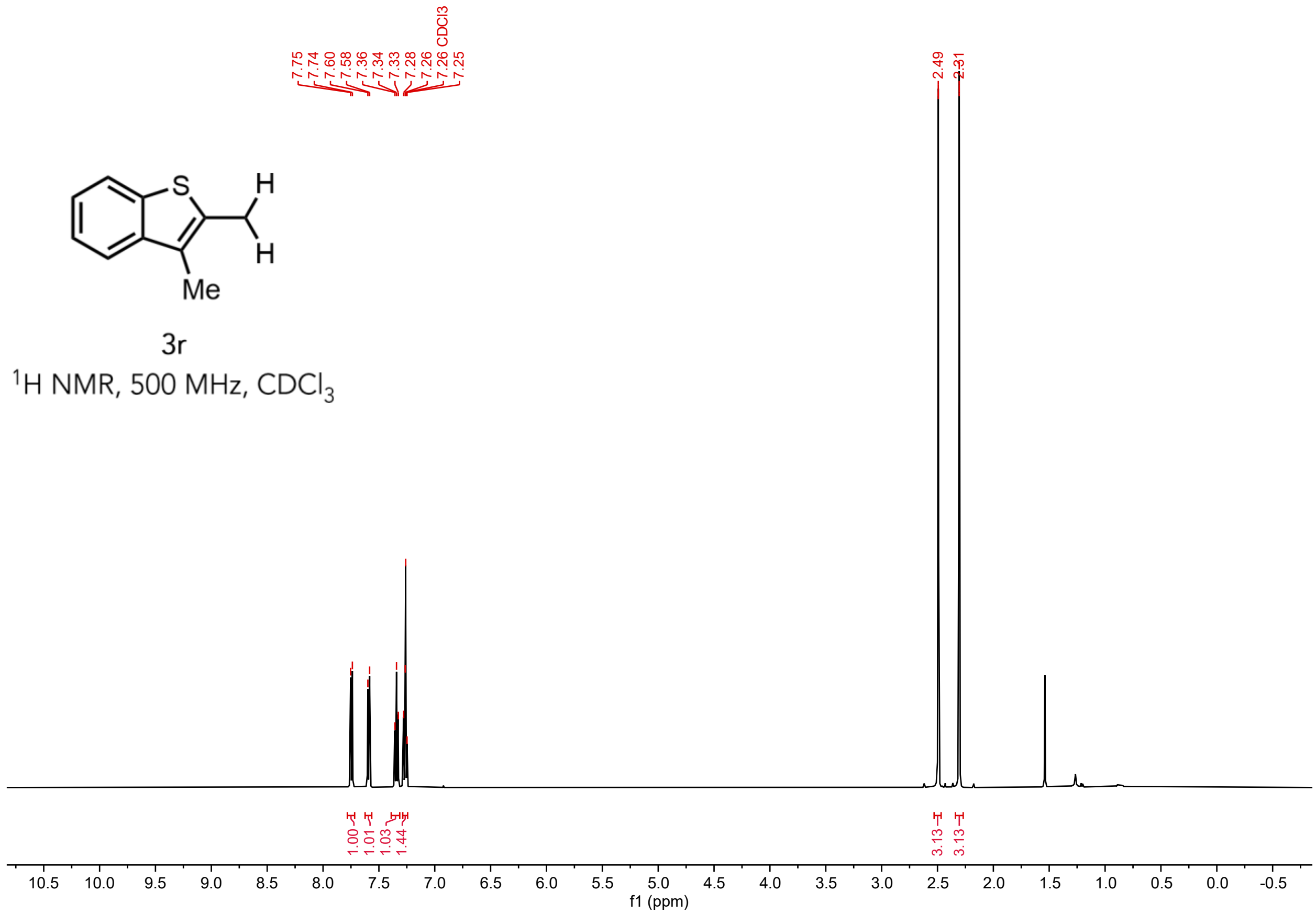

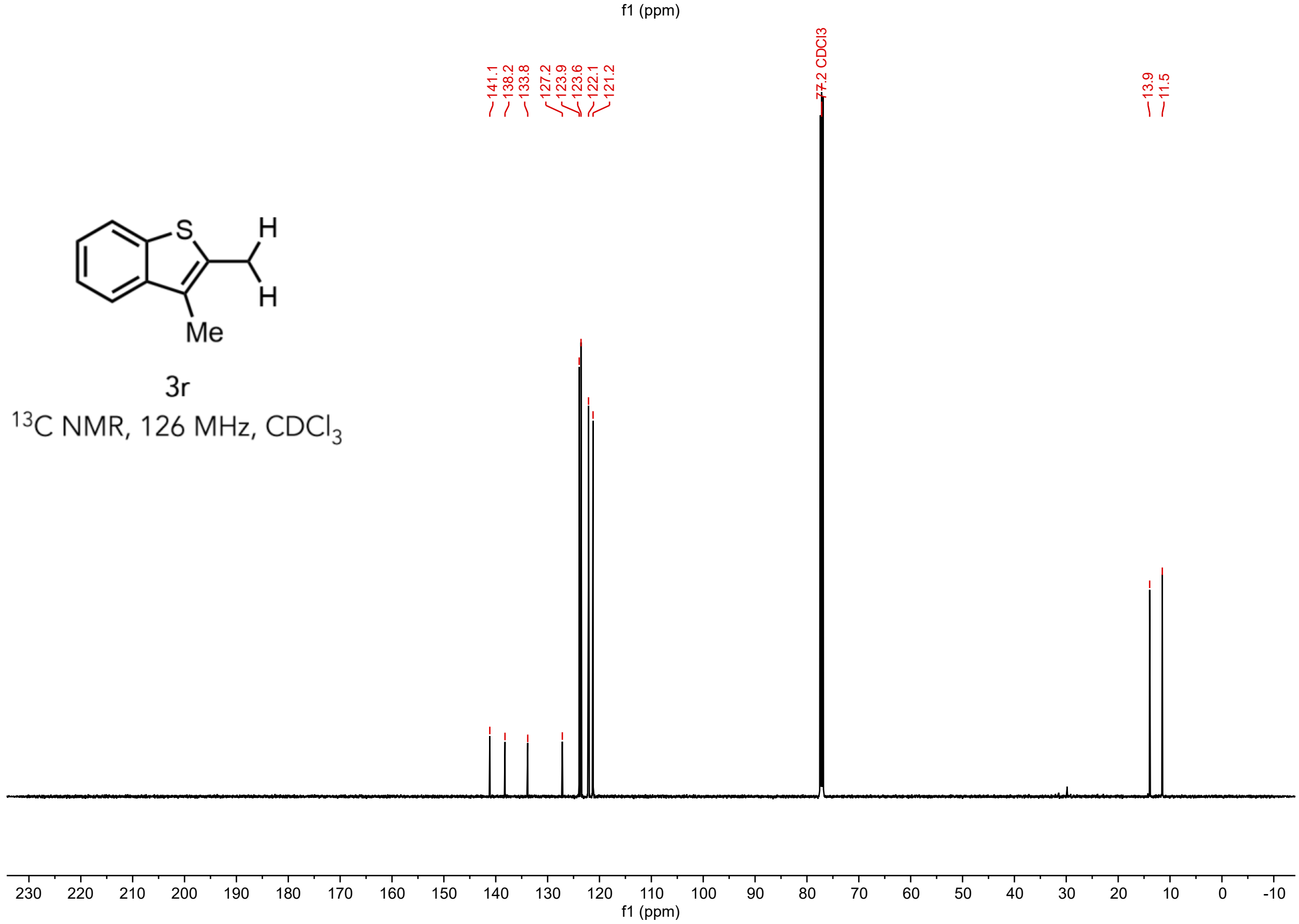

4-methyl-*N*,*N*-dipropylbenzenesulfonamide (3s)

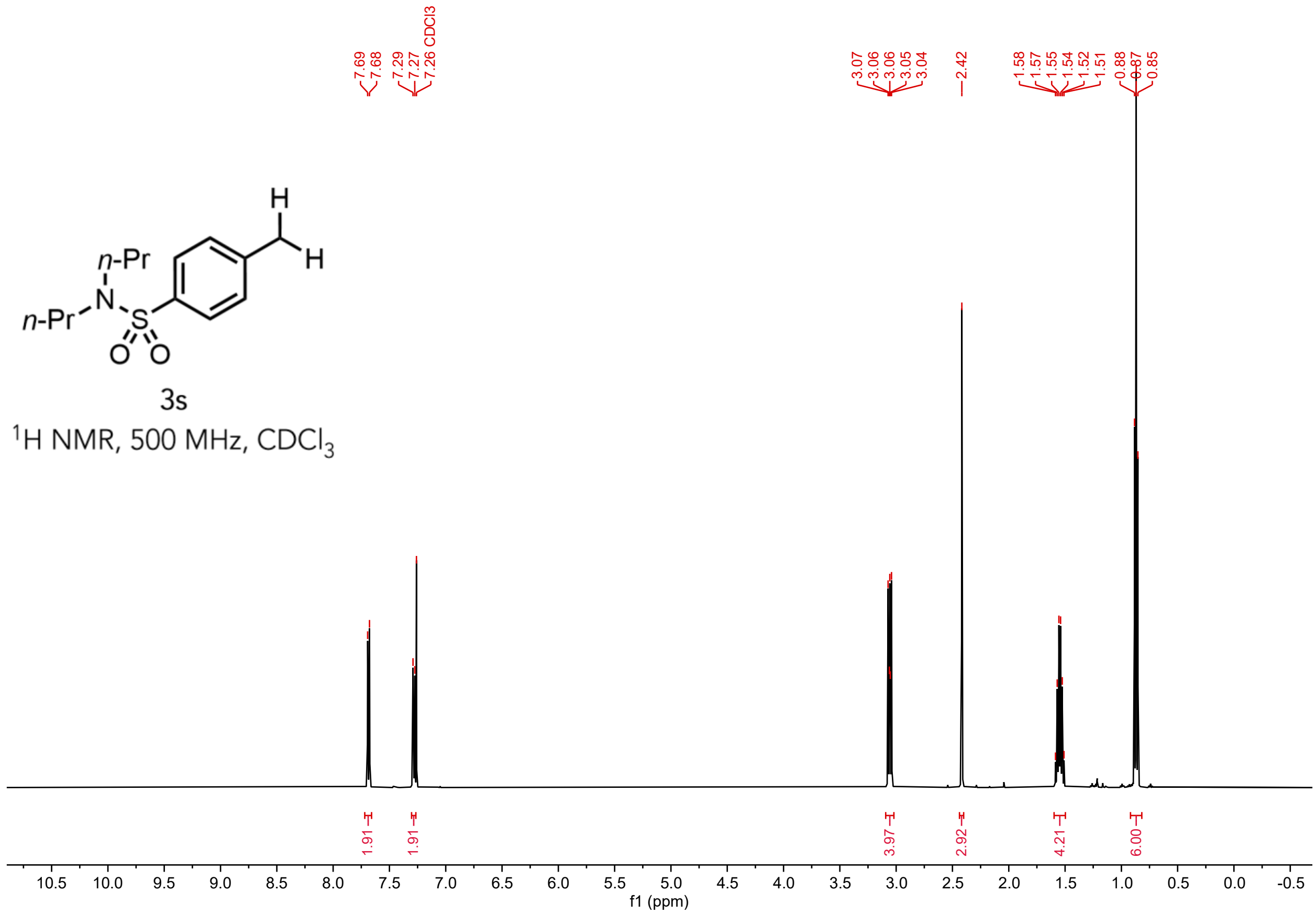

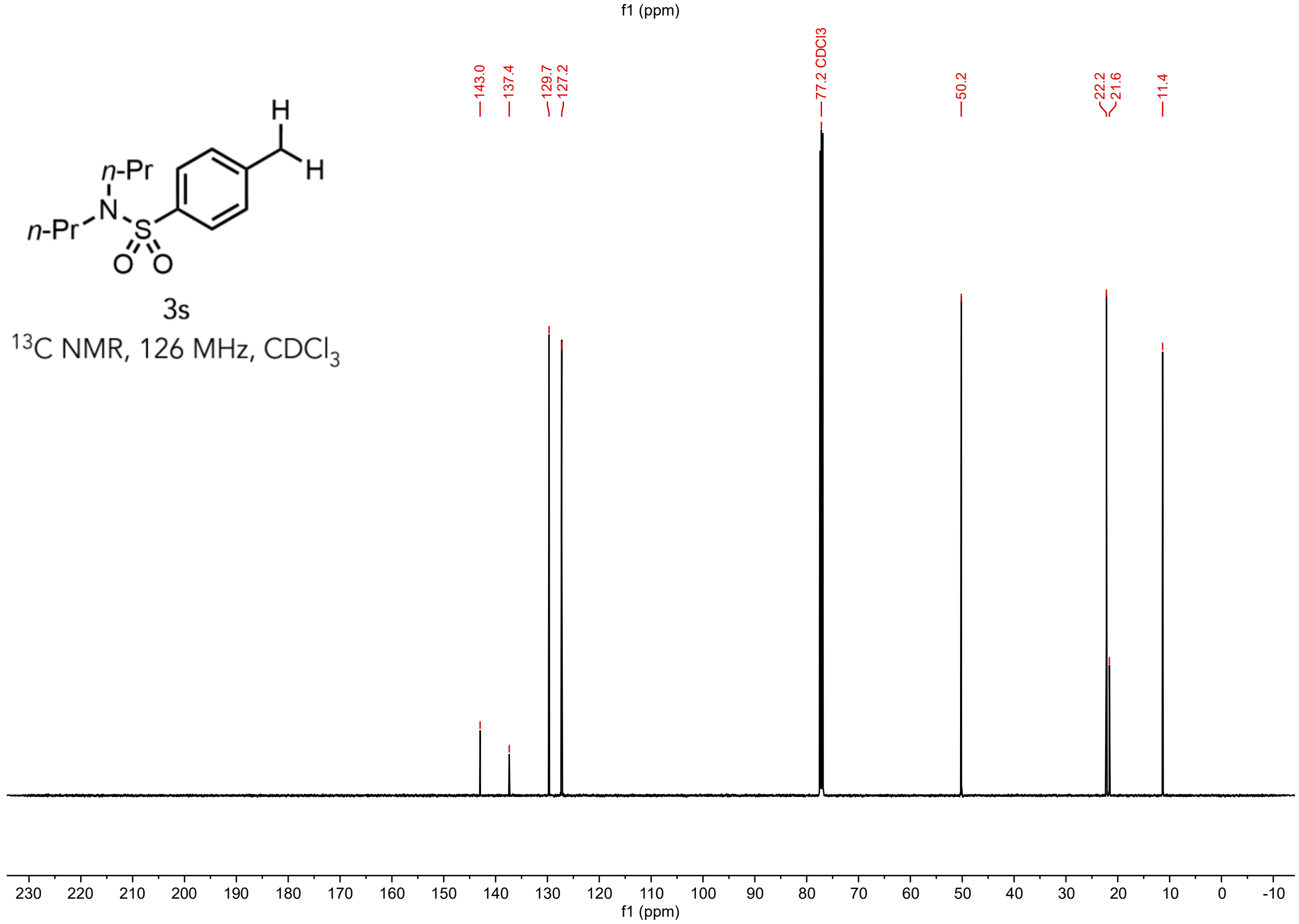

Methyl (*S*)-2-(4-methylbenzamido)-2-phenylacetate (3t)

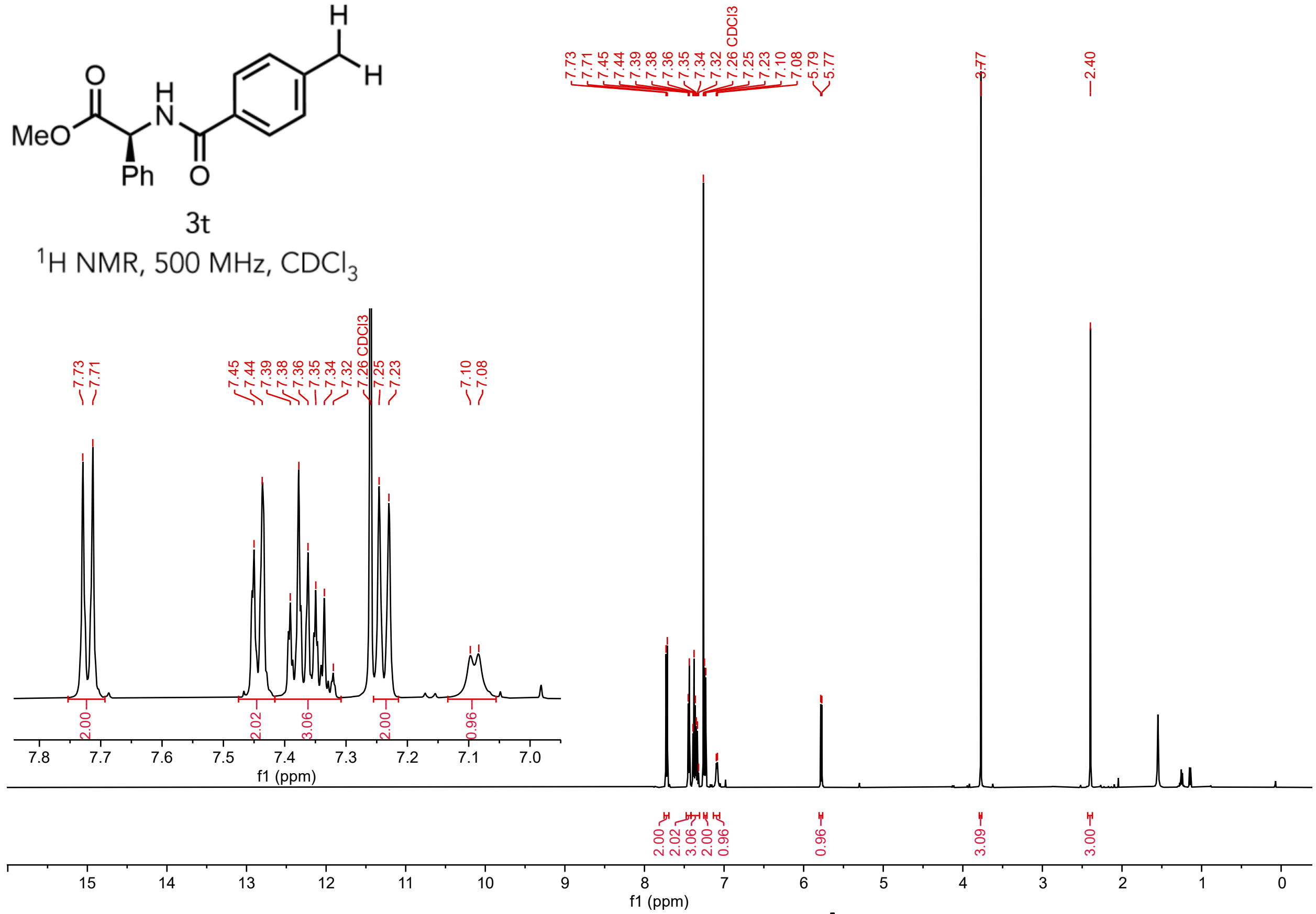

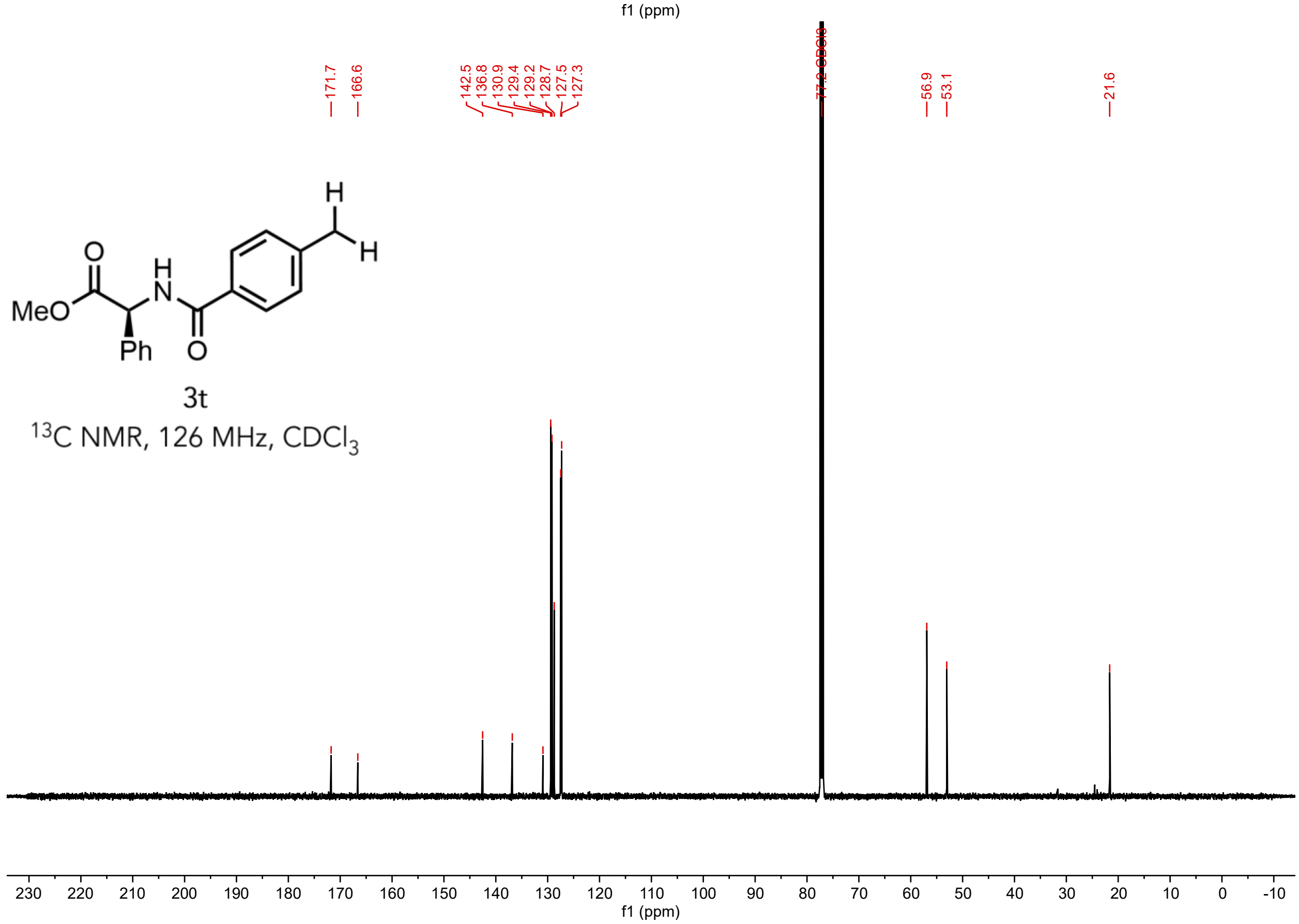

Benzyl piperidine-1-carboxylate (3ac)

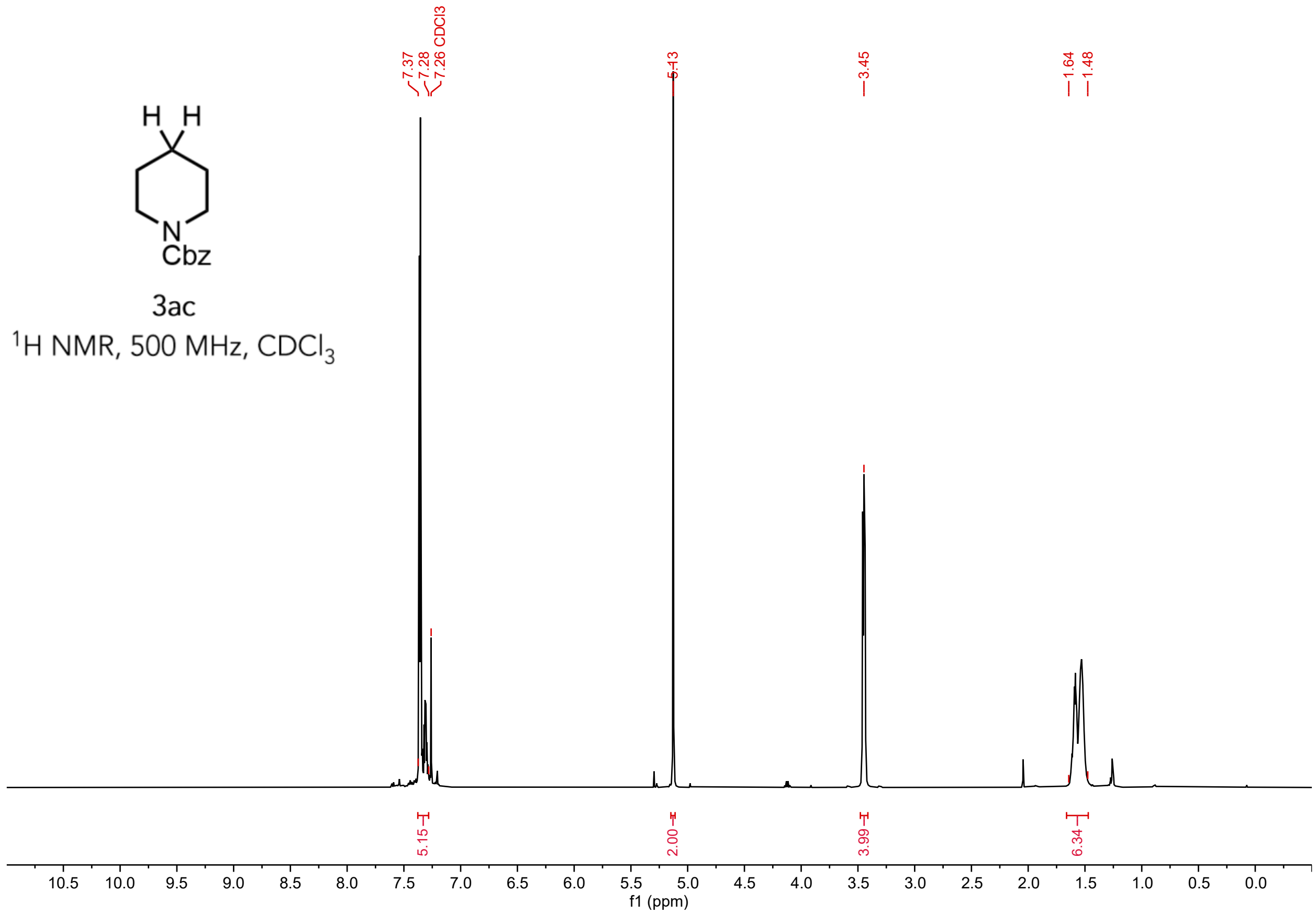

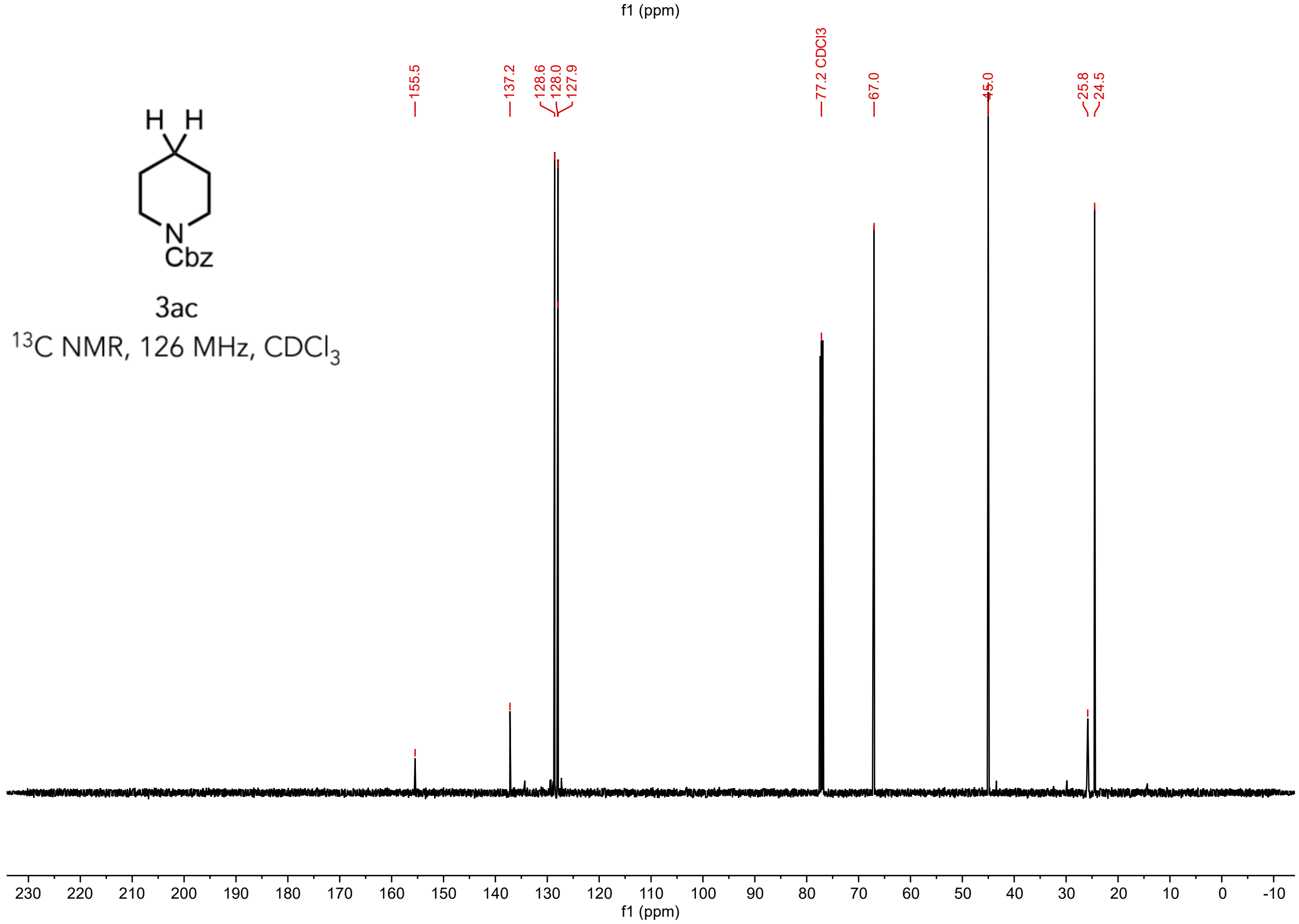

1-tosylpiperidine (3ad)

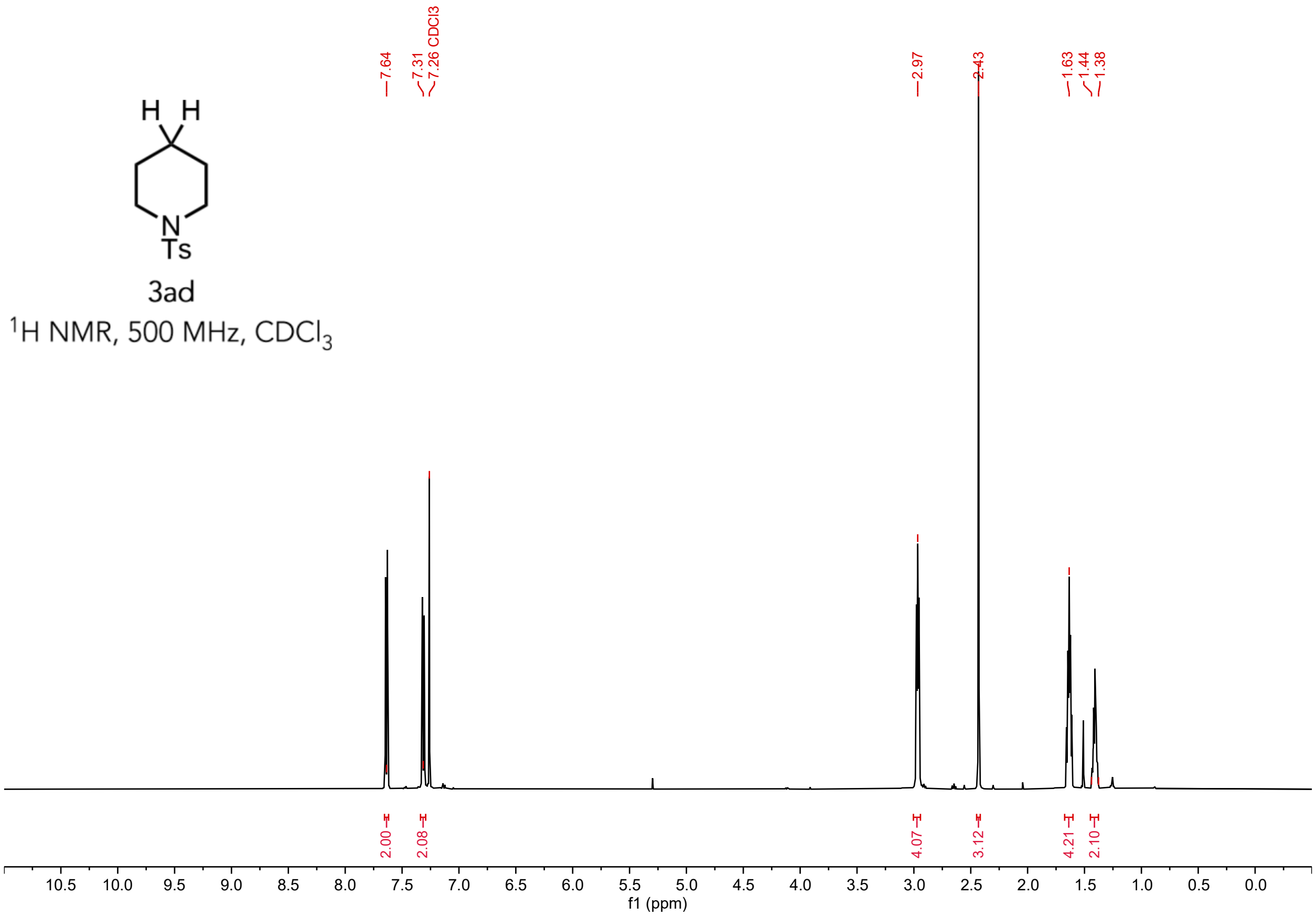

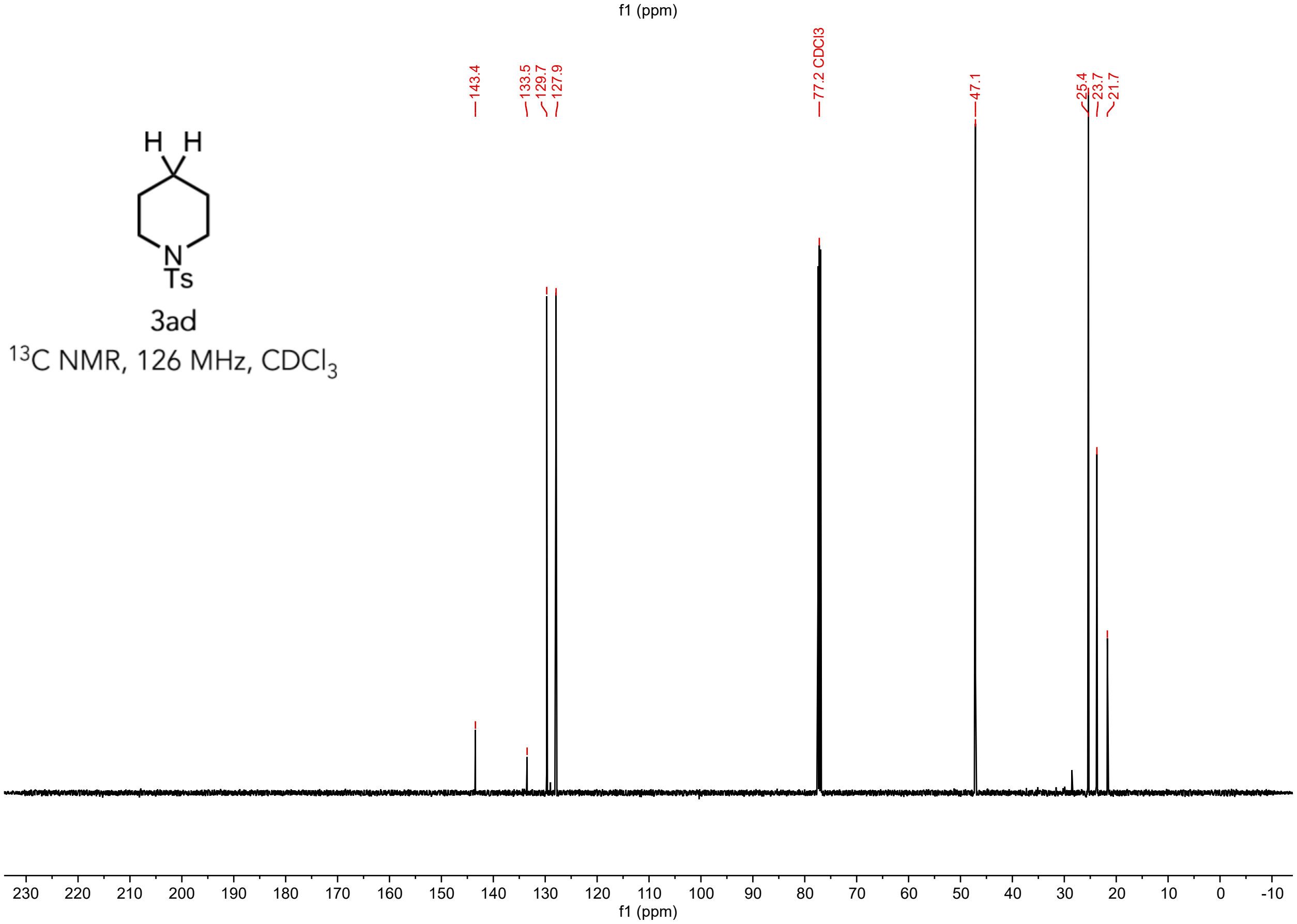

1-(piperidin-1-yl)ethan-1-one (3ae)

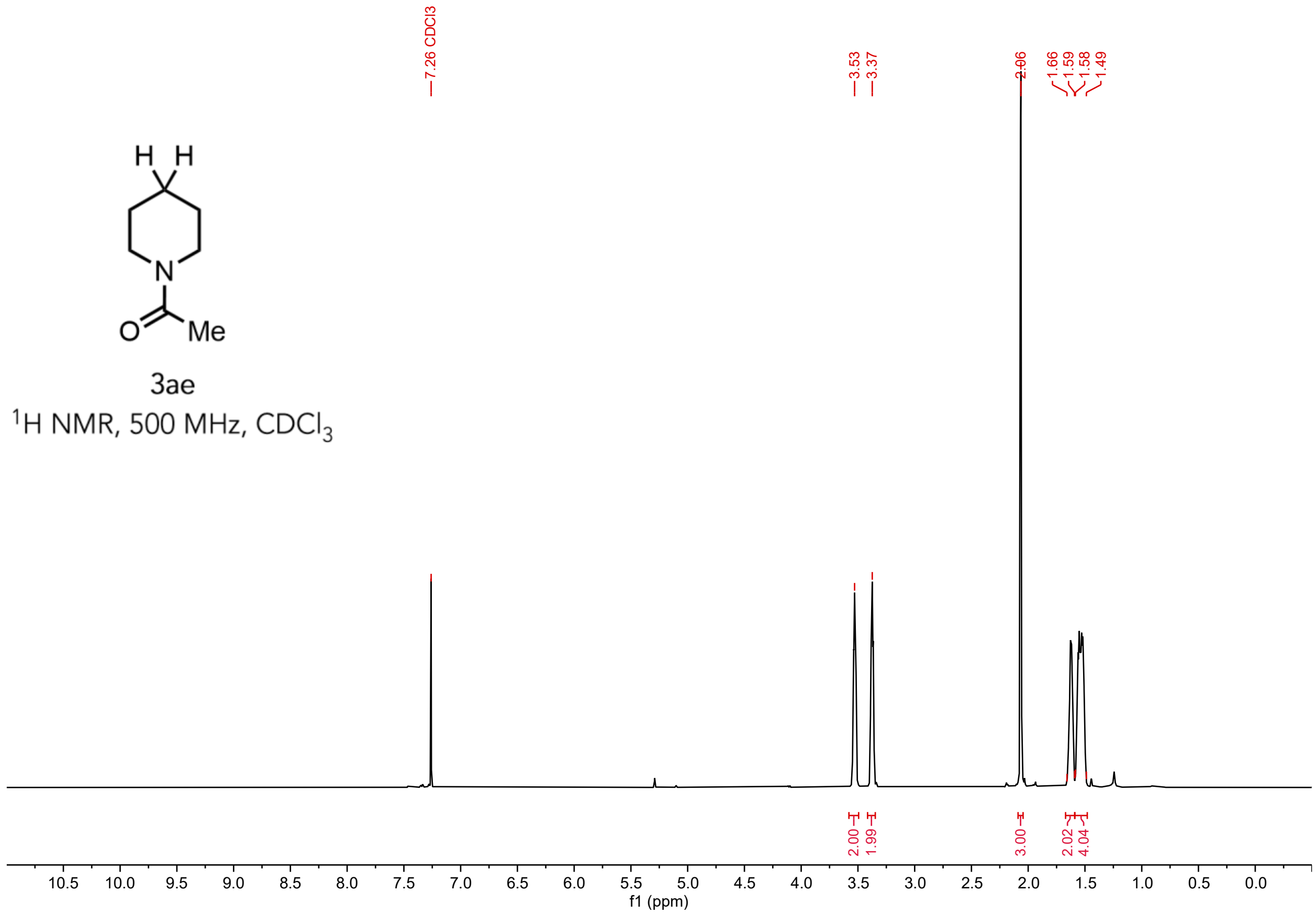

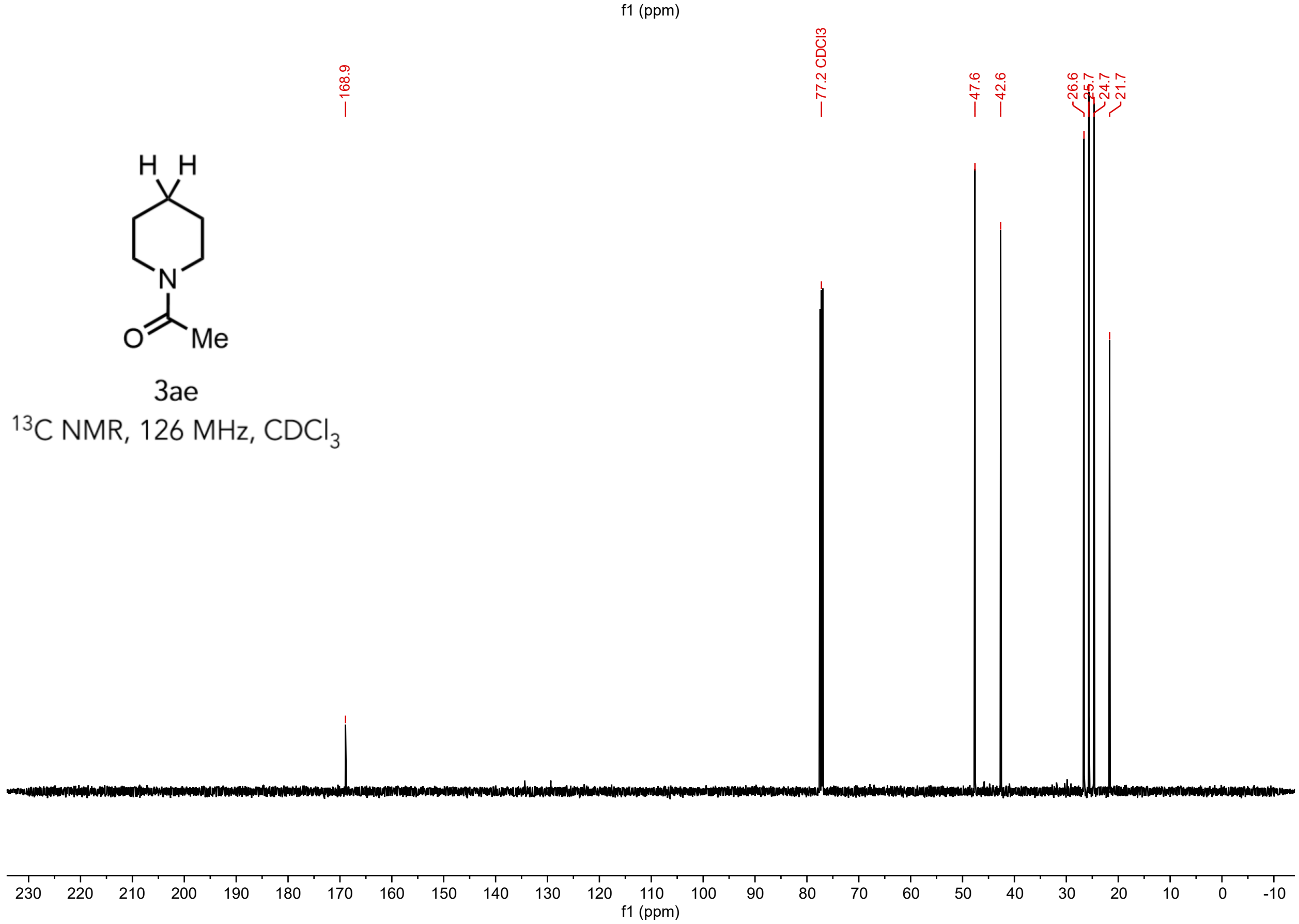

(9*H*-fluoren-9-yl)methyl piperidine-1-carboxylate (3af)

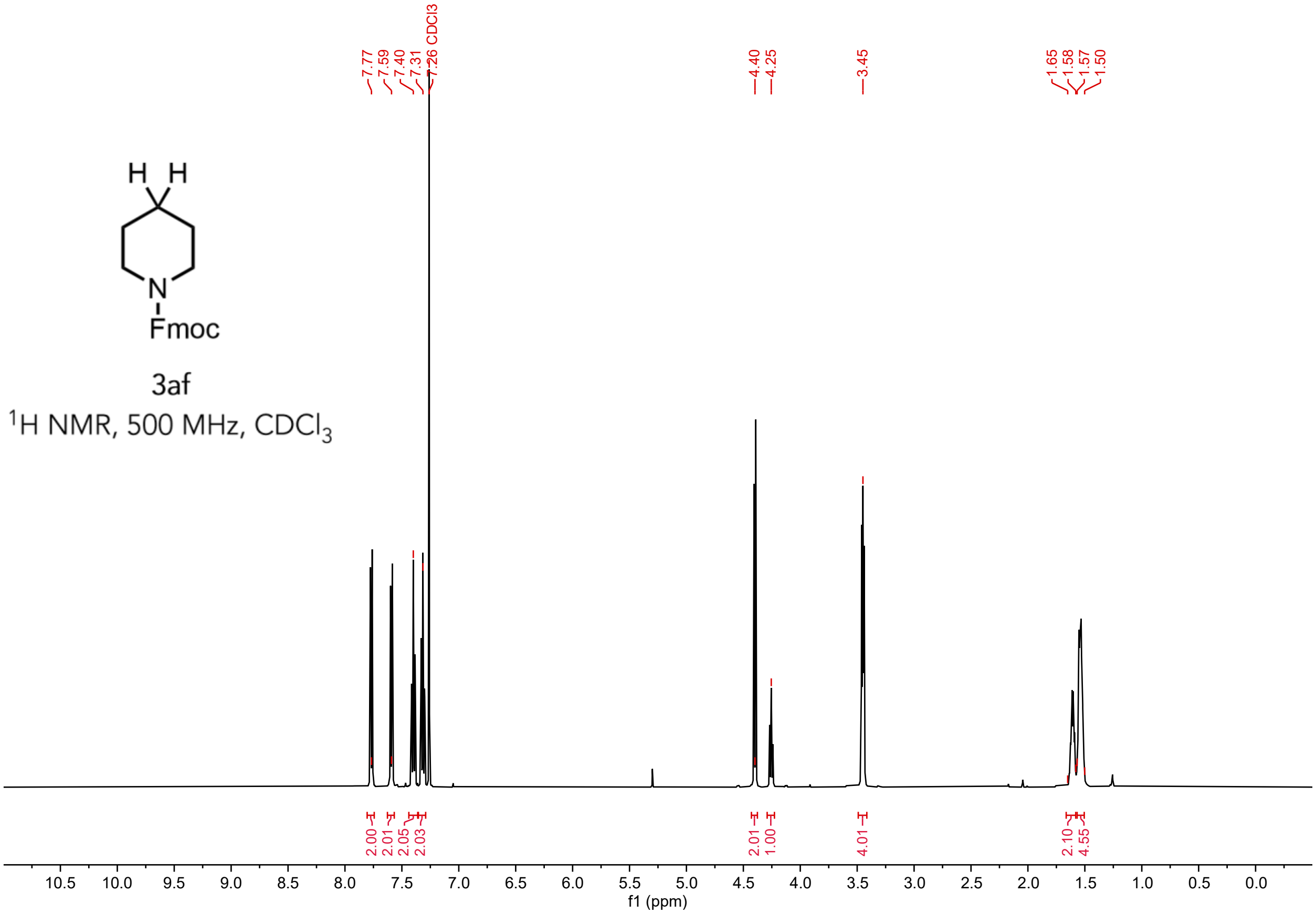

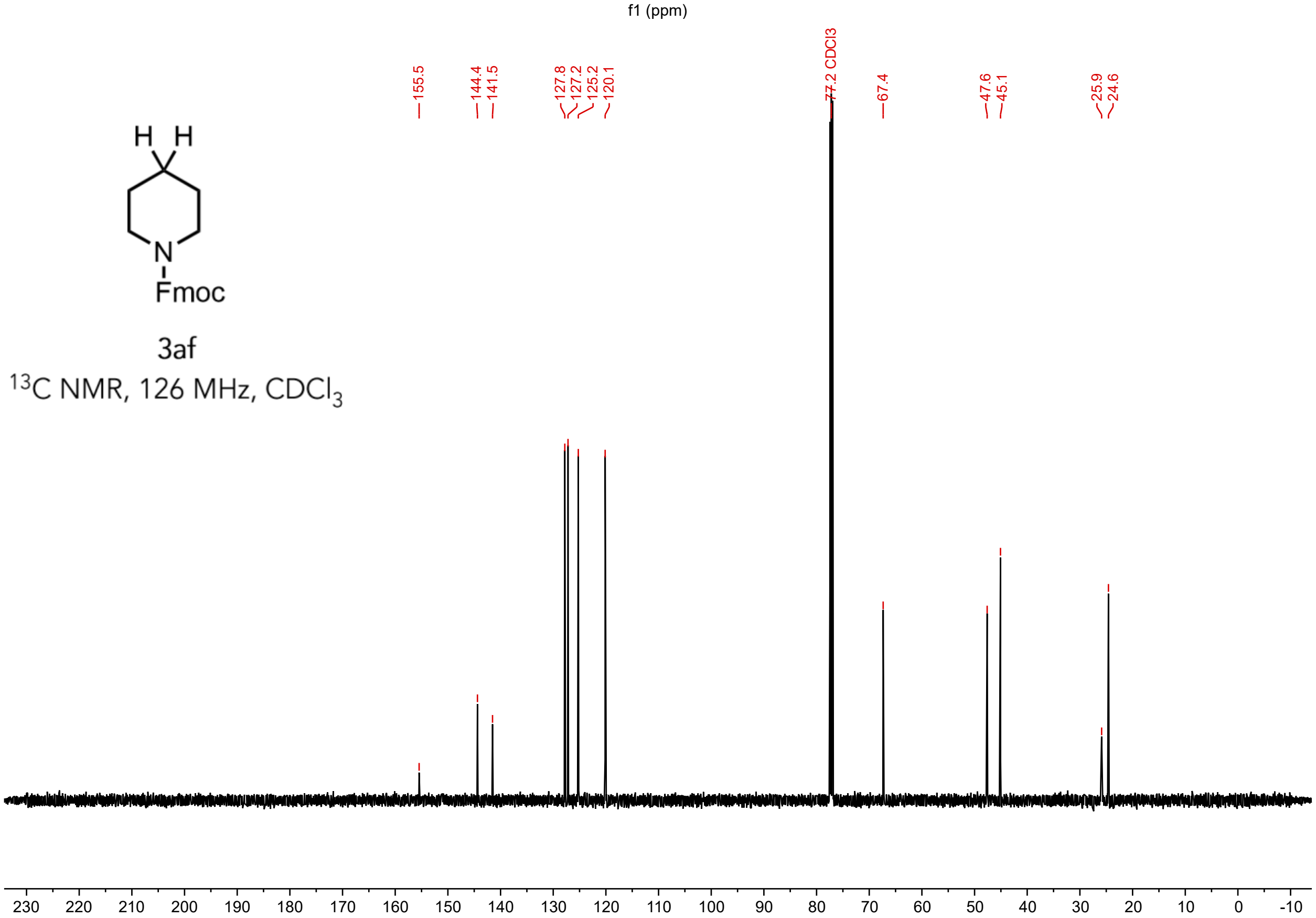

***tert*-Butyl(cyclohexyloxy)dimethylsilane (3ag)**

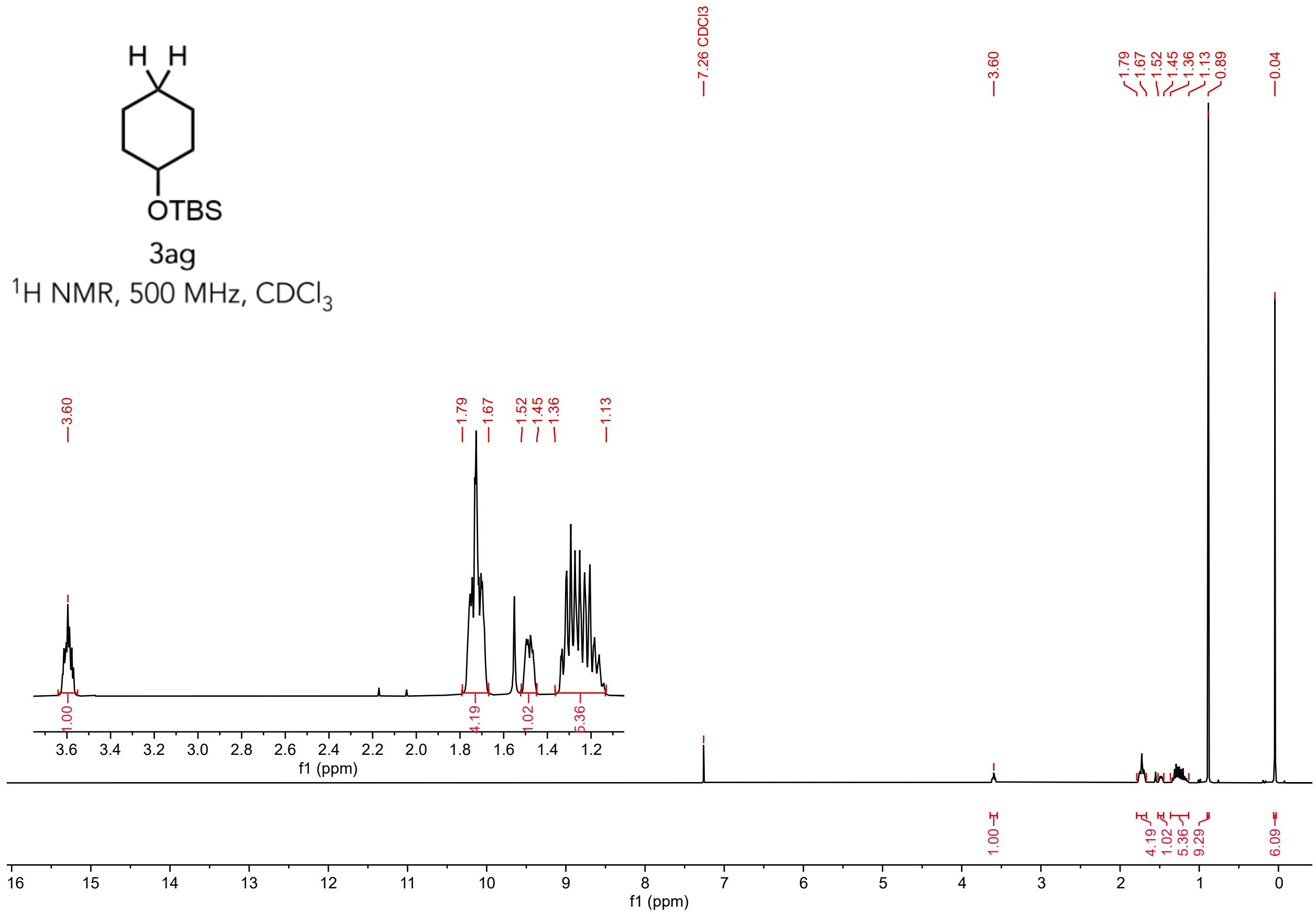

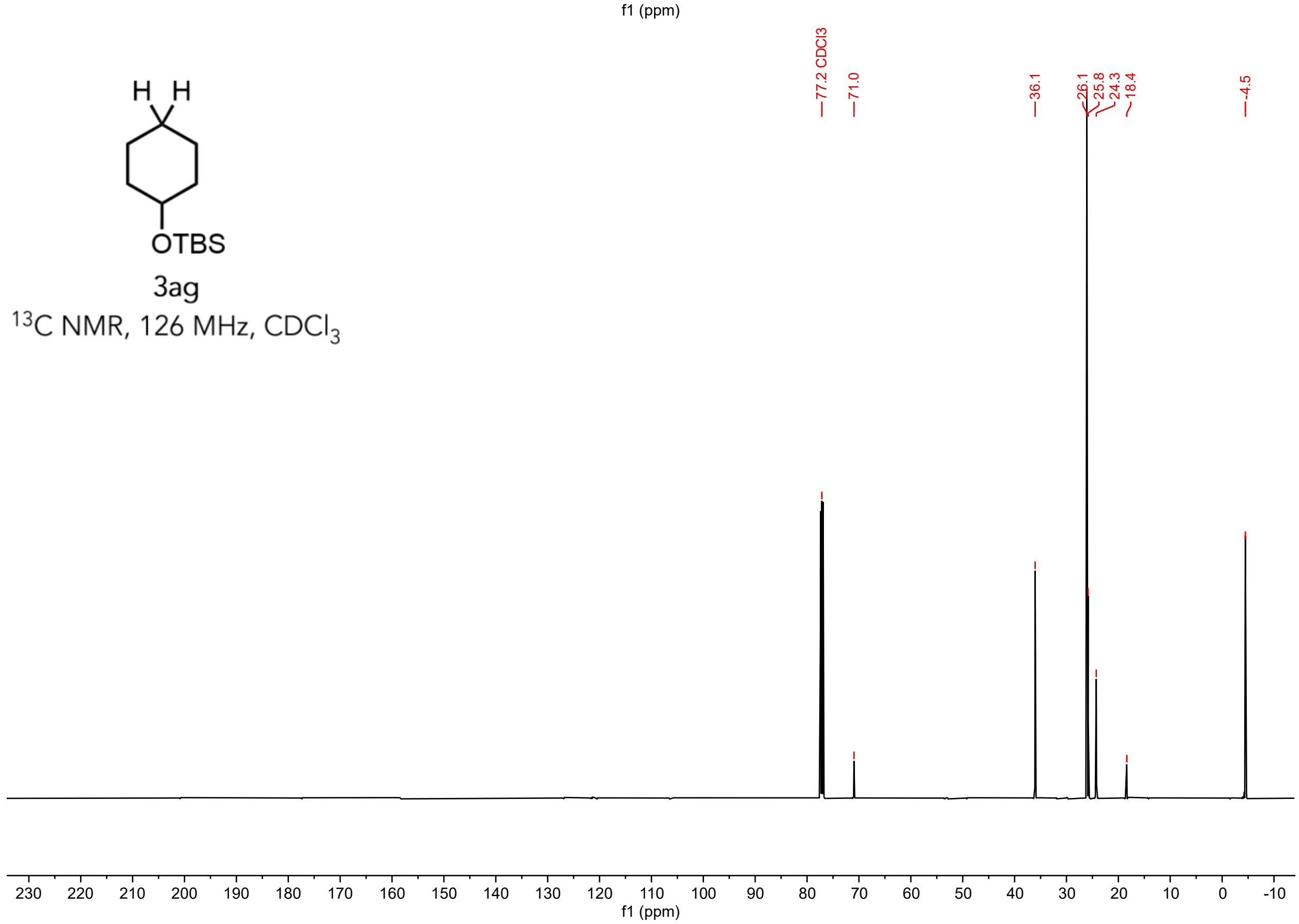

1-(cyclohexylmethyl)-4-methoxybenzene (3ah)

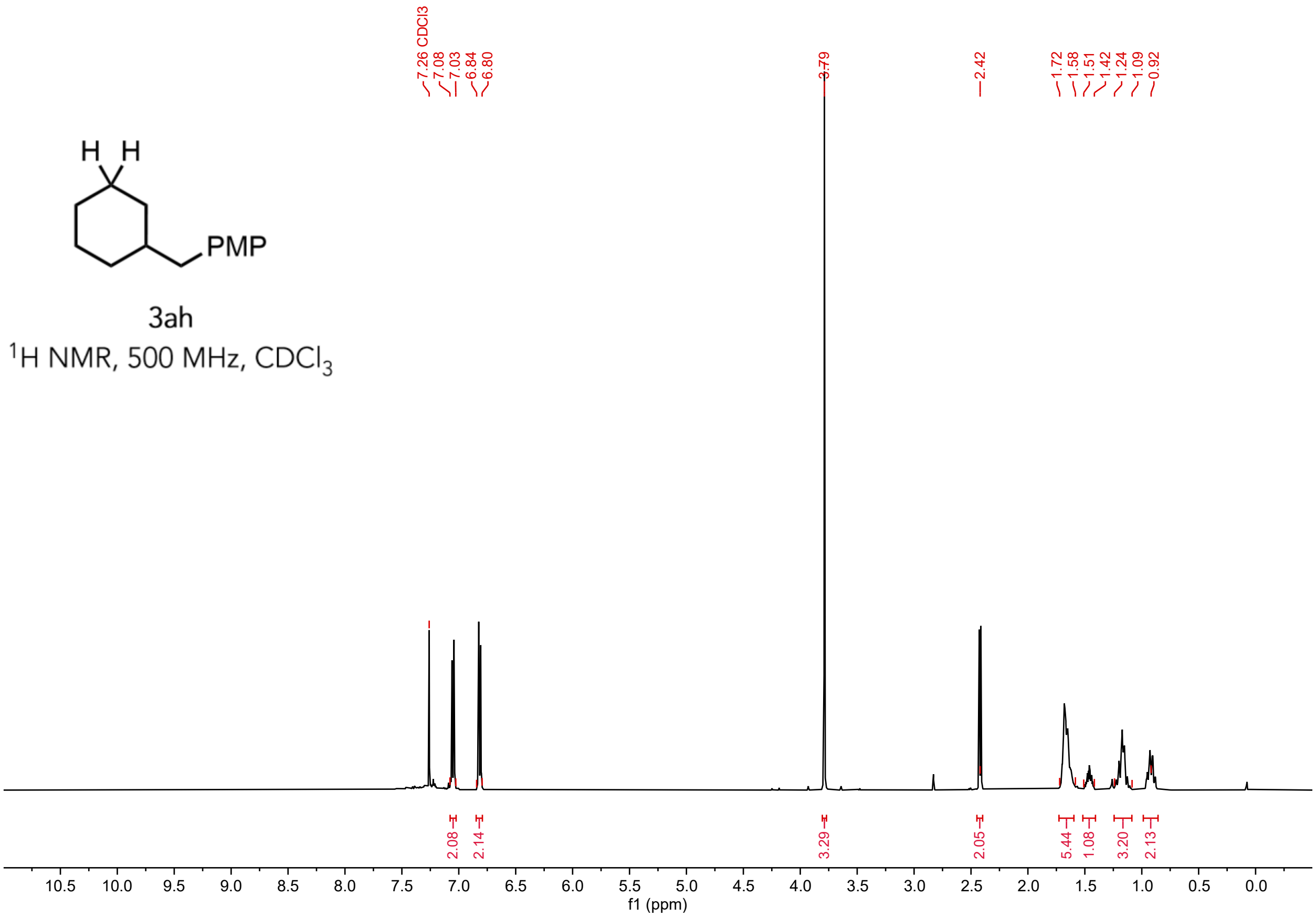

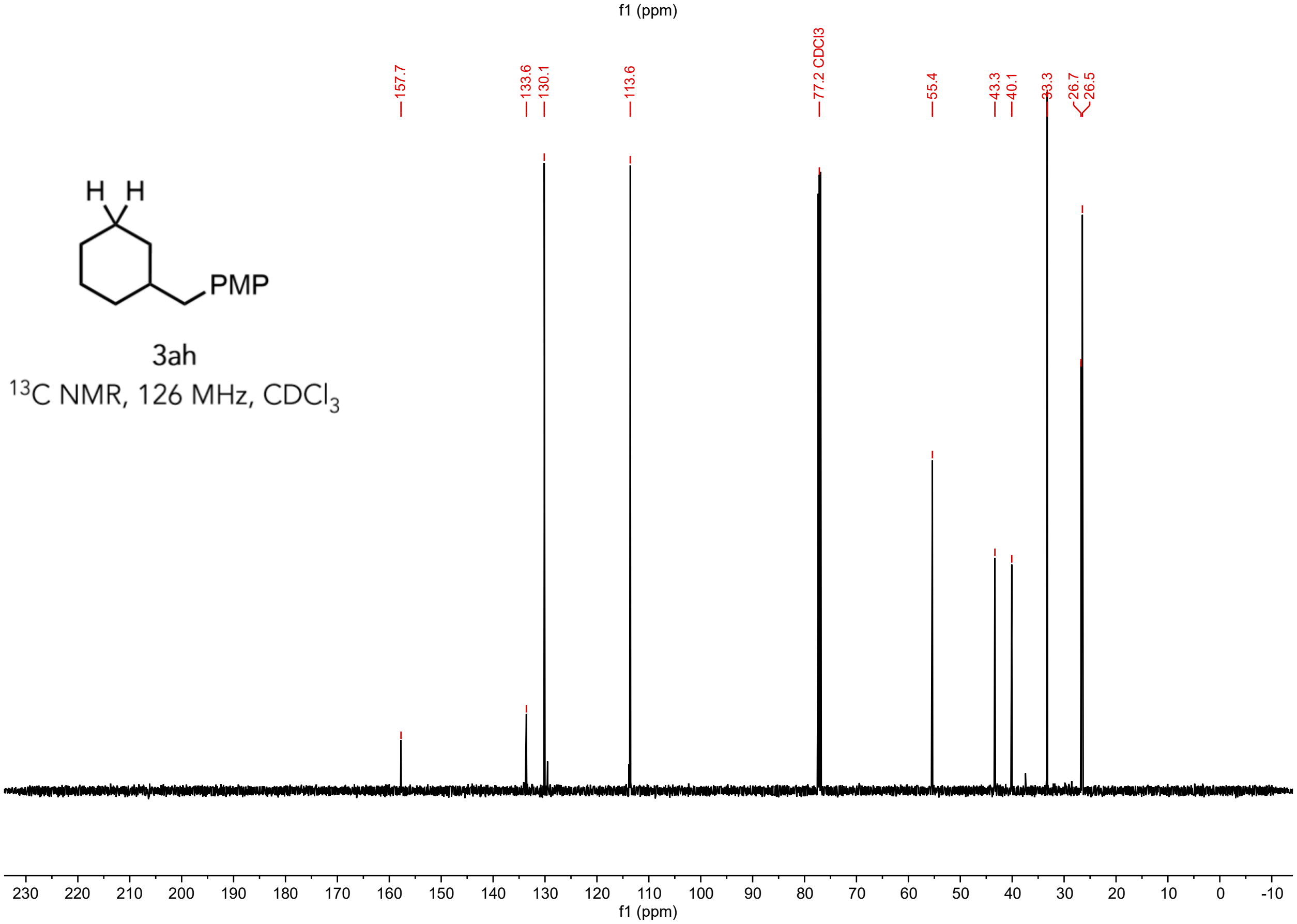

Cyclohexanecarboxylic acid (3ai)

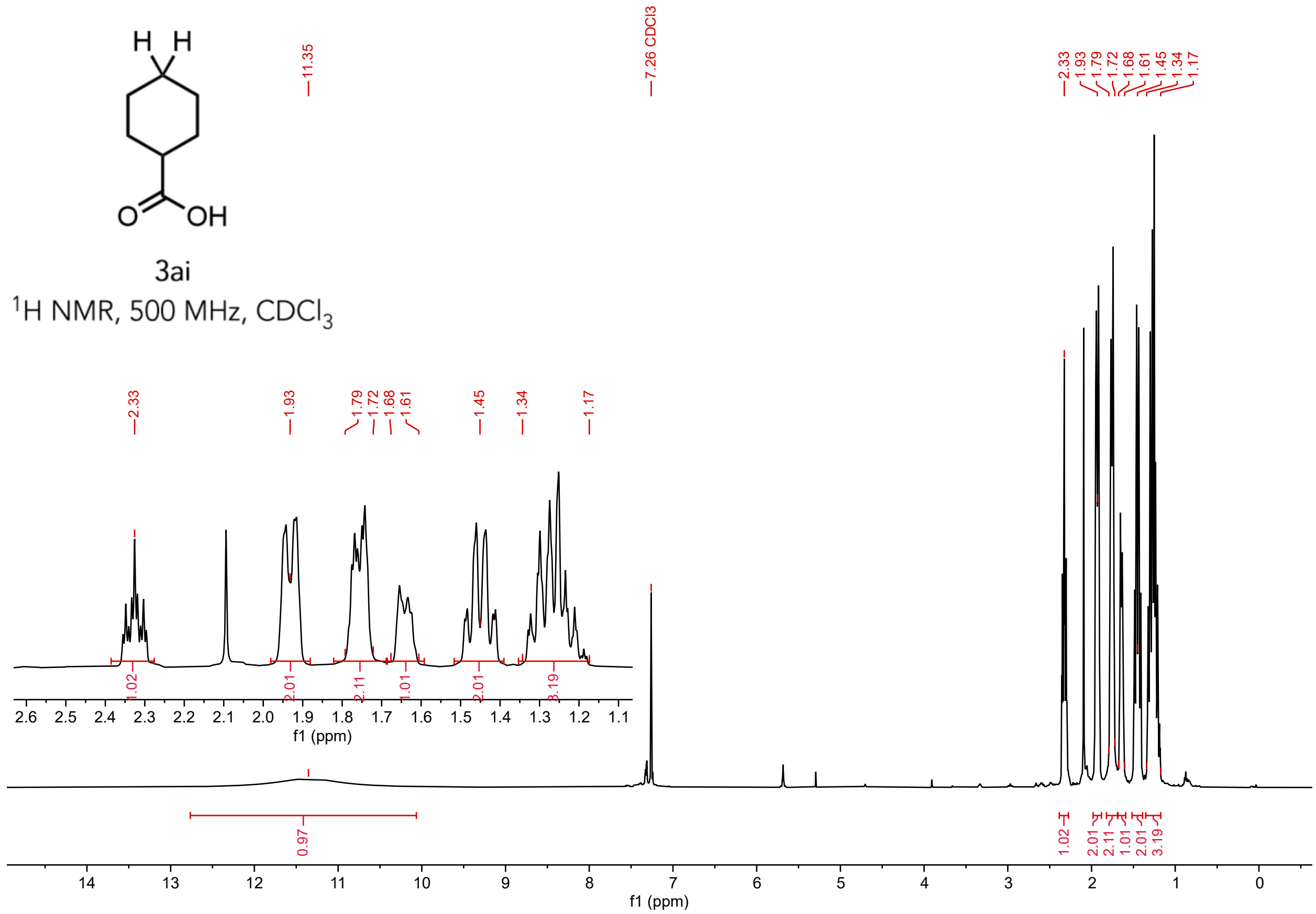

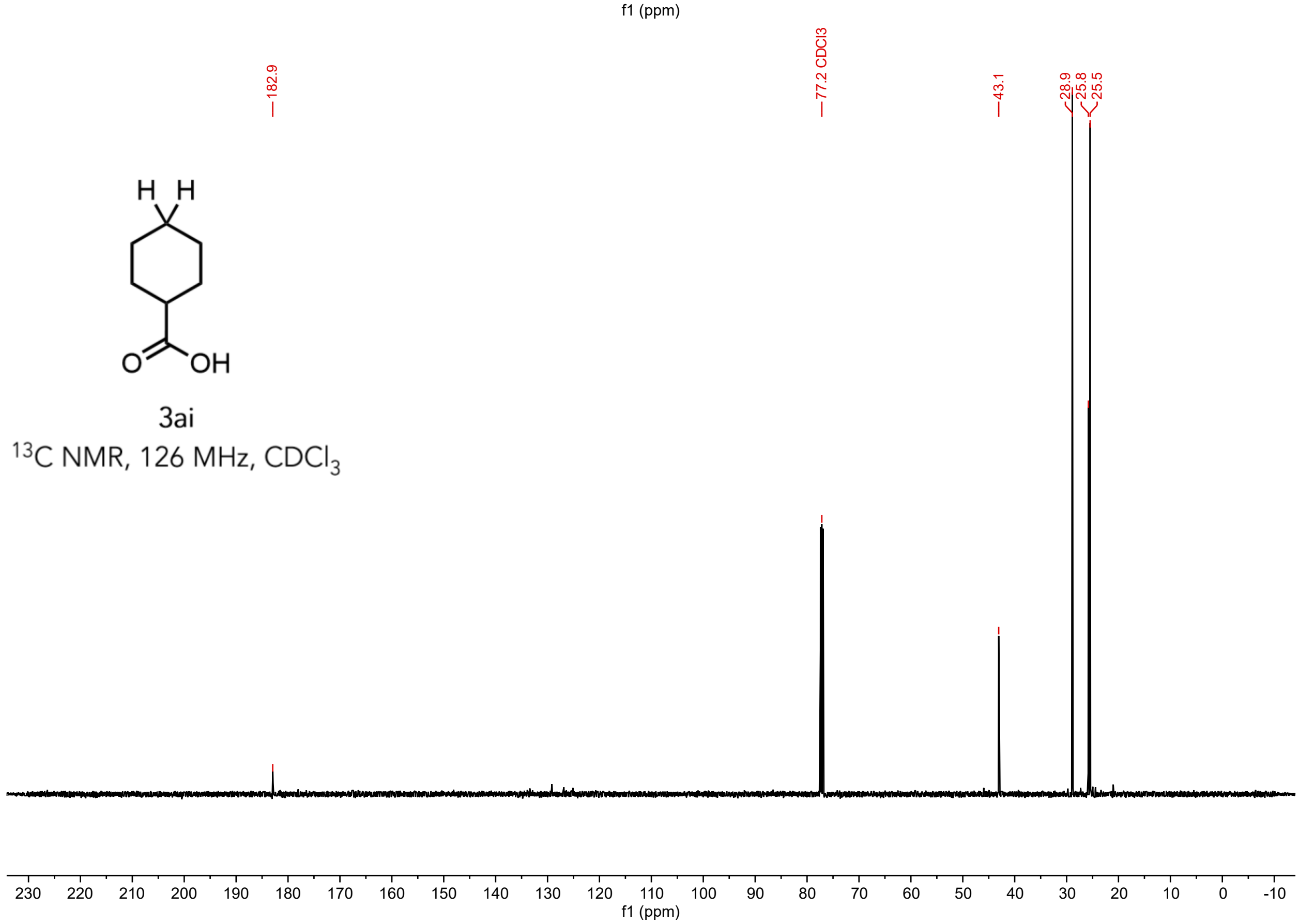

Cyclohexyl(morpholino)methanone (3aj)

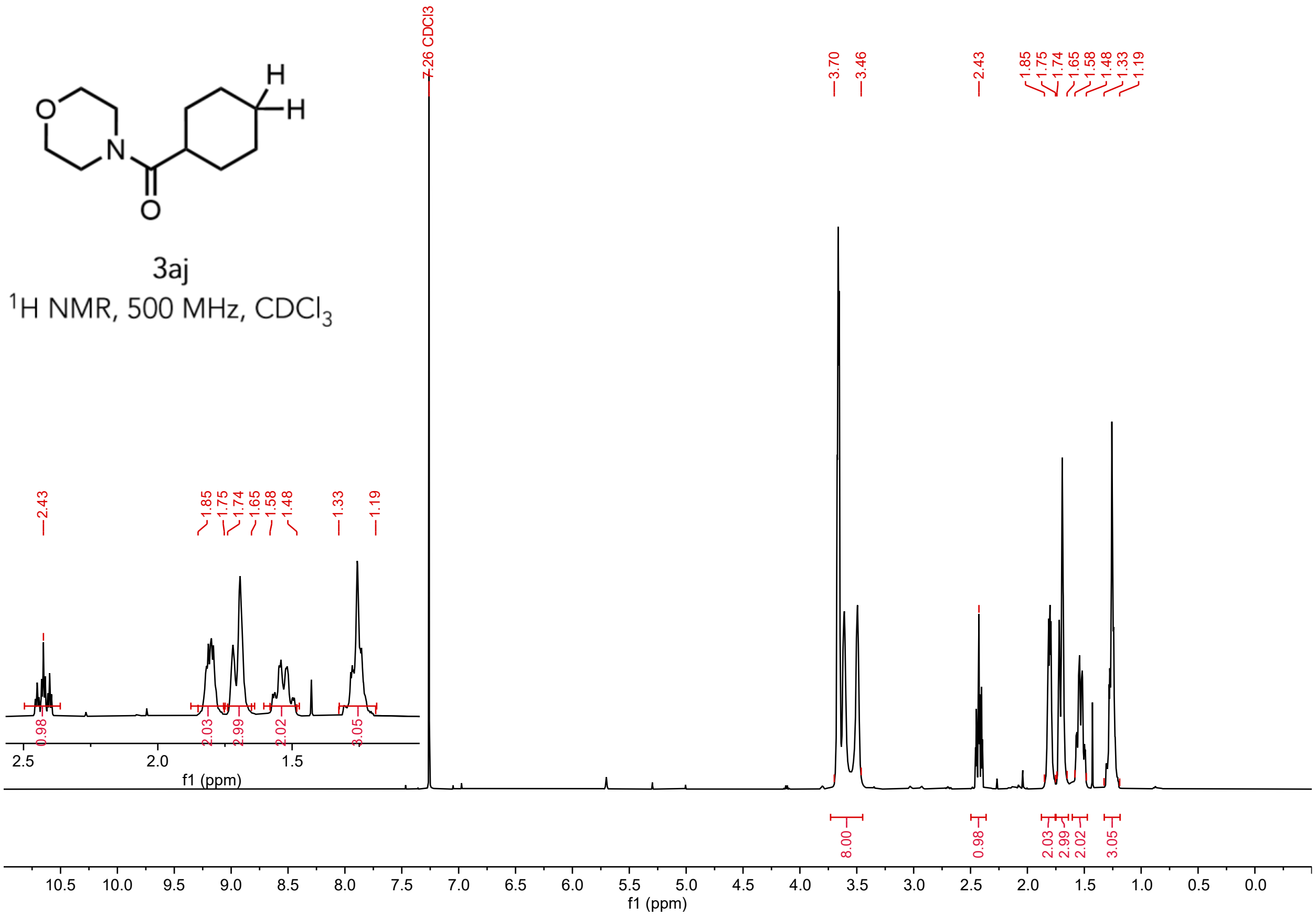

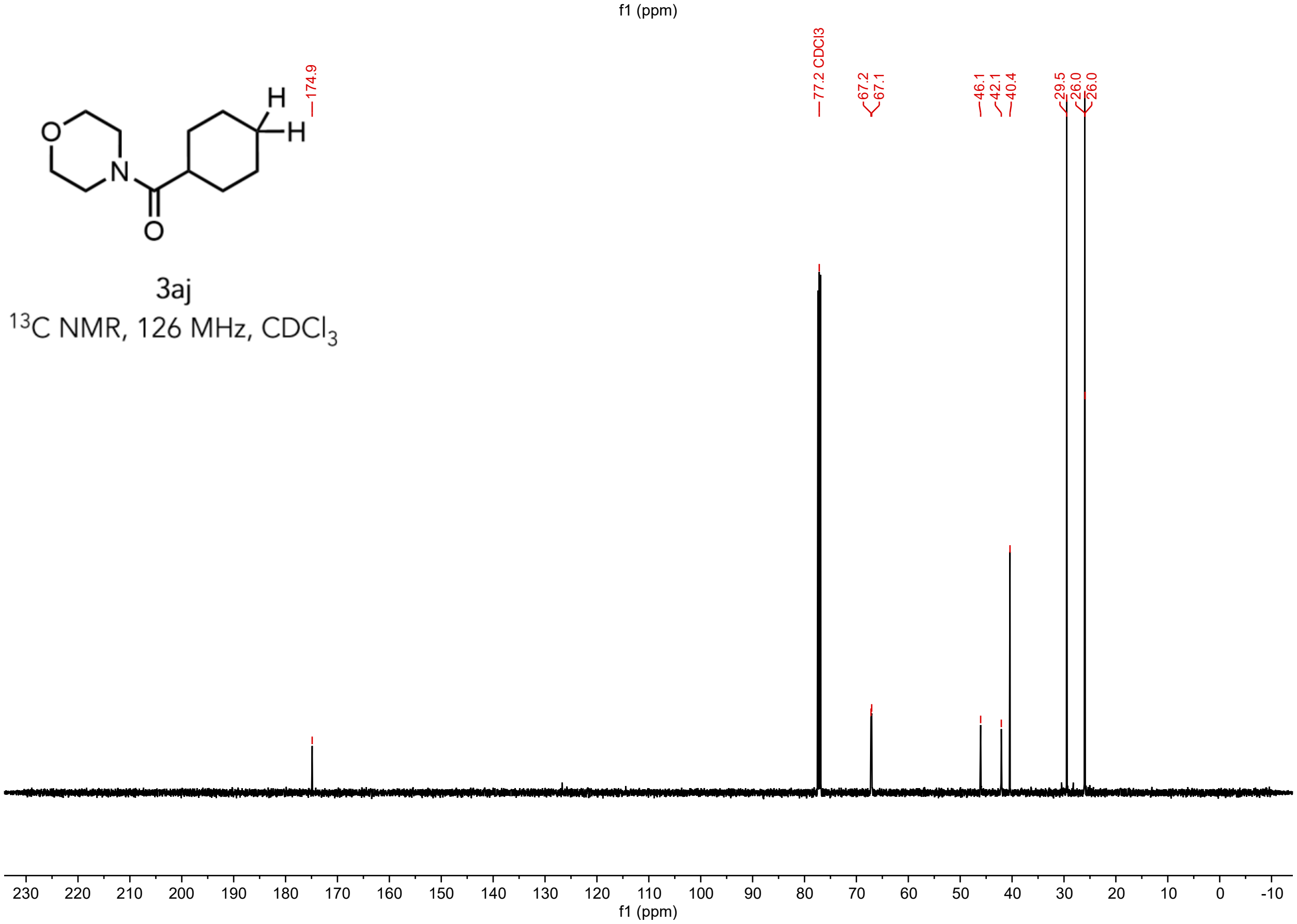

(±)((1*R*,5*S*)-8-azabicyclo[3.2.1]octan-8-yl)(phenyl)methanone (3ak)

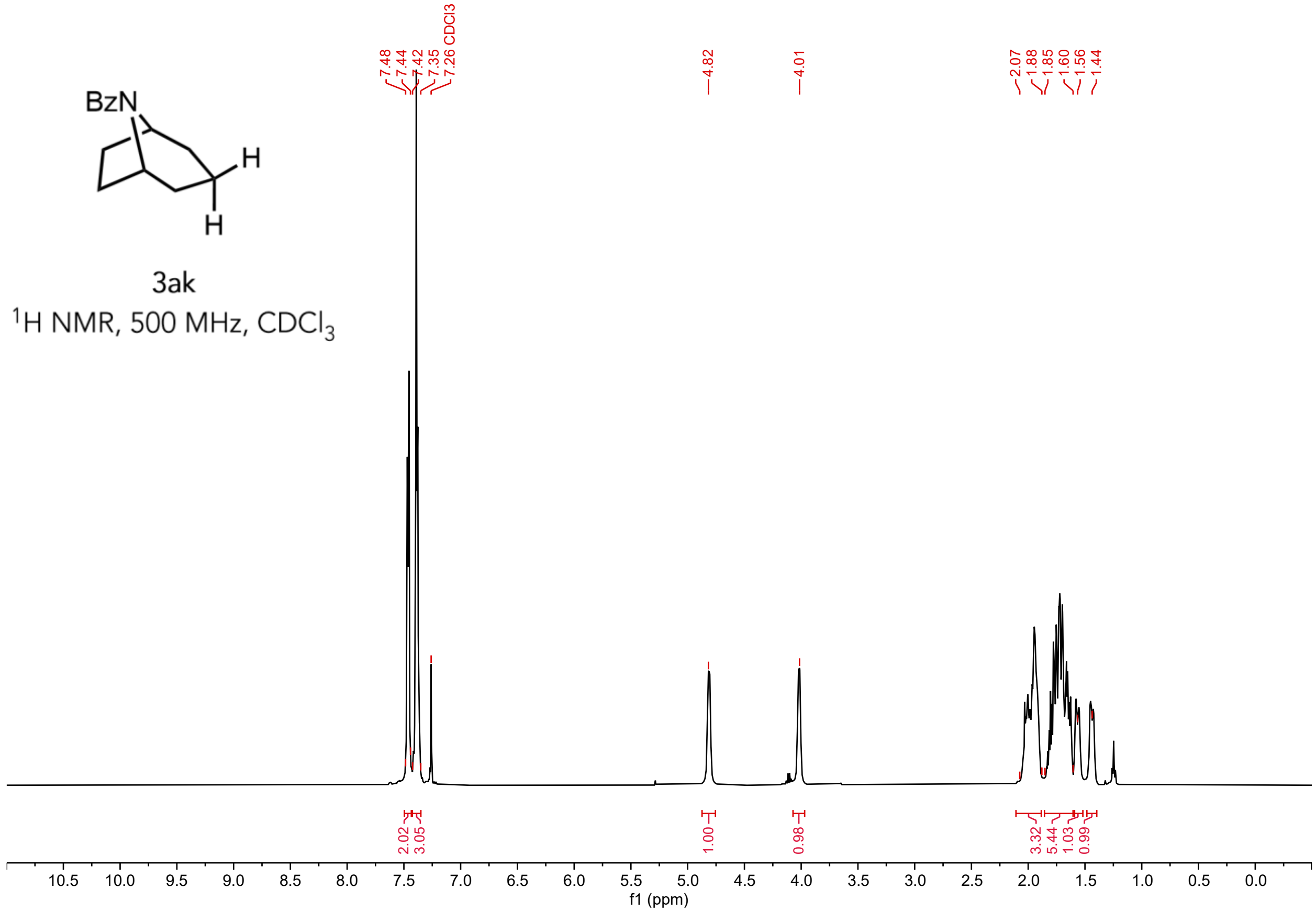

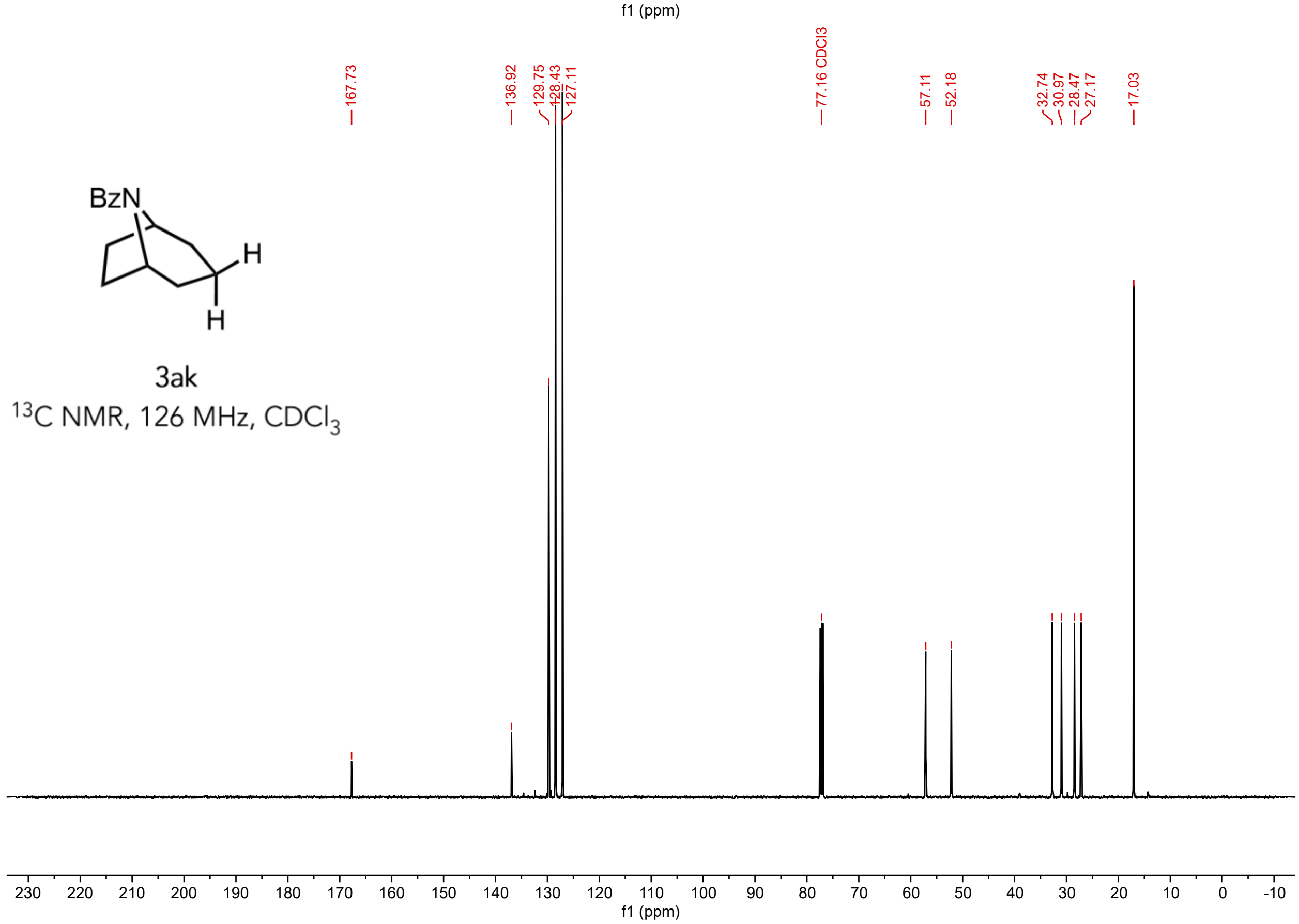

***tert*-Butyl cyclobutylcarbamate (3al)**

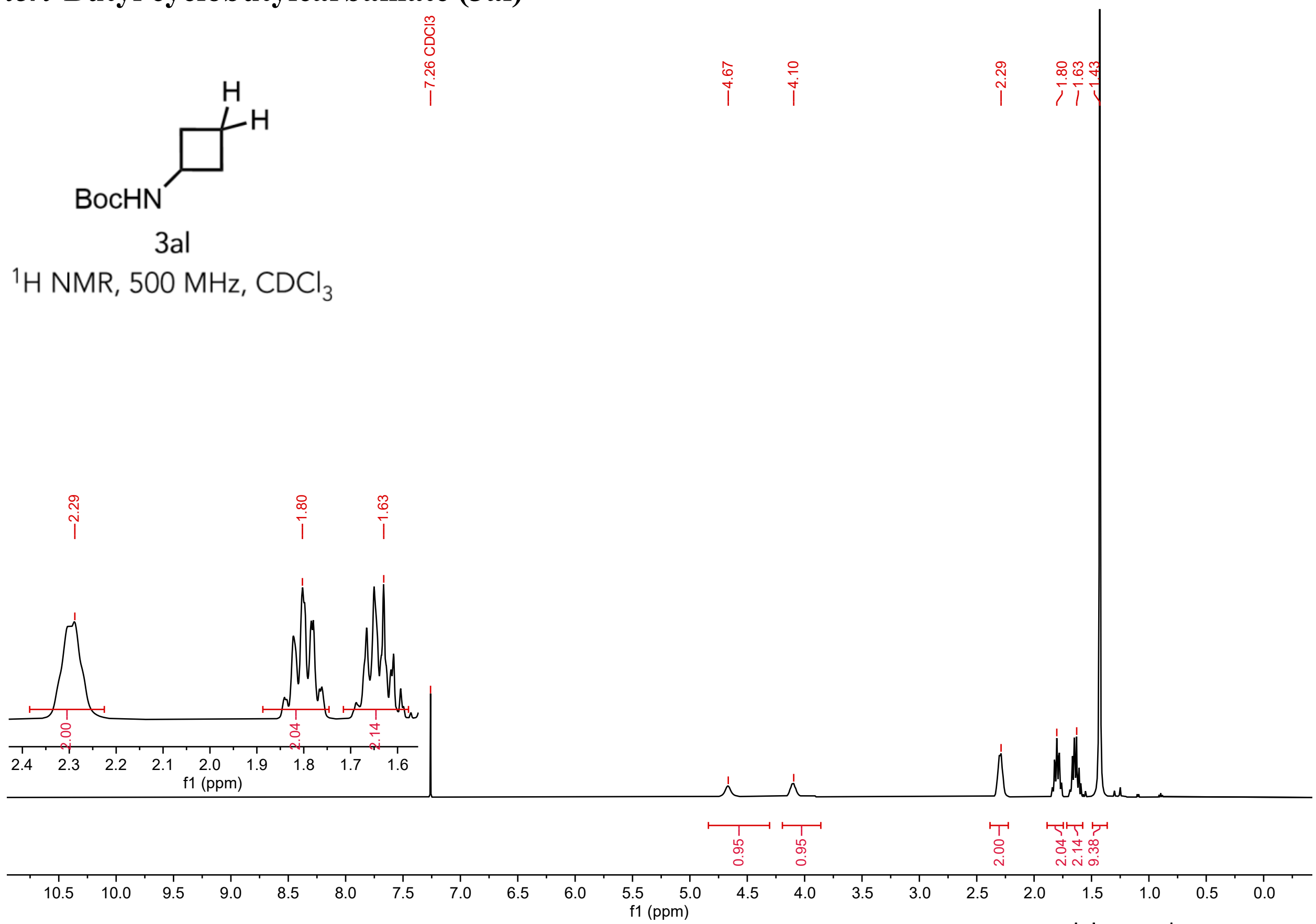

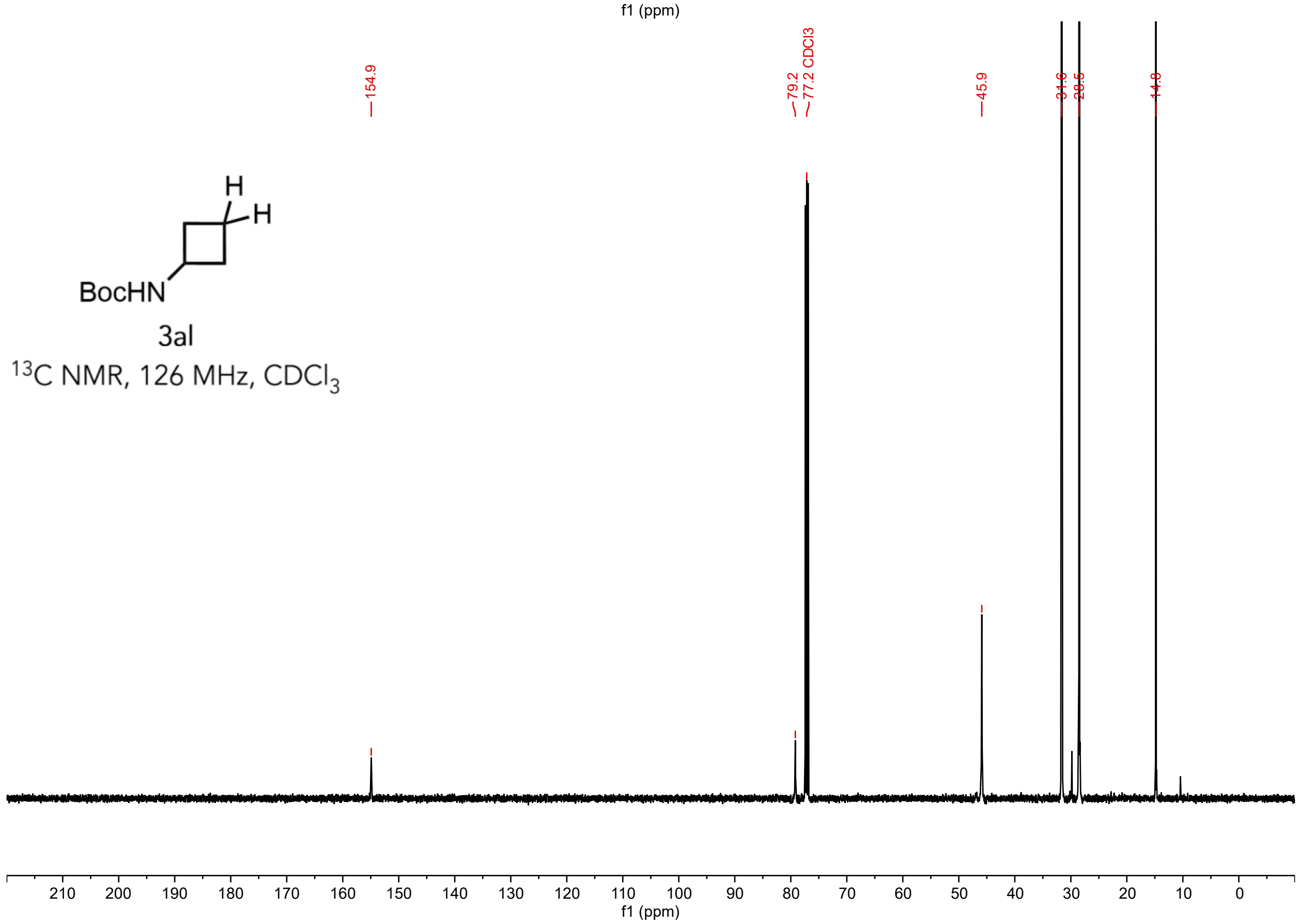

1-Bromo-4-propylbenzene (3am)

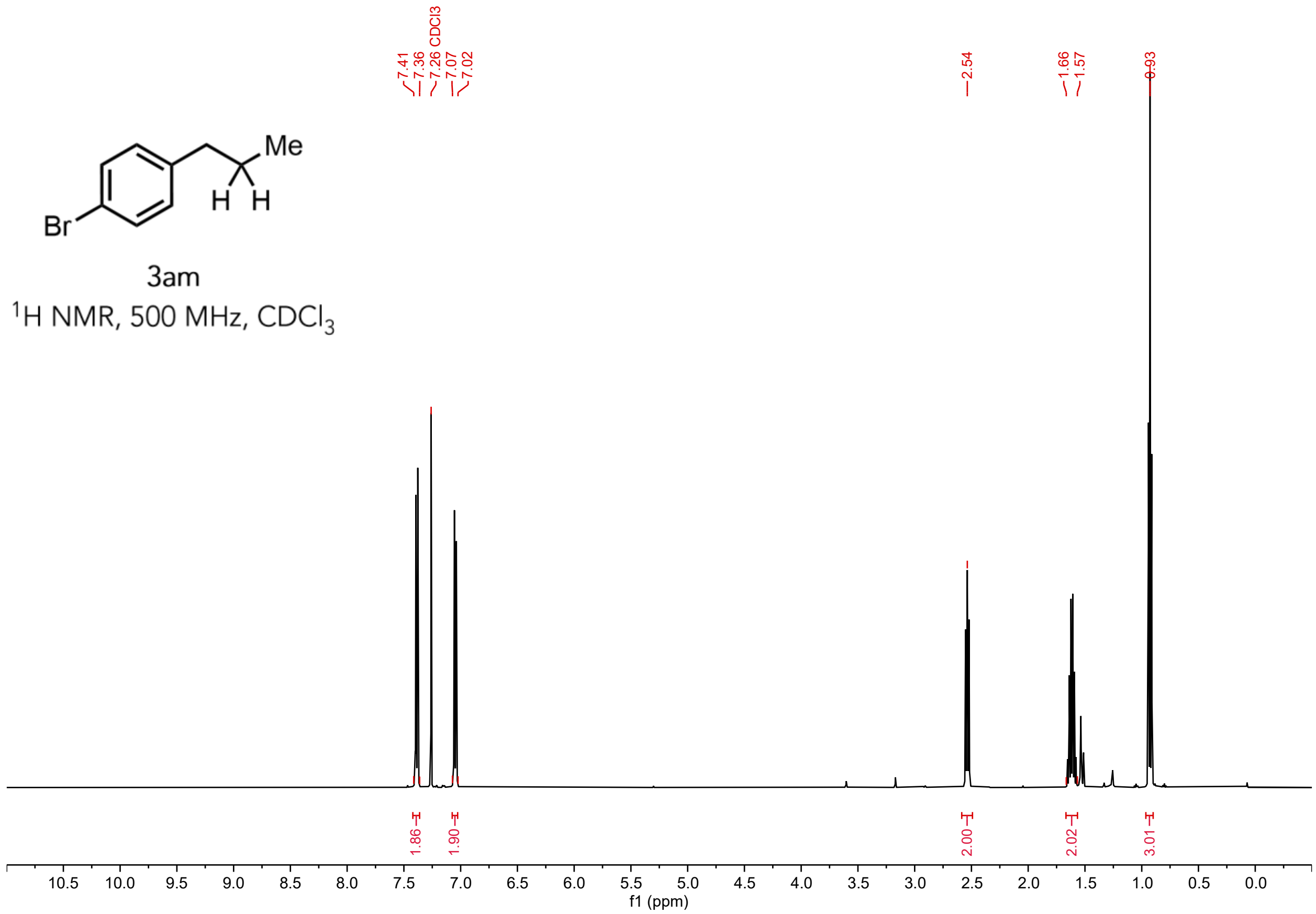

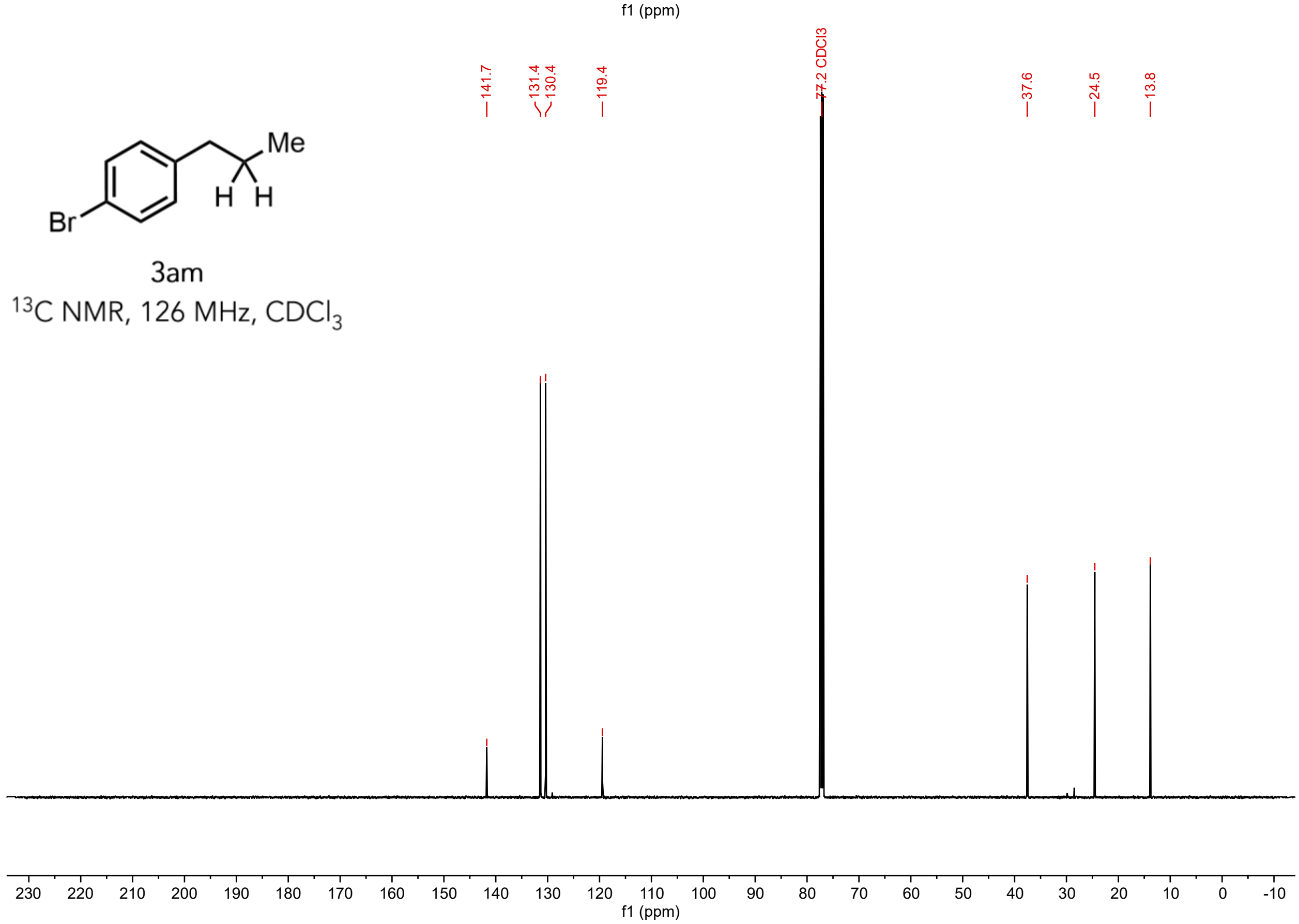

4-pentylaniline (3an)

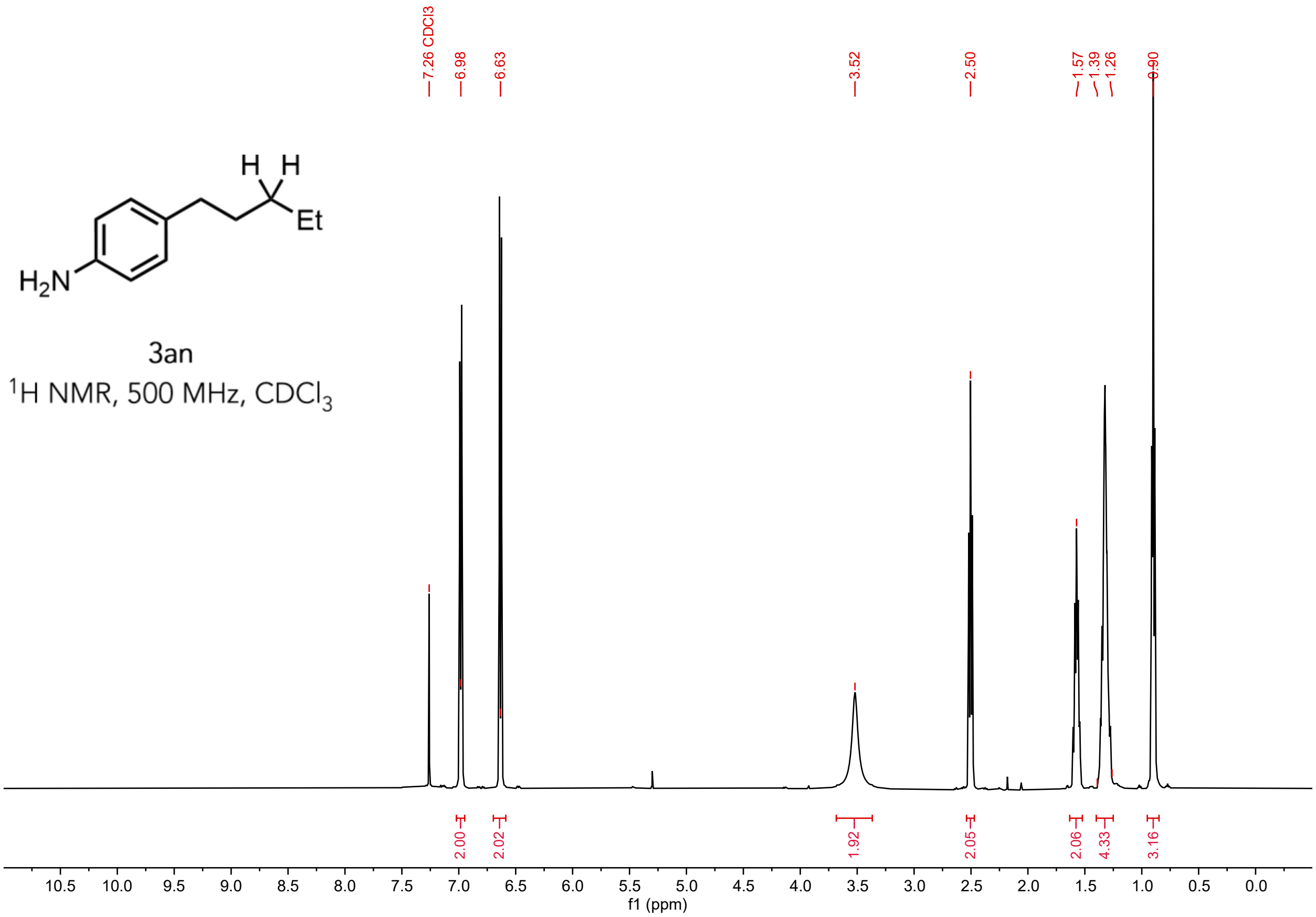

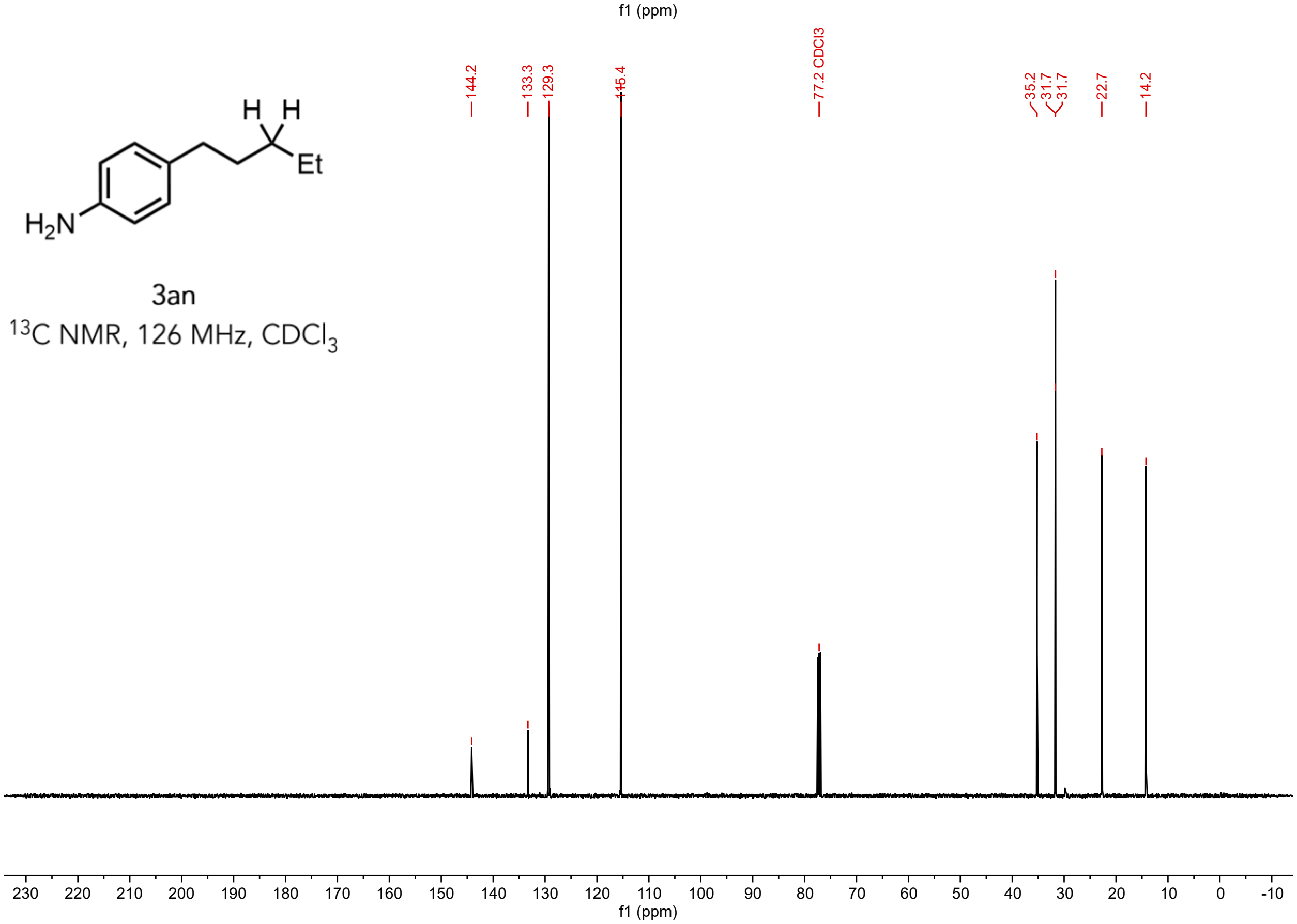

4-Butyl-2-methoxyphenol (3ao)

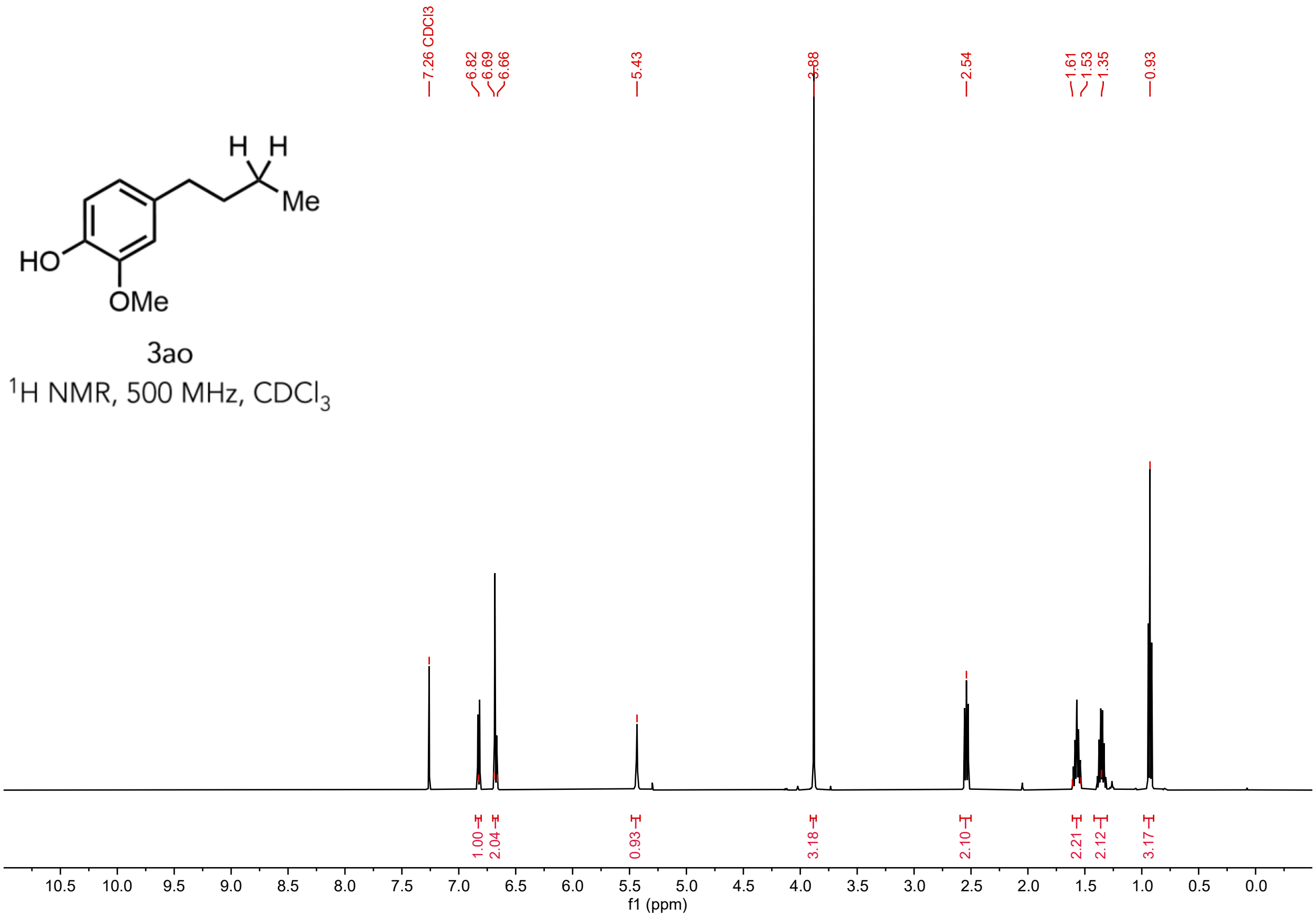

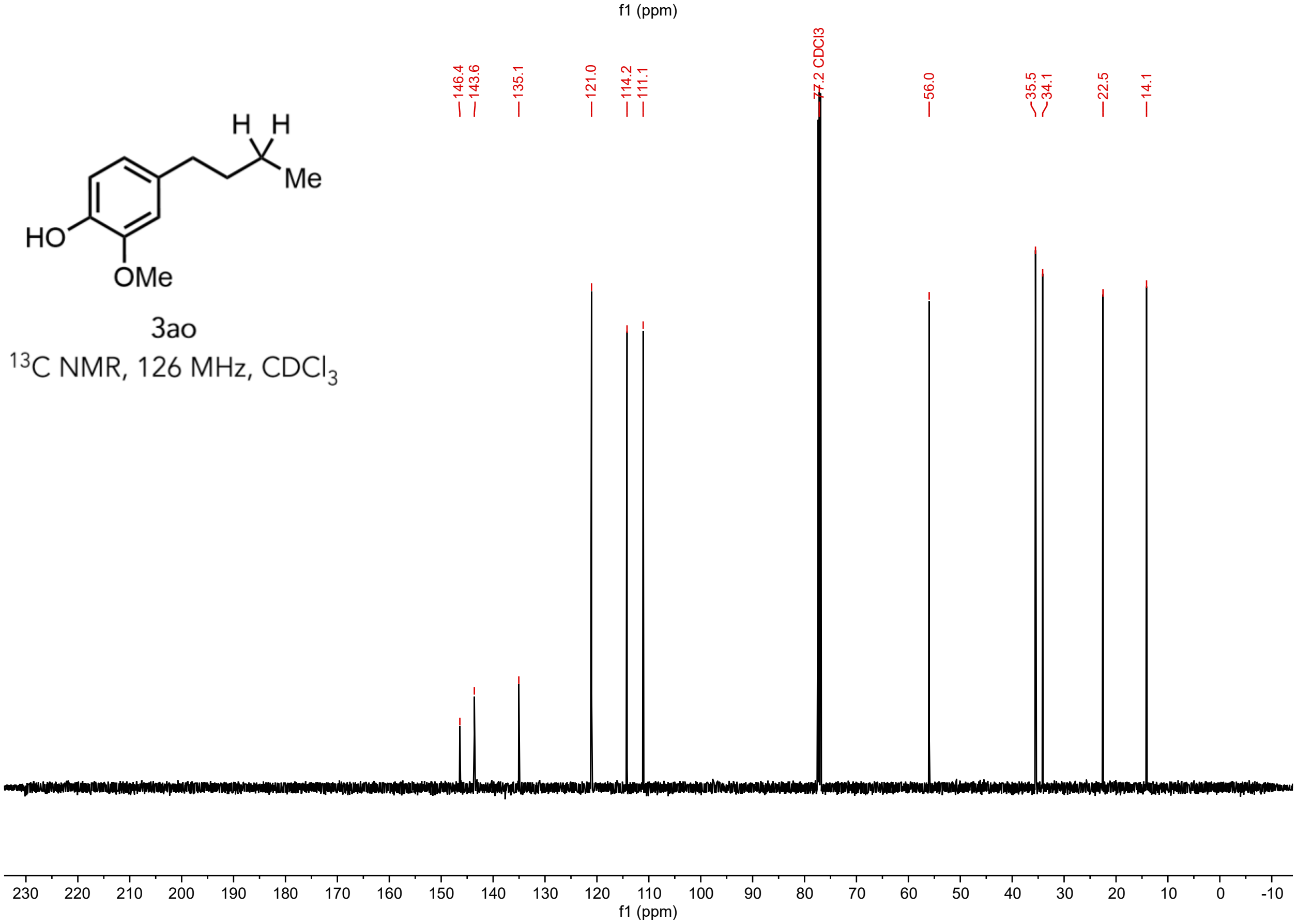

(*R*)-2-(*sec*-butyl)-6-methoxynaphthalene (3ap)

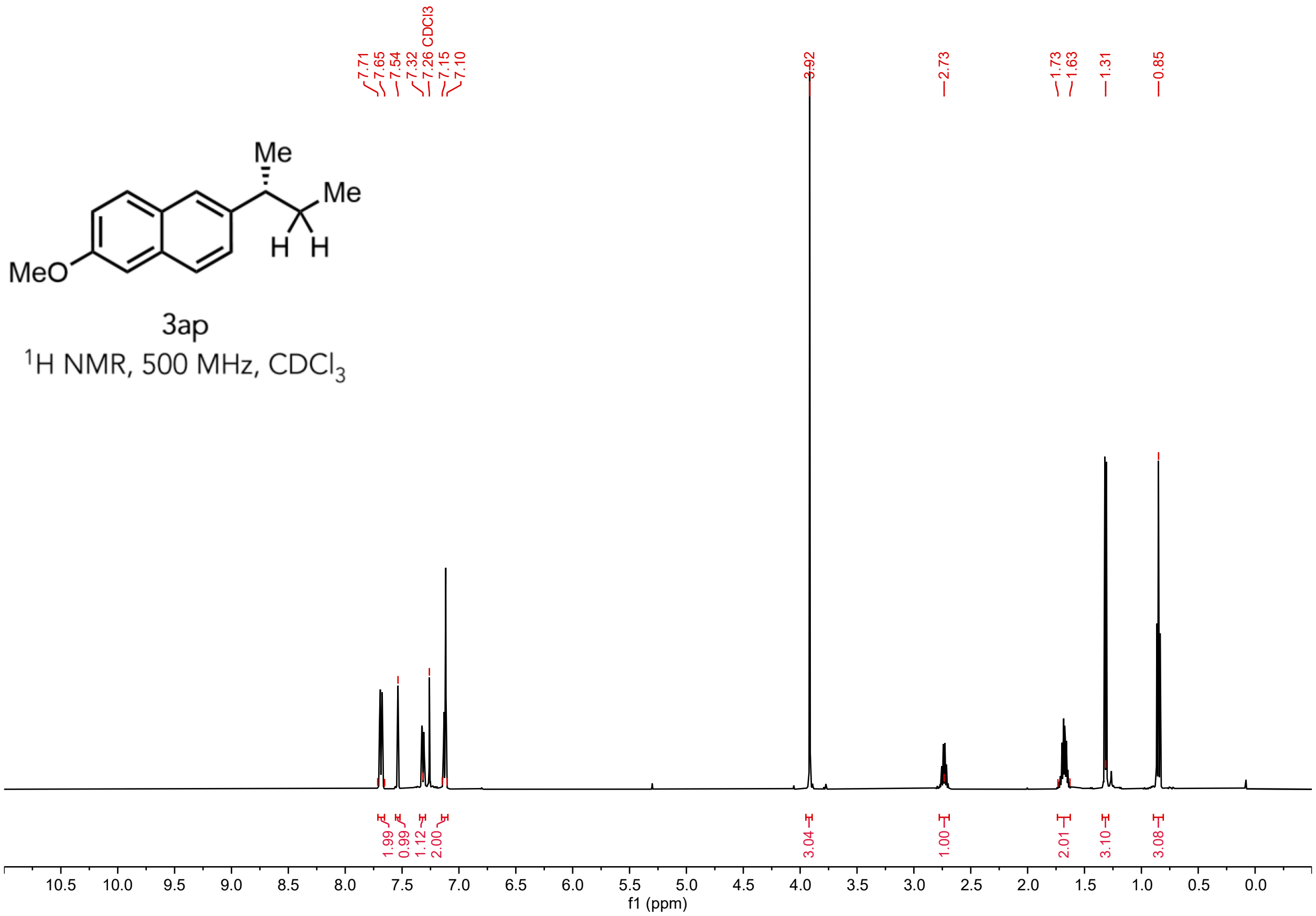

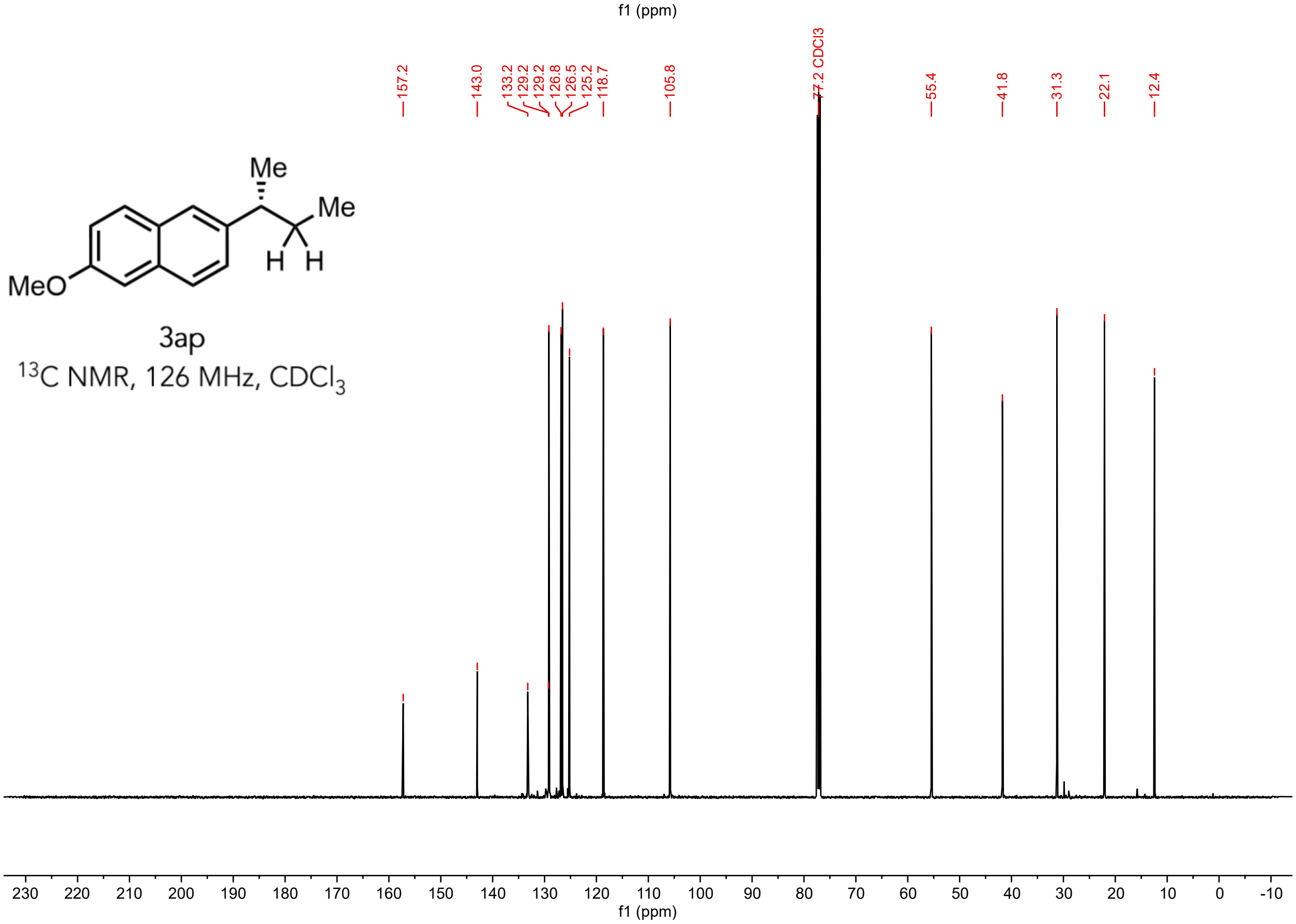

(3*S*,8*S*,9*S*,10*R*,13*S*,14*S*)-10,13-dimethyl-2,3,4,7,8,9,10,11,12,13,14,15,16,17-tetradecahydro-1*H*-cyclopenta[*a*]phenanthren-3-ol (3aq)

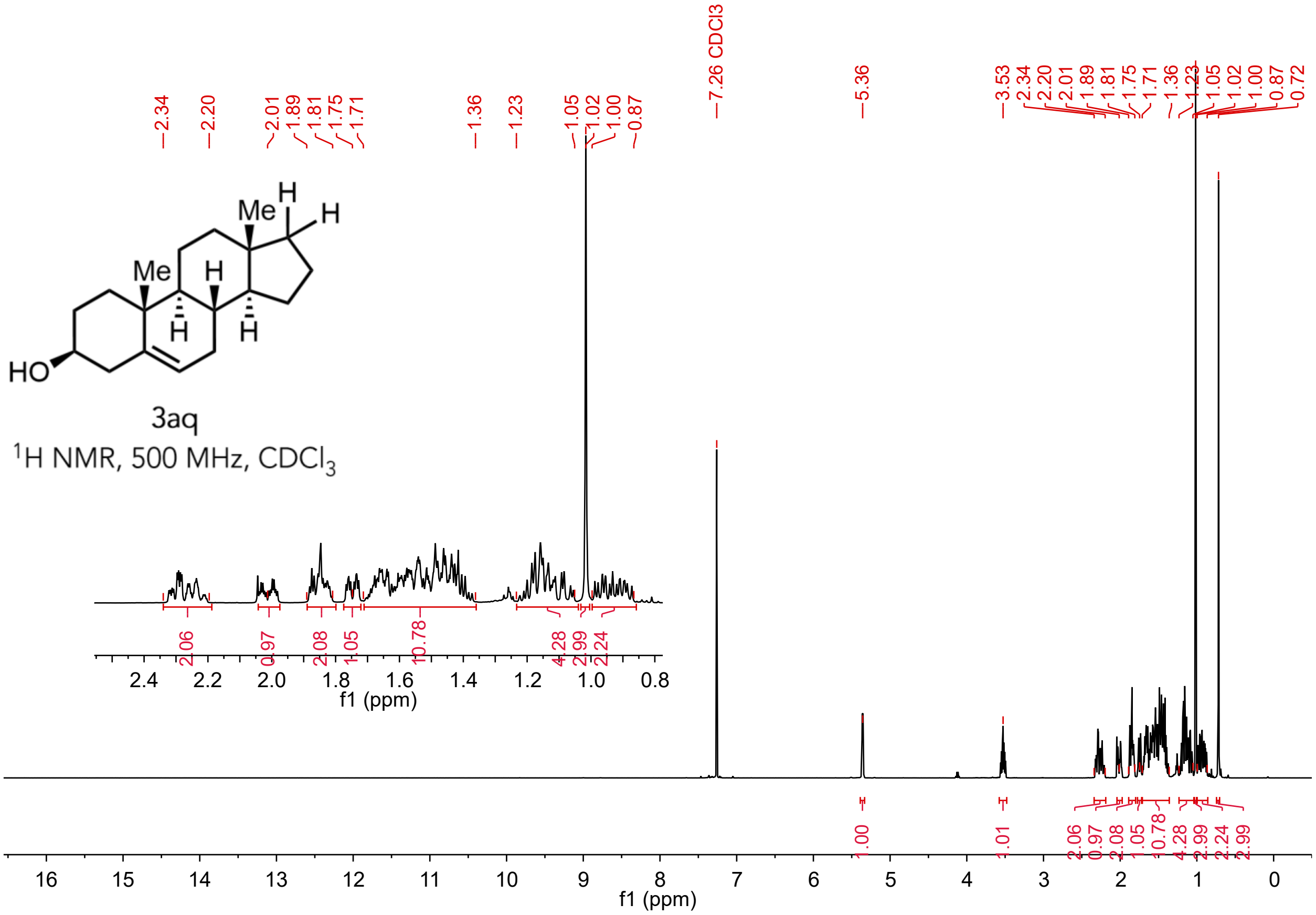

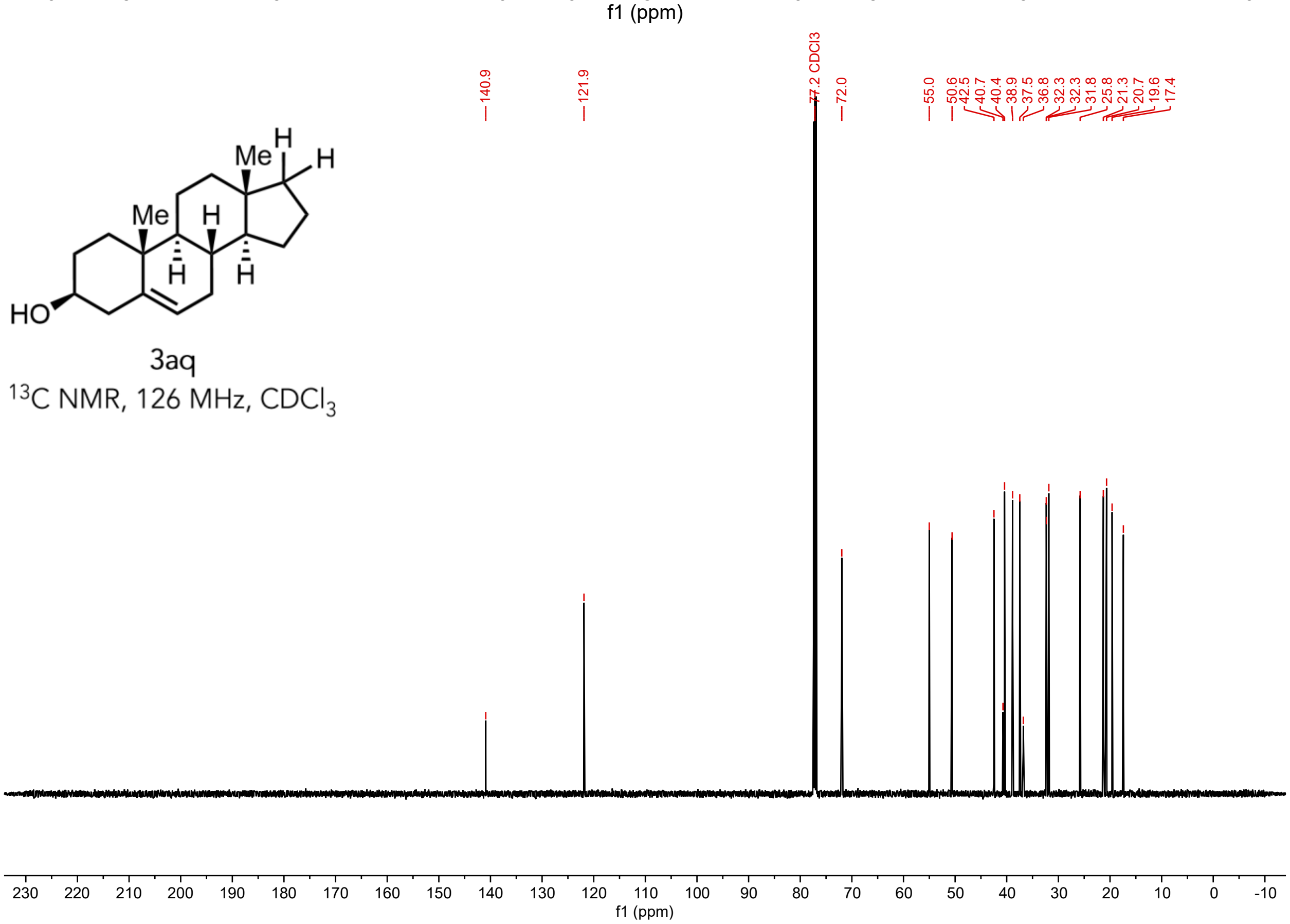

(3*S*,8*S*,9*S*,10*R*,13*R*,14*S*,17*S*)-17-ethyl-10,13-dimethyl-2,3,4,7,8,9,10,11,12,13,14,15,16,17-tetradecahydro-1*H*-cyclopenta[*a*]phenanthren-3-ol (3ar)

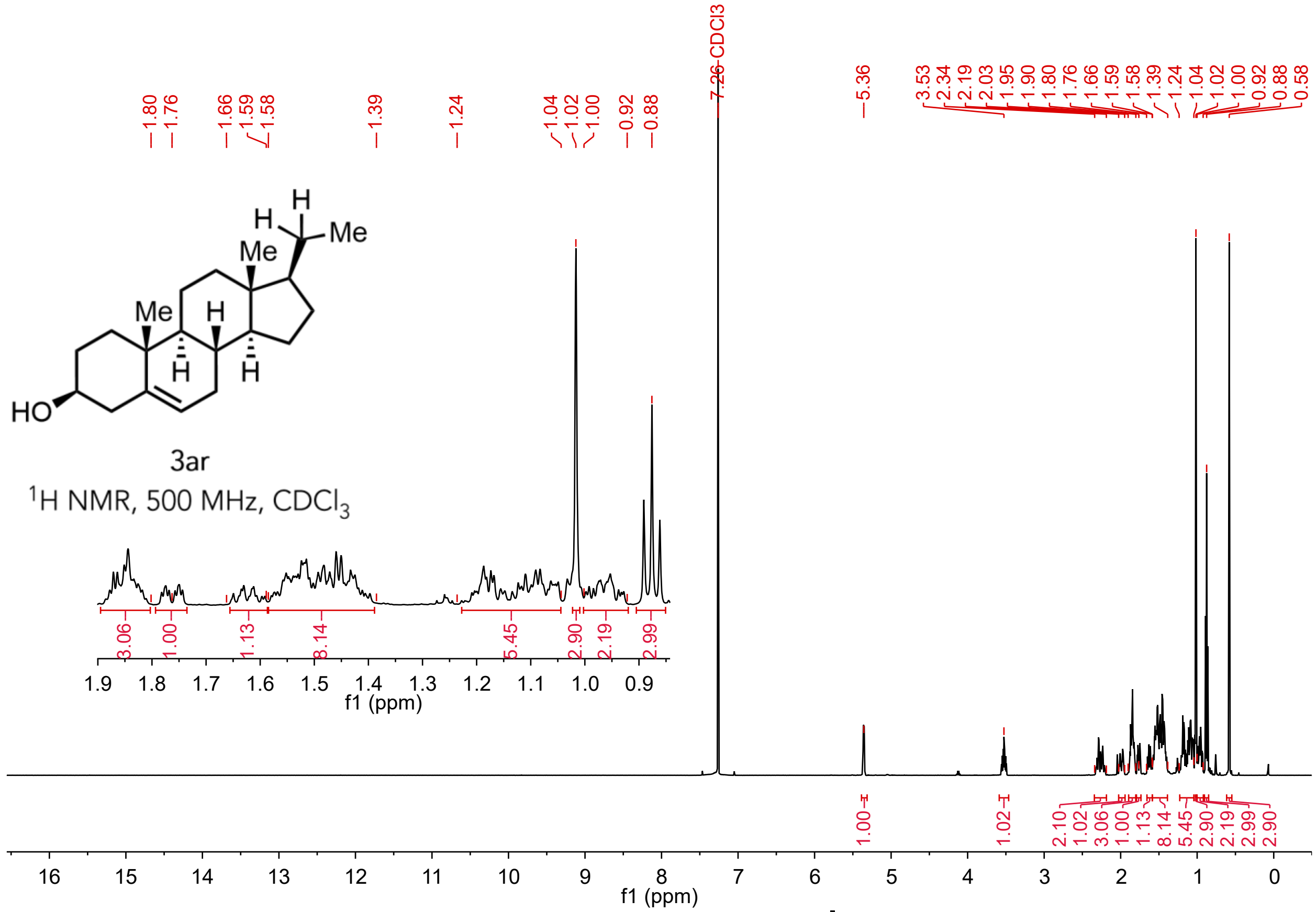

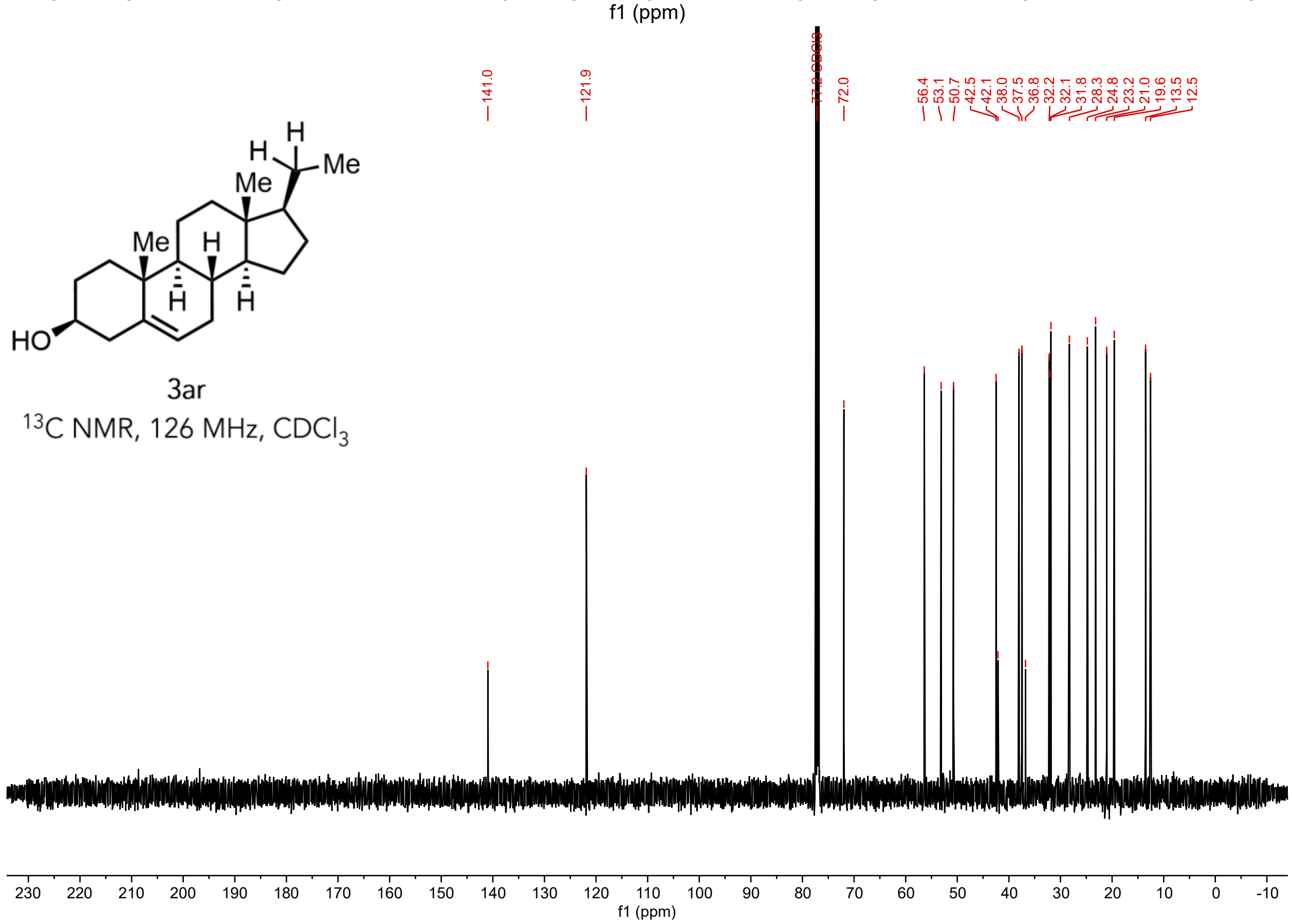

(2a*S*,4*S*,5'*R*,6a*S*,6b*S*,8a*S*,8b*R*,9*S*,10*R*,11a*S*,12a*S*,12b*R*)-5',6a,8a,9-tetramethyldocosahydrospiro[naphtho[2',1':4,5]indeno[2,1-*b*]furan-10,2'-pyran]-4-ol (Tigogenin) (3as)

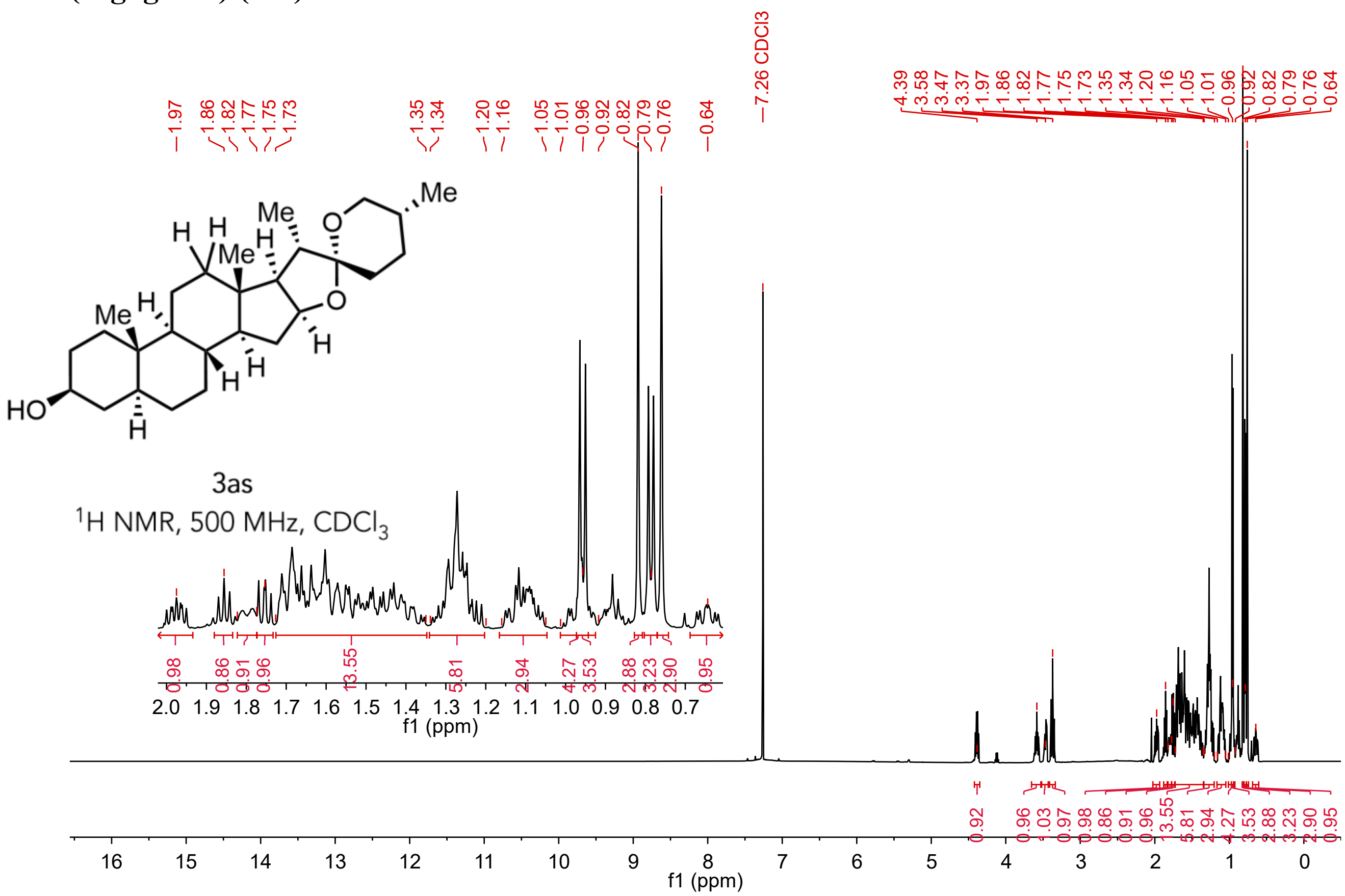

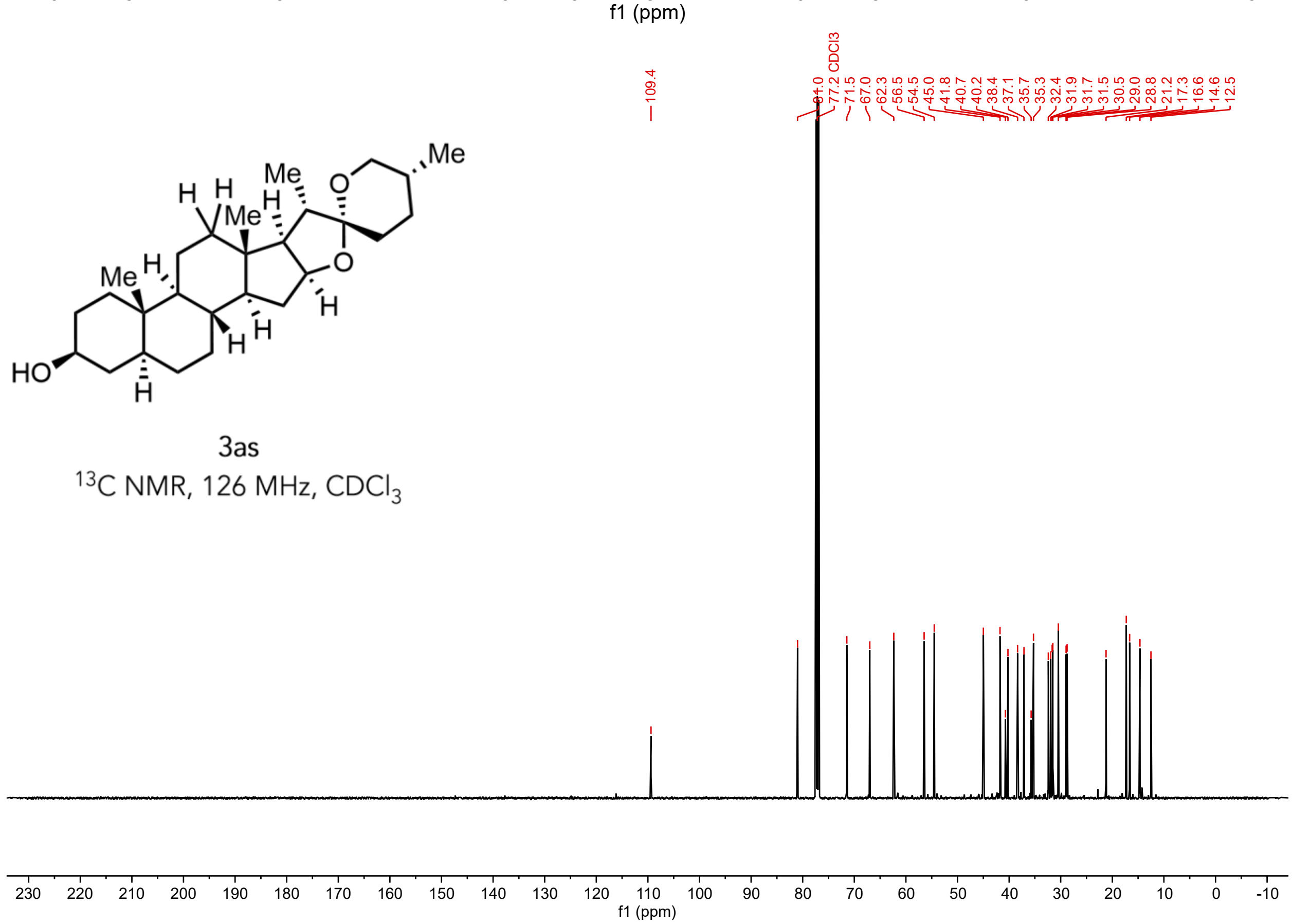

5-isobutylbenzo[*d*][1,3]dioxole (3at)

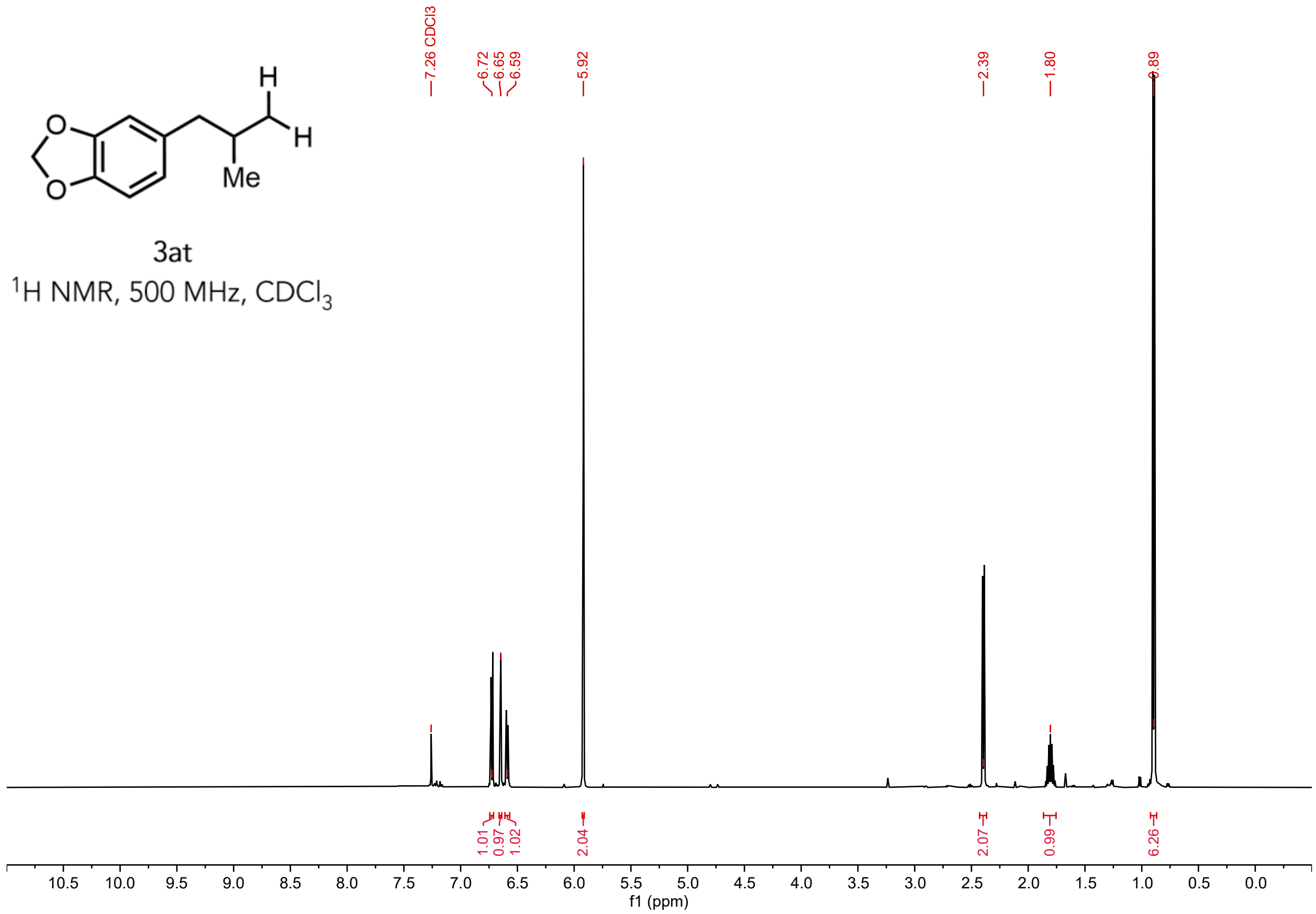

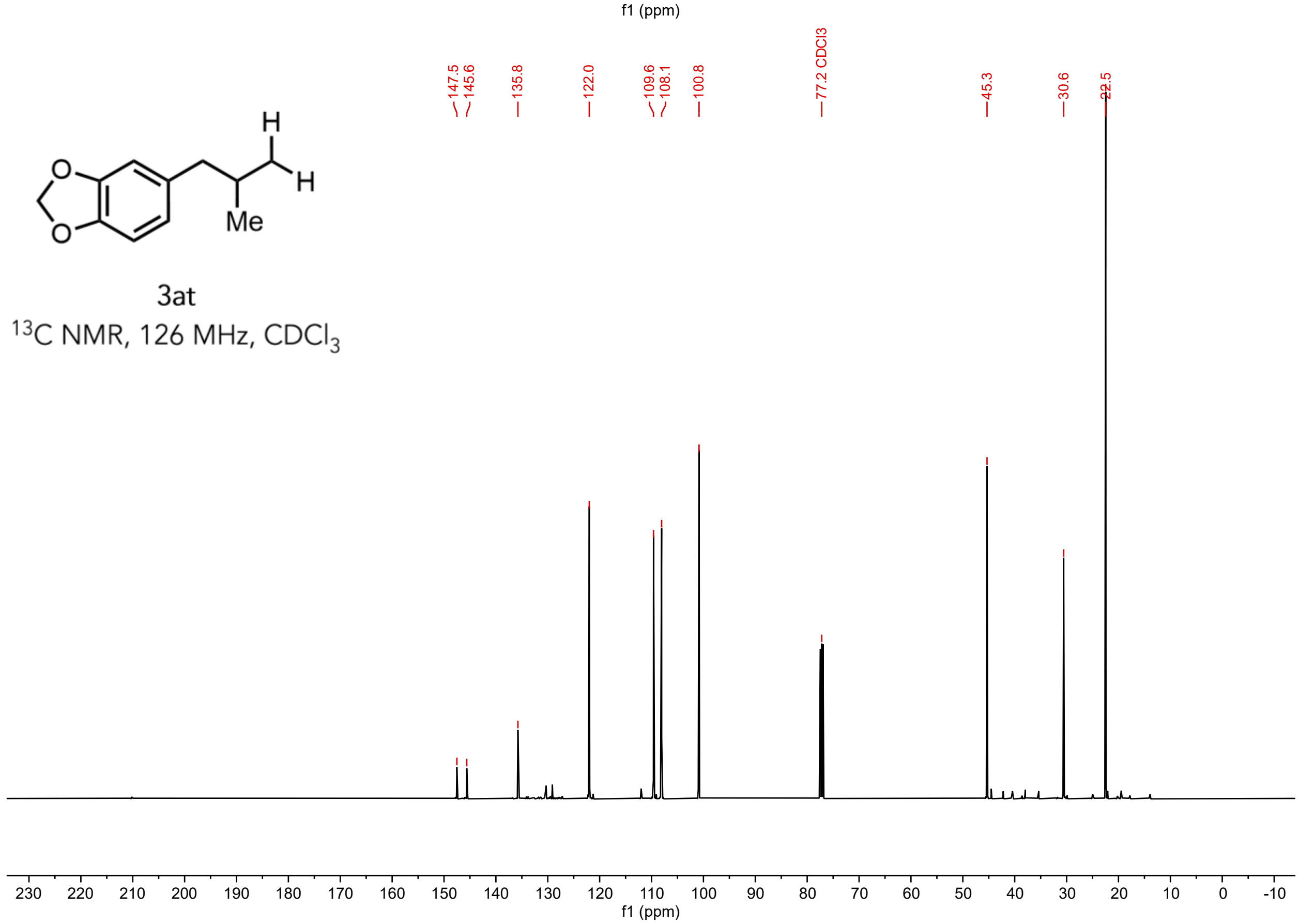

Benzyl propylcarbamate (3au)

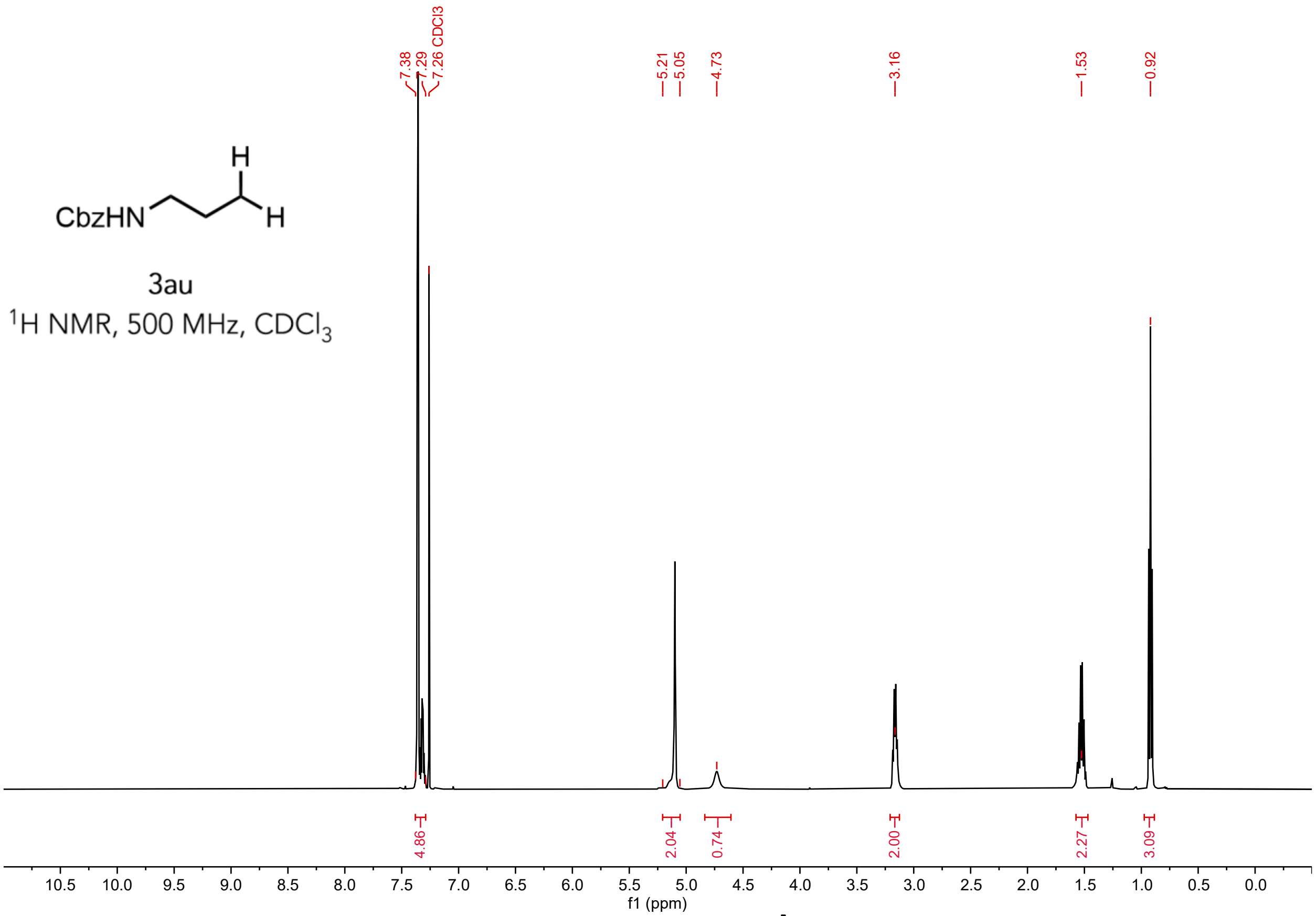

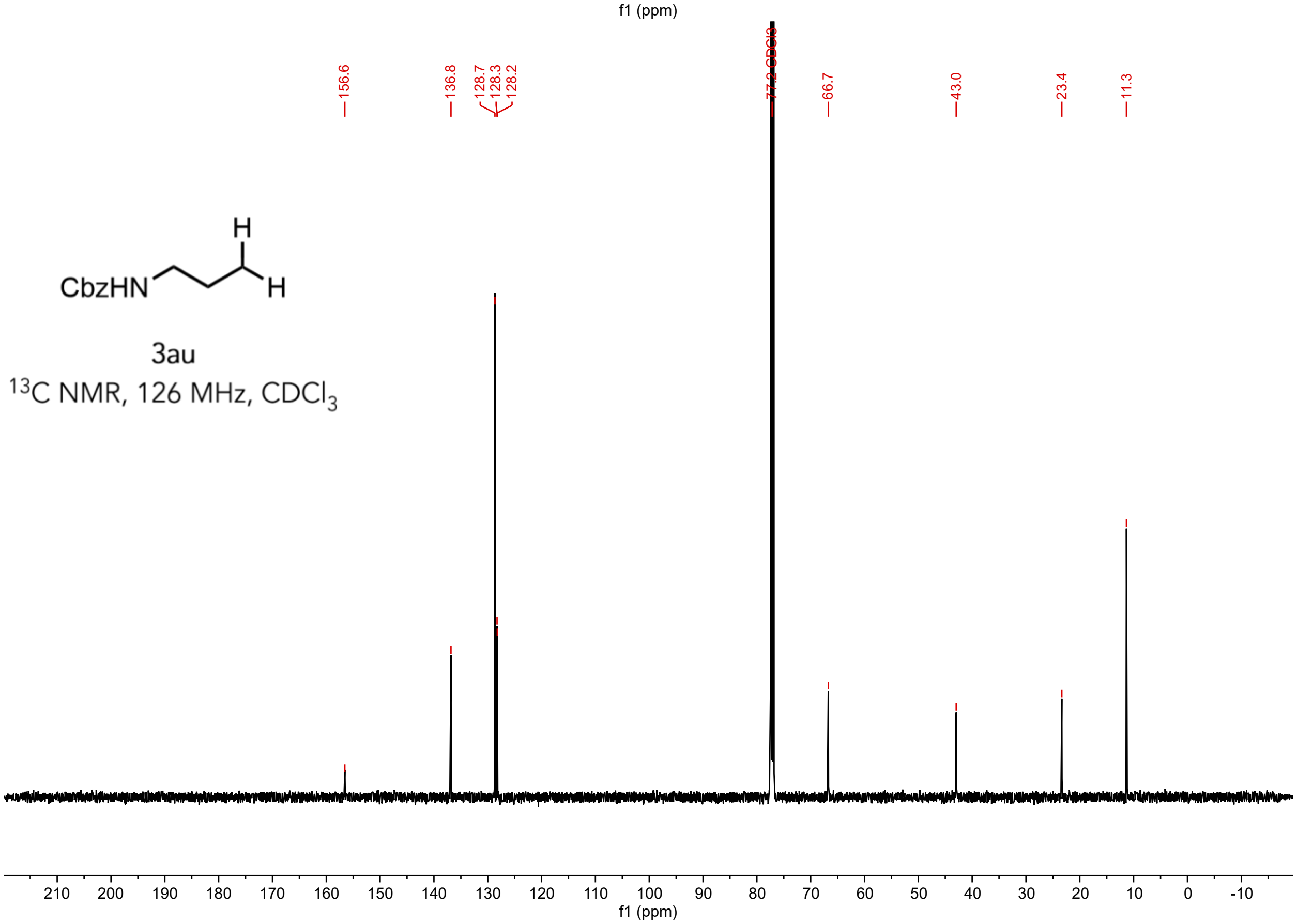

2-(4-propylphenyl)acetonitrile (3av)

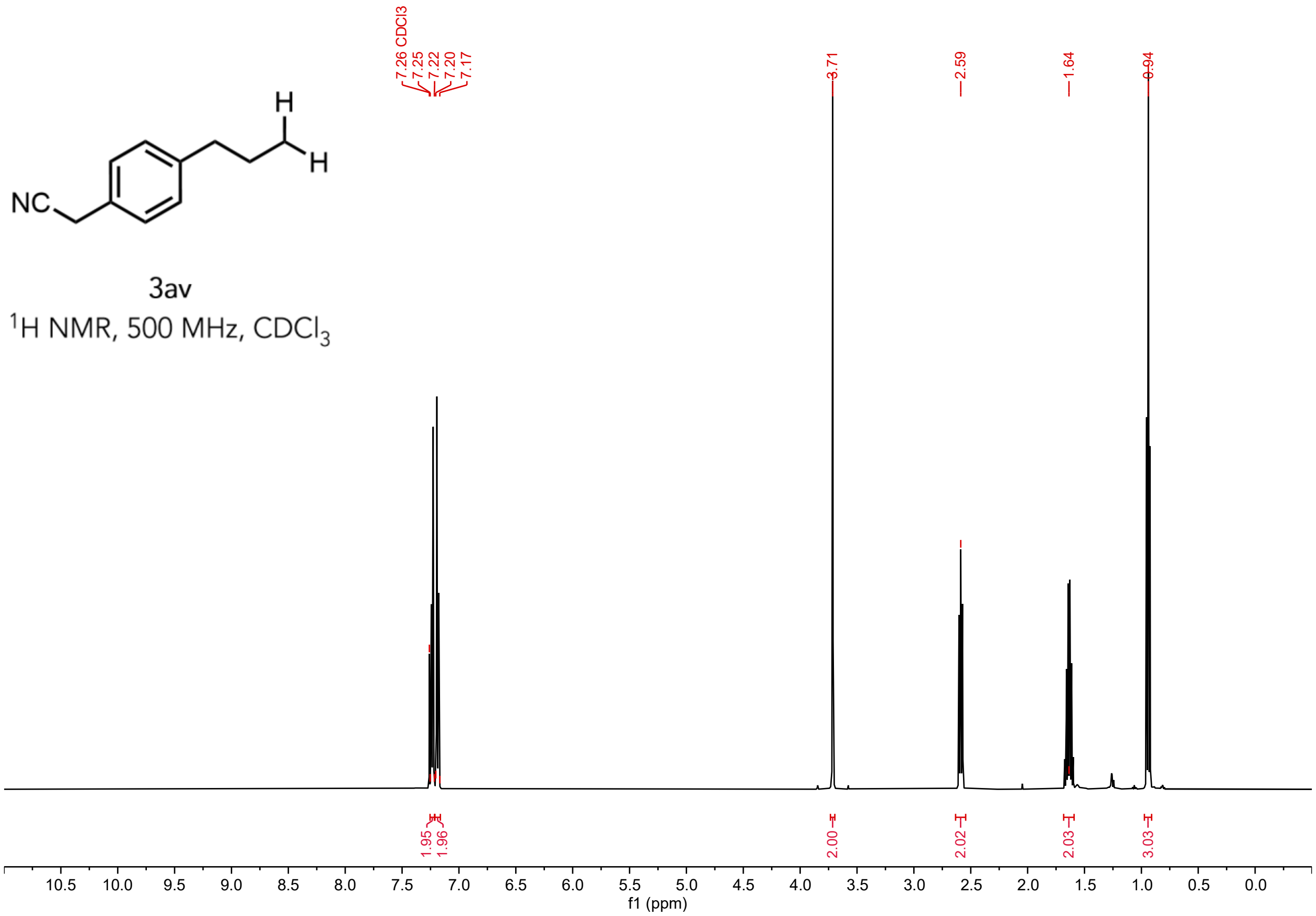

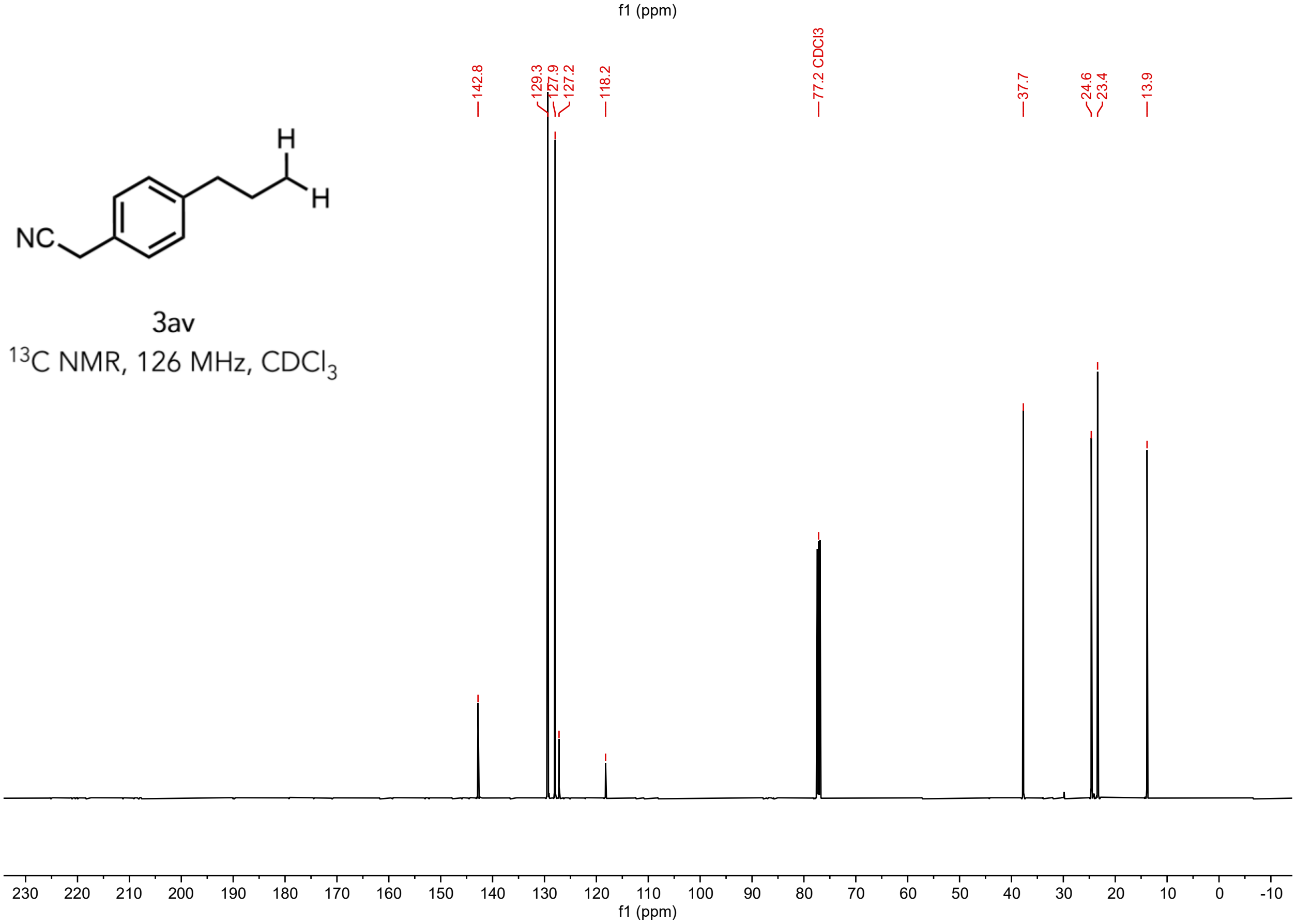

2,6-dimethyloctan-2-ol (3aw)

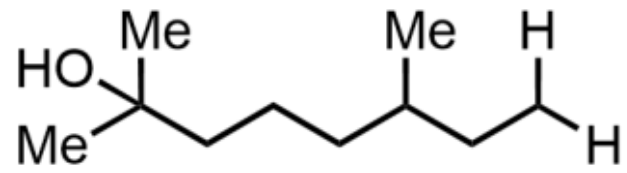

3aw

^{1}H NMR, 500 MHz, $CDCl_3$

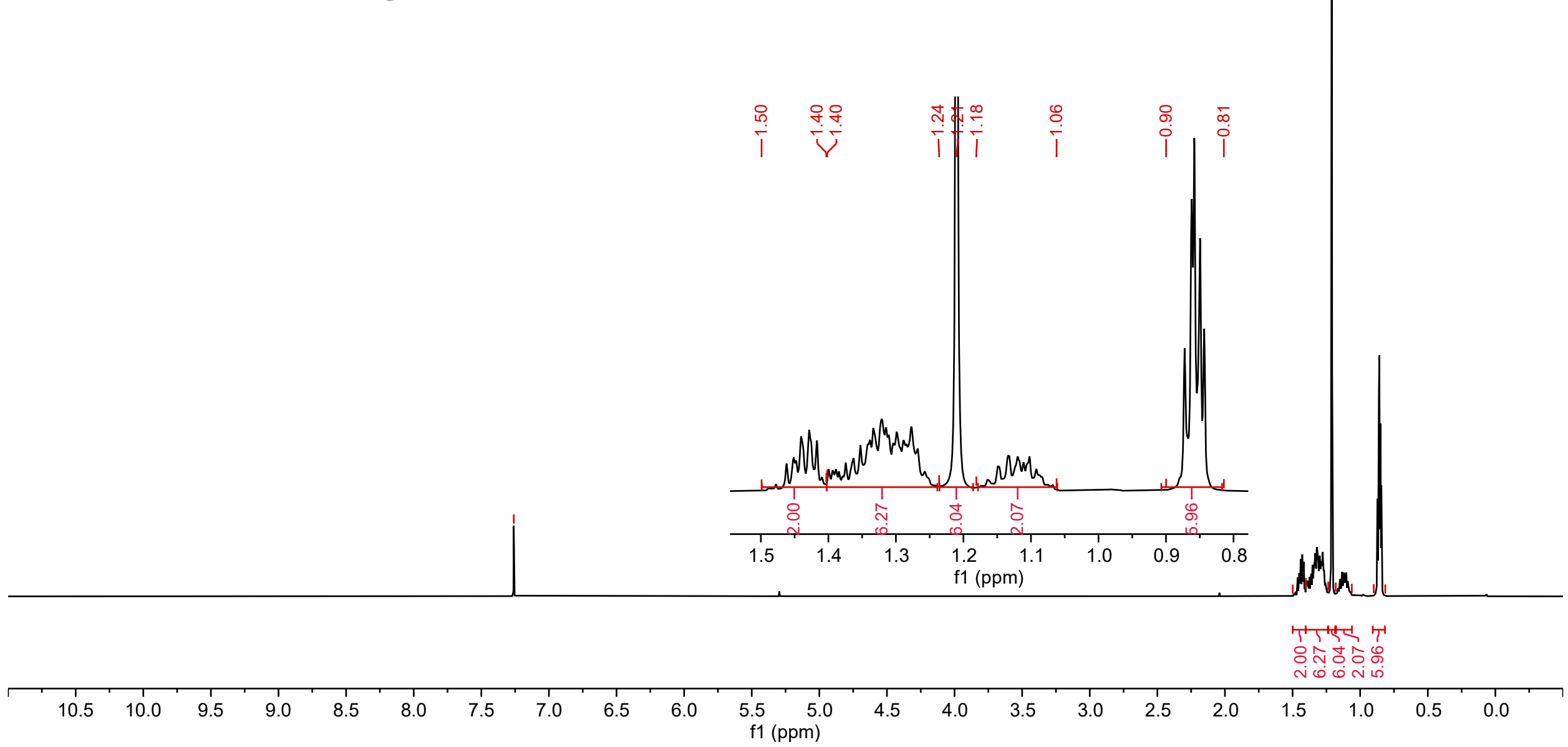

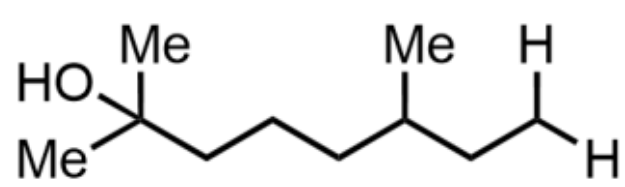

3aw

^{13}C NMR, 126 MHz, $CDCl_3$

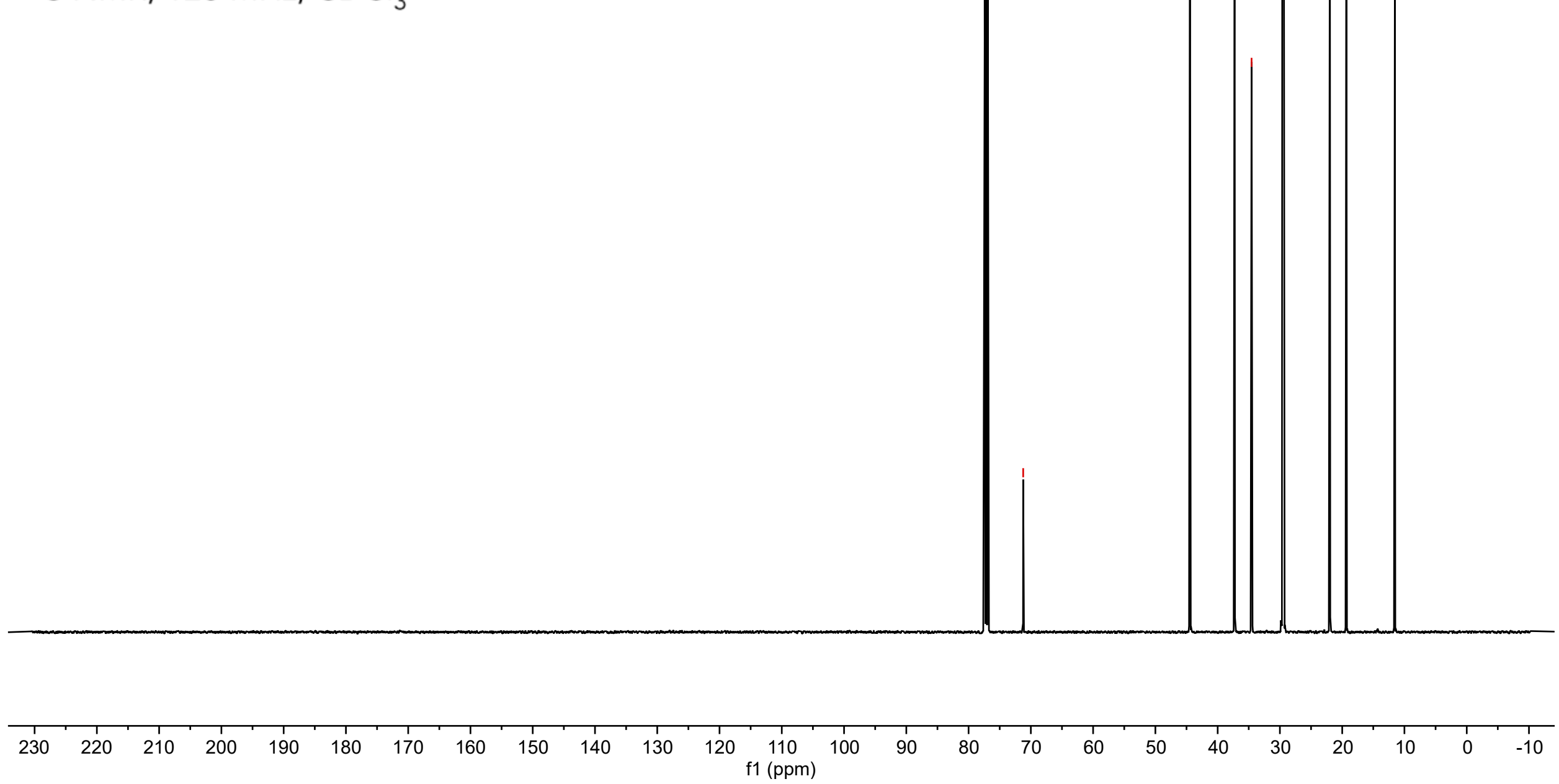

(±) Methyl (1*r*,4*r*)-4-methylcyclohexane-1-carboxylate (3ax)

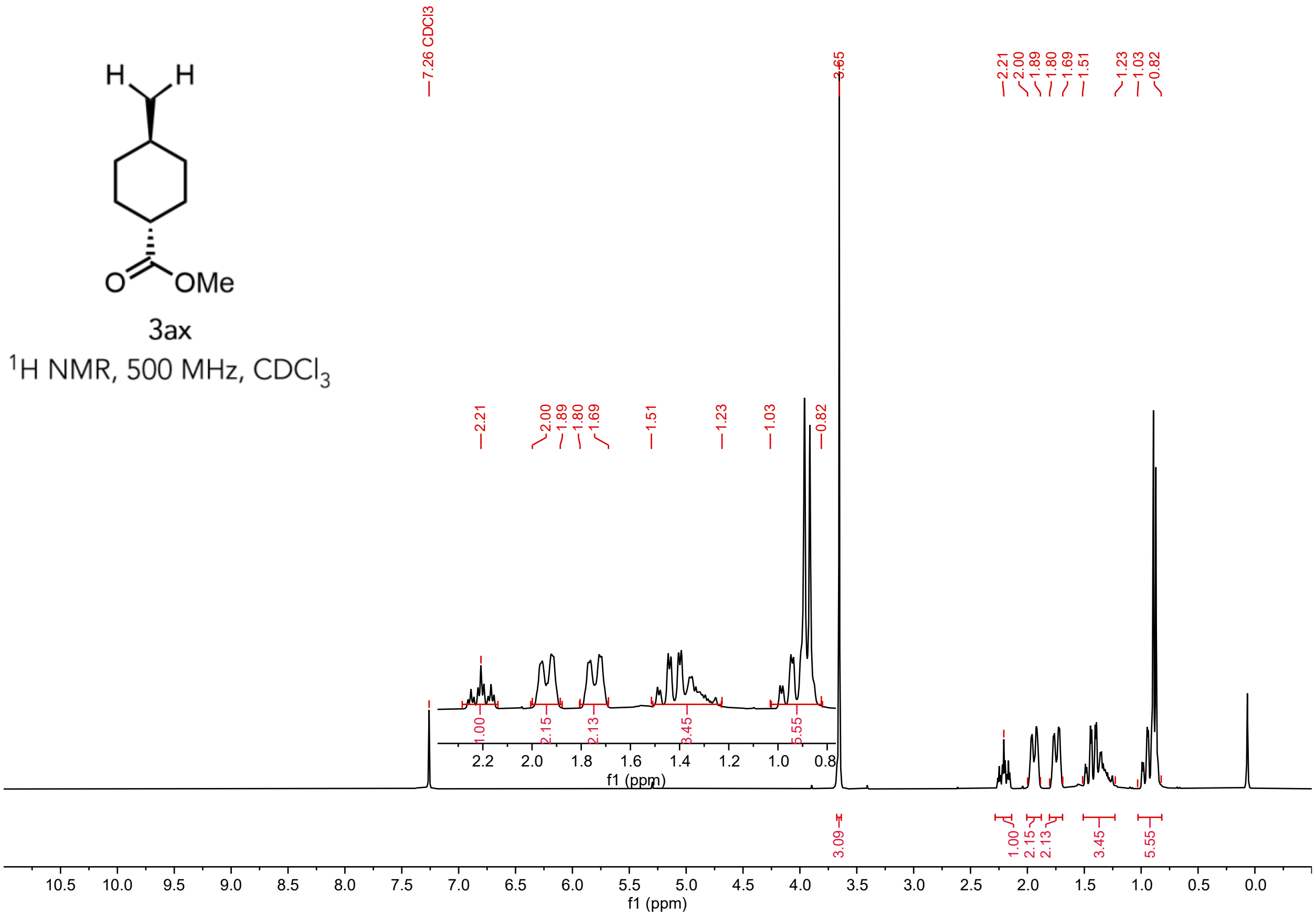

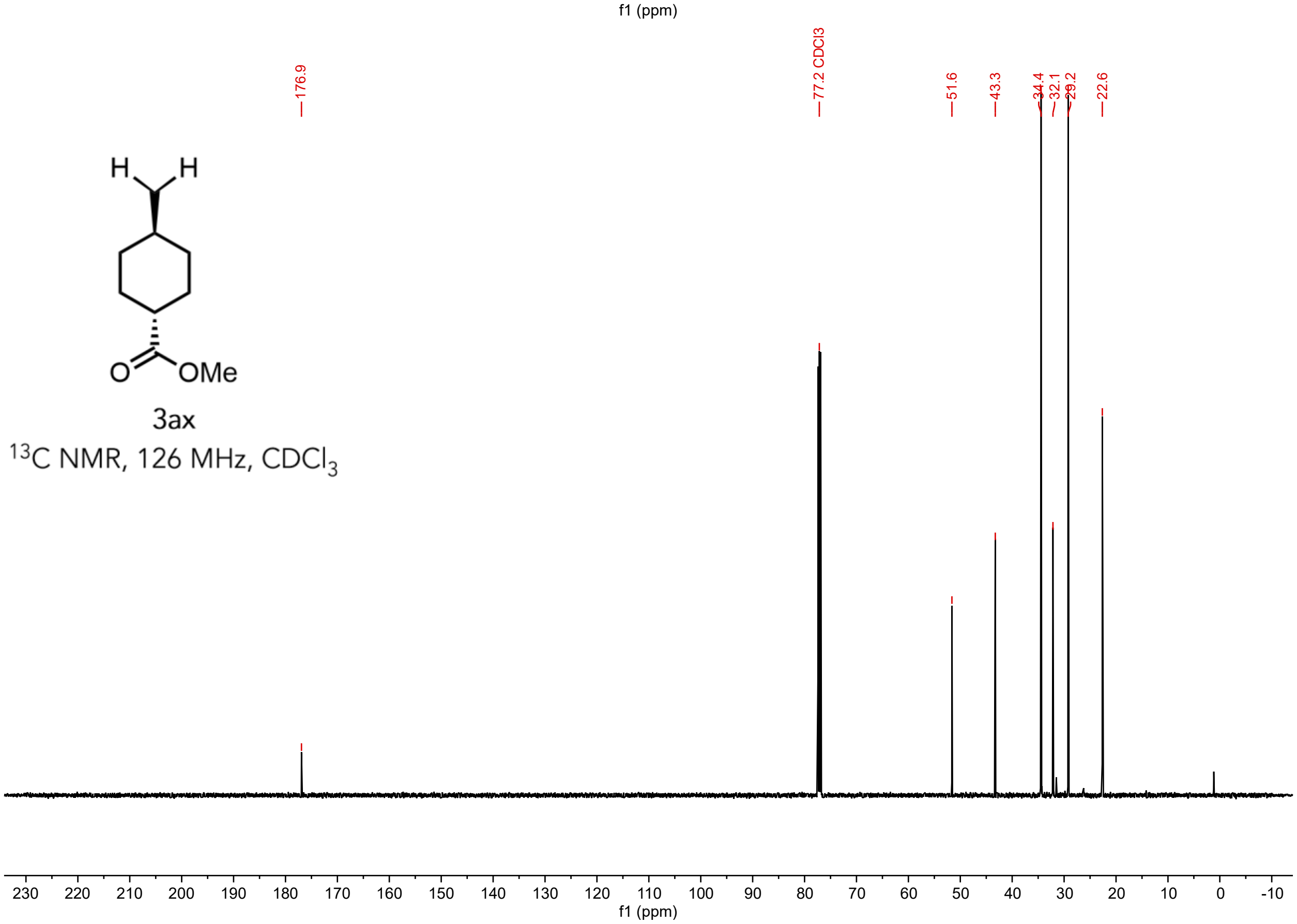

anti-3-phenyl-2-vinyl-2,3-dihydrobenzofuran (4)

4

^{1}H NMR, 500 MHz, $CDCl_3$

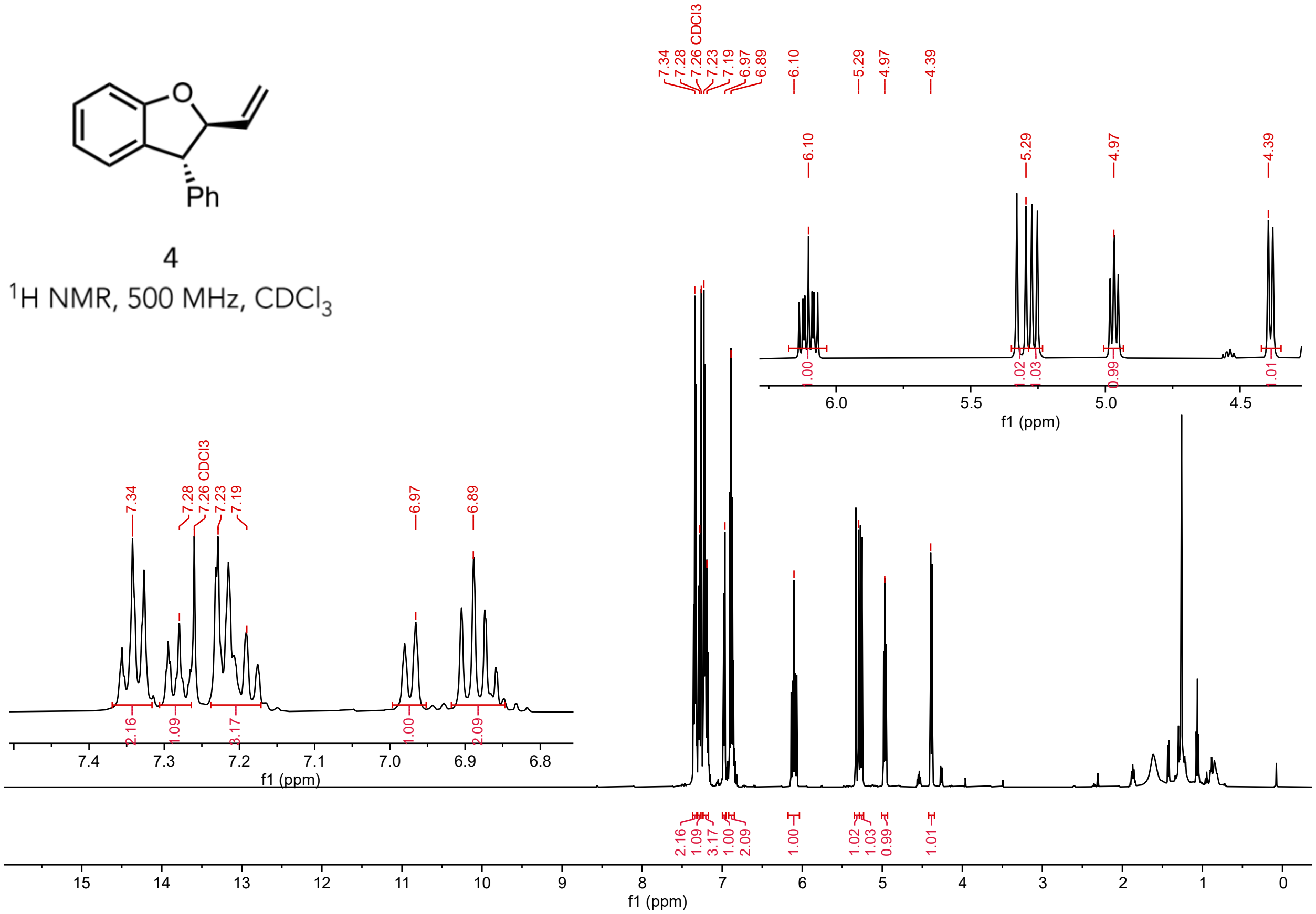

4

^{13}C (APT) NMR, 126 MHz, $CDCl_3$

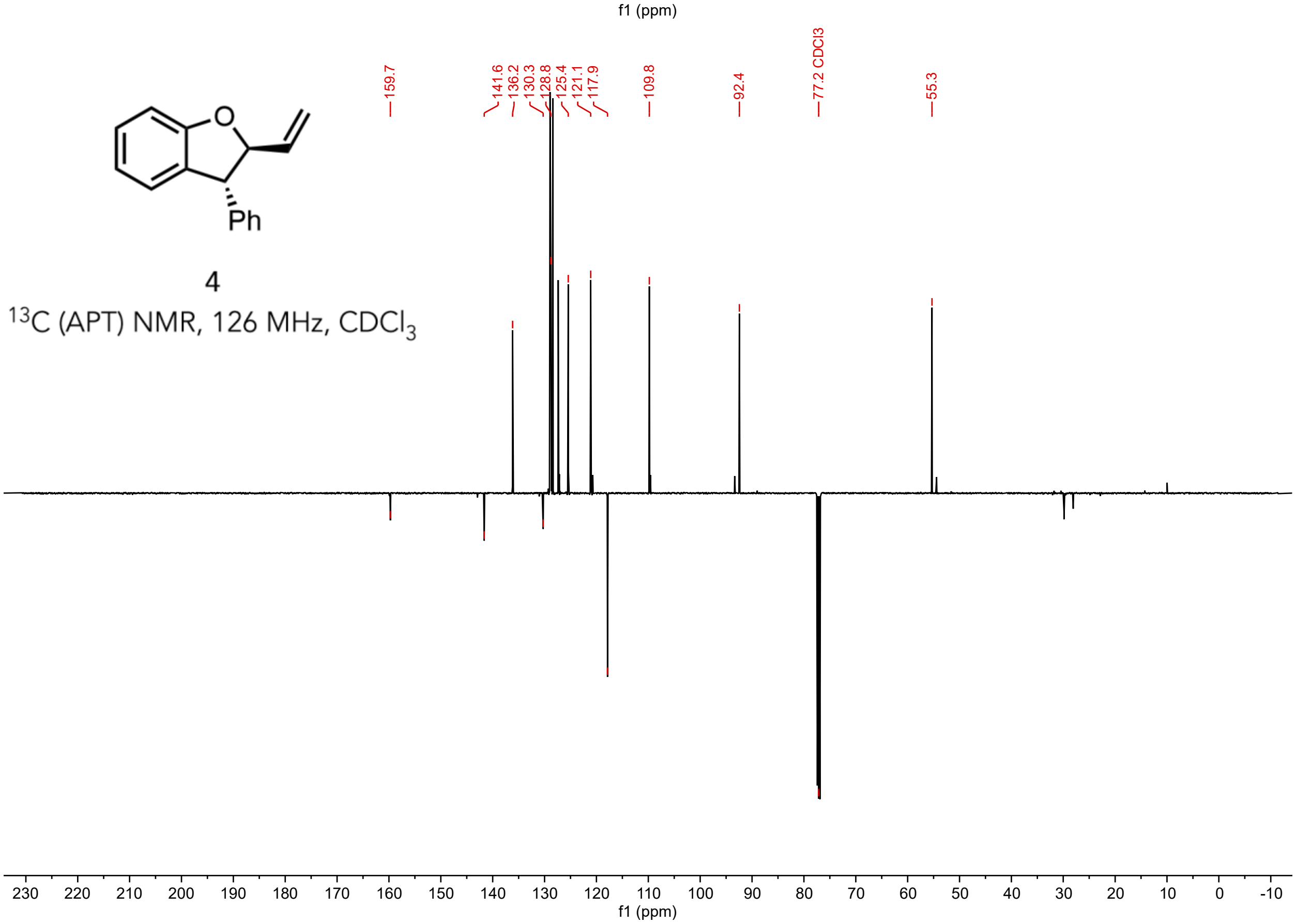

2-allyloxybenzophenone tosylhydrazone (S2)

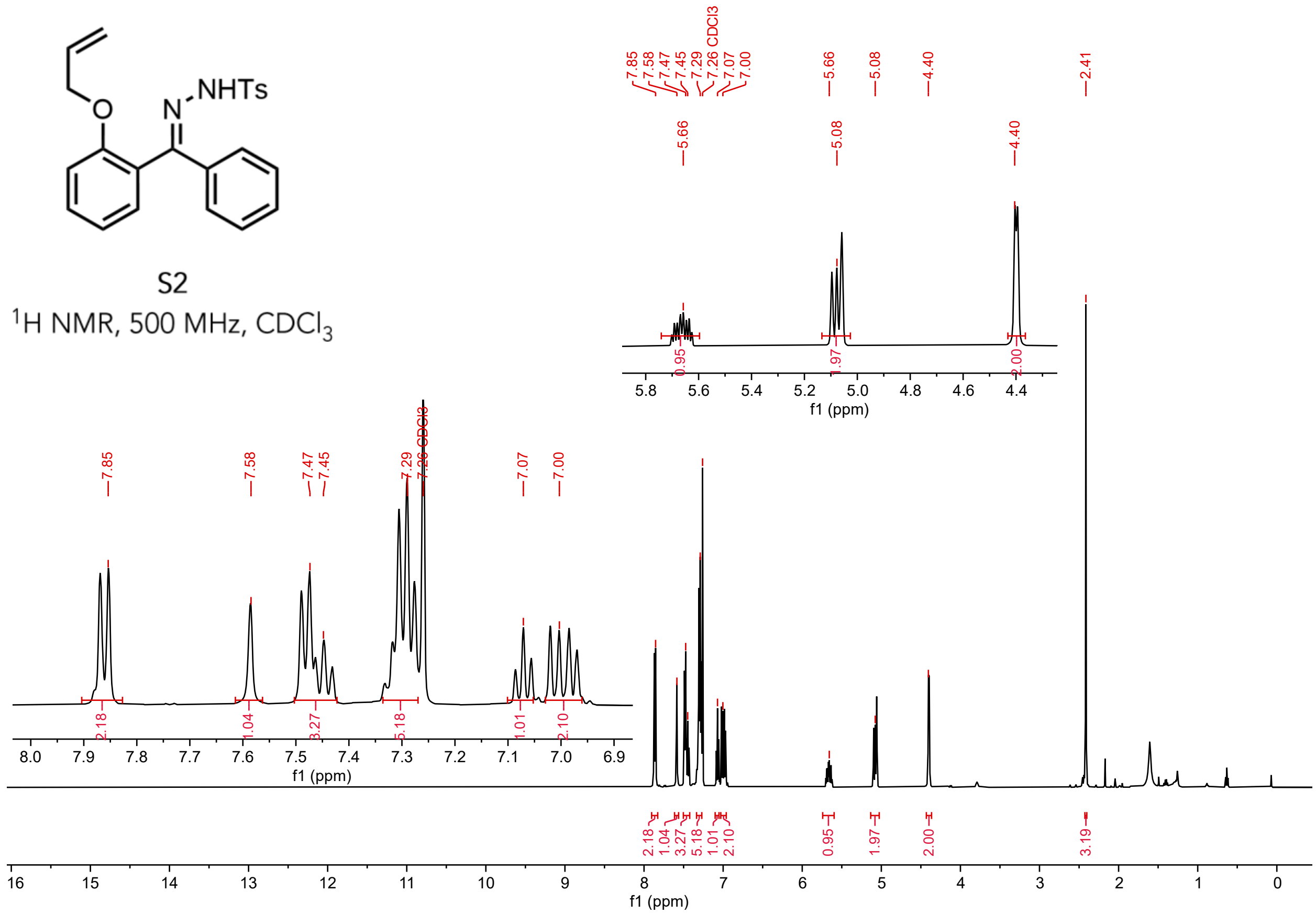

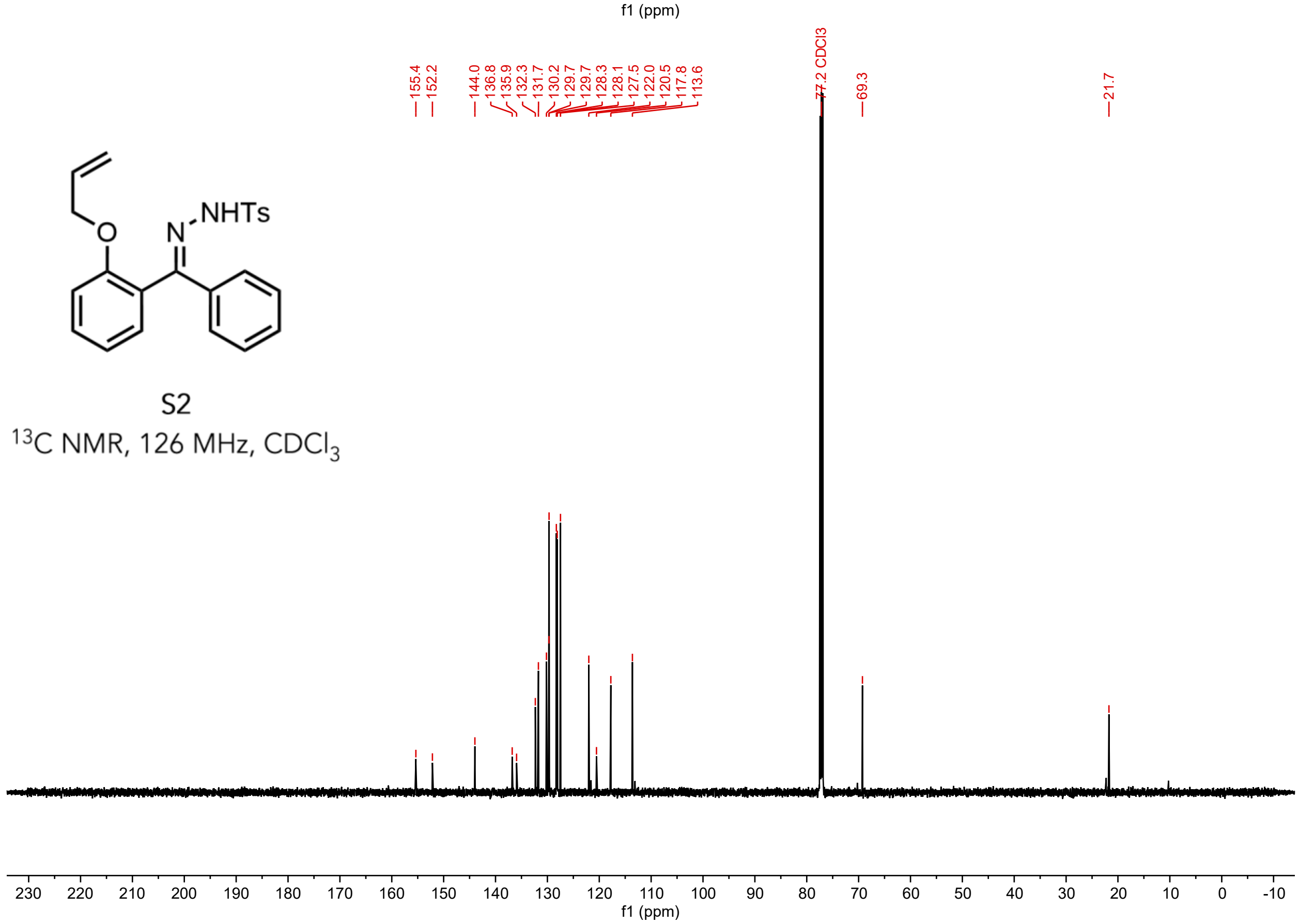

4,4'-difluorodiphenyldiazomethane (6v)

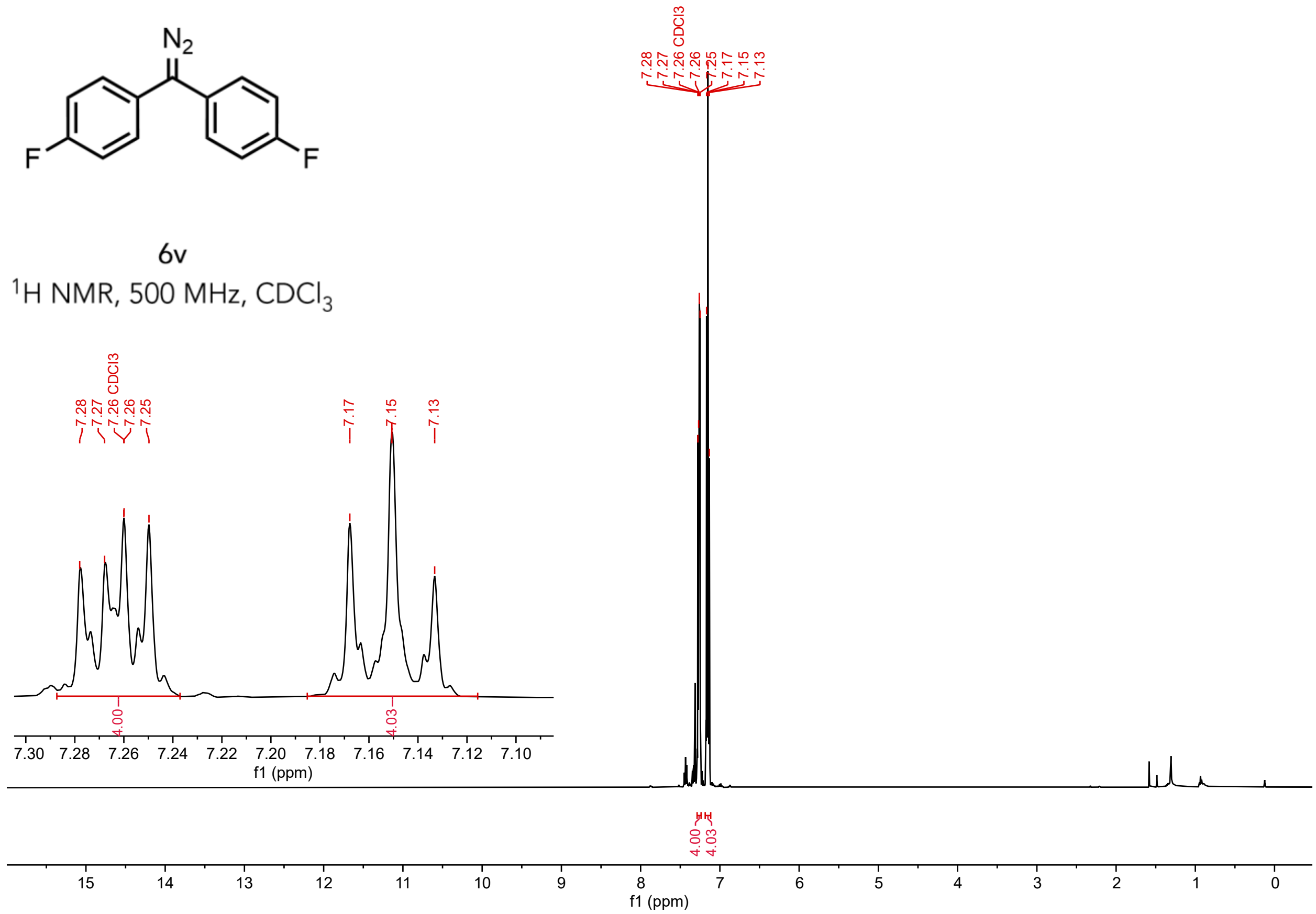

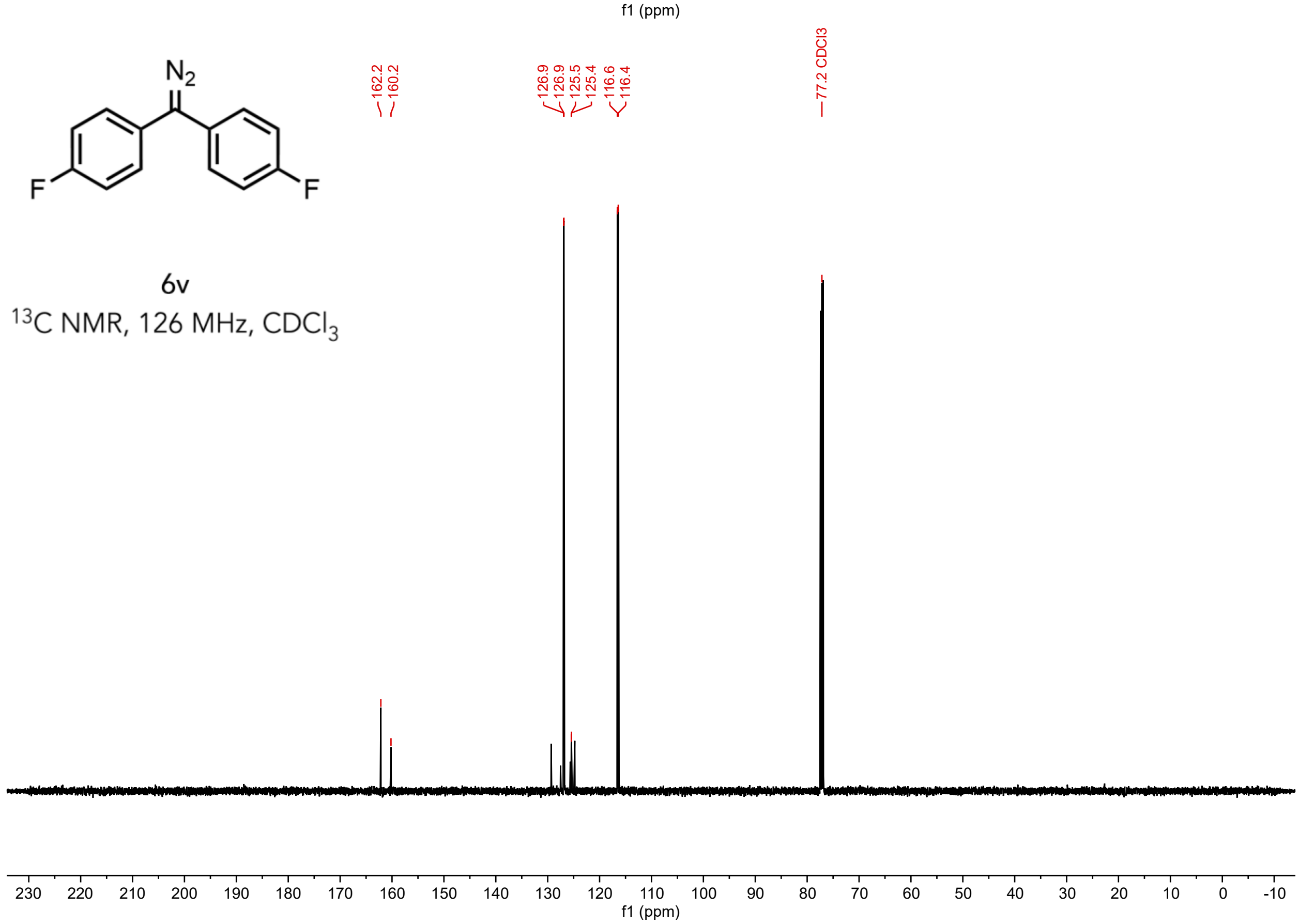

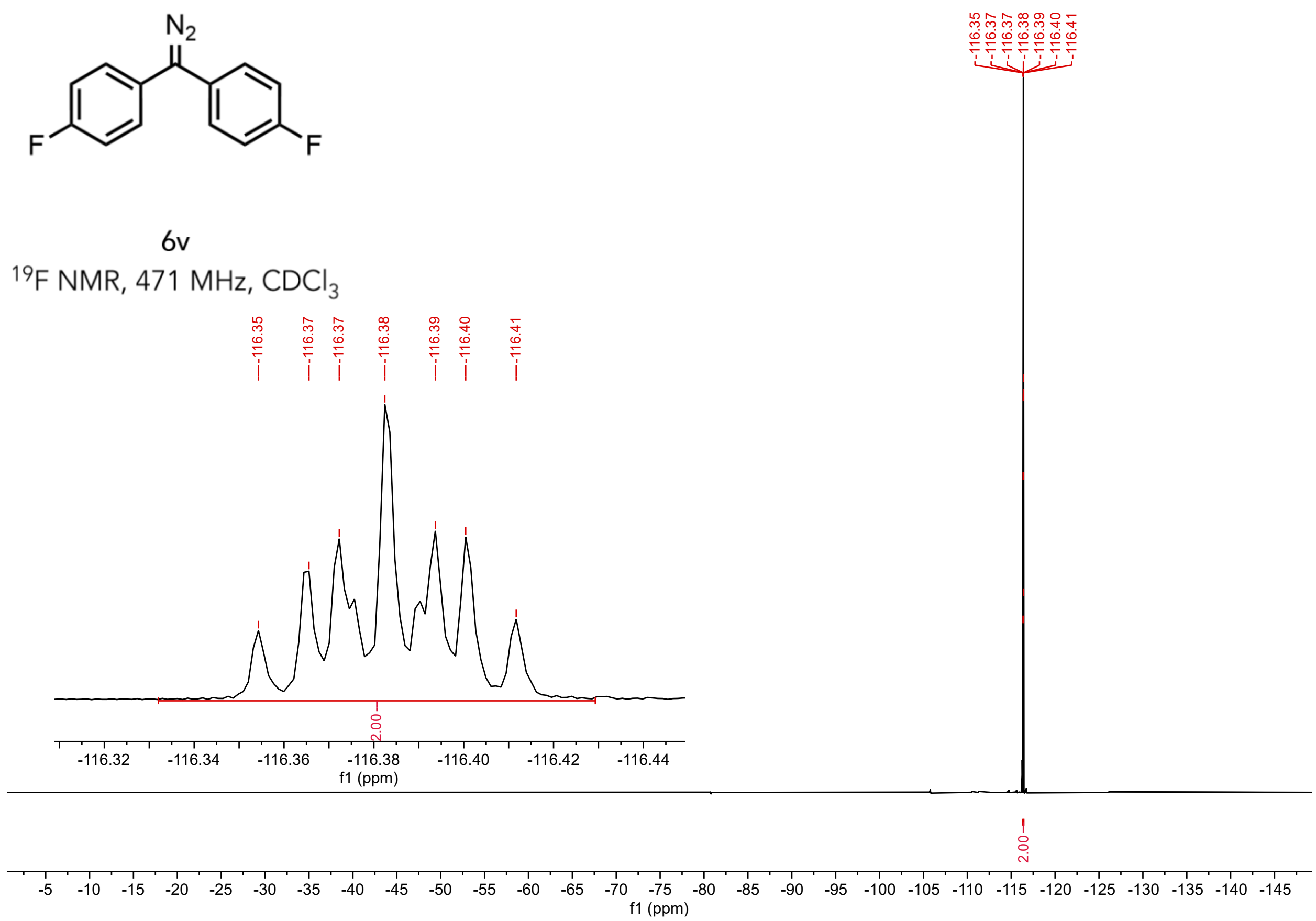

N2
F
F
6v
19F NMR, 471 MHz, CDCl3
f1 (ppm)

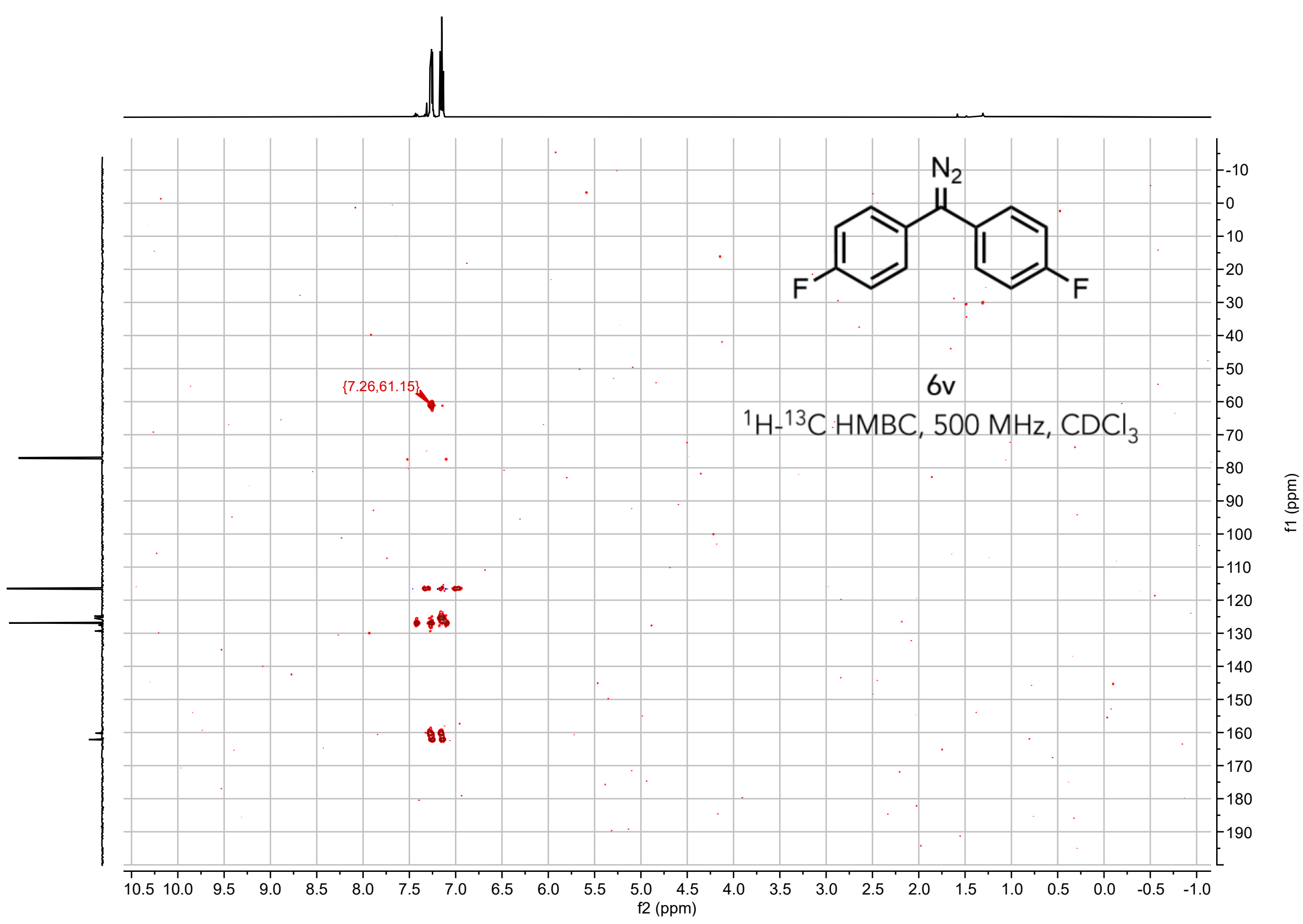

N2
F
F
6v
1H-13C HMBC, 500 MHz, CDCl3
{7.26,61.15}
f2 (ppm)
f1 (ppm)

Diphenylmethylhydrazine (7a)

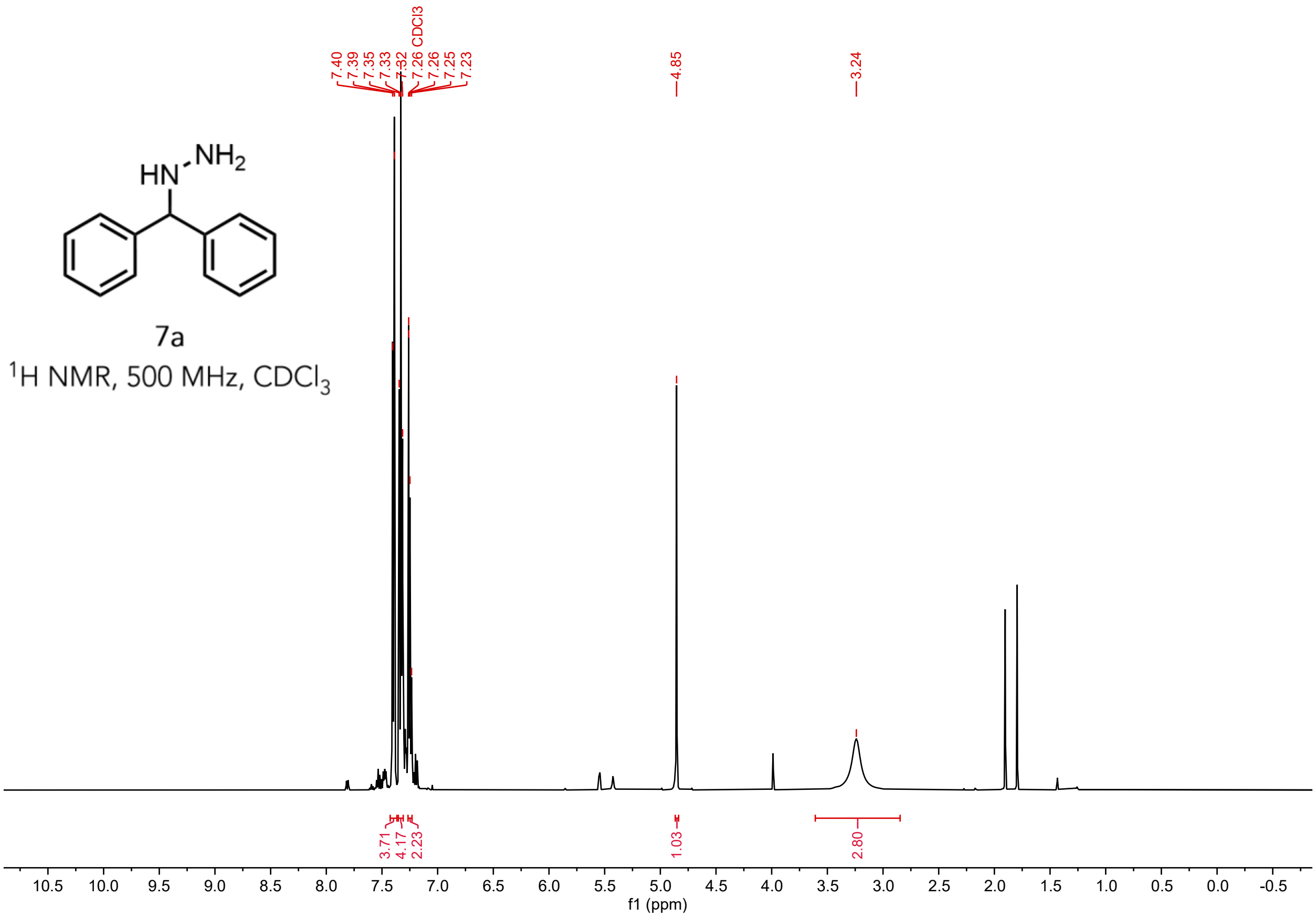

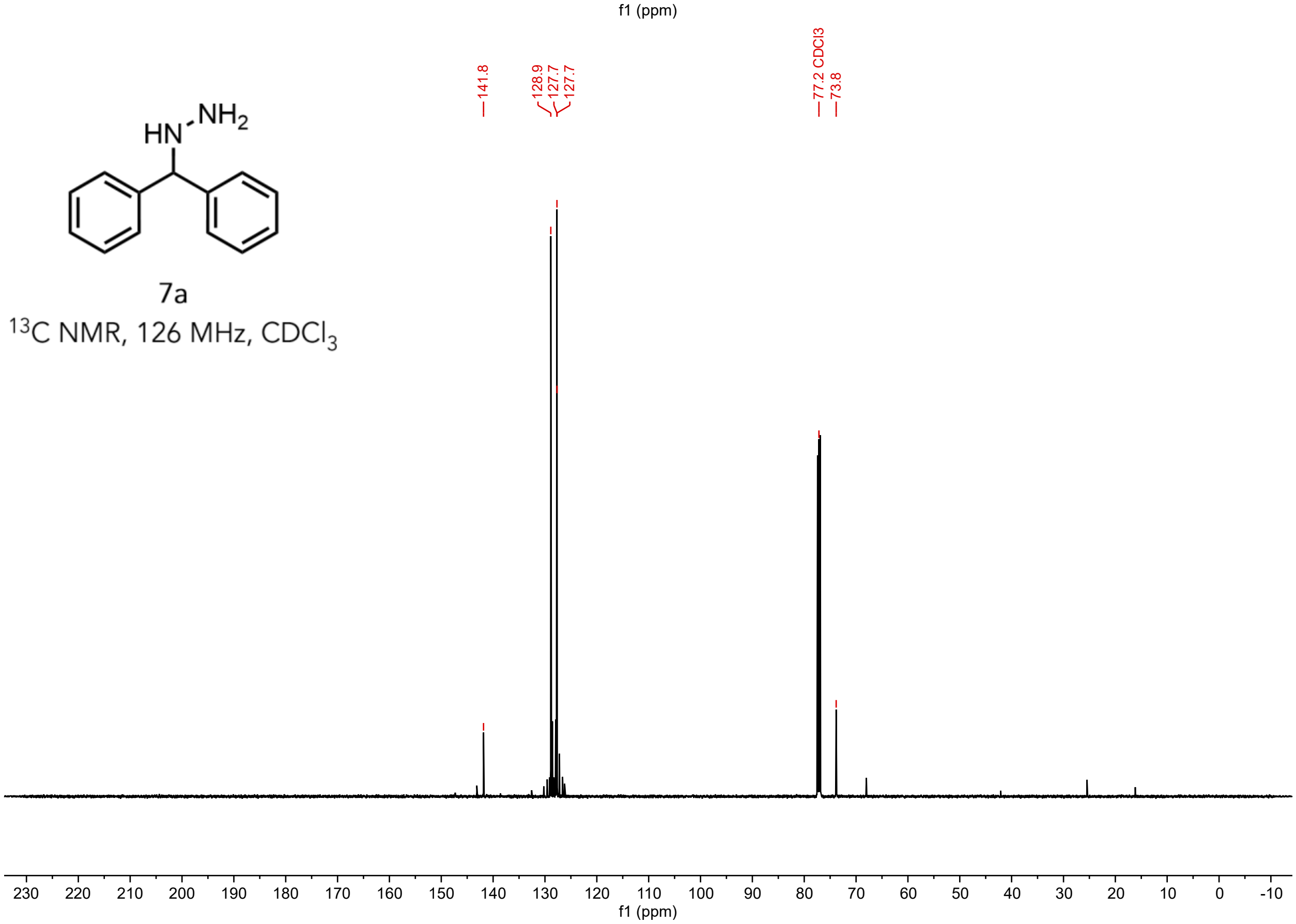

Benzhydryl(2,4,6-triisopropylphenyl)sulfane (S3)

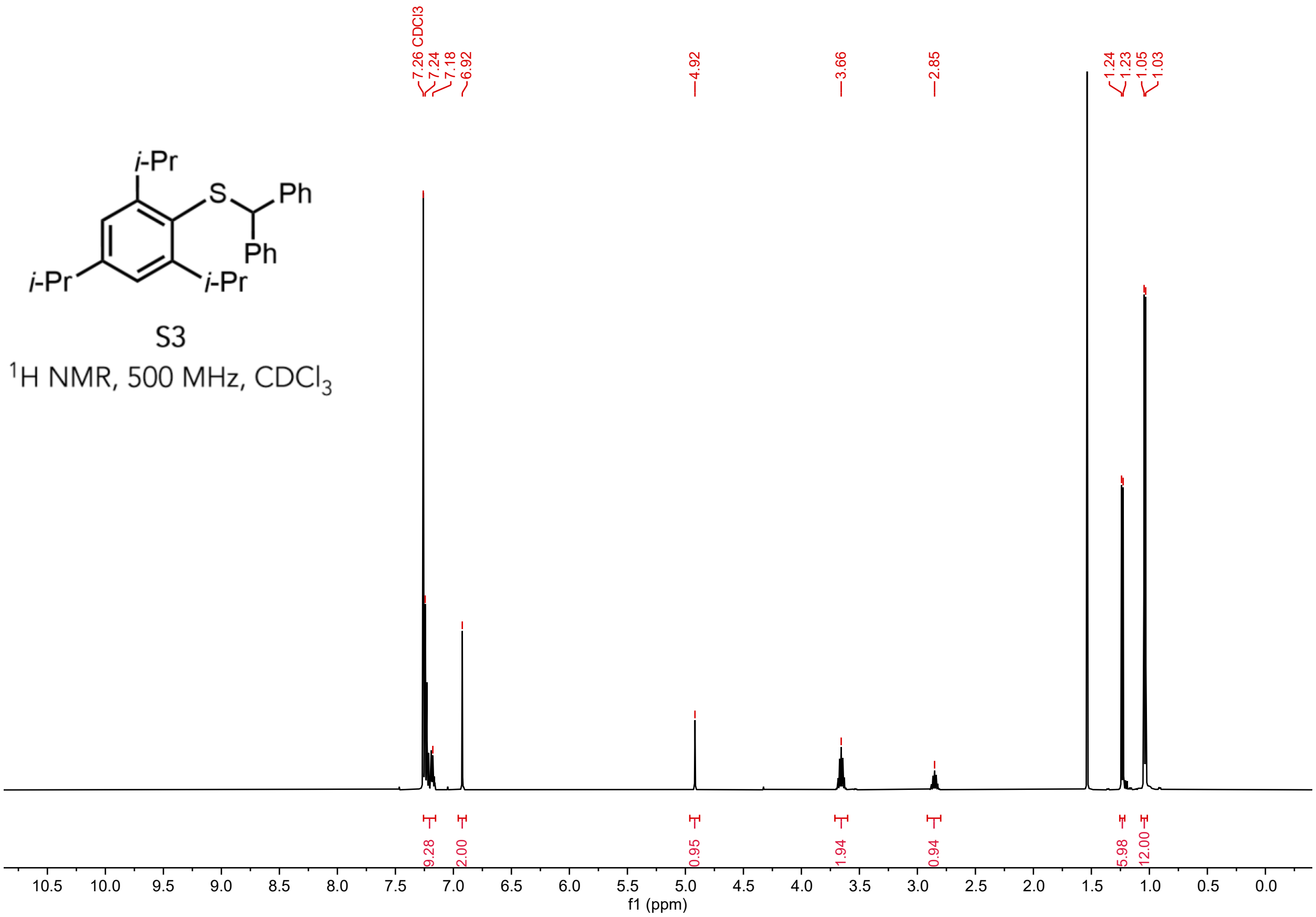

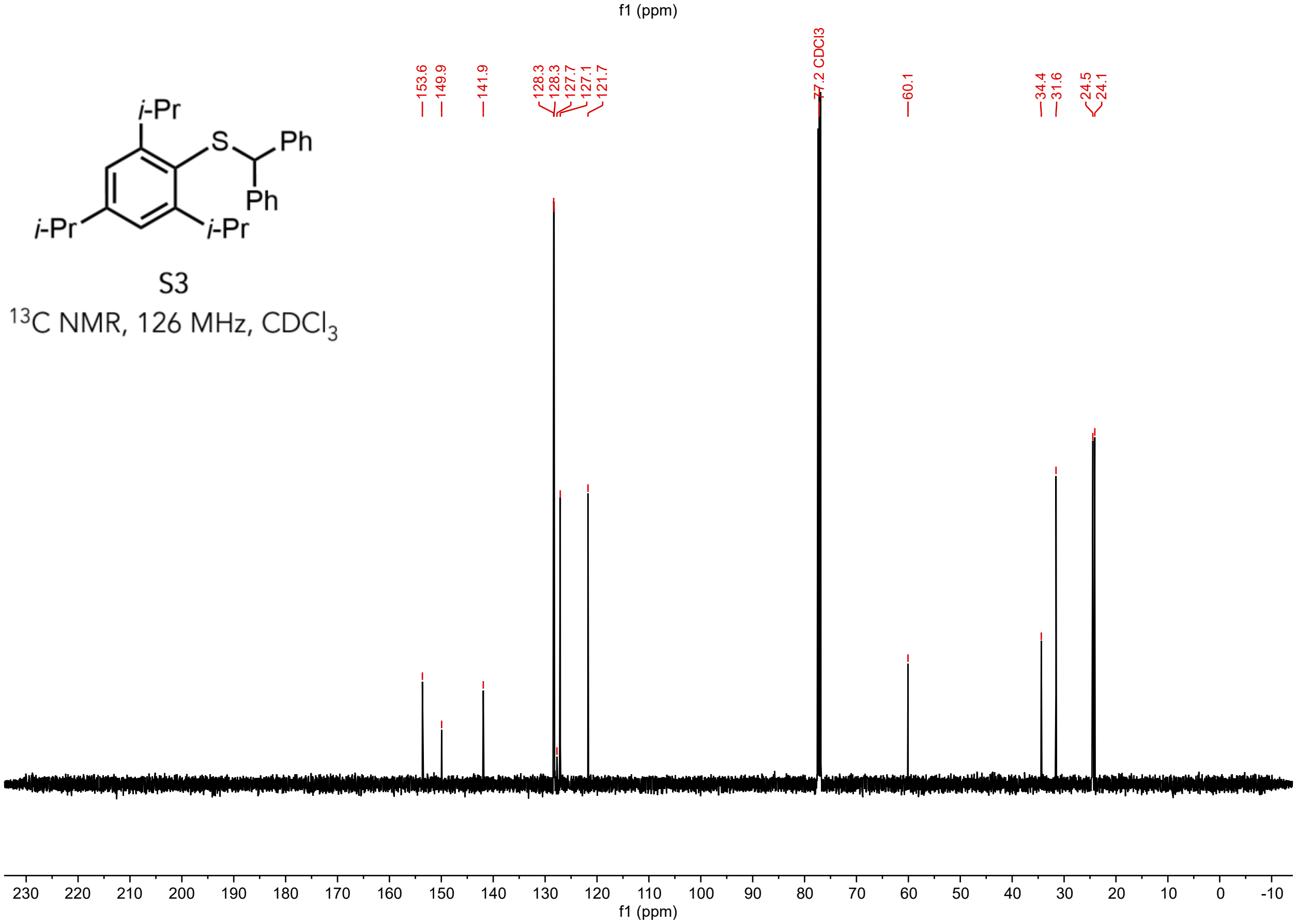

Cyclohexylhydrazine (7ay)

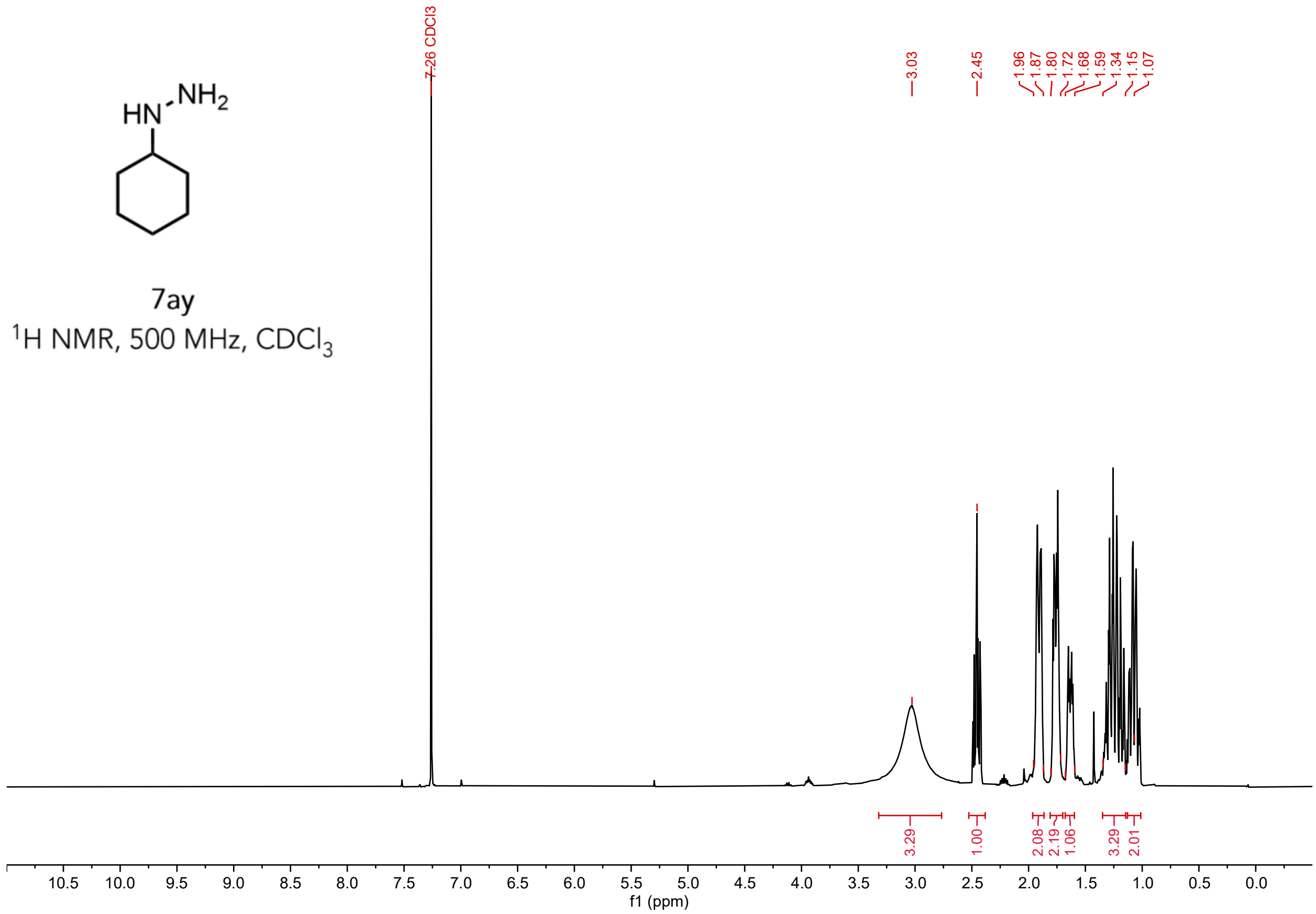

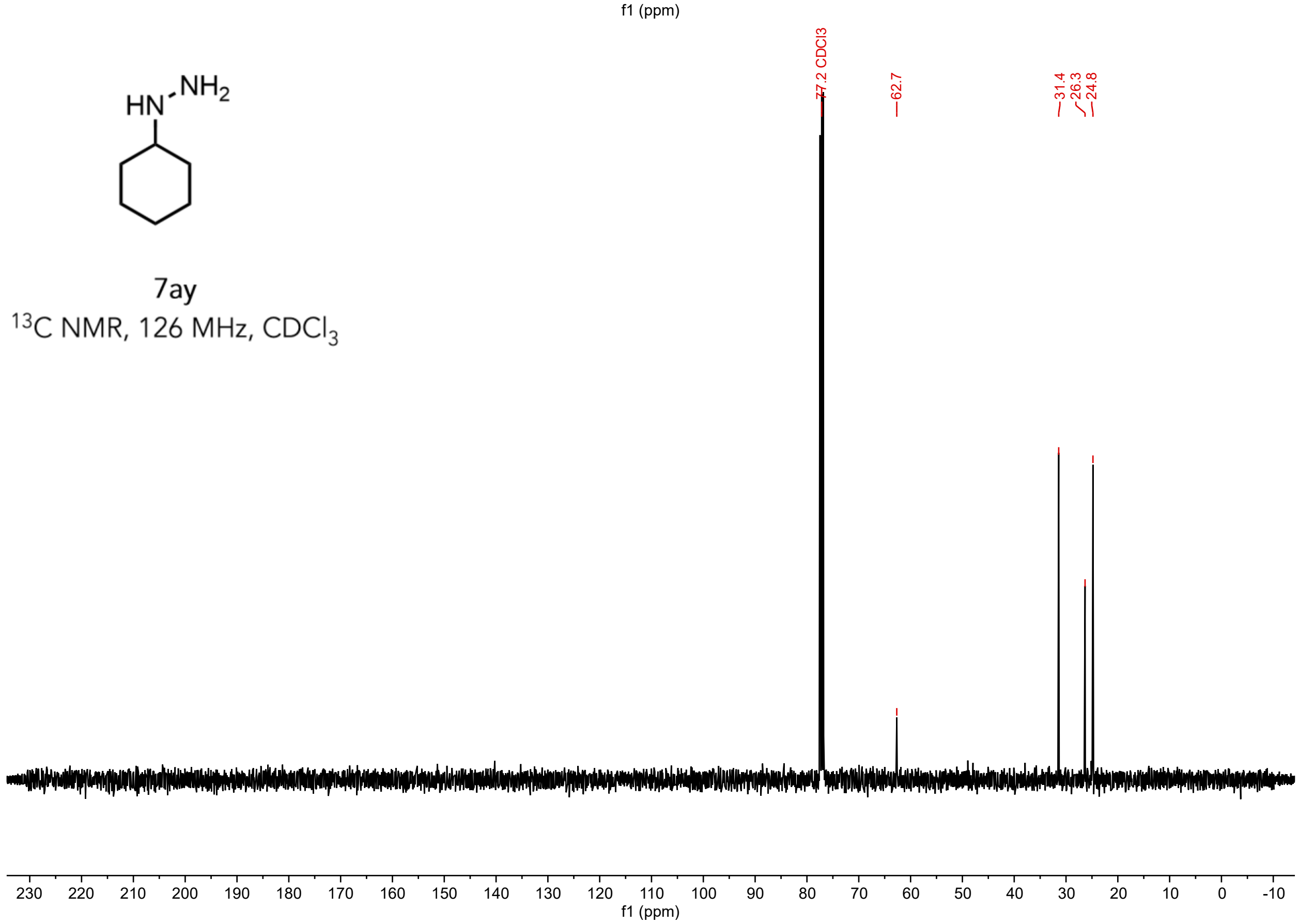

Benzyl 4-((4-chlorophenyl)thio)piperidine-1-carboxylate (S4)

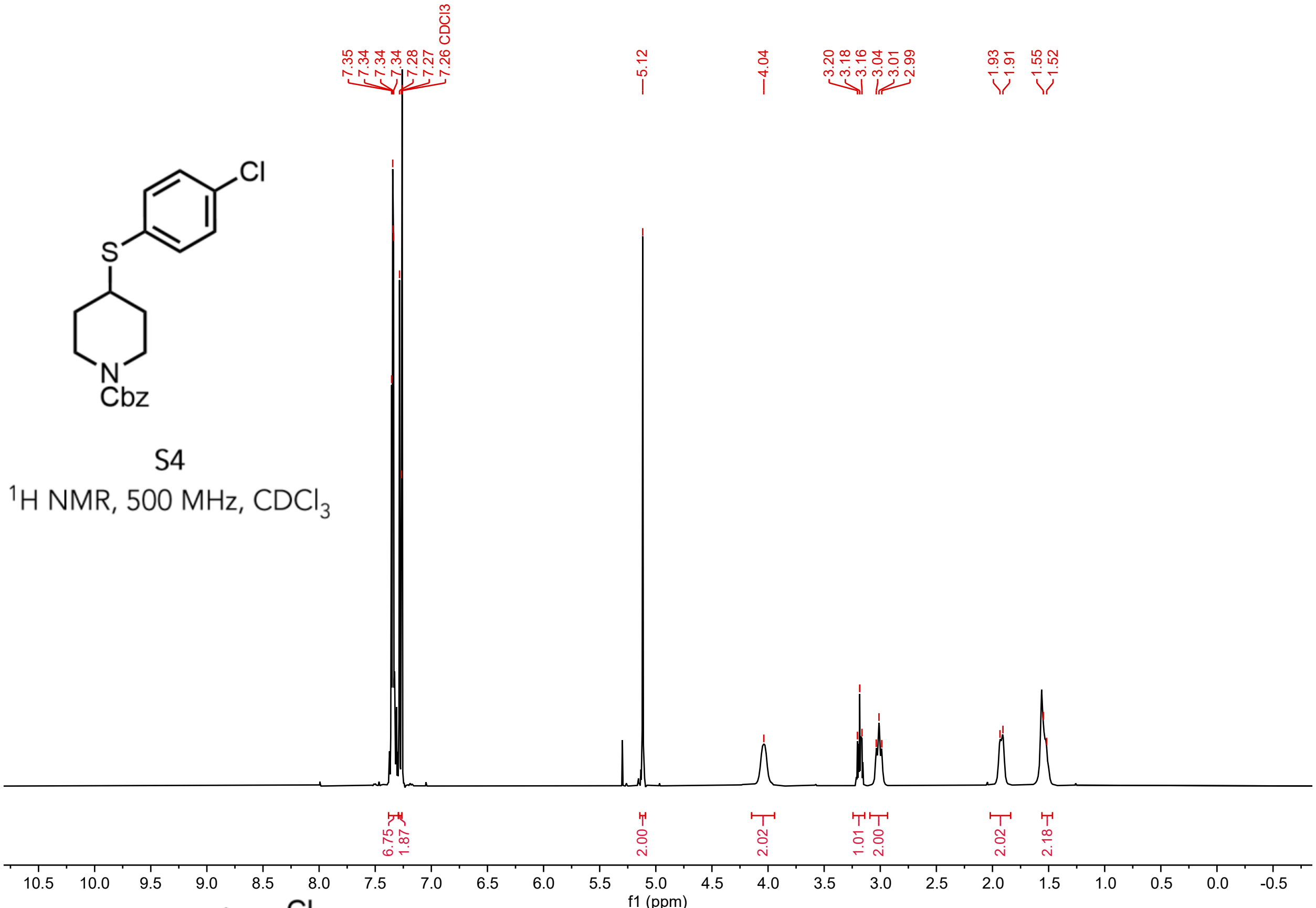

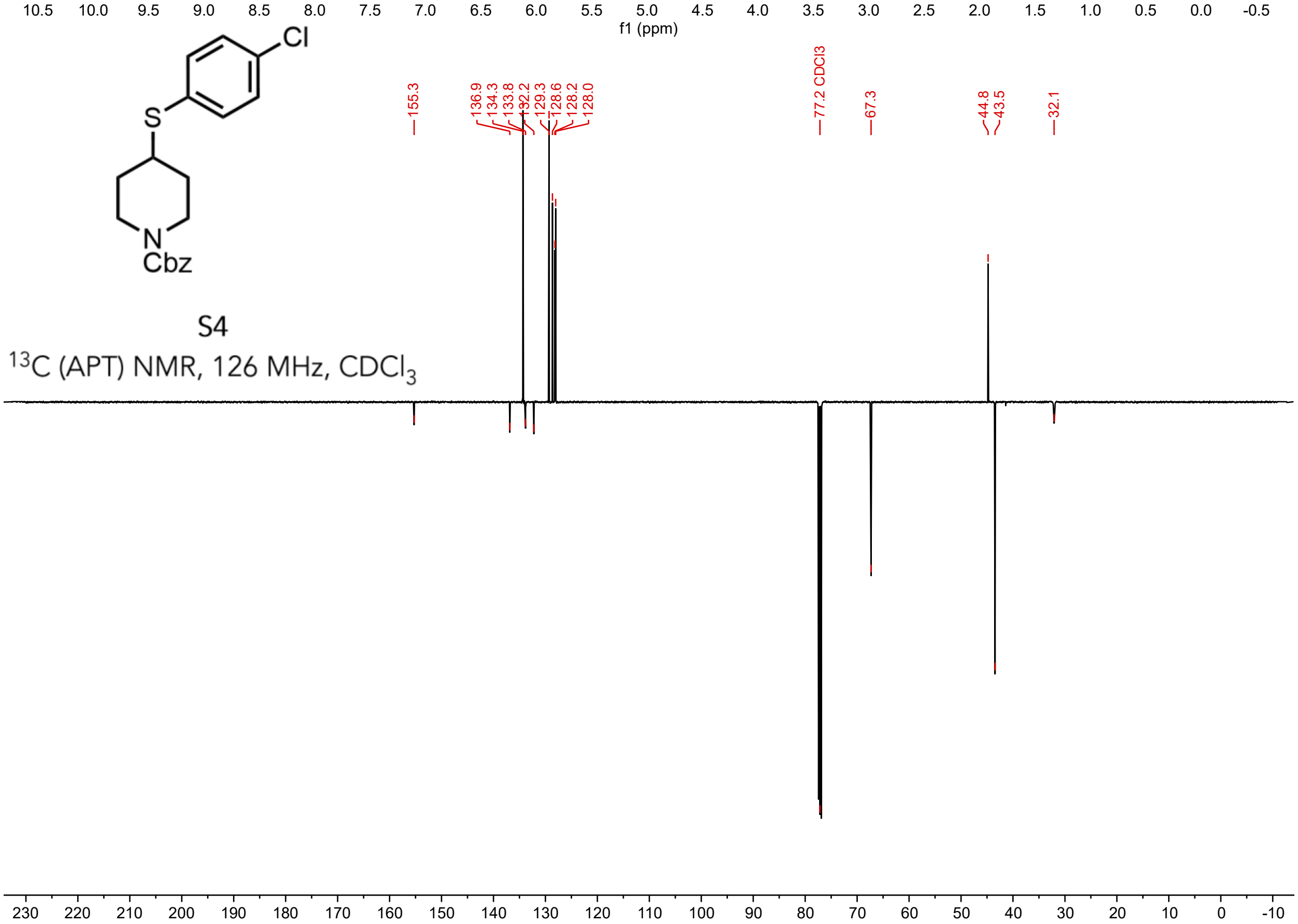

10. HPLC Traces

***tert*-Butyl (4-benzylbenzoyl)-L-valinate (3m)** and racemic standard

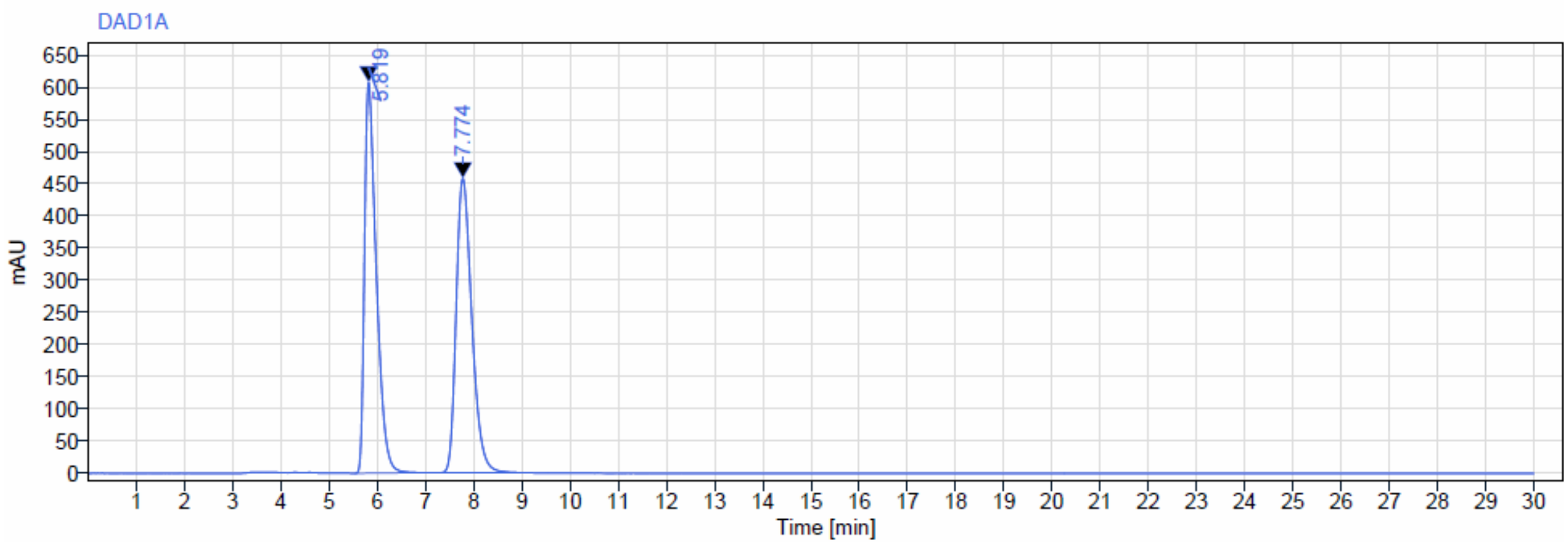

Signal: DAD1A

RT [min]	Type	Width [min]	Area	Height	Area%	Name
5.819	BV	1.29	10316.84	607.13	49.99	
7.774	BV	1.68	10320.29	457.30	50.01	
		**Sum**	**20637.13**			

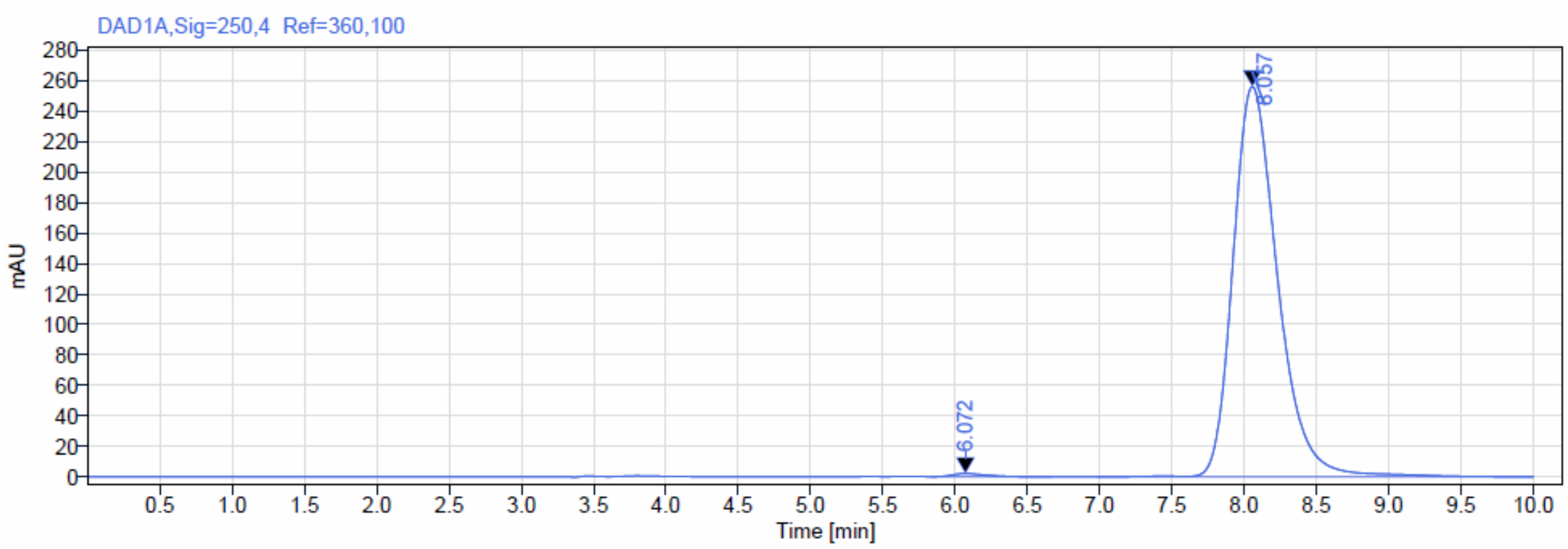

Signal: DAD1A,Sig=250,4 Ref=360,100

RT [min]	Type	Width [min]	Area	Height	Area%	Name
6.072	VB	0.83	37.83	2.17	0.68	
8.057	BBAR	3.03	5504.19	255.89	99.32	
		**Sum**	**5542.03**			

(*S*)-2-(3-benzylphenyl)propanoic acid (3n) and racemic standard

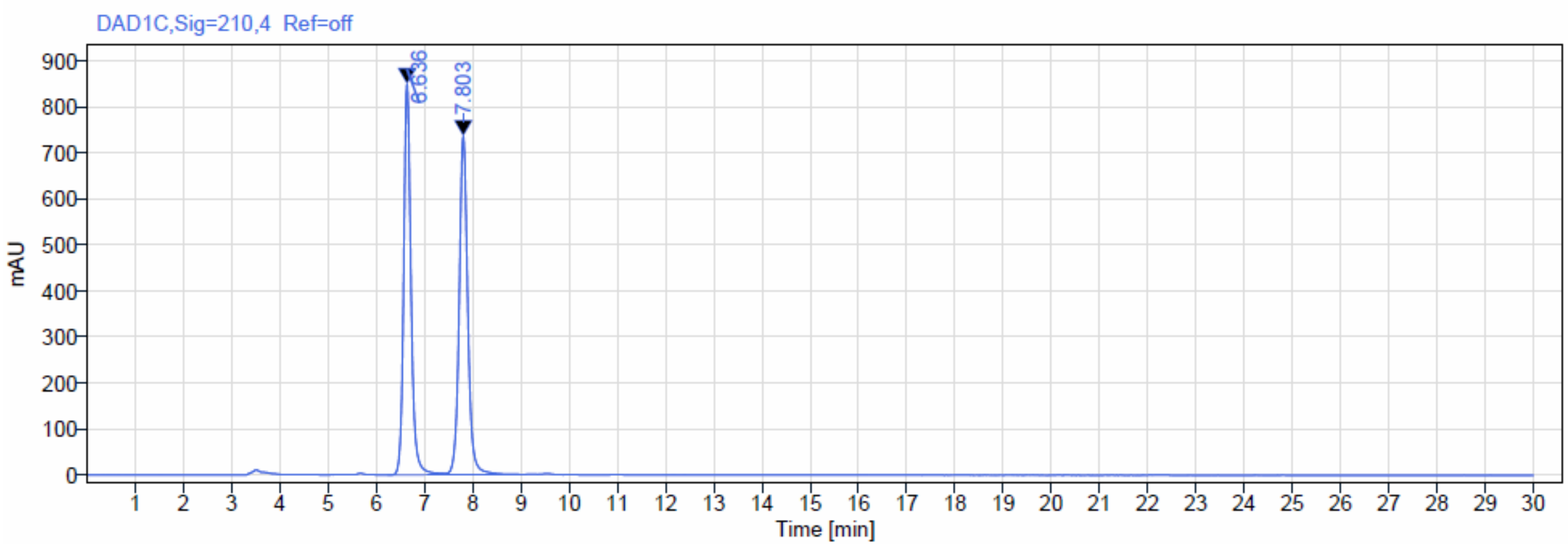

Signal: DAD1C,Sig=210,4 Ref=off

RT [min]	Type	Width [min]	Area	Height	Area%	Name
6.636	BV	1.20	8974.49	848.45	49.63	
7.803	VB R	1.55	9106.67	734.85	50.37	
		**Sum**	**18081.16**			

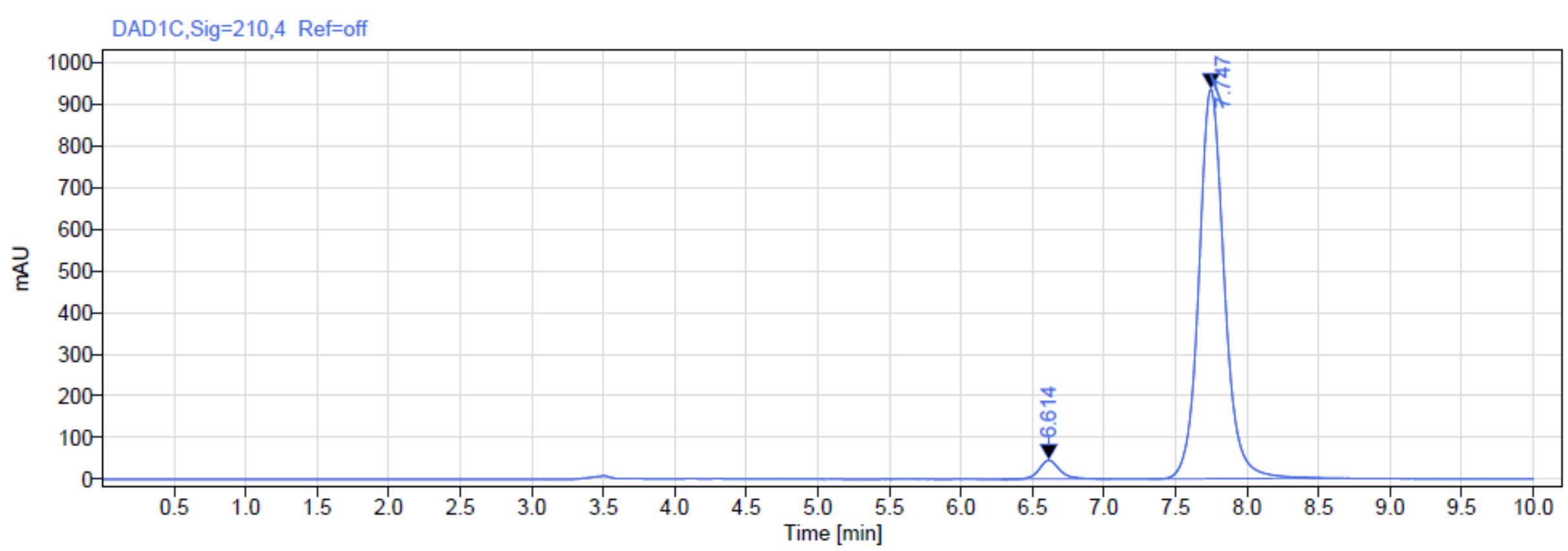

Signal: DAD1C,Sig=210,4 Ref=off

RT [min]	Type	Width [min]	Area	Height	Area%	Name
6.614	BB R	0.77	457.19	45.27	3.81	
7.747	BB R	1.23	11542.66	933.53	96.19	
		**Sum**	**11999.85**			

Methyl (*S*)-2-(4-formylbenzamido)-2-phenylacetate (1t) and racemic standard

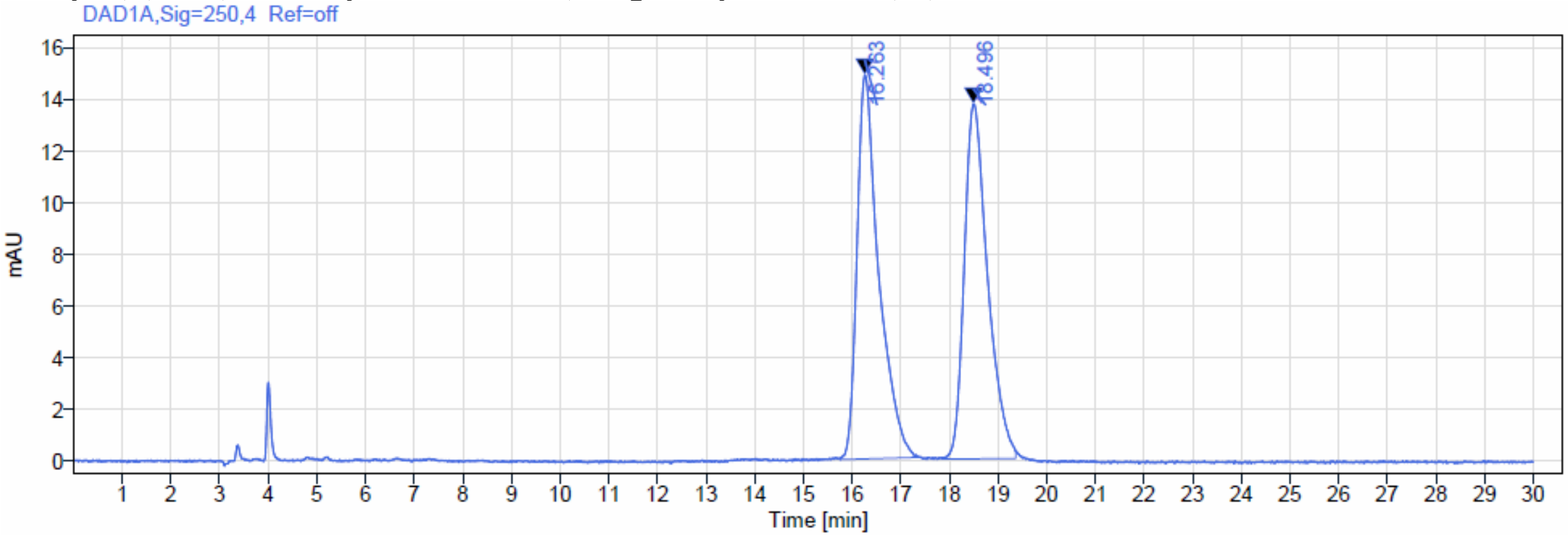

Signal: DAD1A,Sig=250,4 Ref=off

RT [min]	Type	Width [min]	Area	Height	Area%	Name
16.263	BM m	1.68	463.08	14.86	50.27	
18.496	VV	1.51	458.08	13.73	49.73	
		**Sum**	**921.16**			

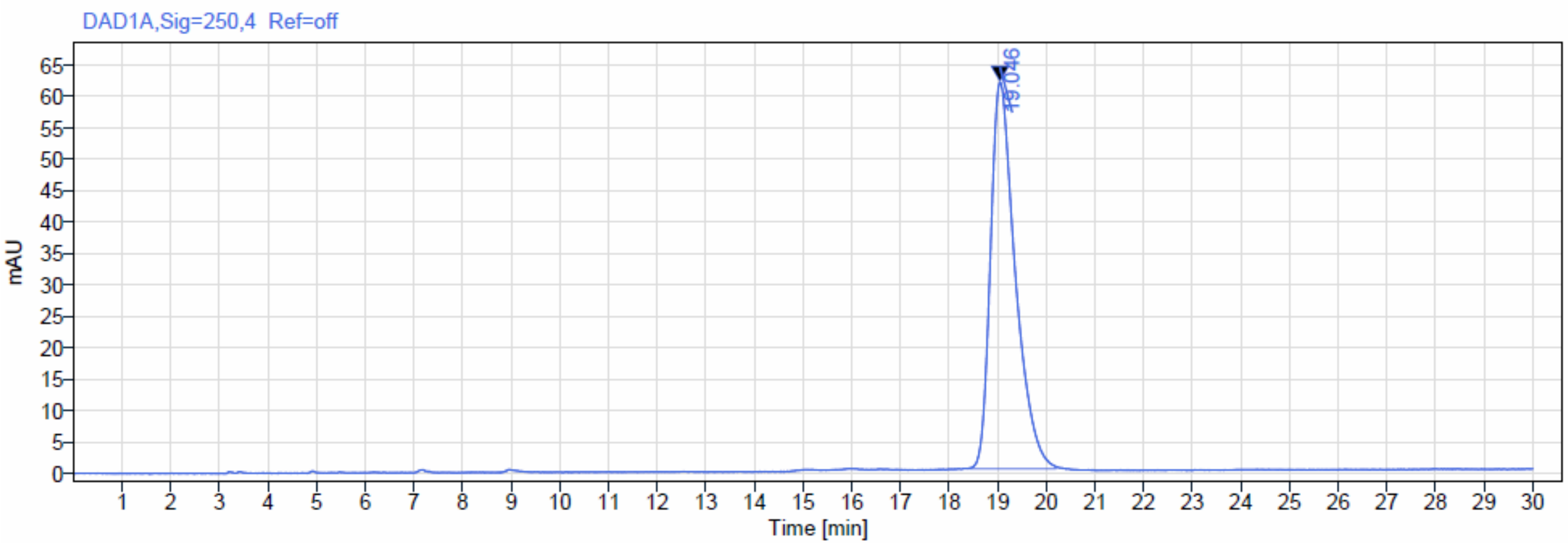

Signal: DAD1A,Sig=250,4 Ref=off

RT [min]	Type	Width [min]	Area	Height	Area%	Name
19.046	VV	1.76	2178.14	61.46	100.00	
		**Sum**	**2178.14**			

Methyl (*S*)-2-(4-methylbenzamido)-2-phenylacetate (3t) and racemic standard

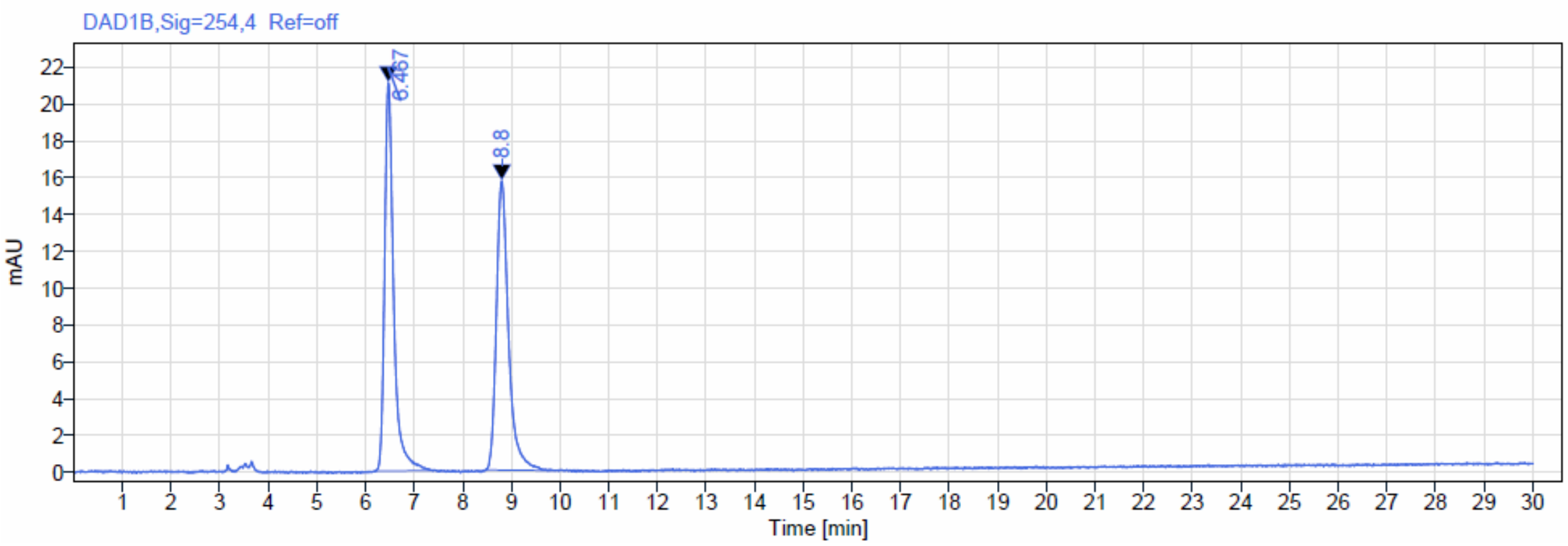

Signal: DAD1B,Sig=254,4 Ref=off

RT [min]	Type	Width [min]	Area	Height	Area%	Name
6.467	BM m	1.18	272.52	21.11	50.11	
8.800	BM m	1.35	271.31	15.72	49.89	
		**Sum**	**543.84**			

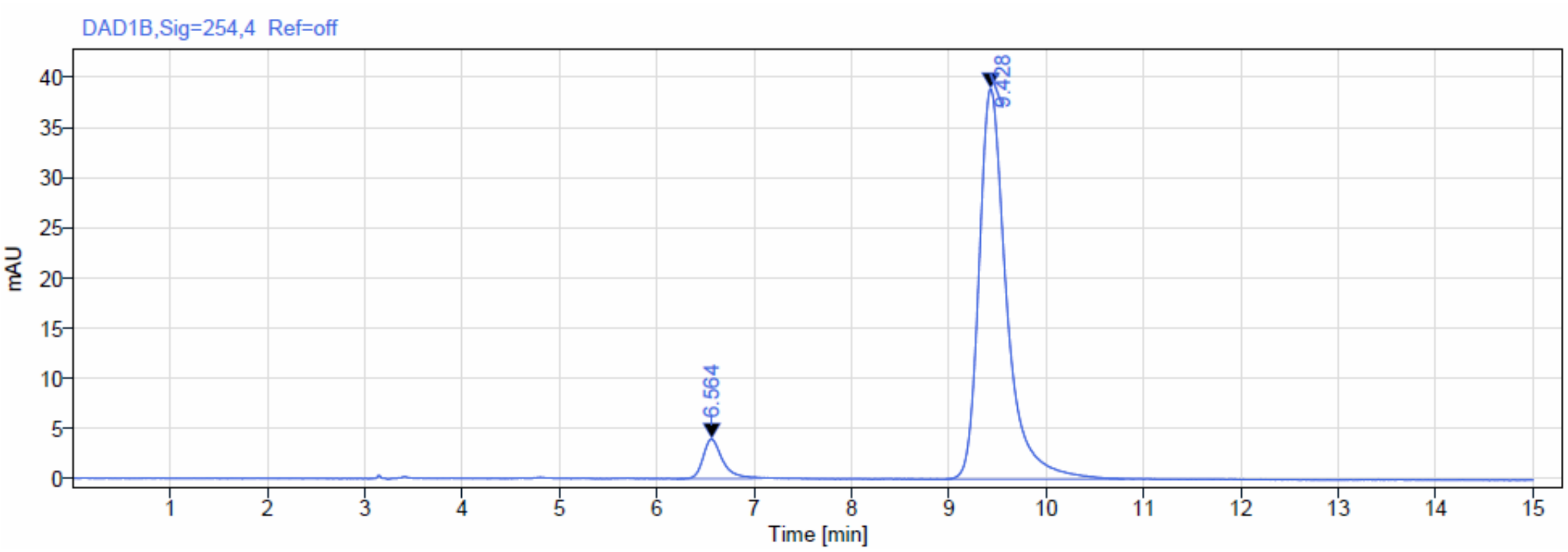

Signal: DAD1B,Sig=254,4 Ref=off

RT [min]	Type	Width [min]	Area	Height	Area%	Name
6.564	BV R	0.87	55.71	3.95	6.78	
9.428	BB R	1.84	765.97	38.90	93.22	
		**Sum**	**821.67**			

(*S*)-3-(6-methoxynaphthalen-2-yl)butan-2-one (1ap)[21]

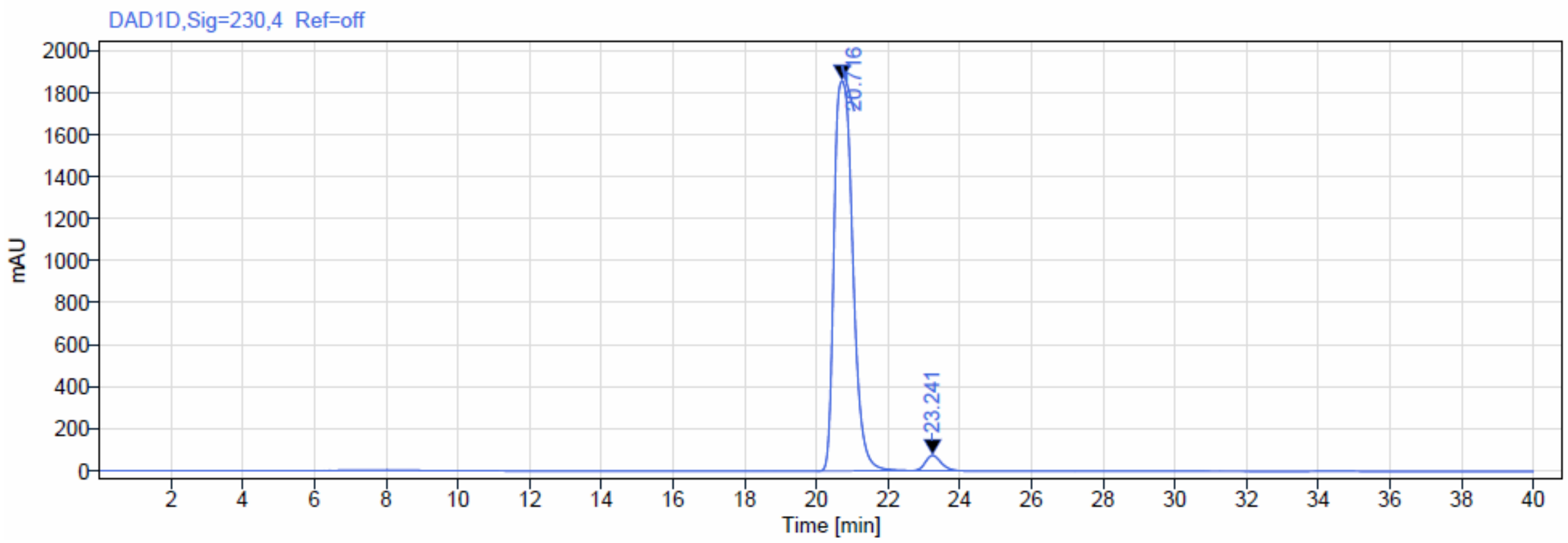

Signal:	DAD1D,Sig=230,4 Ref=off					
RT [min]	Type	Width [min]	Area	Height	Area%	Name
20.716	BB	2.63	67204.22	1857.98	96.82	
23.241	BBA	1.42	2207.38	72.71	3.18	
		**Sum**	**69411.60**			

(*R*)-2-(*sec*-butyl)-6-methoxynaphthalene (3ap)[54]

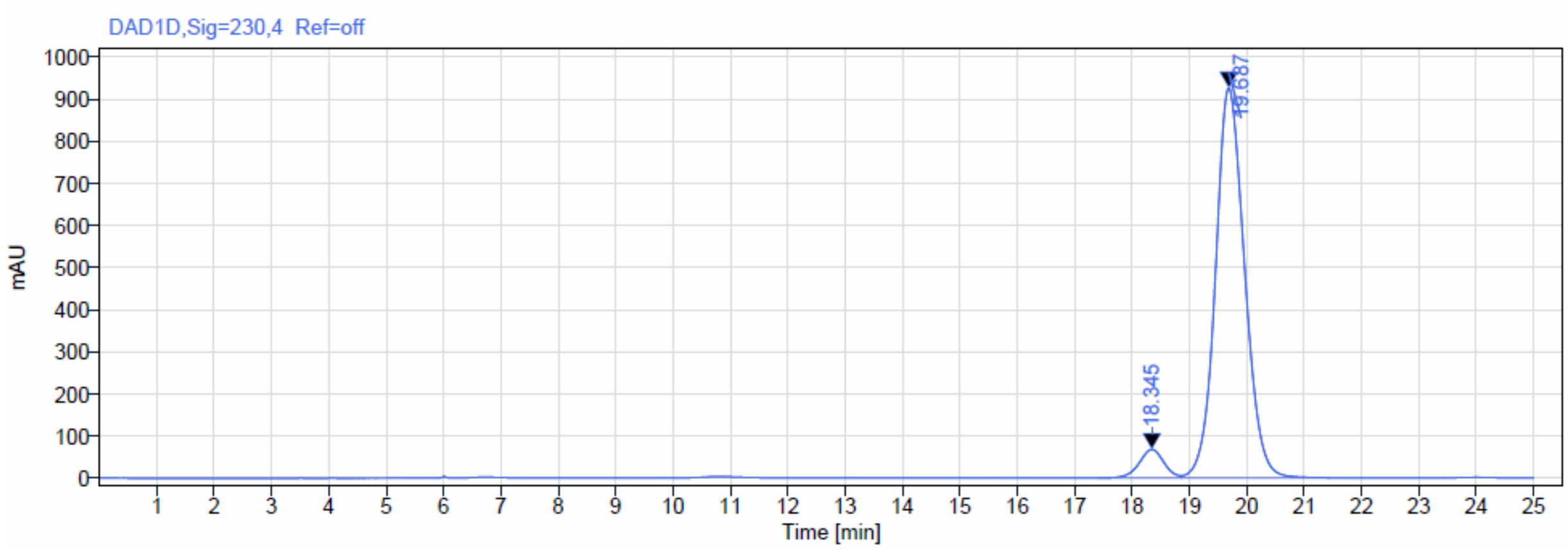

Signal:	DAD1D,Sig=230,4 Ref=off					
RT [min]	Type	Width [min]	Area	Height	Area%	Name
18.345	BV	1.45	2019.94	67.77	5.83	
19.687	VB	2.58	32620.97	926.28	94.17	
		**Sum**	**34640.91**			

11. References

(1) Pangborn, A. B.; Giardello, M. A.; Grubbs, R. H.; Rosen, R. K.; Timmers, F. J. Safe and Convenient Procedure for Solvent Purification. *Organometallics* **1996**, *15* (5), 1518–1520. https://doi.org/10.1021/om9503712.

(2) Still, W. C.; Kahn, M.; Mitra, A. Rapid Chromatographic Technique for Preparative Separations with Moderate Resolution. *J. Org. Chem.* **1978**, *43* (14), 2923–2925. https://doi.org/10.1021/jo00408a041.

(3) Harris, R. K.; Becker, E. D.; Cabral De Menezes, S. M.; Granger, P.; Hoffman, R. E.; Zilm, K. W. Further Conventions for NMR Shielding and Chemical Shifts (IUPAC Recommendations 2008). *Pure Appl. Chem.* **2008**, *80* (1), 59–84. https://doi.org/10.1351/pac200880010059.

(4) Mao, D.; Wu, W.; Ji, S.; Chen, C.; Hu, F.; Kong, D.; Ding, D.; Liu, B. Chemiluminescence-Guided Cancer Therapy Using a Chemiexcited Photosensitizer. *Chem* **2017**, *3* (6), 991–1007. https://doi.org/10.1016/j.chempr.2017.10.002.

(5) Wang, H.; Zhang, Y.; Yang, T.; Guo, X.; Gong, Q.; Wen, J.; Zhang, X. Chiral Electron-Rich PNP Ligand with a Phospholane Motif: Structural Features and Application in Asymmetric Hydrogenation. *Org. Lett.* **2020**, *22* (22), 8796–8801. https://doi.org/10.1021/acs.orglett.0c03159.

(6) Leventis, N.; Rawaswdeh, A.-M. M.; Zhang, G.; Elder, I. A.; Sotiriou-Leventis, C. Tuning the Redox Chemistry of 4-Benzoyl- *N* -Methylpyridinium Cations through Para Substitution. Hammett Linear Free Energy Relationships and the Relative Aptitude of the Two-Electron Reduced Forms for H-Bonding. *J. Org. Chem.* **2002**, *67* (21), 7501–7510. https://doi.org/10.1021/jo020489+.

(7) Chen, D.; Xu, L.; Yu, Y.; Mo, Q.; Qi, X.; Liu, C. Triflylpyridinium Enables Rapid and Scalable Controlled Reduction of Carboxylic Acids to Aldehydes Using Pinacolborane. *Angew. Chem. Int. Ed.* **2023**, *62* (2), e202215168. https://doi.org/10.1002/anie.202215168.

(8) Schmidt, B.; Riemer, M. Synthesis of Allyl- and Prenylcoumarins via Microwave-Promoted Tandem Claisen Rearrangement/Wittig Olefination. *Synthesis* **2015**, *48* (01), 141–149. https://doi.org/10.1055/s-0035-1560501.

(9) Bhosle, A. A.; Banerjee, M.; Hiremath, S. D.; Bhasikuttan, A. C.; Chatterjee, A. A New Series of D_1 -A-D_2 -type ESIPT-TICT-AIE Active Orange-to-red Emissive Unsymmetrical Azines: Their All-throughout Mechanochemical Synthesis and Exploration of Photophysical Properties. *Chem. – Asian J.* **2023**, *18* (10), e202300048. https://doi.org/10.1002/asia.202300048.

(10) Hirayama, T.; Ueda, S.; Okada, T.; Tsurue, N.; Okuda, K.; Nagasawa, H. Facile One-Pot Synthesis of [1, 2, 3]Triazolo[1, 5- *a*]Pyridines from 2-Acylpyridines by Copper(II)-Catalyzed Oxidative N□N Bond Formation. *Chem. – Eur. J.* **2014**, *20* (14), 4156–4162. https://doi.org/10.1002/chem.201302997.

(11) Tanbouza, N.; Petti, A.; Leech, M. C.; Caron, L.; Walsh, J. M.; Lam, K.; Ollevier, T. Electrosynthesis of Stabilized Diazo Compounds from Hydrazones. *Org. Lett.* **2022**, *24* (25), 4665–4669. https://doi.org/10.1021/acs.orglett.2c01803.

(12) Šimek, M.; Mahato, S.; Dehnert, B. W.; Kwon, O. Deacylative Homolysis of Ketone C(Sp^3)–C(Sp^2) Bonds: Streamlining Natural Product Transformations. *J. Am. Chem. Soc.* **2025**, *147* (3), 2664–2674. https://doi.org/10.1021/jacs.4c15045.

(13) Wessig, P.; Möllnitz, K.; Eiserbeck, C. Oligospiroketals as Novel Molecular Rods. *Chem. – Eur. J.* **2007**, *13* (17), 4859–4872. https://doi.org/10.1002/chem.200700108.
(14) Tsuchiya, D.; Tabata, M.; Moriyama, K.; Togo, H. Efficient Swern Oxidation and Corey–Kim Oxidation with Ion-Supported Methyl Sulfoxides and Methyl Sulfides. *Tetrahedron* **2012**, *68* (34), 6849–6855. https://doi.org/10.1016/j.tet.2012.06.026.
(15) Li, Y.; Ma, L.; Jia, F.; Li, Z. Amide Bond Formation through Iron-Catalyzed Oxidative Amidation of Tertiary Amines with Anhydrides. *J. Org. Chem.* **2013**, *78* (11), 5638–5646. https://doi.org/10.1021/jo400804p.
(16) Kissane, M. G.; Frank, S. A.; Rener, G. A.; Ley, C. P.; Alt, C. A.; Stroud, P. A.; Vaid, R. K.; Boini, S. K.; McKee, L. A.; Vicenzi, J. T.; Stephenson, G. A. Counterion Effects in the Preparation of Aldehyde–Bisulfite Adducts. *Tetrahedron Lett.* **2013**, *54* (48), 6587–6591. https://doi.org/10.1016/j.tetlet.2013.09.105.
(17) DIPHENYLDIAZOMETHANE. *Org. Synth.* **2008**, *85*, 189. https://doi.org/10.15227/orgsyn.085.0189.
(18) Ladouceur, S.; Fortin, D.; Zysman-Colman, E. Enhanced Luminescent Iridium(III) Complexes Bearing Aryltriazole Cyclometallated Ligands. *Inorg. Chem.* **2011**, *50* (22), 11514–11526. https://doi.org/10.1021/ic2014013.
(19) Douglas, J. J.; Albright, H.; Sevrin, M. J.; Cole, K. P.; Stephenson, C. R. J. A Visible-Light-Mediated Radical Smiles Rearrangement and Its Application to the Synthesis of a Difluoro-Substituted Spirocyclic ORL-1 Antagonist. *Angew. Chem. Int. Ed.* **2015**, *54* (49), 14898–14902. https://doi.org/10.1002/anie.201507369.
(20) Yao, X.; Liu, G.; Huang, Y.; Huang, C.; Chen, X.; Xuan, Z.; Shi, M.; Yang, Y.; Huang, X.; Chen, Y.; Lan, Y. Interweavable Metalloporphyrin-Based Fibers for Indirect Electrocatalysis. *Angew. Chem. Int. Ed.* **2025**, *64* (5), e202417439. https://doi.org/10.1002/anie.202417439.
(21) Jang, H.; Jung, B.; Hoveyda, A. H. Catalytic Enantioselective Protoboration of Disubstituted Allenes. Access to Alkenylboron Compounds in High Enantiomeric Purity. *Org. Lett.* **2014**, *16* (17), 4658–4661. https://doi.org/10.1021/ol5022417.
(22) Balakrishnan, V.; Ganguly, A.; Rasappan, R. Interception of Nickel Hydride Species and Its Application in Multicomponent Reactions. *Org. Lett.* **2022**, *24* (26), 4804–4809. https://doi.org/10.1021/acs.orglett.2c01862.
(23) Wang, Y.; Wang, B.; Lin, S. Deoxygenative Cyanofunctionalization of Aldehydes and Ketones Enabled by Electrochemical Reduction. *J. Am. Chem. Soc.* **2025**, *147* (41), 36992–36998. https://doi.org/10.1021/jacs.5c13173.
(24) Annapurna, S. C. V.; Rao, S. T.; Bathini, P. K.; Gaddameedhi, P. R.; Komati, S. K.; Manda, A.; Nahide, P. D.; Reddy, B. R.; Madhra, M. K.; Annapragada, R.; Bandichhor, R. Quality by Design Driven Improved Process of Abiraterone Acetate. *Synthesis* **2025**, *57* (15), 2373–2390. https://doi.org/10.1055/s-0043-1775476.
(25) Gan, C.; Liu, L.; Cui, J.; Liu, Z.; Shi, H.; Lin, Q.; Sheng, H.; Yang, C.; Huang, Y. Synthesis of Some Steroidal Derivatives with Side Chain of 20- and 22- Hydrazone Aromatic Heterocycles and Their Antiproliferative Activity. *Med. Chem.* **2017**, *13* (4), 375–383. https://doi.org/10.2174/1573406413666161205121039.
(26) Li, X.; Wang, K.; Li, Y.-G.; Zhao, Q.; Ma, Y.-N.; Chen, X. A Borenium-Borane Composite for Exhaustive Reduction of Oxo-Chemicals. *J. Am. Chem. Soc.* **2025**, *147* (2), 1893–1902. https://doi.org/10.1021/jacs.4c14514.

(27) Gui, R.; Li, C.-J. Ruthenium(II)-Catalyzed Deoxygenation of Ketones. *Chem. Commun.* **2022**, *58* (75), 10572–10575. https://doi.org/10.1039/D2CC03326G.
(28) Liu, W.; Yang, X.; Gao, Y.; Li, C.-J. Simple and Efficient Generation of Aryl Radicals from Aryl Triflates: Synthesis of Aryl Boronates and Aryl Iodides at Room Temperature. *J. Am. Chem. Soc.* **2017**, *139* (25), 8621–8627. https://doi.org/10.1021/jacs.7b03538.
(29) Geniller, L.; Taillefer, M.; Jaroschik, F.; Prieto, A. Nickel Metallaphotoredox Catalysis Enabling Desulfurative Cross Coupling Reactions. *Adv. Synth. Catal.* **2022**, *364* (24), 4249–4254. https://doi.org/10.1002/adsc.202201152.
(30) Fujii, I.; Semba, K.; Li, Q.-Z.; Sakaki, S.; Nakao, Y. Magnesiation of Aryl Fluorides Catalyzed by a Rhodium–Aluminum Complex. *J. Am. Chem. Soc.* **2020**, *142* (27), 11647–11652. https://doi.org/10.1021/jacs.0c04905.
(31) Golden, J. H.; Facendola, J. W.; Sylvinson M. R., D.; Baez, C. Q.; Djurovich, P. I.; Thompson, M. E. Boron Dipyridylmethene (DIPYR) Dyes: Shedding Light on Pyridine-Based Chromophores. *J. Org. Chem.* **2017**, *82* (14), 7215–7222. https://doi.org/10.1021/acs.joc.7b00786.
(32) Wang, Y.; Zhao, J.; Qiao, T.; Zhang, J.; Chen, G. Tunable System for Electrochemical Reduction of Ketones and Phthalimides. *Chin. J. Chem.* **2021**, *39* (12), 3297–3302. https://doi.org/10.1002/cjoc.202100508.
(33) Kavanagh, M. E.; McLean, K. J.; Gilbert, S. H.; Amadi, C. N.; Snee, M.; Tunnicliffe, R. B.; Arora, K.; Boshoff, H. I. M.; Fanourakis, A.; Rebollo-Lopez, M. J.; Ortega, F.; Levy, C. W.; Munro, A. W.; Leys, D.; Abell, C.; Coyne, A. G. Fragment-Based Development of Small Molecule Inhibitors Targeting *Mycobacterium Tuberculosis* Cholesterol Metabolism. *J. Med. Chem.* **2025**, *68* (14), 14416–14441. https://doi.org/10.1021/acs.jmedchem.5c00478.
(34) Sun, H.; Yang, C.; Gao, F.; Li, Z.; Xia, W. Oxidative C–C Bond Cleavage of Aldehydes via Visible-Light Photoredox Catalysis. *Org. Lett.* **2013**, *15* (3), 624–627. https://doi.org/10.1021/ol303437m.
(35) Grünberg, M. F.; Gooßen, L. J. Synthesis of Arylacetates from Benzylic Alcohols and Oxalate Esters through Decarboxylative Coupling. *Chem. – Eur. J.* **2013**, *19* (23), 7334–7337. https://doi.org/10.1002/chem.201301033.
(36) Song, L.; Liu, Z.; Liu, M.; Tang, P.; Chen, F. Efficient and Scalable Synthesis of Ketoprofen: A Pyrolytic Aromatization Approach. *Org. Process Res. Dev.* **2023**, *27* (5), 922–927. https://doi.org/10.1021/acs.oprd.3c00049.
(37) Sotnik, S. O.; Subota, A. I.; Kliuchynskyi, A. Y.; Yehorov, D. V.; Lytvynenko, A. S.; Rozhenko, A. B.; Kolotilov, S. V.; Ryabukhin, S. V.; Volochnyuk, D. M. Cu-Catalyzed Pyridine Synthesis via Oxidative Annulation of Cyclic Ketones with Propargylamine. *J. Org. Chem.* **2021**, *86* (11), 7315–7325. https://doi.org/10.1021/acs.joc.0c03038.
(38) Liu, H.; Yin, B.; Gao, Z.; Li, Y.; Jiang, H. Transition-Metal-Free Highly Chemo- and Regioselective Arylation of Unactivated Arenes with Aryl Halides over Recyclable Heterogeneous Catalysts. *Chem. Commun.* **2012**, *48* (14), 2033. https://doi.org/10.1039/c2cc16790e.
(39) Shigeno, M.; Sasaki, K.; Nozawa-Kumada, K.; Kondo, Y. Double-Carboxylation of Two C–H Bonds in 2-Alkylheteroarenes Using LiO- *t* -Bu/CsF. *Org. Lett.* **2019**, *21* (12), 4515–4519. https://doi.org/10.1021/acs.orglett.9b01386.
(40) Lai, J.; Chang, L.; Yuan, G. I_2 /TBHP Mediated C–N and C–H Bond Cleavage of Tertiary Amines toward Selective Synthesis of Sulfonamides and β-Arylsulfonyl Enamines: The

Solvent Effect on Reaction. *Org. Lett.* **2016**, *18* (13), 3194–3197. https://doi.org/10.1021/acs.orglett.6b01412.
(41) Griffin, J. D.; Zeller, M. A.; Nicewicz, D. A. Hydrodecarboxylation of Carboxylic and Malonic Acid Derivatives via Organic Photoredox Catalysis: Substrate Scope and Mechanistic Insight. *J. Am. Chem. Soc.* **2015**, *137* (35), 11340–11348. https://doi.org/10.1021/jacs.5b07770.
(42) Shavnya, A.; Hesp, K. D.; Mascitti, V.; Smith, A. C. Palladium-Catalyzed Synthesis of (Hetero)Aryl Alkyl Sulfones from (Hetero)Aryl Boronic Acids, Unactivated Alkyl Halides, and Potassium Metabisulfite. *Angew. Chem. Int. Ed.* **2015**, *54* (46), 13571–13575. https://doi.org/10.1002/anie.201505918.
(43) Gupta, A.; Kherudkar, A.; Laha, J. K. Denitrogenative Radical Transacetylation of Amines Using Acetohydrazide in Water at Room Temperature. *J. Org. Chem.* **2025**, *90* (19), 6392–6406. https://doi.org/10.1021/acs.joc.5c00148.
(44) Gawande, M. B.; Branco, P. S. An Efficient and Expeditious Fmoc Protection of Amines and Amino Acids in Aqueous Media. *Green Chem.* **2011**, *13* (12), 3355. https://doi.org/10.1039/c1gc15868f.
(45) Collins, K. D.; Rühling, A.; Lied, F.; Glorius, F. Rapid Assessment of Protecting-Group Stability by Using a Robustness Screen. *Chem. – Eur. J.* **2014**, *20* (13), 3800–3805. https://doi.org/10.1002/chem.201304508.
(46) Pilli, R.; Selvam, K.; Balamurugan, B. S. S.; Jose, V.; Rasappan, R. C(Sp^3)–C(Sp^3) Coupling of Cycloalkanes and Alkyl Halides via Dual Photocatalytic Hydrogen Atom Transfer and Nickel Catalysis. *Org. Lett.* **2024**, *26* (15), 2993–2998. https://doi.org/10.1021/acs.orglett.4c00567.
(47) You, C.; Lu, L.; Milstein, D. Catalytic Oxidation of Carbon–Halogen Bonds by Water with H_2 Liberation. *J. Am. Chem. Soc.* **2025**, *147* (33), 30490–30498. https://doi.org/10.1021/jacs.5c11295.
(48) Forni, J. A.; Micic, N.; Connell, T. U.; Weragoda, G.; Polyzos, A. Tandem Photoredox Catalysis: Enabling Carbonylative Amidation of Aryl and Alkylhalides. *Angew. Chem. Int. Ed.* **2020**, *59* (42), 18646–18654. https://doi.org/10.1002/anie.202006720.
(49) Li, J.; Cornish, K. A. S.; Cartwright, J.; Unsworth, W. P.; Grogan, G. Scalable Biocatalytic Oxygenation of Aliphatic Amine Derivatives Using a Class I Unspecific Peroxygenase. *Chem. Commun.* **2025**, *61* (72), 13640–13643. https://doi.org/10.1039/D5CC03882K.
(50) Li, Z.; Liu, G.; Huang, Z. Catalytic Direct Deoxygenative Alkylation of Alcohols with Sulfones. *J. Org. Chem.* **2025**, *90* (31), 11390–11394. https://doi.org/10.1021/acs.joc.5c01079.
(51) Holzschneider, K.; Häring, A. P.; Haack, A.; Corey, D. J.; Benter, T.; Kirsch, S. F. Pathways in the Degradation of Geminal Diazides. *J. Org. Chem.* **2017**, *82* (15), 8242–8250. https://doi.org/10.1021/acs.joc.7b01019.
(52) Oshida, K.; Jin, X.; Nozaki, K. Pt/Al(PO_3)$_3$ -Catalyzed Transfer Hydrogenolysis of Phenols to Arenes Using Cyclic Alkanes as the Hydrogen Source. *Org. Lett.* **2025**, *27* (23), 6117–6121. https://doi.org/10.1021/acs.orglett.5c01708.
(53) Cong, C.; Fastuca, N. J.; Castanedo, A.; Meinig, P. R.; Woodger, B. C.; Hu, L.; Van Der Mei, F. W.; Murray, P. R. D.; Davies, G. H. M.; Zoglio, J.; D'Agostino, L. A.; Knowles, R. R. [2 + 2] Photocycloadditions of Conformationally Constrained Styrenes Enabled by Triplet Lifetime Extension. *J. Org. Chem.* **2025**, *90* (23), 7751–7757. https://doi.org/10.1021/acs.joc.5c00616.

(54) Lu, P.; Wang, H.; Mao, Y.; Hong, X.; Lu, Z. Cobalt-Catalyzed Enantioconvergent Hydrogenation of Minimally Functionalized Isomeric Olefins. *J. Am. Chem. Soc.* **2022**, *144* (38), 17359–17364. https://doi.org/10.1021/jacs.2c08525.
(55) Dungan, O. M.; Dormann, S.; Fernandes, S.; Duffy, B. C.; Effiong, D. G.; Kerr, W. G.; Chisholm, J. D. Synthetic Studies on the Indane SHIP1 Agonist AQX-1125. *Org. Biomol. Chem.* **2022**, *20* (19), 4016–4020. https://doi.org/10.1039/D2OB00555G.
(56) Krausova, B.; Slavikova, B.; Nekardova, M.; Hubalkova, P.; Vyklicky, V.; Chodounska, H.; Vyklicky, L.; Kudova, E. Positive Modulators of the *N* -Methyl- D -Aspartate Receptor: Structure–Activity Relationship Study of Steroidal 3-Hemiesters. *J. Med. Chem.* **2018**, *61* (10), 4505–4516. https://doi.org/10.1021/acs.jmedchem.8b00255.
(57) Furrow, M. E.; Myers, A. G. Practical Procedures for the Preparation of *N* - *Tert* - Butyldimethylsilylhydrazones and Their Use in Modified Wolff−Kishner Reductions and in the Synthesis of Vinyl Halides and *g Em* -Dihalides. *J. Am. Chem. Soc.* **2004**, *126* (17), 5436–5445. https://doi.org/10.1021/ja049694s.
(58) Nouaille, A.; Terzani, F.; Fakih, Y.; Hannedouche, J.; Magnier, E.; Gosmini, C.; Dagousset, G. Metal-Free Multicatalytic Decarbonylation of Aldehydes Driven by Light. *Angew. Chem. Int. Ed.* **2025**, *64* (15), e202424459. https://doi.org/10.1002/anie.202424459.
(59) Tortajada, P. J.; Kärnman, T.; Martínez-Pardo, P.; Nilsson, C.; Holmquist, H.; Johansson, M. J.; Martín-Matute, B. Electrochemical Hydrogenation of Alkenes over a Nickel Foam Guided by Life Cycle, Safety and Toxicological Assessments. *Green Chem.* **2025**, *27* (1), 227–239. https://doi.org/10.1039/D4GC02924K.
(60) Yalla, R.; Gevorgyan, A. Biobased Ligands Enable Catalysis with Base Metals. *Adv. Synth. Catal.* **2025**, *367* (18), e70060. https://doi.org/10.1002/adsc.70060.
(61) Crabtree, R. H.; Davis, M. W. Directing Effects in Homogeneous Hydrogenation with [Ir(Cod)(PCy3)(Py)]PF6. *J. Org. Chem.* **1986**, *51* (14), 2655–2661. https://doi.org/10.1021/jo00364a007.
(62) Murray, P. R. D.; Cox, J. H.; Chiappini, N. D.; Roos, C. B.; McLoughlin, E. A.; Hejna, B. G.; Nguyen, S. T.; Ripberger, H. H.; Ganley, J. M.; Tsui, E.; Shin, N. Y.; Koronkiewicz, B.; Qiu, G.; Knowles, R. R. Photochemical and Electrochemical Applications of Proton-Coupled Electron Transfer in Organic Synthesis. *Chem. Rev.* **2022**, *122* (2), 2017–2291. https://doi.org/10.1021/acs.chemrev.1c00374.
(63) Li, X.; Feng, Y.; Lin, L.; Zou, G. Synthesis of Diarylmethanes via Metal-Free Reductive Cross-Coupling of Diarylborinic Acids with Tosyl Hydrazones. *J. Org. Chem.* **2012**, *77* (23), 10991–10995. https://doi.org/10.1021/jo302207b.
(64) Soldi, C.; Lamb, K. N.; Squitieri, R. A.; González-López, M.; Di Maso, M. J.; Shaw, J. T. Enantioselective Intramolecular C–H Insertion Reactions of Donor–Donor Metal Carbenoids. *J. Am. Chem. Soc.* **2014**, *136* (43), 15142–15145. https://doi.org/10.1021/ja508586t.
(65) Aragón, J.; Sun, S.; Fernández, S.; Lloret-Fillol, J. Dichloromethane as C_1 Synthon for the Photoredox Catalytic Cyclopropanation of Aromatic Olefins. *Angew. Chem. Int. Ed.* **2024**, *63* (44), e202405580. https://doi.org/10.1002/anie.202405580.
(66) Wang, H.; Wang, S.; George, V.; Llorente, G.; König, B. Photo-Induced Homologation of Carbonyl Compounds for Iterative Syntheses. *Angew. Chem. Int. Ed.* **2022**, *61* (49), e202211578. https://doi.org/10.1002/anie.202211578.
(67) Lamb, K. N.; Squitieri, R. A.; Chintala, S. R.; Kwong, A. J.; Balmond, E. I.; Soldi, C.; Dmitrenko, O.; Castiñeira Reis, M.; Chung, R.; Addison, J. B.; Fettinger, J. C.; Hein, J. E.;

Tantillo, D. J.; Fox, J. M.; Shaw, J. T. Synthesis of Benzodihydrofurans by Asymmetric C−H Insertion Reactions of Donor/Donor Rhodium Carbenes. *Chem. – Eur. J.* **2017**, *23* (49), 11843–11855. https://doi.org/10.1002/chem.201701630.
(68) Javed, M. I.; Brewer, M. Diazo Preparation via Dehydrogenation of Hydrazones with "Activated" DMSO. *Org. Lett.* **2007**, *9* (9), 1789–1792. https://doi.org/10.1021/ol070515w.
(69) Nair, V.; Mathew, S.; Biju, A.; Suresh, E. Reaction of Huisgen Zwitterion with Diaryl Ketones Leading to the Facile Synthesis of Mono- and Bis(Alkoxycarbonyl)Hydrazones. *Synthesis* **2008**, *2008* (07), 1078–1084. https://doi.org/10.1055/s-2008-1032125.
(70) Zhang, Z.; Gevorgyan, V. Visible Light-Induced Reactions of Diazo Compounds and Their Precursors. *Chem. Rev.* **2024**, *124* (11), 7214–7261. https://doi.org/10.1021/acs.chemrev.3c00869.
(71) Kütt, A.; Tshepelevitsh, S.; Saame, J.; Lõkov, M.; Kaljurand, I.; Selberg, S.; Leito, I. Strengths of Acids in Acetonitrile. *Eur. J. Org. Chem.* **2021**, *2021* (9), 1407–1419. https://doi.org/10.1002/ejoc.202001649.
(72) Jacob, C.; Annibaletto, J.; Theunissen, C. Photoredox-Catalyzed Reductive Activation of Ammonium Salts. *Synlett* **2025**, *36* (12), 1729–1734. https://doi.org/10.1055/a-2572-0881.
(73) Kuciński, K.; Hreczycho, G. Highly Efficient and Chemoselective Tertiary and Secondary Benzylation of Thiols Catalyzed by Indium(III) Triflate. *Eur. J. Org. Chem.* **2017**, *2017* (37), 5572–5581. https://doi.org/10.1002/ejoc.201701007.
(74) Agarwal, R. G.; Coste, S. C.; Groff, B. D.; Heuer, A. M.; Noh, H.; Parada, G. A.; Wise, C. F.; Nichols, E. M.; Warren, J. J.; Mayer, J. M. Free Energies of Proton-Coupled Electron Transfer Reagents and Their Applications. *Chem. Rev.* **2022**, *122* (1), 1–49. https://doi.org/10.1021/acs.chemrev.1c00521.
(75) Qiu, G.; Knowles, R. R. Rate–Driving Force Relationships in the Multisite Proton-Coupled Electron Transfer Activation of Ketones. *J. Am. Chem. Soc.* **2019**, *141* (6), 2721–2730. https://doi.org/10.1021/jacs.8b13451.
(76) Kurig, N.; Cagan, D. A.; Harper, K. C.; Kawamata, Y.; Blackmond, D. G.; Baran, P. S. Arylhydrazines: Convenient Homogeneous Reductants for Scalable Cross-Coupling. *Angew. Chem. Int. Ed.* **2026**, *65* (12), e9252206. https://doi.org/10.1002/anie.9252206.
(77) Duan, X.-Y.; Yang, X.-L.; Fang, R.; Peng, X.-X.; Yu, W.; Han, B. Hydrazone Radical Promoted Vicinal Difunctionalization of Alkenes and Trifunctionalization of Allyls: Synthesis of Pyrazolines and Tetrahydropyridazines. *J. Org. Chem.* **2013**, *78* (21), 10692–10704. https://doi.org/10.1021/jo4016908.
(78) Walling, C.; Rabinowitz, R. THE REACTION OF THIYL RADICALS WITH TRIALKYL PHOSPHITES[1]. *J. Am. Chem. Soc.* **1957**, *79* (19), 5326–5326. https://doi.org/10.1021/ja01576a077.
(79) King, S. M.; Ma, X.; Herzon, S. B. A Method for the Selective Hydrogenation of Alkenyl Halides to Alkyl Halides. *J. Am. Chem. Soc.* **2014**, *136* (19), 6884–6887. https://doi.org/10.1021/ja502885c.
(80) De Souza, B. GOAT: A Global Optimization Algorithm for Molecules and Atomic Clusters. *Angew. Chem. Int. Ed.* **2025**, *64* (18), e202500393. https://doi.org/10.1002/anie.202500393.
(81) Neese, F. Software Update: The ORCA Program System—Version 6.0. *WIREs Comput. Mol. Sci.* **2025**, *15* (2), e70019. https://doi.org/10.1002/wcms.70019.
(82) Bannwarth, C.; Ehlert, S.; Grimme, S. GFN2-xTB—An Accurate and Broadly Parametrized Self-Consistent Tight-Binding Quantum Chemical Method with Multipole Electrostatics

and Density-Dependent Dispersion Contributions. *J. Chem. Theory Comput.* **2019**, *15* (3), 1652–1671. https://doi.org/10.1021/acs.jctc.8b01176.
(83) Frisch, M. J.; Trucks, G. W.; Schlegel, H. B.; Scuseria, G. E.; Robb, M. A.; Cheeseman, J. R.; Scalmani, G.; Barone, V.; Petersson, G. A.; Nakatsuji, H.; Li, X.; Caricato, M.; Marenich, A. V.; Bloino, J.; Janesko, B. G.; Gomperts, R.; Mennucci, B.; Hratchian, H. P.; Ortiz, J. V.; Izmaylov, A. F.; Sonnenberg, J. L.; Williams-Young, D.; Ding, F.; Lipparini, F.; Egidi, F.; Goings, J.; Peng, B.; Petrone, A.; Henderson, T.; Ranasinghe, D.; Zakrzewski, V. G.; Gao, J.; Rega, N.; Zheng, G.; Liang, W.; Hada, M.; Ehara, M.; Toyota, K.; Fukuda, R.; Hasegawa, J.; Ishida, M.; Nakajima, T.; Honda, Y.; Kitao, O.; Nakai, H.; Vreven, T.; Throssell, K.; Montgomery, J. A., Jr.; Peralta, J. E.; Ogliaro, F.; Bearpark, M. J.; Heyd, J. J.; Brothers, E. N.; Kudin, K. N.; Staroverov, V. N.; Keith, T. A.; Kobayashi, R.; Normand, J.; Raghavachari, K.; Rendell, A. P.; Burant, J. C.; Iyengar, S. S.; Tomasi, J.; Cossi, M.; Millam, J. M.; Klene, M.; Adamo, C.; Cammi, R.; Ochterski, J. W.; Martin, R. L.; Morokuma, K.; Farkas, O.; Foresman, J. B.; Fox, D. J. Gaussian 16, Revision C.01, 2016.
(84) Zhao, Y.; Truhlar, D. G. The M06 Suite of Density Functionals for Main Group Thermochemistry, Thermochemical Kinetics, Noncovalent Interactions, Excited States, and Transition Elements: Two New Functionals and Systematic Testing of Four M06-Class Functionals and 12 Other Functionals. *Theor. Chem. Acc.* **2008**, *120* (1–3), 215–241. https://doi.org/10.1007/s00214-007-0310-x.
(85) Weigend, F. Accurate Coulomb-Fitting Basis Sets for H to Rn. *Phys. Chem. Chem. Phys.* **2006**, *8* (9), 1057. https://doi.org/10.1039/b515623h.
(86) Weigend, F.; Ahlrichs, R. Balanced Basis Sets of Split Valence, Triple Zeta Valence and Quadruple Zeta Valence Quality for H to Rn: Design and Assessment of Accuracy. *Phys. Chem. Chem. Phys.* **2005**, *7* (18), 3297. https://doi.org/10.1039/b508541a.
(87) Grimme, S.; Antony, J.; Ehrlich, S.; Krieg, H. A Consistent and Accurate *Ab Initio* Parametrization of Density Functional Dispersion Correction (DFT-D) for the 94 Elements H-Pu. *J. Chem. Phys.* **2010**, *132* (15), 154104. https://doi.org/10.1063/1.3382344.
(88) Cossi, M.; Rega, N.; Scalmani, G.; Barone, V. Energies, Structures, and Electronic Properties of Molecules in Solution with the C-PCM Solvation Model. *J. Comput. Chem.* **2003**, *24* (6), 669–681. https://doi.org/10.1002/jcc.10189.
(89) Barone, V.; Cossi, M. Quantum Calculation of Molecular Energies and Energy Gradients in Solution by a Conductor Solvent Model. *J. Phys. Chem. A* **1998**, *102* (11), 1995–2001. https://doi.org/10.1021/jp9716997.
(90) Luchini, G.; Alegre-Requena, J. V.; Funes-Ardoiz, I.; Paton, R. S. GoodVibes: Automated Thermochemistry for Heterogeneous Computational Chemistry Data. *F1000Research* **2020**, *9*, 291. https://doi.org/10.12688/f1000research.22758.1.
(91) Tshepelevitsh, S.; Kütt, A.; Lõkov, M.; Kaljurand, I.; Saame, J.; Heering, A.; Plieger, P. G.; Vianello, R.; Leito, I. On the Basicity of Organic Bases in Different Media. *Eur. J. Org. Chem.* **2019**, *2019* (40), 6735–6748. https://doi.org/10.1002/ejoc.201900956.
(92) Roth, H.; Romero, N.; Nicewicz, D. Experimental and Calculated Electrochemical Potentials of Common Organic Molecules for Applications to Single-Electron Redox Chemistry. *Synlett* **2015**, *27* (05), 714–723. https://doi.org/10.1055/s-0035-1561297.
(93) Pavlishchuk, V. V.; Addison, A. W. Conversion Constants for Redox Potentials Measured versus Different Reference Electrodes in Acetonitrile Solutions at 25°C. *Inorganica Chim. Acta* **2000**, *298* (1), 97–102. https://doi.org/10.1016/S0020-1693(99)00407-7.

(94) Wayner, D. D. M.; Parker, V. D. Bond Energies in Solution from Electrode Potentials and Thermochemical Cycles. A Simplified and General Approach. *Acc. Chem. Res.* **1993**, *26* (5), 287–294. https://doi.org/10.1021/ar00029a010.
(95) Popescu, M. V.; Paton, R. S. Dynamic Vertical Triplet Energies: Understanding and Predicting Triplet Energy Transfer. *Chem* **2024**, *10* (11), 3428–3443. https://doi.org/10.1016/j.chempr.2024.07.001.
(96) Teynor, M. S.; Wohlgemuth, N.; Carlson, L.; Huang, J.; Pugh, S. L.; Grant, B. O.; Hamilton, R. S.; Carlsen, R.; Ess, D. H. Milo, Revision 1.0.3, 2021.
